# A genome-wide siRNA screen identifies a druggable host pathway essential for the Ebola virus life cycle

**DOI:** 10.1186/s13073-018-0570-1

**Published:** 2018-08-07

**Authors:** Scott Martin, Abhilash I. Chiramel, Marie Luisa Schmidt, Yu-Chi Chen, Nadia Whitt, Ari Watt, Eric C. Dunham, Kyle Shifflett, Shelby Traeger, Anne Leske, Eugen Buehler, Cynthia Martellaro, Janine Brandt, Lisa Wendt, Andreas Müller, Stephanie Peitsch, Sonja M. Best, Jürgen Stech, Stefan Finke, Angela Römer-Oberdörfer, Allison Groseth, Heinz Feldmann, Thomas Hoenen

**Affiliations:** 1 Division of Preclinical Innovation, National Center for Advancing Translational Sciences, National Institutes of Health, 31 Center Drive, Bethesda, MD 20892 USA; 2 Laboratory of Virology, Division of Intramural Research, National Institute for Allergy and Infectious Diseases, National Institutes of Health, 903 S 4th St., Hamilton, MT 59840 USA; 3 Institute of Molecular Virology and Cell Biology, Friedrich-Loeffler-Institut, Südufer 10, 17493 Greifswald, Insel Riems Germany; 4 Junior Research Group Arenavirus Biology, Friedrich-Loeffler-Institut, Südufer 10, 17493 Greifswald, Insel Riems Germany; 5 Present address: Department of Discovery Oncology, Genentech, 1 DNA Way, South San Francisco, CA 94080 USA

**Keywords:** Ebola virus, Carbamoyl phosphate synthetase 2 aspartate transcarbamylase and dihydroorotase, De novo pyrimidine synthesis, Dihydroorotate dehydrogenase, Host factor, Teriflunomide, siRNA screen, Non-segmented negative-sense RNA viruses, NNSV

## Abstract

**Background:**

The 2014–2016 Ebola virus (EBOV) outbreak in West Africa highlighted the need for improved therapeutic options against this virus. Approaches targeting host factors/pathways essential for the virus are advantageous because they can potentially target a wide range of viruses, including newly emerging ones and because the development of resistance is less likely than when targeting the virus directly. However, systematic approaches for screening host factors important for EBOV have been hampered by the necessity to work with this virus at biosafety level 4 (BSL4).

**Methods:**

In order to identify host factors involved in the EBOV life cycle, we performed a genome-wide siRNA screen comprising 64,755 individual siRNAs against 21,566 human genes to assess their activity in EBOV genome replication and transcription. As a screening platform, we used reverse genetics-based life cycle modelling systems that recapitulate these processes without the need for a BSL4 laboratory.

**Results:**

Among others, we identified the de novo pyrimidine synthesis pathway as an essential host pathway for EBOV genome replication and transcription, and confirmed this using infectious EBOV under BSL4 conditions. An FDA-approved drug targeting this pathway showed antiviral activity against infectious EBOV, as well as other non-segmented negative-sense RNA viruses.

**Conclusions:**

This study provides a minable data set for every human gene regarding its role in EBOV genome replication and transcription, shows that an FDA-approved drug targeting one of the identified pathways is highly efficacious in vitro*,* and demonstrates the power of life cycle modelling systems for conducting genome-wide host factor screens for BSL4 viruses.

**Electronic supplementary material:**

The online version of this article (10.1186/s13073-018-0570-1) contains supplementary material, which is available to authorized users.

## Background

Ebolaviruses (i.e., viruses within the genus *Ebolavirus*) are members of the filovirus family and cause severe hemorrhagic fever in humans and non-human primates [1]. Human-pathogenic ebolaviruses (Ebola virus (EBOV, species *Zaire ebolavirus*), Sudan virus (species *Sudan ebolavirus*), Taï Forest virus (species *Taï Forest ebolavirus*), and the recently identified Bundibugyo virus (species *Bundibugyo ebolavirus*)) are all found in Central and West Africa, while the human apathogenic Reston virus (species *Reston ebolavirus*) is found in South East Asia [2]. Another apparently apathogenic filovirus, Lloviu virus, has been suggested to be present in Europe [3, 4]. Despite recent progress in the development of countermeasures, particularly the development of effective vaccine candidates [5, 6], no approved approaches currently exist for the treatment or prevention of ebolavirus disease, which can result in case fatality rates of up to 90%. The most promising current strategies consist of monoclonal antibody cocktails [7]. However, while these approaches are extremely promising in non-human primate models of Ebola virus disease [8], they are most likely only effective against a single virus species. Further, they are directed against the glycoprotein, which is prone to mutations [9], opening up the possibility for the emergence of resistant virus variants. Host-directed therapies targeting cellular factors that are essential for supporting the virus life cycle have the potential to circumvent such resistance issues. Further, related viruses are likely to be dependent on the cell for a similar set of resources, and indeed in recent years even more distantly related viruses have been found to exploit common host pathways (e.g., the endosomal transport and vesiculation machinery for budding [10]). Therefore, if host factors can be identified that are important for a wider range of viruses, there also is a high probability that such therapies would be effective against related newly emerging virus species. Also, from an economic point of view, such broad-spectrum therapies would be much more attractive to develop clinically than single virus approaches.

The synthesis of new RNA molecules, in the form of both genome replication and mRNA transcription, is a fundamental aspect of the ebolavirus life cycle. It is facilitated by four viral proteins, the nucleoprotein NP, which encapsidates the genome, the viral polymerase L, the polymerase cofactor VP35, and the transcriptional activator VP30 [11], which together with the RNA form the ribonucleoprotein complex (RNP). Minigenome systems allow researchers to model EBOV genome replication and transcription under biosafety level (BSL) 1 or 2 conditions (dependent on local regulations), whereas work with infectious ebolaviruses is restricted to BSL 4 facilities, which constitutes a significant obstacle to research and the development of countermeasures against these viruses [12]. In minigenome systems, a miniature version of the viral genome in which viral genes have been removed and replaced by a reporter gene (e.g., luciferase) is expressed in mammalian cells [13]. Coexpressed viral RNP proteins recognize the minigenome through its non-coding terminal leader- and trailer-sequences, which are retained in the minigenome, resulting in minigenome replication and transcription. This leads to production of reporter mRNAs and thus reporter activity directly reflecting these processes. While the fundamental mechanics of ebolavirus genome replication and transcription are well established [11], relatively little is known about host factors that participate in these processes. Protein phosphatases 1 and 2 (PP1, PP2) have been shown to dephosphorylate VP30 [14, 15], and Sec61α was shown to contribute to the function of VP24 in regulating genome replication and transcription [16]. Further, DNA Topoisomerase I was shown to interact with the ebolavirus polymerase, and functional studies have confirmed a role for this protein and particularly its phosphodiester bridge-cleaving and recombination activities, in ebolavirus genome replication and transcription [17]. However, the identity of other host proteins and pathways that may contribute to ebolavirus genome replication, transcription, and mRNA translation, as well as the potential mechanism(s) behind their involvement, have remained largely unknown.

In order to identify host factors that are involved in ebolavirus genome replication and transcription, we performed a genome-wide siRNA screen with 64,755 siRNAs against 21,566 human genes using an EBOV minigenome system that we had optimized for high-throughput screening. Identified hits were validated using infectious EBOV, and Carbamoyl-phosphate synthetase 2, aspartate transcarbamylase, and dihydroorotase (CAD), a trifunctional enzyme in the cellular de novo pyrimidine synthesis pathway [18], emerged as one of the top hits from this screen, suggesting that the de novo pyrimidine synthesis pathway is important for genome replication and transcription. Therefore, the FDA-approved de novo pyrimidine synthesis-inhibitor teriflunomide was evaluated for its efficacy, first against EBOV, and then against other non-segmented and segmented negative-sense RNA viruses, confirming that this pathway plays a role in the life cycle of a wide range of viruses.

## Methods

### Cells, viruses, and plasmids

VeroE6 (African green monkey kidney, Collection of Cell Lines in Veterinary Medicine CCLV-RIE 0929) and HEK293 (human embryonic kidney, CCLV-RIE 0197) cells were maintained in Dulbecco’s modified Eagle’s medium (DMEM; Life Technologies) supplemented with 10% fetal bovine serum (FBS; Life Technologies), 2 mM l-glutamine (Q, Life Technologies), and 1× Pen Strep (PS, Life Technologies). BSR-T7/5 cells (Baby Hamster Kidney cells stably expressing the T7 polymerase [19], CCLV-RIE0583) were grown in Glasgow’s minimal essential medium (Sigma) supplemented with 10% Newborn Calf Serum (Sigma-Aldrich) and PS and were treated with 1 mg/ml G-418 disulphate (AppliChem) added at every second passage. HuH-7 (human hepatocellular carcinoma, CCLV-RIE1079) cells were maintained in a 1:1 mixture of Ham’s F12 (ThermoFisher) and Iscove’s modified Dulbecco’s medium (ThermoFisher Scientific) containing 10% FBS and supplemented with Q and PS. All cells were cultured at 37 °C in the presence of 5% CO2 in a humidified incubator.

The recombinant EBOV expressing firefly luciferase (Ebola virus H.sapiens-rec/COD/1976/Mayinga-rgEBOV-luc2, GenBank accession number KF990214; here called rgEBOV-luc2) or green fluorescent protein (GFP; Ebola virus H.sapiens-rec/COD/1976/Mayinga-rgEBOV-GFP, GenBank accession number KF990213; here called rgEBOV-GFP) have been previously described [20]. All work with infectious EBOV was performed in the BSL 4 laboratory at the Rocky Mountain Laboratories (RML), Division of Intramural Research (DIR), National Institute of Allergy and Infectious Diseases (NIAID), National Institutes of Health (NIH), following approved standard operating procedures.

pCAGGS expression plasmids for the EBOV proteins, T7-polymerase, Tim1, luc2, and expression plasmids for the EBOV monocistronic (p1cis-vRNA-RLuc) and tetracistronic (p4cis-vRNA-RLuc) minigenomes have been previously described [21]. To create a monocistronic minigenome expressing nano-luciferase (p1cis-vRNA-nLuc), the Renilla luciferase open reading frame was exchanged against the nano-luciferase open reading frame from pNL1.1 (Promega).

pCAGGS expression plasmids for JUNV NP, Z, and GPC have been previously published [22]. The JUNV L ORF was amplified from viral RNA using reverse-transcription-PCR and cloned into pCAGGS for eukaryotic expression. To generate a JUNV minigenome, S-segment RNA was reverse transcribed and cloned into the T7-driven expression vector pAmp [23]. NP and GPC open reading frames were removed, and a nano-luciferase cassette was introduced into the GPC locus. Detailed cloning strategies are available upon request. All plasmid sequences were confirmed by Sanger sequencing.

### Inhibitors

A 20 mM teriflunomide (Sigma-Aldrich) stock solution was prepared in DMSO and further diluted in the appropriate cell culture medium. Diluted teriflunomide or DMSO corresponding to 1% of the supernatant volume was added to cells. A 25 mM orotic acid (Sigma-Aldrich) stock solution was prepared in 0.1 M NaOH and further diluted in 0.1 M NaOH. Diluted orotic acid or 0.1 M NaOH corresponding to 4% of the supernatant volume was added to cells. Inhibitors were added at the time of transfection/infection, and in the case of medium changes, fresh inhibitors were added at the time of the medium change. All concentrations indicated in the figures are final concentrations.

### Inhibitor testing with EBOV minigenomes and trVLP assays

Minigenome and trVLP assays were performed in 12/96-well format as previously described [21]. Briefly, HEK293 cells were transfected with 62.5/6.9 ng pCAGGS-NP, 62.5/6.9 ng pCAGGS-VP35, 37.5/4.2 ng pCAGGS-VP30, 500/55.6 ng pCAGGS-L, 125/13.9 ng pCAGGS-T7, 125/13.9 ng monocistronic minigenome, and 12.5/1.4 ng pCAGGS-luc2, using Transit LT1 (Mirus) at a ratio of 3 μl transfection reagent per microgram DNA. For negative controls, empty pCAGGS was substituted for pCAGGS-L. Twenty-four hours post transfection, the medium was exchanged against DMEM with PS/Q, but only 5% FBS. For minigenome assays, 48 h post transfection, cells were lysed in 200/100 μl 1× Lysis Juice (PJK), and the reporter activity in 40 μl lysate was measured in opaque white plates after addition to 40 μl Beetle Juice (PJK), Renilla Glo Juice (PJK), or Nano-Glo Luciferase Assay Reagent (Promega), using either an Infinite F200 PRO (Tecan—used for inhibitor experiments) or a Glomax Multi (Promega—used for optimization experiments) microplate reader. Reporter activity is either reported for both luciferases (i.e., nano-luciferase or Renilla luciferase as a measure for genome replication and transcription, and firefly luciferase as a measure for plasmid-driven gene expression), or values normalized to the firefly luciferase values are shown. For trVLP assays, 48 h post transfection of producer cells, HEK293 target cells (10,000 cells per well) were reverse transfected in 96-well format with 6.9 ng pCAGGS-NP, 6.9 ng pCAGGS-VP35, 4.2 ng pCAGGS-VP30, 55.6 ng pCAGGS-L, and 13.9 ng pCAGGS-Tim1, again using Transit LT1 (Mirus) at a ratio of 3 μl transfection reagent per microgram DNA. After 24 h, the cell culture supernatant was replaced with 50 μl DMEM with PS/Q, and 5% FBS, 50 μl supernatant of producer cells clarified by centrifugation at 800×*g* for 5 min at room temperature, and 50 μl of drug diluted in DMEM with PS/Q, and 0% FBS. After an additional 48 h, cells were harvested and reporter activity was determined as described above.

### siRNA-screening with EBOV minigenomes

siRNA screening was conducted using a human genome-wide library of Silencer Select siRNAs (Ambion) comprising 3 siRNAs per gene for 21,566 genes. Briefly, 2 pmol of siRNA (20 nM final concentration) was pre-spotted into 384 well plates, and 0.04 μL Lipofectamine RNAiMax (ThermoFisher Scientific) was added in 20 μL of serum-free medium. This mixture was incubated at ambient temperature for 30 min prior to adding 2400 HEK293 cells in 20 μL of 20% serum DMEM media. The Silencer select aneg #2 (Ambion) and an anti-EBOV-L siRNA (UUUAUAUACAGCUUCGUACtt) served as negative and positive siRNA controls, respectively, and were included in 16 replicates each on every plate. Forty-eight hours post siRNA transfection 1.656 ng pCAGGS-NP, 1.656 ng pCAGGS-VP35, 1.008 ng pCAGGS-VP30, 13.344 ng pCAGGS-L, 3.336 ng pCAGGS-T7, 3.336 ng monocistronic minigenome, and 0.336 ng pCAGGS-luc2 were combined in a volume of 1.9 μl OptiMEM (ThermoFisher Scientific) with 0.05 μl Transit LT1 (Mirus) and incubated for 15 min, before 3.6 μl DMEM with 5% FBS was added to the transfection mix, and 6 μl diluted mix was added to the cells. After an additional 48 h, cells were assayed using the Nano-Glo Dual Luciferase Reporter Assay System (Promega).

Knockdown efficacy of the anti-NXF1 and anti-CAD siRNAs used in this study was confirmed by Western Blotting (Additional file 1: Figure S6).

### Analysis of siRNA screening data

Activity for both firefly and nano-luciferases was calculated for each well as a percentage of the median activity of that luciferase in negative control siRNA-treated wells on the same plate. The log-ratio of these two percentages was then calculated. A robust *Z*-score was calculated from this log-ratio by subtracting the median and dividing by the median absolute deviation (MAD). A seed-corrected version of this *Z*-score was then calculated by subtracting the median *Z*-score of all siRNAs having the same hexamer seed sequence (bases 2–7). In cases where subtraction of the median would result in changing the sign of the *Z*-score, the seed-corrected *Z*-score was set to zero (making the assumption that the majority of activity for that siRNA could be attributed to seed-based off-target effects). RSA was performed as described by König et al. [24]. String analysis was performed using String version 10.5 [25], with a minimum required interaction score of 0.4 (medium confidence).

### JUNV trVLP assay for siRNA and inhibitor testing

BSR-T7/5 cells in six-well format with ~ 50% confluency were transfected with 1000 ng pCAGGS-JUNV-L, 250 ng pCAGGS-JUNV-NP, 250 ng pCAGGS-T7, and 250 ng of the JUNV minigenome using Transit LT1 (Mirus) at a ratio of 3 μl transfection reagent per microgram DNA. Twenty-four hours post transfection, the same cells were transfected with 125 ng pCAGGS-GPC and 125 ng pCAGGS-Z. For siRNA experiments, HEK293 cells were reverse transfected in 96-well format by incubating 2 pmol lyophilized siRNA in 50 μl OptiMEM with 0.2 μl Lipofectamine RNAiMax for 30 min and then adding 5000 cells in 50 μl DMEM with 10% FBS, PS/Q. For inhibitor experiments, HUH7 target cells were seeded into 96-well plates for ~ 50% confluency on the next day. After 24 h, both types of target cells were transfected with 13.9 ng pCAGGS-JUNV-NP and 55.6 pCAGGS-JUNV-L. After 24 h (72 h after the 1st transfection of producer cells), supernatant of producer cells was harvested, pooled, spun down at 800×*g* for 5 min at room temperature, and the supernatant of target cells was replaced with 200 μl clarified producer cell supernatant, or with 100 μl clarified producer cell supernatant and 100 μl diluted drug. After 24 h, medium was exchanged against 100 μl medium with PS/Q, and 10% FBS. After an additional 24 h, cells were harvested and reporter activity was measured as described for the EBOV trVLP assay.

### Infection experiments in the presence of siRNAs or inhibitors

For siRNA experiments, HEK293 cells were reverse transfected in 96-well format by incubating 2 pmol lyophilized siRNA in 50 μl OptiMEM with 0.2 μl Lipofectamine RNAiMax (ThermoFisher Scientific) for 30 min and then adding 5000 cells in 50 μl DMEM with 10% FBS, PS/Q. After 48 h, supernatants were removed and cells infected with 1000 TCID_50_ rgEBOV-luc2 in a volume of 100 μl DMEM with 5% FBS, PS/Q. After an additional 48 h, 100 μl ONE-Glo reagent (Promega) was added to the cells, and reporter activity was measured after 10 min using a Glomax Multi microplate reader. Four biological replicates per siRNA in two independent experiments (two biological replicates per experiment) were obtained on a total of eight 96-well plates. On each plate, there were 8 wells each for negative siRNA (aneg #2), mock-infection, and no siRNA controls, and 4 wells each for the AllStars Cell Death Control siRNA (Qiagen) and the anti-EBOV L siRNA controls. For inhibitor experiments, HEK293 (EBOV, NDV, RABV) or VeroE6 (EBOV, IAV) cells were infected with GFP-expressing viruses at an MOI of 0.1 (EBOV, RABV, IAV) or 0.05 (NDV). Supernatants were harvested 48 h post infection, and titers were determined by TCID_50_ analysis. To assess the effects on cell viability, a CellTiter-GLO assay (Promega) was performed in parallel following the manufacturer’s instructions.

### Statistics

Paired two-tailed *t* tests were performed using the GraphPad online QuickCalc (https://www.graphpad.com/quickcalcs/ttest1/). *Z*′ factors (separation band/dynamic range of the assay: [(*μ*_c+_ − 3σ_c+_) − (*μ*_c−_ − 3σ_c−_)]/(*μ*_c+_ − *μ*_c−_), with *μ* being the mean and σ being the standard deviation of the positive control c+ or the negative control c− of the assay) were calculated as previously described [26]. Non-linear regression analysis for dose response curves was performed using GraphPad Prism 7.04.

## Results

### Optimization of an EBOV minigenome system for high-throughput screening

**Fig. 1:**
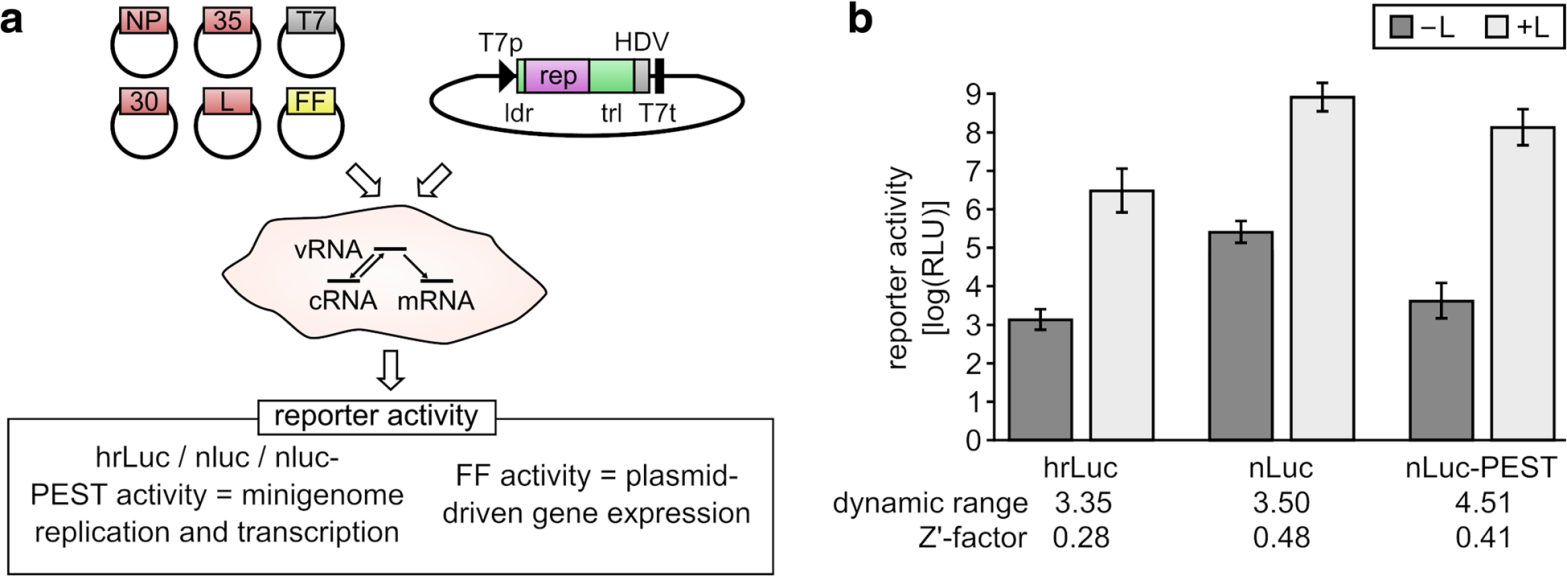
Minigenome optimization. **a** Graphical representation of the EBOV minigenome assay and structure of the minigenome. A minigenome consisting of a reporter open reading frame (rep) flanked by the EBOV non-coding leader (ldr) and trailer (trl) regions under the control of a T7 promoter (T7p) and terminator (T7t) is expressed in mammalian cells. An HDV-ribozyme (HDV) ensures an authentic 3′ end of the minigenome. T7 polymerase (T7), which initially transcribes the minigenome, and the EBOV proteins NP, VP35 (35), VP30 (30) and L, which are necessary for replication and transcription of the minigenome, are produced from RNA polymerase II-driven expression vectors. Firefly luciferase (FF) is expressed from an additional RNA polymerase II-driven expression plasmid and serves as a transfection/cell viability control, and is used to normalize minigenome reporter activity. **b** Reporter activity from minigenomes expressing various reporters. Minigenomes encoding various reporters, either Renilla luciferase (hrLuc), nano-luciferase (nLuc) or a nano-luciferase with an attached PEST-sequence (nLuc-PEST), were tested in the assay shown in panel **a**. The viral polymerase was substituted for empty vector in duplicate samples to establish the background reporter activity for each construct. Nano-luciferase and Renilla luciferase values were normalized to firefly luciferase values. Means and standard deviations of 28 biological replicates from 3 independent experiments in 96-well format are shown

The aim of this study was to identify host factors involved in genome replication and transcription of EBOV. Since work with infectious EBOV is restricted to BSL 4 laboratories, we endeavored to use a minigenome assay, which models EBOV genome replication and transcription under BSL 2 conditions (Fig. 1a). As a first step, we optimized this assay for high-throughput applications. Using the standard Renilla luciferase reporter, we achieved *Z*′ factors (i.e., the ratio of separation band (the difference between the mean of the positive control − 3 standard deviations and the mean of the negative control + 3 standard deviations) to dynamic range (i.e., the difference of the mean of the positive control and the mean of the negative control)) of 0.28 and a dynamic range of about 3.3 log_10_ in 96-well format (Fig. 1b). However, this was deemed insufficient for the planned large-scale screen, as *Z*′ factors around 0.5 or higher are generally considered to be indicative for assays well suited for high-throughput screens, whereas *Z*′ factors around or below 0.25 are considered insufficient. Therefore, we tested two additional reporters, i.e., a nano-luciferase reporter and a nano-luciferase reporter fused to a PEST sequence (to decrease intracellular half-life), due to their greatly increased brightness. While using these reporters increases minigenome-plasmid-derived background activity (as seen in the –L control) to an extent similar to the increase in positive signals, it also reduces the impact of two other sources of background signals, i.e., luminometer noise and cellular autoluminescence. Indeed, both nano-luciferases showed an improved dynamic range and an improved separation band, with *Z*′ factors of 0.48 and 0.41, respectively, which was considered sufficient to proceed with the large-scale siRNA screen in 384-well format, for which the basic nano-luciferase construct was selected.

### Genome-wide siRNA screen for factors involved in EBOV genome replication and transcription

**Fig. 2:**
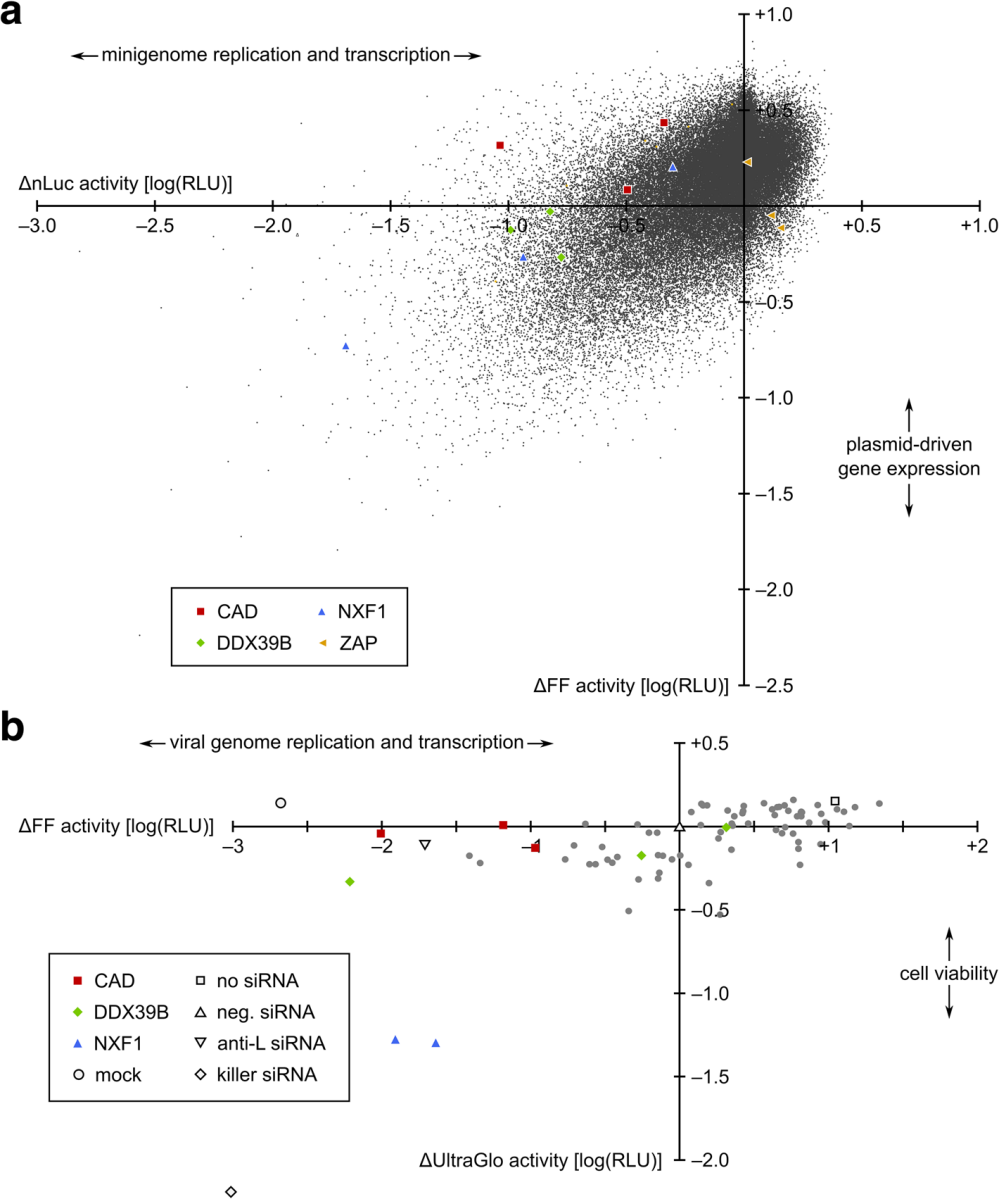
Genome-wide siRNA screen results. **a** Primary siRNA screen results using the minigenome system. A classical minigenome assay in the presence of host factor-directed siRNAs targeting each gene in the human genome in triplicate was performed in HEK293 cells. Reporter activity relative to the negative siRNA control is shown on a log scale for both nano-luciferase (nluc; reflects minigenome replication and transcription) and Firefly luciferase (FF; reflects control plasmid-driven gene expression). Results in the presence of siRNAs targeting the host factors CAD, DDX39B, and NXF1, which showed a high likelihood to be true positive hits in a follow-up analysis, and ZAP, which has been previously shown to be an antiviral factor, are highlighted as colored shapes, as indicated. **b** Secondary assay results based on infection with a luciferase-expressing EBOV. Cells were infected with a recombinant EBOV expressing Firefly luciferase (FF) in the presence of selected host factor-directed siRNAs from the primary screen shown in a). In parallel, an ATP-based cell viability assay was performed using UltraGlo luciferase activity as a readout. Mean results from 4 biological replicates from 2 independent experiments are shown relative to the negative control siRNA (neg. siRNA). Results in the presence of siRNAs targeting the host factors CAD, DDX39B, and NXF1 are highlighted as colored shapes, and controls (mock: no infection; no siRNA; anti-L: siRNA directed against EBOV L; killer siRNA: cytotoxic siRNA as a transfection control) are shown as empty shapes, as indicated

For the screen (Additional file 1: Figure S1), human HEK293 cells were reverse transfected with siRNAs from the Ambion Silencer Select siRNA library, and after 48 h, a second transfection was performed with the minigenome assay components including a control-expression plasmid encoding firefly luciferase. After 48 h, reporter activity was measured (Fig. 2a; Additional file 1: Figure S2a). Nano-luciferase activity served as readout for genome replication and transcription, whereas firefly luciferase served as a control for plasmid driven-gene expression (and thus indirectly also for cell viability), which is essential for a functional minigenome assay. In total, 64,755 siRNA targeting 21,566 human genes were individually screened, and the complete dataset has been deposited in the Genome RNAi database [27]. Information regarding the distribution of negative and positive controls, which were included in 16 biological replicates each on every plate, can be found in Additional file 1: Figure S2b. We observed a left-shift in the data which is consistent with the expectation that there are more host factors that are necessary either for general cell health, plasmid-driven gene expression, or specifically minigenome replication and transcription, than there are host factors that act as viral restriction factors.

Redundant siRNA analysis (RSA) [24] was performed on both uncorrected and seed-corrected data. RSA imputes a *p* value based on the activity of all siRNAs targeting a given gene in the context of the entire sample population, rather than using an absolute threshold. Seed correction is a method for estimating the contribution of off-target effects to the observed activity for a given siRNA [28]. Roughly 650 genes scored with an RSA *p* value of less than 0.01 in either direction (up- or downregulation of the normalized reporter signal) (Additional file 2). Intriguingly, one of the best scoring hits for a potential restriction factor was the Zinc finger antiviral protein (ZAP, also known as ZC3HAV1, seed-corrected RSA logP = − 3.4), which has previously been shown to inhibit EBOV genome replication and transcription [29]. As it was not feasible to follow up on all 650 hits from the RSA analysis, 232 of these genes were selected for further evaluation using three additional siRNAs each. Selection was based on a number of factors, such as the magnitude of activity and limited effects on control luciferase levels (Additional file 3). The vast majority of selected genes (203) scored with an RSA *p* value of < 0.005. Results of the primary and secondary screens were combined and analyzed again by RSA and common seed analysis (Additional file 4). Results from the follow-up evaluation (Additional file 5, Additional file 1: Figure S3) were converted into *Z*-scores using primary screen metrics. Genes with a total of 3 or more siRNAs (both primary and secondary testing) with a *Z*-score of at least 1.5 were considered “validated” (75 genes, *p* < 0.01). Thirty-one of these genes were selected for further testing using infectious EBOV (Fig. 2b, Additional file 6). For this purpose, we used a recombinant EBOV expressing firefly luciferase from an additional transcriptional unit (rgEBOV-luc2), which we have recently developed [20]. This virus is not only very well suited for screening assays in 96-well format under BSL 4 conditions, but allows for a very rapid and sensitive quantification of EBOV genome replication and transcription during infection [21]. To this end, HEK293 cells were reverse transfected with siRNAs, infected with rgEBOV-luc2 24 h later, and analyzed for reporter activity (reflecting genome replication and transcription) after another 48 h. In parallel, effects of the siRNAs on cell viability were measured using a commercial cell viability assay based on intracellular ATP concentration [30].

Results identified 3 host factors (CAD, NXF1, and DDX39B) where the best siRNA resulted in a reduction in reporter activity (corresponding to viral genome replication and transcription) comparable to that seen when targeting the viral polymerase L directly, i.e., by about 2 log compared to the negative control siRNA. String analysis of those factors indicated that they are associated with each other, increasing the confidence that these are, indeed, true-positive hits with biological relevance. Two of these three factors, i.e., CAD and NXF1, were also remarkable insofar as all examined siRNAs targeting these factors showed a strong reduction in reporter activity following reporter virus infection (Fig. 2b). In the case of CAD, this reduction was by 9.3-fold, 15.1-fold, and 102.3-fold, and in the case of NXF1 by 43.7-fold and 81.3-fold. Further, for DDX39B, which was the target of the siRNA showing the strongest reduction in reporter activity, two out of three siRNAs resulted in a reduction by 1.8-fold and 162.18-fold, respectively. In the presence of siRNAs targeting NXF1 we also observed a decrease in cell viability, whereas with the other siRNAs no concomitant decrease in cell viability was observed.

### Confirmation of the role of de novo pyrimidine synthesis in EBOV genome replication and transcription using teriflunomide

**Fig. 3:**
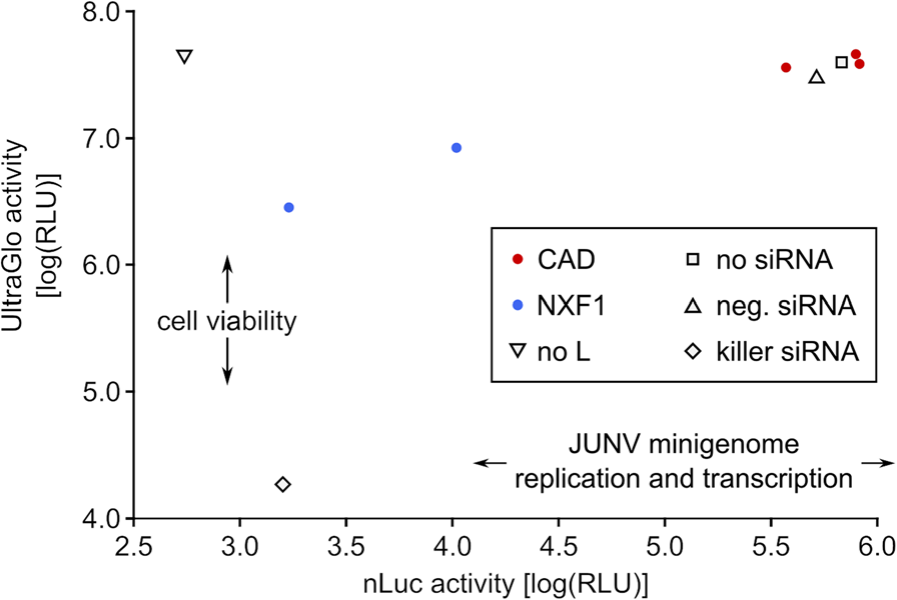
Effect of CAD knockdown on Junín virus minigenome replication and transcription. To exclude an unspecific role of CAD on T7-driven minigenome systems in general, Junín virus (JUNV) minigenomes encoding nano-luciferase (nLuc) were replicated and transcribed in cells pretransfected with siRNAs against CAD, NXF1, or control siRNAs (no siRNA; neg. siRNA: negative control siRNA; killer siRNA: cytotoxic siRNA as transfection control). As an additional control the JUNV polymerase was omitted from the assay (no L). In parallel, an ATP-based cell viability assay was performed using UltraGlo luciferase activity as a readout. Means from 3 different experiments are shown

Based on these results, we focused on CAD, and first assessed whether this protein is required only for EBOV, or also for other genetically distinct classes of RNA viruses, such as the New world arenavirus Junín virus (JUNV), which is a negative-sense RNA virus with an ambisense coding strategy that replicates in the cytoplasm in discrete viral inclusion bodies similar to EBOV [31, 32]. To this end, we tested the influence of either CAD or NXF1 siRNAs on replication and transcription of a JUNV minigenome (Fig. 3). While we saw a strong influence of NXF1 on JUNV minigenome replication and transcription, this was again accompanied by a strong reduction in plasmid-driven gene expression, and thus potentially not due to a specific role of this gene in viral genome replication or transcription. No influence on minigenome replication and transcription could be seen in the case of CAD knockdown. This indicates that the effect we see on EBOV genome replication and transcription after knockdown of CAD is due to a specific function of this host factor in EBOV genome replication and transcription, rather than a generalized effect on cell health, overall (plasmid-driven or cellular) gene expression, or a minigenome-specific process such as T7-driven initial transcription of minigenomes, which is common to both the EBOV and JUNV minigenome systems.

**Fig. 4:**
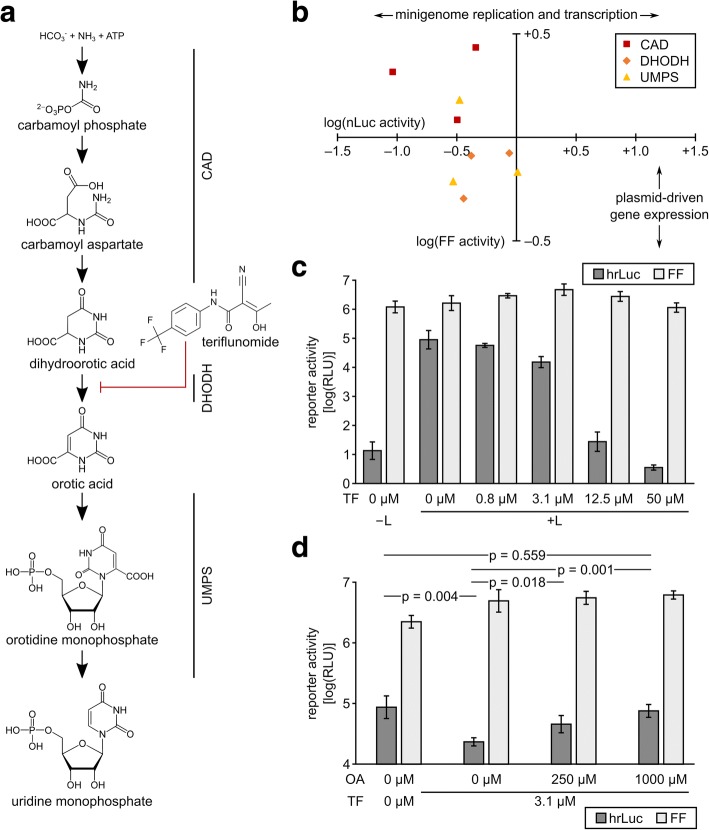
Effects of teriflunomide on minigenome replication and transcription. **a** De novo pyrimidine synthesis pathway. The production of uridine monophosphate from hydrogencarbonate, ammonia, and ATP by carbamoyl-phosphate synthetase 2, aspartate transcarbamylase, and dihydroorotase (CAD), dihydroorotate dehydrogenase (DHODH), and uridine monophosphate synthetase (UMPS) is shown, and the reaction blocked by teriflunomide is indicated. **b** Influence of knockdown of enzymes in the de novo pyrimidine synthesis pathway on minigenome activity in the primary siRNA screen. A subset of the data from Fig. 2a), comprising data for siRNAs against CAD, DHODH, and UMPS, is shown. **c** Effect of teriflunomide on ebolavirus minigenome replication and transcription. An ebolavirus minigenome assay was performed in the presence of various concentrations of teriflunomide, as indicated, using Renilla luciferase (hrLuc) as a readout for minigenome replication and transcription, and firefly (FF) luciferase expression as a readout for plasmid-driven gene expression. As a negative control, the viral polymerase was omitted (−L). Means and standard deviations from 3 independent experiments are shown. **d** Substrate rescue experiment with orotic acid. Minigenome assays were performed as indicated in panel **c** in the presence of 3.13 μM teriflunomide (TF), with increasing amounts of orotic acid (OA) added, as indicated. Means and standard deviations from 5 independent experiments are shown. *p* values from paired two-tailed t-tests are indicated

Since CAD is the first of several enzymes in the de novo pyrimidine synthesis pathway (Fig. 4a), we revisited the data from the primary screen to assess whether knockdown of other factors in the same pathway also impaired EBOV minigenome replication and transcription. CAD is followed in the metabolic pathway by dihydroorotate dehydrogenase (DHODH), which further metabolizes dihydroorotate into orotic acid, and the bifunctional uridine monophosphate synthetase (UMPS), which metabolizes orotic acid into uridine monophosphate. Indeed, for knockdown of DHODH and UMPS, we found a reduction of minigenome-encoded reporter activity in the primary screen data, albeit only for two out of three siRNAs for each gene (Fig. 4b). These reductions ranged from 2.4- to 3.4-fold. By comparison, for CAD, we observed reductions of 2.2- to 10.9-fold, depending on the siRNA used. We then further confirmed the role of the de novo pyrimidine synthesis pathway for EBOV genome replication and transcription by using teriflunomide, a known inhibitor of DHODH, the second enzyme in this pathway [33]. We observed a strong, dose-dependent effect of teriflunomide on minigenome replication and transcription, further validating a role of de novo pyrimidine synthesis in these processes (Fig. 4c). This decrease was also accompanied by a trend towards reduced minigenome replication as assessed by RT-qPCR [34] (Additional file 1: Figure S4). In contrast, the formation of inclusion bodies, which are sites of genome replication [31], was not affected in the presence of teriflunomide, and we also did not observe hypermutation as a result of teriflunomide treatment (Additional file 1: Figure S4). As teriflunomide inhibits the production of orotic acid, we next performed a substrate rescue experiment (Fig. 4d), showing that by providing exogenous orotic acid in the presence of teriflunomide, we could fully restore EBOV genome replication and transcription.

### Activity of teriflunomide against EBOV transcription and replication-competent virus-like particles

**Fig. 5:**
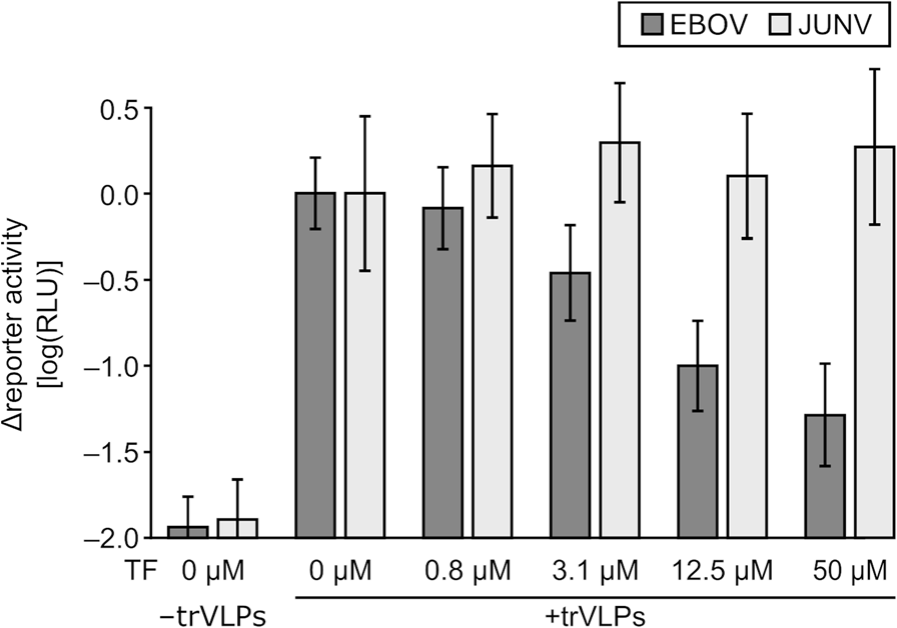
Effects of teriflunomide on infection with trVLPs. Cells were infected with either EBOV or JUNV trVLPs containing their respective minigenomes, and incubated for 48 h in the presence of the indicated amounts of teriflunomide. Afterwards, minigenome-encoded reporter activity was measured in the infected cells and is graphed on a logarithmic scale relative to untreated controls. Means and standard deviations of 12 biological replicates from 3 independent experiments are shown

While minigenome assays are ideally suited to assess genome replication and transcription in isolation, they have the disadvantage that they require a number of processes which have no equivalent in the EBOV life cycle. These include the initial transcription of viral minigenome RNA from cDNA plasmids by an exogenously provided T7-RNA polymerase, illegitimate encapsidation of these initially transcribed, naked minigenomes by NP, and dependence on plasmid-driven gene expression for expression of the RNP proteins and T7-RNA polymerase. While we did not observe an effect of teriflunomide on control plasmid-driven gene expression in our minigenome experiments, we could not rule out an off-target effect of teriflunomide on the other artificial processes. To address this issue, we utilized a transcription and replication-competent virus-like-particle (trVLP) assay, which authentically models the complete virus life cycle (with the exception of primary transcription), and which is independent of these artificial processes in target cells [12]. As a control, we used a similar assay for JUNV. As expected based on our CAD knockdown results, we observed no effect on JUNV minigenome replication and transcription in cells infected with JUNV trVLPs and treated with teriflunomide (Fig. 5). In contrast, we observed a strong dose-dependent decrease in reporter activity reflecting EBOV minigenome replication and transcription in cells infected with EBOV trVLPs in the presence of teriflunomide (Fig. 5), consistent with both the CAD knockdown data and the effects of teriflunomide in the minigenome assay.

### Activity of teriflunomide against infectious EBOV and other viruses

**Fig. 6:**
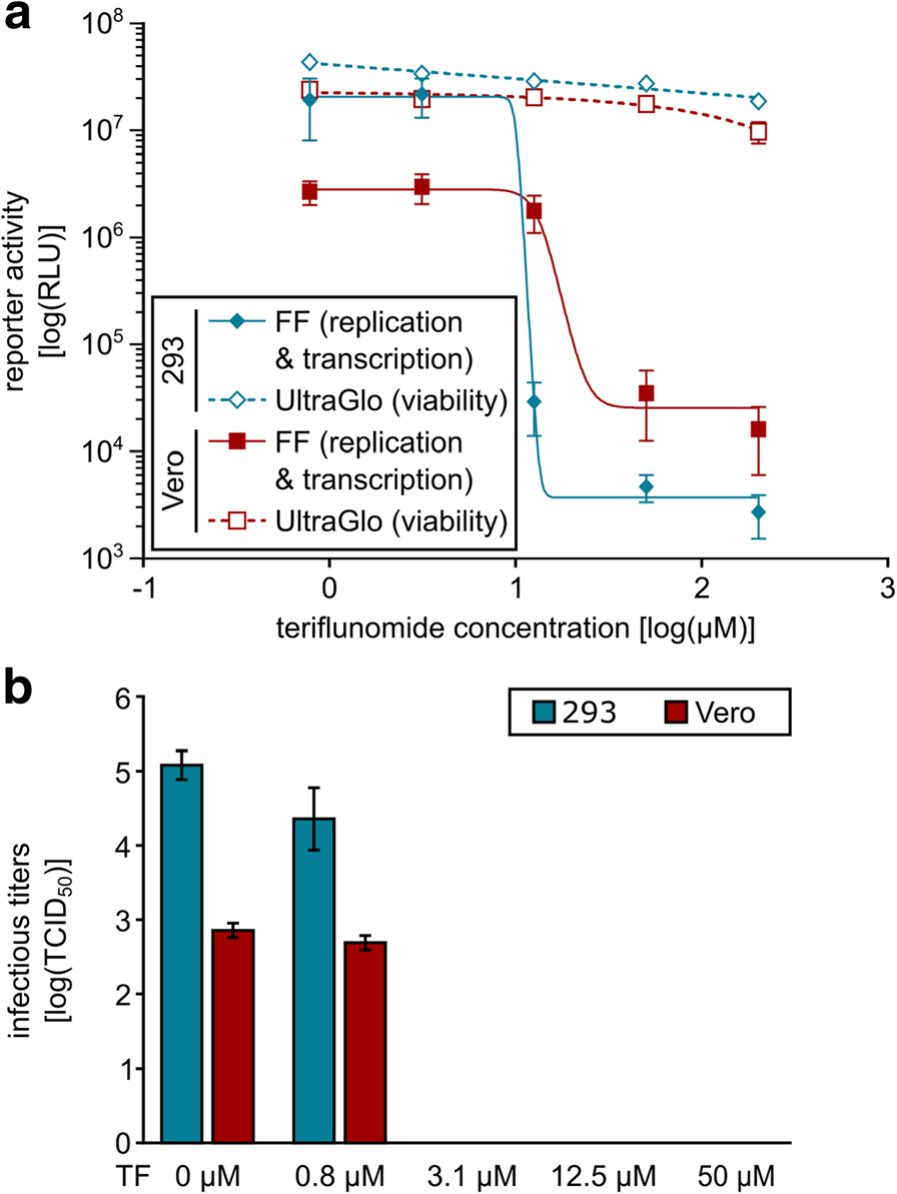
Effects of teriflunomide on infectious EBOV titres. **a** Effects of teriflunomide on a luciferase-expressing EBOV. HEK293 or VeroE6 cells were infected with a recombinant EBOV expressing firefly luciferase (FF) at an MOI of 0.05 in the presence of the indicated amounts of teriflunomide. In parallel, an ATP-based cell viability assay was performed using UltraGlo luciferase activity as a readout. Forty-eight hours post infection, reporter activity was measured. Means and standard deviations of 8 biological replicates from 2 independent experiments are shown. **b** Effect of teriflunomide on infectious titers of EBOV. Susceptible cells were infected with a GFP-expressing EBOV at an MOI of 0.05 in the presence of the indicated amounts of teriflunomide (TF). Forty-eight hours post infection, supernatants were harvested and titers determined by TCID50 analysis. Means and standard deviations from 3 independent experiments are shown

Given that teriflunomide is an FDA-approved drug, and thus should be investigated as a potential therapeutic option, we next sought to assess whether this inhibitor would also be functional against infectious EBOV. To this end, we pretreated cells with increasing doses of teriflunomide and then infected them with a luciferase expressing EBOV (rgEBOV-luc2), for which it has been shown that luciferase activity mirrors infectious titer production [35]. As expected, we observed a strong, dose-dependent decrease in reporter activity in both HEK293 and VeroE6 cells with IC_50_ concentrations of ~ 10 and 13 μM, respectively, further confirming the importance of de novo pyrimidine synthesis for the life cycle of infectious EBOV and also excluding a cell-type specific action of teriflunomide (Fig. 6a). Consistent with these results, we could also observe a decrease in infectious titers, as measured by TCID50, in the supernatant of cells infected with EBOV in the presence of teriflunomide with IC50 concentrations of ~ 3 and 4 μM, respectively (Fig. 6b).

Finally, we tested whether teriflunomide might also be active against other RNA viruses, using Newcastle disease virus (NDV, paramyxovirus), Rabies virus (RABV, rhabdovirus), and Influenza A virus (IAV, orthomyxovirus) (Additional file 1: Figure S5), and observed a strong effect of teriflunomide on NDV, which suggests that the de novo pyrimidine synthesis pathway is required for multiple Mononegaviruses.

## Discussion

One of the major challenges in studying EBOV is the fact that work with infectious virus can only be done under BSL 4 conditions, restricting this work to a few facilities worldwide. As a consequence, high-throughput applications, which are often demanding in terms of the necessary infrastructure as well as expertise, have in the past rarely been used to study EBOV. One of the reasons for this is that there has been little overlap between laboratories capable of working under BSL 4 conditions, and groups with the necessary specialization for high-throughput screening techniques [12], although for the purposes of antiviral screening reporter-expressing viruses have also been developed that are amenable to high-throughput screening under BSL 4 conditions [20, 35]. Life-cycle modelling systems alleviate this problem, as they provide the possibility to study aspects of the virus life cycle or the life cycle in its entirety under BSL 1 or 2 conditions (depending on local regulation), also in combination with high-throughput applications [35–38]. Here, we have successfully used a minigenome system in combination with a genome-wide siRNA screen, combining the expertise of a facility dedicated to performing such screens and that of a laboratory dedicated to studying BSL 4 agents.

While in this study, we have focused on only a few of the most promising hits from our primary screen as part of our secondary assay, and specifically on host factors required for genome replication and transcription, further in-depth analysis of the data might reveal additional pro- and antiviral factors playing a role in these processes. To facilitate this, the raw data from our primary screen have been posted to the GenomeRNAi database [27], making them available for data mining. Also, integrating the results from this screen with genome-wide siRNA screens for other viruses might help to reveal shared host factors, which might then be attractive as drug targets. Finally, these data might be of interest to researchers studying specific pathways or host factors, as they easily allow access to functional information about the effect of knockdown of these factors on genome replication and transcription for every human gene. For example, once we had identified the de novo pyrimidine synthesis pathway as being important for EBOV genome replication and transcription, based on the results observed after knockdown of CAD, we were able to go back to the primary screen data and find similar (albeit somewhat less pronounced) effects for knockdown of DHODH and UMPS, other factors in this same pathway, thereby further supporting our initial finding and the relevance of this pathway. Of course, such analyses come with the caveat that the individual siRNAs in the genome-wide screen are not validated in terms of their knockdown efficacy (although we experimentally confirmed knockdown efficacy of the siRNAs used for NXF1 and CAD (Additional file 1: Figure S6)); however, this issue is somewhat mitigated by the fact that data from 3 siRNAs per gene are available.

A number of inhibitors of the de novo pyrimidine synthesis pathway have been shown to exhibit antiviral activity against a wide variety of viruses in vitro [39–42], and in some cases also in vivo [43]. Two examples of these inhibitors are the FDA-approved drug leflunomide, which is an inhibitor of DHODH as well as of protein kinases, and its active metabolite teriflunomide. These have been shown to inhibit the replication of cytomegalovirus (CMV), BK virus, human herpes viruses, and respiratory syncytial virus (RSV), the latter of which is arguably the closest non-filovirus relative to ebolaviruses [44–48]. Further, the DHODH inhibitor A3 has been shown to inhibit negative sense RNA viruses such as orthomyxoviruses, paramyxoviruses, rhabdoviruses, and arenaviruses, positive sense RNA viruses such as togaviruses and flaviviruses, as well as DNA viruses and retroviruses [39, 42]. Also, a number of other inhibitors against this pathway show efficacy against one or more viruses in vitro [41, 49–52]. For EBOV, Uebelhoer et al. as well as Welch et al. have shown that 6-azauridine, which targets UMPS, inhibits replication and transcription in a minigenome system, as well virus replication in vitro [35, 38]. Our own results clearly support a role of de novo pyrimidine synthesis both based on inhibitor data using teriflunomide, but also on the level of genomic data, with the first enzyme in this pathway, CAD, emerging as one of the strongest hits of our secondary assay. The fact that we saw a stronger effect for knockdown of CAD than for knockdown of DHODH or UMPS might be due to the fact that CAD is considered the rate-limiting commitment step in de novo pyrimidine synthesis [53]. Together with existing data from others showing that inhibition of UMPS inhibits EBOV replication [35, 38], all three enzyme complexes in the de novo pyrimidine synthesis pathway have now been shown to be important for EBOV genome replication and transcription, clearly implicating this pathway as critical for the EBOV life cycle. In addition to de novo synthesis, cells can also use salvage pathways to acquire pyrimidines, which is the predominant pathway in resting cells [53]. In contrast, de novo pyrimidine synthesis is associated with dividing cells, and intriguingly, it has been shown that EBOV requires dividing cells in order to replicate [54].

With respect to the exact role of de novo pyrimidine synthesis in the viral life cycle, and the mechanism by which inhibitors of this pathway exert their antiviral activity, several mechanisms have been proposed for other viruses: (1) De novo pyrimidine synthesis is required for B and T cell proliferation [55], and thus, its inhibition depletes the target cell pools for viruses replicating in these cells, such as Epstein-Barr virus [56]. Since we did not use these cells in our assays, we exclude this mechanism as an explanation for the observed results in our system. Further, while in vivo such a mechanism could potentially contribute, EBOV is not believed to infect either B or T cells, and thus, this remains an unlikely factor. (2) De novo pyrimidine synthesis has also been shown to be important for the synthesis of UDP-sugars, which are building blocks for the glycosylation of viral proteins. For example, in the case of CMV it has been shown that inhibition of de novo pyrimidine synthesis impacts the production of infectious virus particles by interfering with proper glycosylation of CMV glycoproteins [57]. Since we observed a very strong effect of teriflunomide in the absence of the only EBOV glycoprotein GP_1,2_, this also appears not to be the major contributing mechanism, although in the trVLP system and infectious context we cannot exclude that this still plays some role. (3) Inhibition of DHODH has been reported to revert mRNA export-blocks induced either by Influenza virus NS1 or Vesicular Stomatitis Virus M, and it has been suggested that this is mediated by an increase in protein levels of the mRNA export factor NXF1 [58]. This might explain the fact that we see an increase in firefly luciferase activity at low concentrations of teriflunomide, since the firefly luciferase mRNA is transcribed by the nuclear RNA-polymerase II (PolII), and thus, the mRNA export rate should influence the expression level of firefly luciferase. However, an inhibition of mRNA export has not been described as a feature of EBOV infection, and VP40, which we have previously shown to affect expression of PolII-transcribed genes [59], was absent in the minigenome assay, speaking against a significant role of this mechanism. (4) Inhibition of de novo pyrimidine synthesis has also been shown to amplify cellular innate immune responses to viral RNA and to lead to higher expression of antiviral genes, including RIG-I and ISG56 [51, 60]. Surprisingly, depending on the exact inhibitor used, as well as possibly the virus antagonized, this mechanism seems to be either dependent on IFN production, or use a non-canonical, IFN-independent pathway for ISG-expression [41, 51, 61]. For example, the DHODH inhibitor FA-613, which exerts its antiviral effect against Influenza virus through stimulating innate immunity, is non-functional in IFN-deficient Vero cells [41], and also in our experiments, we did not observe an effect of teriflunomide against Influenza virus in Vero cells. However, teriflunomide did show strong activity against EBOV in these cells, suggesting that the antiviral effect against EBOV is due to a mechanism unrelated to IFN production. Further speaking against a role of innate immune modulation is the fact that minigenome assays were performed in the presence of overexpressed VP35, which is a strong antagonist of innate immunity not only on the level of IFN production [62] but also on the level of RNA sensing [63] and direct antagonization of antiviral effectors [64–66]. Thus, it seems unlikely, albeit not impossible, that this mechanism plays an important role in response to inhibition of the de novo pyrimidine synthesis. (5) Finally, a direct effect in depleting the intracellular pyrimidine pool is thought to be directly responsible for the decrease in genome replication and transcription [41, 42]. Given that we could completely restore minigenome replication and transcription by providing exogenous orotic acid to the cells, we consider this mechanism the most likely explanation for the antiviral effect of teriflunomide on EBOV genome replication and transcription.

Given that the DHODH inhibitor teriflunomide is an FDA- and EU-approved drug for the treatment of multiple sclerosis, with favorable long-term safety data [67], and that it proved highly efficient at inhibiting EBOV infection in vitro, it will be of great interest to test this compound in animal models for EBOV infection. Indeed, it is promising to note that other inhibitors targeting de novo pyrimidine synthesis have been showing promise in vivo [43], and in particular leflunomide, which inhibits DHODH, is being used clinically in the treatment of BK virus infection in transplant recipients [46, 48] as well as for treating CMV infections [47]. Nevertheless, whether or not teriflunomide has potential for inhibiting EBOV in vivo remains the subject of future studies.

## Conclusions

In summary, we have performed a genome-wide siRNA screen for host factors important for genome replication and transcription of EBOV, the primary data from which will allow data mining for novel pro- and antiviral factors against these processes. In our own analysis, de novo pyrimidine synthesis emerged as the top pathway important for genome replication and transcription in our screen. This was further confirmed using an inhibitor of this pathway, which is FDA- and EU-approved, and thus should be further evaluated for its efficacy against EBOV in vivo. Overall, this study highlights the power of reverse genetics-based life cycle modelling systems for understanding the biology of highly pathogenic viruses, and for the development of new approaches for countermeasures.

## Additional files

Additional file 1: Supplementary figures and methods. (PDF 884 kb)

Additional file 2: Uncorrected and seed-corrected logP values from RSA analysis, excel file format. (XLSX 1121 kb)

Additional file 3: Genes selected for follow-up, excel file format. (XLSX 14 kb)

Additional file 4: Results of common seed analysis, pdf file format. (PDF 2900 kb)

Additional file 5: Results of secondary screen, excel file format. (XLSX 50 kb)

Additional file 6: Results of secondary assay, excel file format. (XLSX 18 kb)
