# Supplementary material for: A genome-wide siRNA screen identifies a druggable host pathway essential for the Ebola virus life cycle
Source: Genome Med. 2018 Aug 7;10:58. doi: 10.1186/s13073-018-0570-1 (PMC6090742; doi:10.1186/s13073-018-0570-1)
Supplement: Supplementary file 1 — Supplementary figures and methods. (PDF 884 kb) [file 13073_2018_570_MOESM1_ESM.pdf]

Additional File 1 – Supplementary Figures and Methods

Supplementary Figures

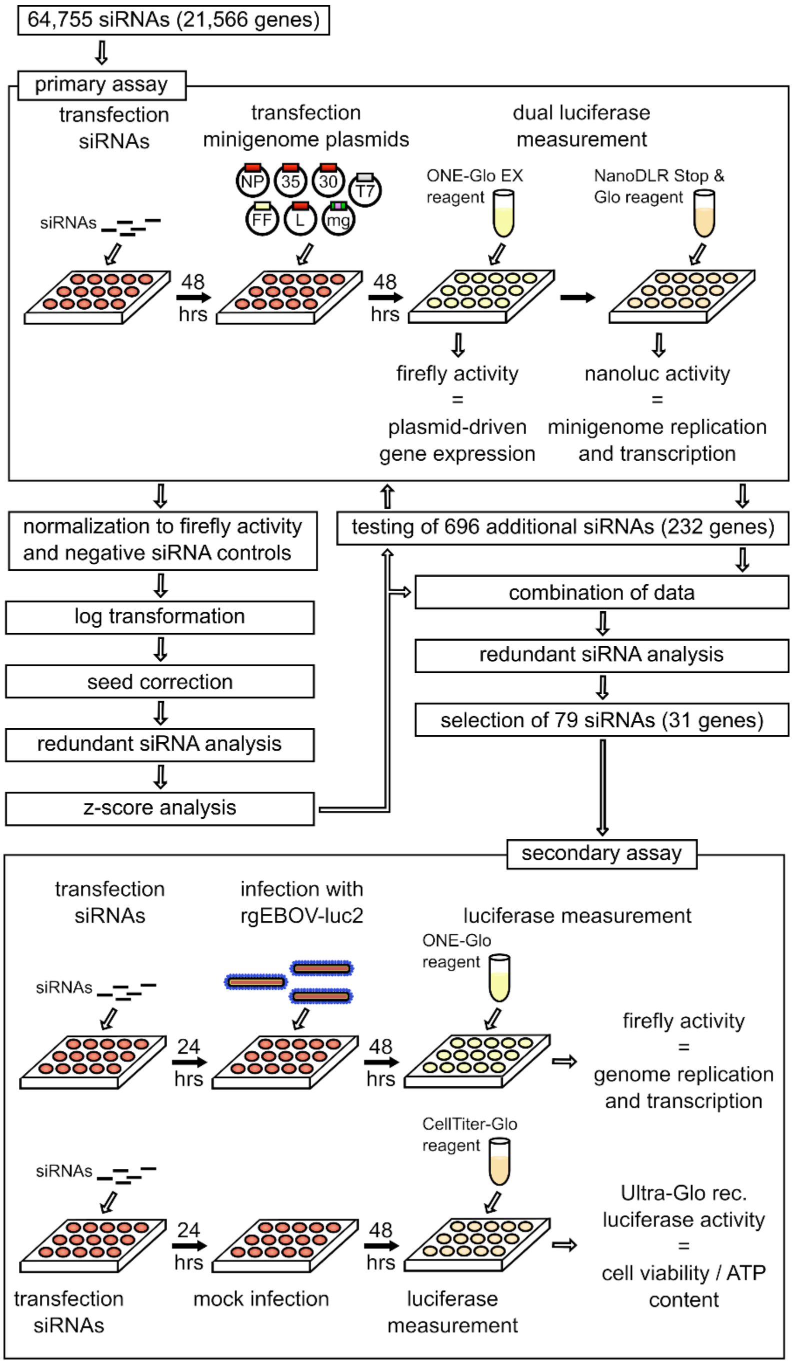

Fig. S1: Schematic representation of the assay pipeline.

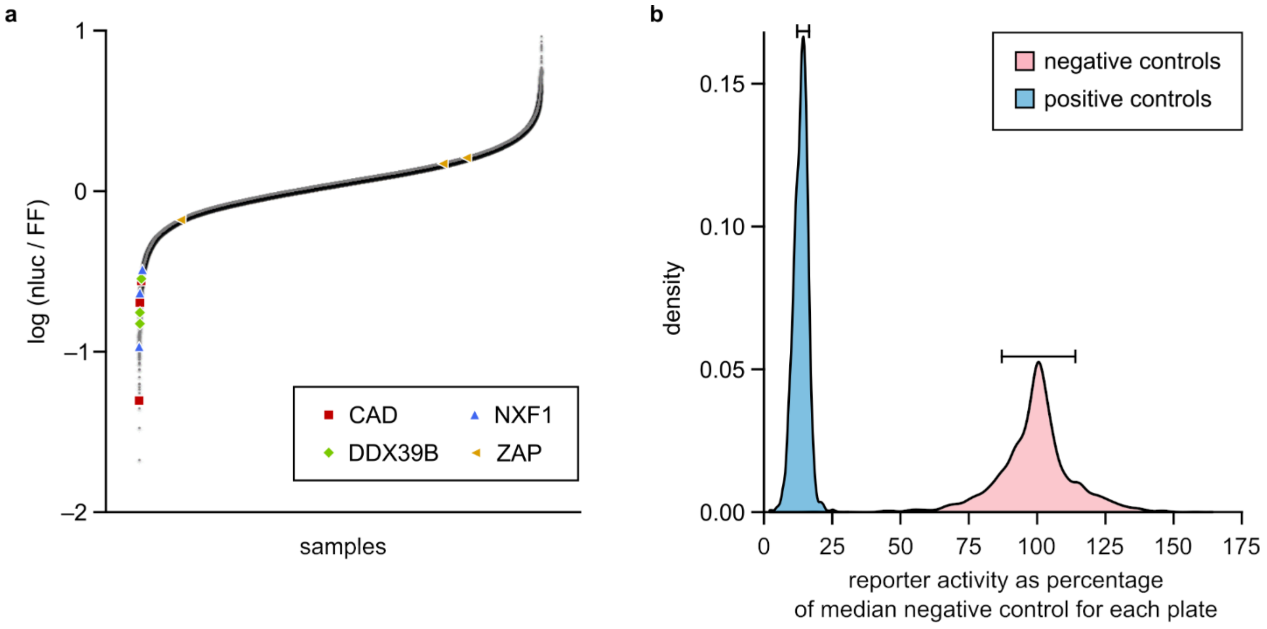

Fig. S2: Distribution of primary screen data. a) Distribution of sample data. Minigenome-derived reporter activity normalized to Firefly reporter activity reflecting plasmid-driven gene expression is shown for all samples. Samples for CAD, NXF1, DDX39B, and ZAP are highlighted. b**) Distribution of controls.** The relative distribution of negative controls (aneg #2 negative control siRNA) and positive control siRNAs (anti-EBOV L siRNA) from all replicates and all plates is shown, and the standard deviation is indicated.

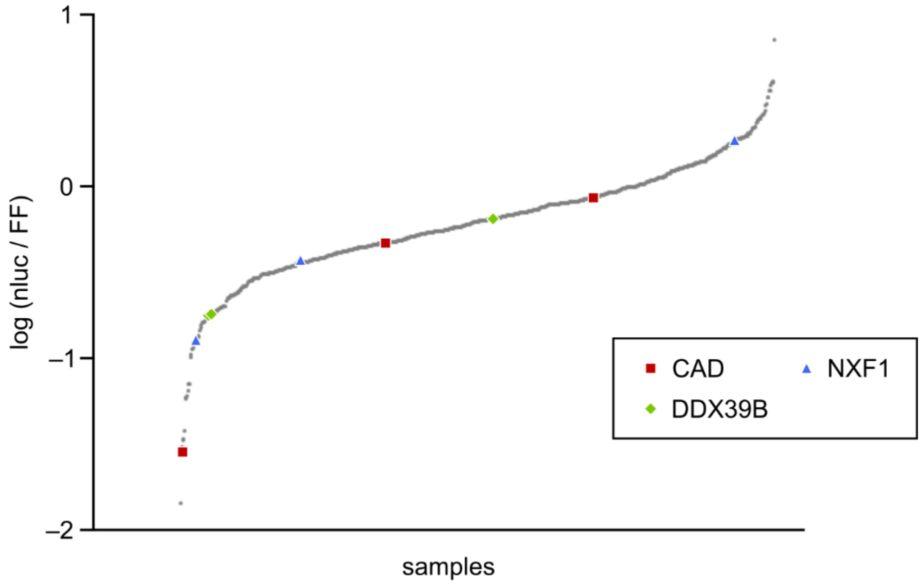

Fig. S3: Results from the secondary screen with 696 additional siRNAs. Minigenome-derived reporter activity normalized to Firefly reporter activity reflecting plasmid-driven gene expression is shown for all samples. Samples for CAD, NXF1, and DDX39B are highlighted.

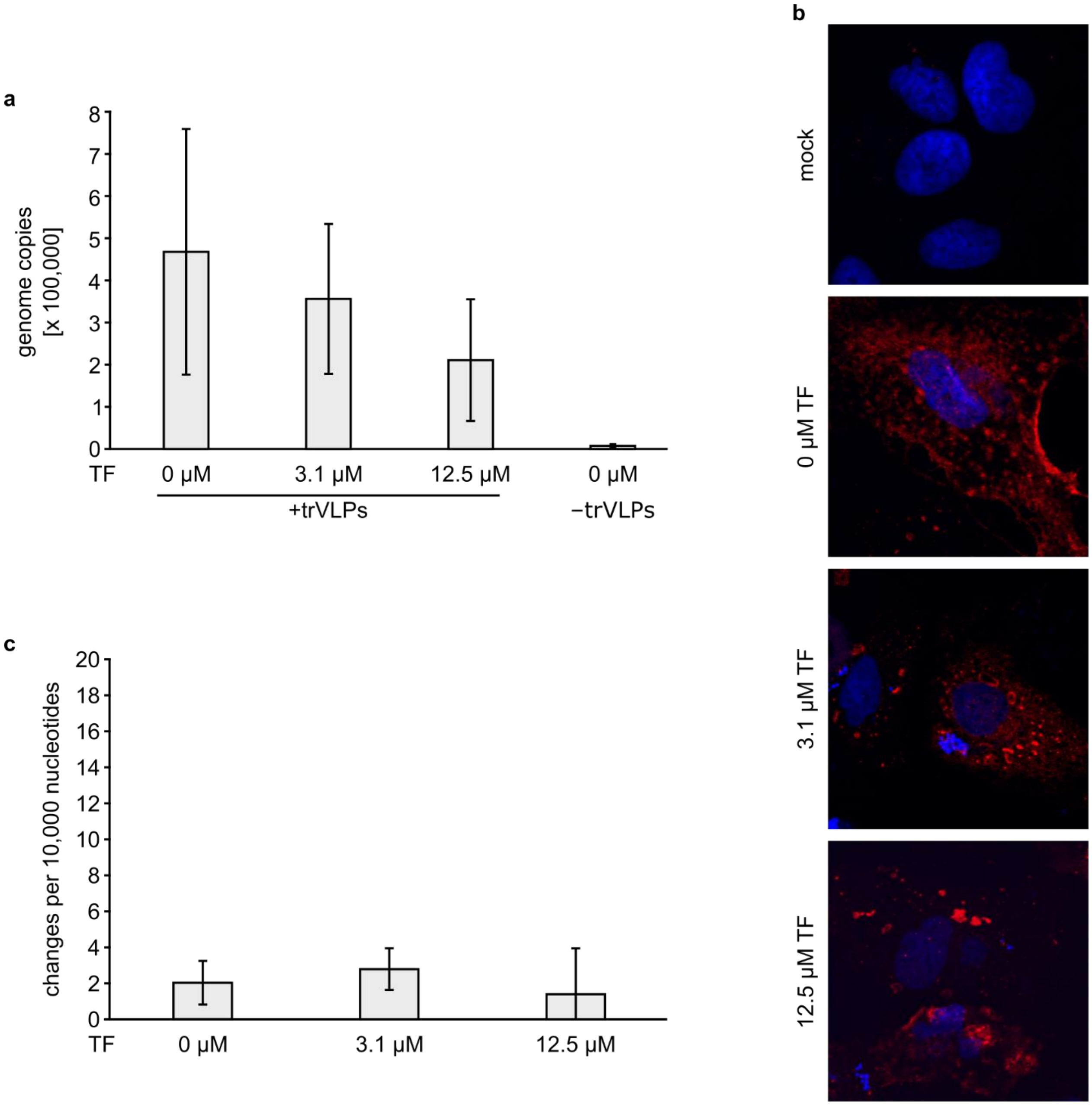

Fig. S4: Mechanistic analysis of the effect of teriflunomide on minigenome replication and transcription. a) Analysis of virus genome replication. 293T cells expressing the EBOV proteins minimally required for genome replication (i.e. NP, VP35, and L, but not VP30) were infected with trVLPs in the presence or absence of teriflunomide, as indicated. 48 hours post infection, total RNA was extracted and an RT-qPCR specifically amplifying viral genomic RNA, but not viral mRNA, was performed. Means and standard deviations from 3 biological replicates are shown. **b) Analysis of inclusion body formation.** 293T cells were

transfected with pCAGGS-NP-flag or remained untransfected (mock). Cells were treated with different amounts of teriflunomide as indicated, or comparable amounts of DMSO. 48 hours post transfection cells were fixed, permeabilized, and stained using a monoclonal anti-flag antibody (shown in red), while nuclei were stained with DAPI (shown in blue). Visualization was performed by laser scanning confocal microscopy. **c) Analysis of viral polymerase error rates.** Minigenome-encoded mRNAs produced in the presence or absence of teriflunomide, as indicated, were analyzed by deep sequencing for mutations within the reporter open reading frame on the mRNA. The average changes per 10,000 nucleotides from two biological replicates per sample are shown, together with the respective standard deviations. Historically reported error rates for RNA viruses in the absence or presence of mutagenic drugs are ~2 exchanges per 10,000 nucleotides in absence of mutagenic drugs, and ~20 exchanges per 10,000 nucleotides in presence of mutagenic drugs [1].

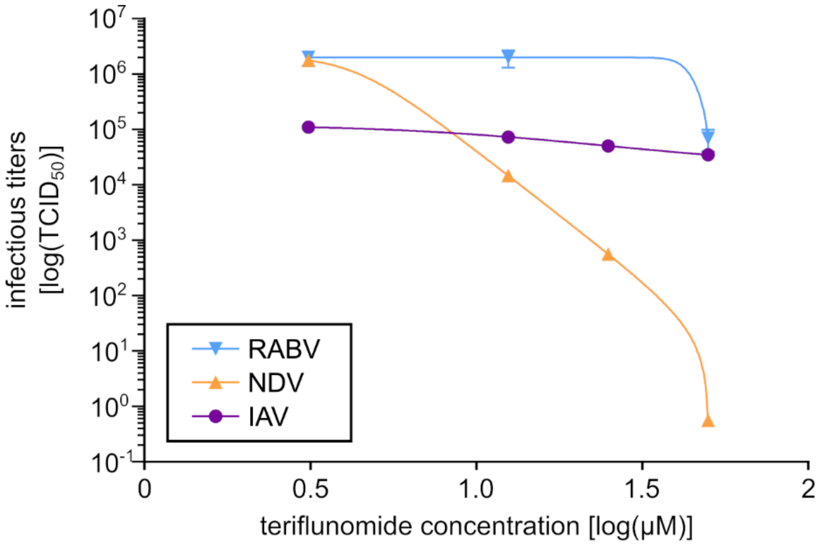

Fig. S5: Effects of teriflunomide on infectious titres of Newcastle Disease Virus (NDV), Rabies virus (RABV), and Influenza A virus (IAV). Susceptible cells were infected with GFP-expressing NDV, RABV, and Influenza virus at an MOI of 0.05 in the presence of the indicated amounts of teriflunomide. 48 hours post infection, supernatants were harvested and titers determined by TCID50 analysis. Means and standard deviations from 3 independent experiments are shown.

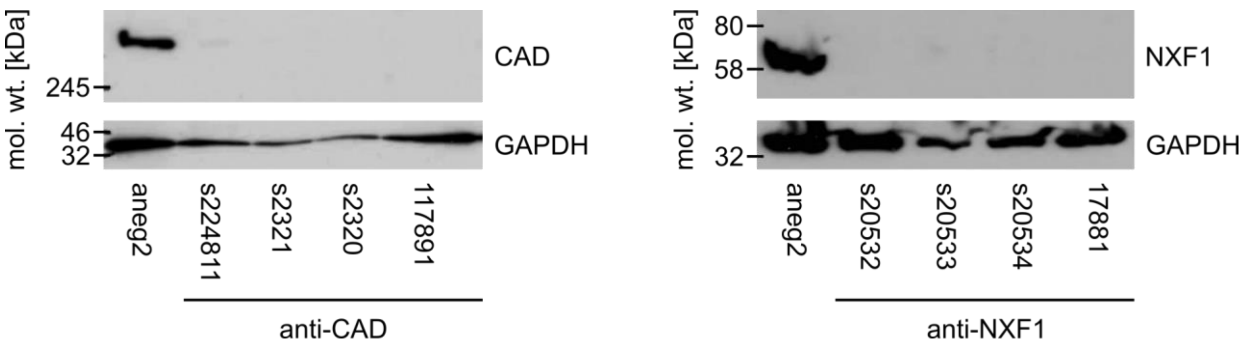

Fig. S6: Validation of siRNA knockdown. 293T cells were transfected with siRNAs against CAD or NXF1, as indicated. 48 hours post transfection endogenous protein levels were analyzed by Western Blotting using CAD- or NXF1-specific monoclonal antibodies..

Supplementary Methods

Viruses

The recombinant NDV expressing eGFP was generated as previously described [2]. Briefly, a synthetic GFP gene consisting of an NDV gene start sequence, an eGFP open reading frame, and an NDV gene end sequence was inserted into the NDV Clone 30 genome. Additionally the sequence at the F protein proteolytic cleavage site was altered from GRQGR*L to RRQKR*F. The whole genome follows the rule of six. The recombinant IAV, which is based on the isolate A/Bayern/74/2009 (H1N1), expresses eGFP from a 9th genome segment, and was generated as previously described [3]. The recombinant RABV SAD eGFP was also generated as previously described [4] from a cDNA plasmid comprising the recombinant cDNA clone SAD L16 [5] with an insertion of an N/P gene border duplication and an eGFP open reading frame at virus nucleotide position 5339.

Analysis of knockdown-efficacy by Western Blotting

293T cells in 12-well format were reverse transfected with 12 pmol siRNA per well against CAD or NXF1, as indicated, using RNAiMax following the manufacturer's instructions. 48 hours post transfection cells were lysed in SDS-PAGE buffer and subjected to Western Blotting using anti-CAD EP710Y (Abcam, 1:250), anti-NXF1 53H8 (Abcam, 1:250) or anti-GAPDH 0411 (Santa Cruz, 1:1000) antibodies, as previously described [6].

Analysis of inclusion body formation

For immunofluorescence assays, 293T cells were seeded on coverslips and transfected with 1000 ng pCAGGS-NP-flag. 48 hours post transfection cells were fixed with 4% PFA. Fixation was stopped by adding 0.1M glycine, and cells were permeabilized and blocked with 0.1% Triton X-100 containing 0.35 % BSA. Subsequently, cells were incubated with a primary mouse anti-FLAG M2 antibody (1:500; Sigma-Aldrich) followed by secondary anti-mouse IgG conjugated with Alexa Fluor 568 (1:500; ThermoFisher Scientific). Coverslips were mounted on a slide glass and nuclei were visualized using ProLong Diamond Antifade

Mountant with DAPI (Life Technologies). Observation of stained cells was performed using a laser scanning confocal microscope (SP5, Leica) with 405 and 561 nm laser lines. Subsequent data analysis was done using ImageJ v1.48 (NIH) [7].

Analysis of genome replication

Replication assays were performed as previously described [6]. Briefly, target 293T cells in 12-well format that had been pre-transfected with pCAGGS-NP (62.5 ng), pCAGGS-VP35 (62.5 ng), pCAGGS-Tim1 (125 ng), as well as pCAGGS-L (500 ng) 24 hours earlier were infected by spincoculation (10 min at 1000 x g) with 1.5 ml trVLP-containing supernatant. These trVLPs contained a tetracistronic minigenome encoding eGFP as a reporter protein. After 1 hour the inoculum was exchanged, and after 48 hours total RNA was isolated using the NucleoSpin RNA kit (Macherey-Nagel) following the manufacturer's instructions with an additional DNA digestion step using the turbo DNA-free kit (ThermoFisher Scientific). RNA samples were then subjected to real-time RT-qPCR using the AgPath-ID One Step RT-PCR kit (Applied Biosystems), with EBOV_IGR: 6FAM-CAA TAG CCA ATA CCA AAC ACC TCC TCC ACA GCT TG-BHQ1 as s probe, and the primers EBOV_IGR-fwd2 TCA CAA TCT ACC TCT TGA AAC AAG AAC and EBOV_IGR-rev2 CAT GAC TTA CTA ATG ATC TCT TAA AAT ATT AAG. To allow for absolute quantification of copy numbers, RNA standards (10^5, 10^7, 10^9 RNA copies) were prepared by *in vitro* transcription using the TranscriptAid T7 High Yield Transcription kit (ThermoFisher Scientific).

Deep sequencing

Deep sequencing was done as previously described [8]. The RNA samples used for analysis of genome replication were reverse transcribed using Superscript III reverse transcriptase with oligo-dT primers (Invitrogen) according to the manufacturer's instructions. A touchdown PCR was done using primers TTT CTG TTG GTG CTG ATA TTG CCT CGG AAT CAC AAA ATT CC and ACT TGC CTG TCG CTC TAT CTT CCT CTG CTA TTA TTC GCC TC, which contain adaptor sequences for subsequent barcoding. Barcoding and library preparation was done using the PCR Barcoding Kit I and a 1D^2 Sequencing Kit (Oxford Nanopore

Technologies) following the manufacturer's instructions. Sequencing was done overnight on a MinION Mk1 using an R9.5 flow cell (Oxford Nanopore Technologies). 86,245 reads spanning the complete eGFP ORF were analyzed in an Ubuntu 18.04 LTS environment. Reads were converted into fastq format using poretools v0.6.0 [9] and demultiplexed using flexbar v3.0.3 with parameters optimized for demultiplexing of MinION data (barcode-tail-length 300, barcode-error-rate 0.2, barcode-gap -1) [10]. Reads were aligned to a reference sequence using lastal v921 [11], and a pileup file was generated using samtools v1.7 [12]. The pileup file was evaluated using pileup2nucl.pl (see below for source code). To calculate the frequency of nucleotide changes, the number of nucleotide changes compared to the reference sequence in each samples was divided by the total number of nucleotides sequenced in the same sample. Background values obtained from deep sequencing a PCR-amplified minigenome plasmid control were subtracted from the obtained nucleotide change frequencies, in order to compensate for inaccuracy of the sequencing device and errors introduced during the PCR steps.

References

1. Crotty S, Cameron CE, Andino R: **RNA virus error catastrophe: direct molecular test by using ribavirin**. *Proc Natl Acad Sci U S A* 2001, **98**(12):6895-6900.
2. Engel-Herbert I, Werner O, Teifke JP, Mebatsion T, Mettenleiter TC, Romer-Oberdorfer A: **Characterization of a recombinant Newcastle disease virus expressing the green fluorescent protein**. *Journal of virological methods* 2003, **108**(1):19-28.
3. Gao Q, Lowen AC, Wang TT, Palese P: **A nine-segment influenza a virus carrying subtype H1 and H3 hemagglutinins**. *J Virol* 2010, **84**(16):8062-8071.
4. Finke S, Granzow H, Hurst J, Pollin R, Mettenleiter TC: **Intergenotypic replacement of lyssavirus matrix proteins demonstrates the role of lyssavirus M proteins in intracellular virus accumulation**. *J Virol* 2010, **84**(4):1816-1827.

5. Schnell MJ, Mebatsion T, Conzelmann KK: **Infectious rabies viruses from cloned cDNA**. *EMBO J* 1994, **13**(18):4195-4203.

6. Schmidt ML, Hoenen T: **Characterization of the catalytic center of the Ebola virus L polymerase**. *PLoS neglected tropical diseases* 2017, **11**(10):e0005996.

7. Schneider CA, Rasband WS, Eliceiri KW: **NIH Image to ImageJ: 25 years of image analysis**. *Nature methods* 2012, **9**(7):671-675.

8. Hoenen T, Groseth A, Rosenke K, Fischer RJ, Hoenen A, Judson SD, Martellaro C, Falzarano D, Marzi A, Squires RB *et al*: **Nanopore Sequencing as a Rapidly Deployable Ebola Outbreak Tool**. *Emerging infectious diseases* 2016, **22**(2):331-334.

9. Loman NJ, Quinlan AR: **Poretools: a toolkit for analyzing nanopore sequence data**. *Bioinformatics* 2014, **30**(23):3399-3401.

10. Roehr JT, Dieterich C, Reinert K: **Flexbar 3.0 - SIMD and multicore parallelization**. *Bioinformatics* 2017, **33**(18):2941-2942.

11. Kielbasa SM, Wan R, Sato K, Horton P, Frith MC: **Adaptive seeds tame genomic sequence comparison**. *Genome research* 2011, **21**(3):487-493.

12. Li H, Handsaker B, Wysoker A, Fennell T, Ruan J, Homer N, Marth G, Abecasis G, Durbin R, Genome Project Data Processing S: **The Sequence Alignment/Map format and SAMtools**. *Bioinformatics* 2009, **25**(16):2078-2079.

Source code for pileup2nucl.pl

```
#!/usr/bin/perl

use warnings;
use strict;

my $refbase;
my $callstring;
my $lineindex = 0;
my $position;
my %count = (
	A => 0,
	C => 0,
	G => 0,
	T => 0
);

# get next reference base and callstring
sub nextLine
{
	my $line;
	my @content;
	$line = <>;
	exit unless defined $line;  # end of file
	@content = split(" ", $line);
	$position = $content[1];
	$refbase = $content[2];
	$callstring = $content[4];
	return;
}

sub removeInDels
{
	my $croppedstring = "";
	my $offset;
	my $nextindel = index($callstring,"-");
	while ($nextindel>0)
	{
		$callstring =~ /\-(\d+)/;
		$offset = $nextindel + $1 + length($1) + 1;
		$croppedstring = $croppedstring . substr($callstring,0,$nextindel);
		$callstring = substr($callstring,$offset);
		$nextindel = index($callstring,"-");
	}
	$callstring = $croppedstring . $callstring;
	$croppedstring = "";
	$nextindel = index($callstring,"+");
```

```
	while ($nextindel>0)
	{
		$callstring =~ /\+(\d+)/;
		$offset = $nextindel + $1 + length($1) + 1;
		$croppedstring = $croppedstring . substr($callstring,0,$nextindel);
		$callstring = substr($callstring,$offset);
		$nextindel = index($callstring,"+");
	}
	$callstring = $croppedstring . $callstring;
}

while (1)
{
	&nextLine;
	&removeInDels;
	$count{'A'} = ($callstring =~ tr/Aa//);
	$count{'C'} = ($callstring =~ tr/Cc//);
	$count{'G'} = ($callstring =~ tr/Gg//);
	$count{'T'} = ($callstring =~ tr/Tt//);
	$count{$refbase} = ($callstring =~ tr/\.,//);
	print "$position\tA $count{'A'}\tC $count{'C'}\tG $count{'G'}\tT $count{'T'}\n";
}
```
