# Supplementary material for: A genome-wide siRNA screen identifies a druggable host pathway essential for the Ebola virus life cycle
Source: Genome Med. 2018 Aug 7;10:58. doi: 10.1186/s13073-018-0570-1 (PMC6090742; doi:10.1186/s13073-018-0570-1)
Supplement: Supplementary file 4 — Results of common seed analysis, pdf file format. (PDF 2900 kb) [file 13073_2018_570_MOESM4_ESM.pdf]

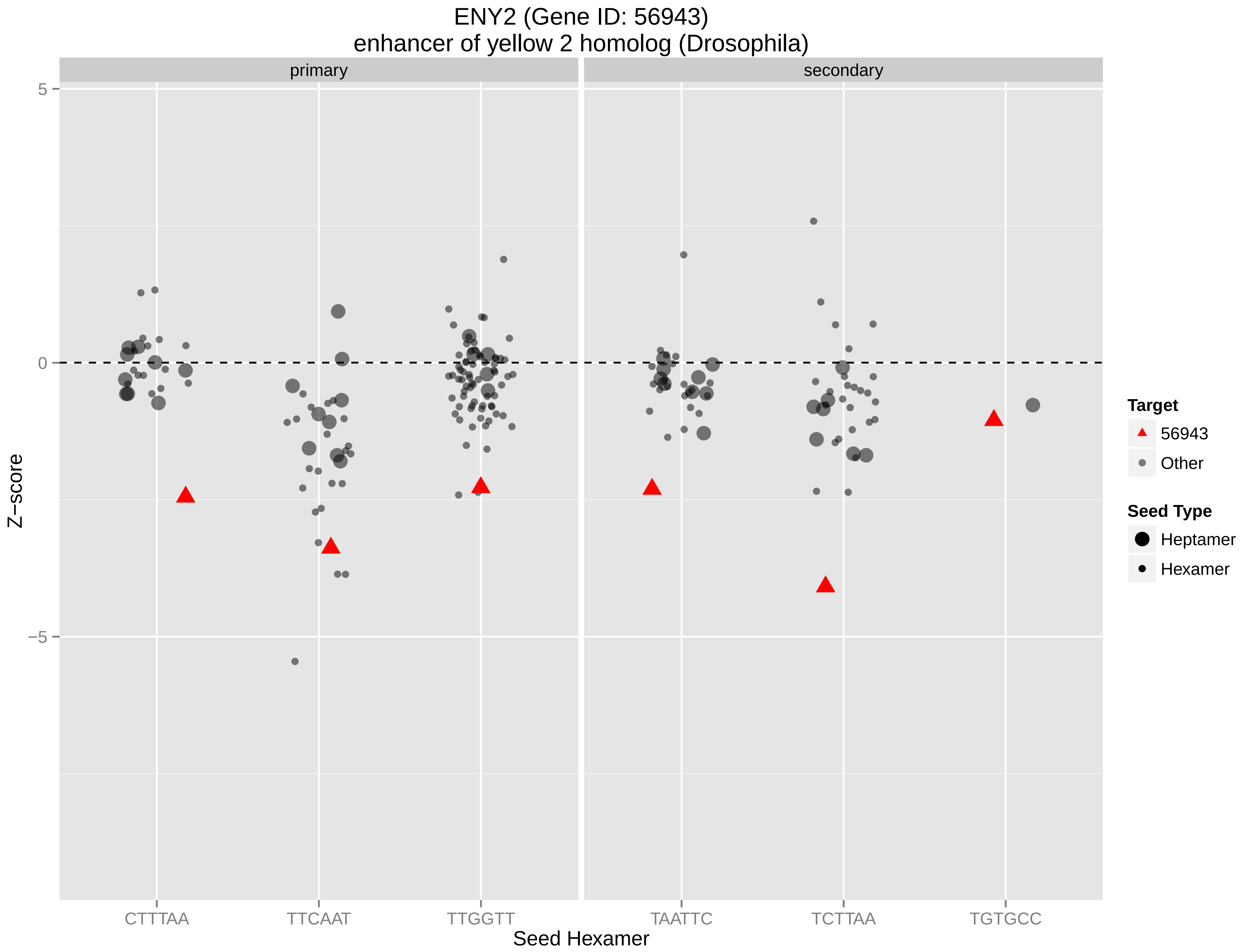

ENY2 (Gene ID: 56943)
enhancer of yellow 2 homolog (Drosophila)
primary
secondary
5
0
−5
Z−score
CTTTAA
TTCAAT
TTGGTT
TAATTC
TCTTAA
TGTGCC
Seed Hexamer
Target
56943
Other
Seed Type
Heptamer
Hexamer

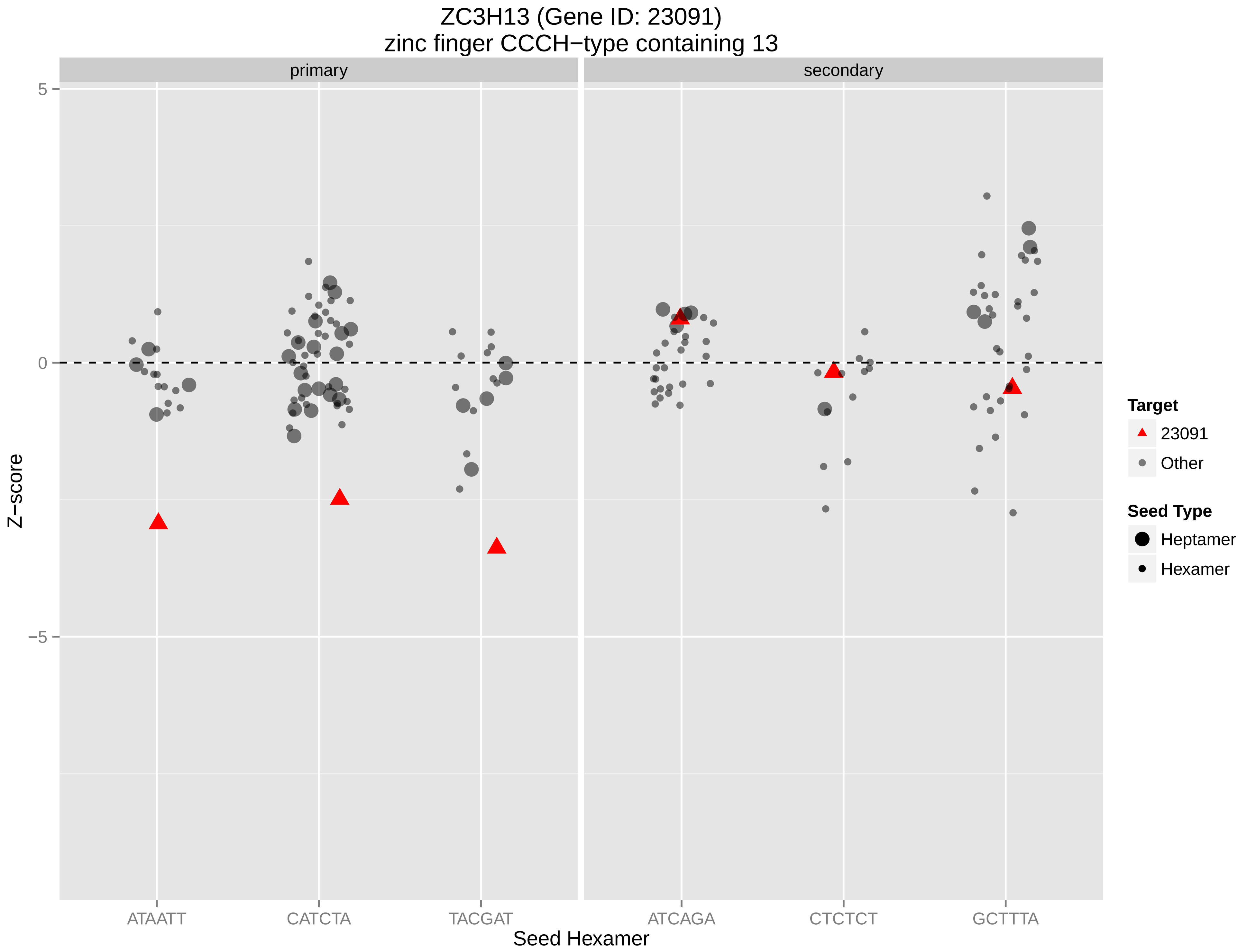
ZC3H13 (Gene ID: 23091)
zinc finger CCCH−type containing 13
primary
secondary
5
0
−5
Z−score
ATAATT
CATCTA
TACGAT
ATCAGA
CTCTCT
GCTTTA
Seed Hexamer
Target
23091
Other
Seed Type
Heptamer
Hexamer

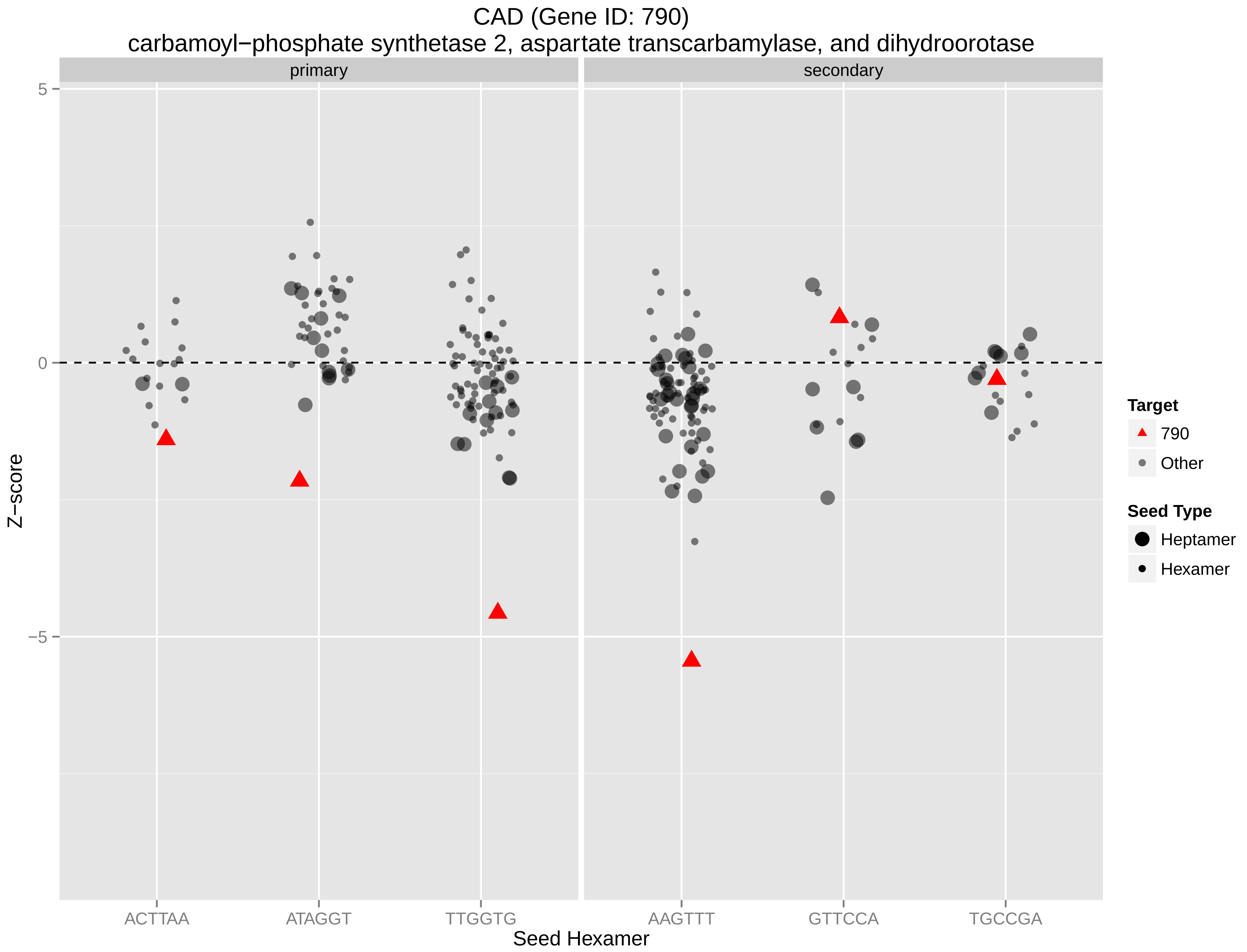
CAD (Gene ID: 790)
carbamoyl−phosphate synthetase 2, aspartate transcarbamylase, and dihydroorotase
primary
secondary
5
0
−5
Z−score
ACTTAA
ATAGGT
TTGGTG
AAGTTT
GTTCCA
TGCCGA
Seed Hexamer
Target
790
Other
Seed Type
Heptamer
Hexamer

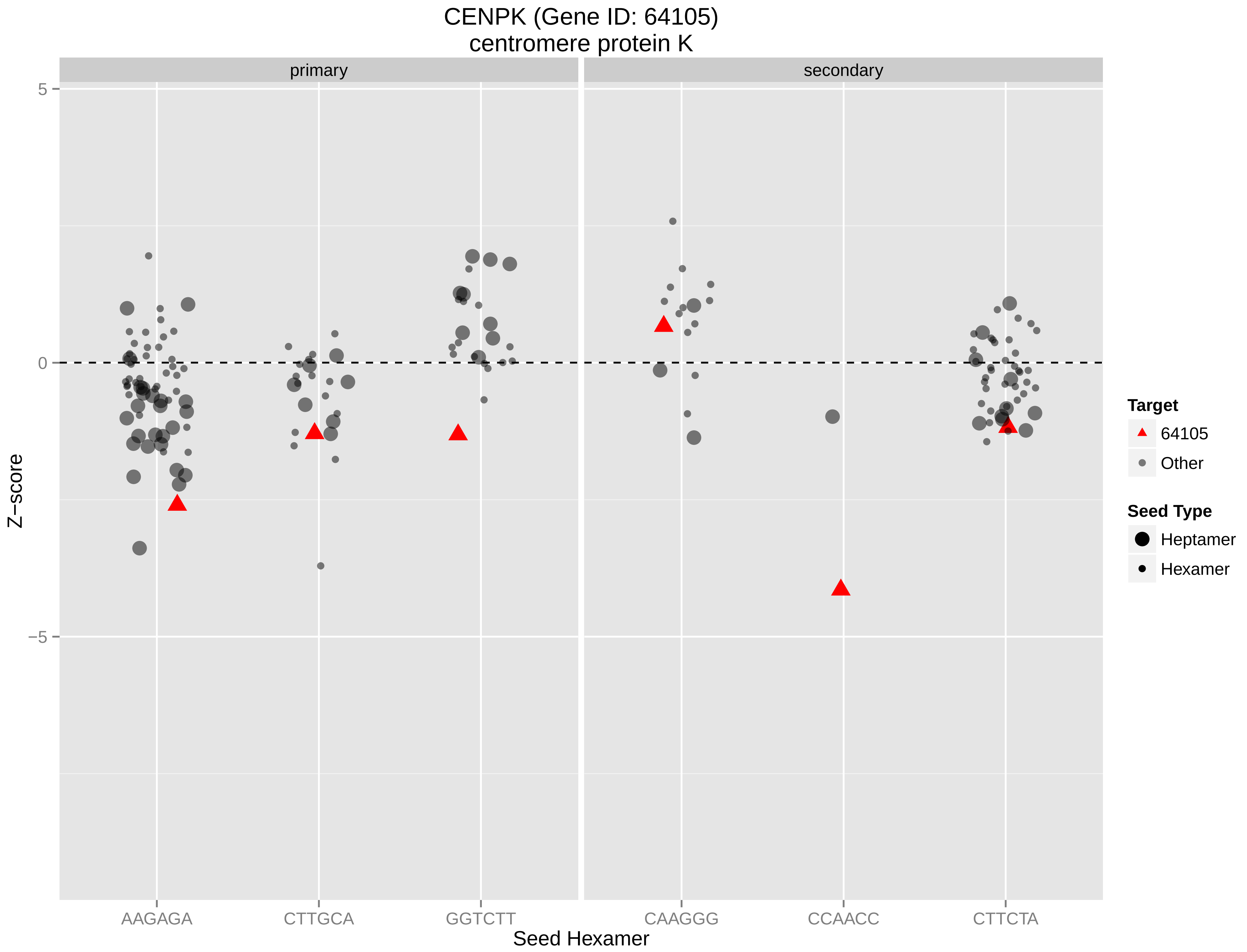
CENPK (Gene ID: 64105)
centromere protein K
primary
secondary
5
0
−5
Z−score
AAGAGA
CTTGCA
GGTCTT
CAAGGG
CCAACC
CTTCTA
Seed Hexamer
Target
64105
Other
Seed Type
Heptamer
Hexamer

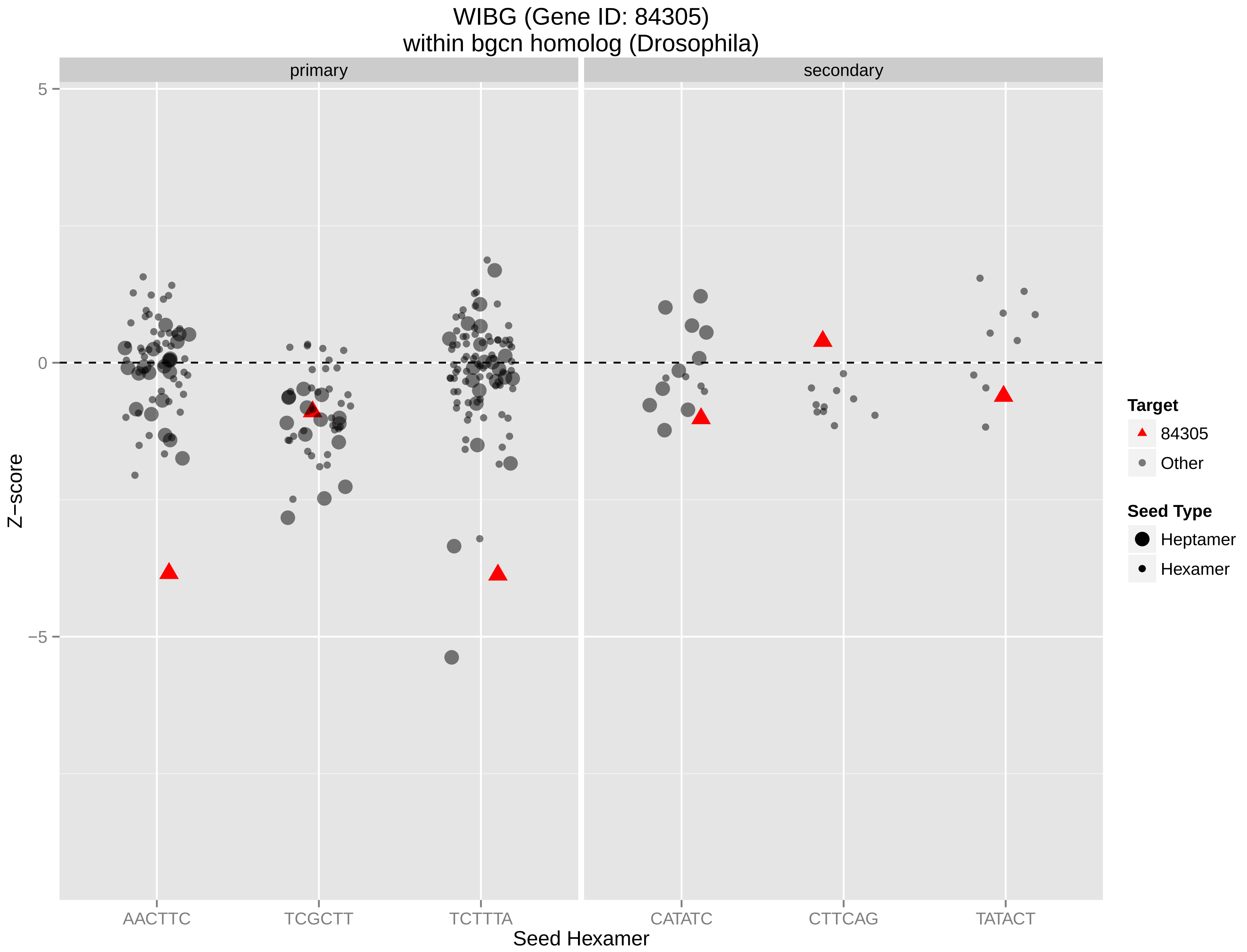
WIBG (Gene ID: 84305)
within bgcn homolog (Drosophila)
primary
secondary
5
0
−5
Z−score
AACTTC
TCGCTT
TCTTTA
CATATC
CTTCAG
TATACT
Seed Hexamer
Target
84305
Other
Seed Type
Heptamer
Hexamer

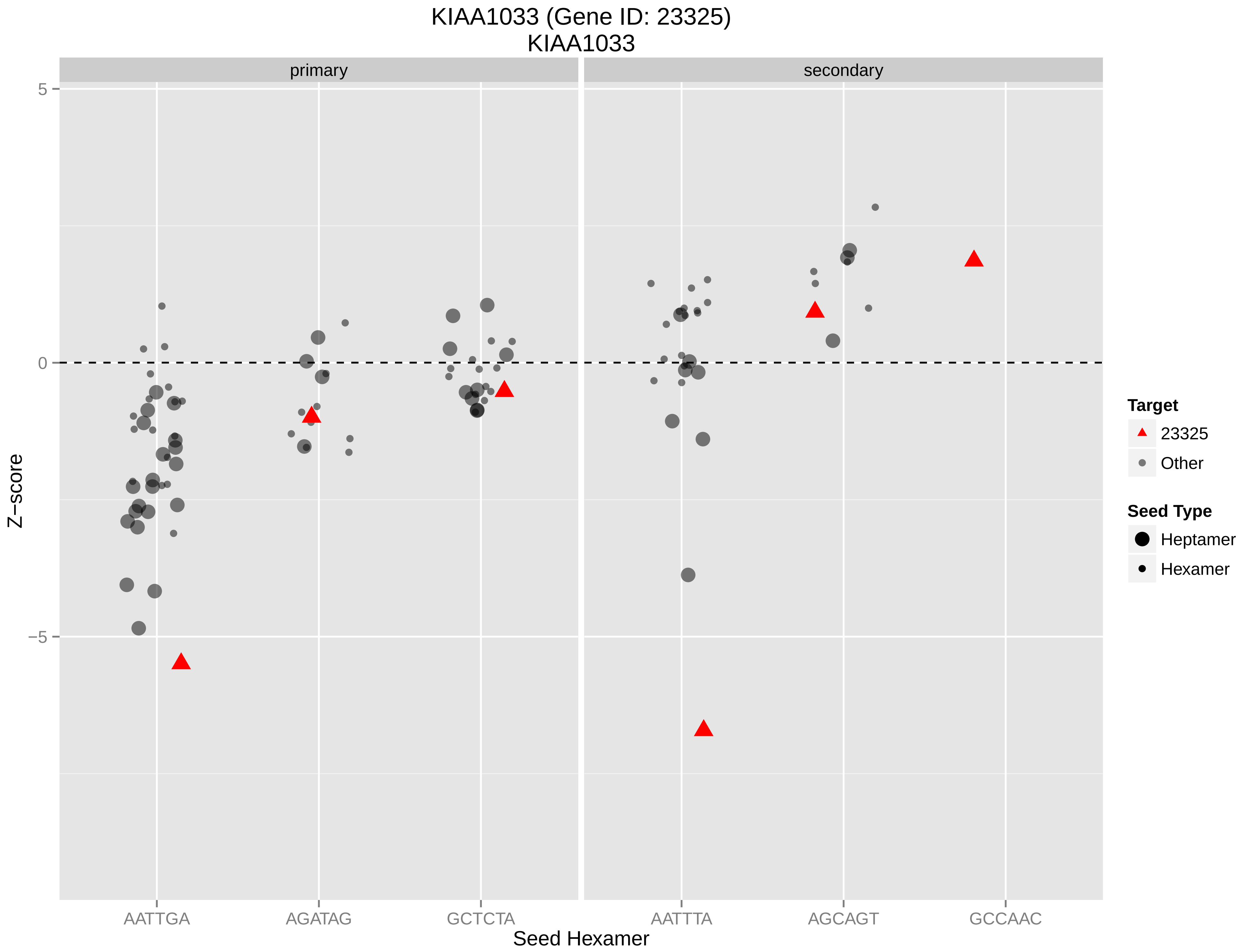

KIAA1033 (Gene ID: 23325)
KIAA1033
primary
secondary
5
0
−5
Z−score
AATTGA
AGATAG
GCTCTA
AATTTA
AGCAGT
GCCAAC
Seed Hexamer
Target
23325
Other
Seed Type
Heptamer
Hexamer

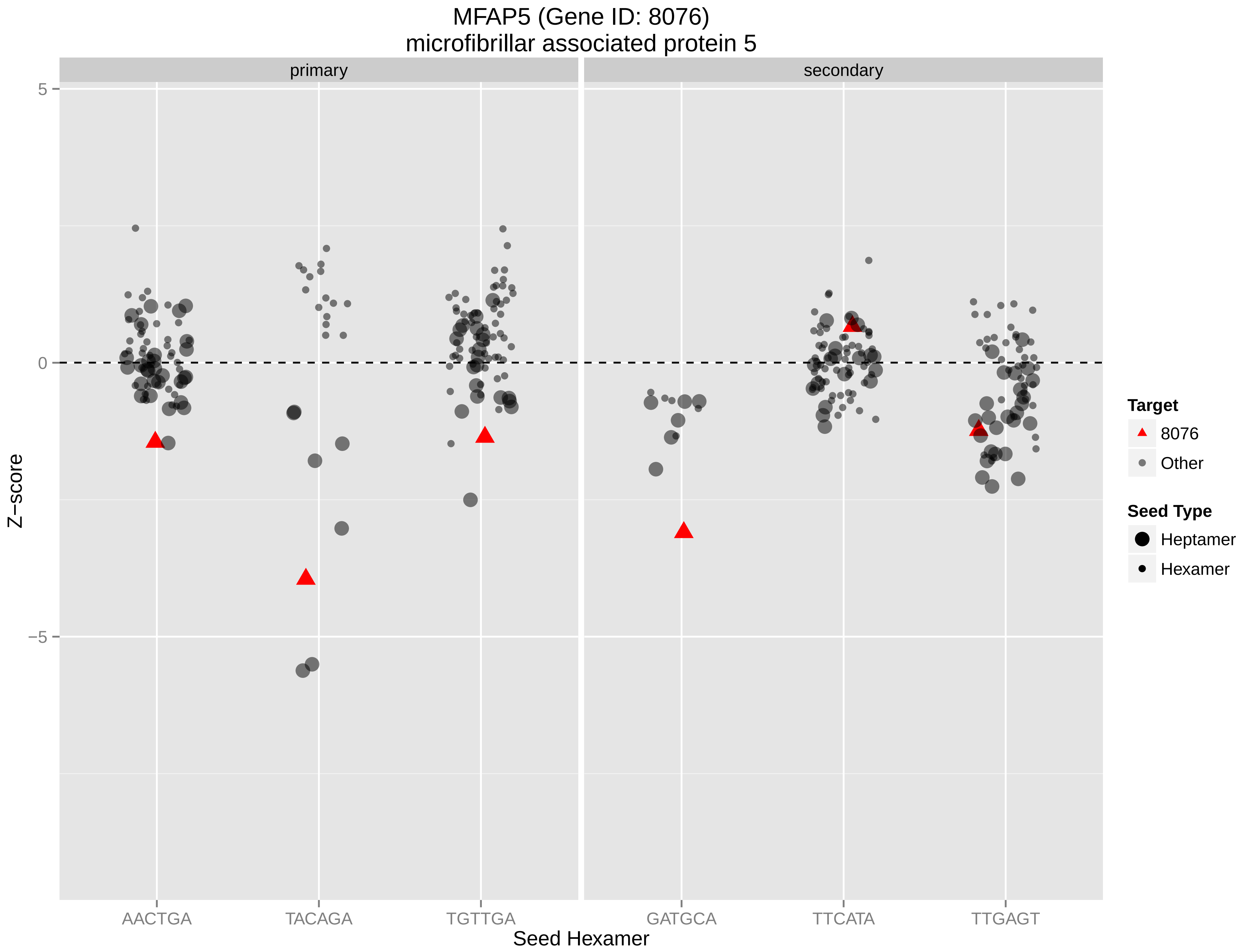
MFAP5 (Gene ID: 8076)
microfibrillar associated protein 5
primary
secondary
5
0
−5
Z−score
AACTGA
TACAGA
TGTTGA
GATGCA
TTCATA
TTGAGT
Seed Hexamer
Target
8076
Other
Seed Type
Heptamer
Hexamer

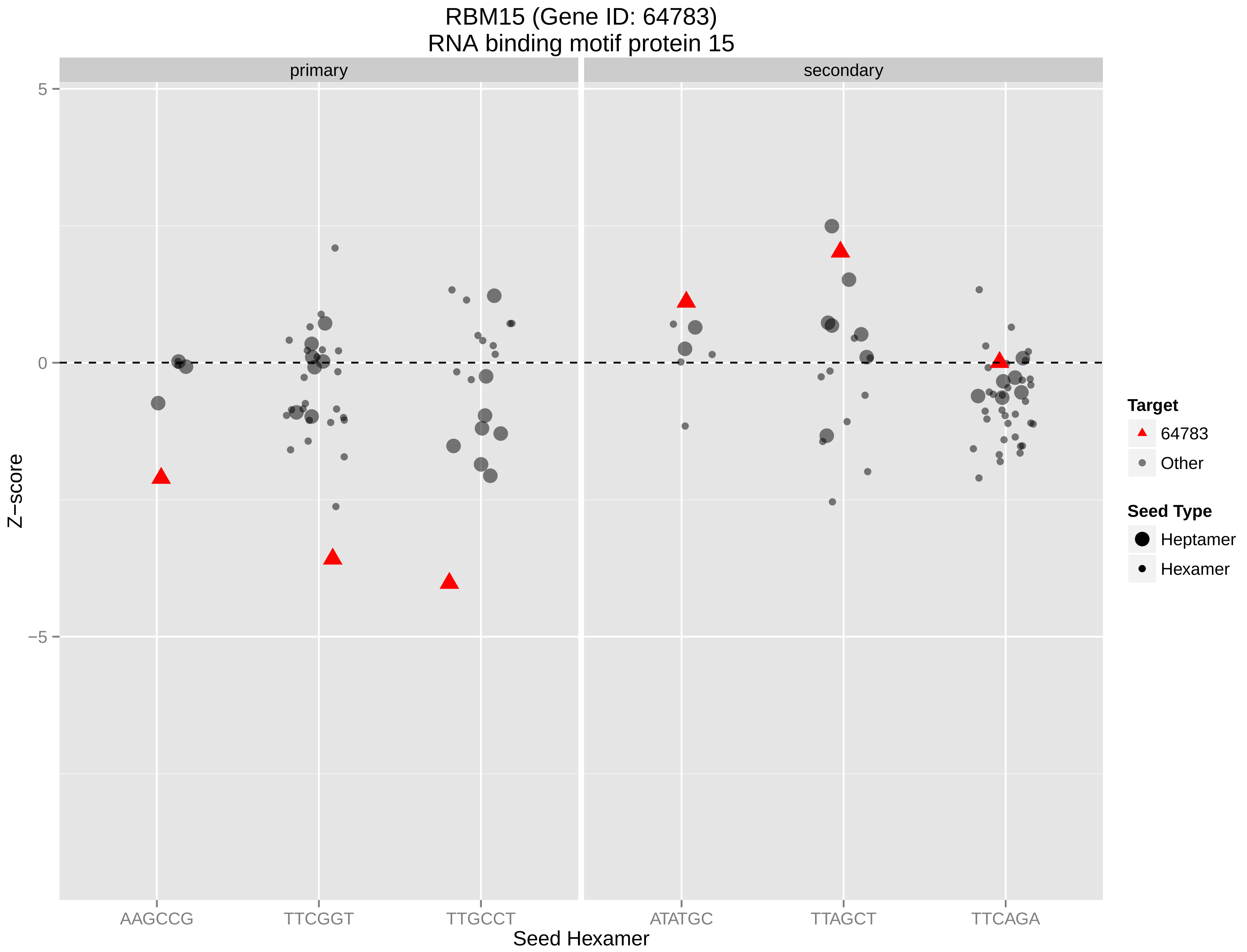
RBM15 (Gene ID: 64783)
RNA binding motif protein 15
primary
secondary
5
0
−5
Z−score
AAGCCG
TTCGGT
TTGCCT
ATATGC
TTAGCT
TTCAGA
Seed Hexamer
Target
64783
Other
Seed Type
Heptamer
Hexamer

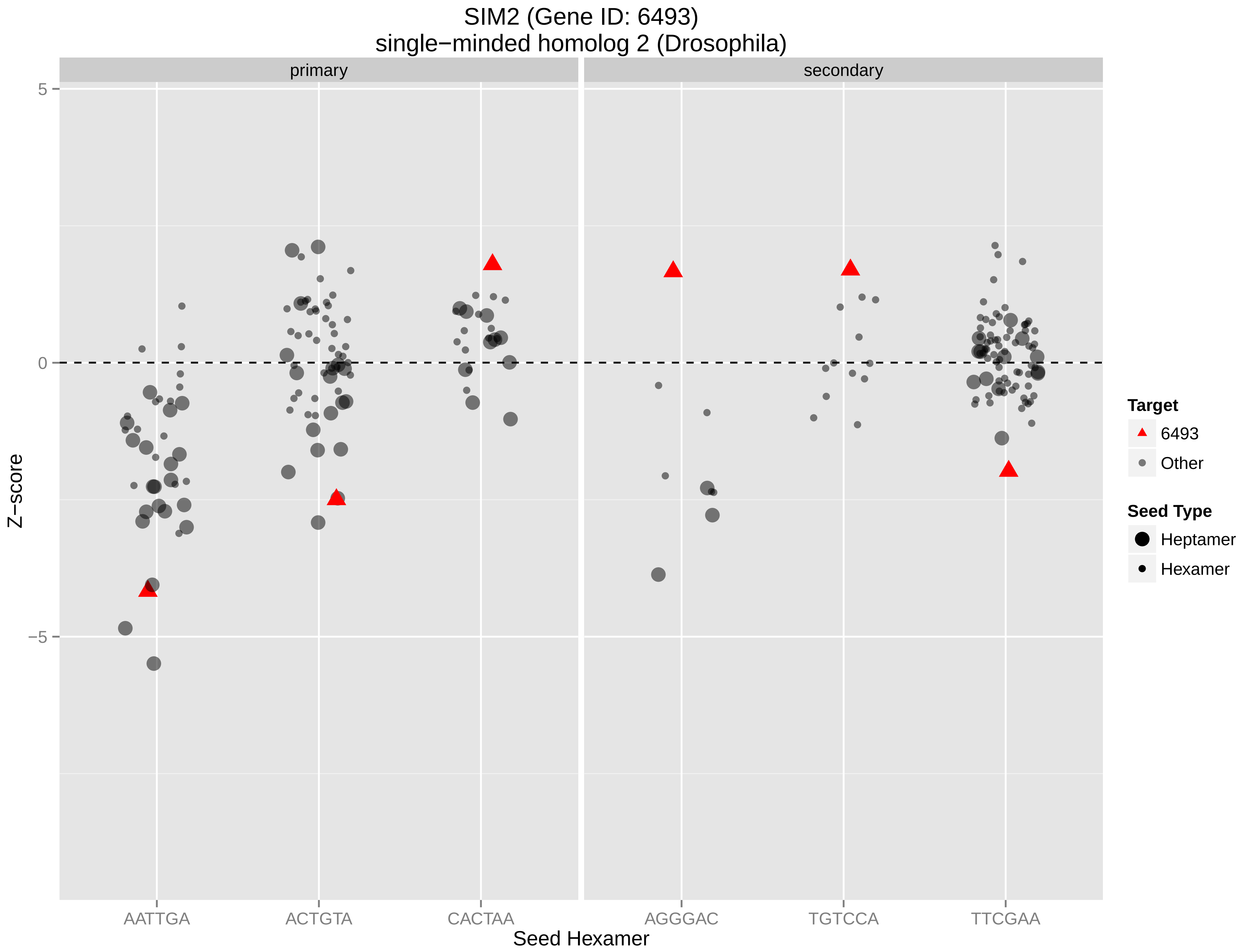
SIM2 (Gene ID: 6493)
single−minded homolog 2 (Drosophila)
primary
secondary
5
0
−5
Z−score
AATTGA
ACTGTA
CACTAA
AGGGAC
TGTCCA
TTCGAA
Seed Hexamer
Target
6493
Other
Seed Type
Heptamer
Hexamer

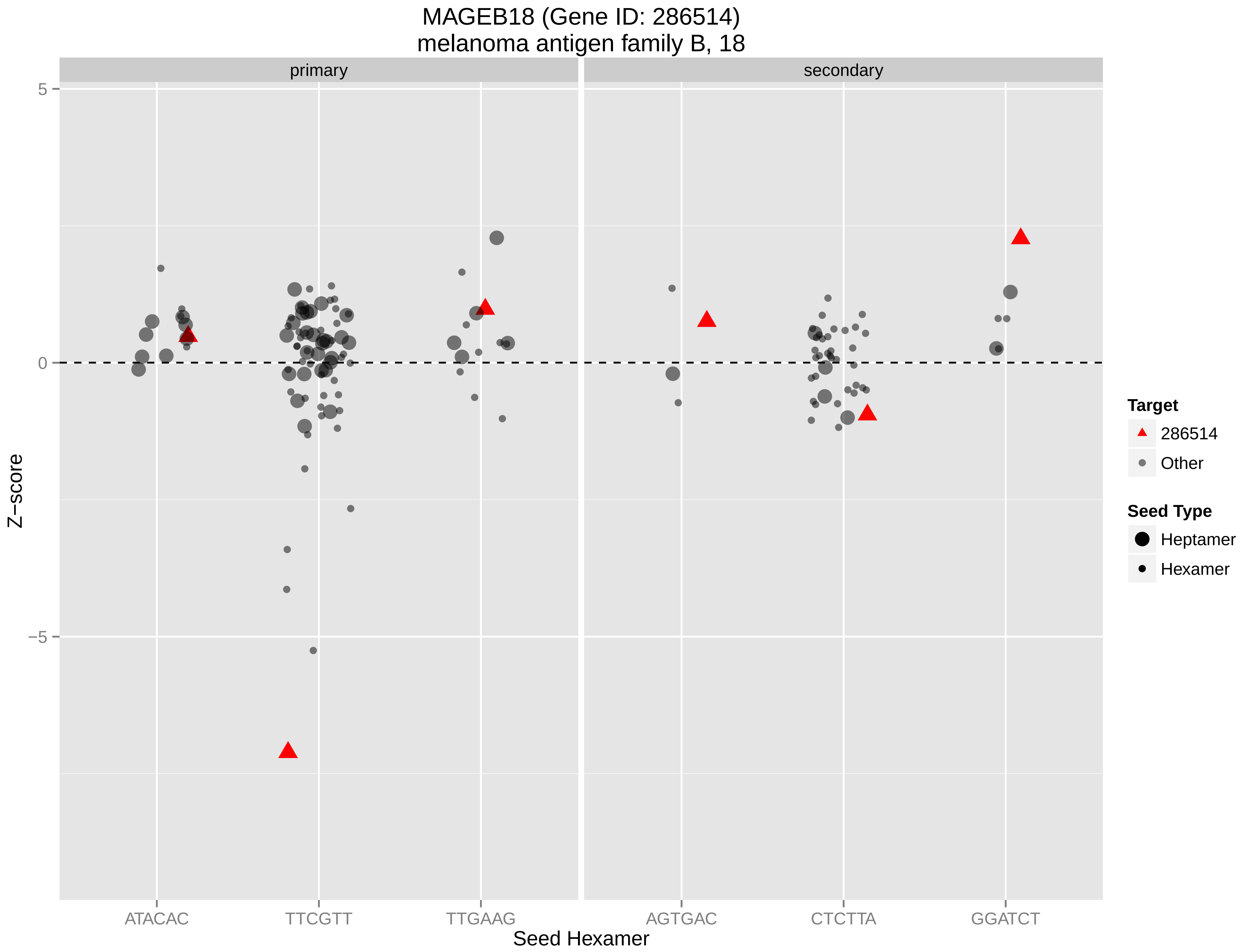
MAGEB18 (Gene ID: 286514)
melanoma antigen family B, 18
primary
secondary
5
0
−5
Z−score
ATACAC
TTCGTT
TTGAAG
AGTGAC
CTCTTA
GGATCT
Seed Hexamer
Target
286514
Other
Seed Type
Heptamer
Hexamer

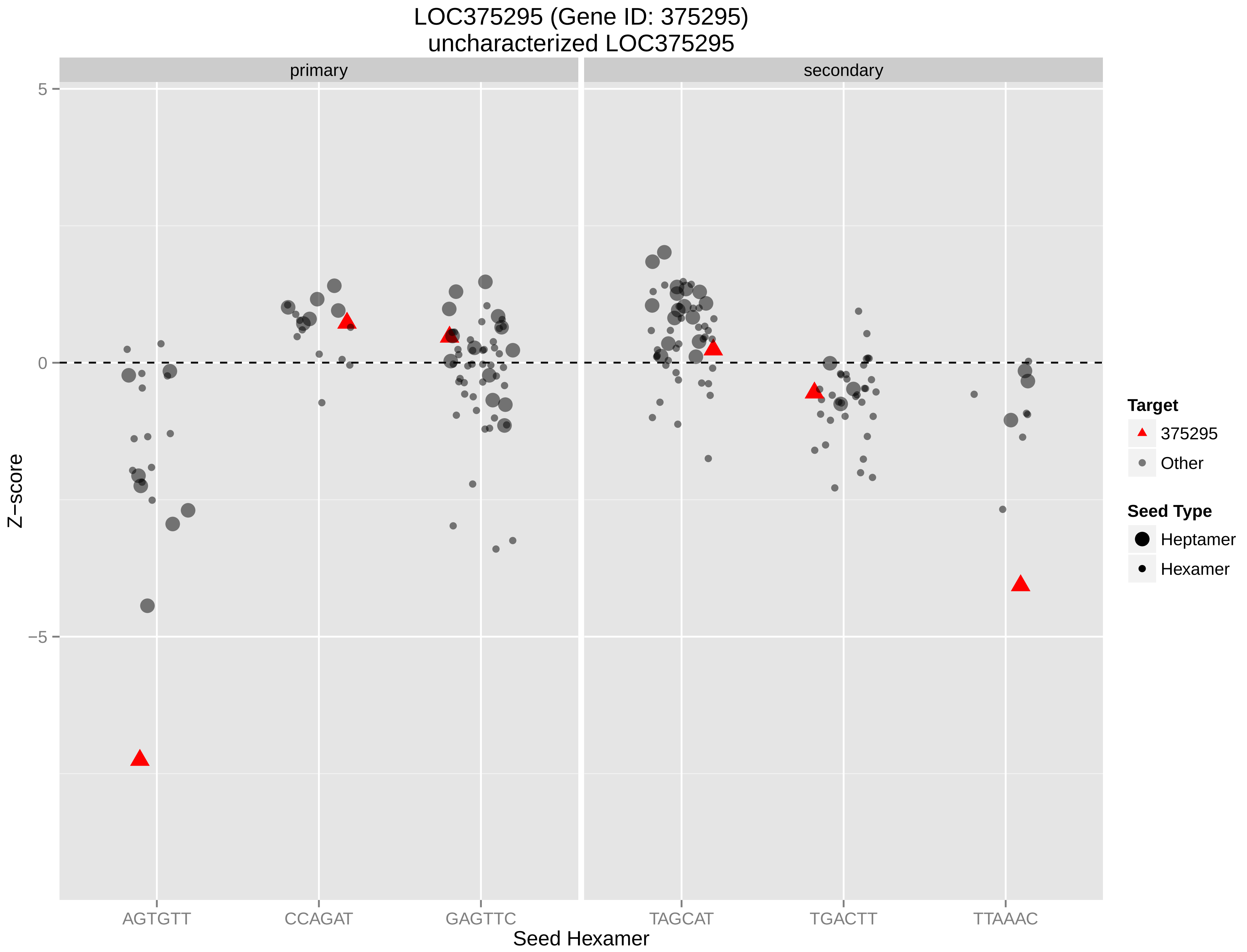

LOC375295 (Gene ID: 375295)
uncharacterized LOC375295
primary
secondary
5
0
−5
Z−score
AGTGTT
CCAGAT
GAGTTC
TAGCAT
TGACTT
TTAAAC
Seed Hexamer
Target
375295
Other
Seed Type
Heptamer
Hexamer

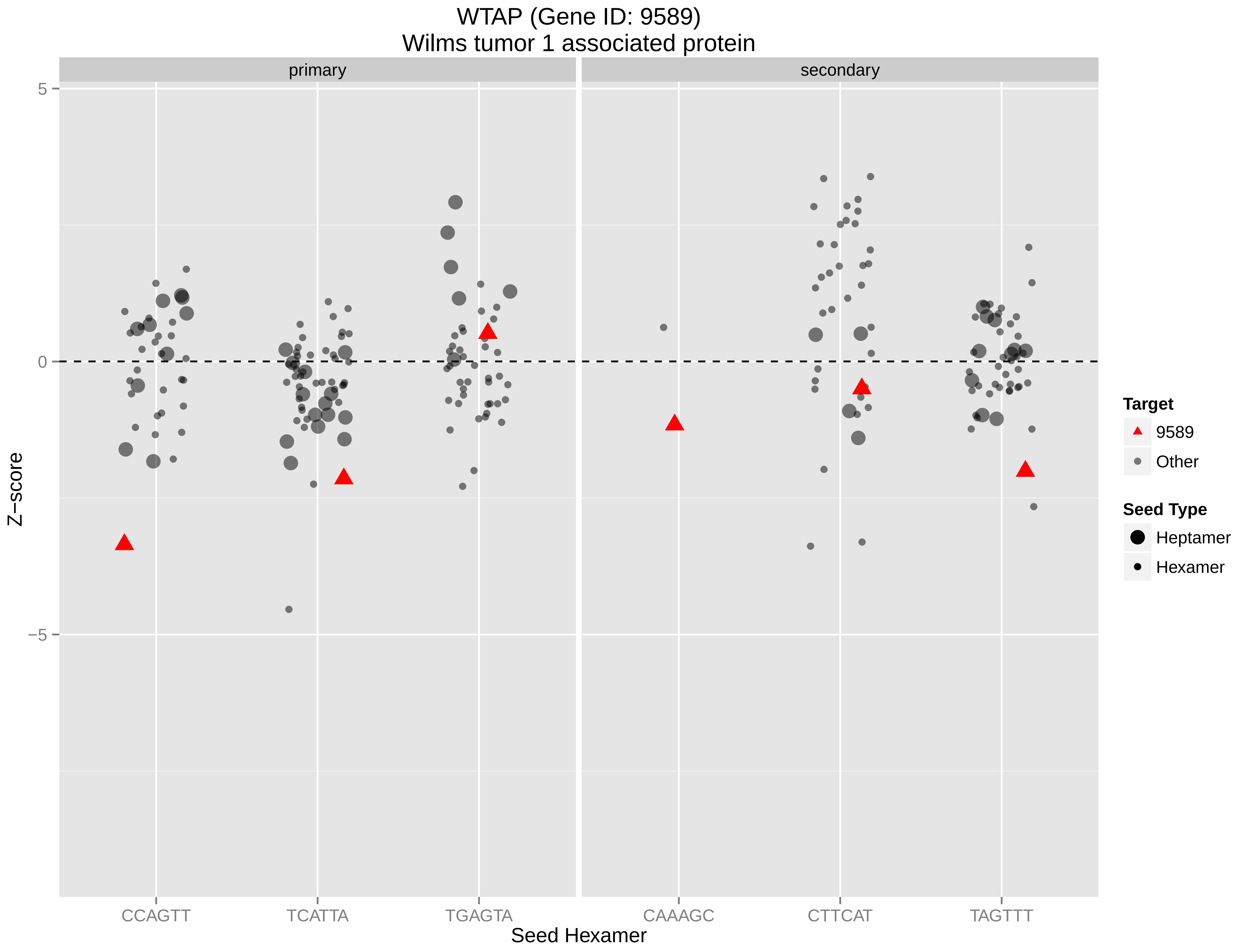

WTAP (Gene ID: 9589)
Wilms tumor 1 associated protein
primary
secondary
5
0
−5
Z−score
CCAGTT
TCATTA
TGAGTA
CAAAGC
CTTCAT
TAGTTT
Seed Hexamer
Target
9589
Other
Seed Type
Heptamer
Hexamer

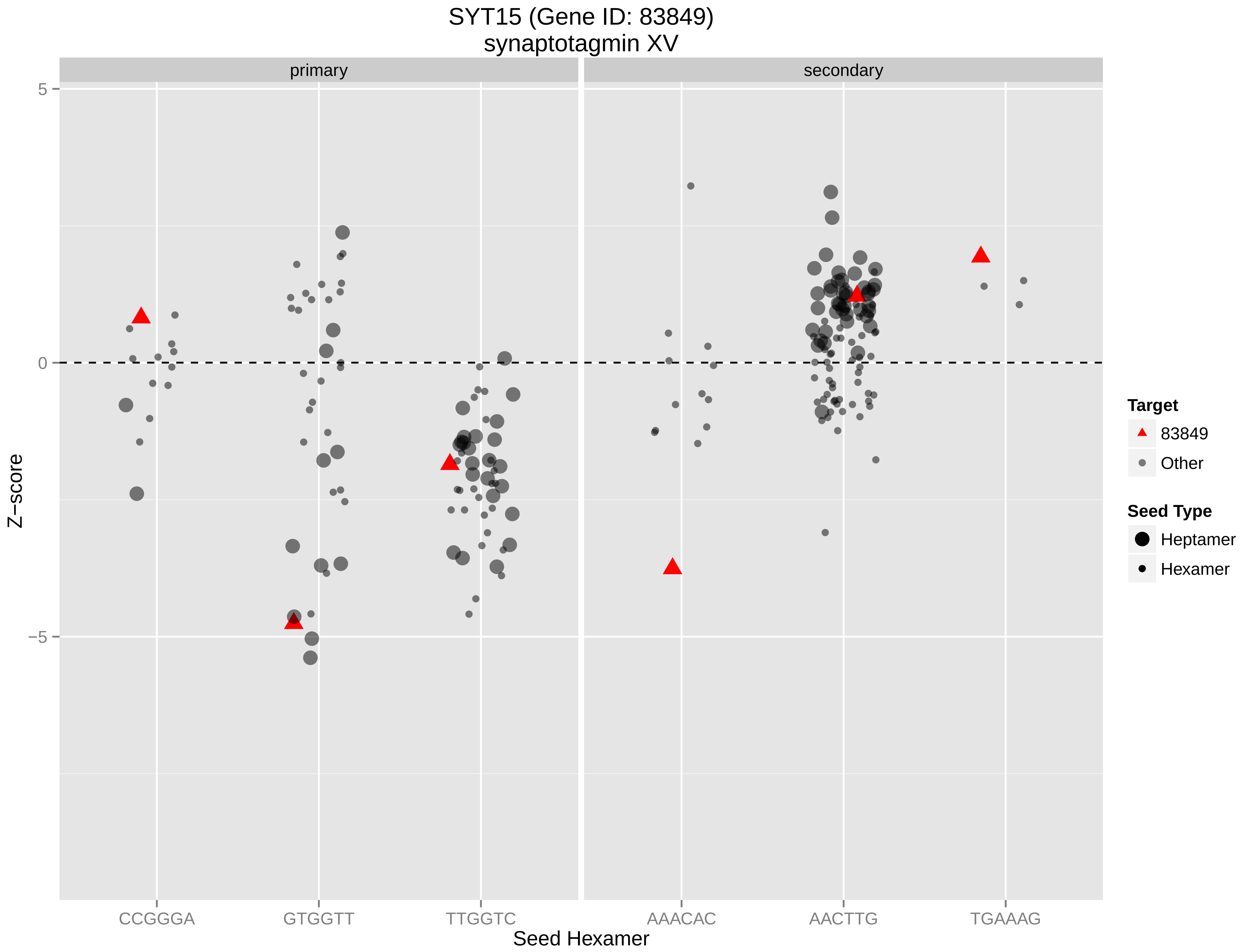

SYT15 (Gene ID: 83849)
synaptotagmin XV
primary
secondary
5
0
−5
Z−score
CCGGGA
GTGGTT
TTGGTC
AAACAC
AACTTG
TGAAAG
Seed Hexamer
Target
83849
Other
Seed Type
Heptamer
Hexamer

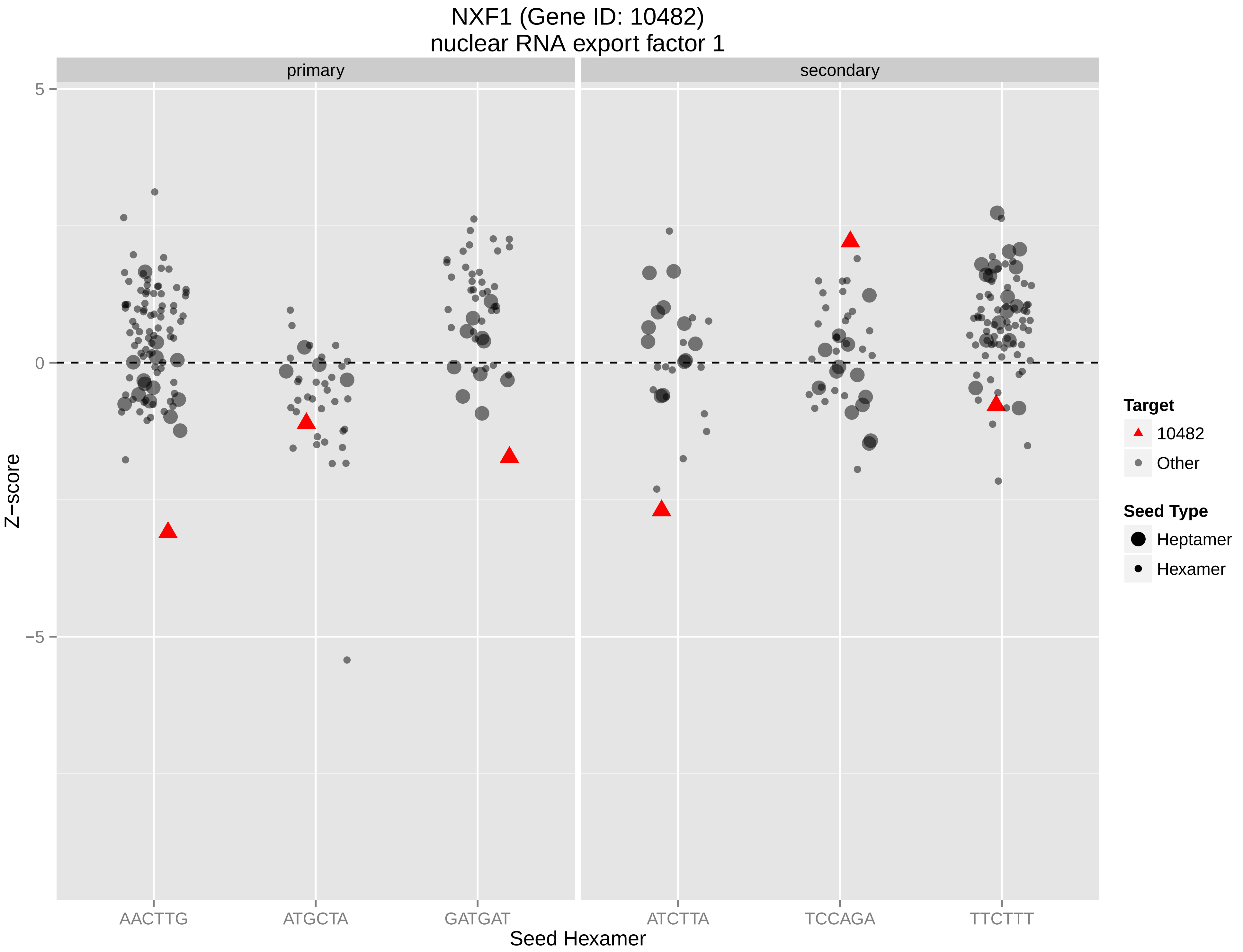
NXF1 (Gene ID: 10482)
nuclear RNA export factor 1
primary
secondary
5
0
−5
Z−score
AACTTG
ATGCTA
GATGAT
ATCTTA
TCCAGA
TTCTTT
Seed Hexamer
Target
10482
Other
Seed Type
Heptamer
Hexamer

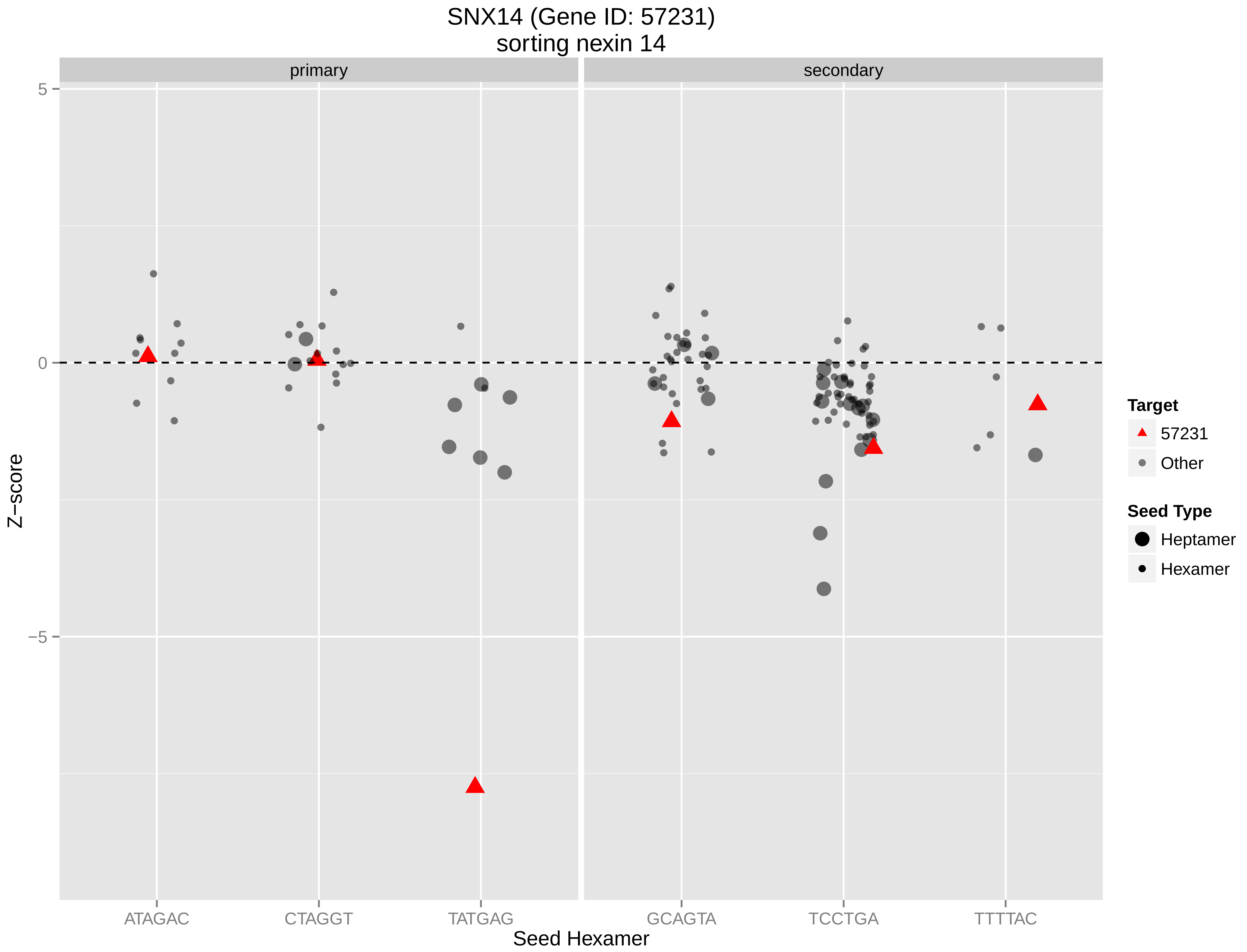

SNX14 (Gene ID: 57231)
sorting nexin 14
primary
secondary
5
0
−5
Z−score
ATAGAC
CTAGGT
TATGAG
GCAGTA
TCCTGA
TTTTAC
Seed Hexamer
Target
57231
Other
Seed Type
Heptamer
Hexamer

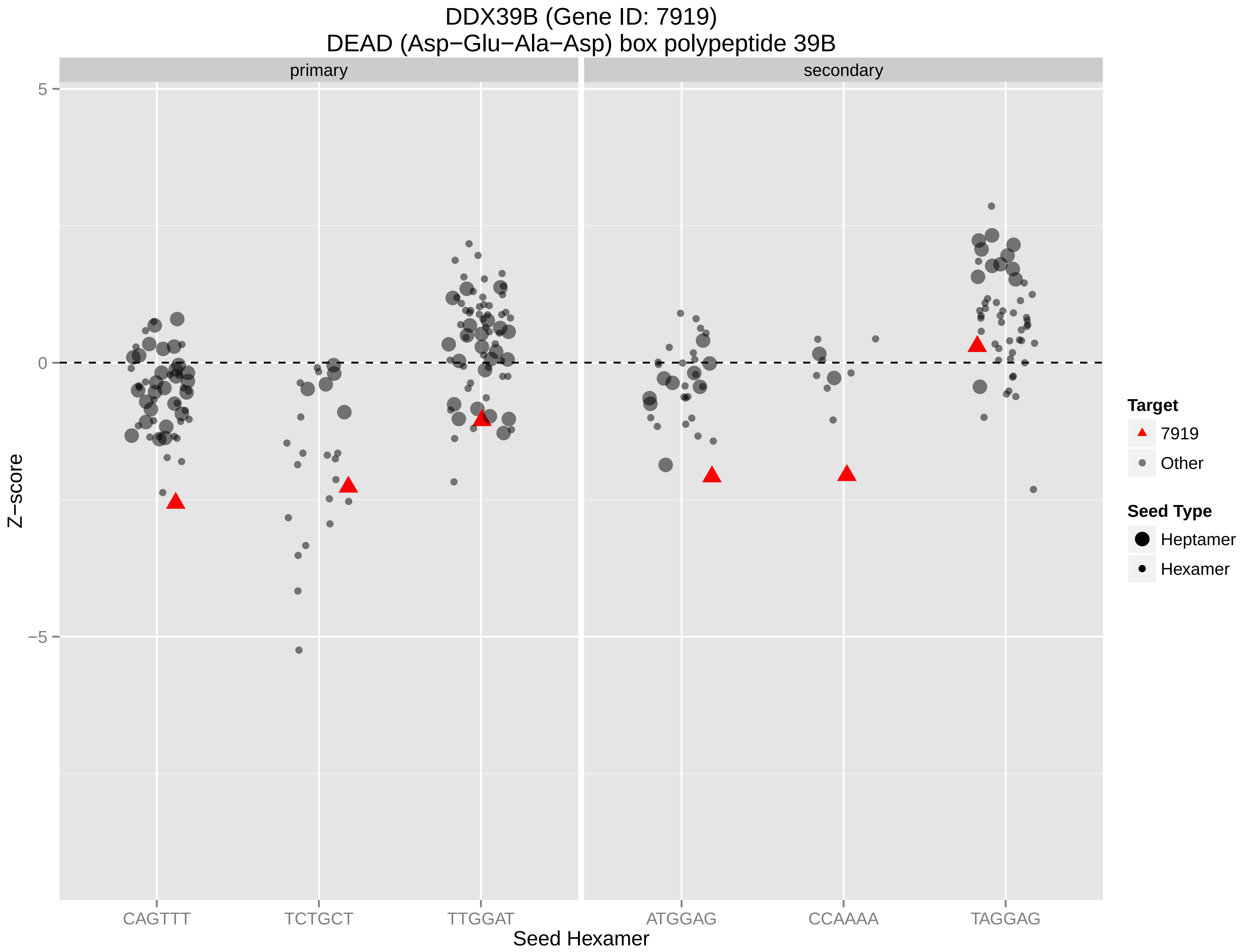

DDX39B (Gene ID: 7919)
DEAD (Asp−Glu−Ala−Asp) box polypeptide 39B
primary
secondary
5
0
−5
Z−score
CAGTTT
TCTGCT
TTGGAT
ATGGAG
CCAAAA
TAGGAG
Seed Hexamer
Target
7919
Other
Seed Type
Heptamer
Hexamer

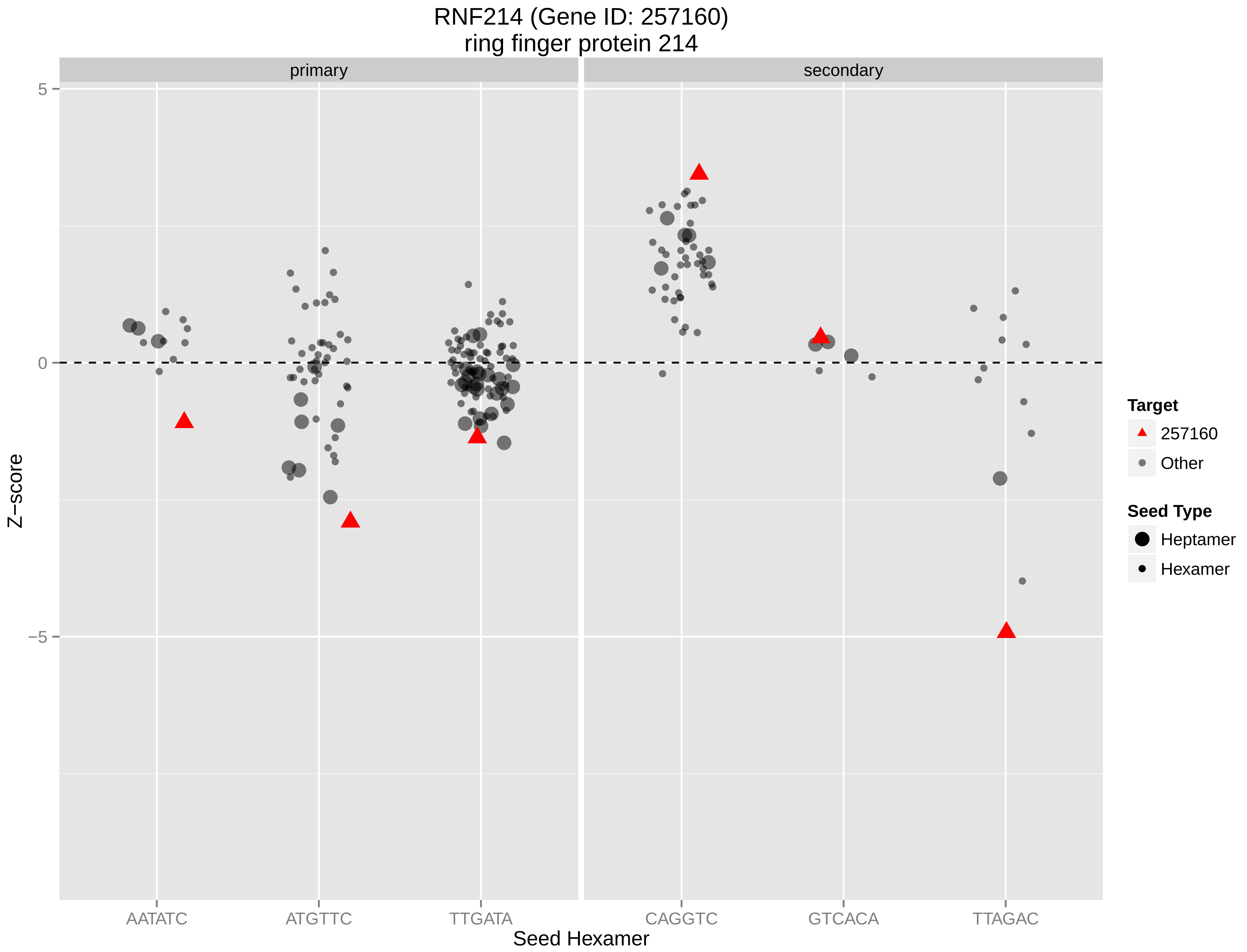
RNF214 (Gene ID: 257160)
ring finger protein 214
primary
secondary
5
0
−5
Z−score
AATATC
ATGTTC
TTGATA
CAGGTC
GTCACA
TTAGAC
Seed Hexamer
Target
257160
Other
Seed Type
Heptamer
Hexamer

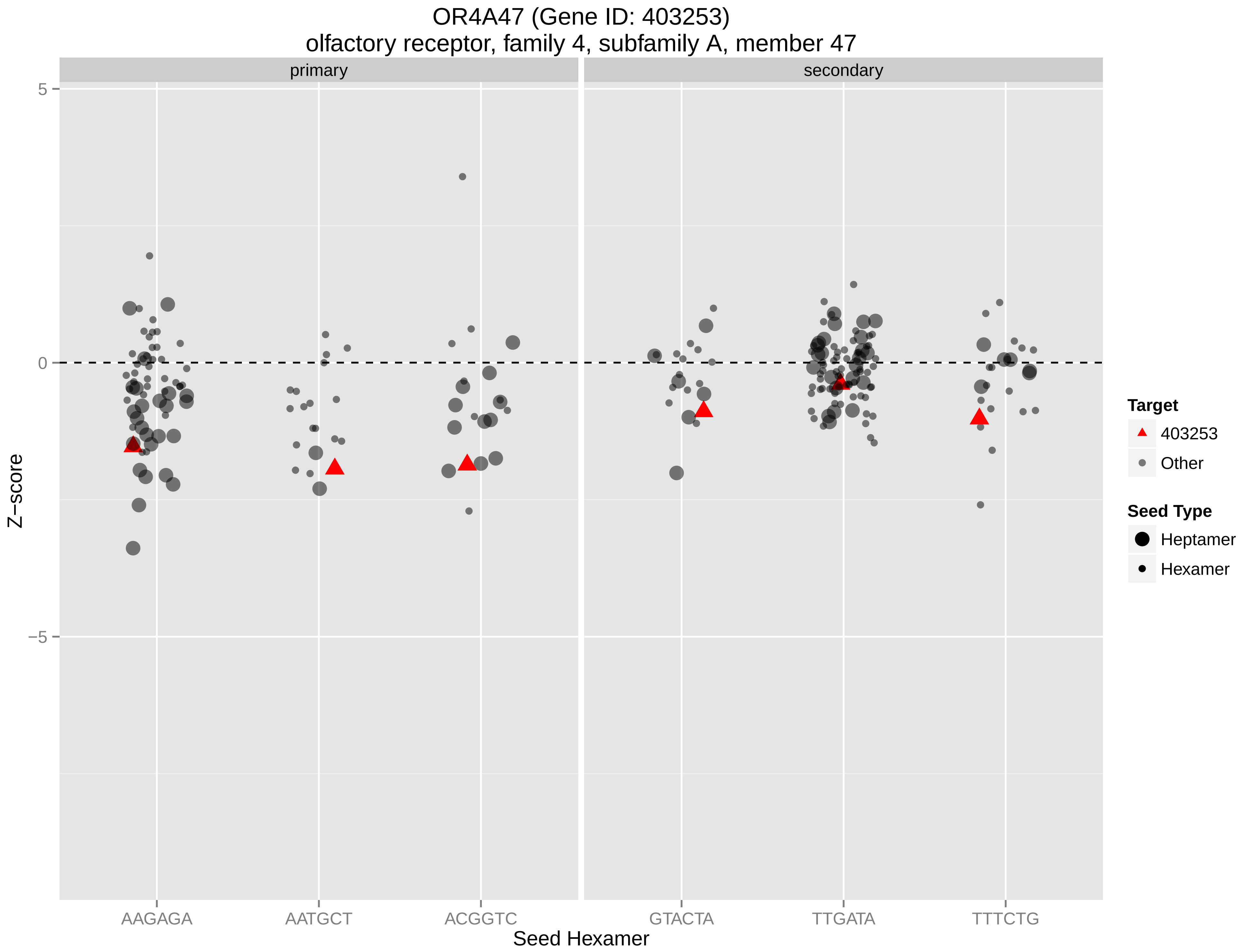
OR4A47 (Gene ID: 403253)
olfactory receptor, family 4, subfamily A, member 47
primary
secondary
5
0
−5
Z−score
AAGAGA
AATGCT
ACGGTC
GTACTA
TTGATA
TTTCTG
Seed Hexamer
Target
403253
Other
Seed Type
Heptamer
Hexamer

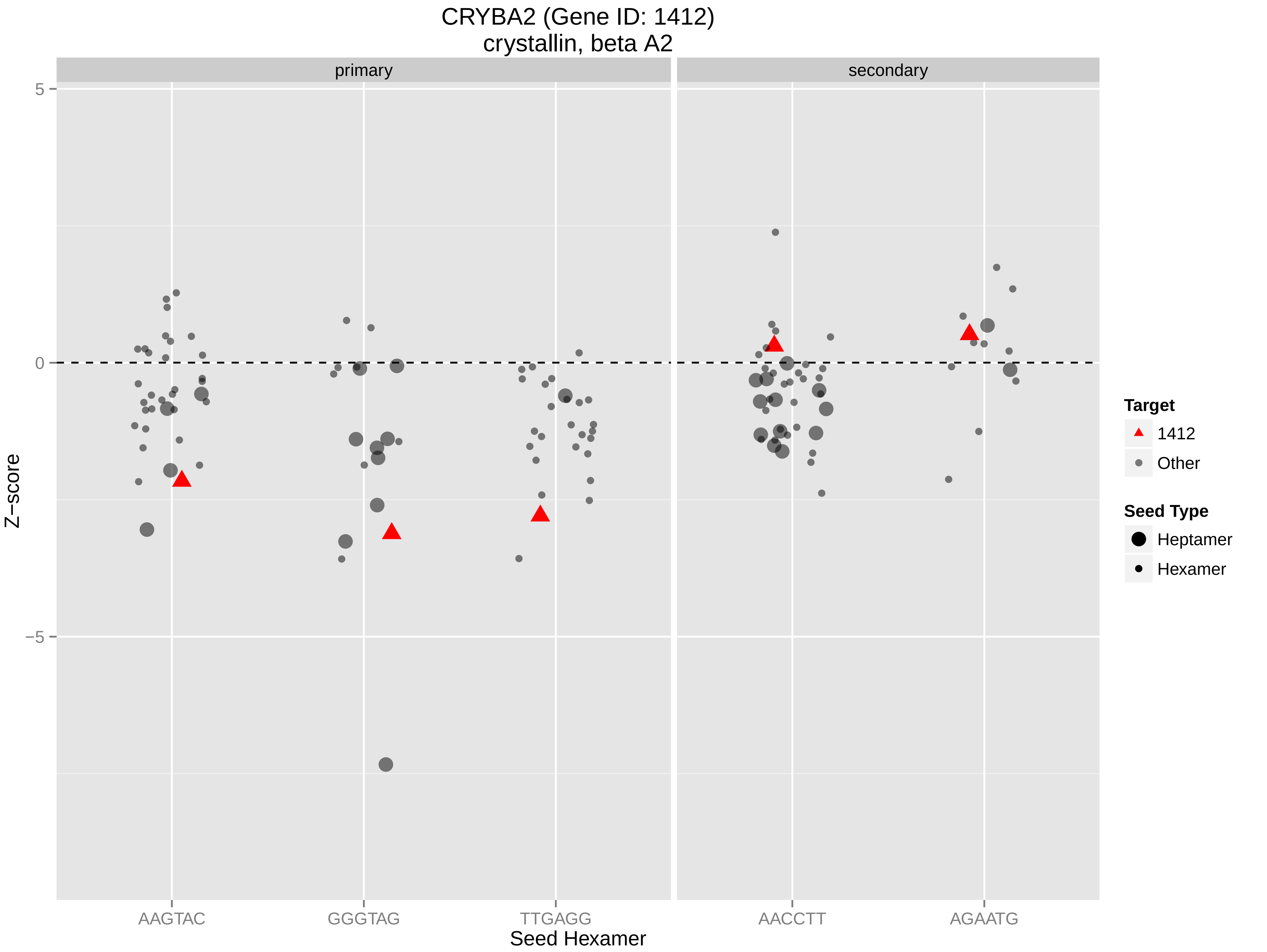
CRYBA2 (Gene ID: 1412)
crystallin, beta A2
primary
secondary
5
0
−5
Z−score
AAGTAC
GGGTAG
TTGAGG
AACCTT
AGAATG
Seed Hexamer
Target
1412
Other
Seed Type
Heptamer
Hexamer

FAM24B (Gene ID: 196792)
family with sequence similarity 24, member B

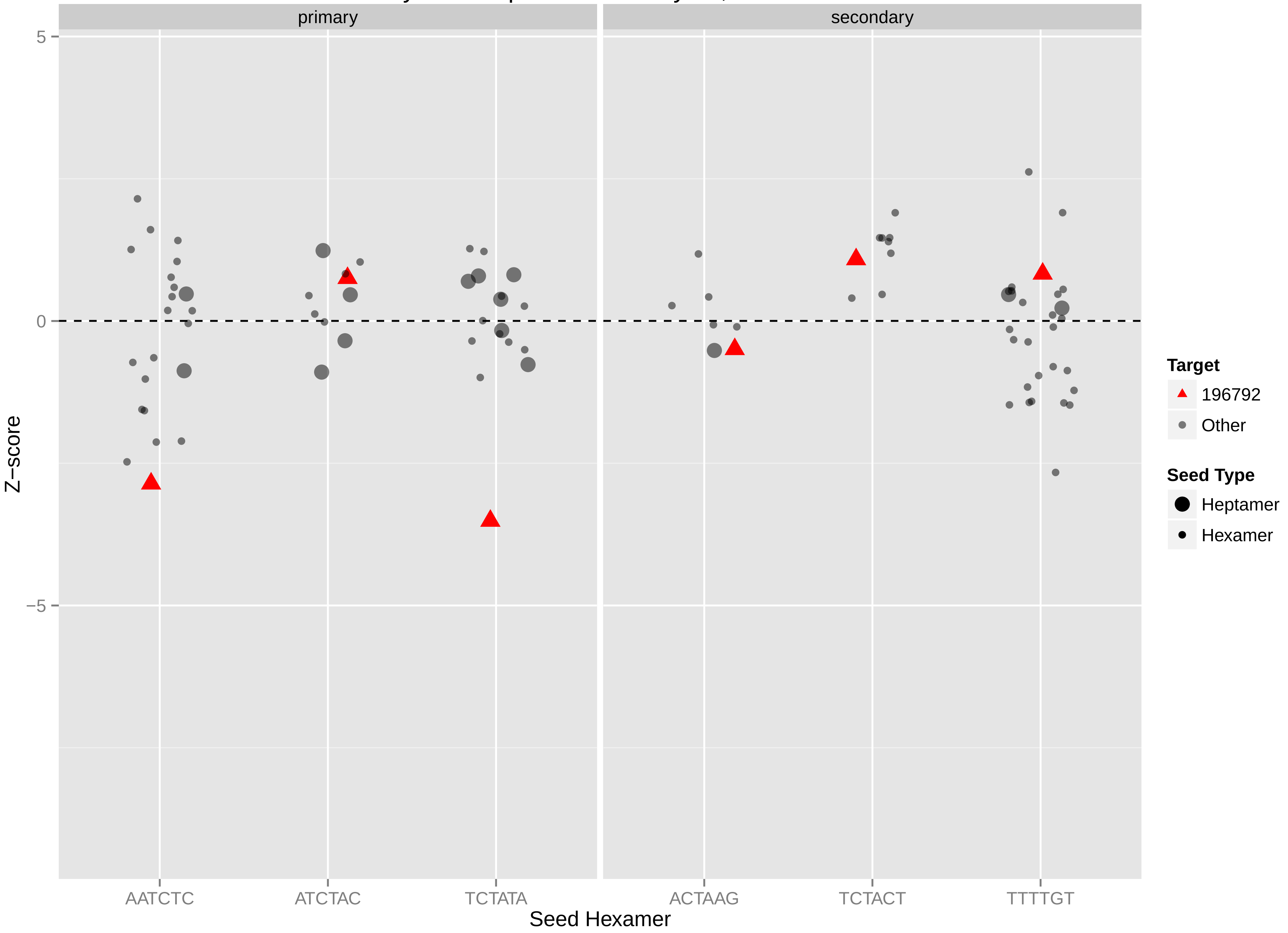

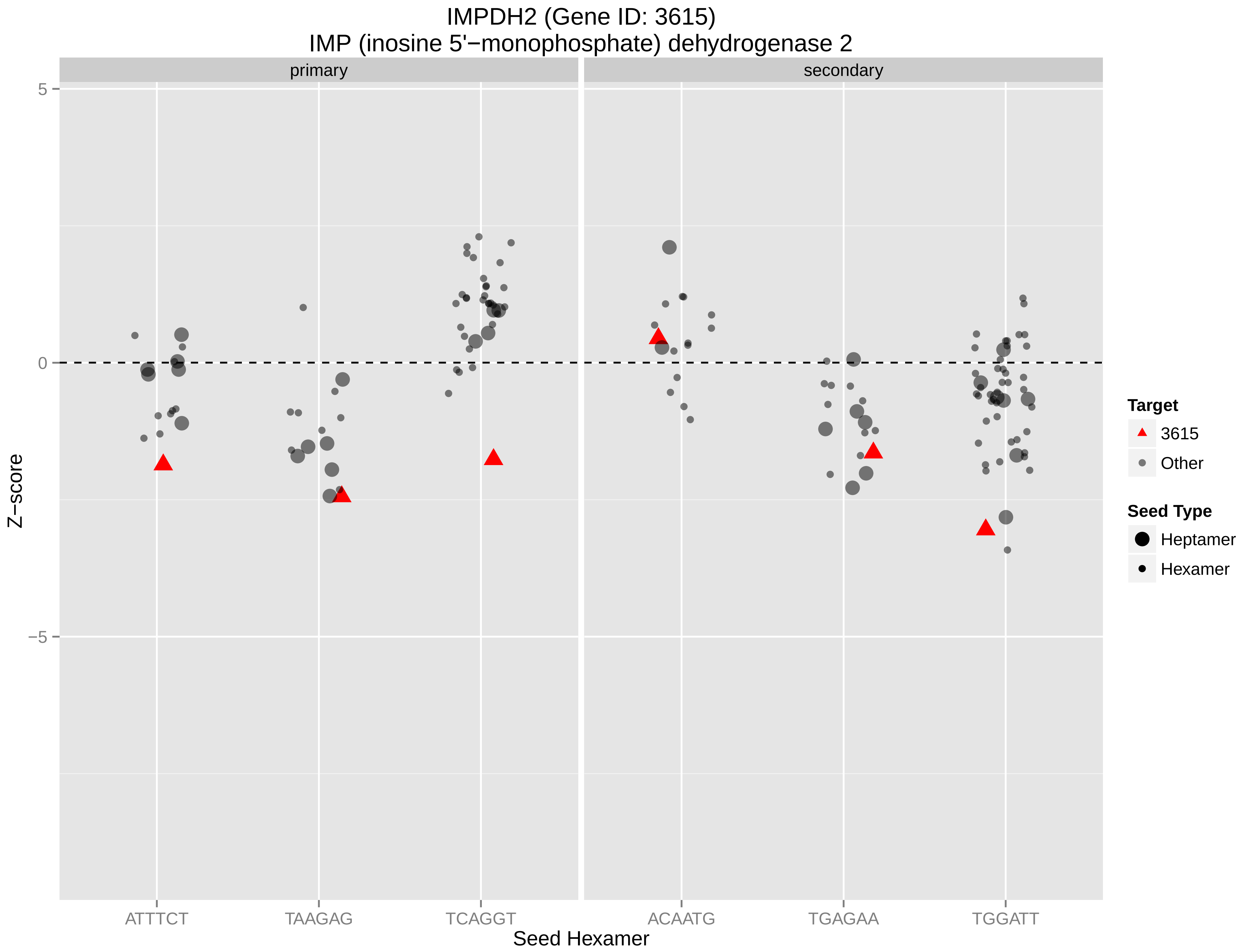
IMPDH2 (Gene ID: 3615)
IMP (inosine 5'−monophosphate) dehydrogenase 2
primary
secondary
5
0
−5
Z−score
ATTTCT
TAAGAG
TCAGGT
ACAATG
TGAGAA
TGGATT
Seed Hexamer
Target
3615
Other
Seed Type
Heptamer
Hexamer

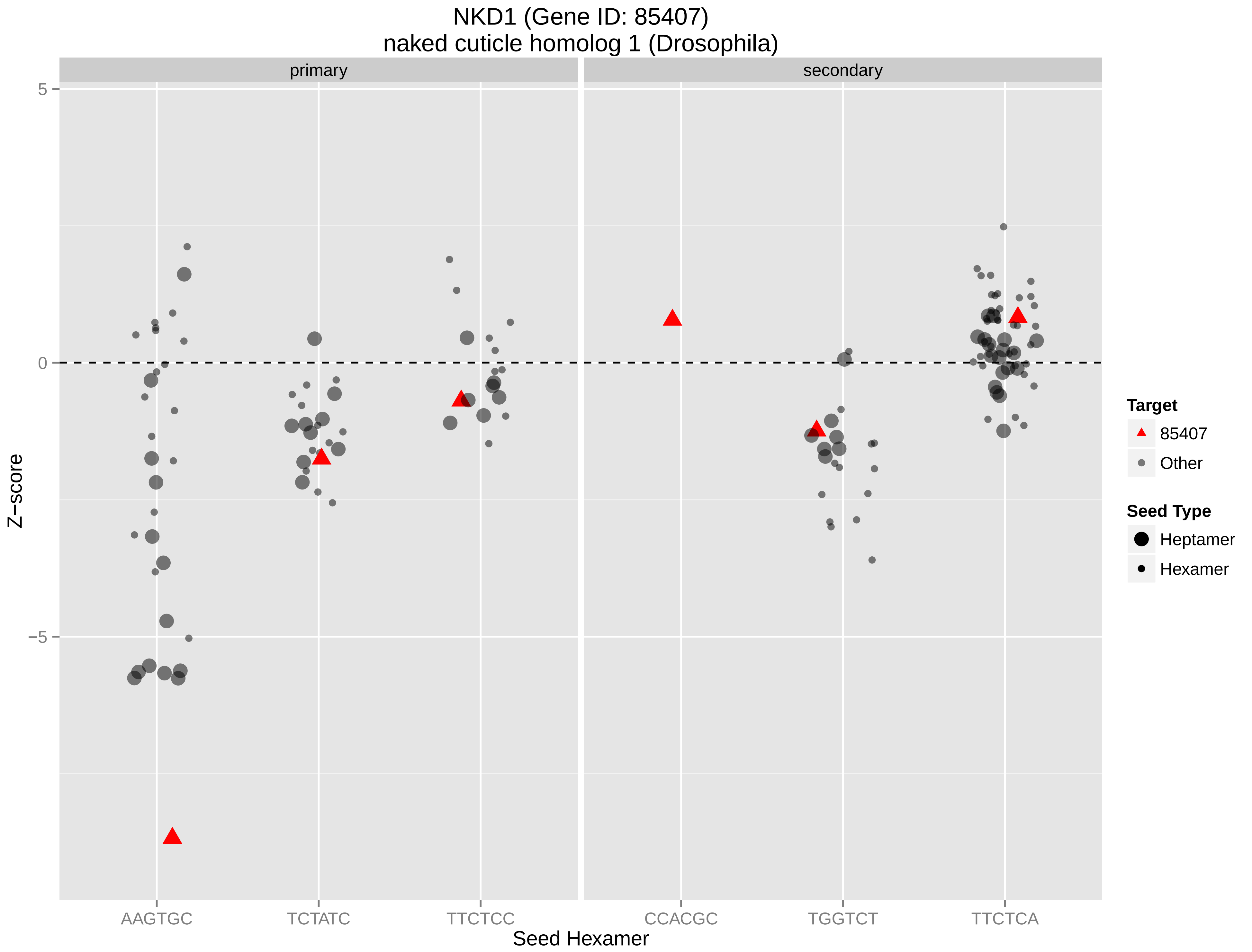

NKD1 (Gene ID: 85407)
naked cuticle homolog 1 (Drosophila)
primary
secondary
5
0
−5
Z−score
AAGTGC
TCTATC
TTCTCC
CCACGC
TGGTCT
TTCTCA
Seed Hexamer
Target
85407
Other
Seed Type
Heptamer
Hexamer

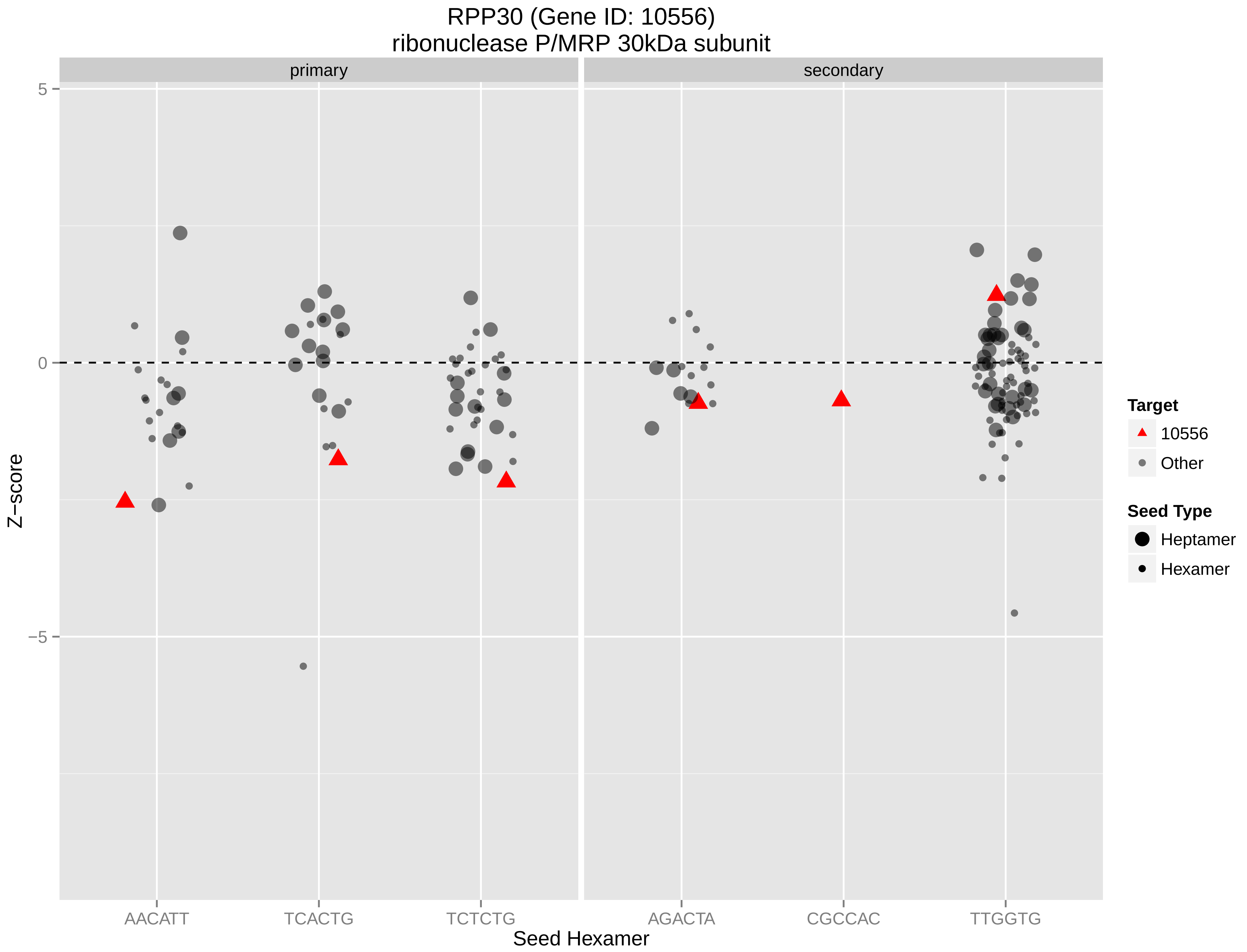

RPP30 (Gene ID: 10556)
ribonuclease P/MRP 30kDa subunit
primary
secondary
5
0
−5
Z−score
AACATT
TCACTG
TCTCTG
AGACTA
CGCCAC
TTGGTG
Seed Hexamer
Target
10556
Other
Seed Type
Heptamer
Hexamer

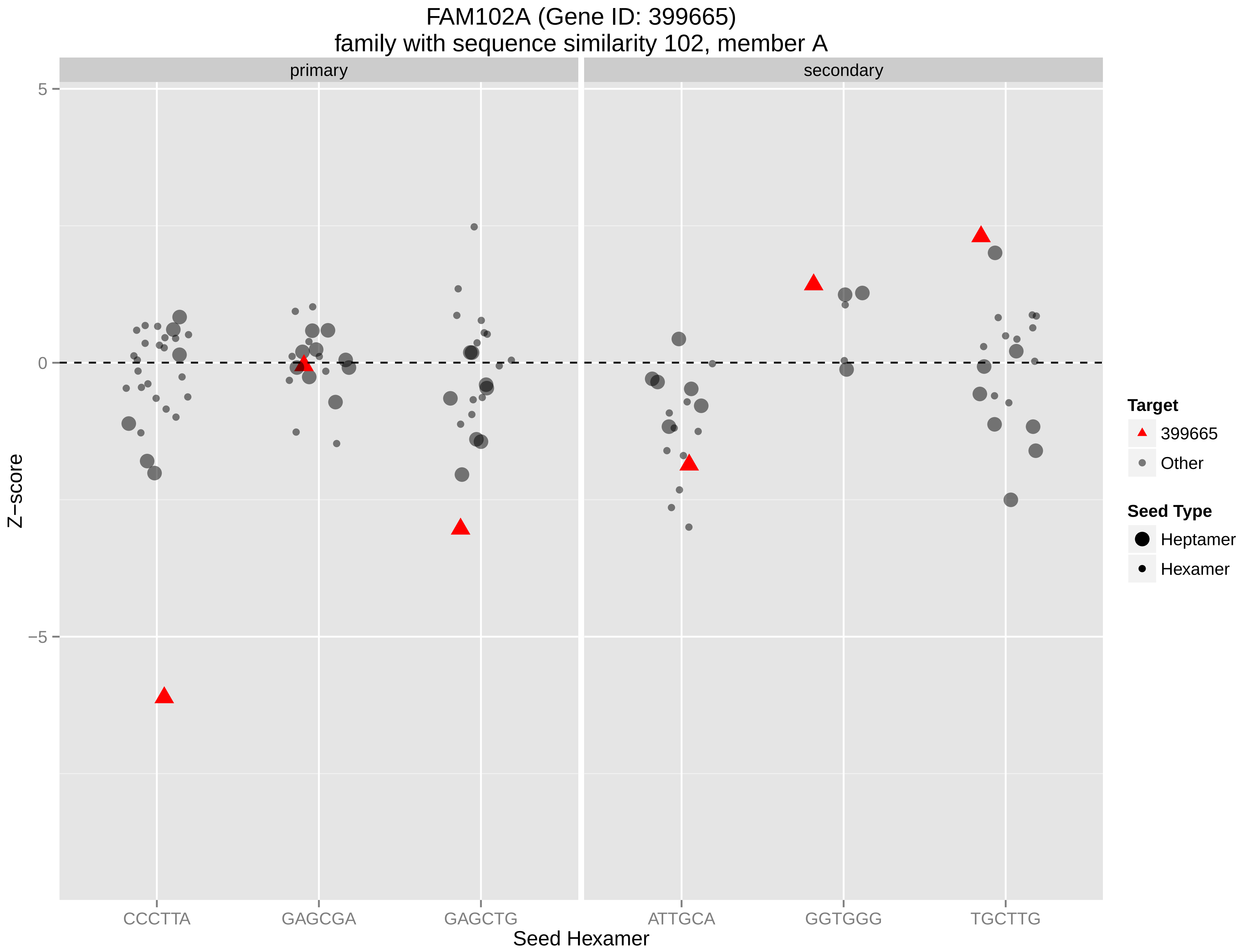
FAM102A (Gene ID: 399665)
family with sequence similarity 102, member A
primary
secondary
5
0
−5
Z−score
CCCTTA
GAGCGA
GAGCTG
ATTGCA
GGTGGG
TGCTTG
Seed Hexamer
Target
399665
Other
Seed Type
Heptamer
Hexamer

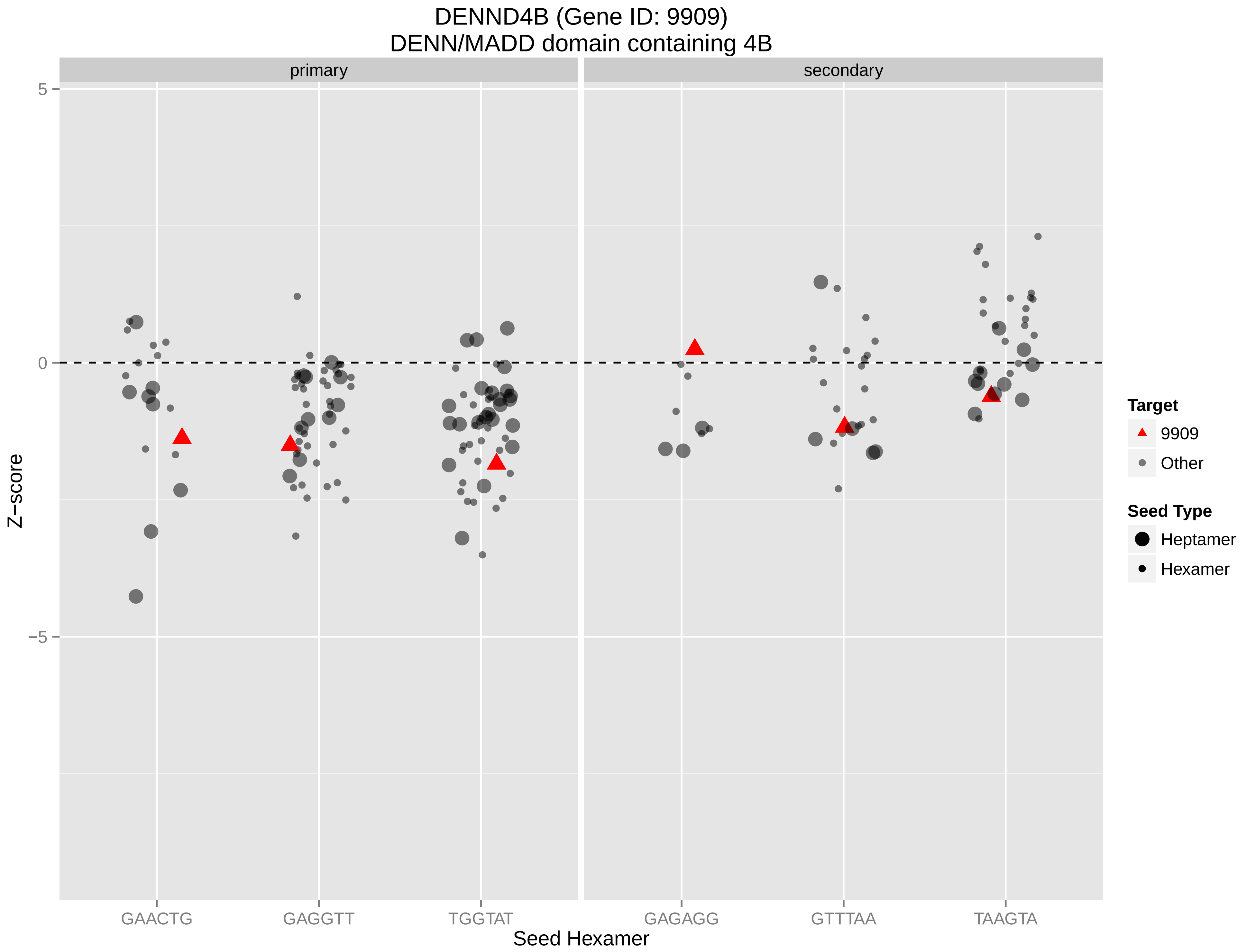
DENND4B (Gene ID: 9909)
DENN/MADD domain containing 4B
primary
secondary
5
0
−5
Z−score
GAACTG
GAGGTT
TGGTAT
GAGAGG
GTTTAA
TAAGTA
Seed Hexamer
Target
9909
Other
Seed Type
Heptamer
Hexamer

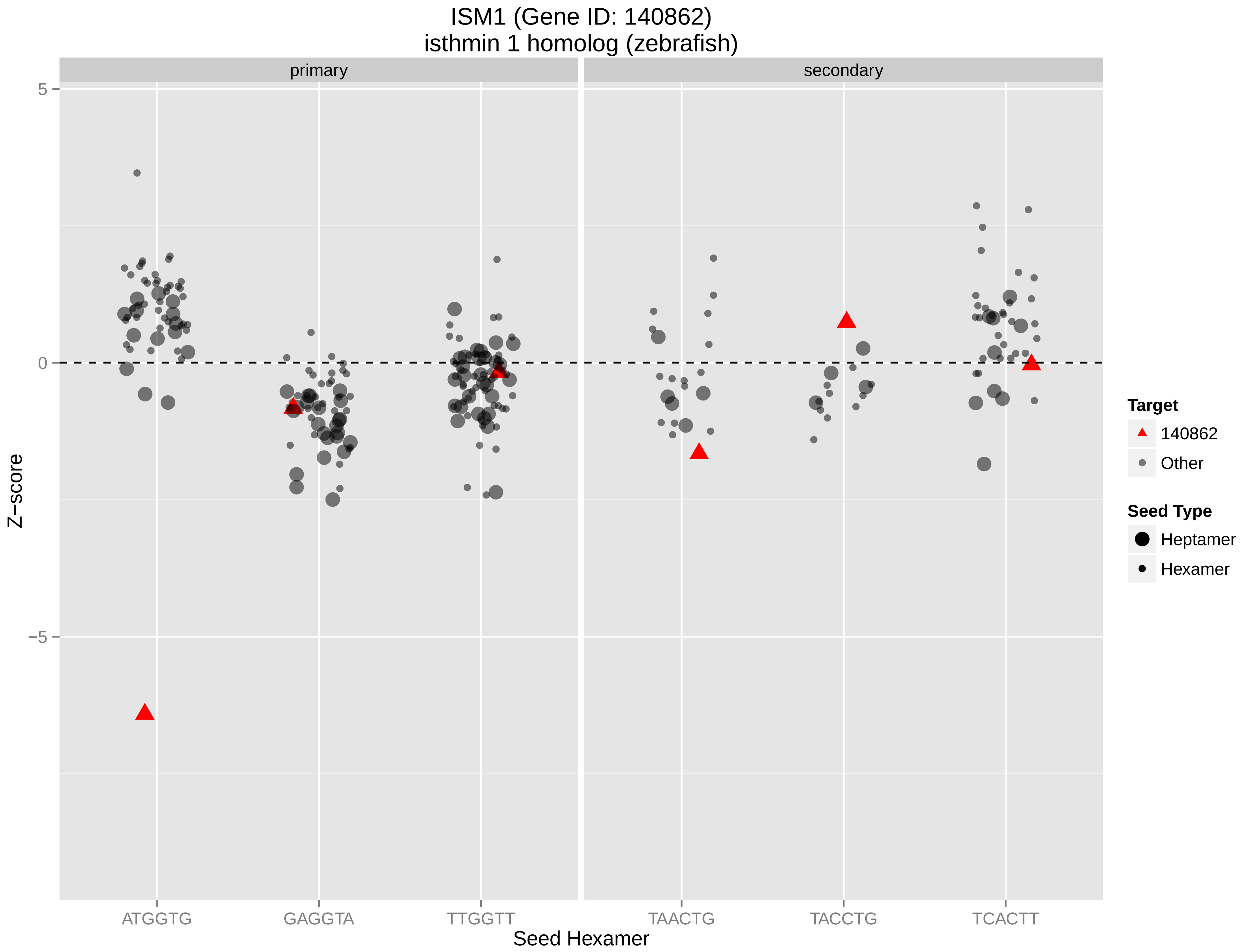

ISM1 (Gene ID: 140862)
isthmin 1 homolog (zebrafish)
primary
secondary
5
0
−5
Z−score
ATGGTG
GAGGTA
TTGGTT
TAACTG
TACCTG
TCACTT
Seed Hexamer
Target
140862
Other
Seed Type
Heptamer
Hexamer

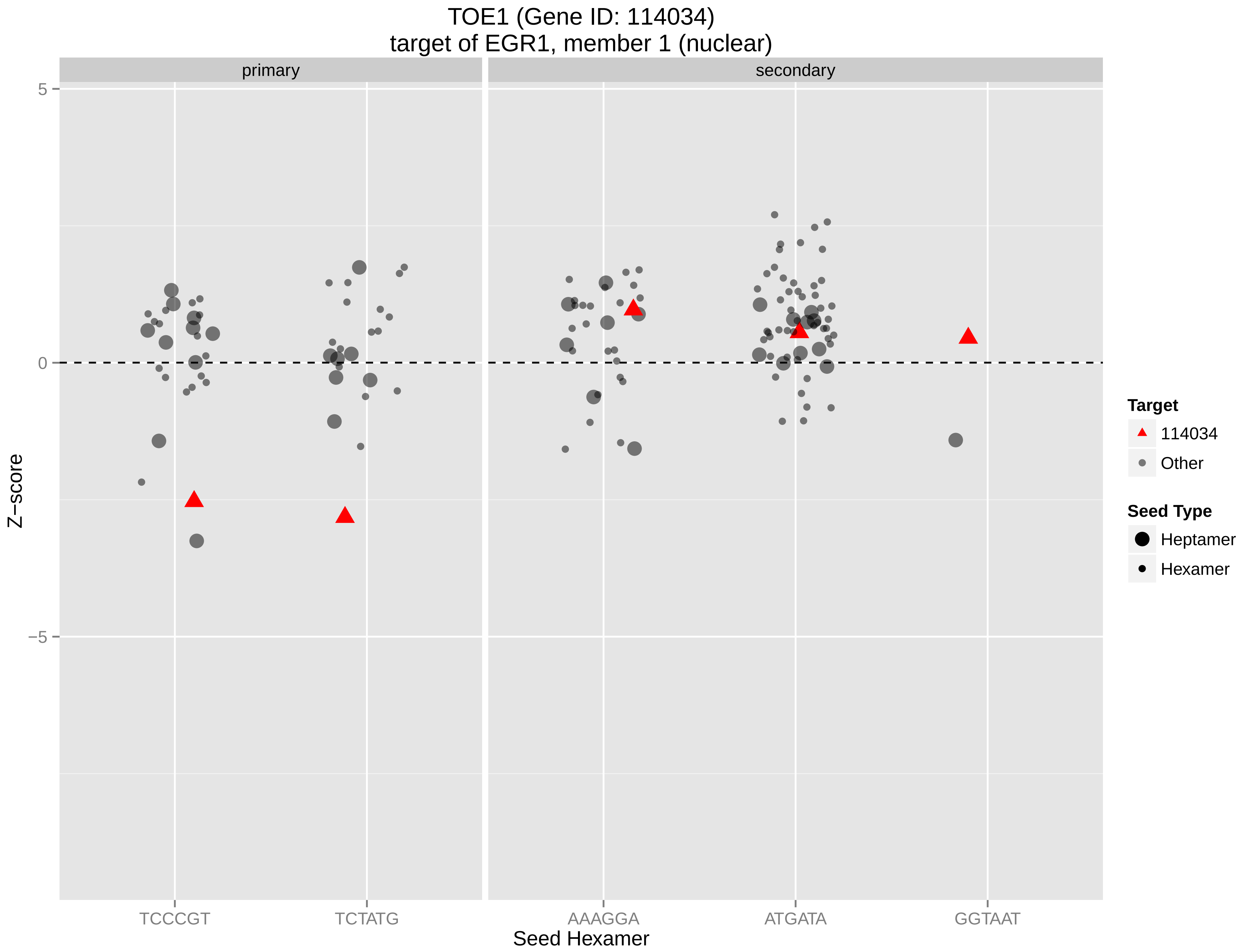
TOE1 (Gene ID: 114034)
target of EGR1, member 1 (nuclear)
primary
secondary
5
0
−5
Z−score
TCCCGT
TCTATG
AAAGGA
ATGATA
GGTAAT
Seed Hexamer
Target
114034
Other
Seed Type
Heptamer
Hexamer

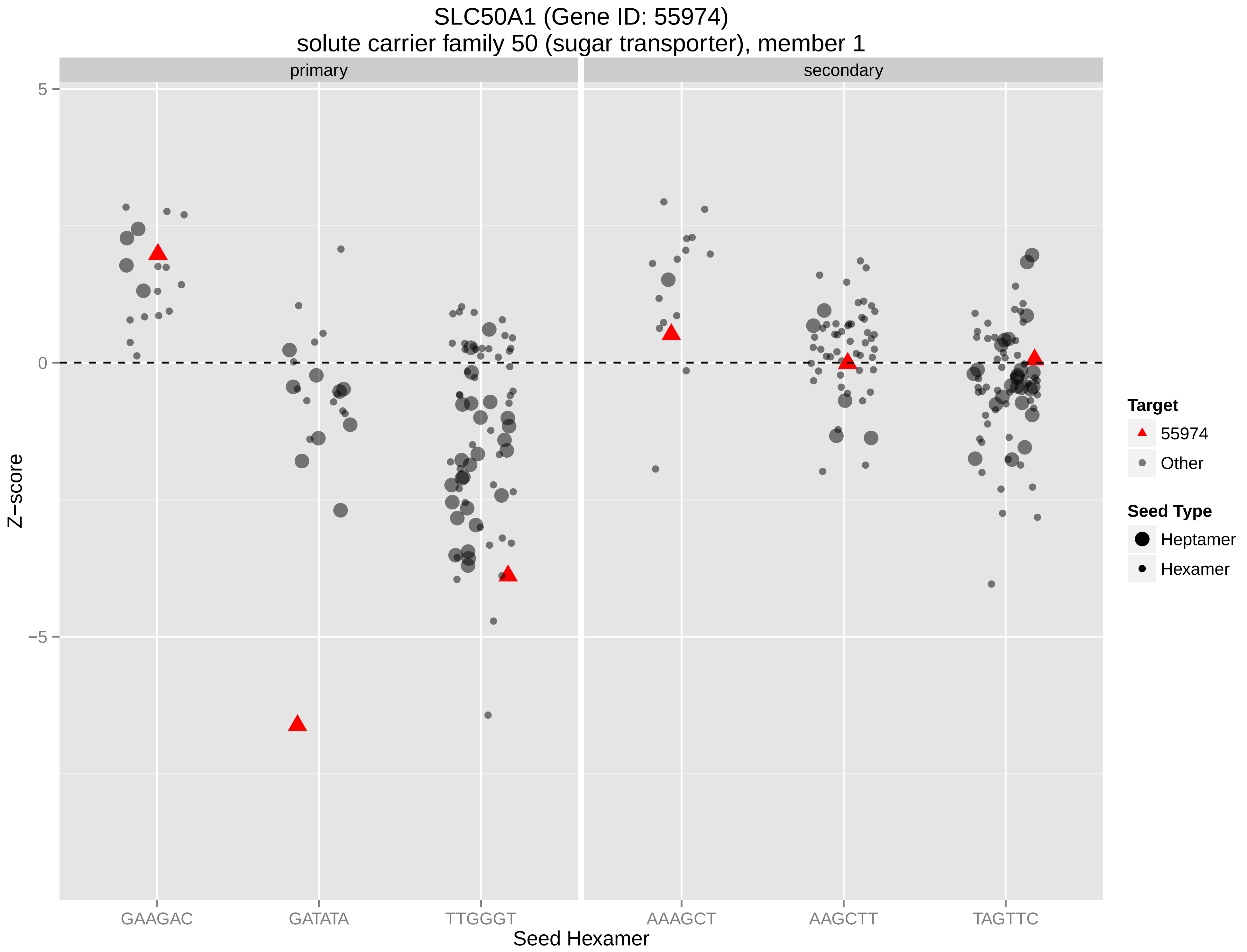

SLC50A1 (Gene ID: 55974)
solute carrier family 50 (sugar transporter), member 1
primary
secondary
5
0
−5
Z−score
GAAGAC
GATATA
TTGGGT
AAAGCT
AAGCTT
TAGTTC
Seed Hexamer
Target
55974
Other
Seed Type
Heptamer
Hexamer

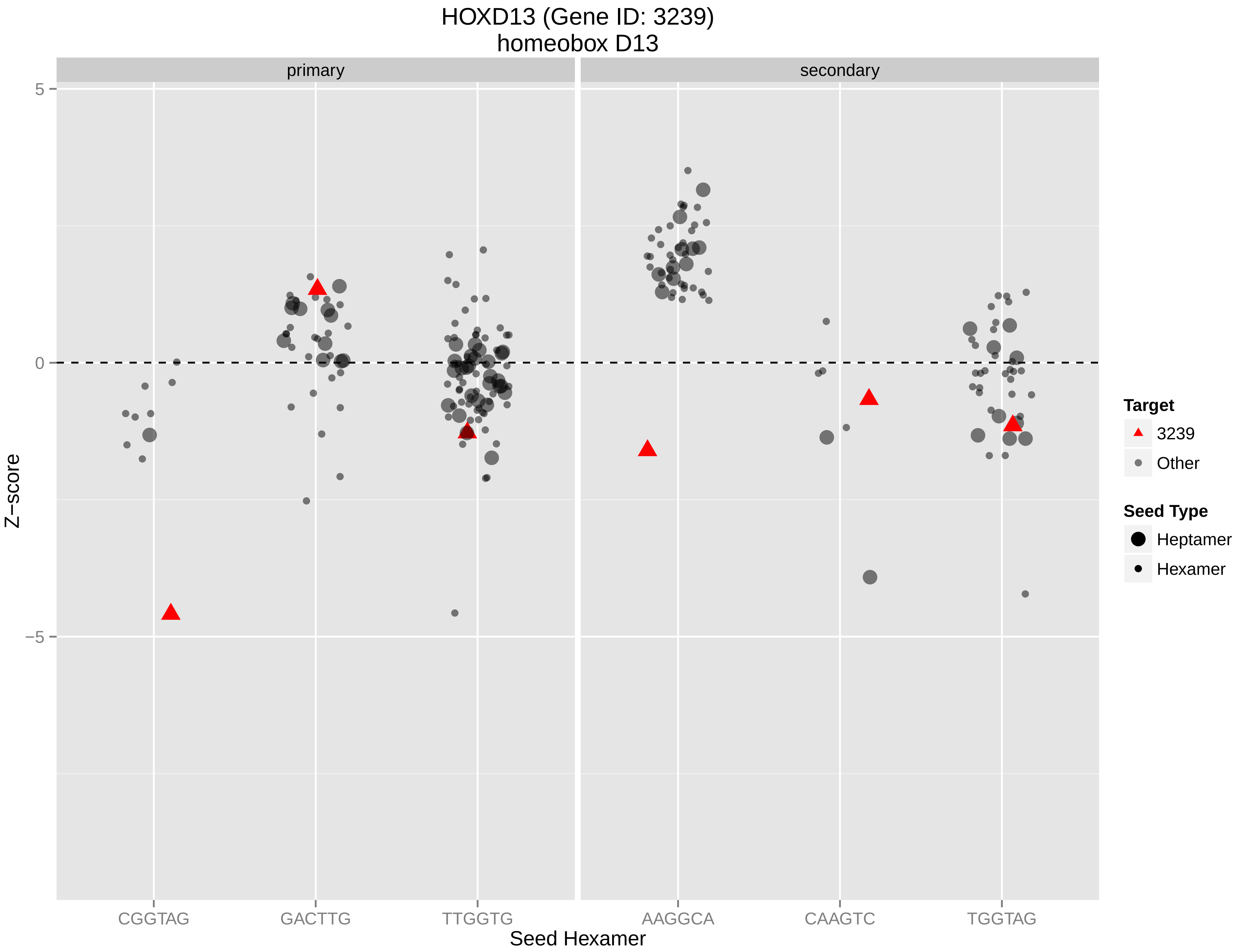

HOXD13 (Gene ID: 3239)
homeobox D13
primary
secondary
5
0
−5
Z−score
CGGTAG
GACTTG
TTGGTG
AAGGCA
CAAGTC
TGGTAG
Seed Hexamer
Target
3239
Other
Seed Type
Heptamer
Hexamer

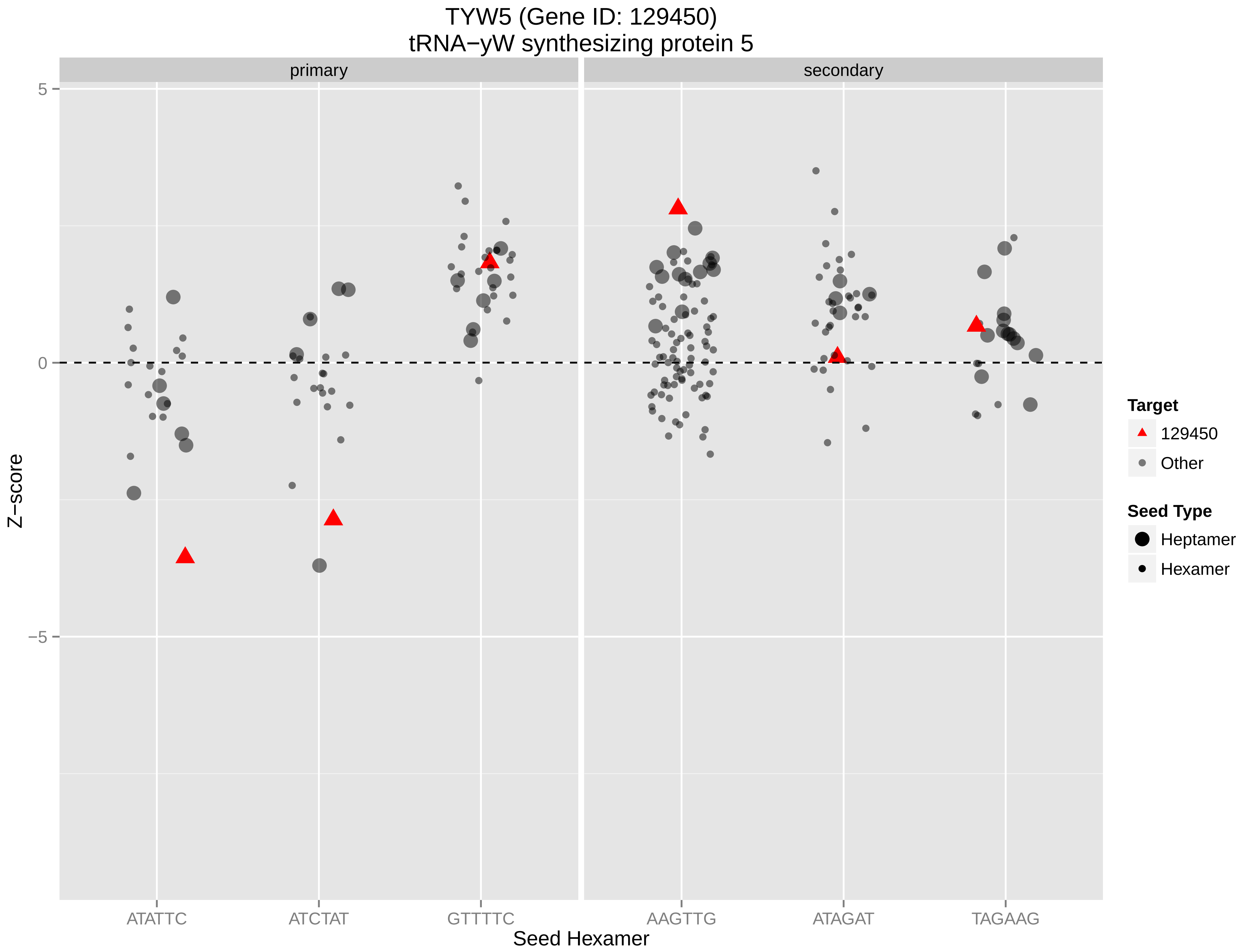
TYW5 (Gene ID: 129450)
tRNA−yW synthesizing protein 5
primary
secondary
5
0
−5
Z−score
ATATTC
ATCTAT
GTTTTC
AAGTTG
ATAGAT
TAGAAG
Seed Hexamer
Target
129450
Other
Seed Type
Heptamer
Hexamer

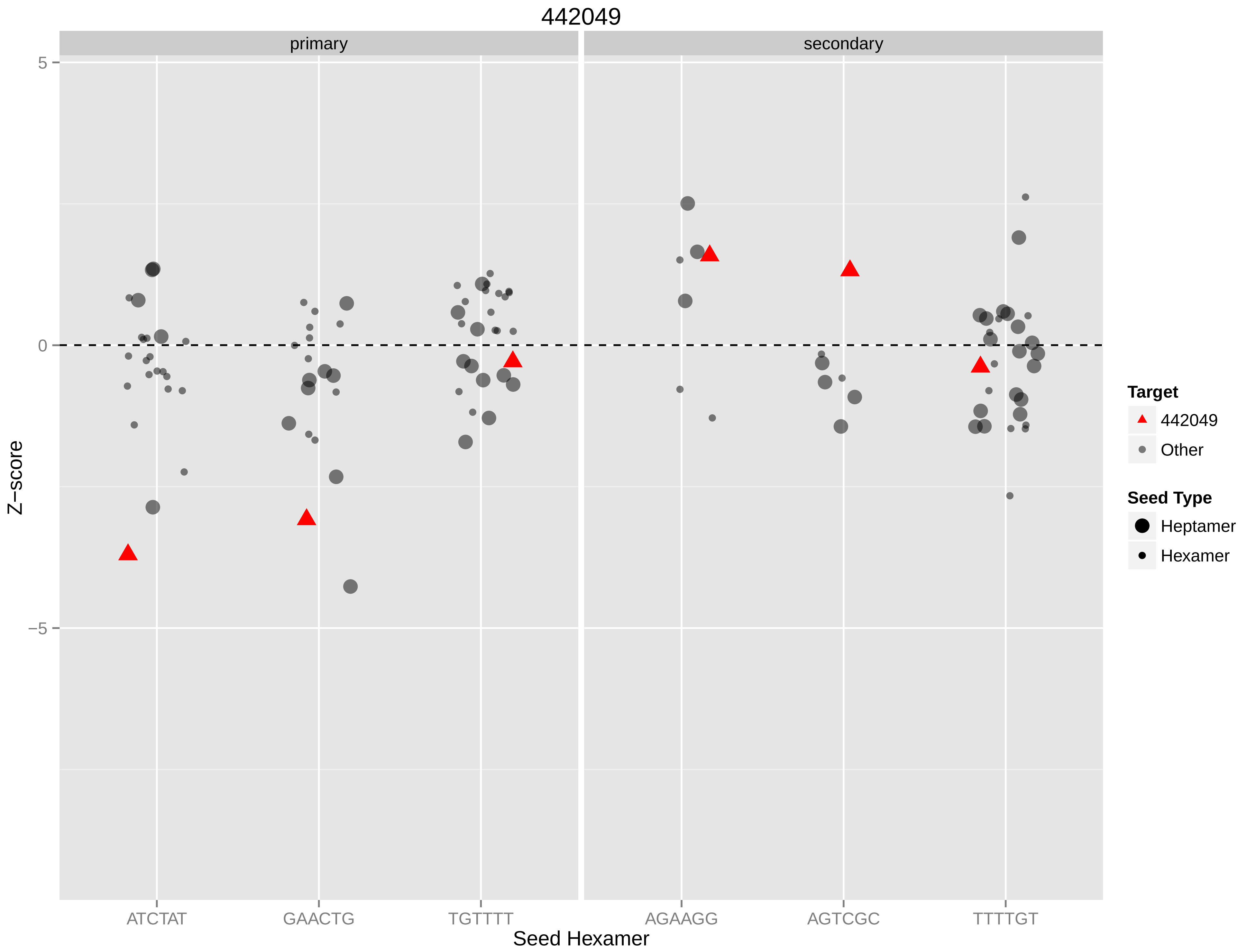
442049
primary
secondary
5
0
−5
Z−score
ATCTAT
GAACTG
TGTTTT
AGAAGG
AGTCGC
TTTTGT
Seed Hexamer
Target
442049
Other
Seed Type
Heptamer
Hexamer

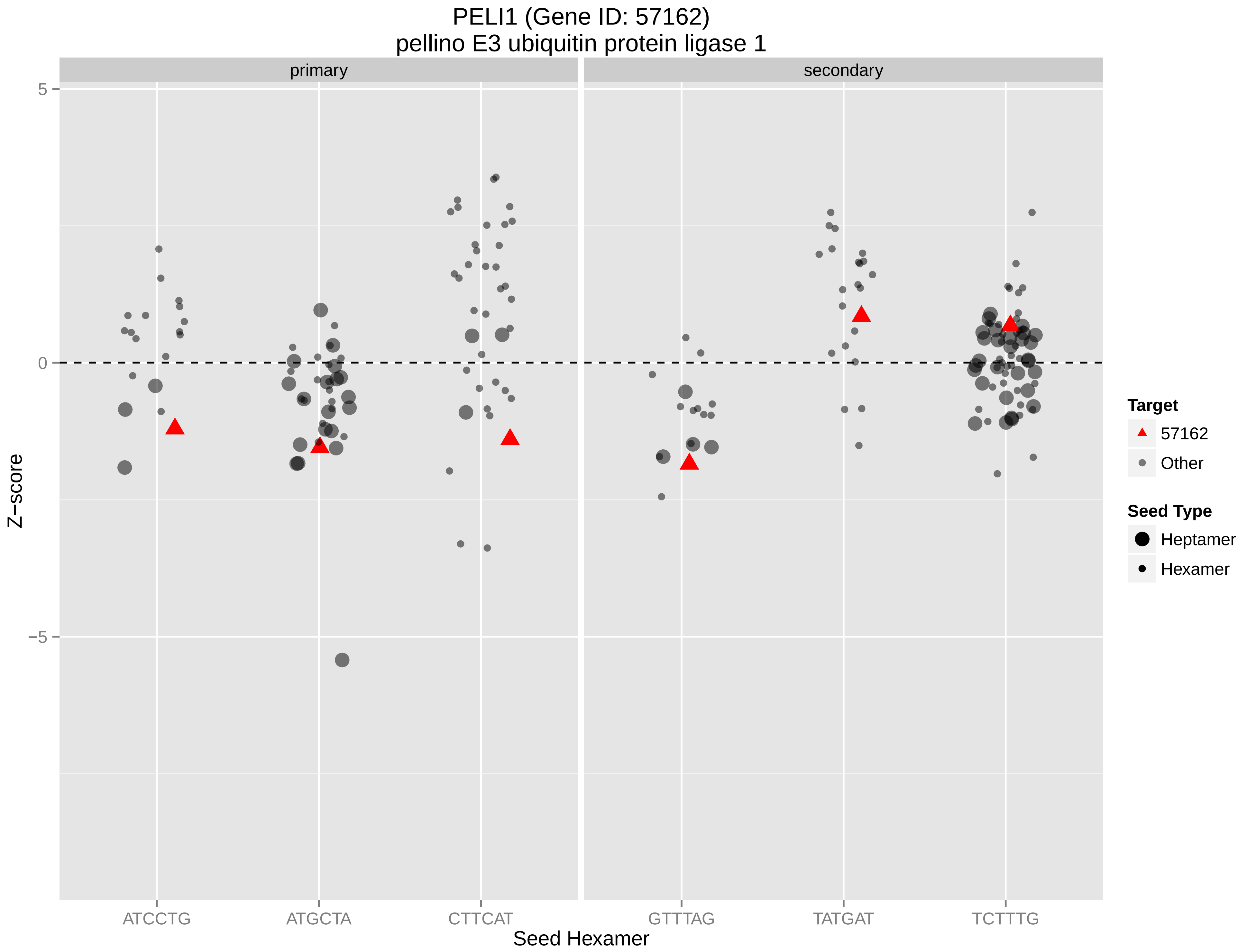
PELI1 (Gene ID: 57162)
pellino E3 ubiquitin protein ligase 1
primary
secondary
5
0
−5
Z−score
ATCCTG
ATGCTA
CTTCAT
GTTTAG
TATGAT
TCTTTG
Seed Hexamer
Target
57162
Other
Seed Type
Heptamer
Hexamer

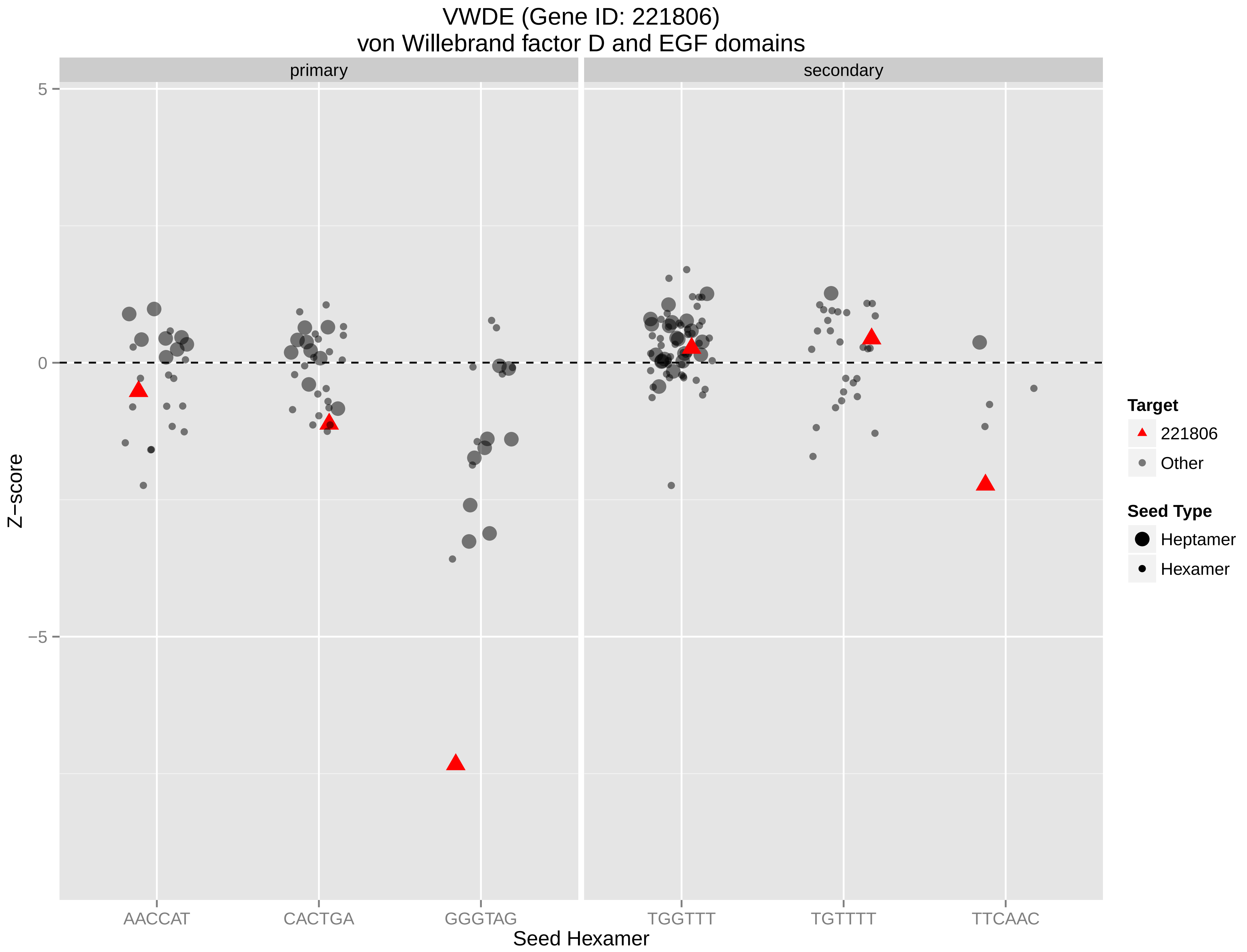

VWDE (Gene ID: 221806)
von Willebrand factor D and EGF domains
primary
secondary
5
0
−5
Z−score
AACCAT
CACTGA
GGGTAG
TGGTTT
TGTTTT
TTCAAC
Seed Hexamer
Target
221806
Other
Seed Type
Heptamer
Hexamer

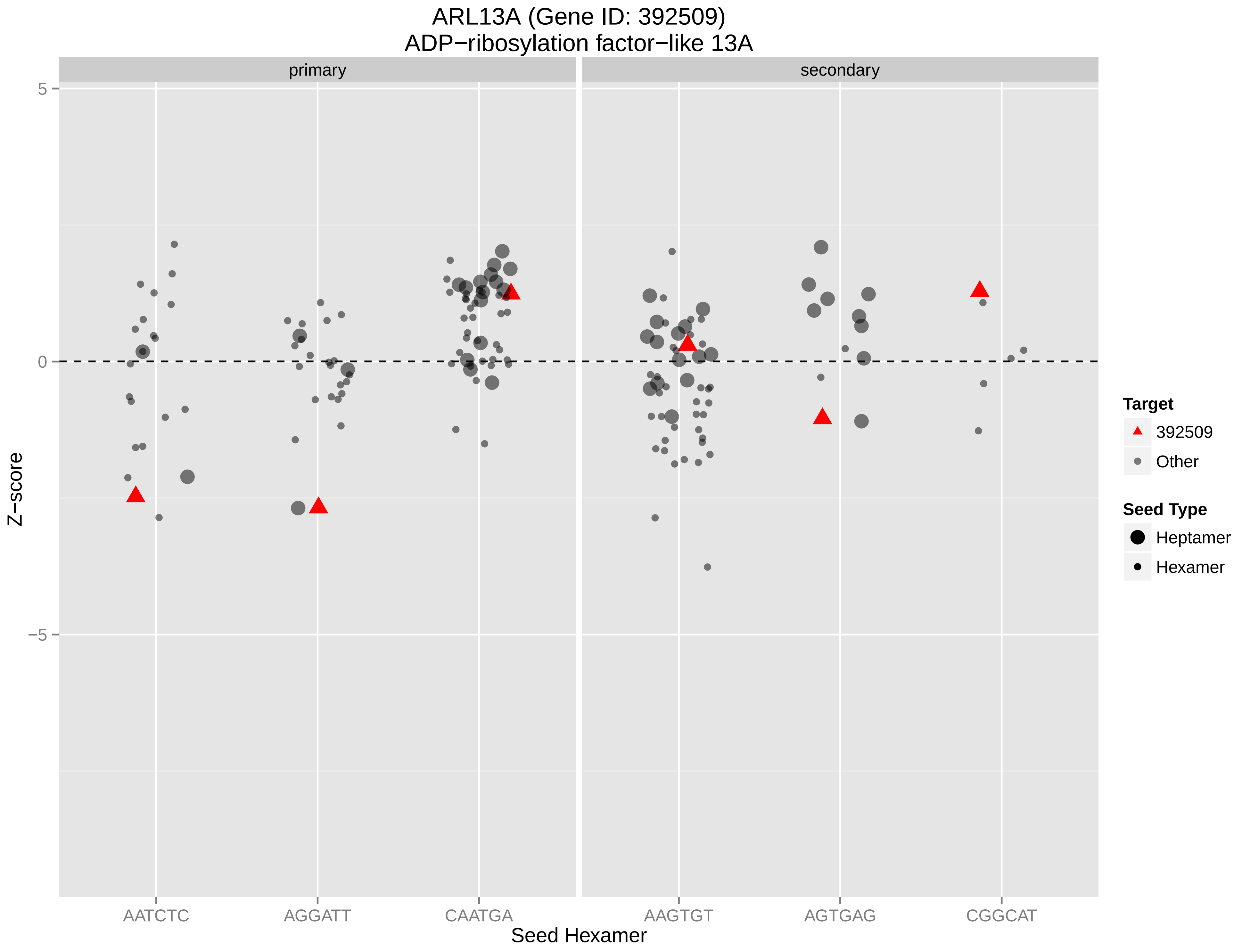

ARL13A (Gene ID: 392509)
ADP−ribosylation factor−like 13A
primary
secondary
5
0
−5
Z−score
AATCTC
AGGATT
CAATGA
AAGTGT
AGTGAG
CGGCAT
Seed Hexamer
Target
392509
Other
Seed Type
Heptamer
Hexamer

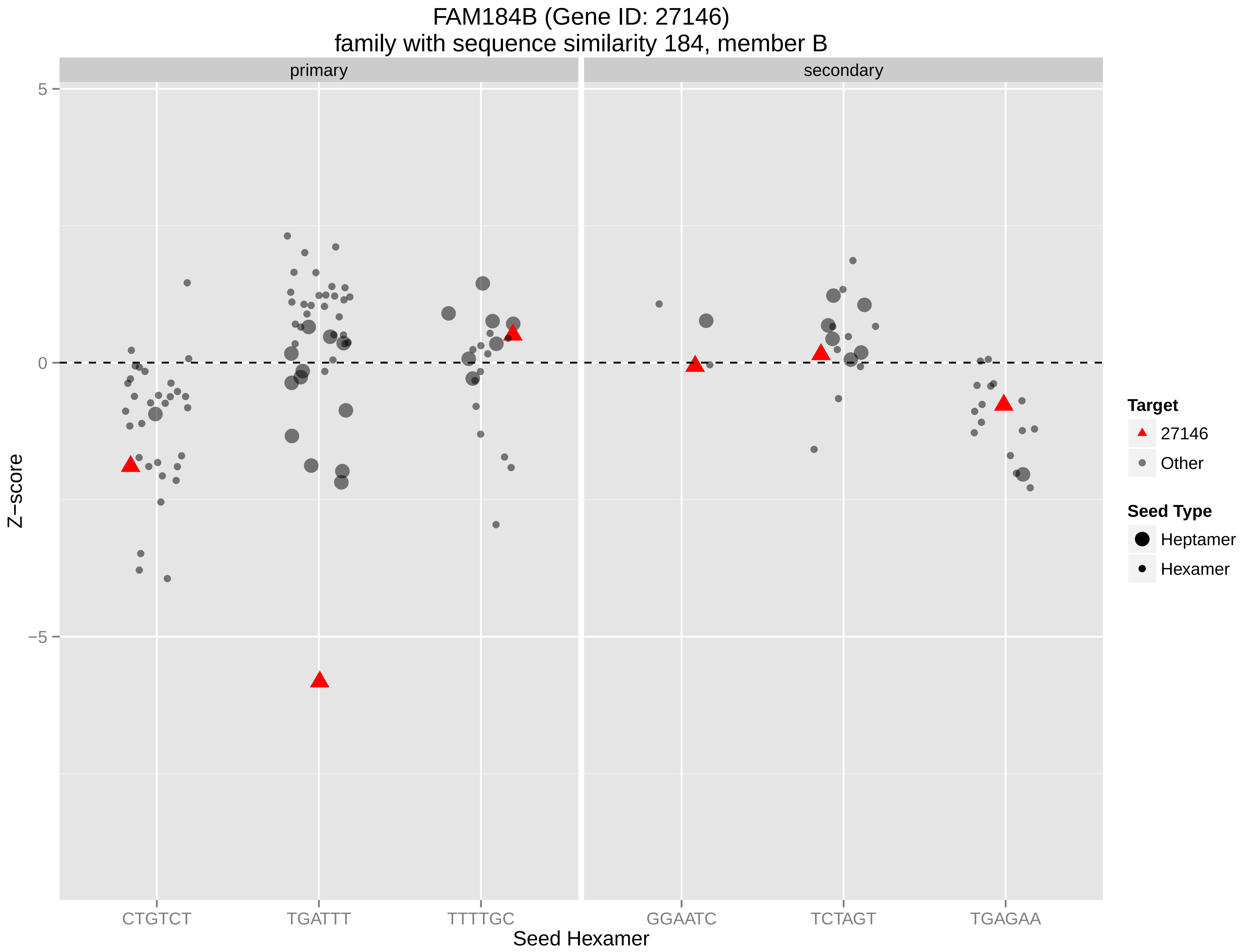

FAM184B (Gene ID: 27146)
family with sequence similarity 184, member B
primary
secondary
5
0
−5
Z−score
CTGTCT
TGATTT
TTTTGC
GGAATC
TCTAGT
TGAGAA
Seed Hexamer
Target
27146
Other
Seed Type
Heptamer
Hexamer

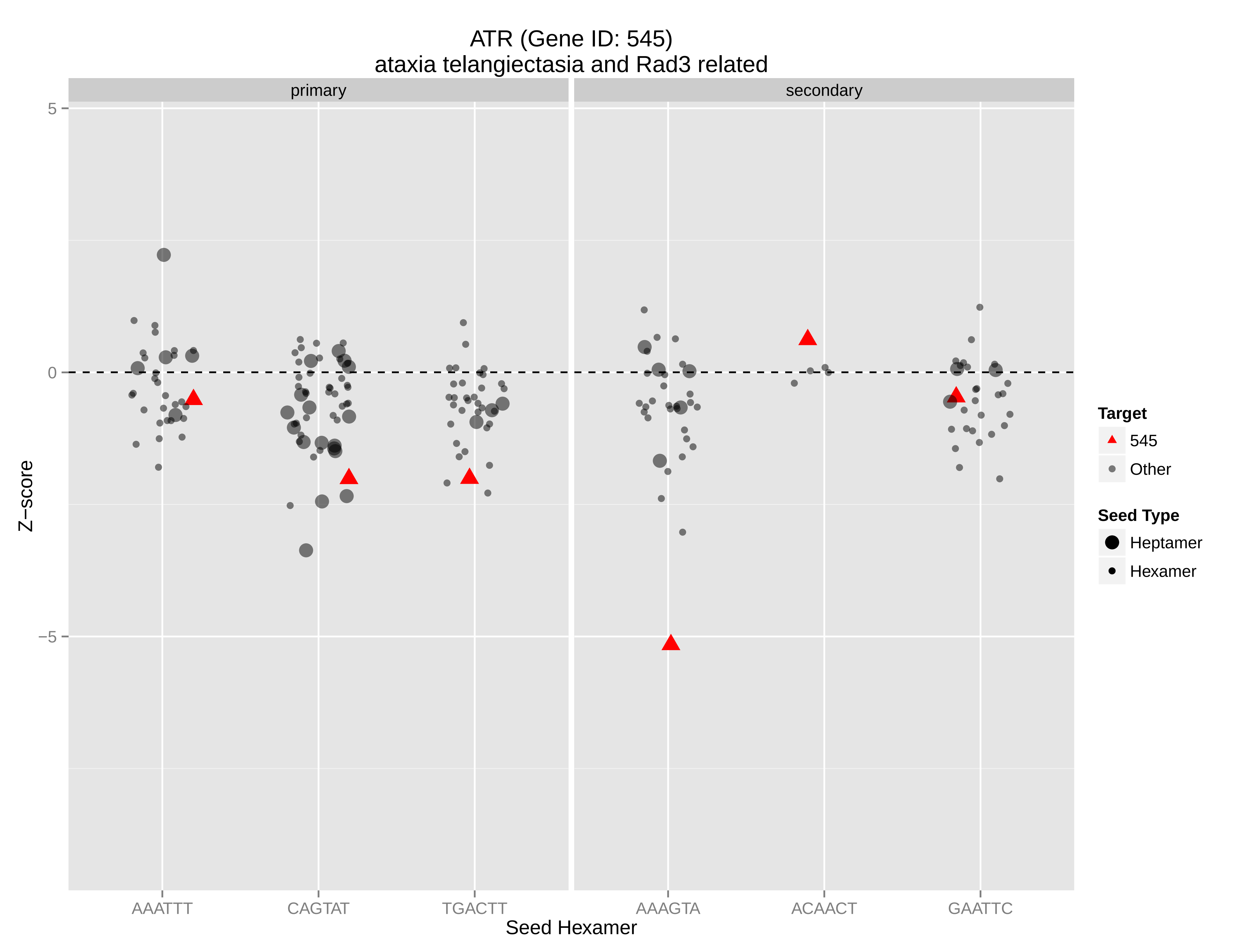

ATR (Gene ID: 545)
ataxia telangiectasia and Rad3 related
primary
secondary
5
0
−5
Z−score
AAATTT
CAGTAT
TGACTT
AAAGTA
ACAACT
GAATTC
Seed Hexamer
Target
545
Other
Seed Type
Heptamer
Hexamer

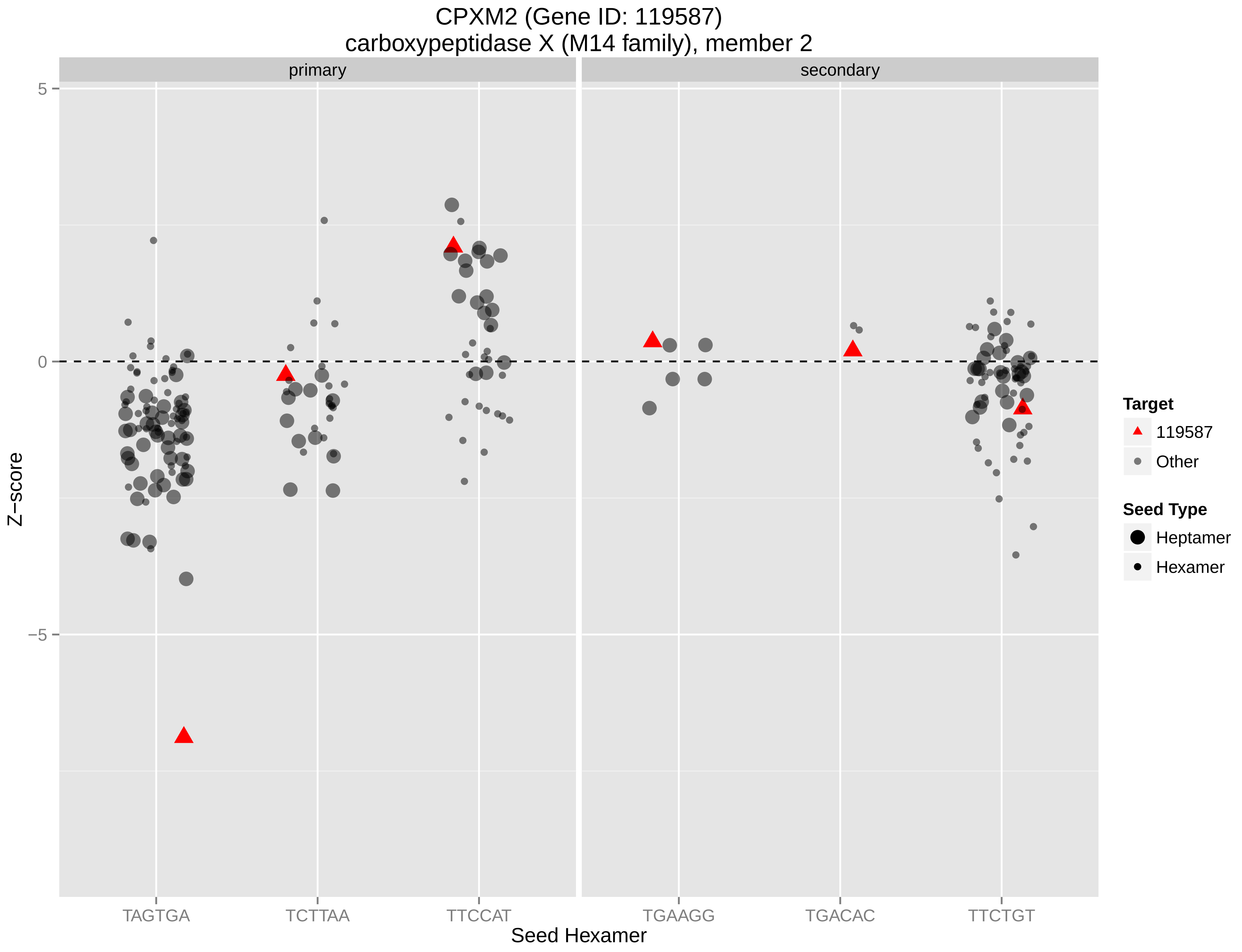
CPXM2 (Gene ID: 119587)
carboxypeptidase X (M14 family), member 2
primary
secondary
5
0
−5
Z−score
TAGTGA
TCTTAA
TTCCAT
TGAAGG
TGACAC
TTCTGT
Seed Hexamer
Target
119587
Other
Seed Type
Heptamer
Hexamer

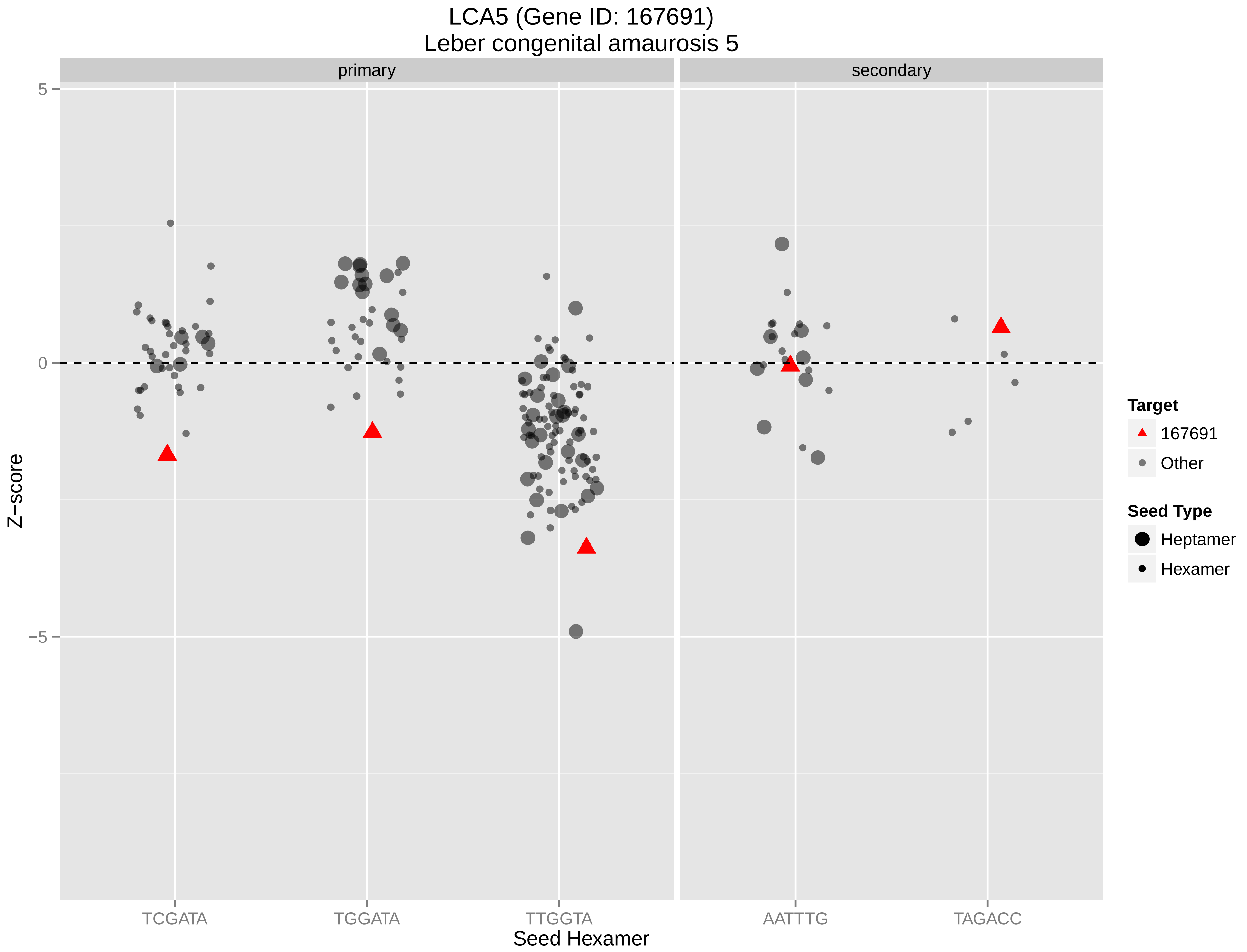
LCA5 (Gene ID: 167691)
Leber congenital amaurosis 5
primary
secondary
5
0
−5
Z−score
TCGATA
TGGATA
TTGGTA
AATTTG
TAGACC
Seed Hexamer
Target
167691
Other
Seed Type
Heptamer
Hexamer

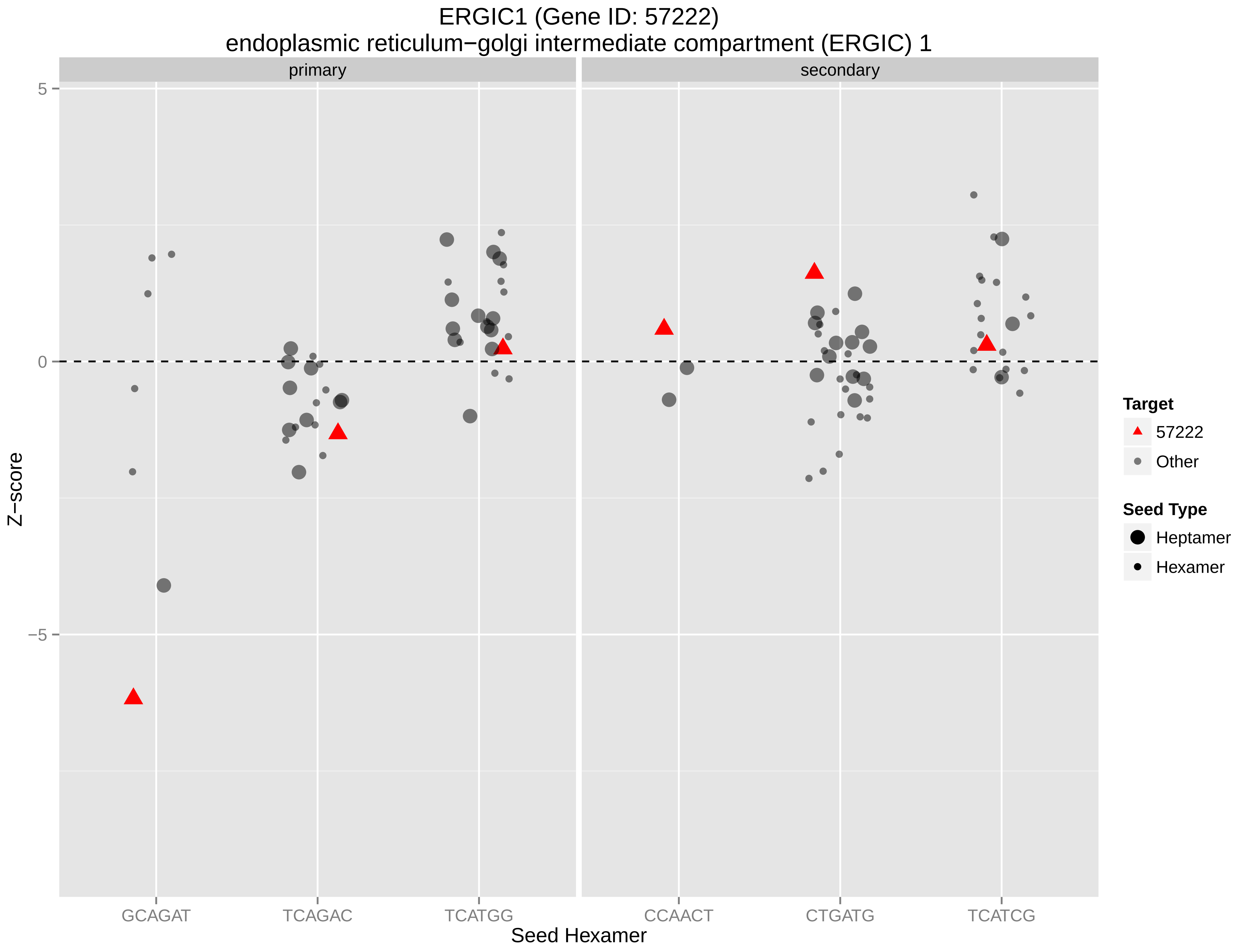
ERGIC1 (Gene ID: 57222)
endoplasmic reticulum−golgi intermediate compartment (ERGIC) 1
primary
secondary
5
0
−5
Z−score
GCAGAT
TCAGAC
TCATGG
CCAACT
CTGATG
TCATCG
Seed Hexamer
Target
57222
Other
Seed Type
Heptamer
Hexamer

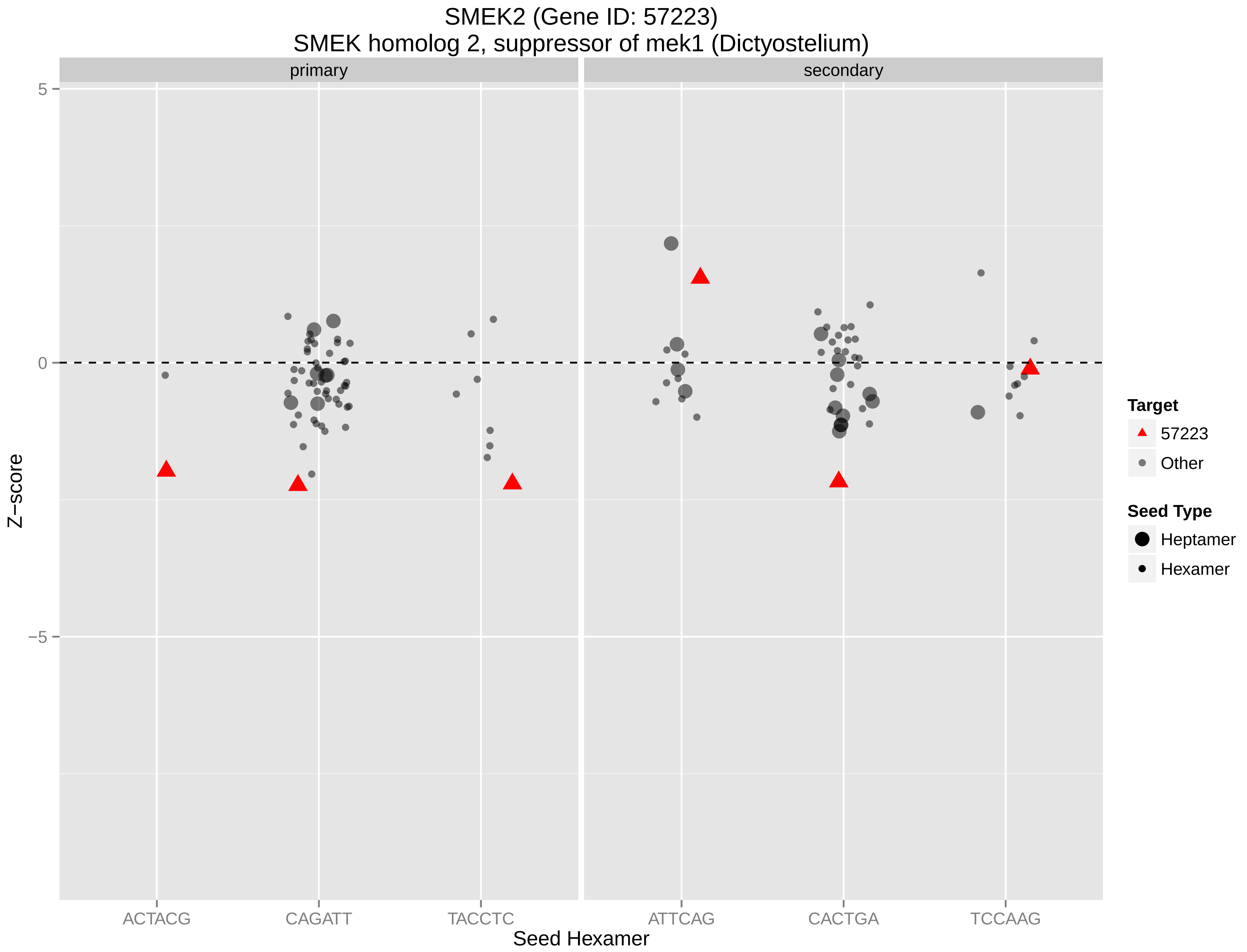
SMEK2 (Gene ID: 57223)
SMEK homolog 2, suppressor of mek1 (Dictyostelium)
primary
secondary
5
0
−5
Z−score
ACTACG
CAGATT
TACCTC
ATTCAG
CACTGA
TCCAAG
Seed Hexamer
Target
57223
Other
Seed Type
Heptamer
Hexamer

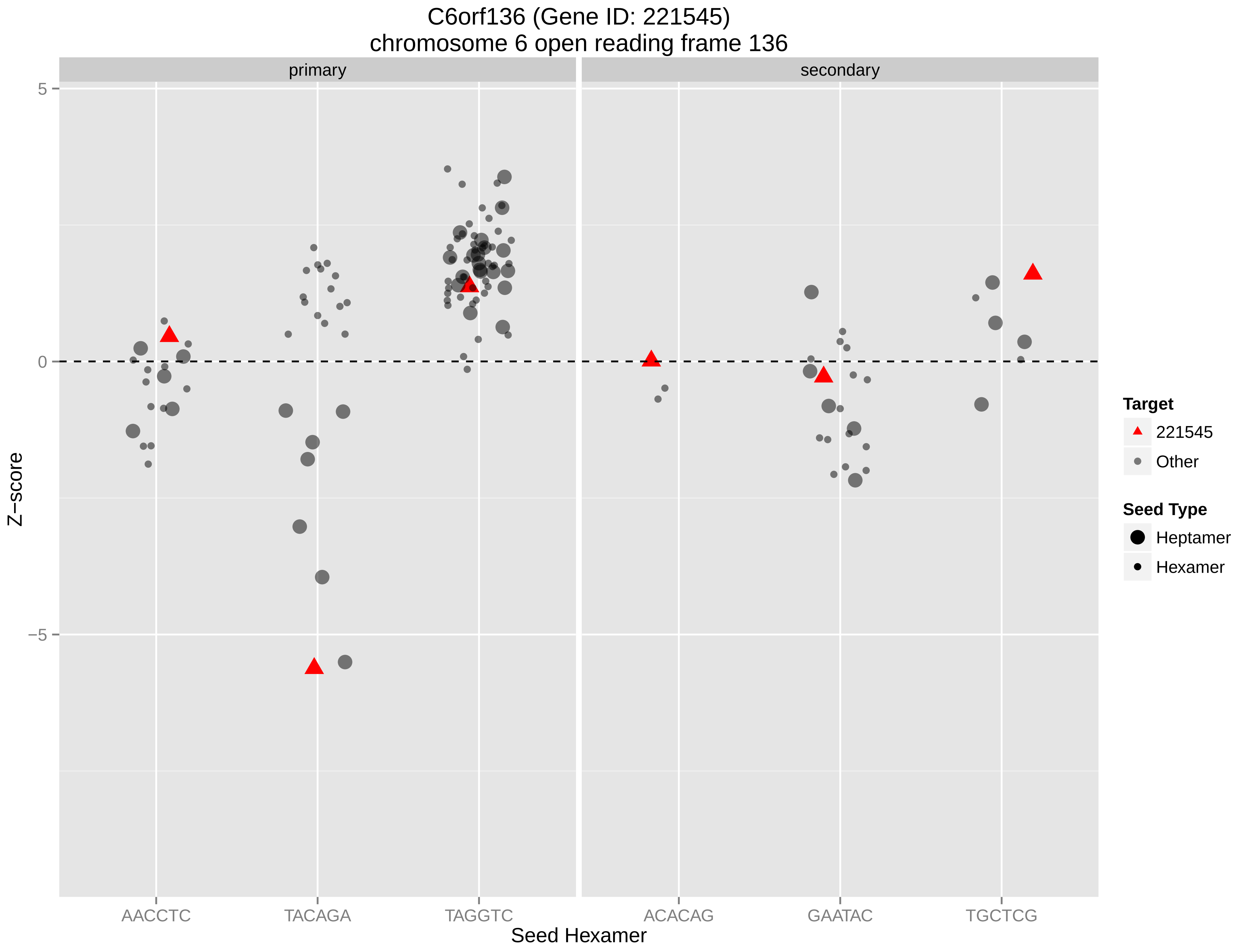

C6orf136 (Gene ID: 221545)
chromosome 6 open reading frame 136
primary
secondary
5
0
−5
Z−score
AACCTC
TACAGA
TAGGTC
ACACAG
GAATAC
TGCTCG
Seed Hexamer
Target
221545
Other
Seed Type
Heptamer
Hexamer

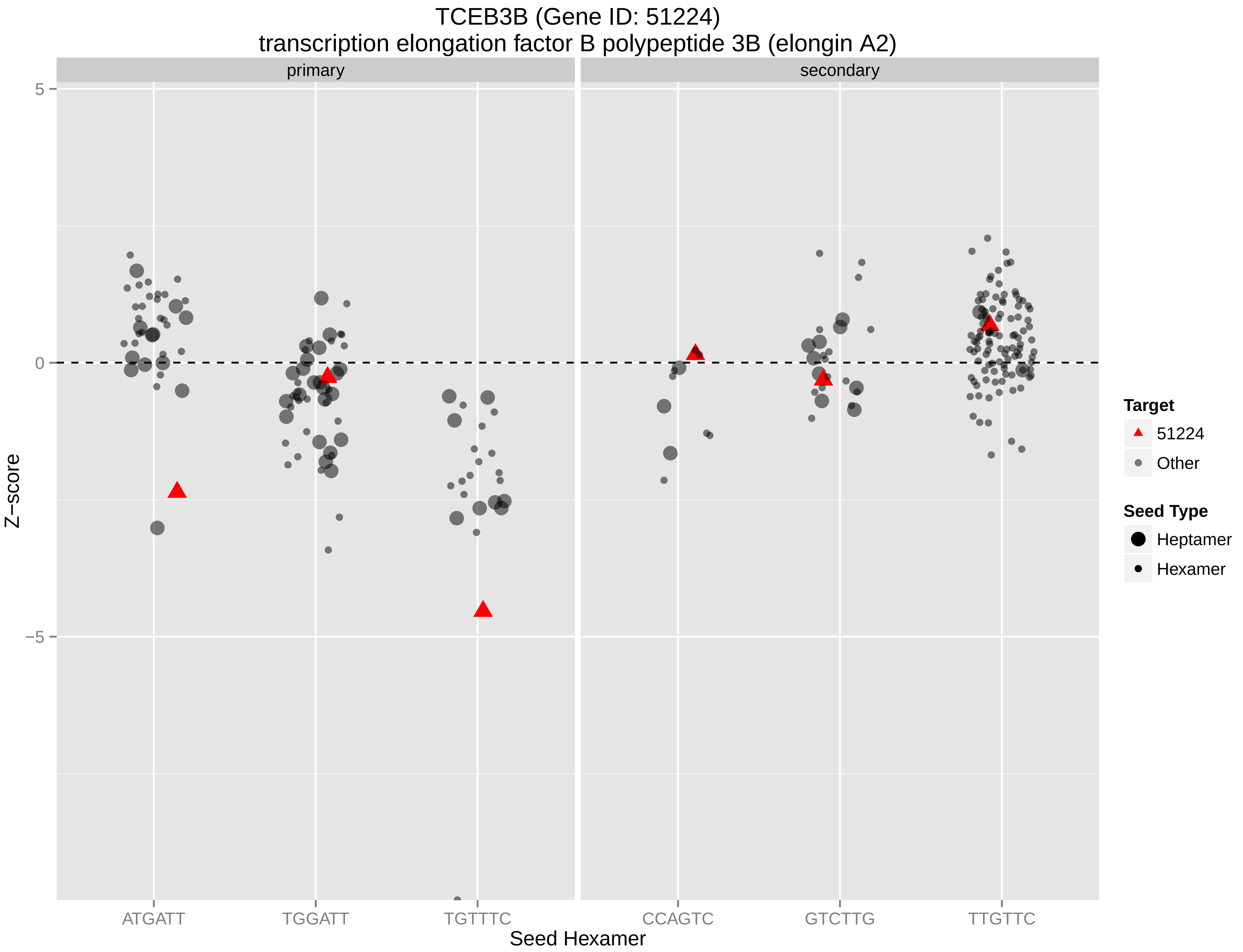
TCEB3B (Gene ID: 51224)
transcription elongation factor B polypeptide 3B (elongin A2)
primary
secondary
5
0
−5
Z−score
ATGATT
TGGATT
TGTTTC
CCAGTC
GTCTTG
TTGTTC
Seed Hexamer
Target
51224
Other
Seed Type
Heptamer
Hexamer

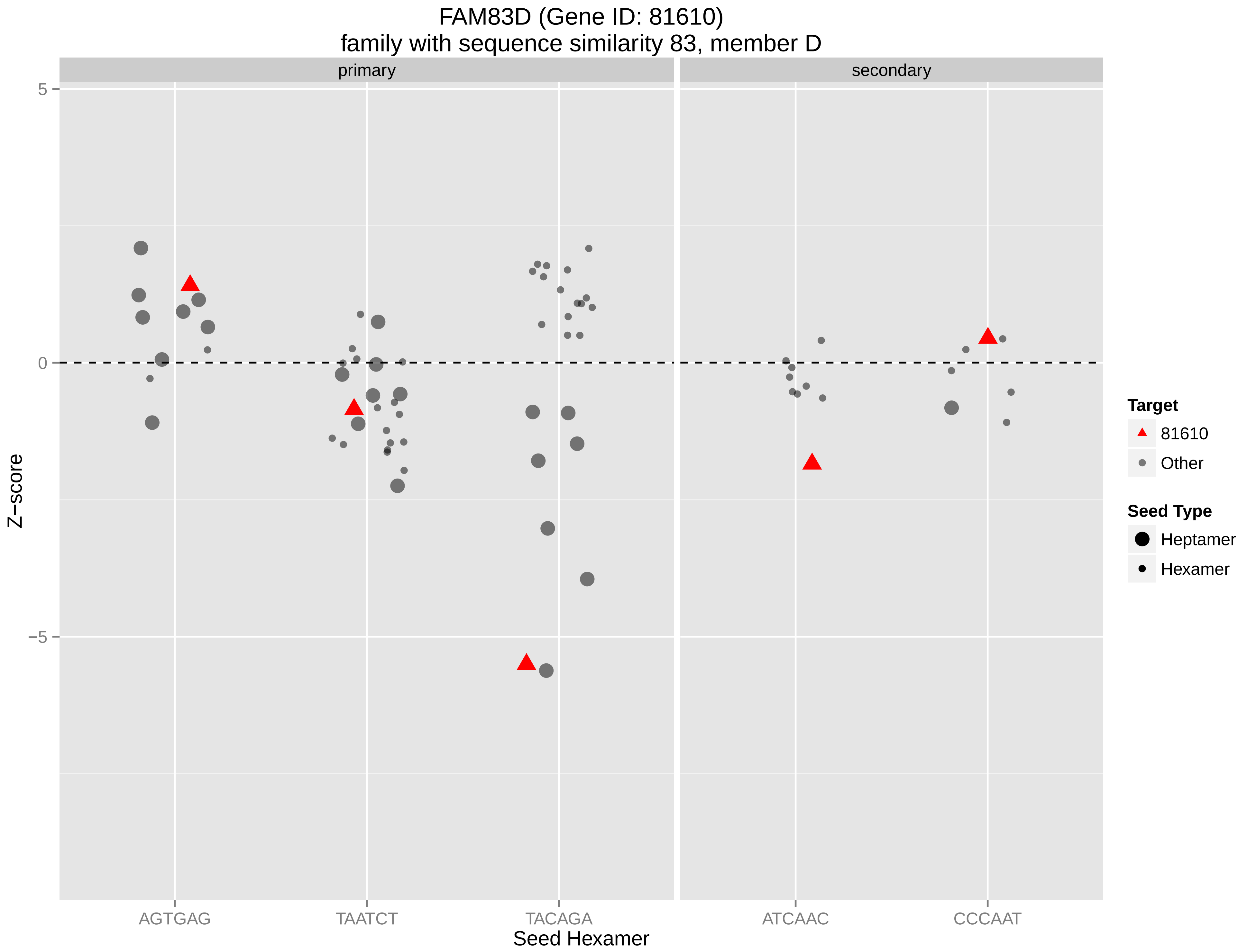
FAM83D (Gene ID: 81610)
family with sequence similarity 83, member D
primary
secondary
5
0
−5
Z−score
AGTGAG
TAATCT
TACAGA
ATCAAC
CCCAAT
Seed Hexamer
Target
81610
Other
Seed Type
Heptamer
Hexamer

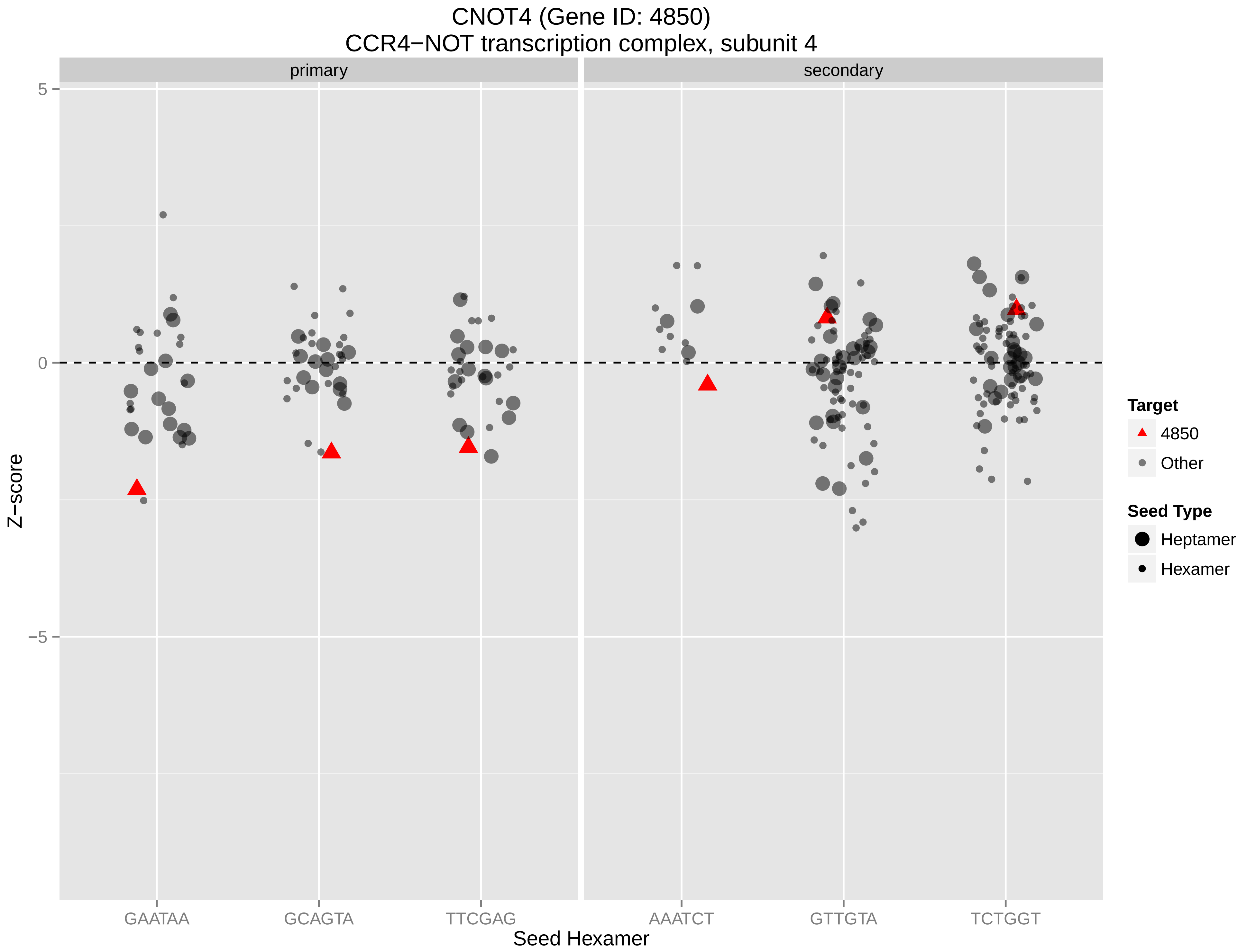
CNOT4 (Gene ID: 4850)
CCR4−NOT transcription complex, subunit 4
primary
secondary
5
0
−5
Z−score
GAATAA
GCAGTA
TTCGAG
AAATCT
GTTGTA
TCTGGT
Seed Hexamer
Target
4850
Other
Seed Type
Heptamer
Hexamer

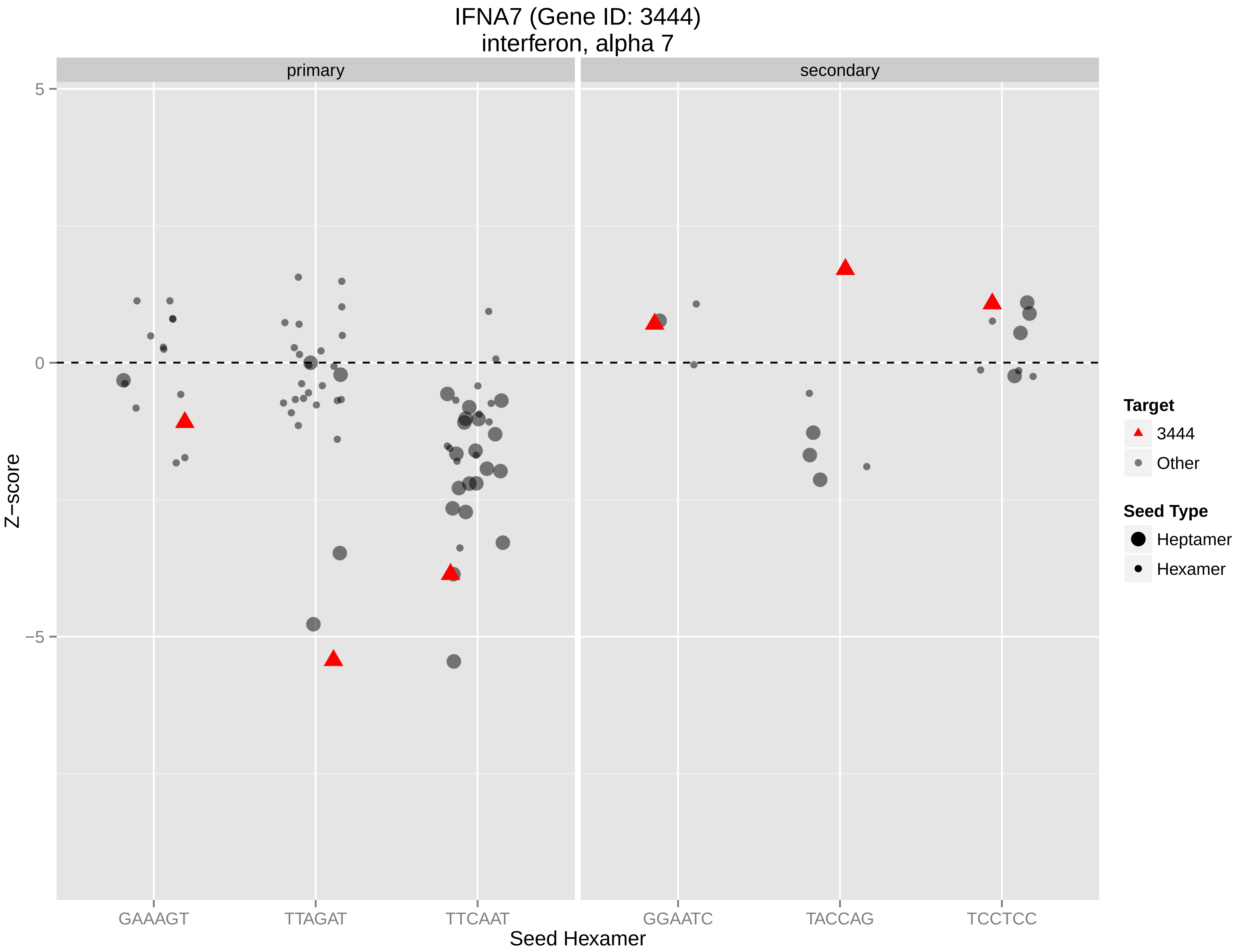
IFNA7 (Gene ID: 3444)
interferon, alpha 7
primary
secondary
5
0
−5
Z−score
GAAAGT
TTAGAT
TTCAAT
GGAATC
TACCAG
TCCTCC
Seed Hexamer
Target
3444
Other
Seed Type
Heptamer
Hexamer

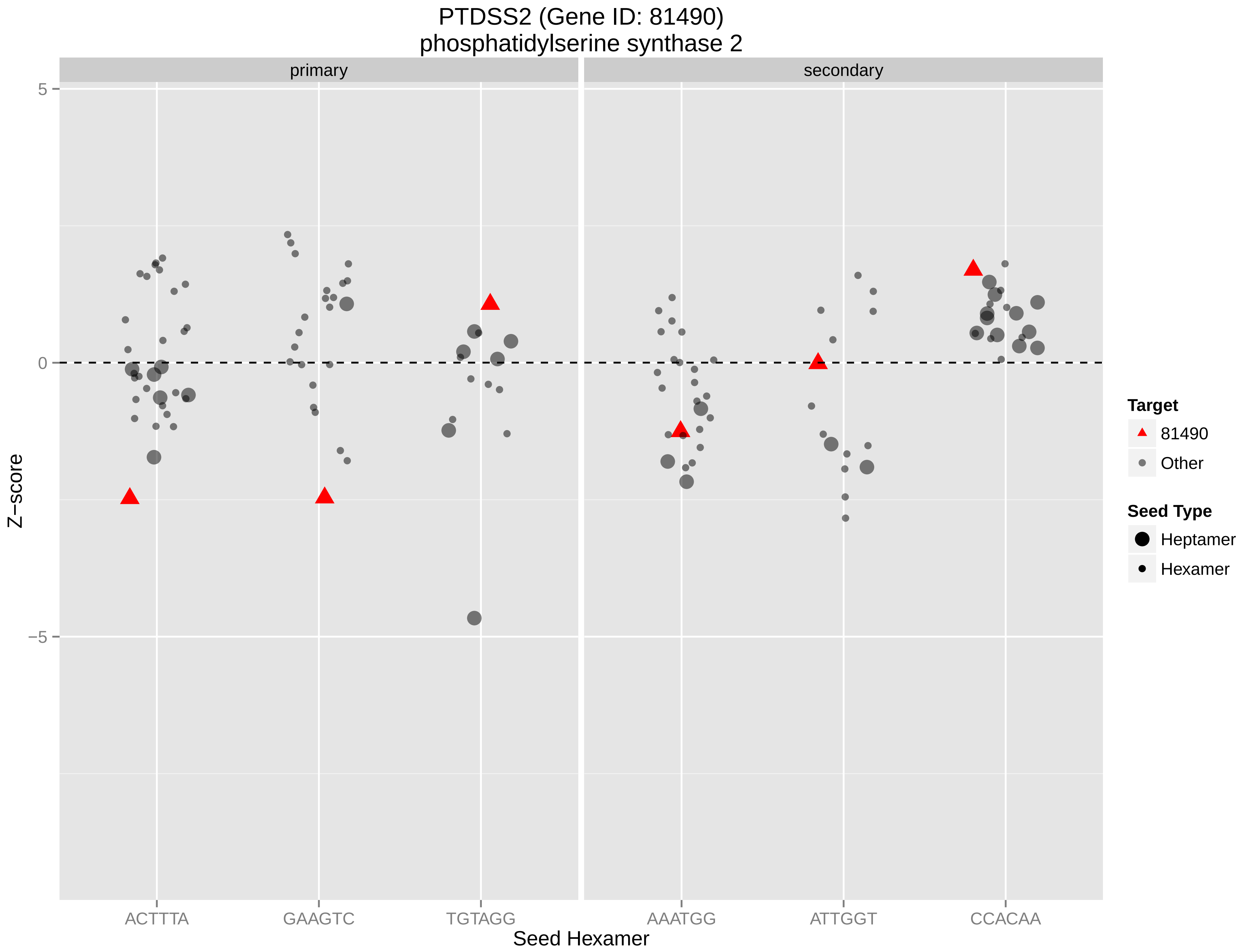

PTDSS2 (Gene ID: 81490)
phosphatidylserine synthase 2
primary
secondary
5
0
−5
Z−score
ACTTTA
GAAGTC
TGTAGG
AAATGG
ATTGGT
CCACAA
Seed Hexamer
Target
81490
Other
Seed Type
Heptamer
Hexamer

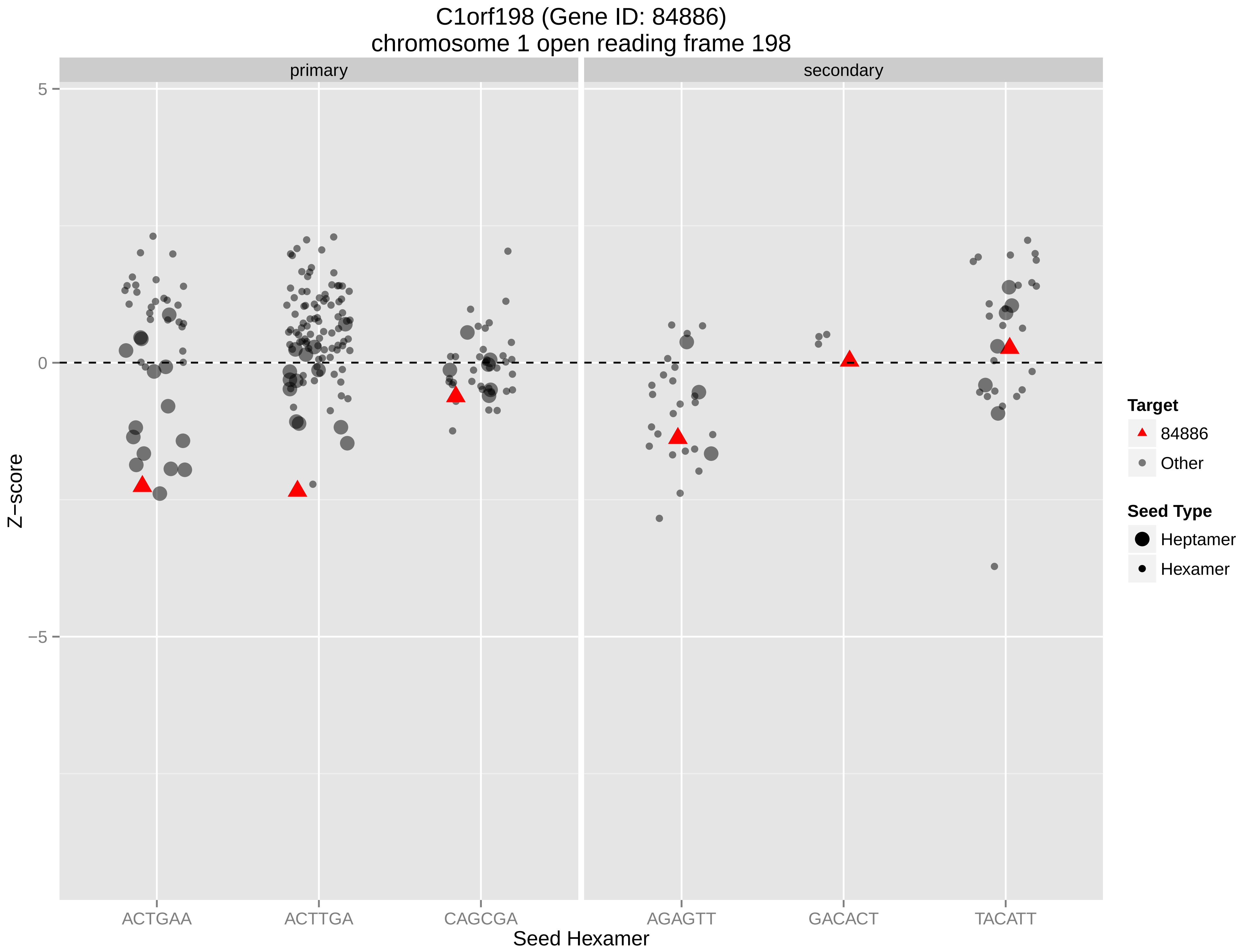

C1orf198 (Gene ID: 84886)
chromosome 1 open reading frame 198
primary
secondary
5
0
−5
Z−score
ACTGAA
ACTTGA
CAGCGA
AGAGTT
GACACT
TACATT
Seed Hexamer
Target
84886
Other
Seed Type
Heptamer
Hexamer

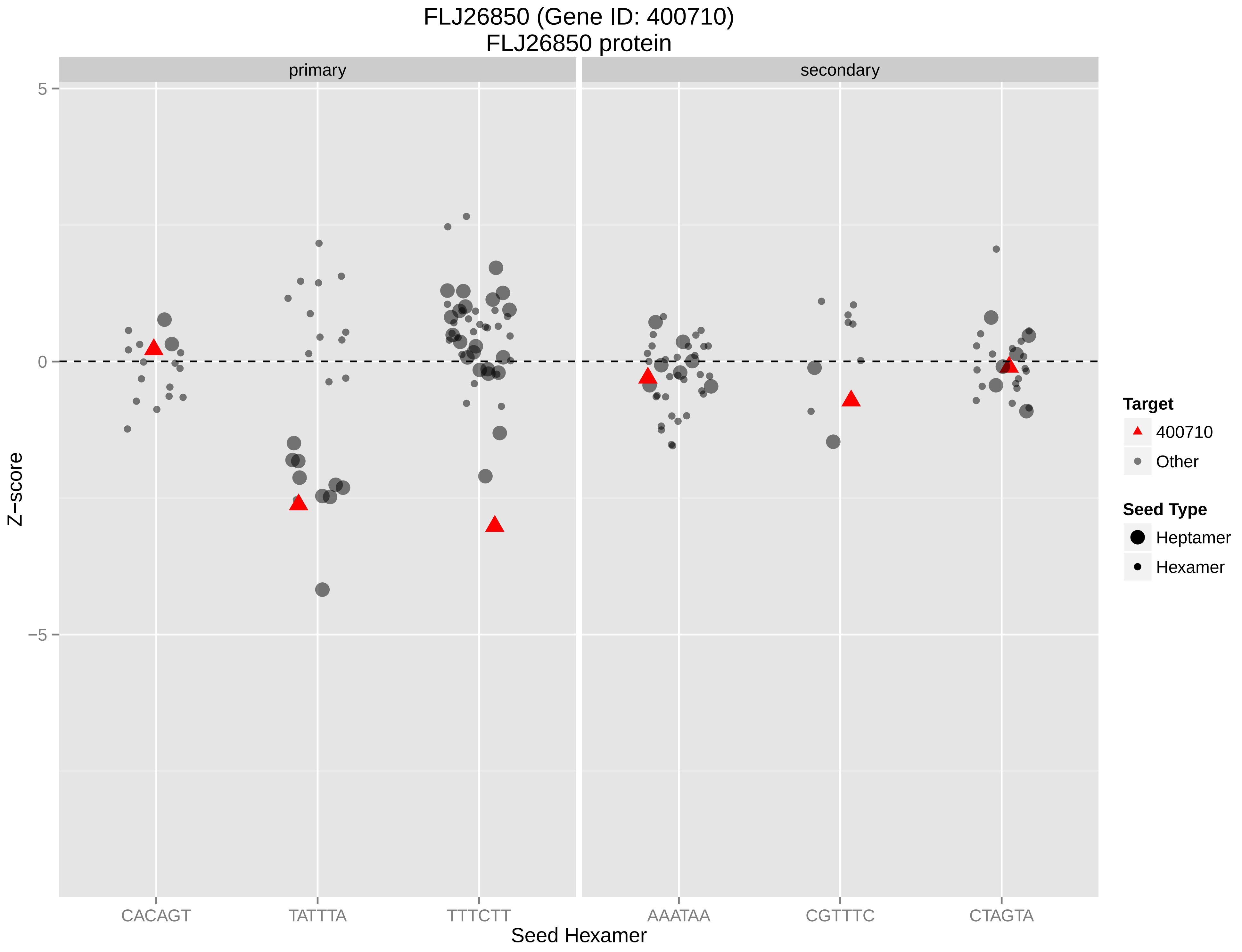
FLJ26850 (Gene ID: 400710)
FLJ26850 protein
primary
secondary
5
0
−5
Z−score
CACAGT
TATTTA
TTTCTT
AAATAA
CGTTTC
CTAGTA
Seed Hexamer
Target
400710
Other
Seed Type
Heptamer
Hexamer

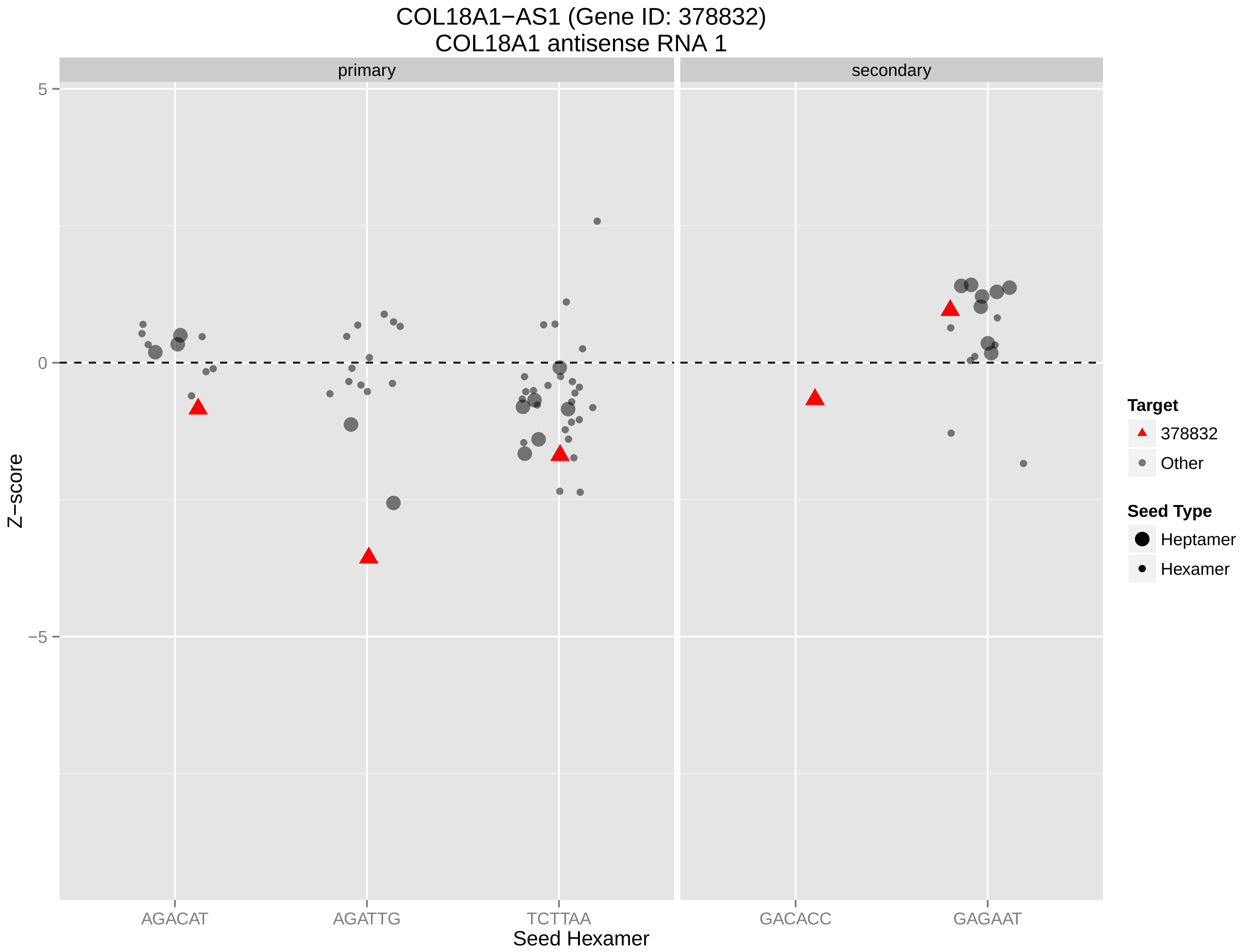
COL18A1−AS1 (Gene ID: 378832)
COL18A1 antisense RNA 1
primary
secondary
5
0
−5
Z−score
AGACAT
AGATTG
TCTTAA
GACACC
GAGAAT
Seed Hexamer
Target
378832
Other
Seed Type
Heptamer
Hexamer

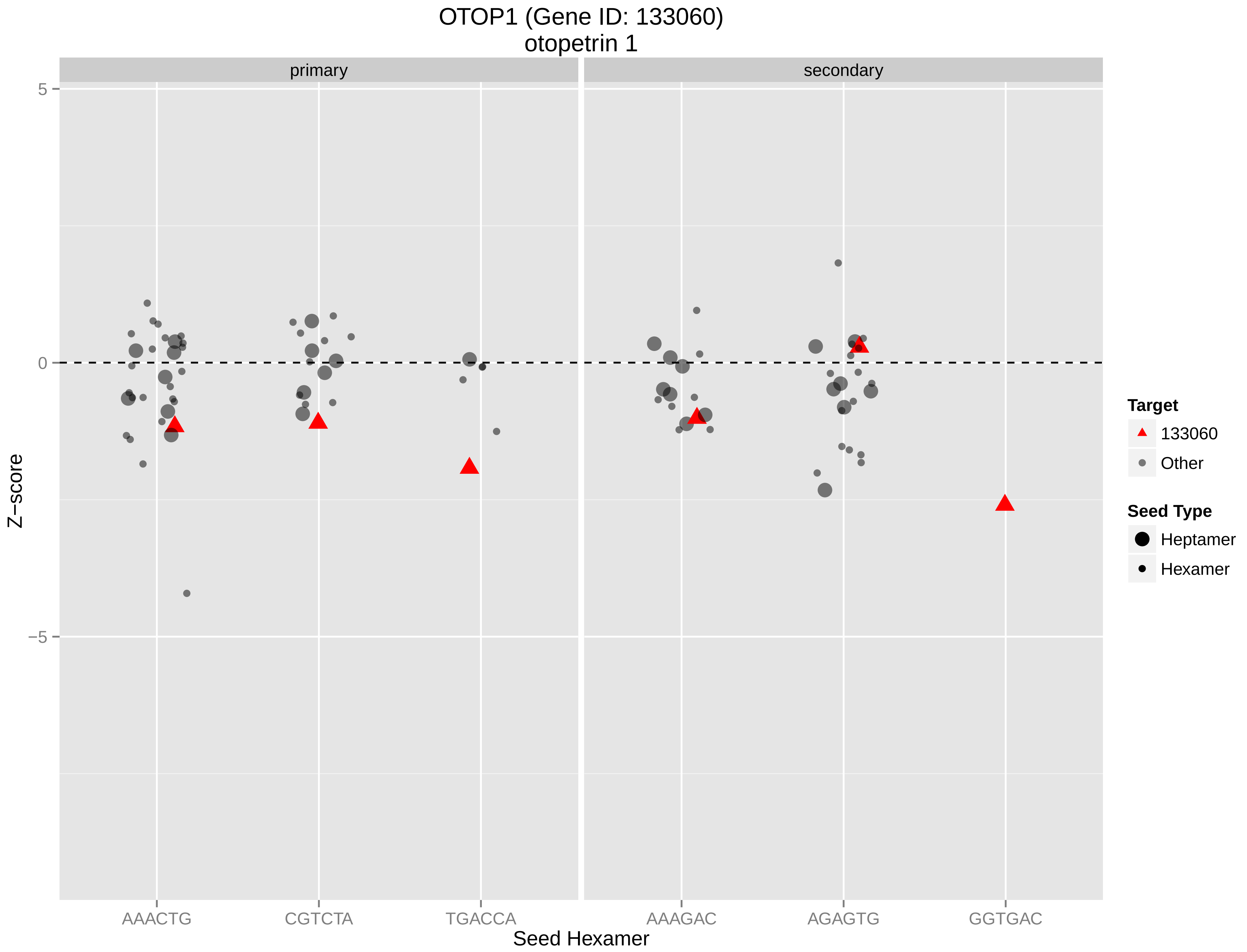

OTOP1 (Gene ID: 133060)
otopetrin 1
primary
secondary
5
0
−5
Z−score
AAACTG
CGTCTA
TGACCA
AAAGAC
AGAGTG
GGTGAC
Seed Hexamer
Target
133060
Other
Seed Type
Heptamer
Hexamer

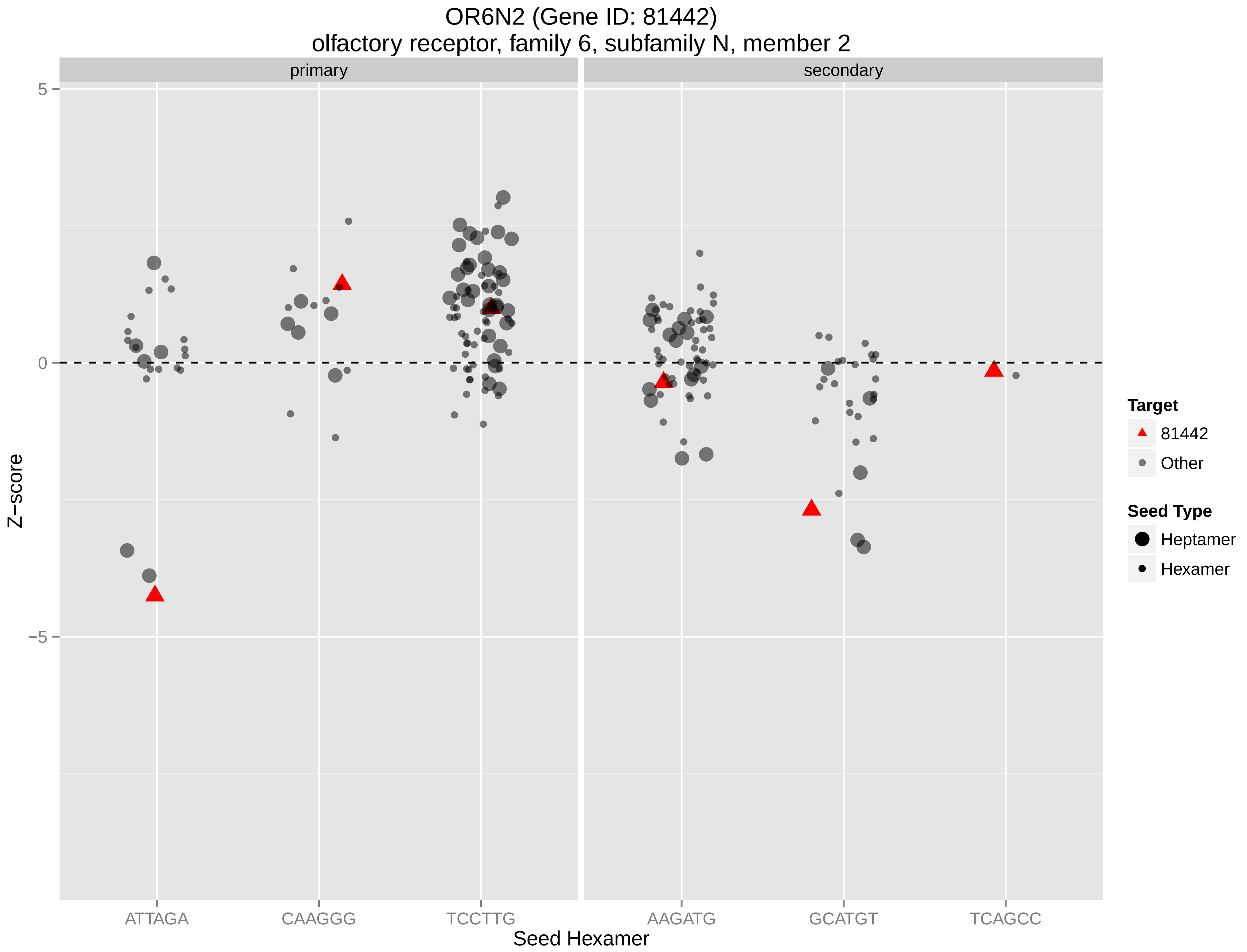
OR6N2 (Gene ID: 81442)
olfactory receptor, family 6, subfamily N, member 2
primary
secondary
5
0
−5
Z−score
ATTAGA
CAAGGG
TCCTTG
AAGATG
GCATGT
TCAGCC
Seed Hexamer
Target
81442
Other
Seed Type
Heptamer
Hexamer

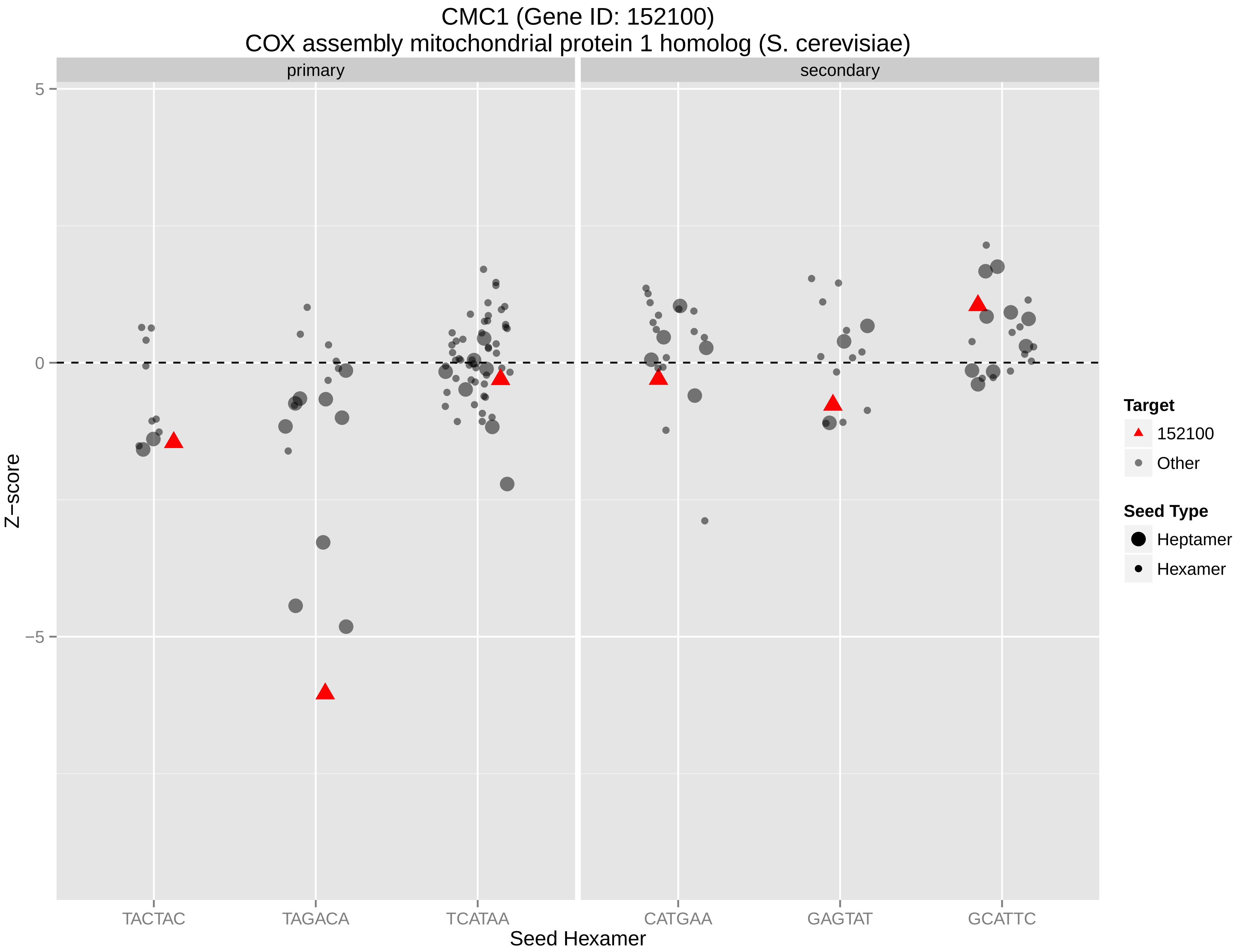
CMC1 (Gene ID: 152100)
COX assembly mitochondrial protein 1 homolog (S. cerevisiae)
primary
secondary
5
0
−5
Z−score
TACTAC
TAGACA
TCATAA
CATGAA
GAGTAT
GCATTC
Seed Hexamer
Target
152100
Other
Seed Type
Heptamer
Hexamer

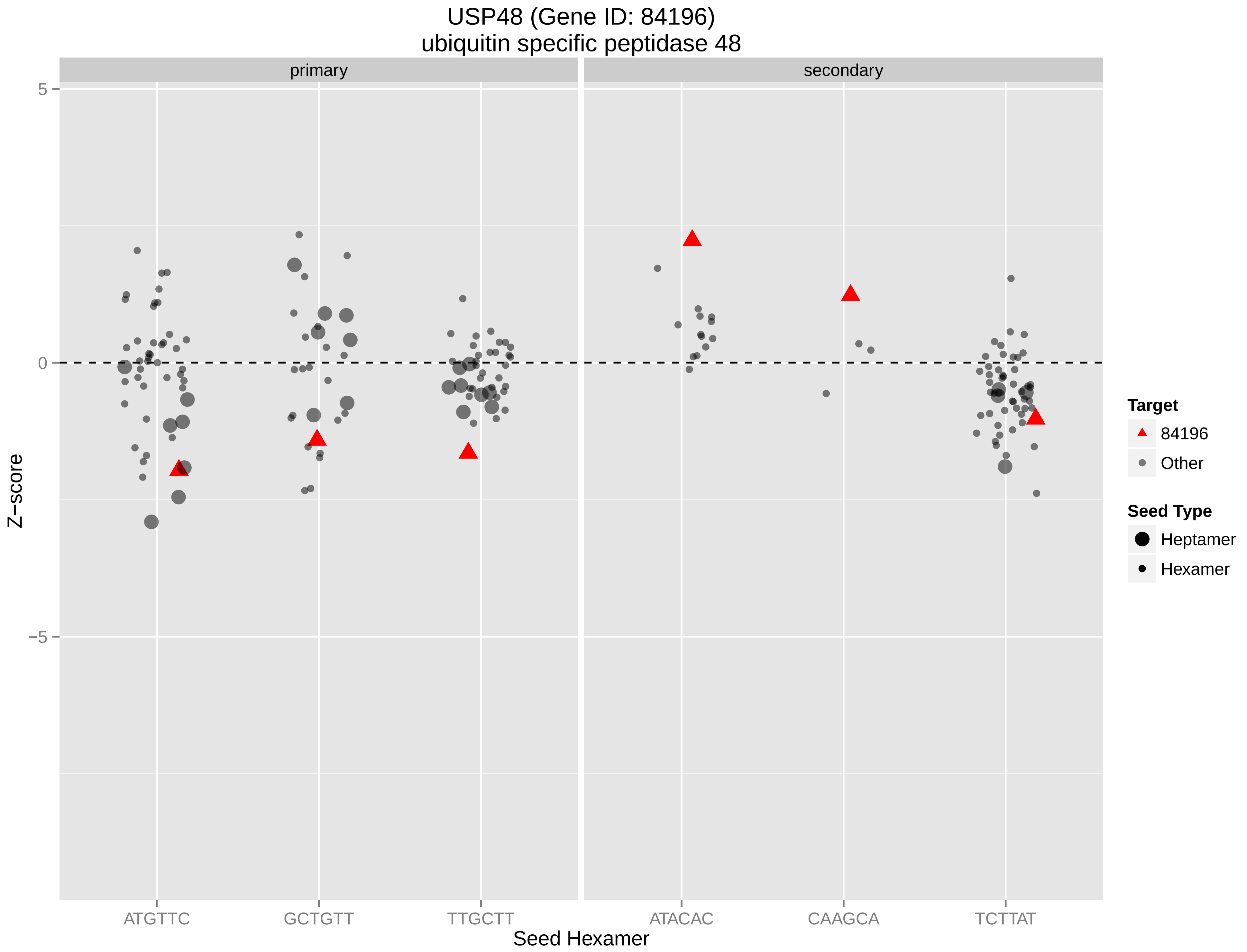

USP48 (Gene ID: 84196)
ubiquitin specific peptidase 48
primary
secondary
5
0
−5
Z−score
ATGTTC
GCTGTT
TTGCTT
ATACAC
CAAGCA
TCTTAT
Seed Hexamer
Target
84196
Other
Seed Type
Heptamer
Hexamer

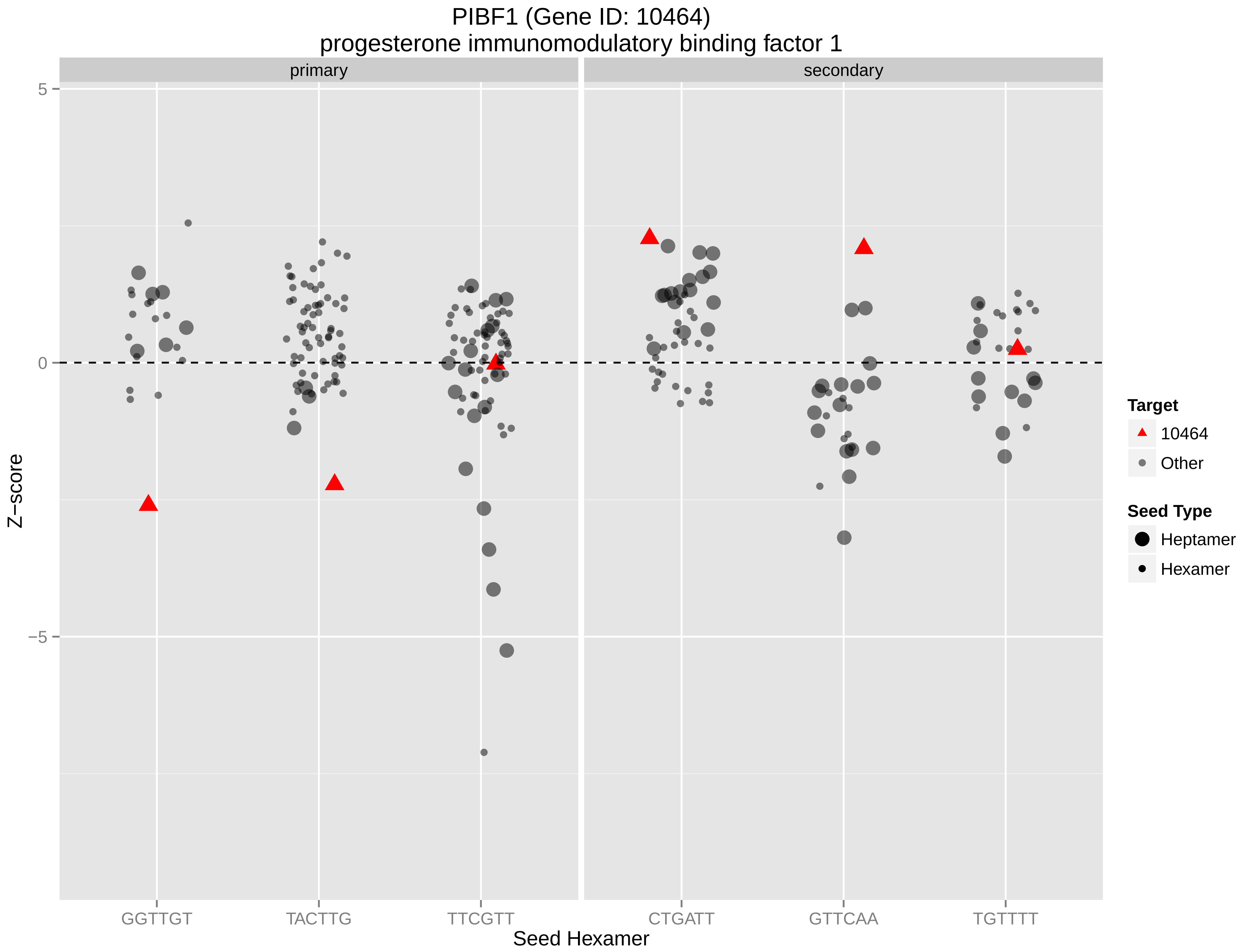
PIBF1 (Gene ID: 10464)
progesterone immunomodulatory binding factor 1
primary
secondary
5
0
−5
Z−score
GGTTGT
TACTTG
TTCGTT
CTGATT
GTTCAA
TGTTTT
Seed Hexamer
Target
10464
Other
Seed Type
Heptamer
Hexamer

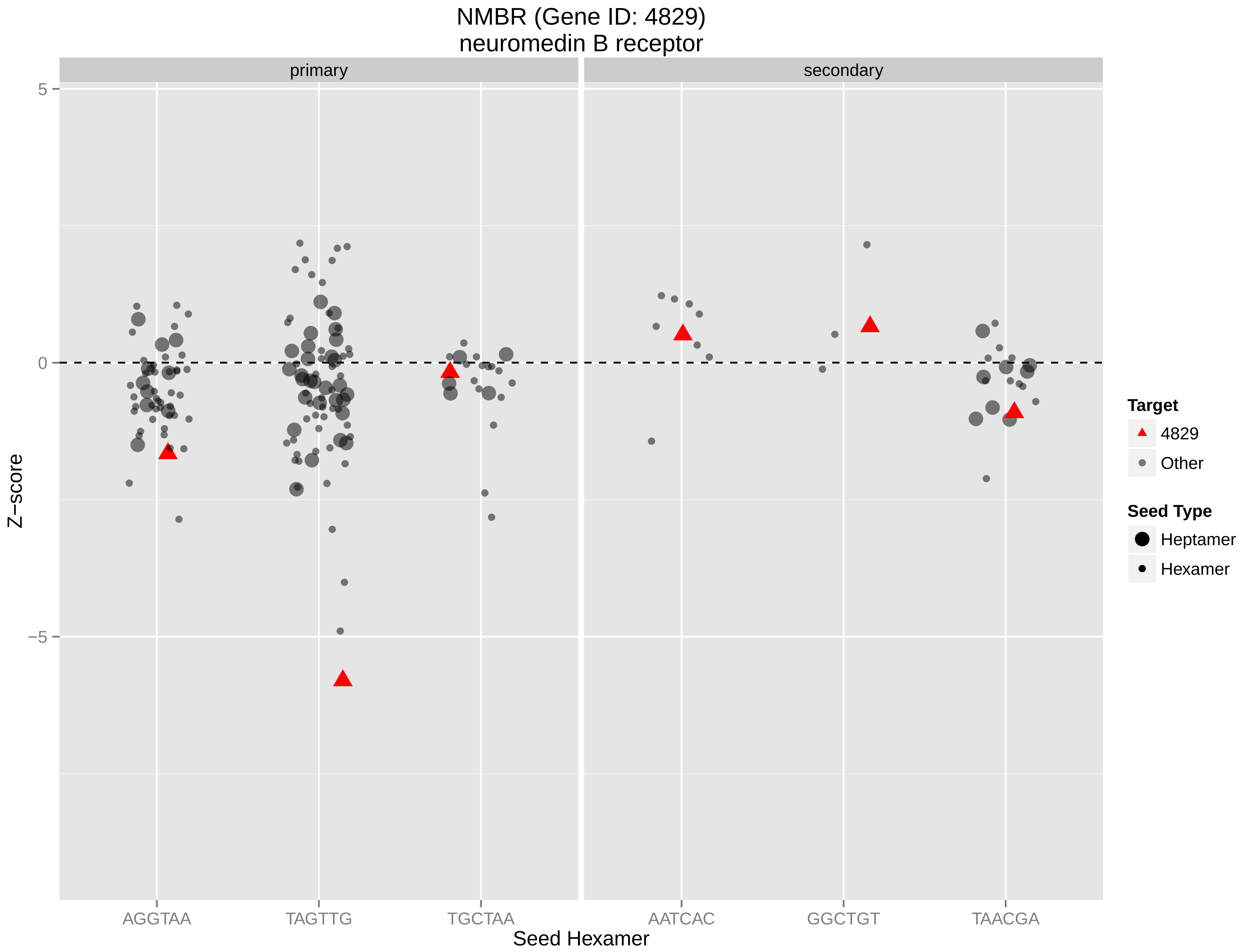
NMBR (Gene ID: 4829)
neuromedin B receptor
primary
secondary
5
0
−5
Z−score
AGGTAA
TAGTTG
TGCTAA
AATCAC
GGCTGT
TAACGA
Seed Hexamer
Target
4829
Other
Seed Type
Heptamer
Hexamer

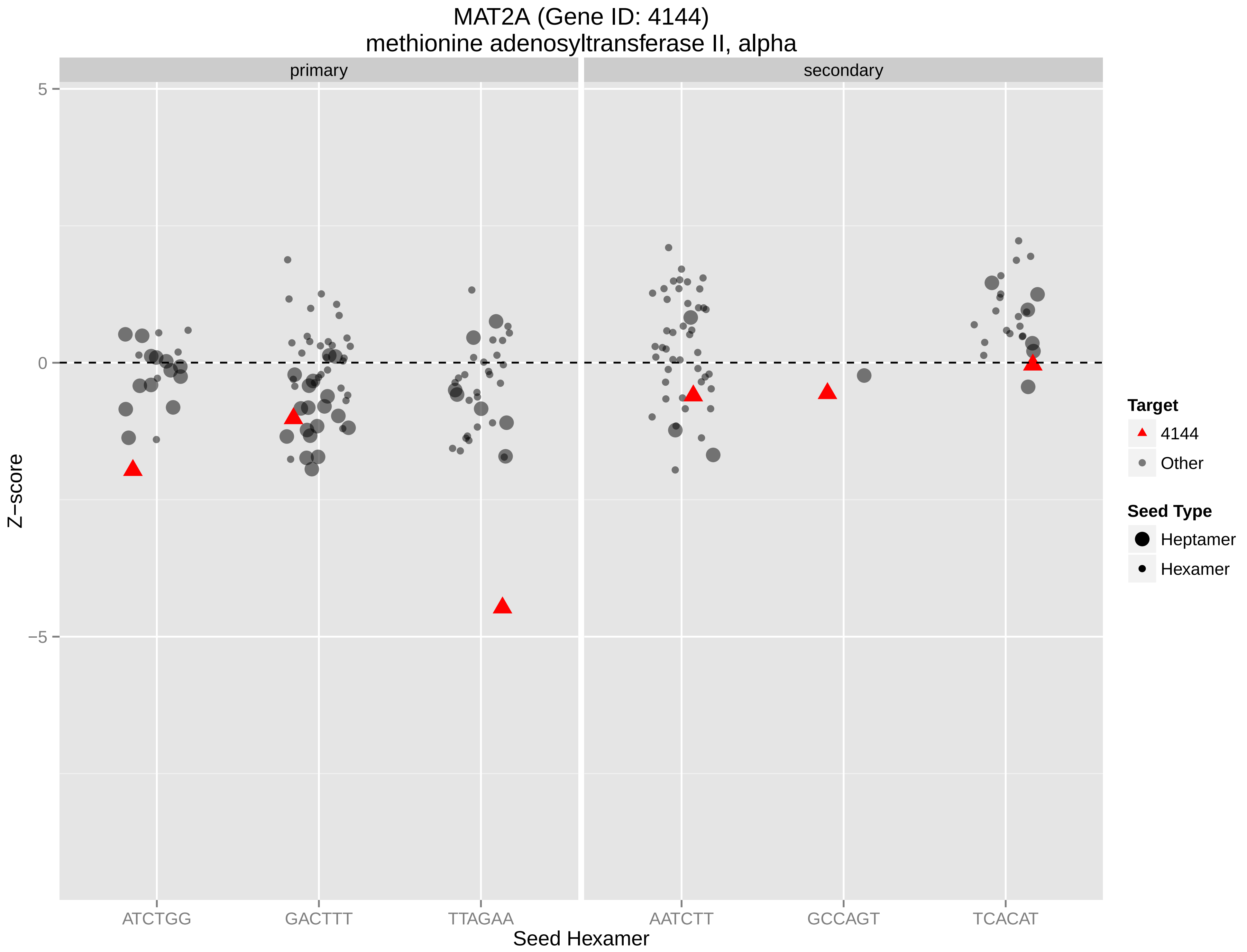

MAT2A (Gene ID: 4144)
methionine adenosyltransferase II, alpha
primary
secondary
5
0
−5
Z−score
ATCTGG
GACTTT
TTAGAA
AATCTT
GCCAGT
TCACAT
Seed Hexamer
Target
4144
Other
Seed Type
Heptamer
Hexamer

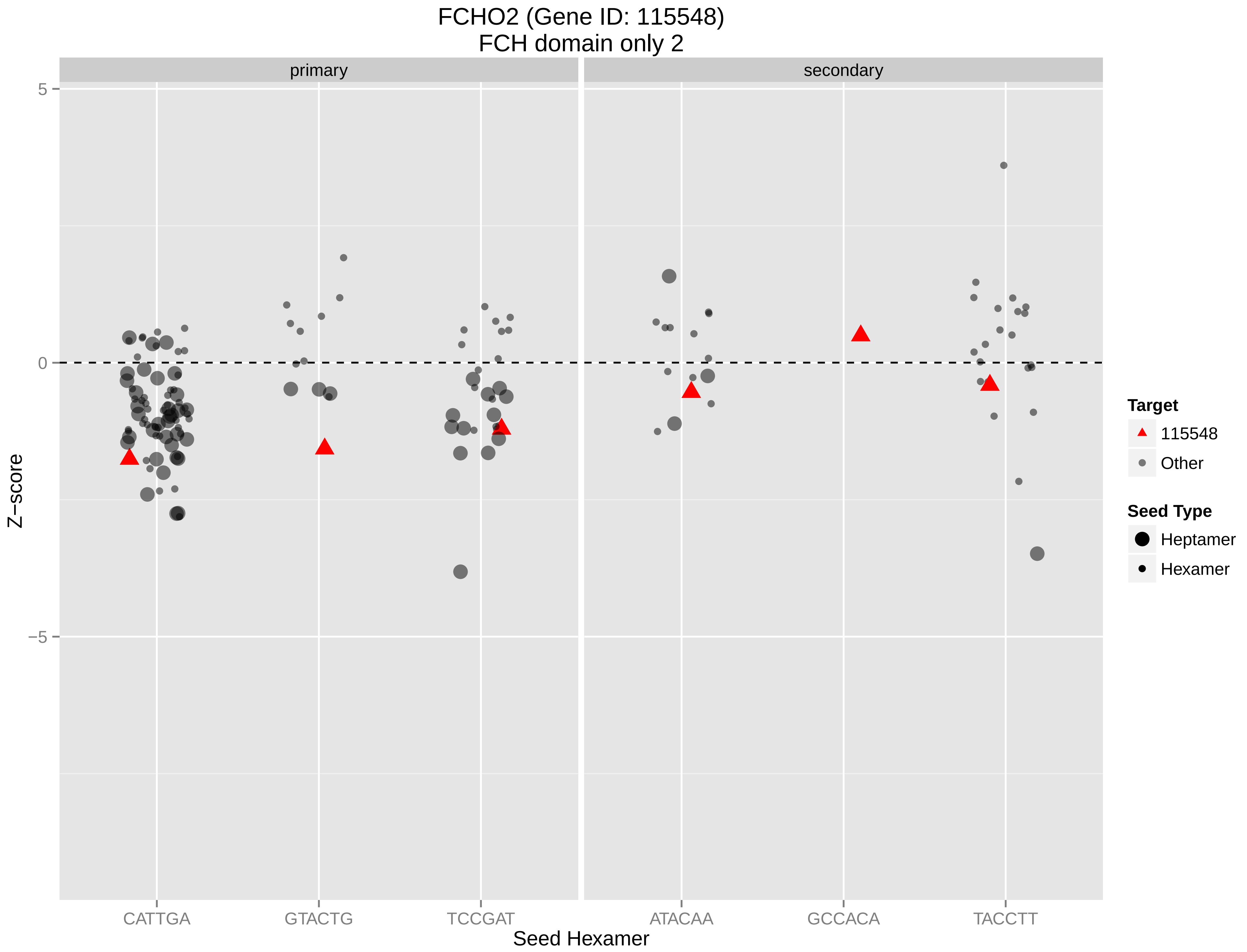

FCHO2 (Gene ID: 115548)
FCH domain only 2
primary
secondary
5
0
−5
Z−score
CATTGA
GTACTG
TCCGAT
ATACAA
GCCACA
TACCTT
Seed Hexamer
Target
115548
Other
Seed Type
Heptamer
Hexamer

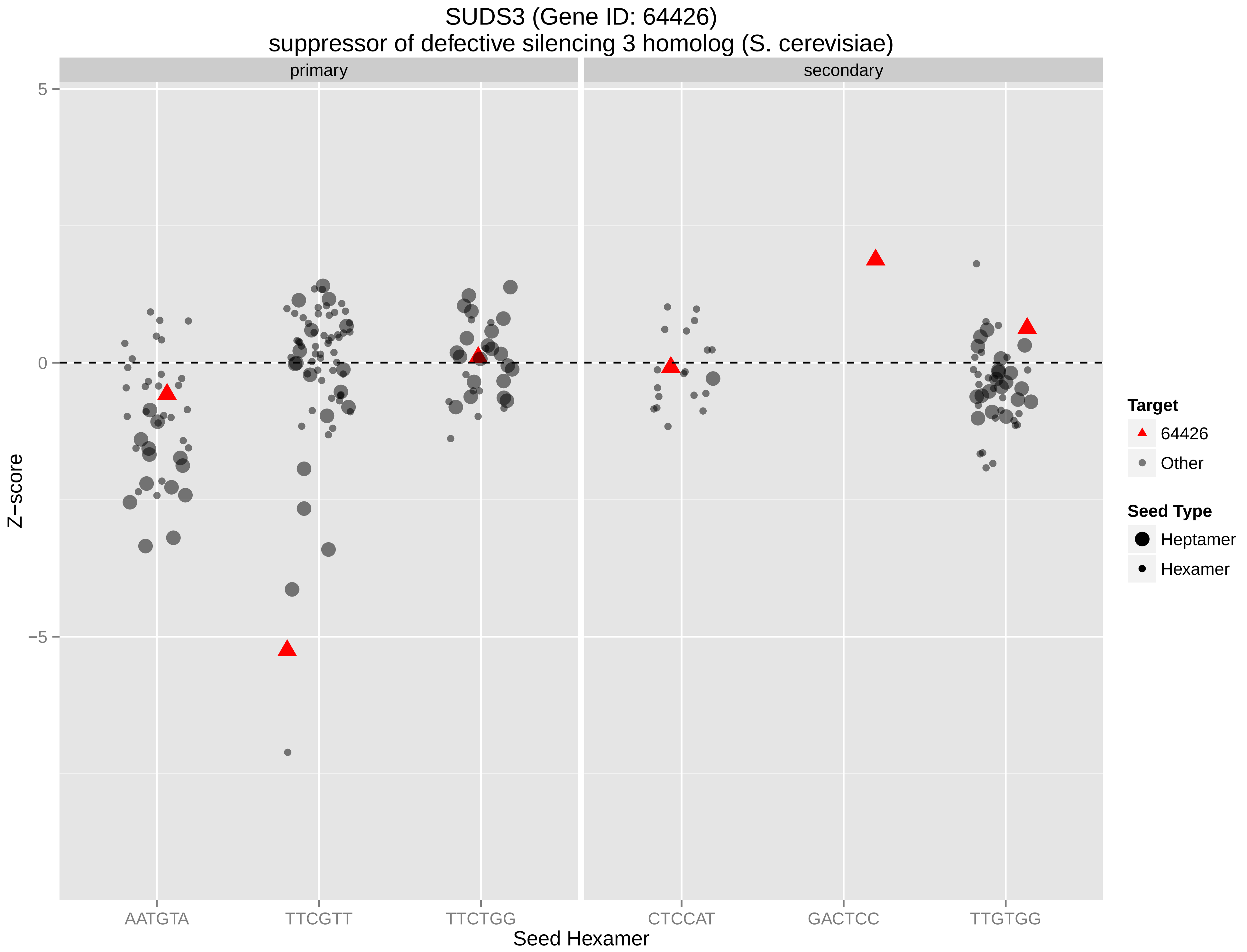

SUDS3 (Gene ID: 64426)
suppressor of defective silencing 3 homolog (S. cerevisiae)
primary
secondary
5
0
−5
Z−score
AATGTA
TTCGTT
TTCTGG
CTCCAT
GACTCC
TTGTGG
Seed Hexamer
Target
64426
Other
Seed Type
Heptamer
Hexamer

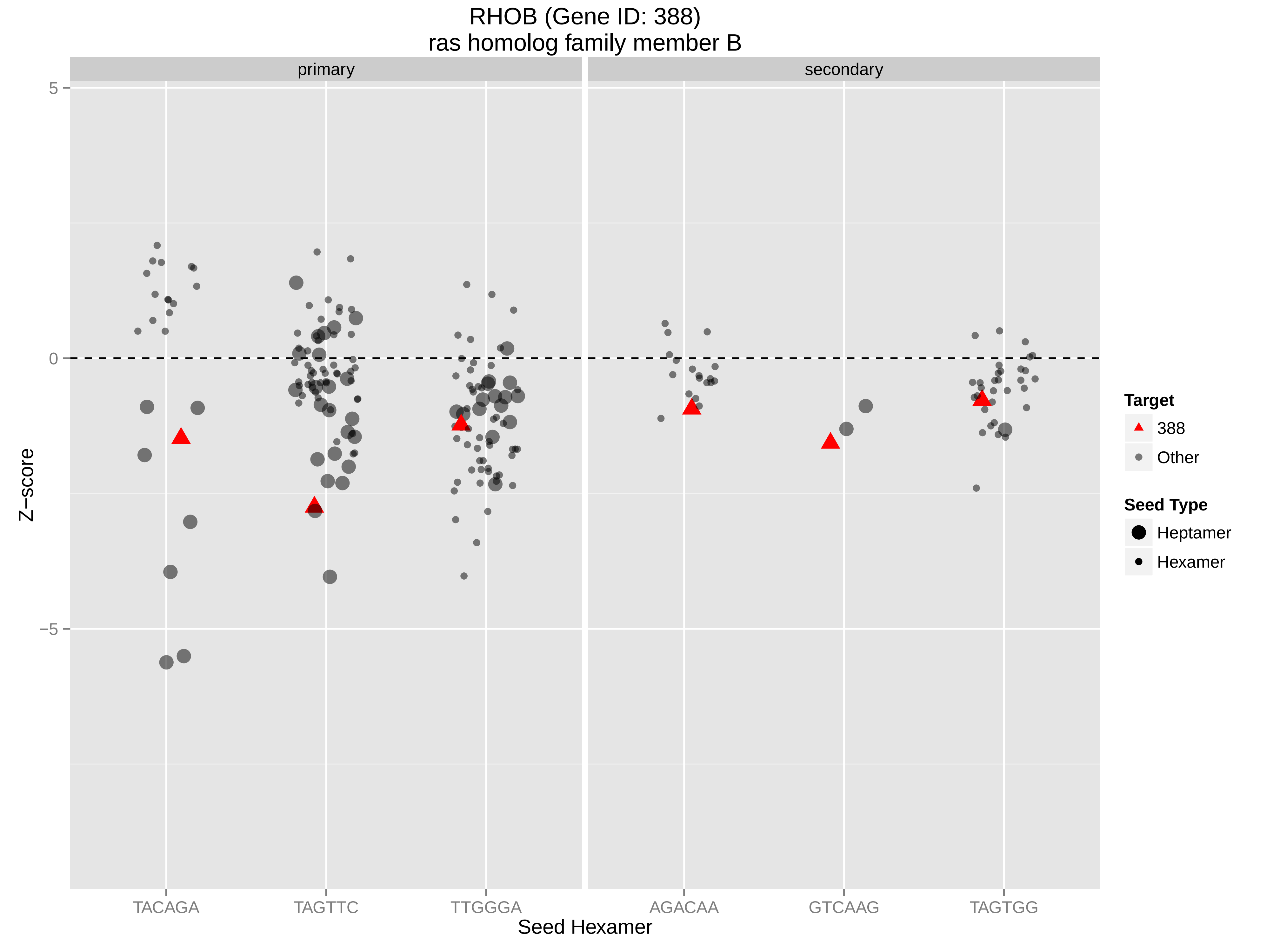
RHOB (Gene ID: 388)
ras homolog family member B
primary
secondary
5
0
−5
Z−score
TACAGA
TAGTTC
TTGGGA
AGACAA
GTCAAG
TAGTGG
Seed Hexamer
Target
388
Other
Seed Type
Heptamer
Hexamer

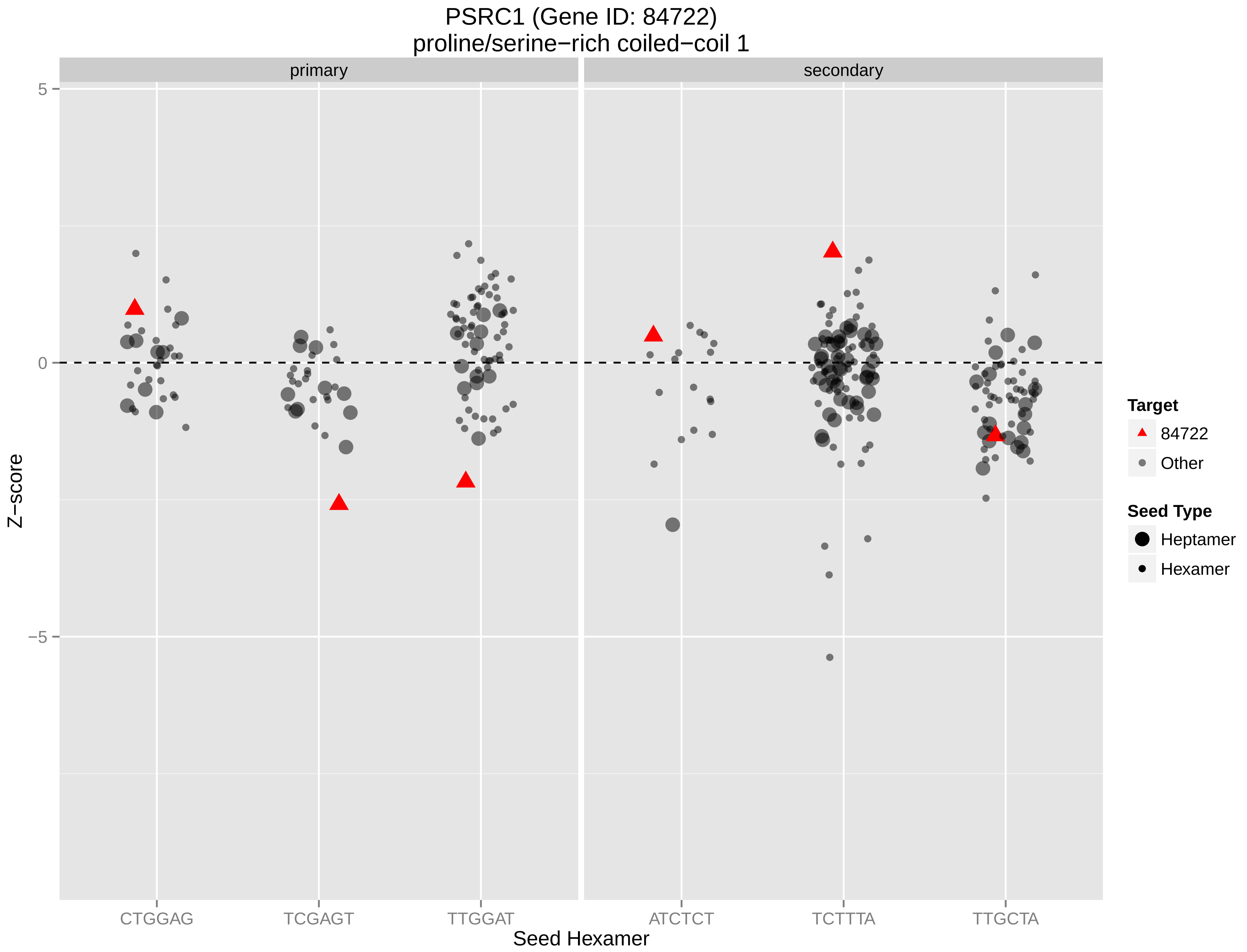

PSRC1 (Gene ID: 84722)
proline/serine−rich coiled−coil 1
primary
secondary
5
0
−5
Z−score
CTGGAG
TCGAGT
TTGGAT
ATCTCT
TCTTTA
TTGCTA
Seed Hexamer
Target
84722
Other
Seed Type
Heptamer
Hexamer

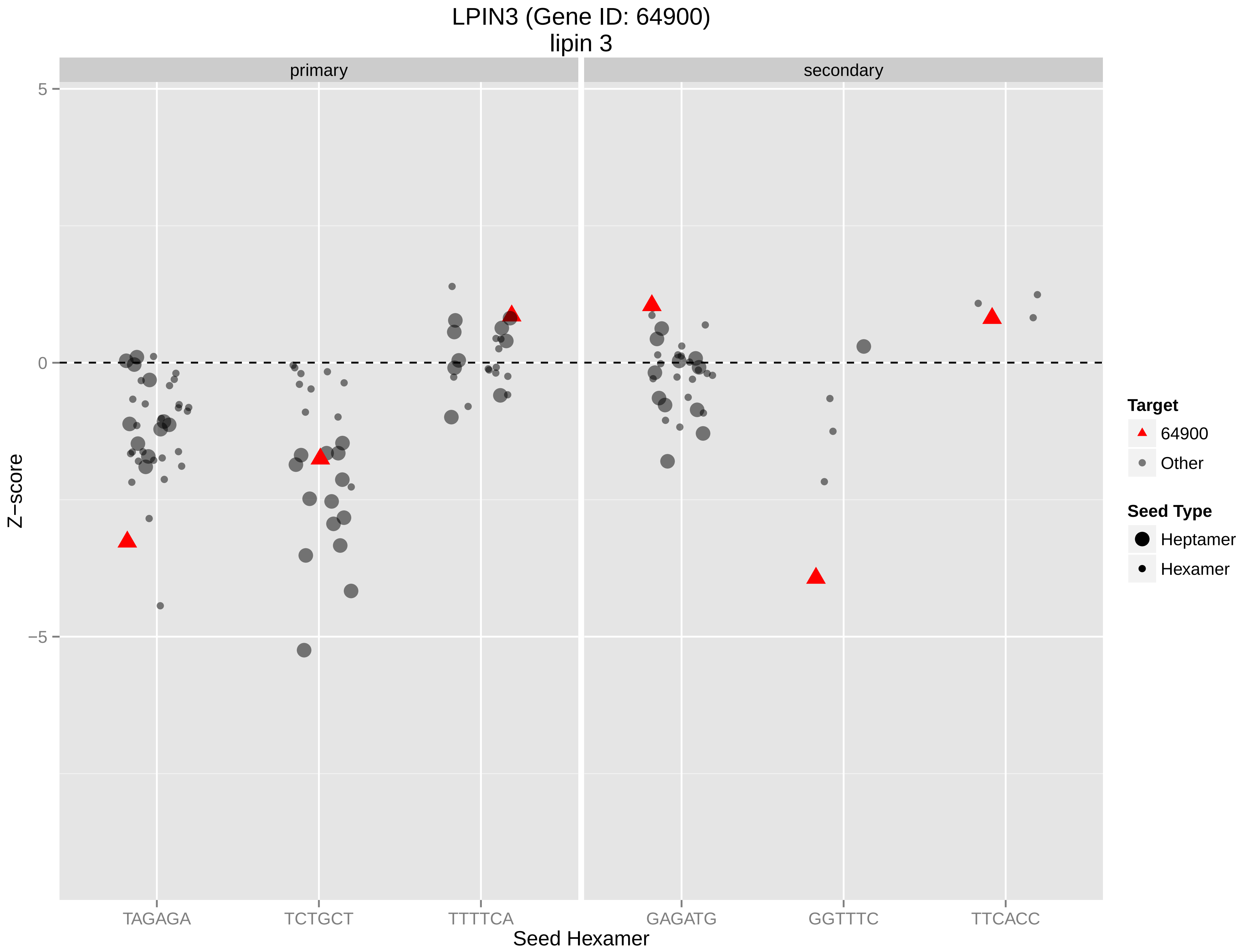
LPIN3 (Gene ID: 64900)
lipin 3
primary
secondary
5
0
−5
Z−score
TAGAGA
TCTGCT
TTTTCA
GAGATG
GGTTTC
TTCACC
Seed Hexamer
Target
64900
Other
Seed Type
Heptamer
Hexamer

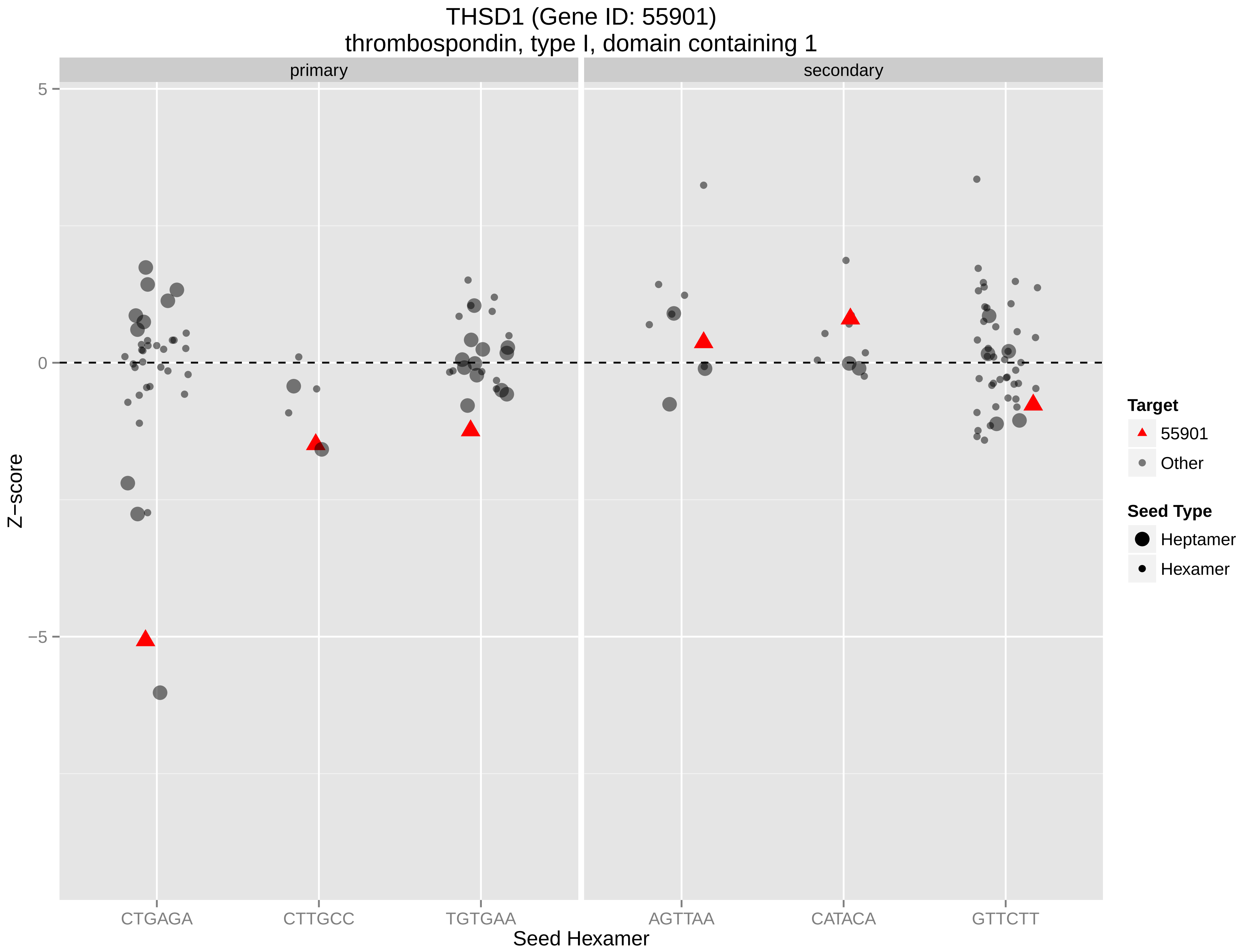

THSD1 (Gene ID: 55901)
thrombospondin, type I, domain containing 1
primary
secondary
5
0
−5
Z−score
CTGAGA
CTTGCC
TGTGAA
AGTTAA
CATACA
GTTCTT
Seed Hexamer
Target
55901
Other
Seed Type
Heptamer
Hexamer

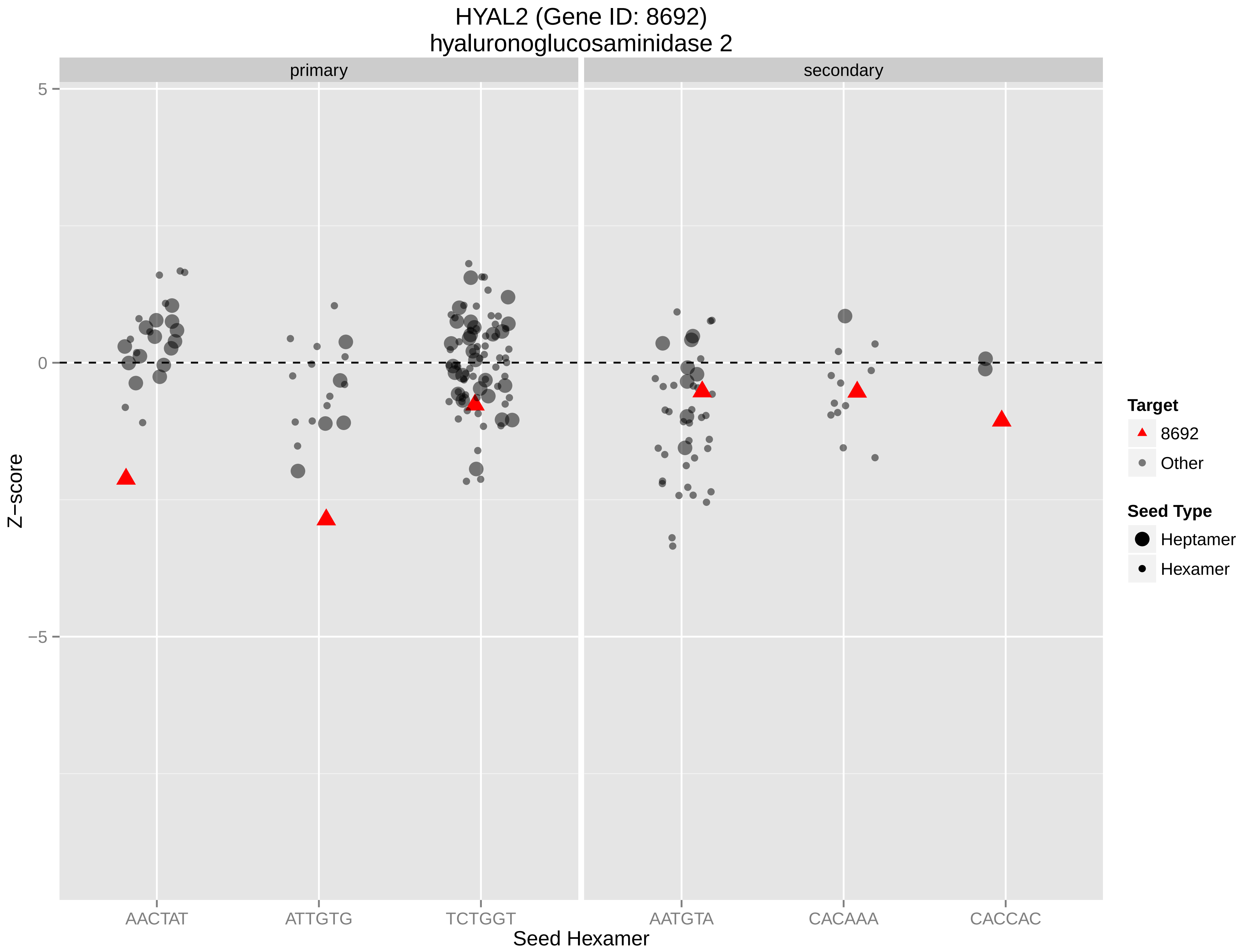
HYAL2 (Gene ID: 8692)
hyaluronoglucosaminidase 2
primary
secondary
5
0
−5
Z−score
AACTAT
ATTGTG
TCTGGT
AATGTA
CACAAA
CACCAC
Seed Hexamer
Target
8692
Other
Seed Type
Heptamer
Hexamer

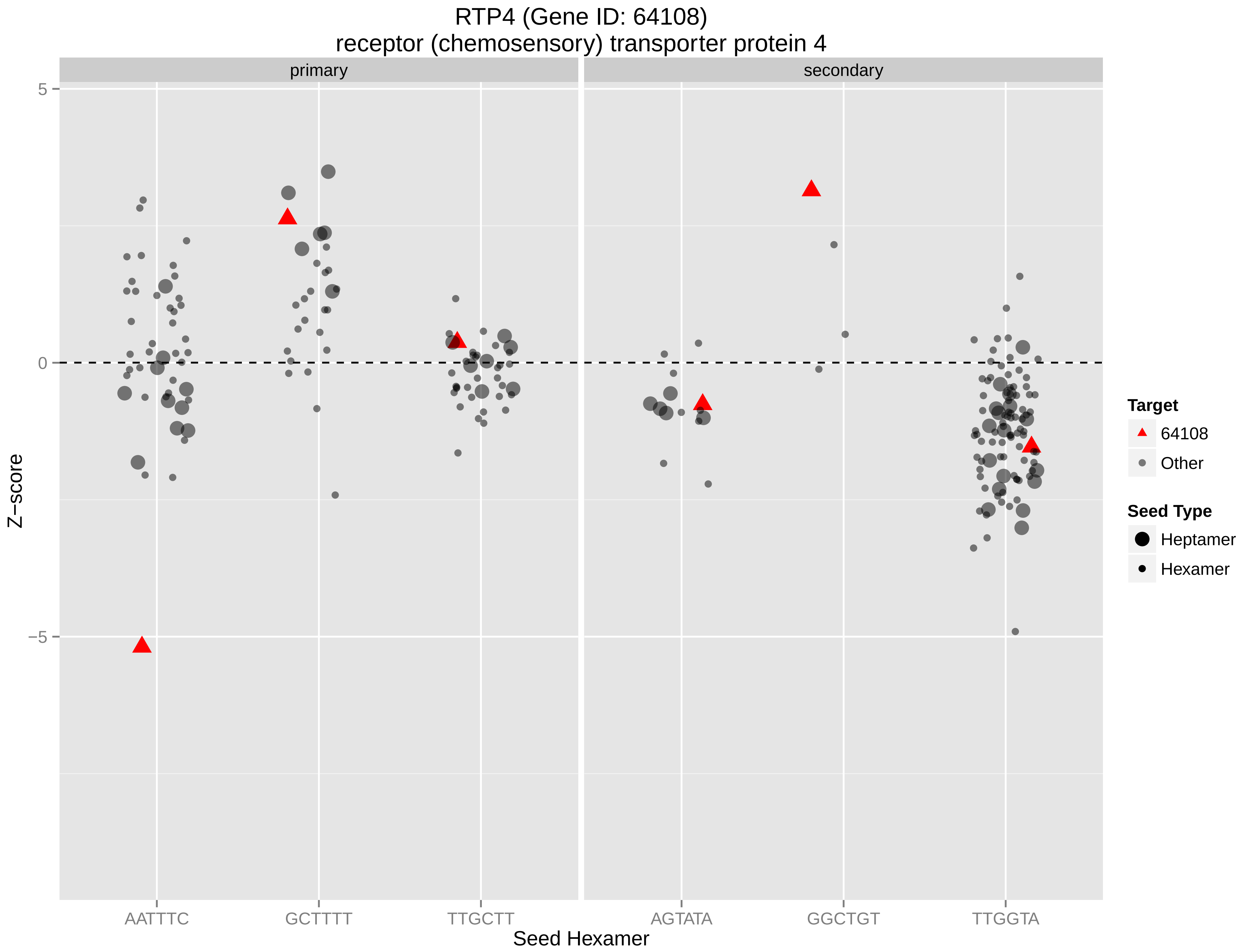

RTP4 (Gene ID: 64108)
receptor (chemosensory) transporter protein 4
primary
secondary
5
0
−5
Z−score
AATTTC
GCTTTT
TTGCTT
AGTATA
GGCTGT
TTGGTA
Seed Hexamer
Target
64108
Other
Seed Type
Heptamer
Hexamer

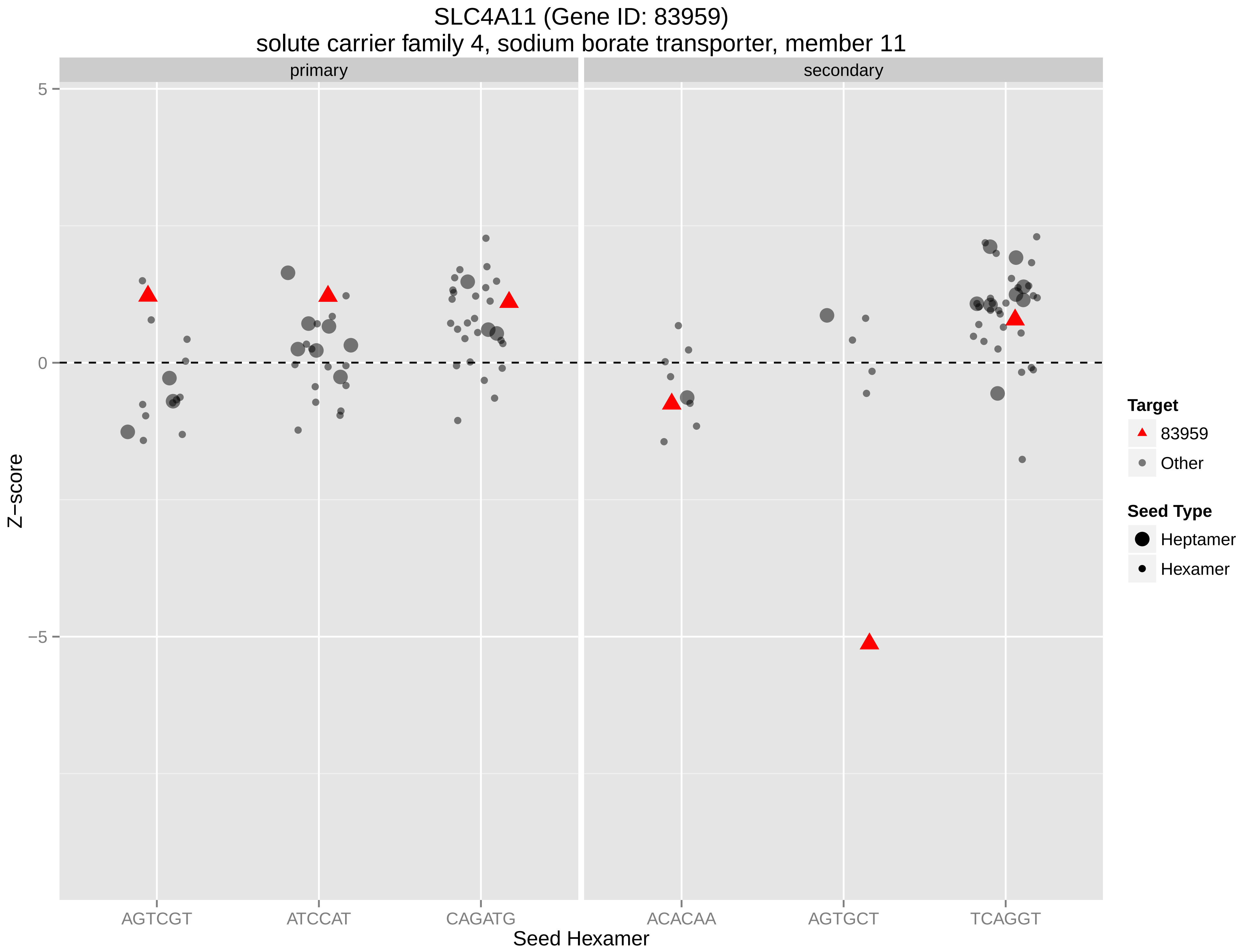
SLC4A11 (Gene ID: 83959)
solute carrier family 4, sodium borate transporter, member 11
primary
secondary
5
0
−5
Z−score
AGTCGT
ATCCAT
CAGATG
ACACAA
AGTGCT
TCAGGT
Seed Hexamer
Target
83959
Other
Seed Type
Heptamer
Hexamer

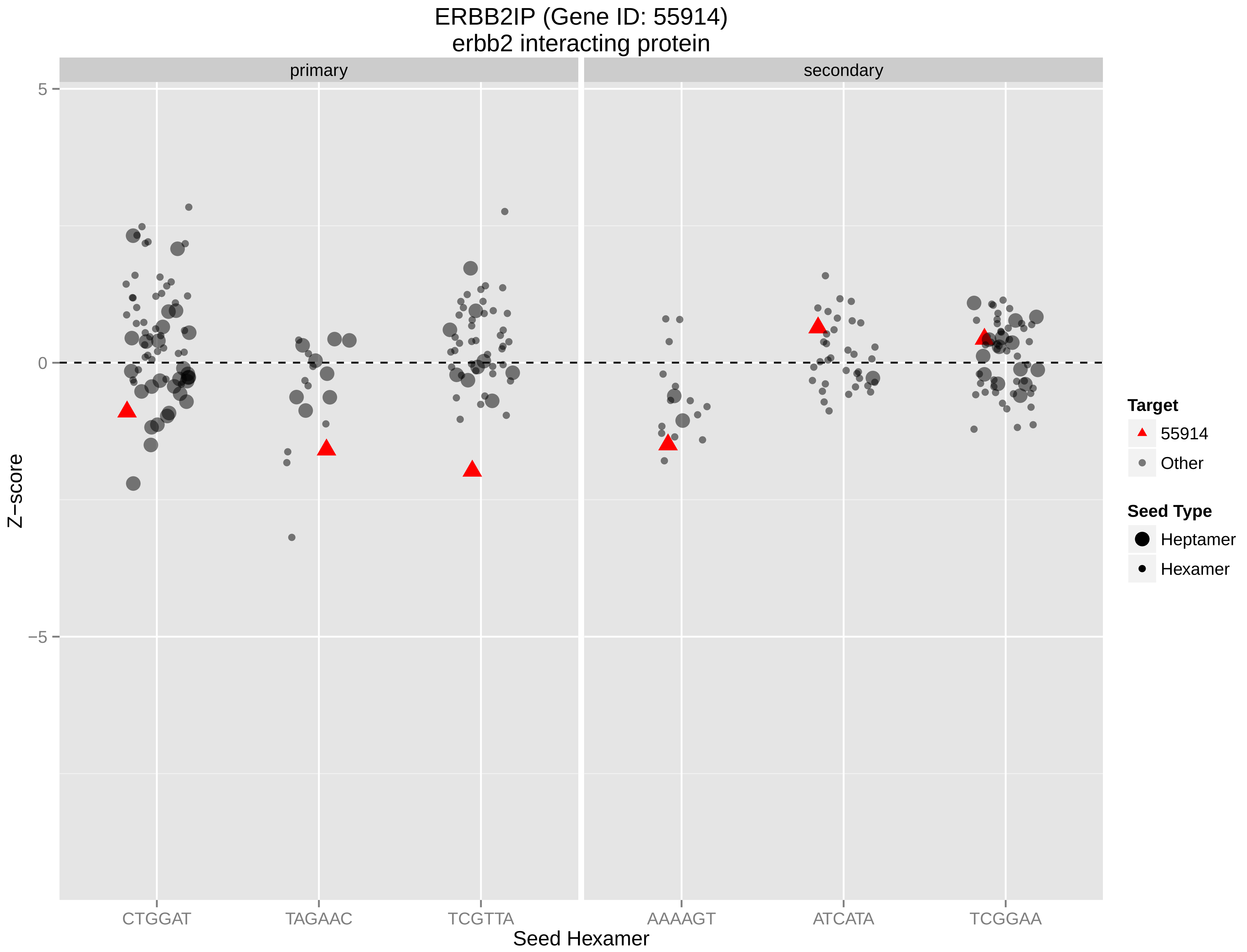
ERBB2IP (Gene ID: 55914)
erbb2 interacting protein
primary
secondary
5
0
−5
Z−score
CTGGAT
TAGAAC
TCGTTA
AAAAGT
ATCATA
TCGGAA
Seed Hexamer
Target
55914
Other
Seed Type
Heptamer
Hexamer

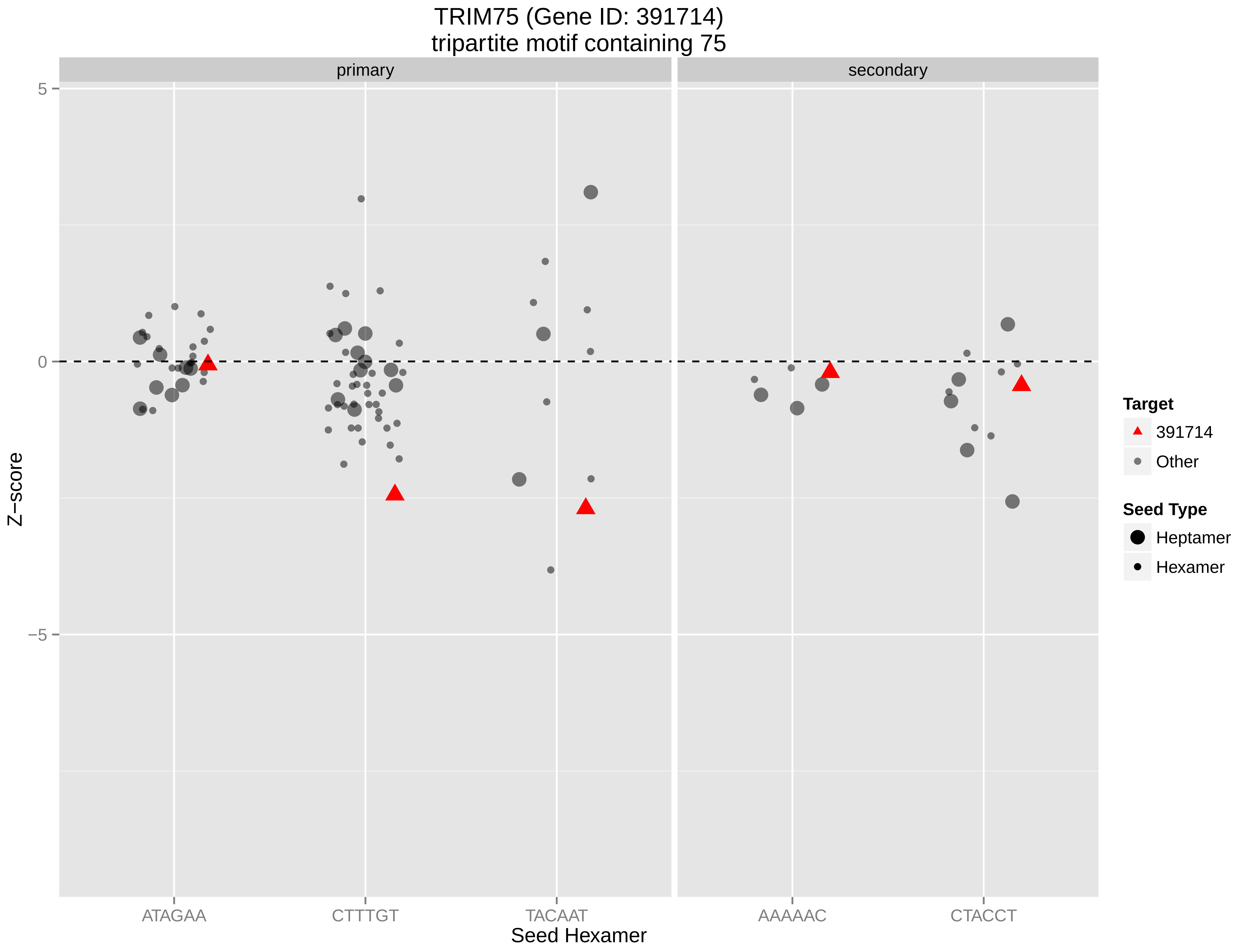

TRIM75 (Gene ID: 391714)
tripartite motif containing 75
primary
secondary
5
0
−5
Z−score
ATAGAA
CTTTGT
TACAAT
AAAAAC
CTACCT
Seed Hexamer
Target
391714
Other
Seed Type
Heptamer
Hexamer

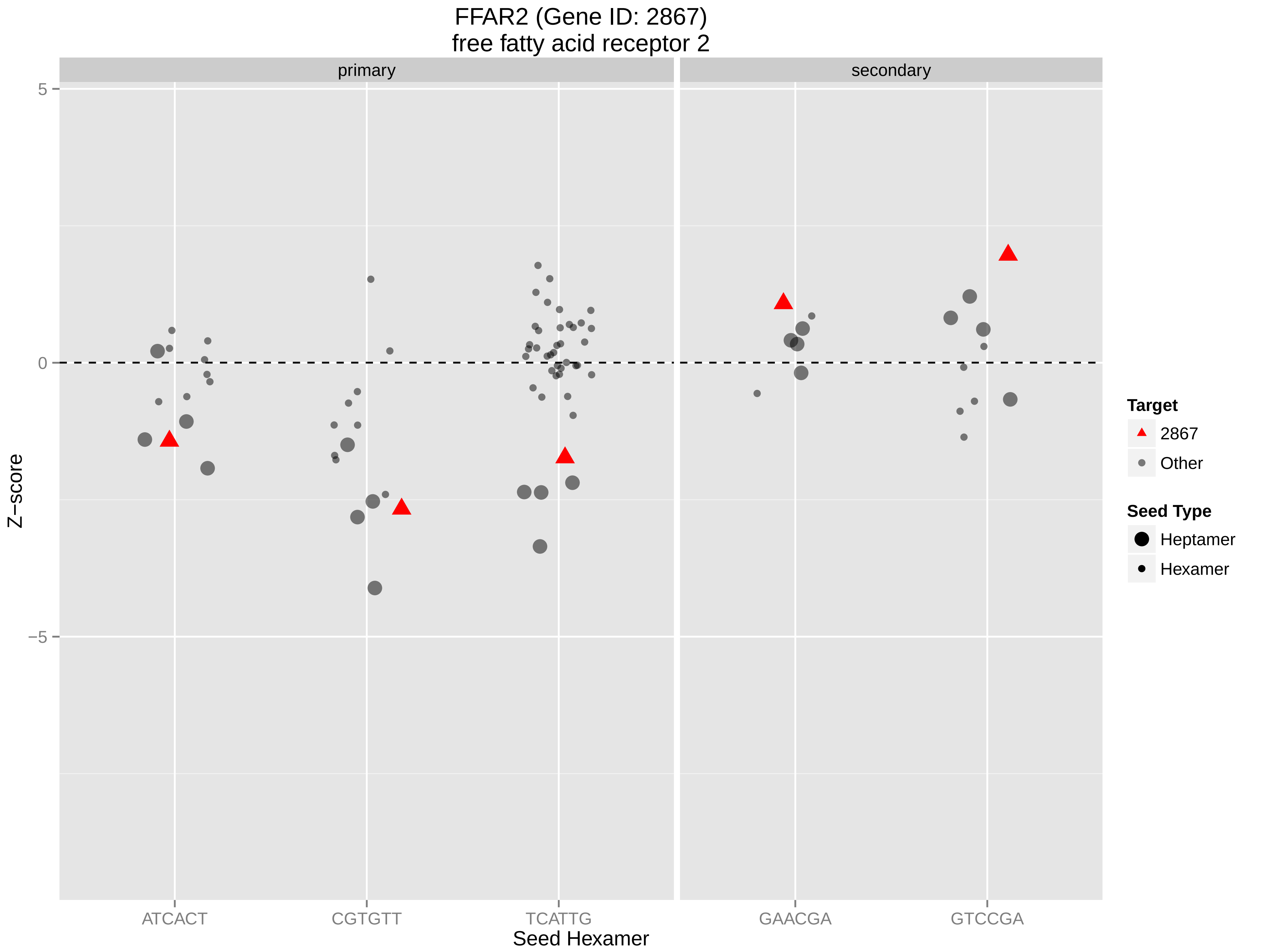
FFAR2 (Gene ID: 2867)
free fatty acid receptor 2
primary
secondary
5
0
−5
Z−score
ATCACT
CGTGTT
TCATTG
GAACGA
GTCCGA
Seed Hexamer
Target
2867
Other
Seed Type
Heptamer
Hexamer

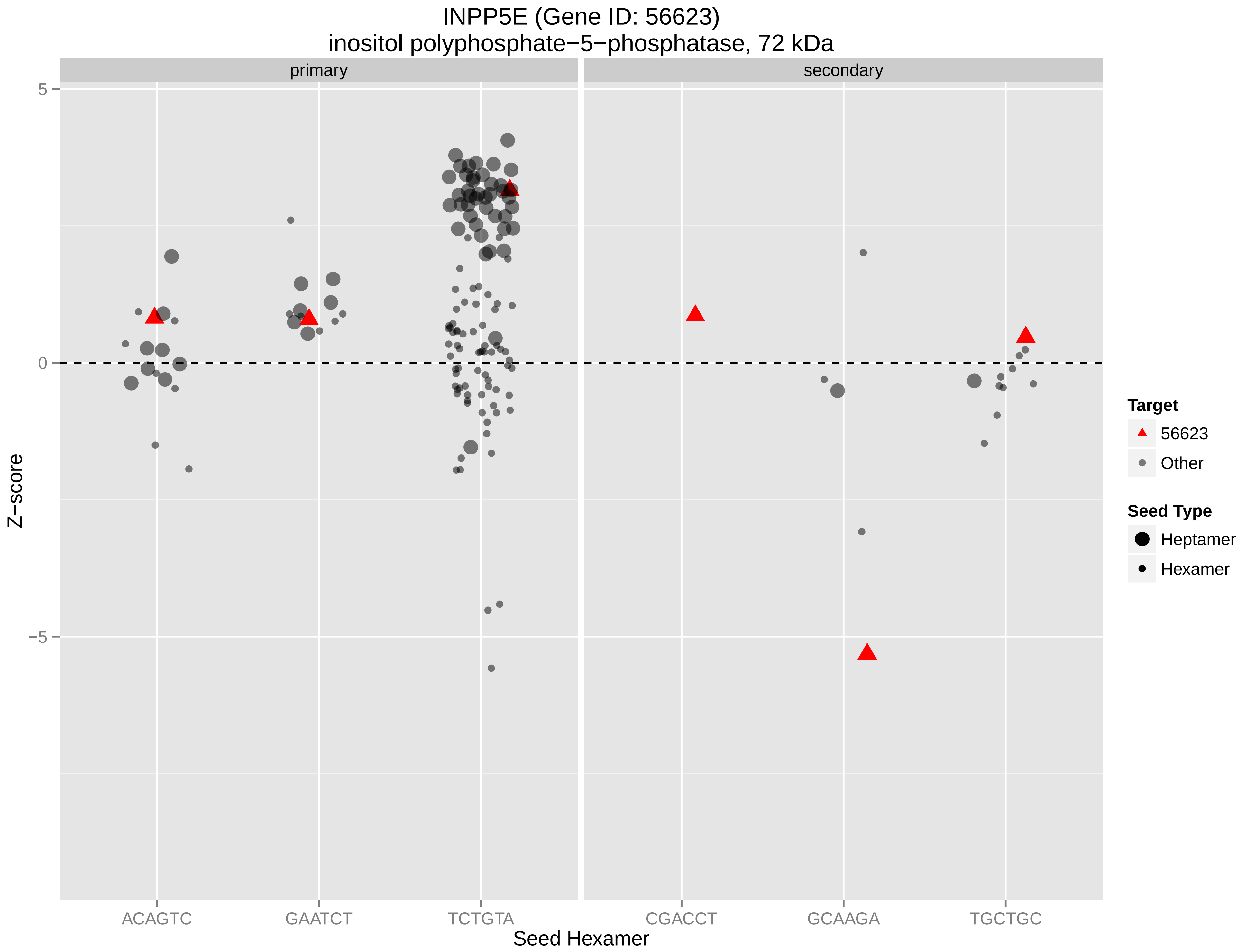
INPP5E (Gene ID: 56623)
inositol polyphosphate−5−phosphatase, 72 kDa
primary
secondary
5
0
−5
Z−score
ACAGTC
GAATCT
TCTGTA
CGACCT
GCAAGA
TGCTGC
Seed Hexamer
Target
56623
Other
Seed Type
Heptamer
Hexamer

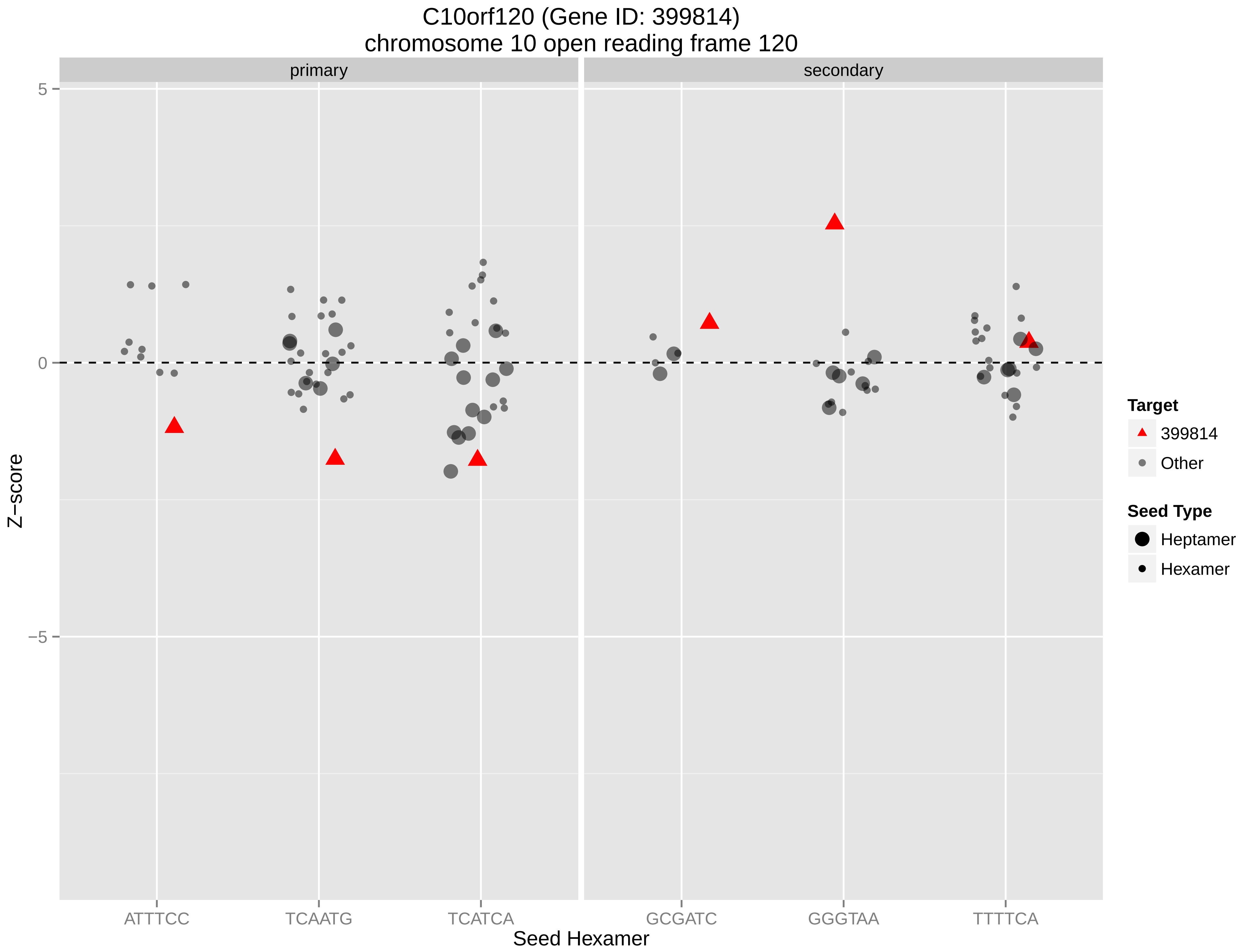
C10orf120 (Gene ID: 399814)
chromosome 10 open reading frame 120
primary
secondary
5
0
−5
Z−score
ATTTCC
TCAATG
TCATCA
GCGATC
GGGTAA
TTTTCA
Seed Hexamer
Target
399814
Other
Seed Type
Heptamer
Hexamer

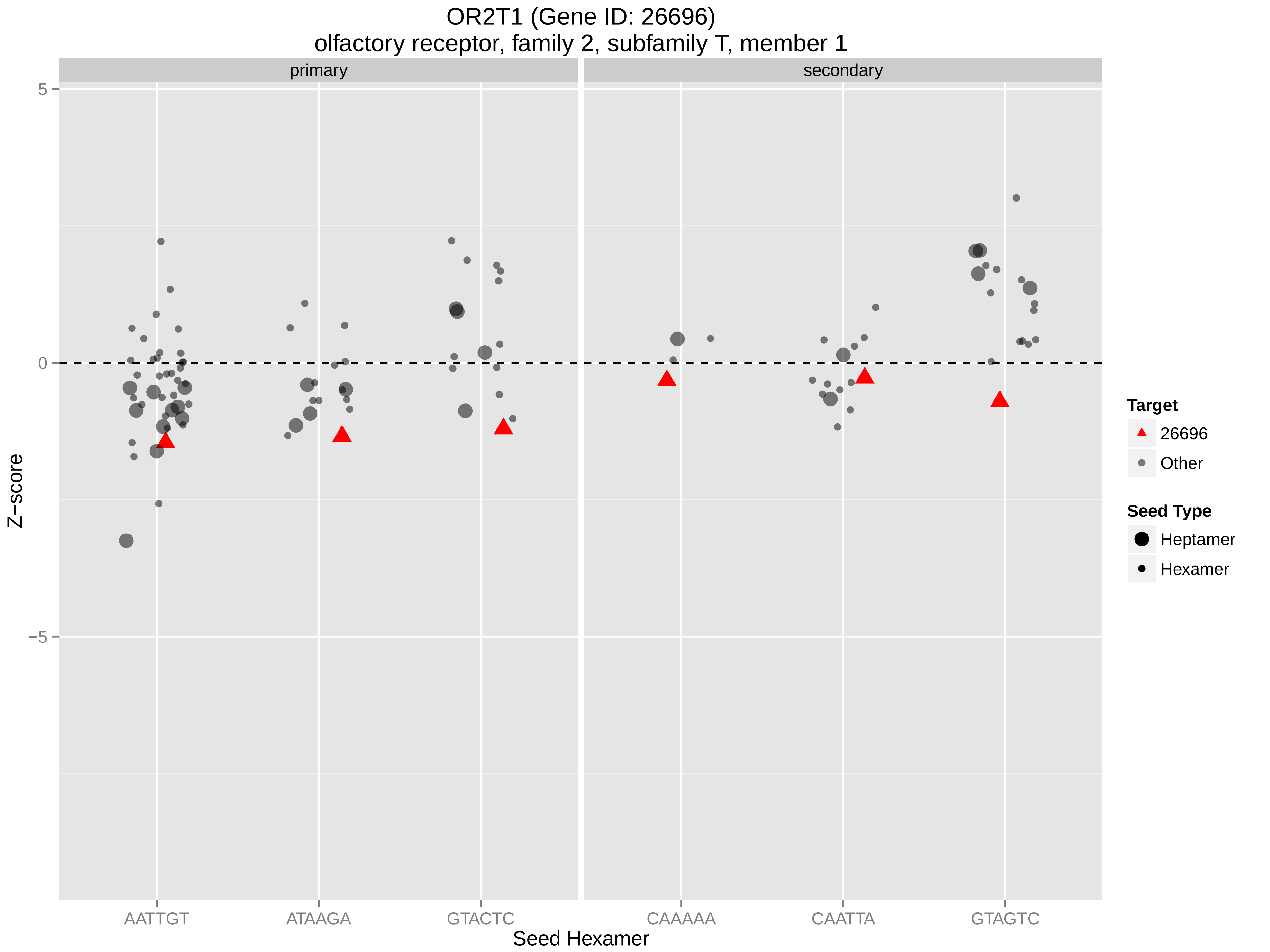

OR2T1 (Gene ID: 26696)
olfactory receptor, family 2, subfamily T, member 1
primary
secondary
5
0
−5
Z−score
AATTGT
ATAAGA
GTACTC
CAAAAA
CAATTA
GTAGTC
Seed Hexamer
Target
26696
Other
Seed Type
Heptamer
Hexamer

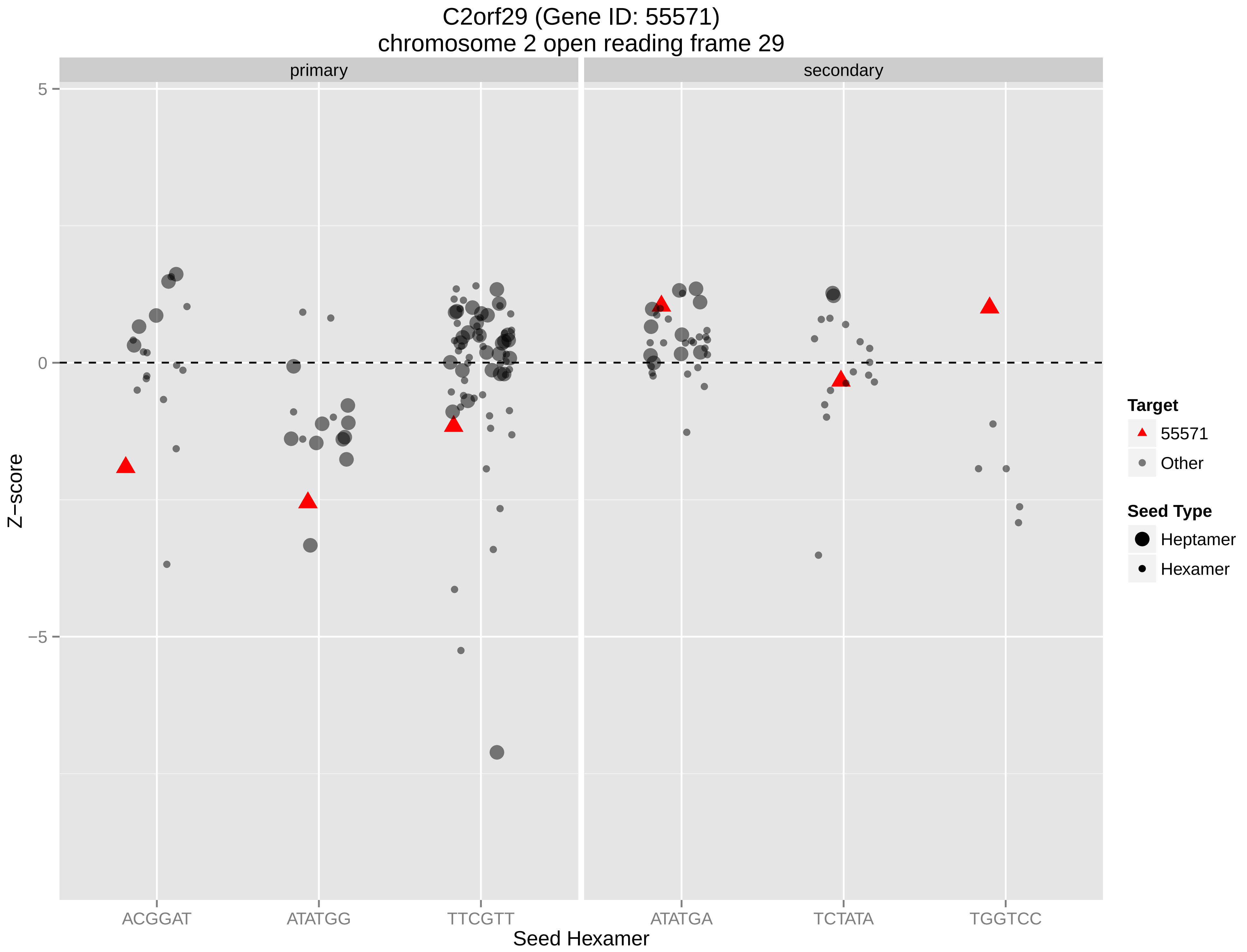
C2orf29 (Gene ID: 55571)
chromosome 2 open reading frame 29
primary
secondary
5
0
−5
Z−score
ACGGAT
ATATGG
TTCGTT
ATATGA
TCTATA
TGGTCC
Seed Hexamer
Target
55571
Other
Seed Type
Heptamer
Hexamer

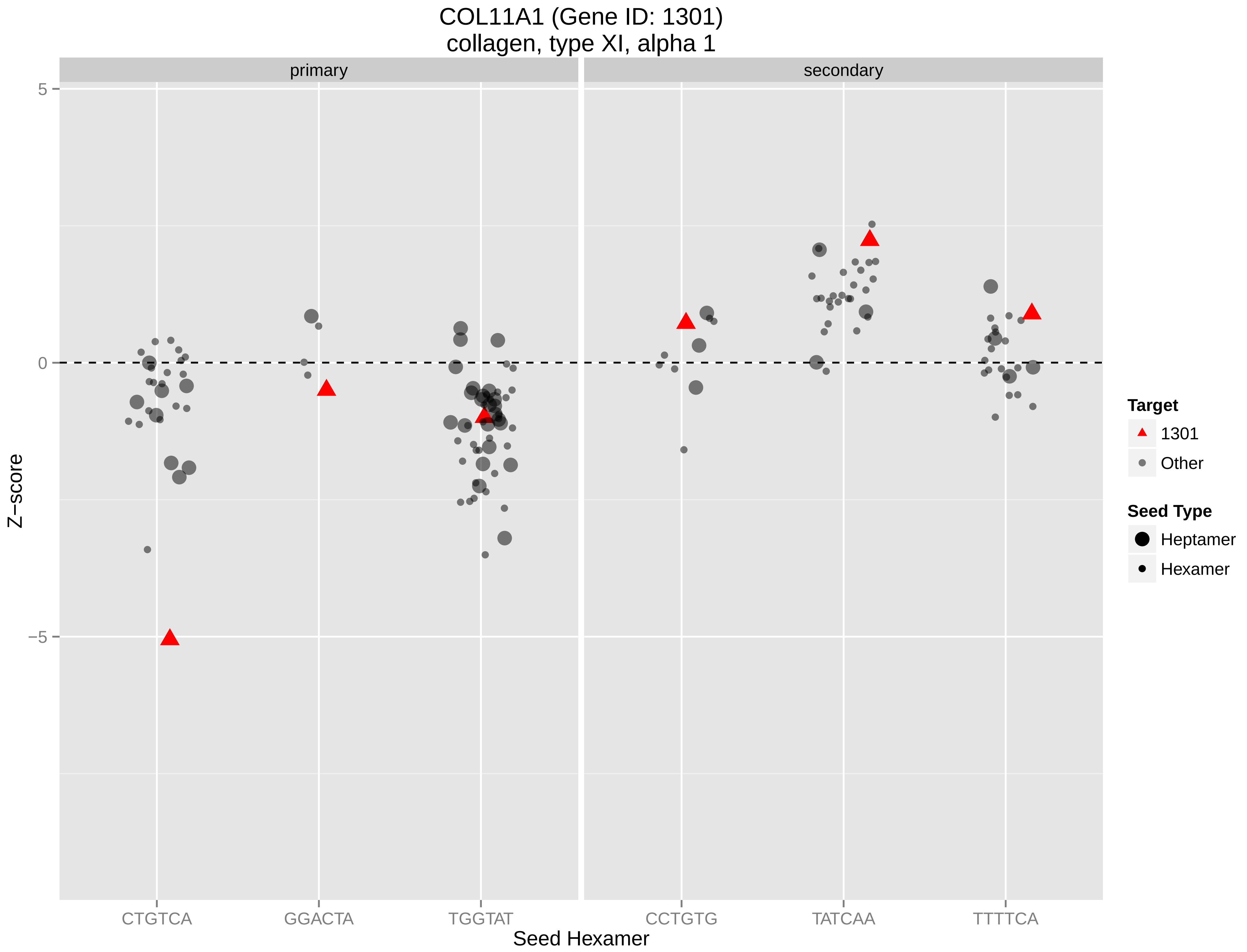
COL11A1 (Gene ID: 1301)
collagen, type XI, alpha 1
primary
secondary
5
0
−5
Z−score
CTGTCA
GGACTA
TGGTAT
CCTGTG
TATCAA
TTTTCA
Seed Hexamer
Target
1301
Other
Seed Type
Heptamer
Hexamer

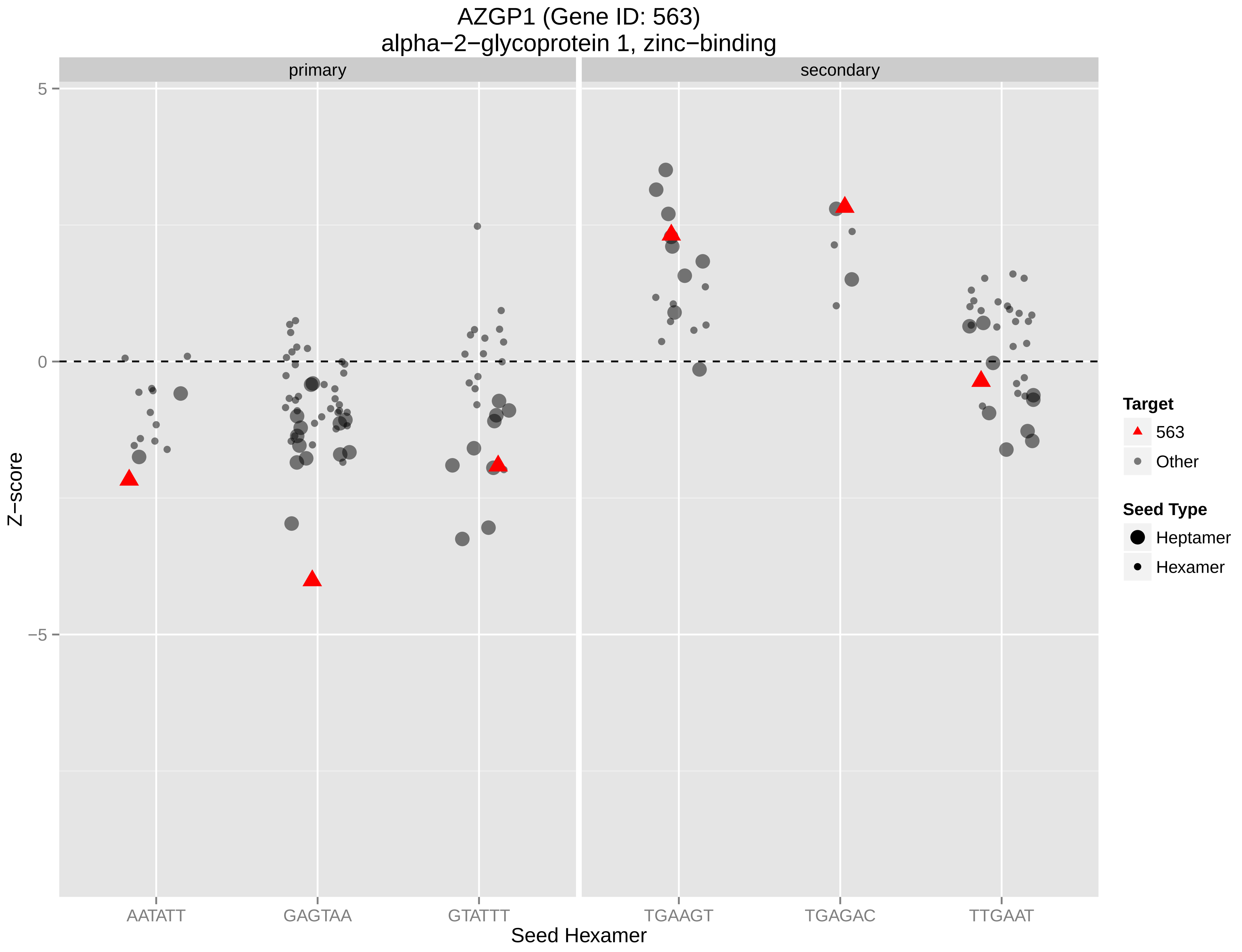

AZGP1 (Gene ID: 563)
alpha−2−glycoprotein 1, zinc−binding
primary
secondary
5
0
−5
Z−score
AATATT
GAGTAA
GTATTT
TGAAGT
TGAGAC
TTGAAT
Seed Hexamer
Target
563
Other
Seed Type
Heptamer
Hexamer

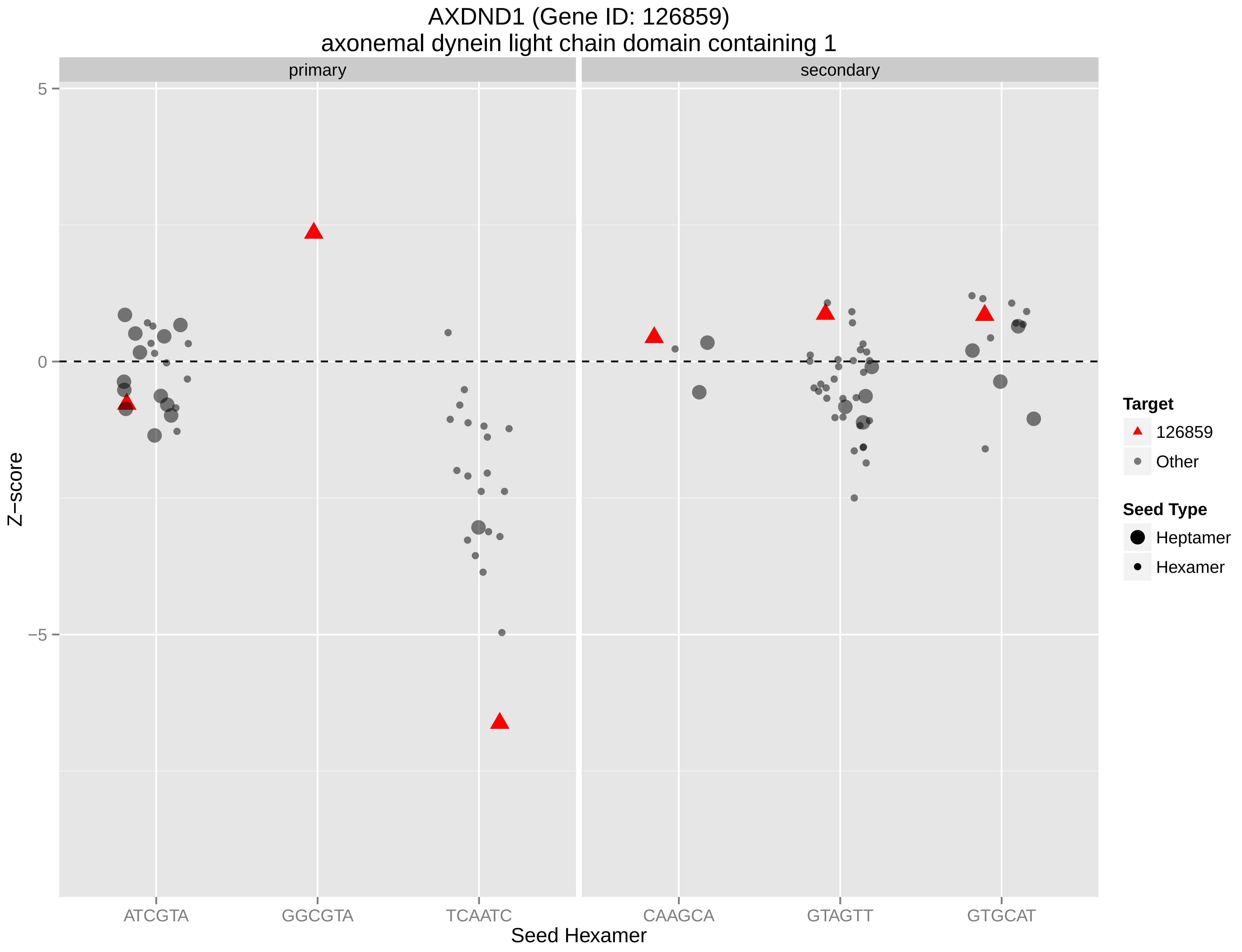

AXDND1 (Gene ID: 126859)
axonemal dynein light chain domain containing 1
primary
secondary
5
0
−5
Z−score
ATCGTA
GGCGTA
TCAATC
CAAGCA
GTAGTT
GTGCAT
Seed Hexamer
Target
126859
Other
Seed Type
Heptamer
Hexamer

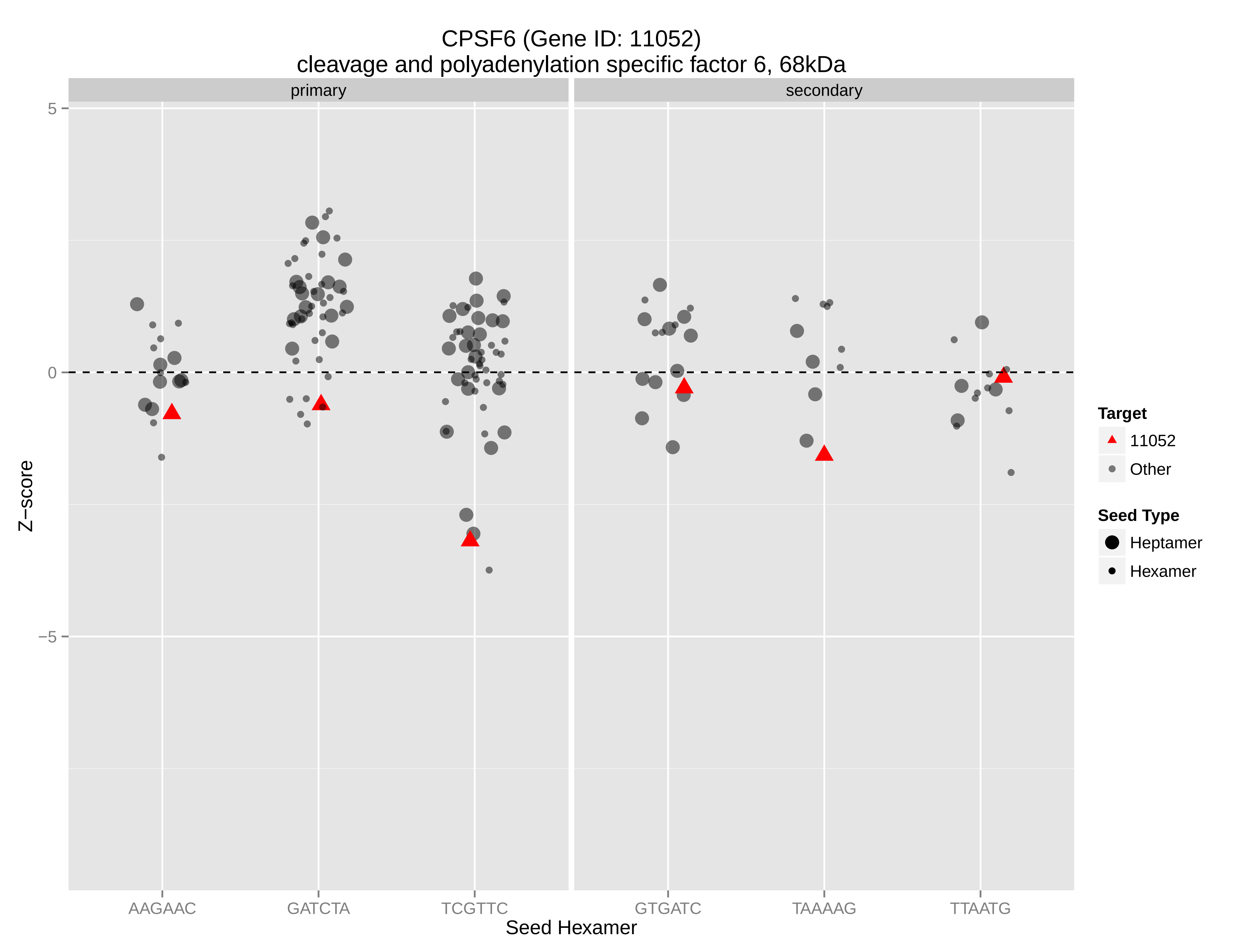

CPSF6 (Gene ID: 11052)
cleavage and polyadenylation specific factor 6, 68kDa
primary
secondary
5
0
−5
Z−score
AAGAAC
GATCTA
TCGTTC
GTGATC
TAAAAG
TTAATG
Seed Hexamer
Target
11052
Other
Seed Type
Heptamer
Hexamer

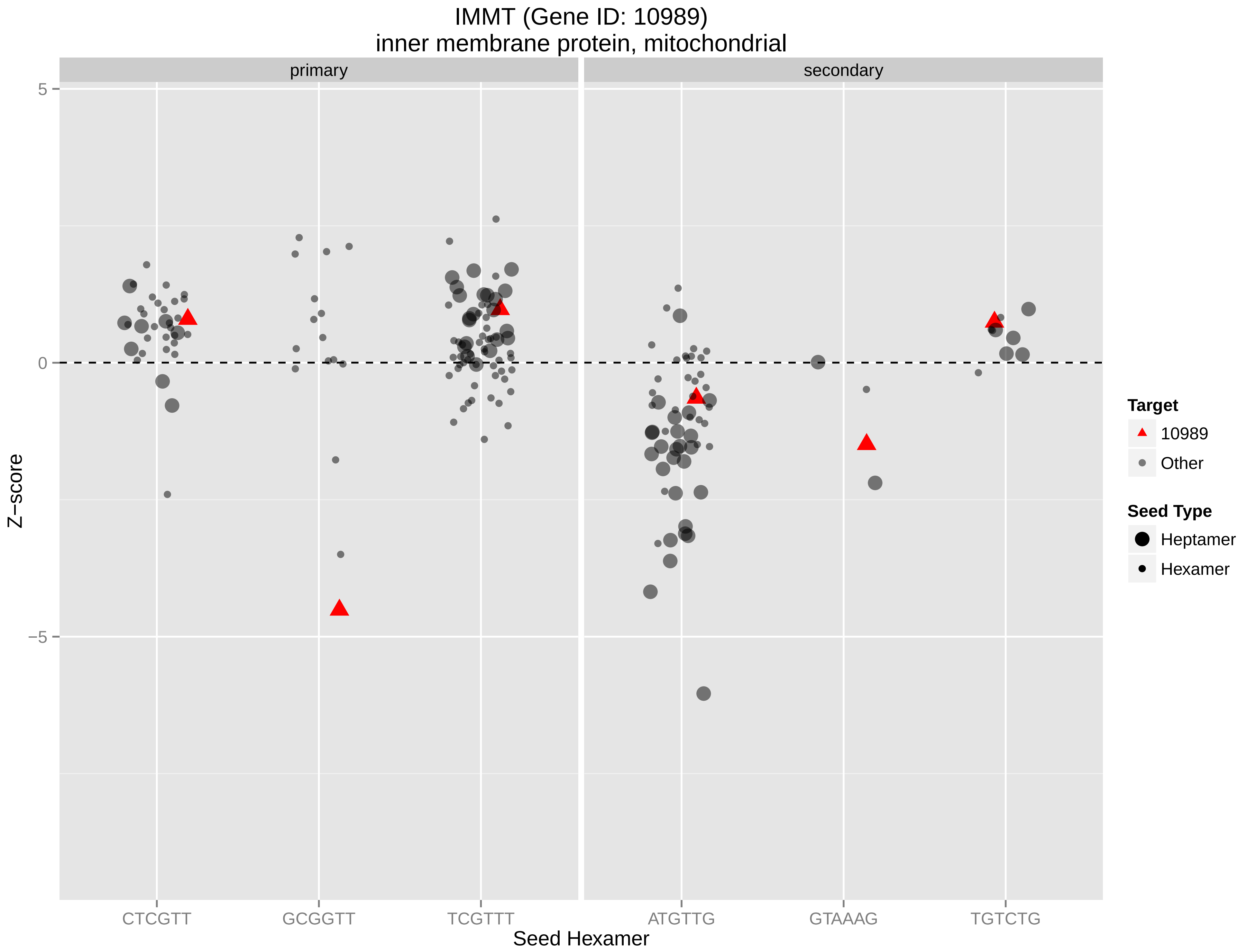

IMMT (Gene ID: 10989)
inner membrane protein, mitochondrial
primary
secondary
5
0
−5
Z−score
CTCGTT
GCGGTT
TCGTTT
ATGTTG
GTAAAG
TGTCTG
Seed Hexamer
Target
10989
Other
Seed Type
Heptamer
Hexamer

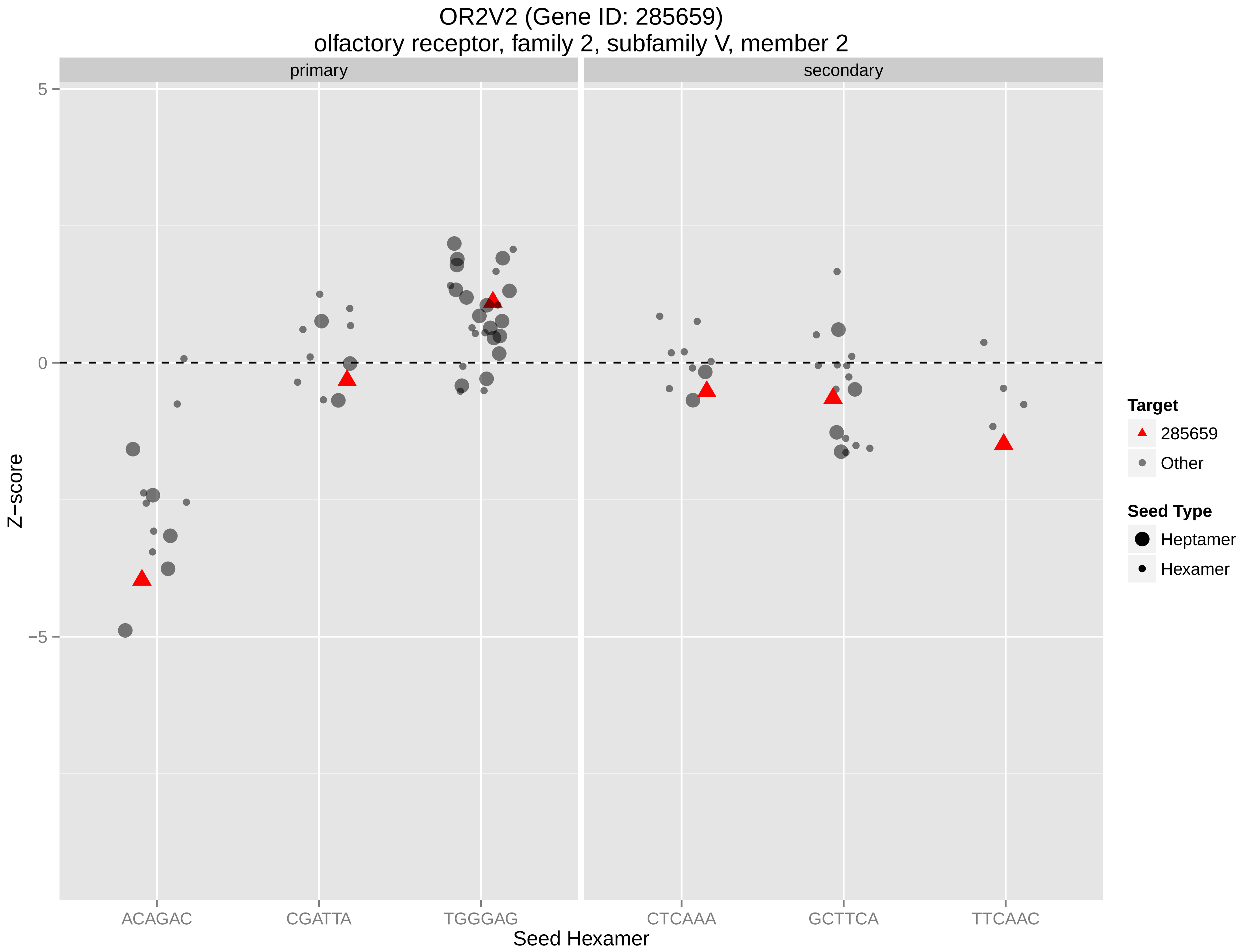

OR2V2 (Gene ID: 285659)
olfactory receptor, family 2, subfamily V, member 2
primary
secondary
5
0
−5
Z−score
ACAGAC
CGATTA
TGGGAG
CTCAAA
GCTTCA
TTCAAC
Seed Hexamer
Target
285659
Other
Seed Type
Heptamer
Hexamer

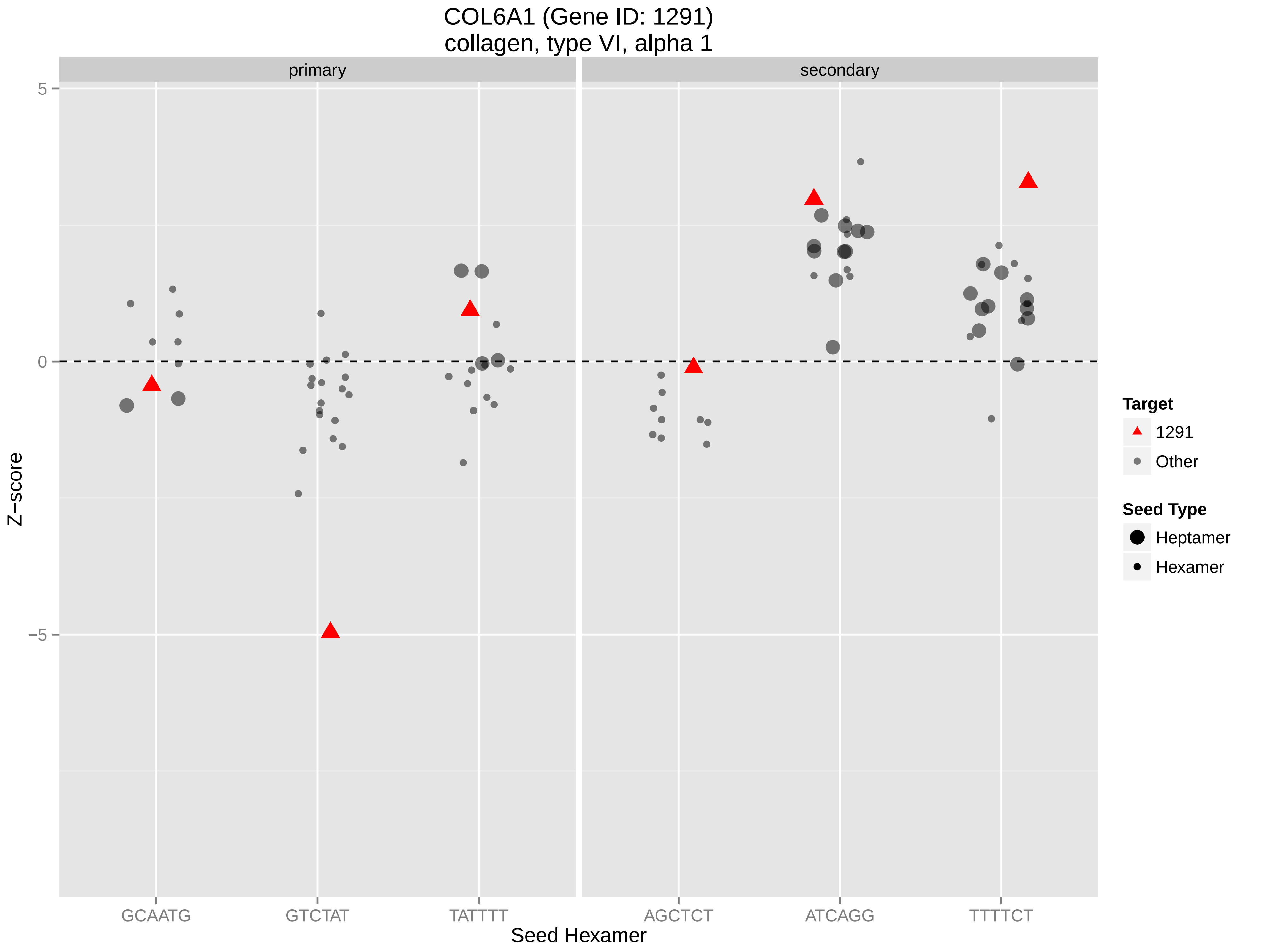
COL6A1 (Gene ID: 1291)
collagen, type VI, alpha 1
primary
secondary
5
0
−5
Z−score
GCAATG
GTCTAT
TATTTT
AGCTCT
ATCAGG
TTTTCT
Seed Hexamer
Target
1291
Other
Seed Type
Heptamer
Hexamer

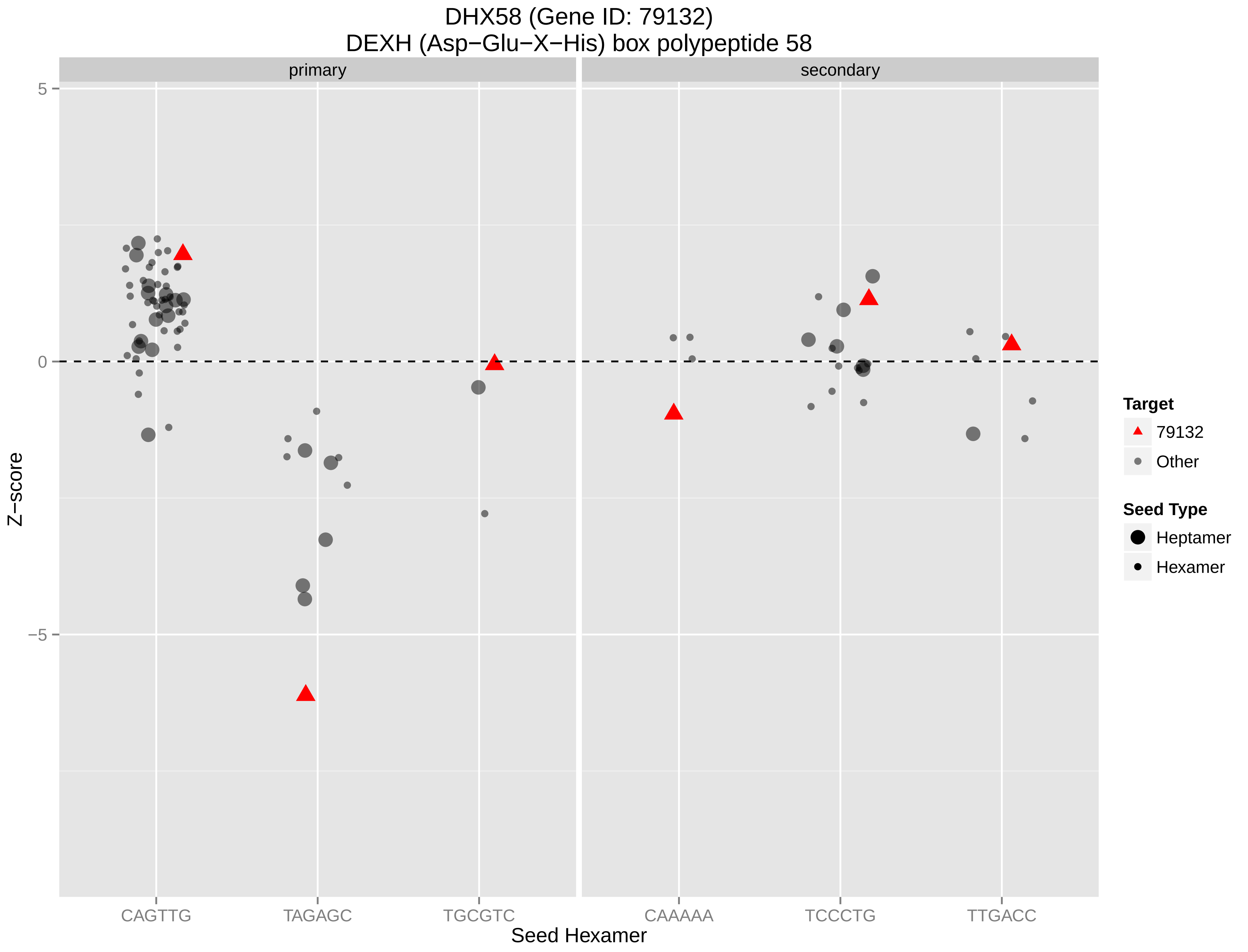

DHX58 (Gene ID: 79132)
DEXH (Asp−Glu−X−His) box polypeptide 58
primary
secondary
5
0
−5
Z−score
CAGTTG
TAGAGC
TGCGTC
CAAAAA
TCCCTG
TTGACC
Seed Hexamer
Target
79132
Other
Seed Type
Heptamer
Hexamer

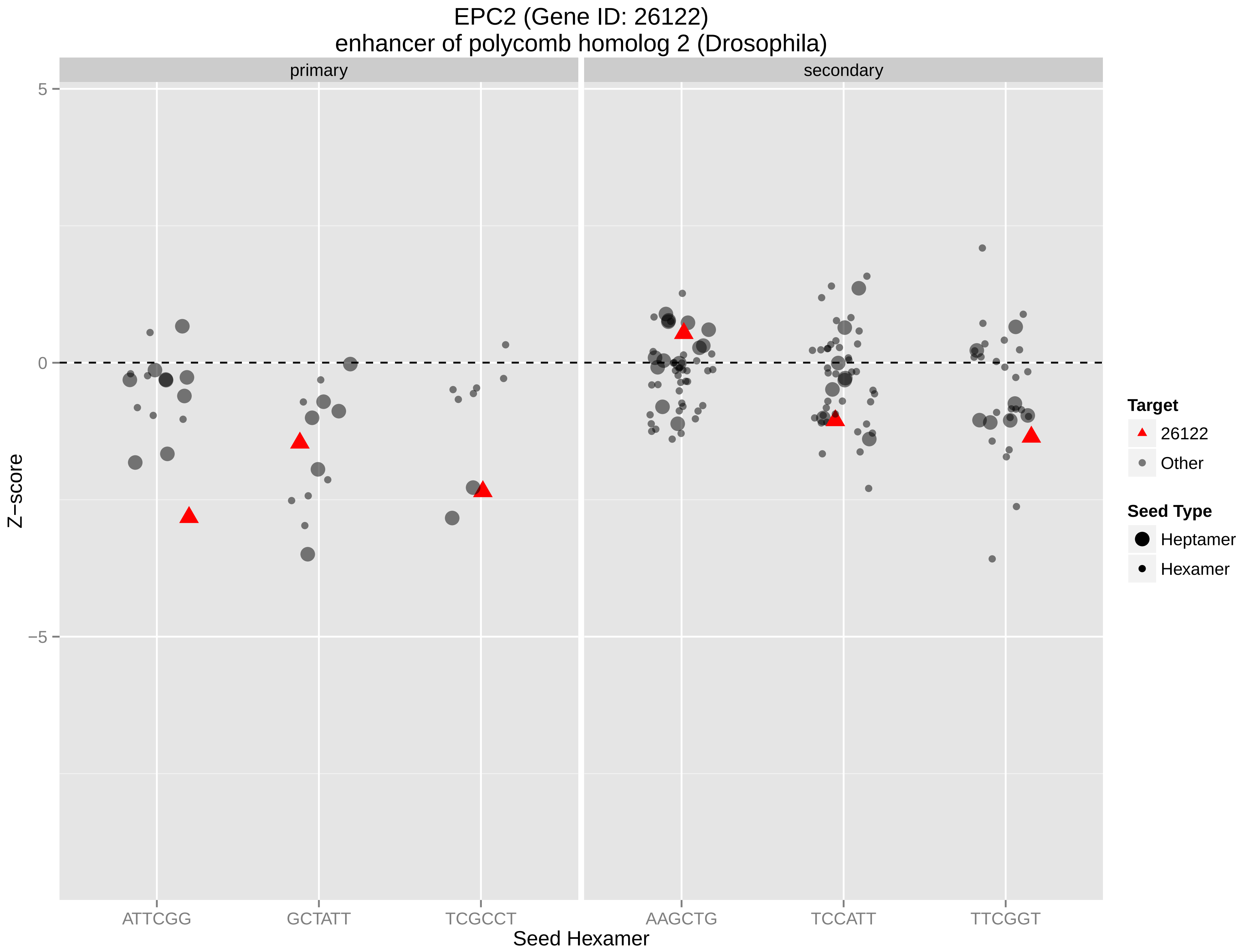

EPC2 (Gene ID: 26122)
enhancer of polycomb homolog 2 (Drosophila)
primary
secondary
5
0
−5
Z−score
ATTCGG
GCTATT
TCGCCT
AAGCTG
TCCATT
TTCGGT
Seed Hexamer
Target
26122
Other
Seed Type
Heptamer
Hexamer

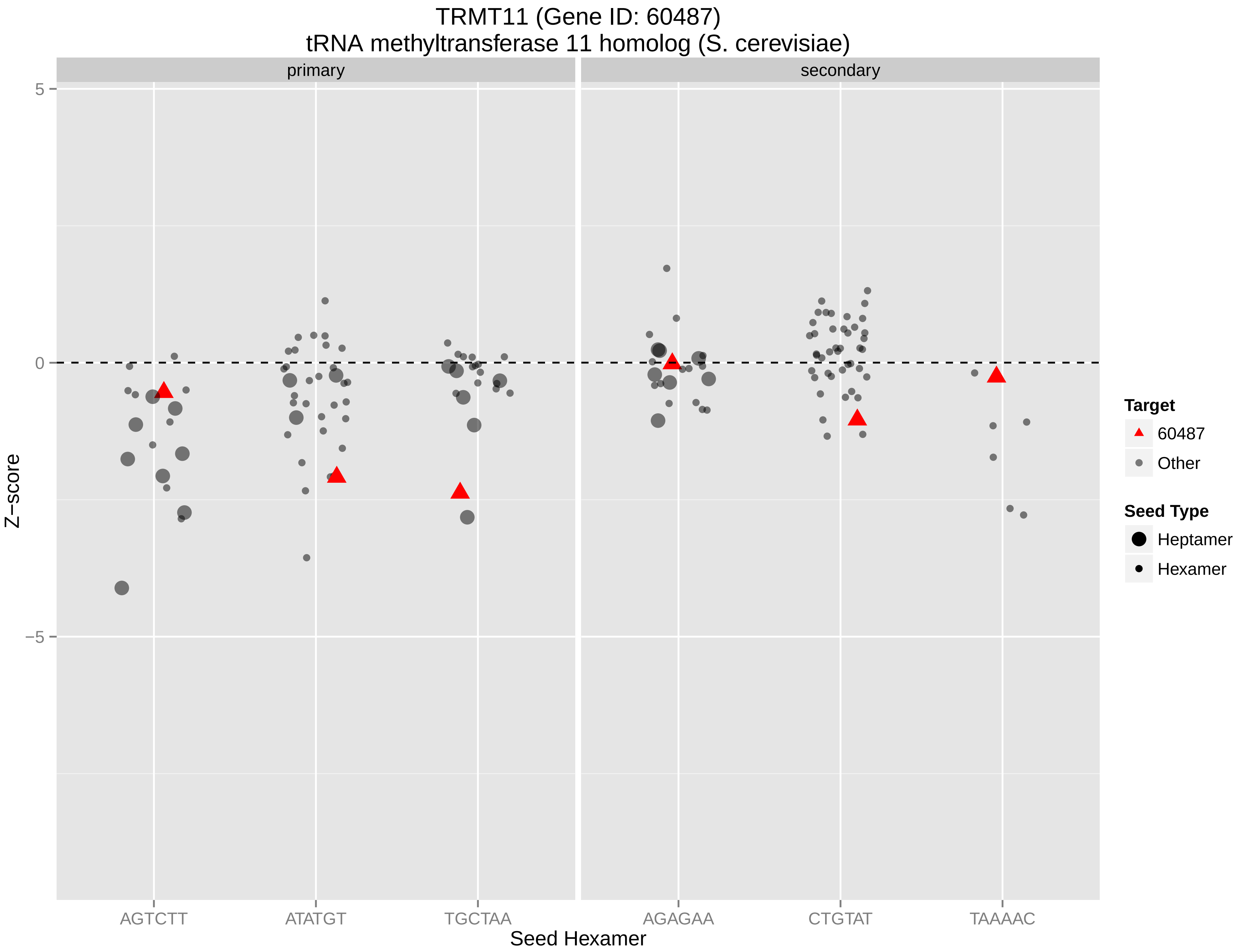
TRMT11 (Gene ID: 60487)
tRNA methyltransferase 11 homolog (S. cerevisiae)
primary
secondary
5
0
−5
Z−score
AGTCTT
ATATGT
TGCTAA
AGAGAA
CTGTAT
TAAAAC
Seed Hexamer
Target
60487
Other
Seed Type
Heptamer
Hexamer

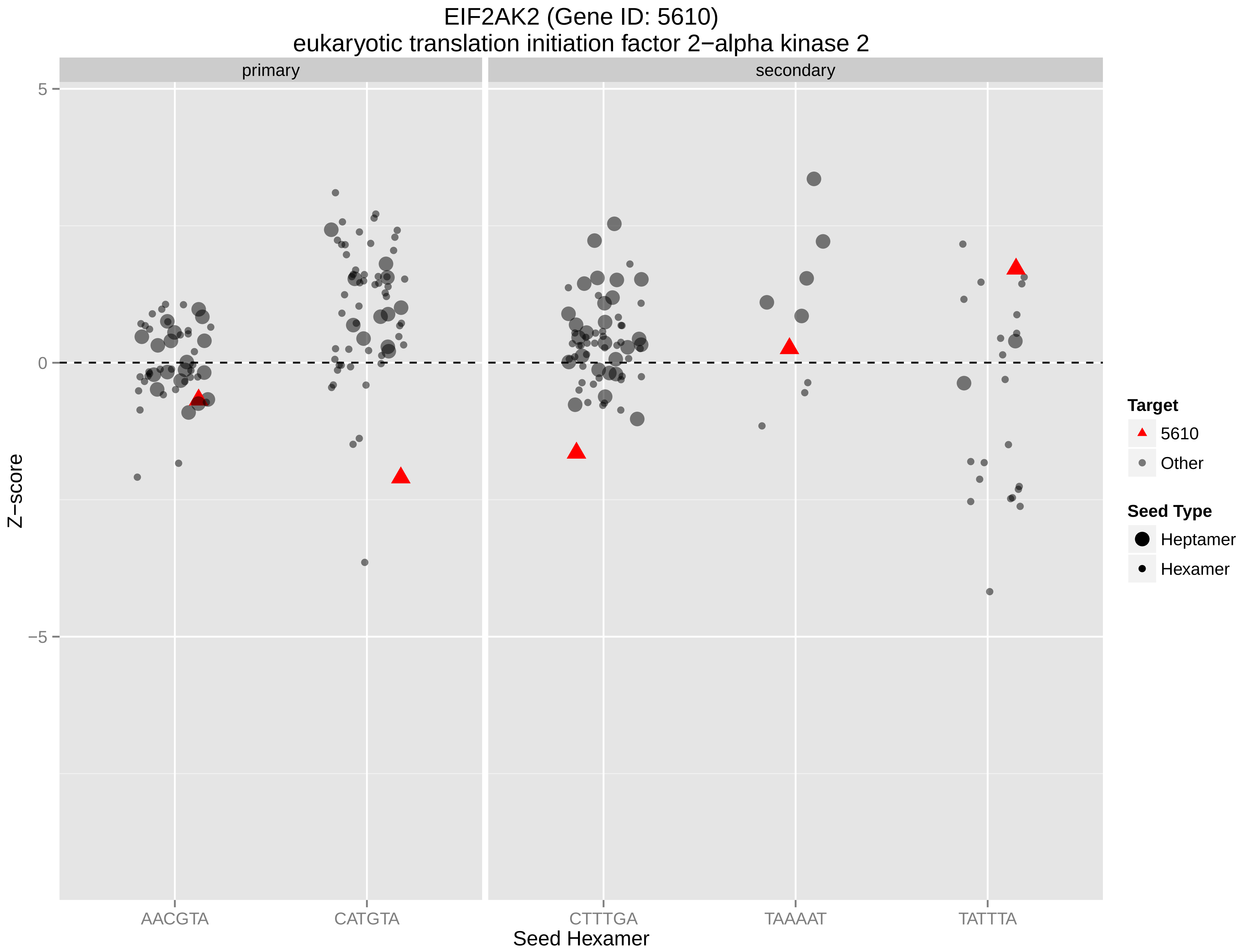

EIF2AK2 (Gene ID: 5610)
eukaryotic translation initiation factor 2−alpha kinase 2
primary
secondary
5
0
−5
Z−score
AACGTA
CATGTA
CTTTGA
TAAAAT
TATTTA
Seed Hexamer
Target
5610
Other
Seed Type
Heptamer
Hexamer

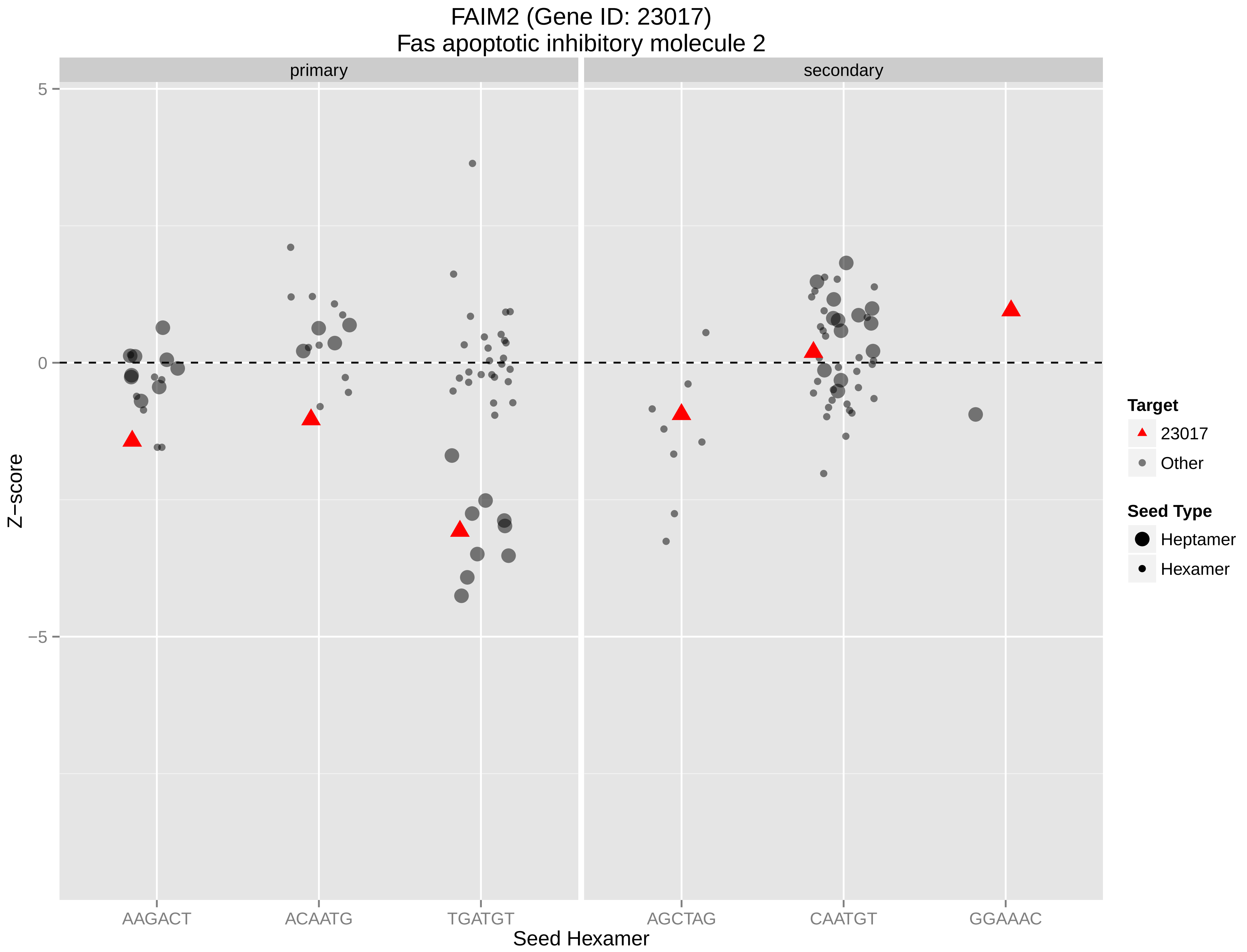

FAIM2 (Gene ID: 23017)
Fas apoptotic inhibitory molecule 2
primary
secondary
5
0
−5
Z−score
AAGACT
ACAATG
TGATGT
AGCTAG
CAATGT
GGAAAC
Seed Hexamer
Target
23017
Other
Seed Type
Heptamer
Hexamer

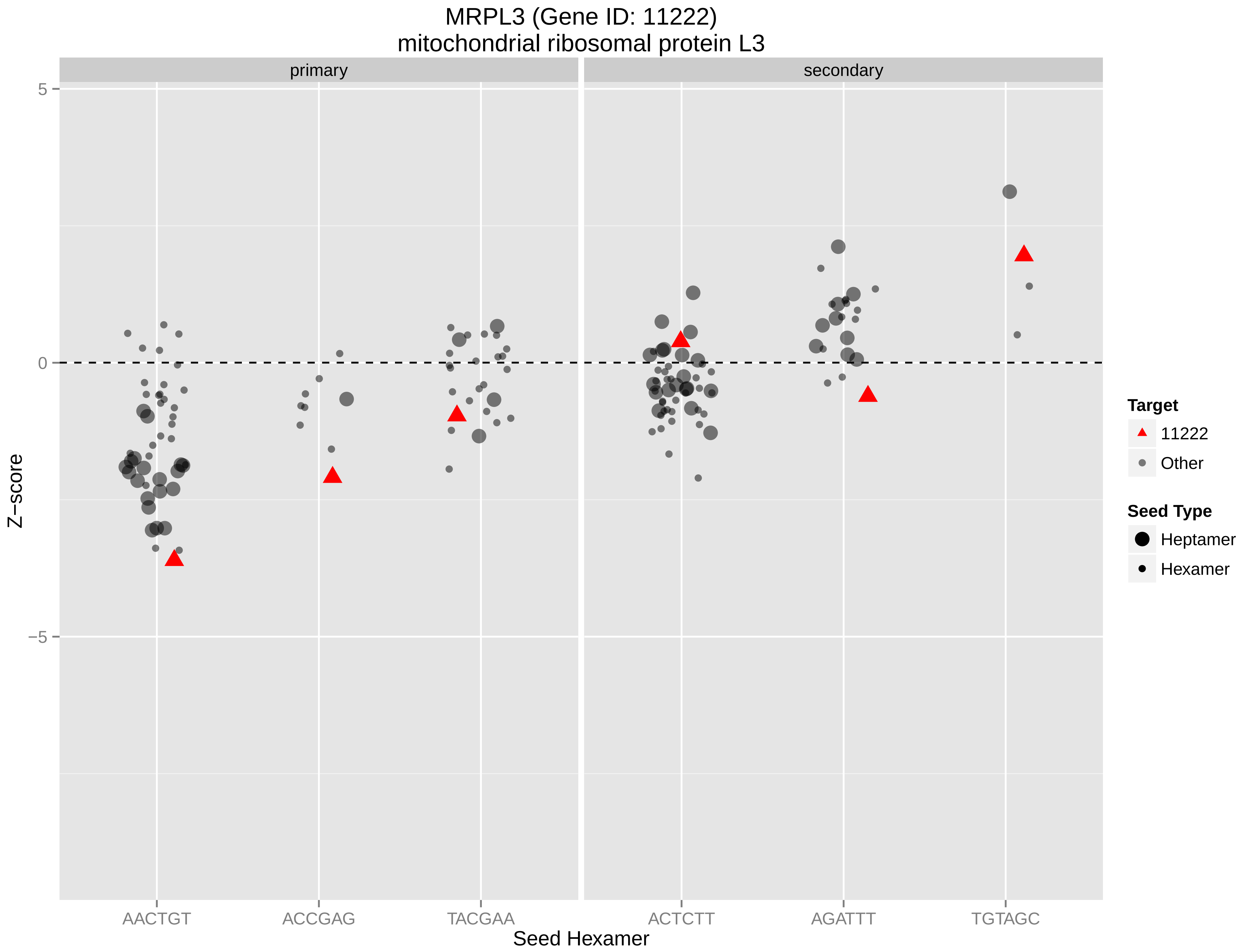
MRPL3 (Gene ID: 11222)
mitochondrial ribosomal protein L3
primary
secondary
5
0
−5
Z−score
AACTGT
ACCGAG
TACGAA
ACTCTT
AGATTT
TGTAGC
Seed Hexamer
Target
11222
Other
Seed Type
Heptamer
Hexamer

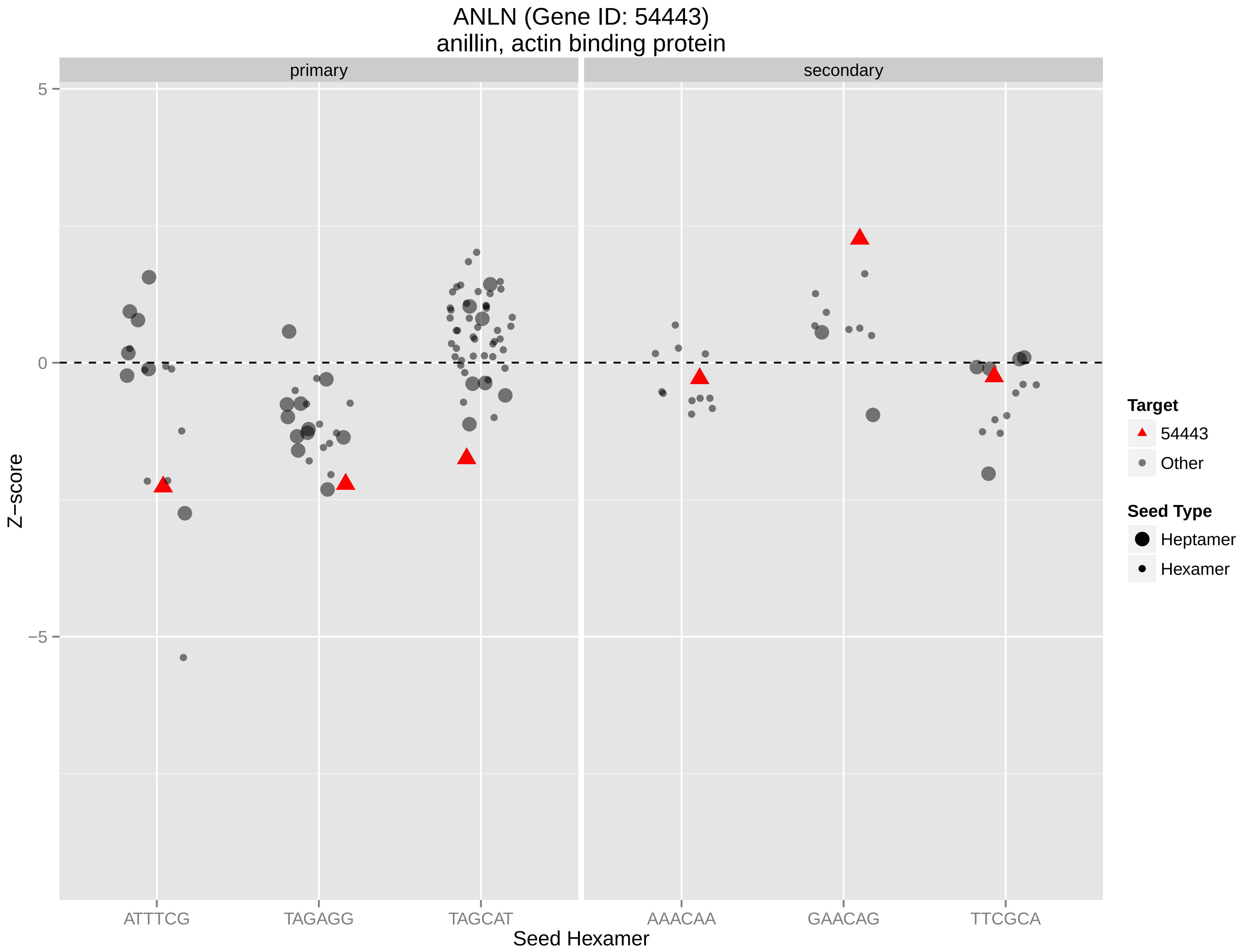

ANLN (Gene ID: 54443)
anillin, actin binding protein
primary
secondary
5
0
−5
Z−score
ATTTCG
TAGAGG
TAGCAT
AAACAA
GAACAG
TTCGCA
Seed Hexamer
Target
54443
Other
Seed Type
Heptamer
Hexamer

TCEB2 (Gene ID: 6923)
transcription elongation factor B (SIII), polypeptide 2 (18kDa, elongin B)

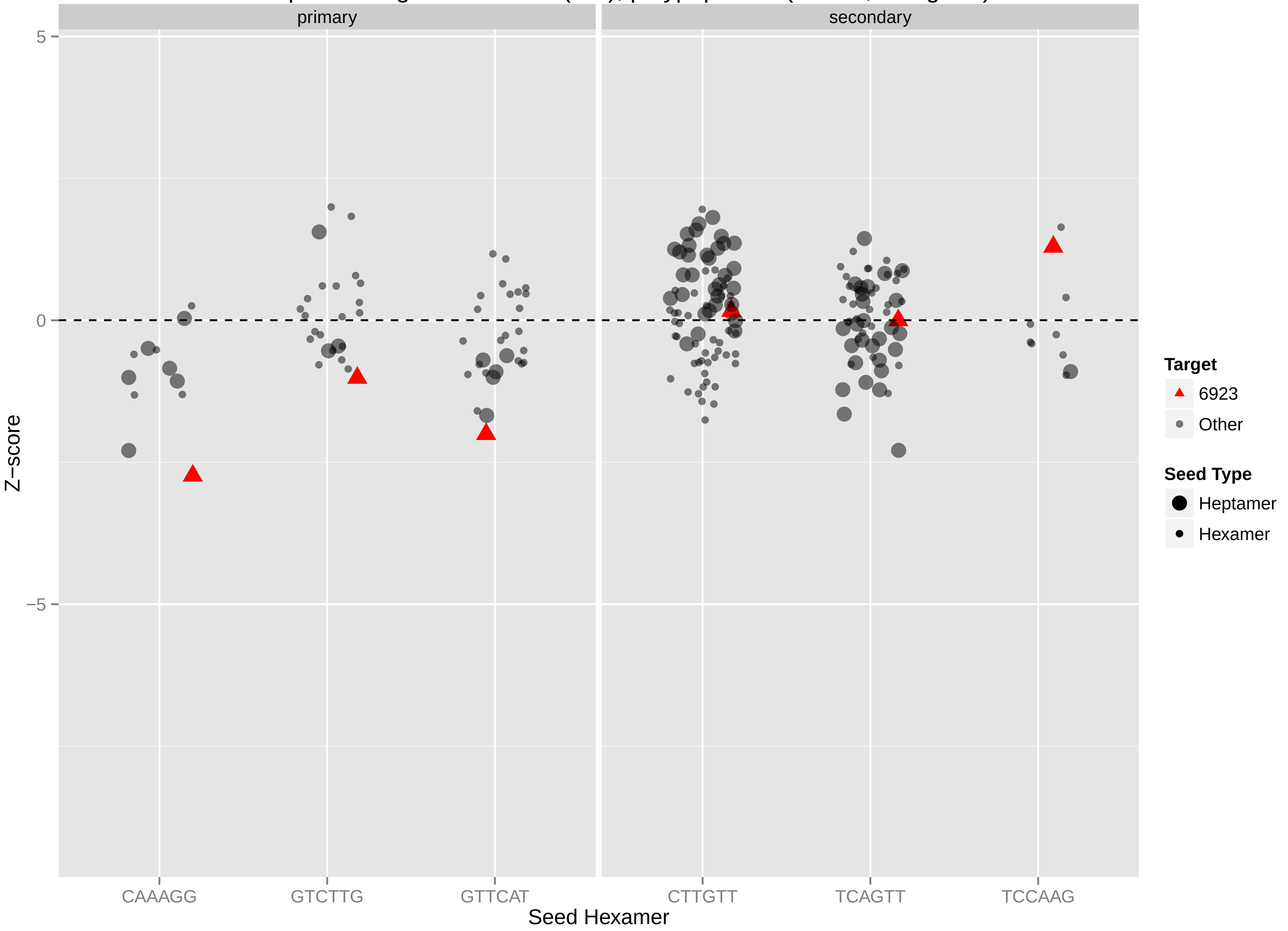

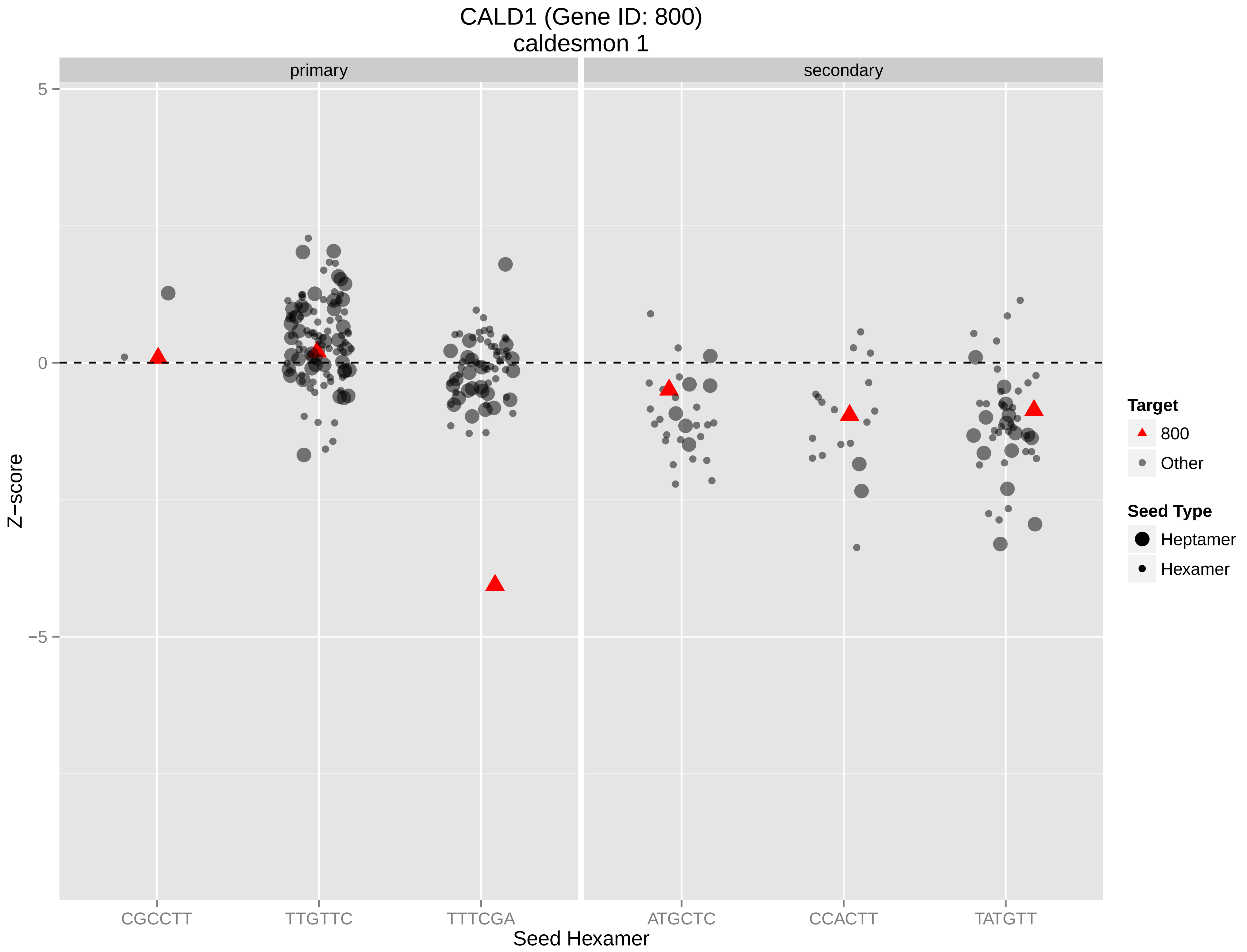

CALD1 (Gene ID: 800)
caldesmon 1
primary
secondary
5
0
−5
Z−score
CGCCTT
TTGTTC
TTTCGA
ATGCTC
CCACTT
TATGTT
Seed Hexamer
Target
800
Other
Seed Type
Heptamer
Hexamer

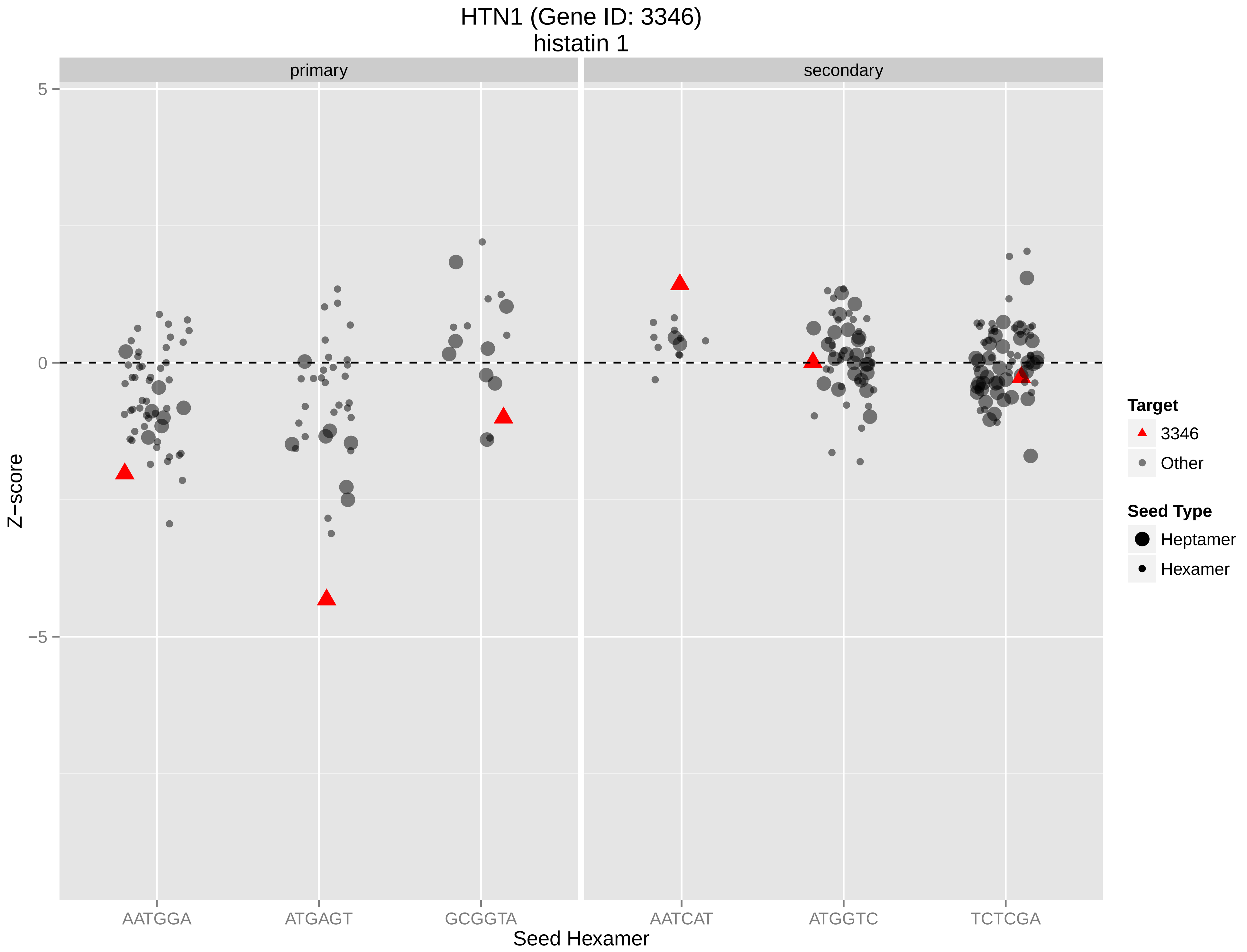

HTN1 (Gene ID: 3346)
histatin 1
primary
secondary
5
0
−5
Z−score
AATGGA
ATGAGT
GCGGTA
AATCAT
ATGGTC
TCTCGA
Seed Hexamer
Target
3346
Other
Seed Type
Heptamer
Hexamer

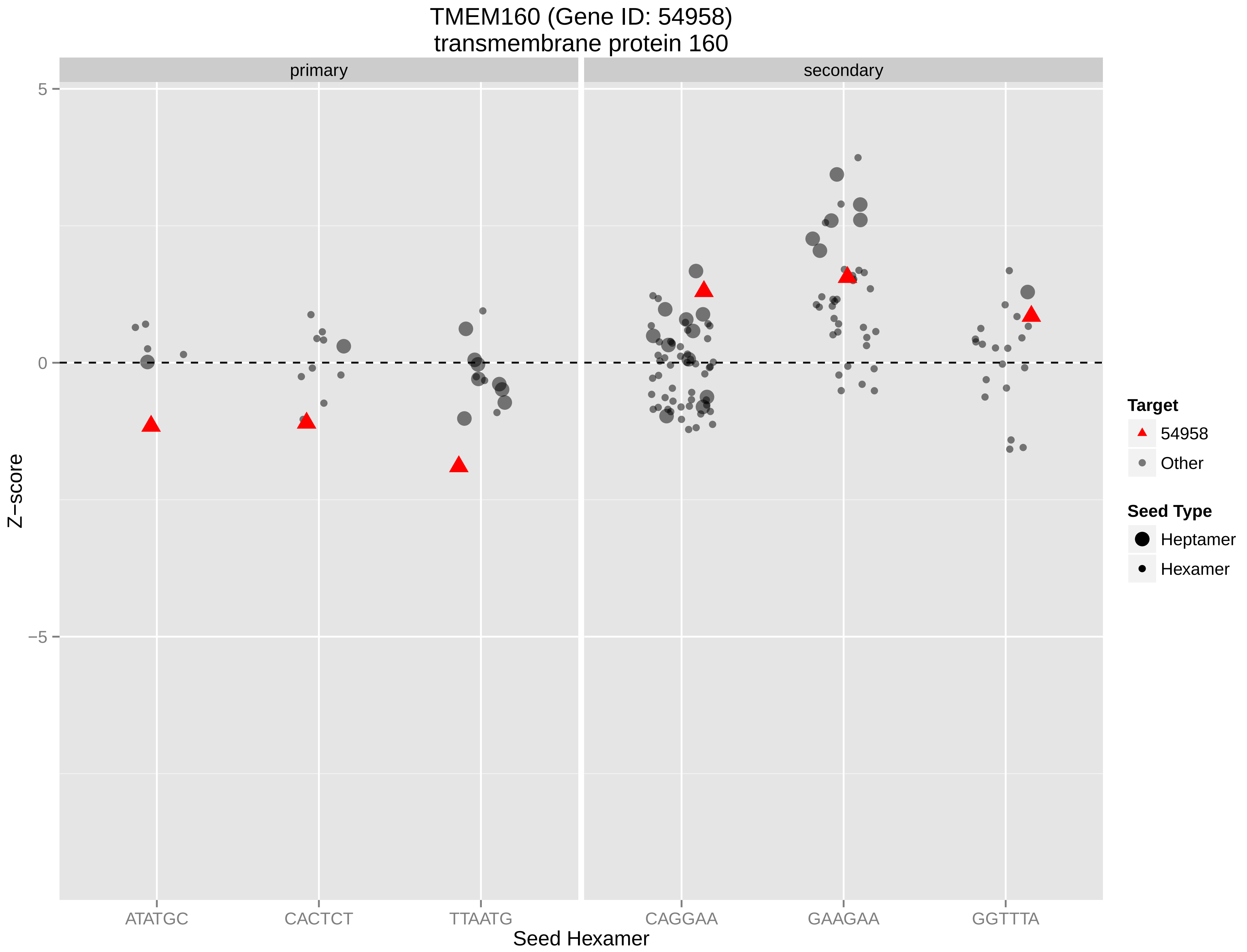

TMEM160 (Gene ID: 54958)
transmembrane protein 160
primary
secondary
5
0
−5
Z−score
ATATGC
CACTCT
TTAATG
CAGGAA
GAAGAA
GGTTTA
Seed Hexamer
Target
54958
Other
Seed Type
Heptamer
Hexamer

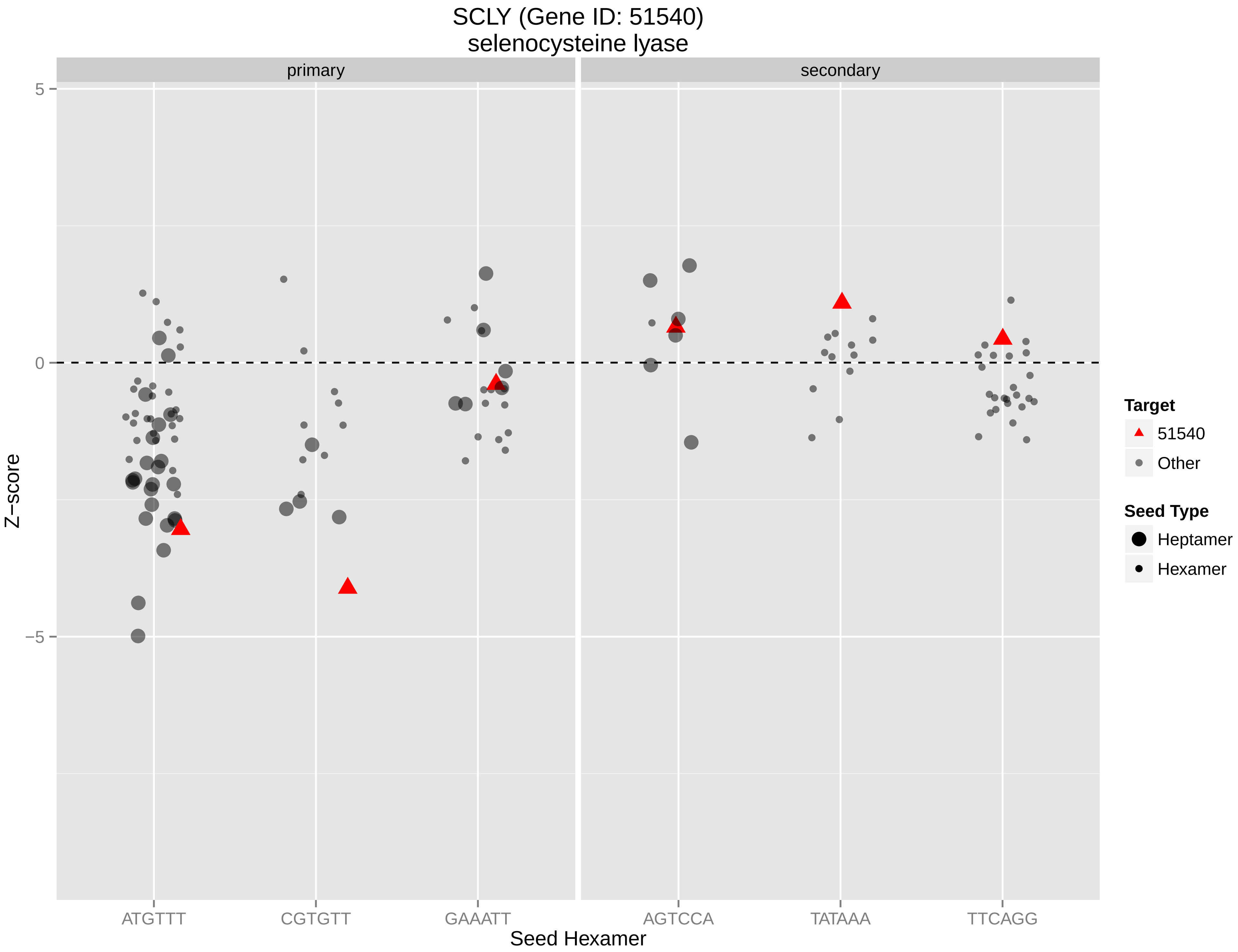
SCLY (Gene ID: 51540)
selenocysteine lyase
primary
secondary
5
0
−5
Z−score
ATGTTT
CGTGTT
GAAATT
AGTCCA
TATAAA
TTCAGG
Seed Hexamer
Target
51540
Other
Seed Type
Heptamer
Hexamer

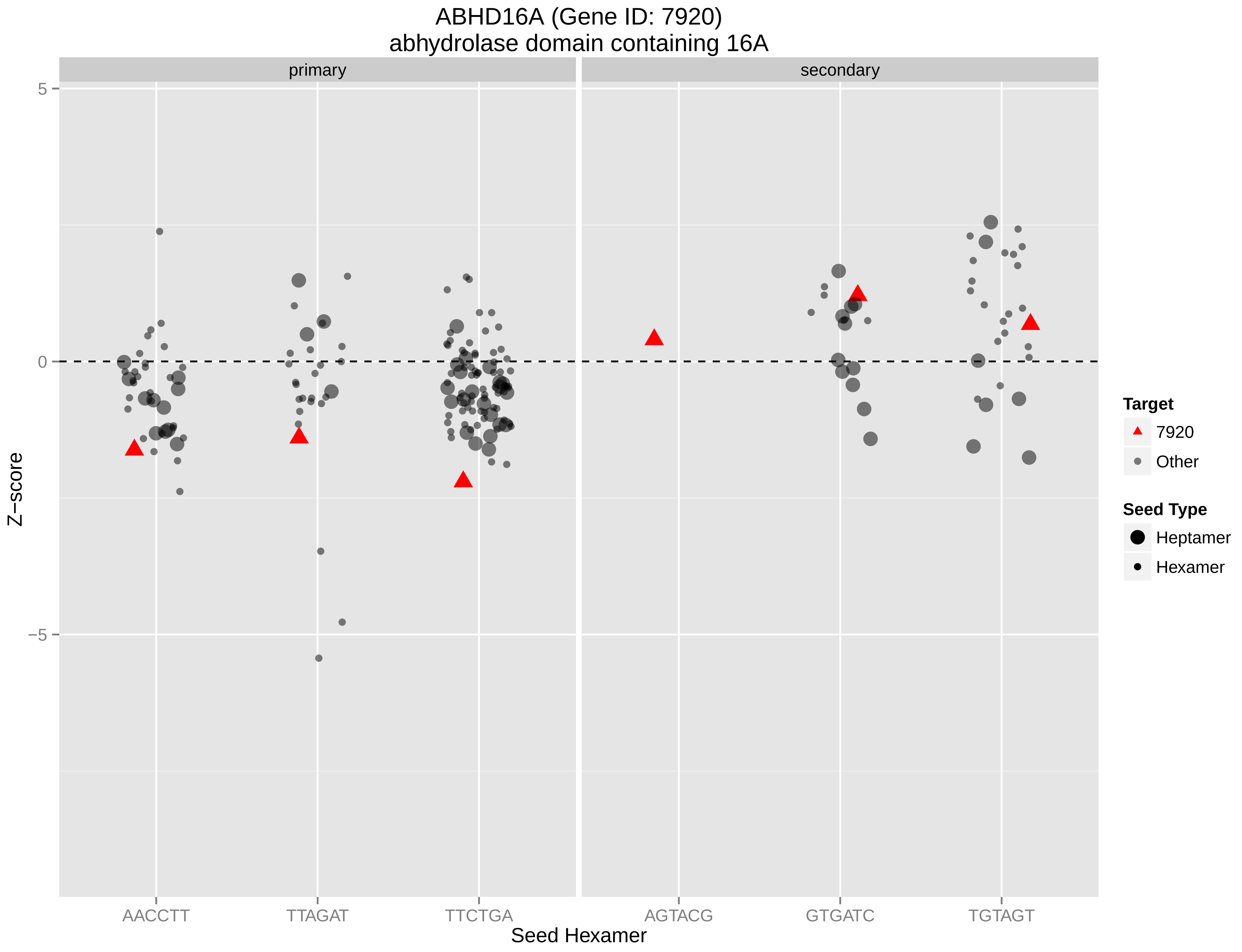
ABHD16A (Gene ID: 7920)
abhydrolase domain containing 16A
primary
secondary
5
0
−5
Z−score
AACCTT
TTAGAT
TTCTGA
AGTACG
GTGATC
TGTAGT
Seed Hexamer
Target
7920
Other
Seed Type
Heptamer
Hexamer

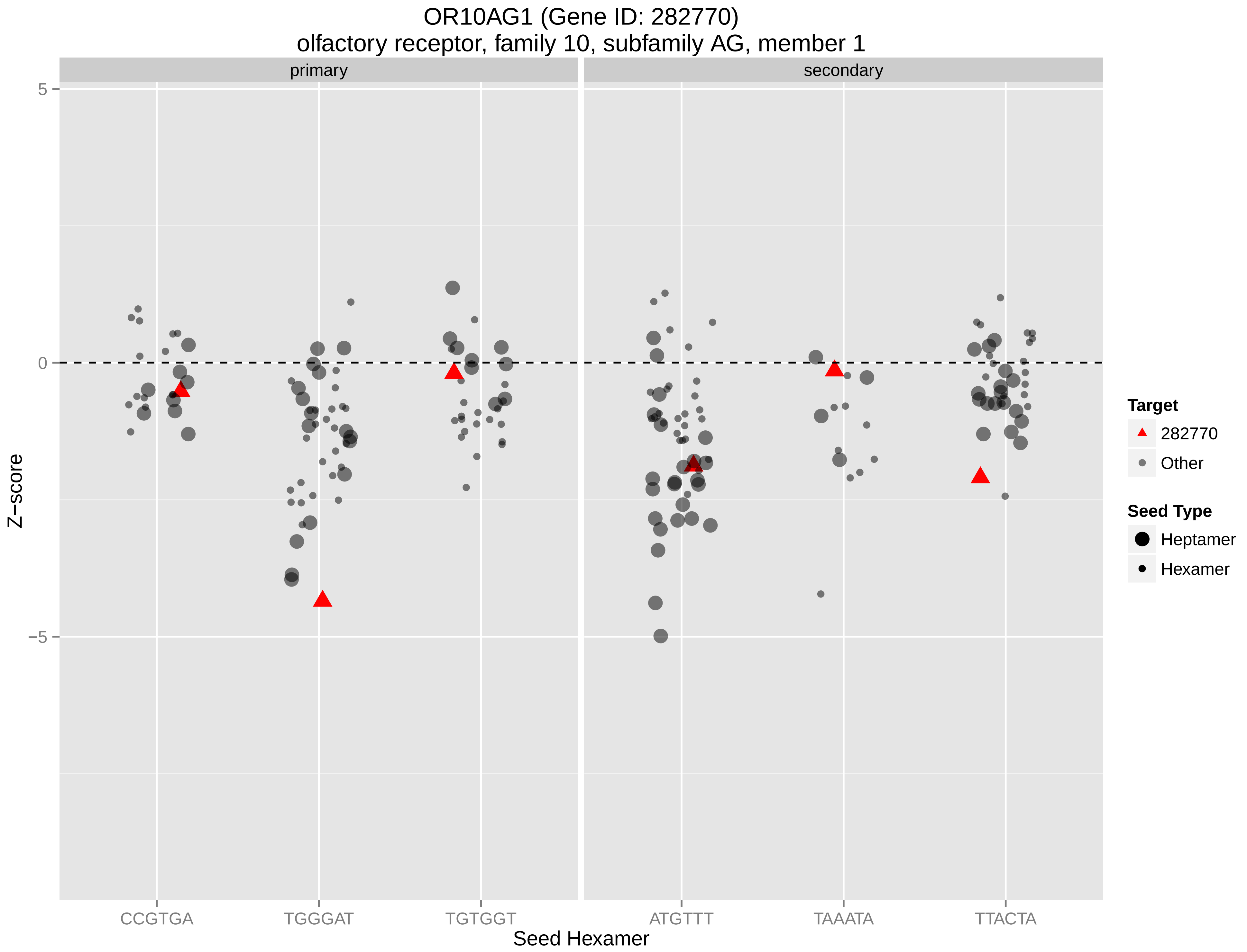
OR10AG1 (Gene ID: 282770)
olfactory receptor, family 10, subfamily AG, member 1
primary
secondary
5
0
−5
Z−score
CCGTGA
TGGGAT
TGTGGT
ATGTTT
TAAATA
TTACTA
Seed Hexamer
Target
282770
Other
Seed Type
Heptamer
Hexamer

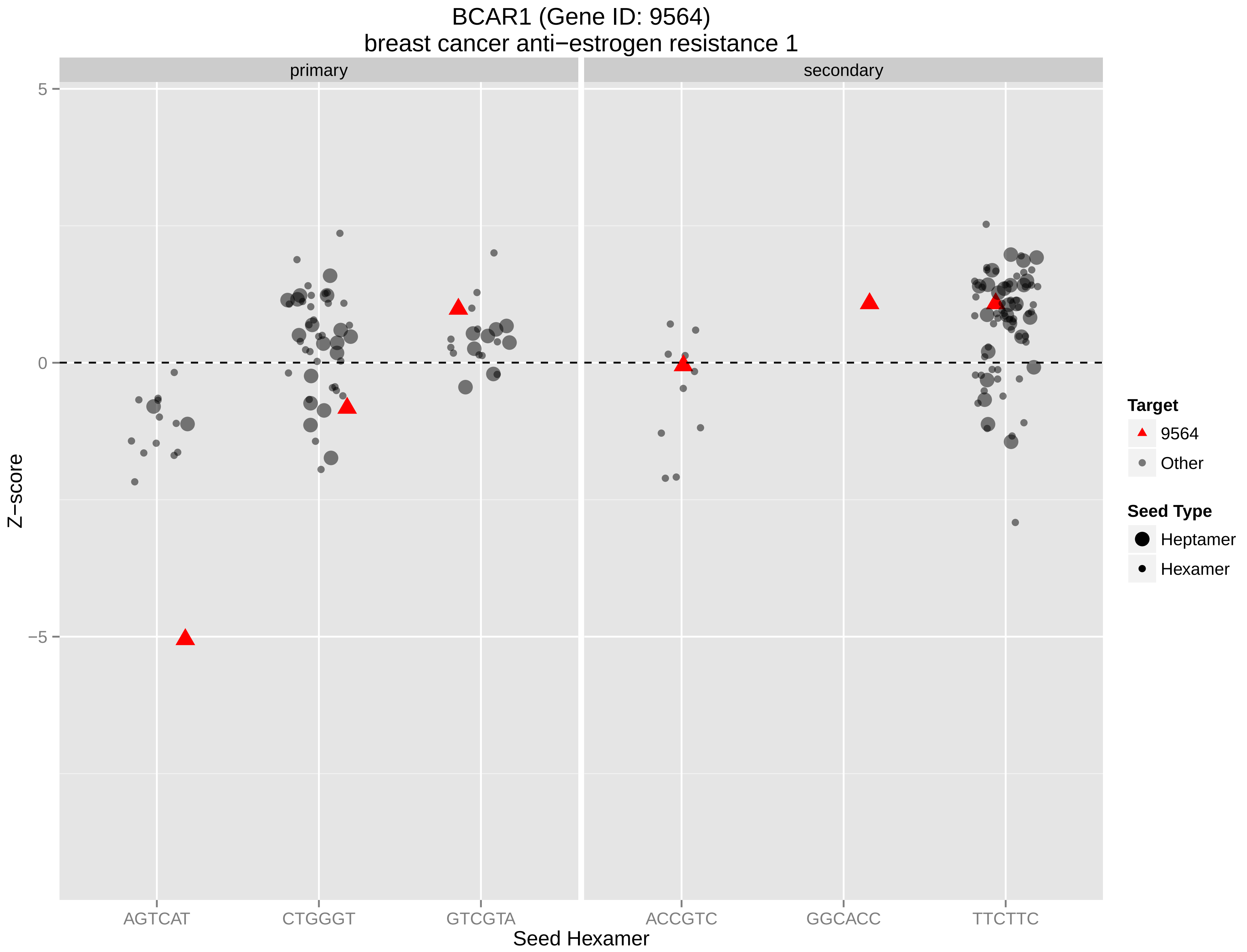

BCAR1 (Gene ID: 9564)
breast cancer anti−estrogen resistance 1
primary
secondary
5
0
−5
Z−score
AGTCAT
CTGGGT
GTCGTA
ACCGTC
GGCACC
TTCTTC
Seed Hexamer
Target
9564
Other
Seed Type
Heptamer
Hexamer

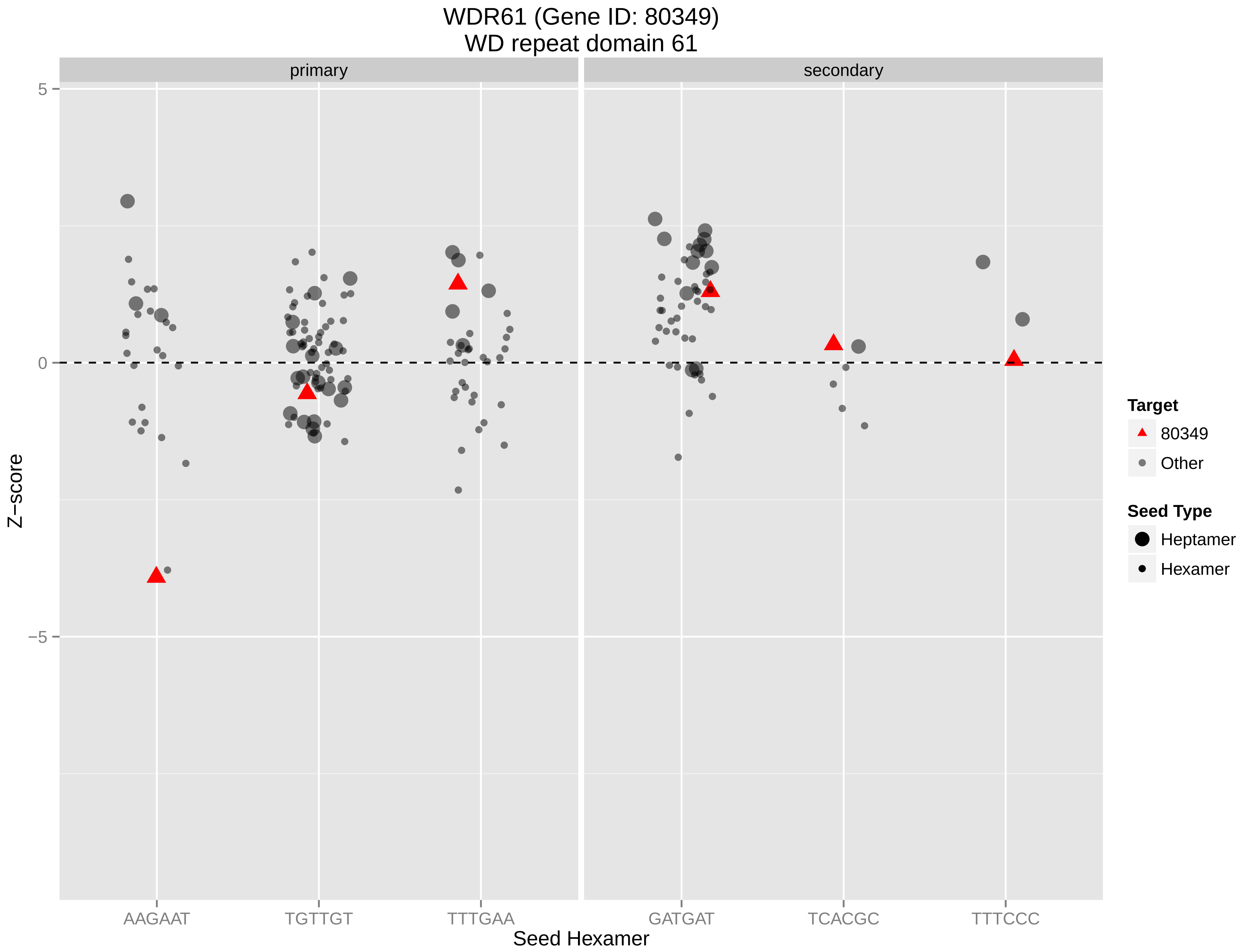
WDR61 (Gene ID: 80349)
WD repeat domain 61
primary
secondary
5
0
−5
Z−score
AAGAAT
TGTTGT
TTTGAA
GATGAT
TCACGC
TTTCCC
Seed Hexamer
Target
80349
Other
Seed Type
Heptamer
Hexamer

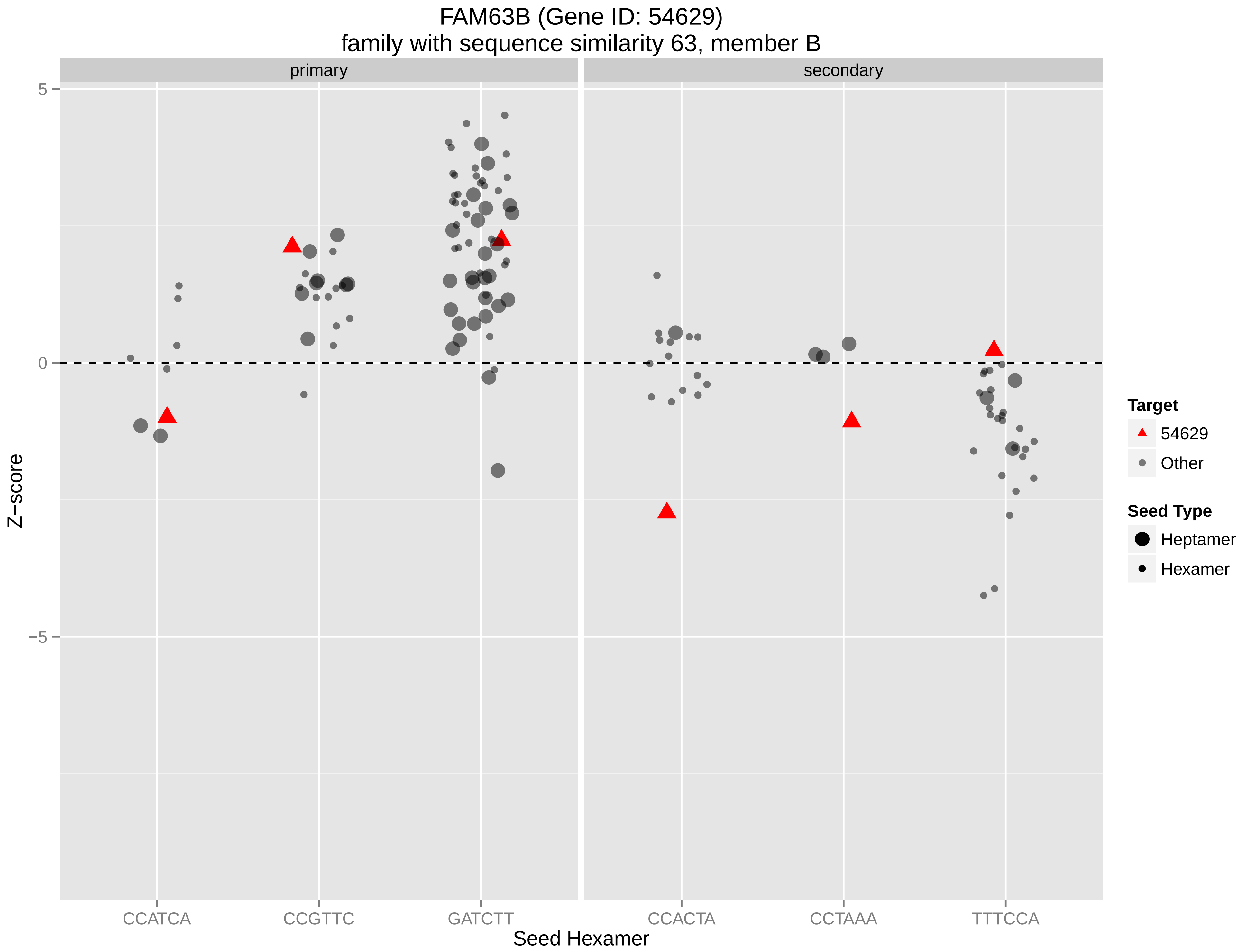

FAM63B (Gene ID: 54629)
family with sequence similarity 63, member B
primary
secondary
5
0
−5
Z−score
CCATCA
CCGTTC
GATCTT
CCACTA
CCTAAA
TTTCCA
Seed Hexamer
Target
54629
Other
Seed Type
Heptamer
Hexamer

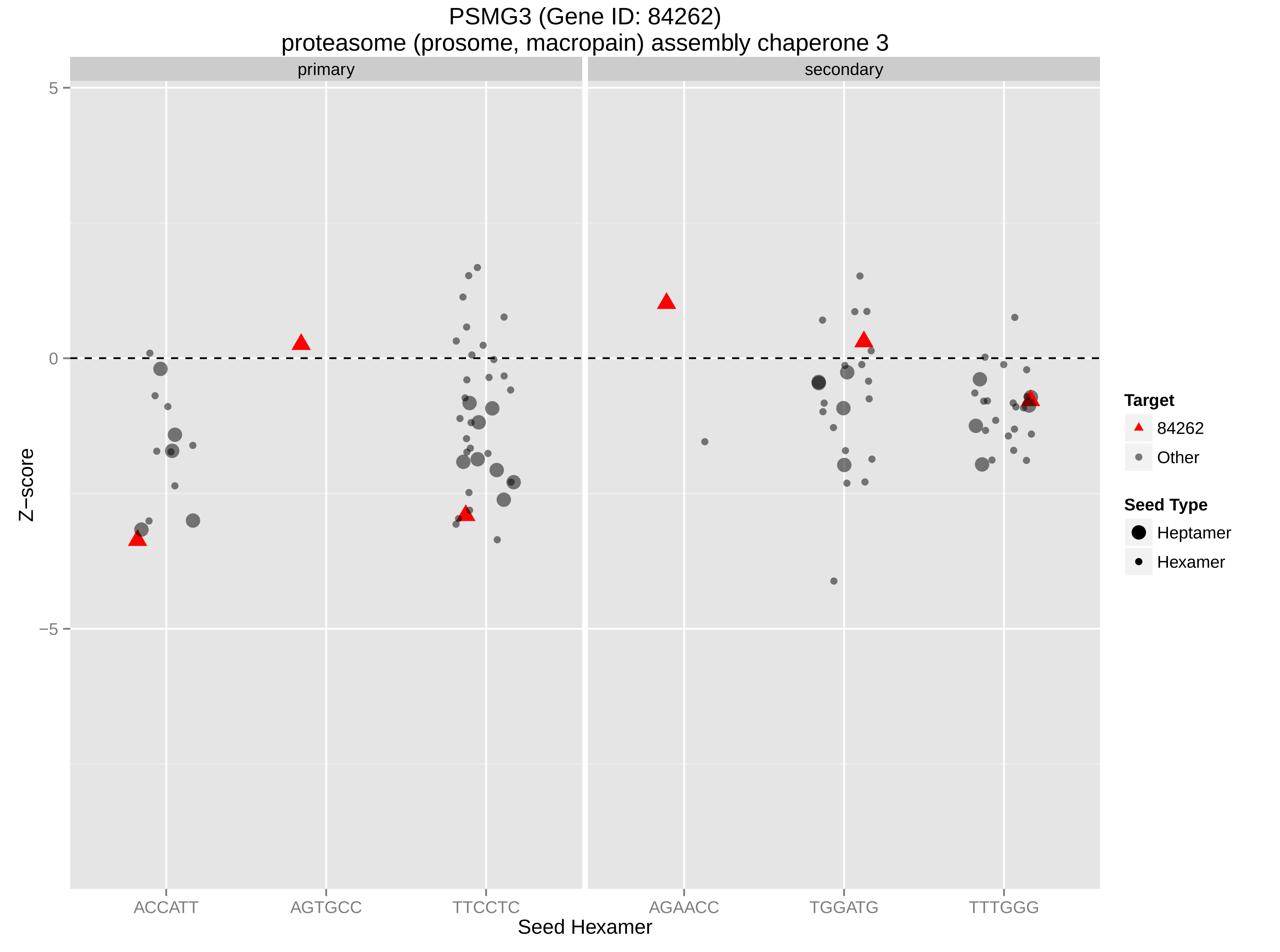

PSMG3 (Gene ID: 84262)
proteasome (prosome, macropain) assembly chaperone 3
primary
secondary
5
0
−5
Z−score
ACCATT
AGTGCC
TTCCTC
AGAACC
TGGATG
TTTGGG
Seed Hexamer
Target
84262
Other
Seed Type
Heptamer
Hexamer

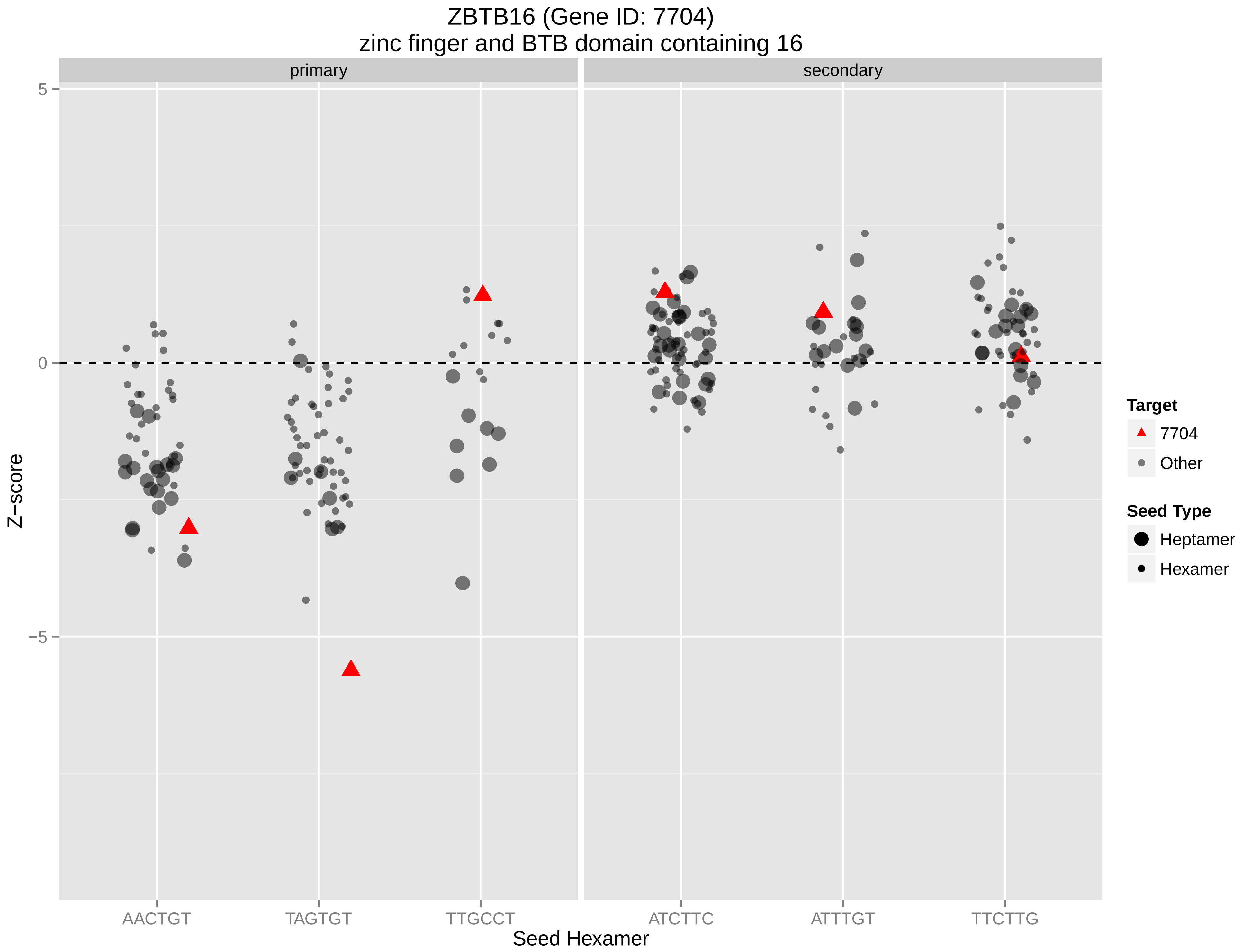

ZBTB16 (Gene ID: 7704)
zinc finger and BTB domain containing 16
primary
secondary
5
0
−5
Z−score
AACTGT
TAGTGT
TTGCCT
ATCTTC
ATTTGT
TTCTTG
Seed Hexamer
Target
7704
Other
Seed Type
Heptamer
Hexamer

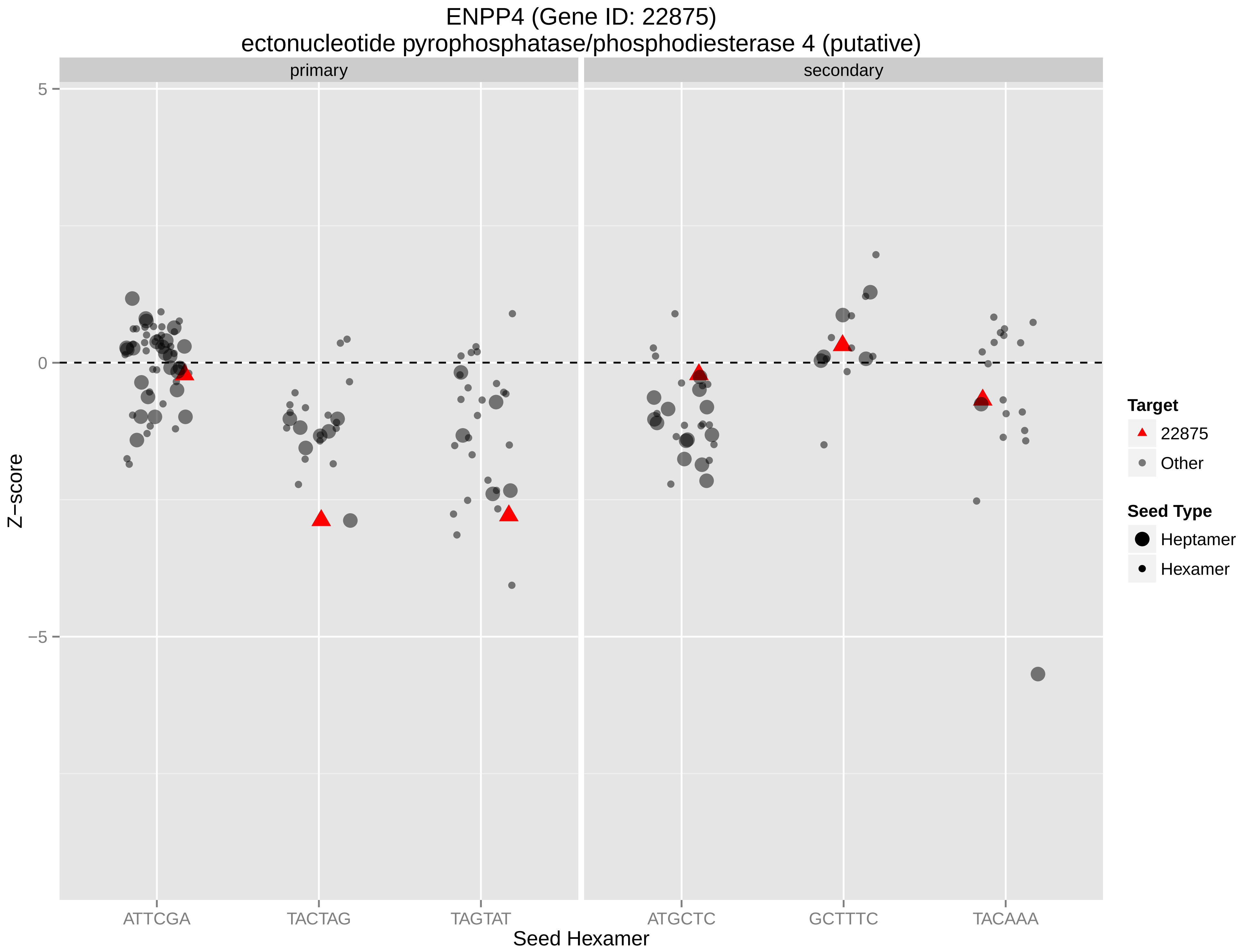

ENPP4 (Gene ID: 22875)
ectonucleotide pyrophosphatase/phosphodiesterase 4 (putative)
primary
secondary
5
0
−5
Z−score
ATTCGA
TACTAG
TAGTAT
ATGCTC
GCTTTC
TACAAA
Seed Hexamer
Target
22875
Other
Seed Type
Heptamer
Hexamer

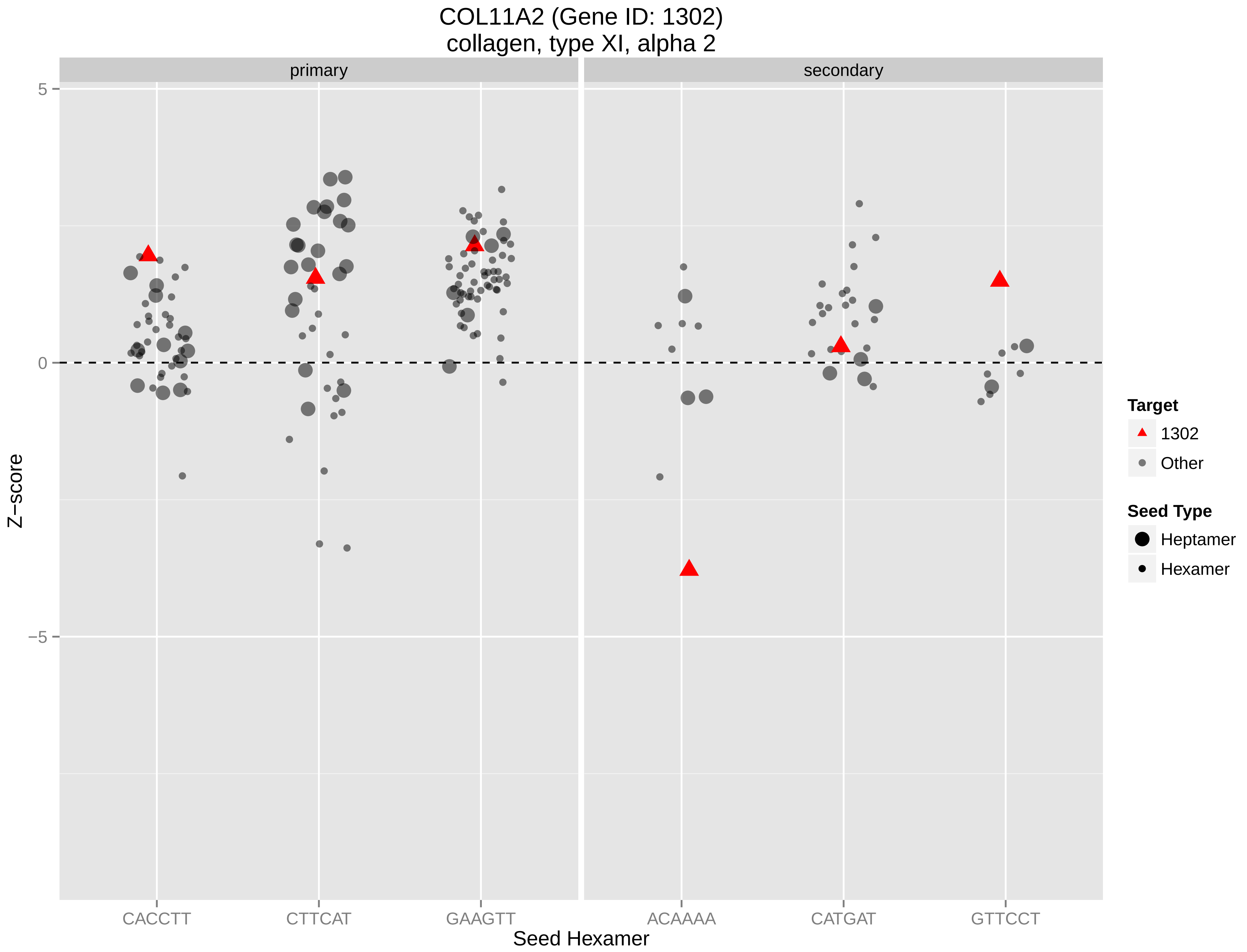
COL11A2 (Gene ID: 1302)
collagen, type XI, alpha 2
primary
secondary
5
0
−5
Z−score
CACCTT
CTTCAT
GAAGTT
ACAAAA
CATGAT
GTTCCT
Seed Hexamer
Target
1302
Other
Seed Type
Heptamer
Hexamer

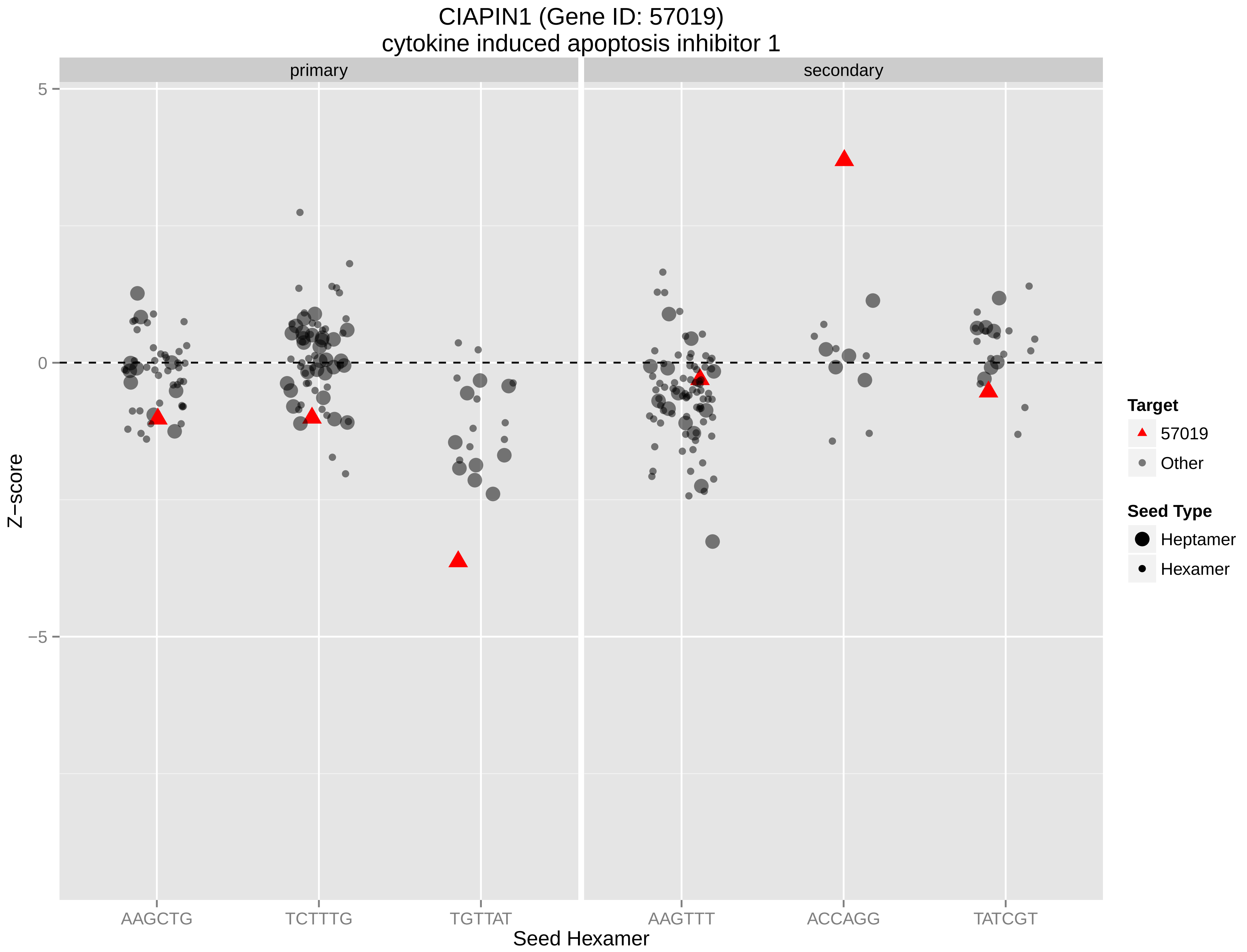

CIAPIN1 (Gene ID: 57019)
cytokine induced apoptosis inhibitor 1
primary
secondary
5
0
−5
Z−score
AAGCTG
TCTTTG
TGTTAT
AAGTTT
ACCAGG
TATCGT
Seed Hexamer
Target
57019
Other
Seed Type
Heptamer
Hexamer

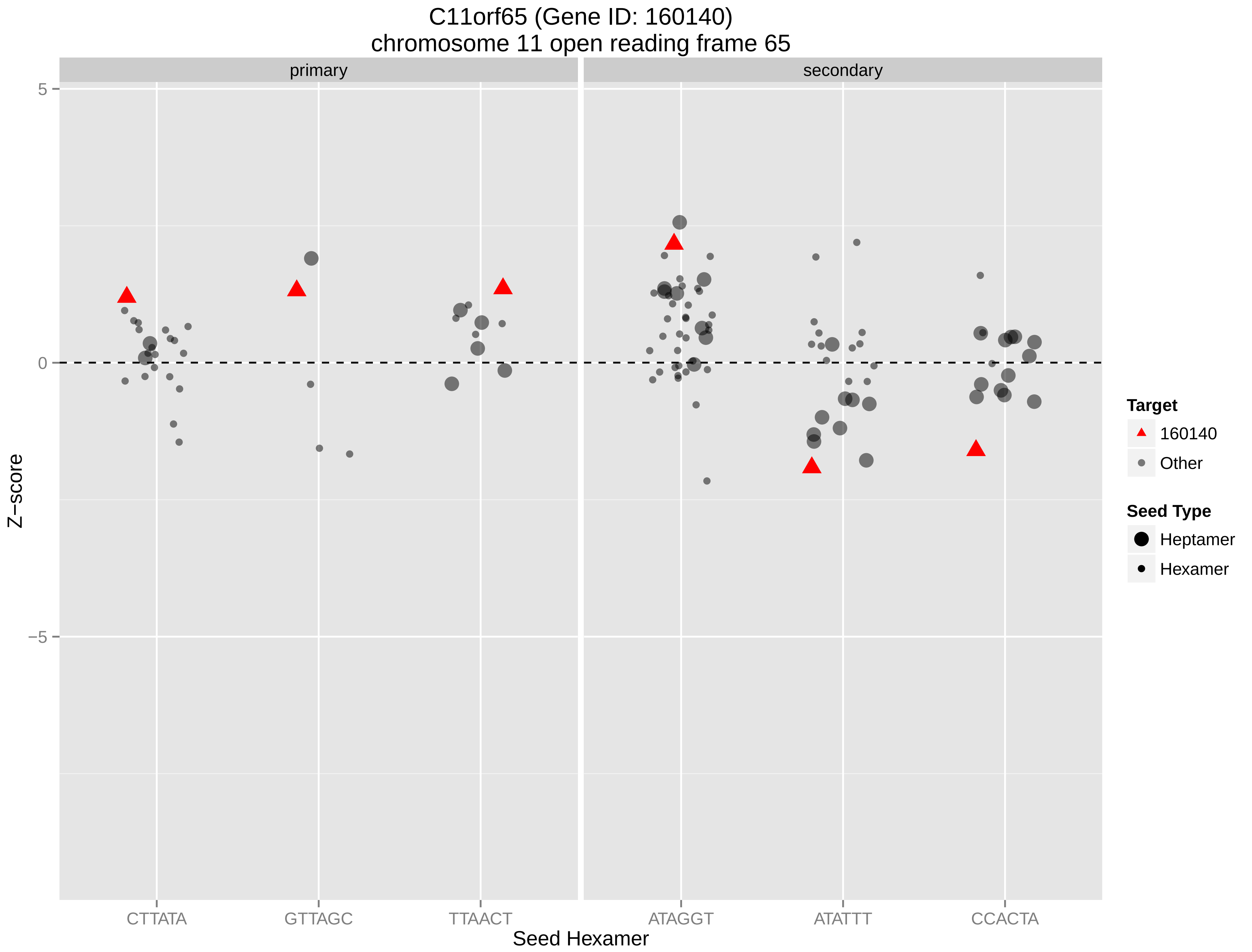

C11orf65 (Gene ID: 160140)
chromosome 11 open reading frame 65
primary
secondary
Z−score
5
0
−5
CTTATA
GTTAGC
TTAACT
ATAGGT
ATATTT
CCACTA
Seed Hexamer
Target
160140
Other
Seed Type
Heptamer
Hexamer

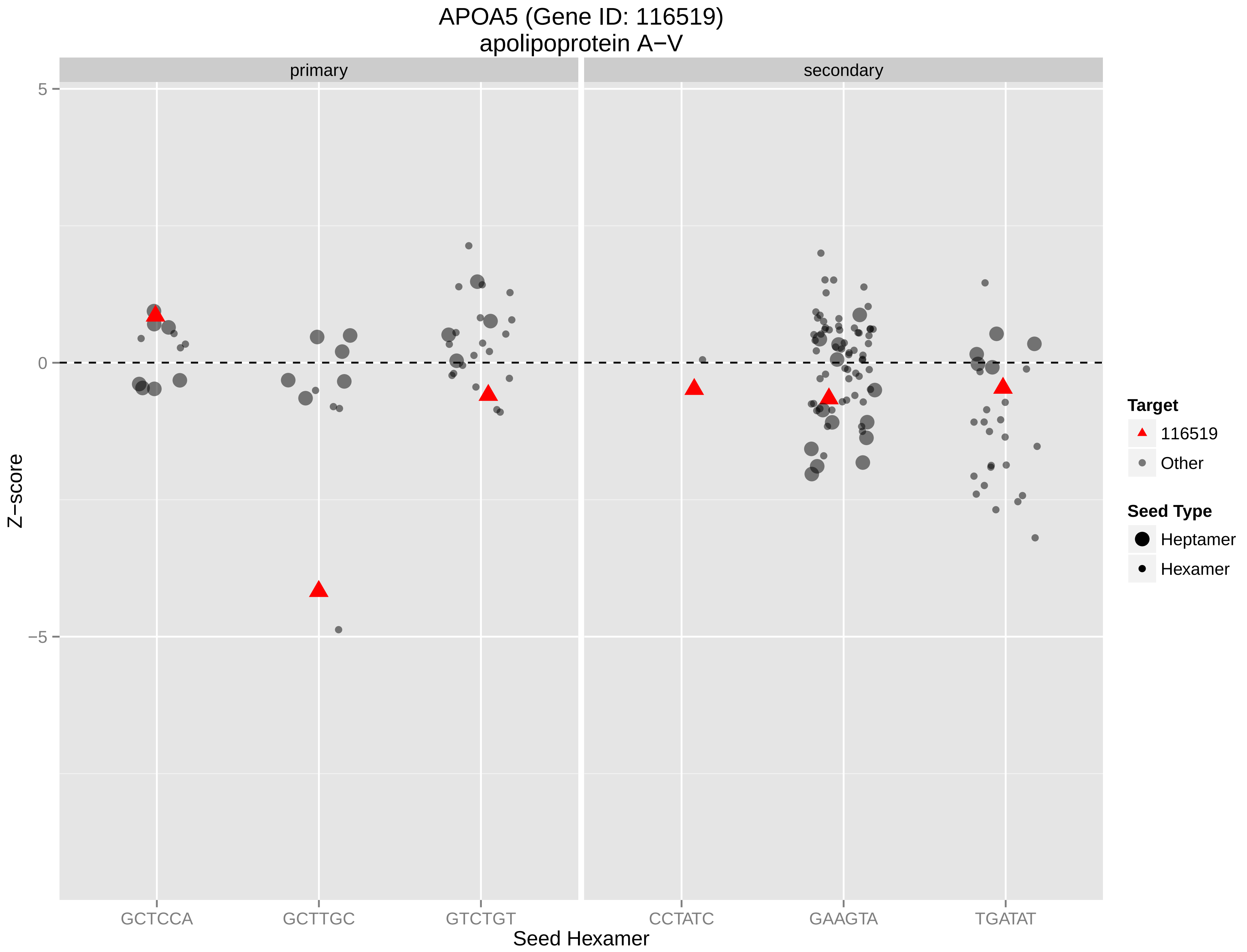

APOA5 (Gene ID: 116519)
apolipoprotein A−V
primary
secondary
5
0
−5
Z−score
GCTCCA
GCTTGC
GTCTGT
CCTATC
GAAGTA
TGATAT
Seed Hexamer
Target
116519
Other
Seed Type
Heptamer
Hexamer

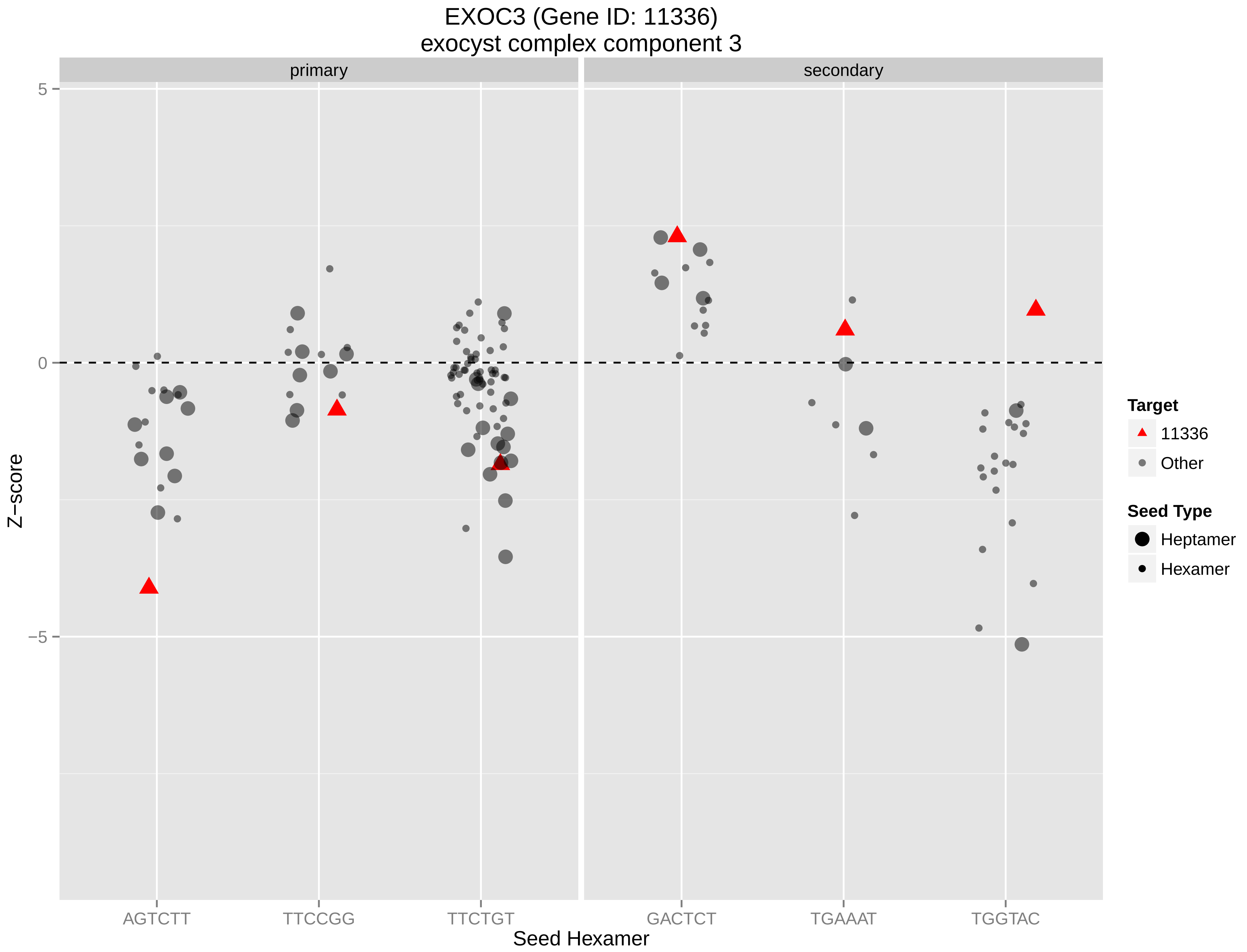
EXOC3 (Gene ID: 11336)
exocyst complex component 3
primary
secondary
5
0
−5
Z−score
AGTCTT
TTCCGG
TTCTGT
GACTCT
TGAAAT
TGGTAC
Seed Hexamer
Target
11336
Other
Seed Type
Heptamer
Hexamer

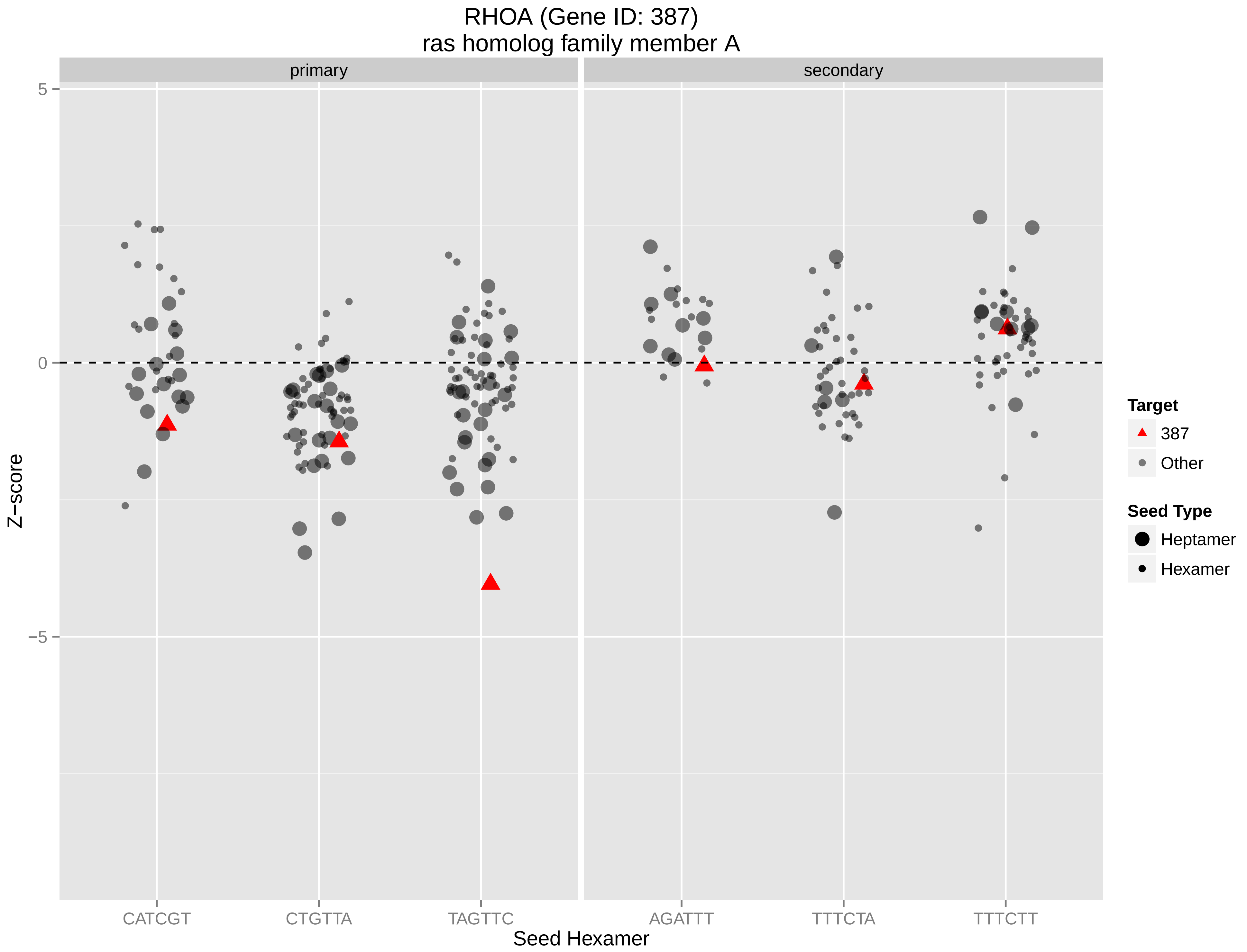
RHOA (Gene ID: 387)
ras homolog family member A
primary
secondary
5
0
−5
Z−score
CATCGT
CTGTTA
TAGTTC
AGATTT
TTTCTA
TTTCTT
Seed Hexamer
Target
387
Other
Seed Type
Heptamer
Hexamer

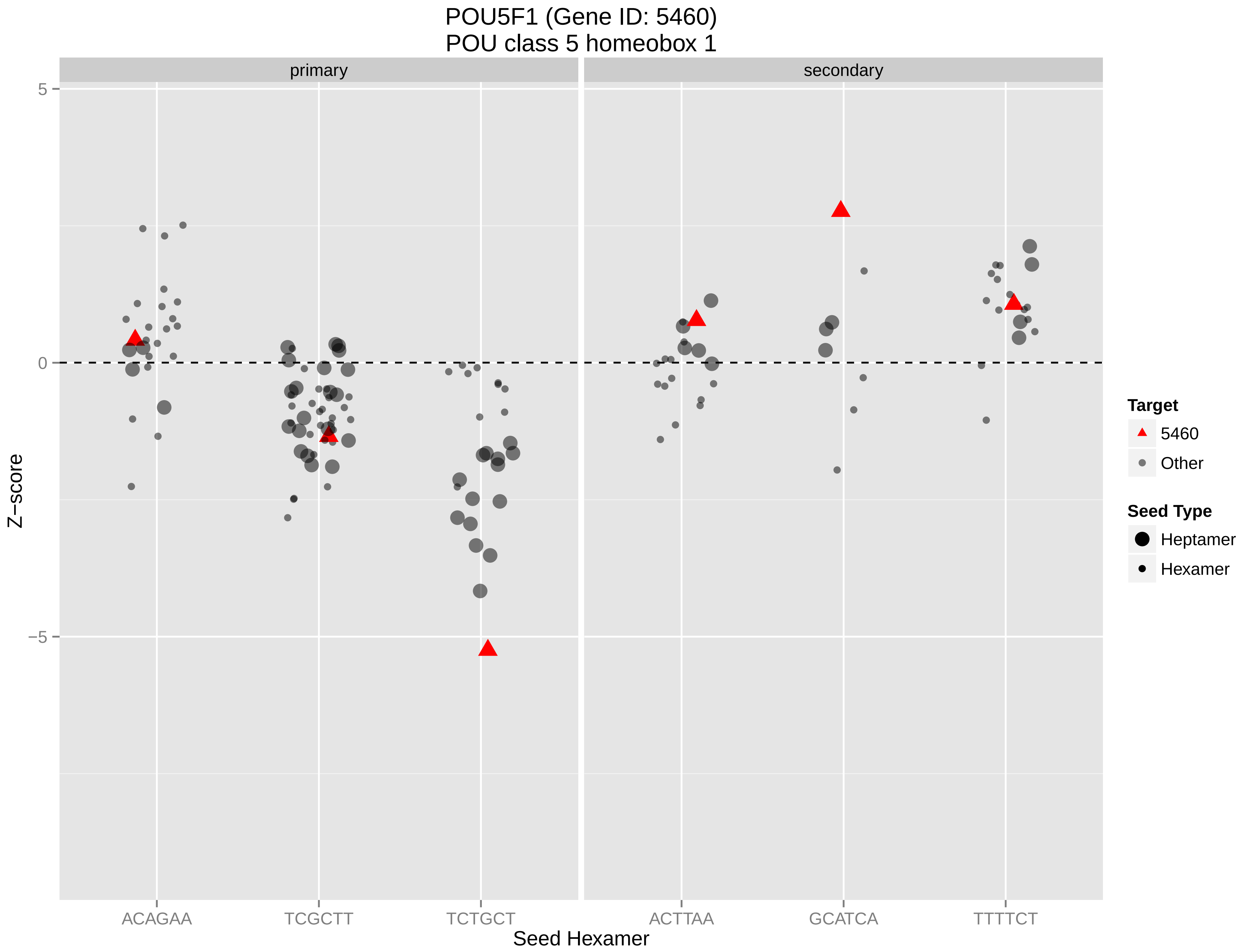

POU5F1 (Gene ID: 5460)
POU class 5 homeobox 1
primary
secondary
5
0
−5
Z−score
ACAGAA
TCGCTT
TCTGCT
ACTTAA
GCATCA
TTTTCT
Seed Hexamer
Target
5460
Other
Seed Type
Heptamer
Hexamer

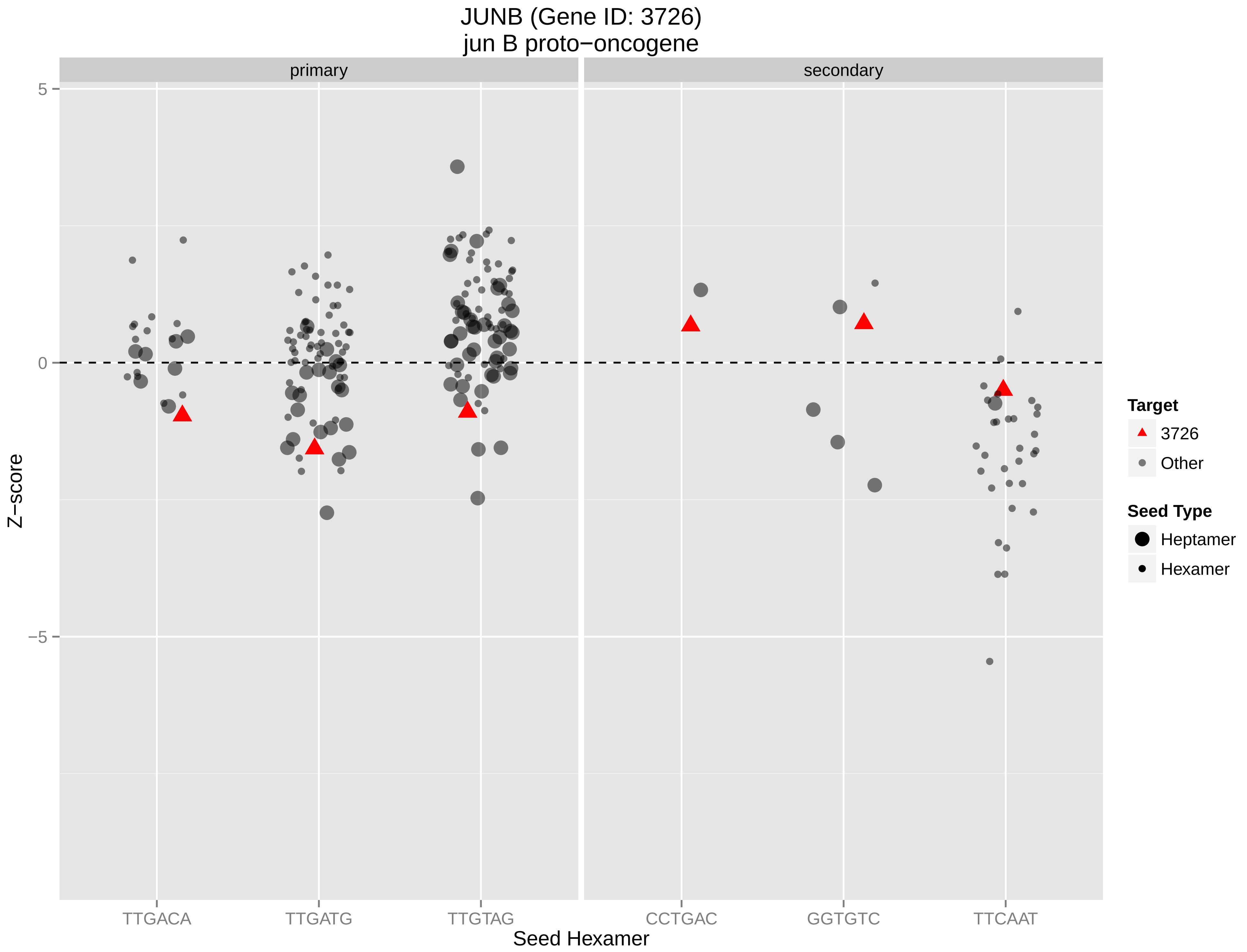

JUNB (Gene ID: 3726)
jun B proto−oncogene
primary
secondary
5
0
−5
Z−score
TTGACA
TTGATG
TTGTAG
CCTGAC
GGTGTC
TTCAAT
Seed Hexamer
Target
3726
Other
Seed Type
Heptamer
Hexamer

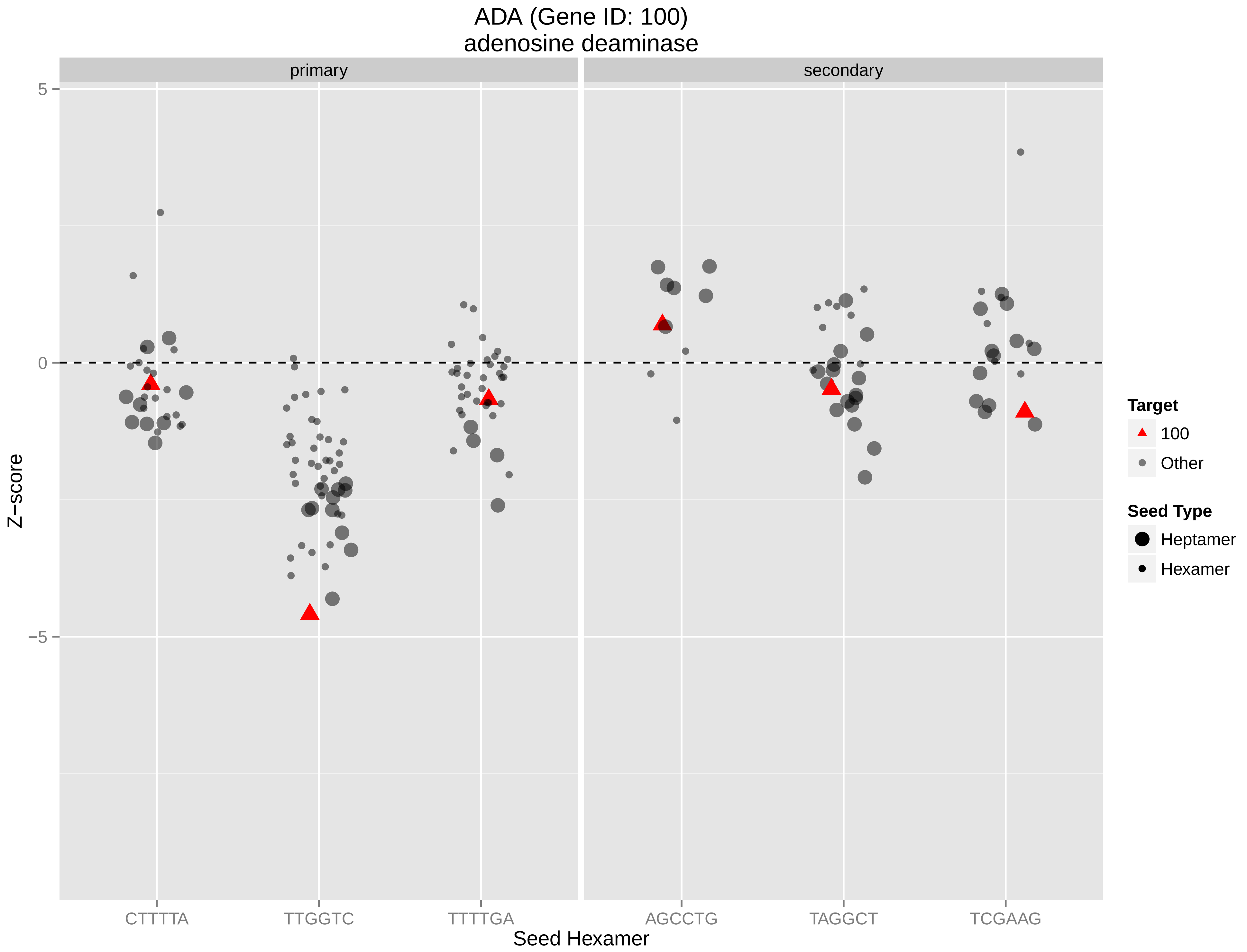
ADA (Gene ID: 100)
adenosine deaminase
primary
secondary
5
0
−5
Z−score
CTTTTA
TTGGTC
TTTTGA
AGCCTG
TAGGCT
TCGAAG
Seed Hexamer
Target
100
Other
Seed Type
Heptamer
Hexamer

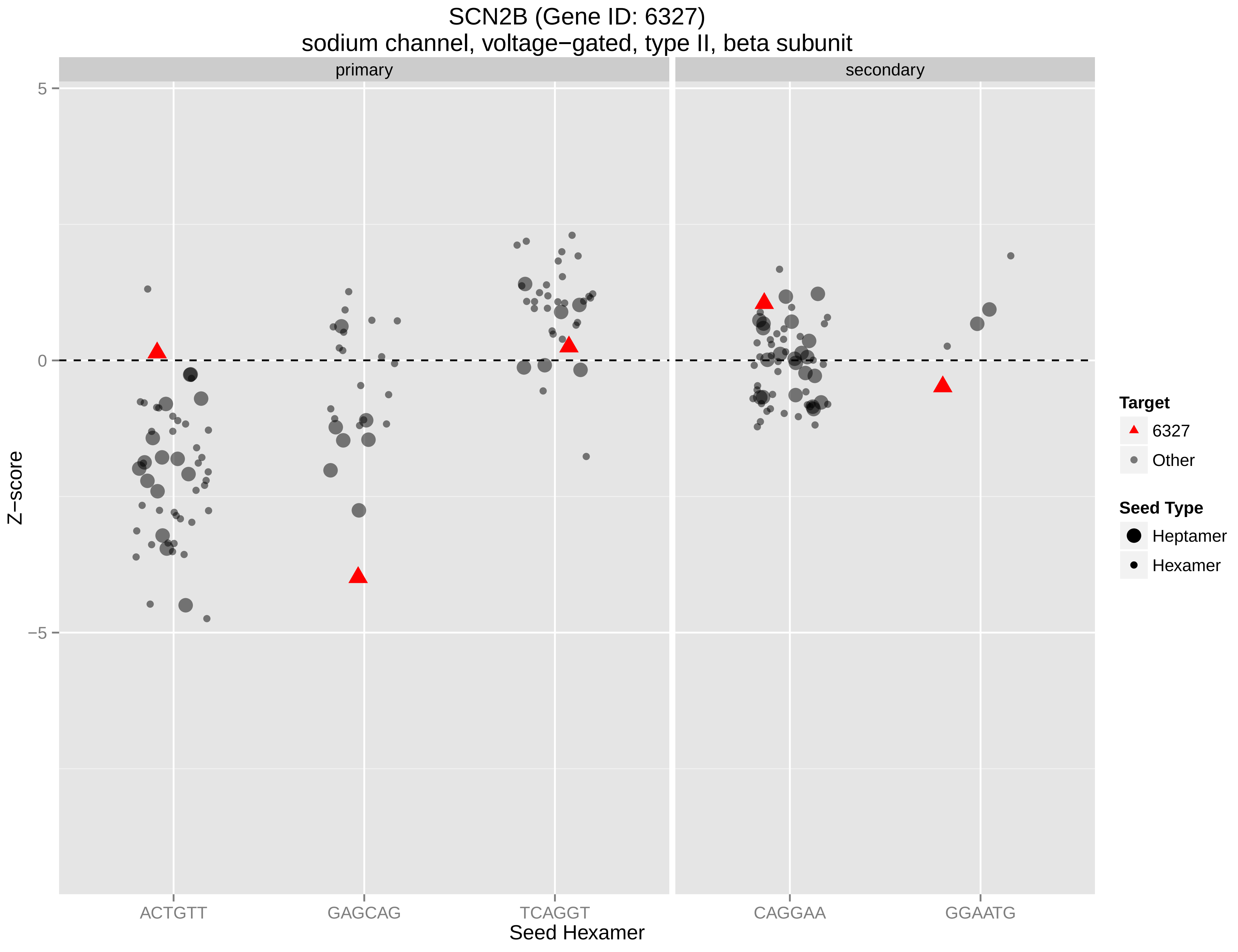

SCN2B (Gene ID: 6327)
sodium channel, voltage−gated, type II, beta subunit
primary
secondary
5
0
−5
Z−score
ACTGTT
GAGCAG
TCAGGT
CAGGAA
GGAATG
Seed Hexamer
Target
6327
Other
Seed Type
Heptamer
Hexamer

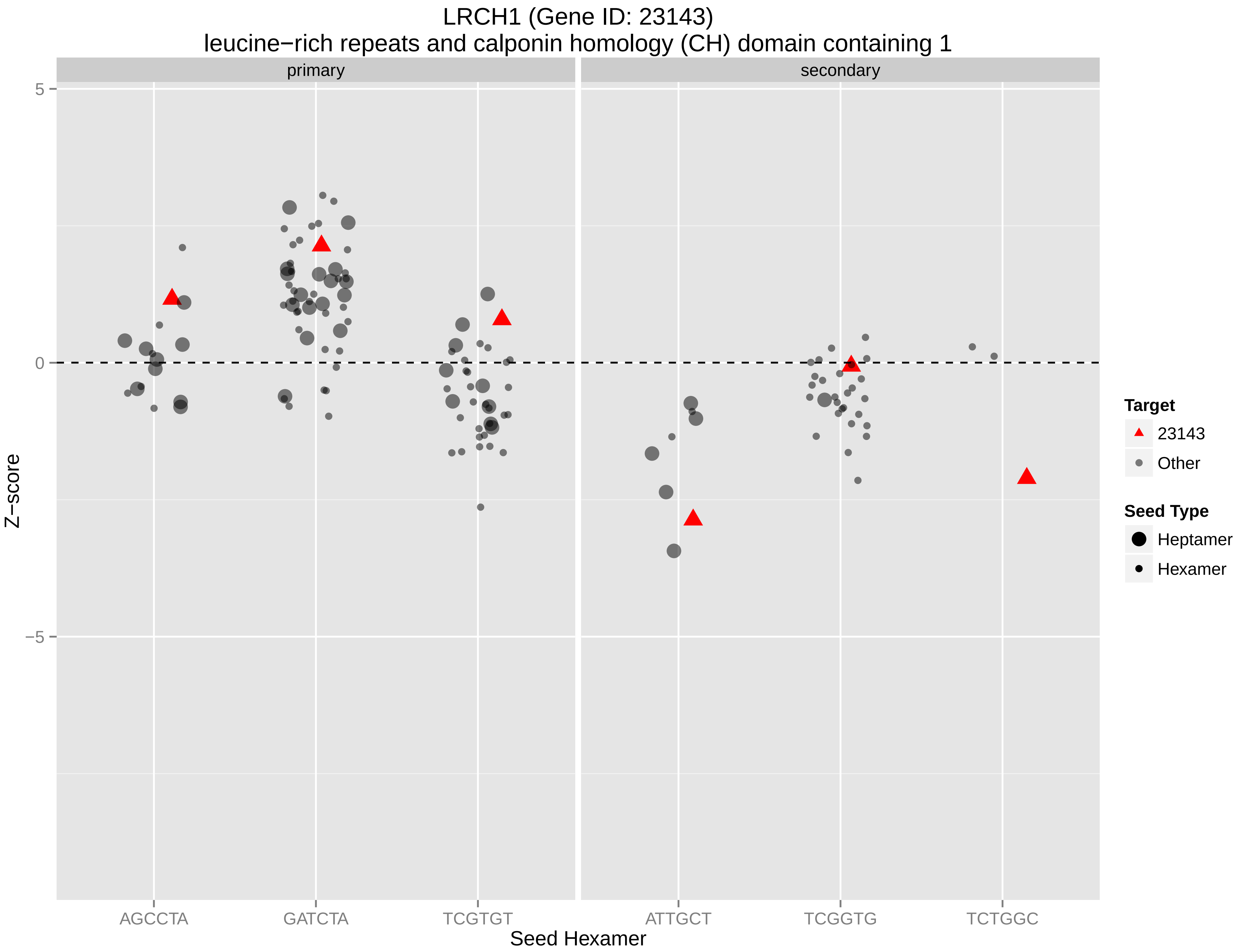

LRCH1 (Gene ID: 23143)
leucine−rich repeats and calponin homology (CH) domain containing 1
primary
secondary
5
0
−5
Z−score
AGCCTA
GATCTA
TCGTGT
ATTGCT
TCGGTG
TCTGGC
Seed Hexamer
Target
23143
Other
Seed Type
Heptamer
Hexamer

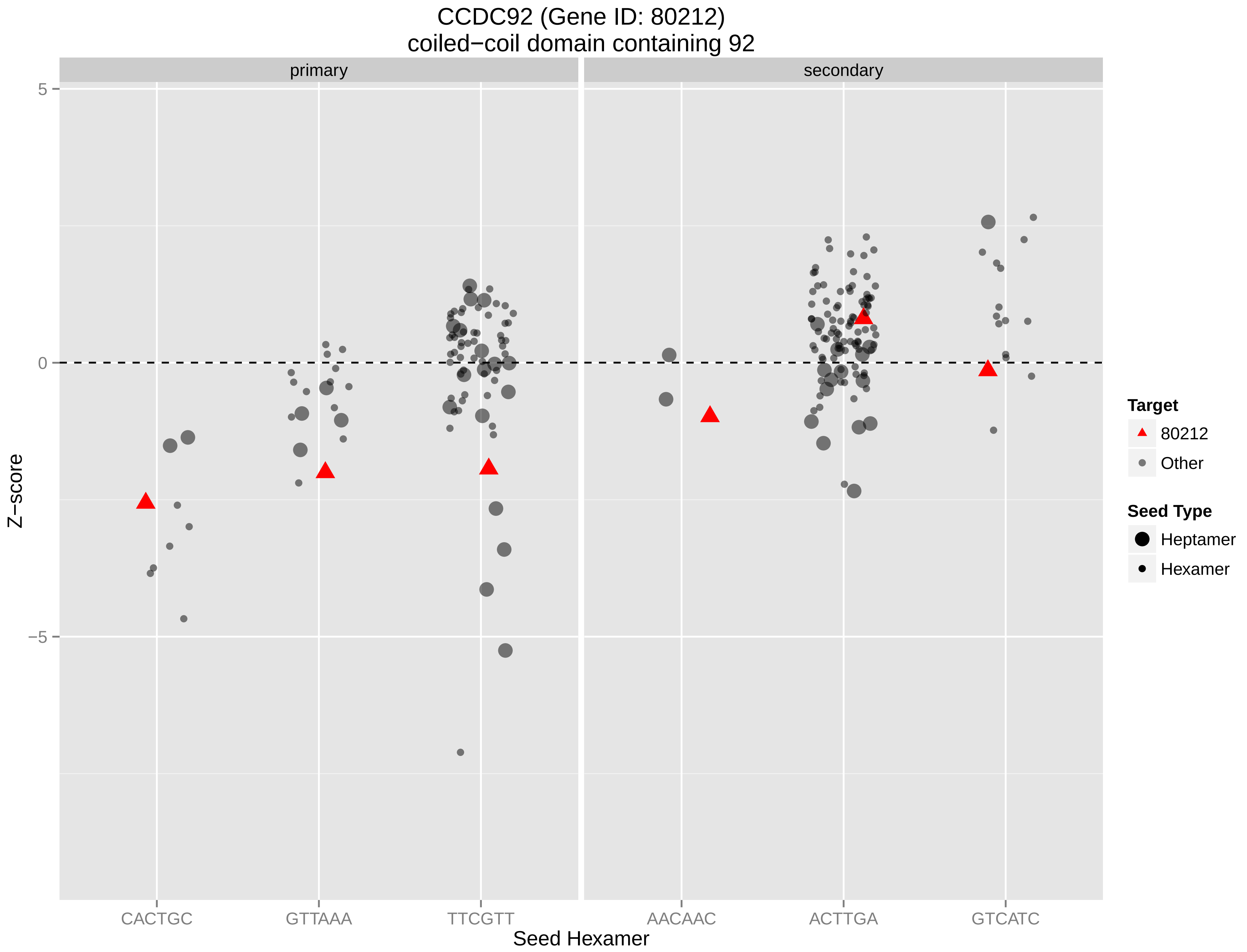
CCDC92 (Gene ID: 80212)
coiled−coil domain containing 92
primary
secondary
5
0
−5
Z−score
CACTGC
GTTAAA
TTCGTT
AACAAC
ACTTGA
GTCATC
Seed Hexamer
Target
80212
Other
Seed Type
Heptamer
Hexamer

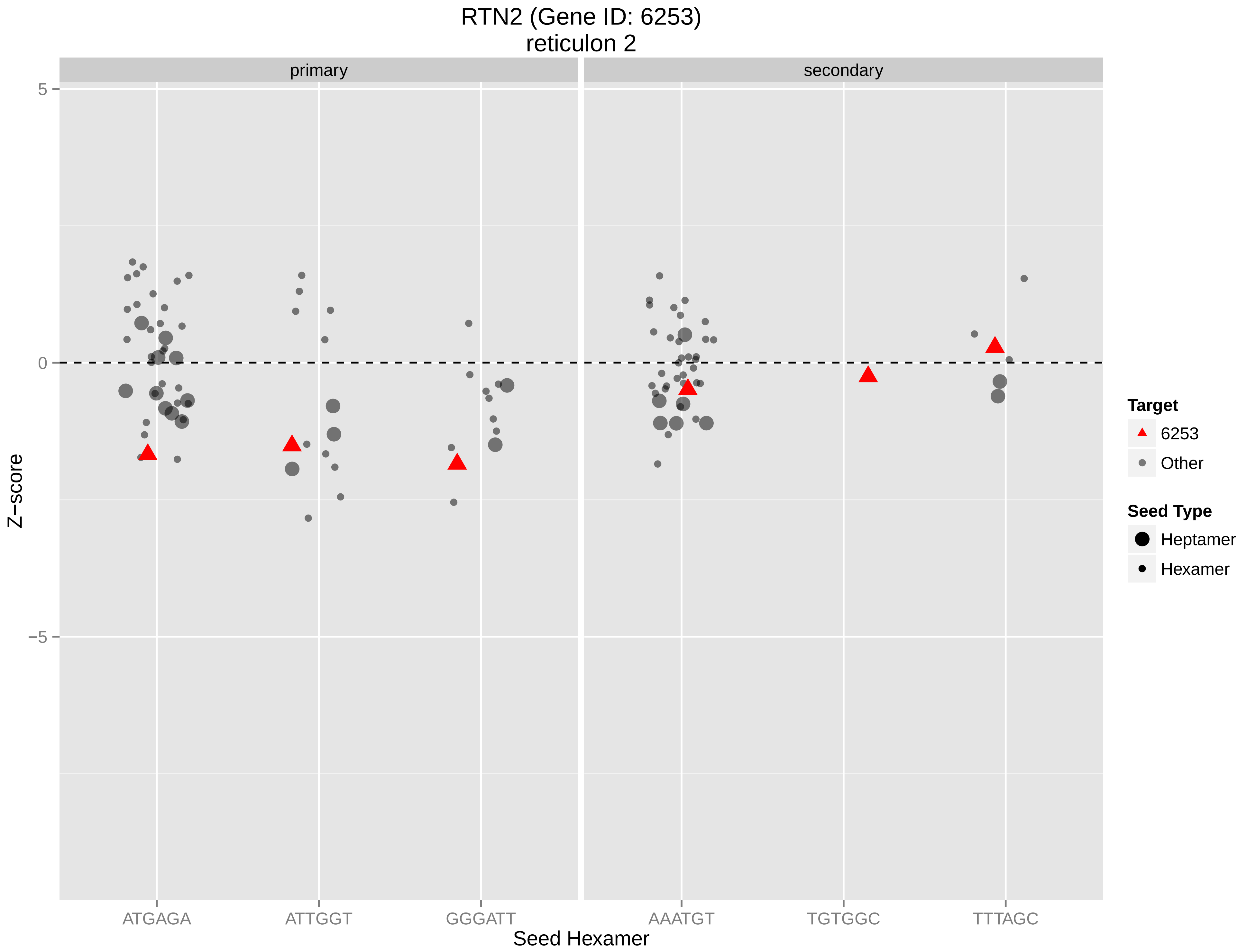
RTN2 (Gene ID: 6253)
reticulon 2
primary
secondary
5
0
−5
Z−score
ATGAGA
ATTGGT
GGGATT
AAATGT
TGTGGC
TTTAGC
Seed Hexamer
Target
6253
Other
Seed Type
Heptamer
Hexamer

TRAPPC2 (Gene ID: 6399)
trafficking protein particle complex 2

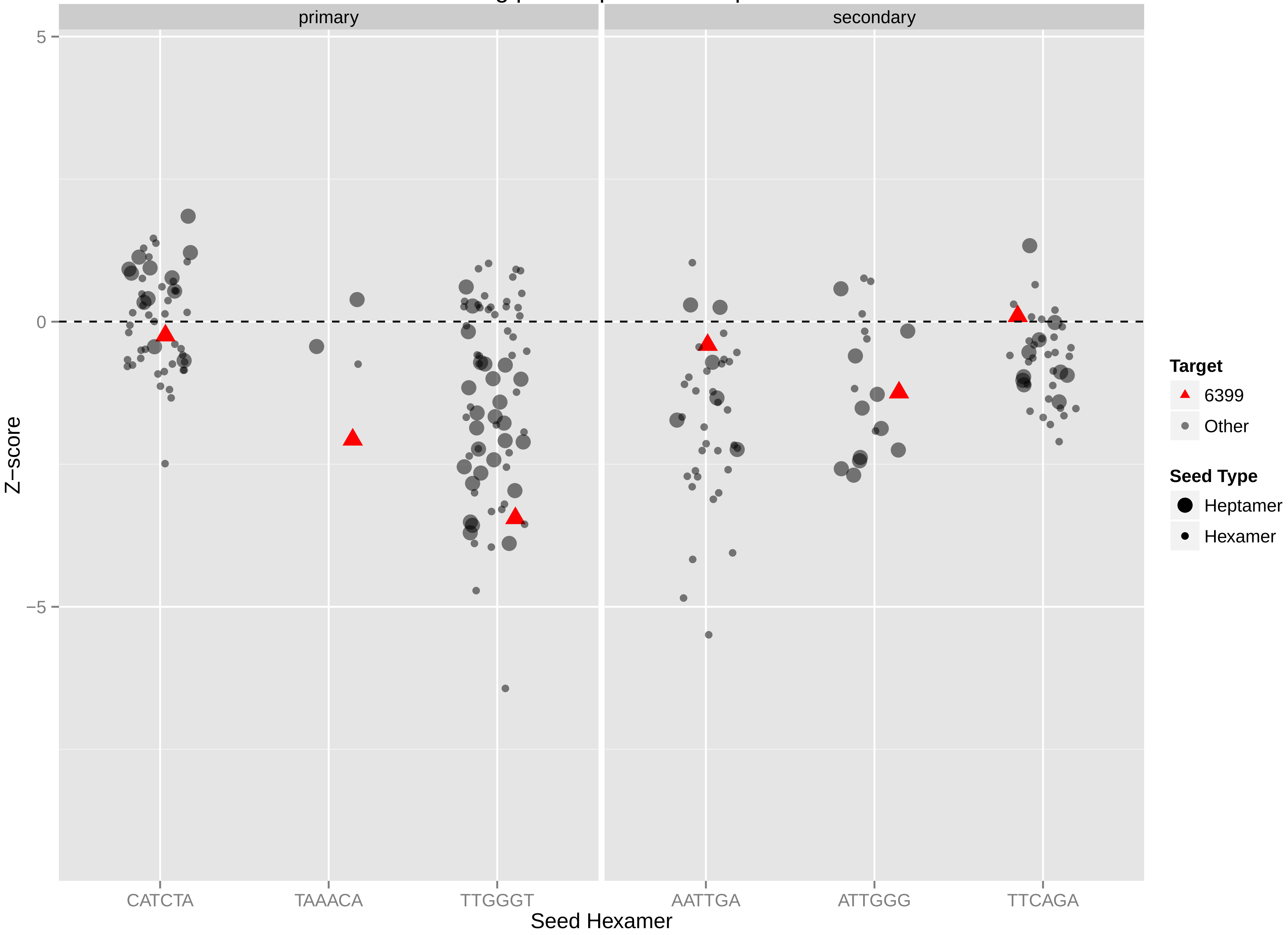

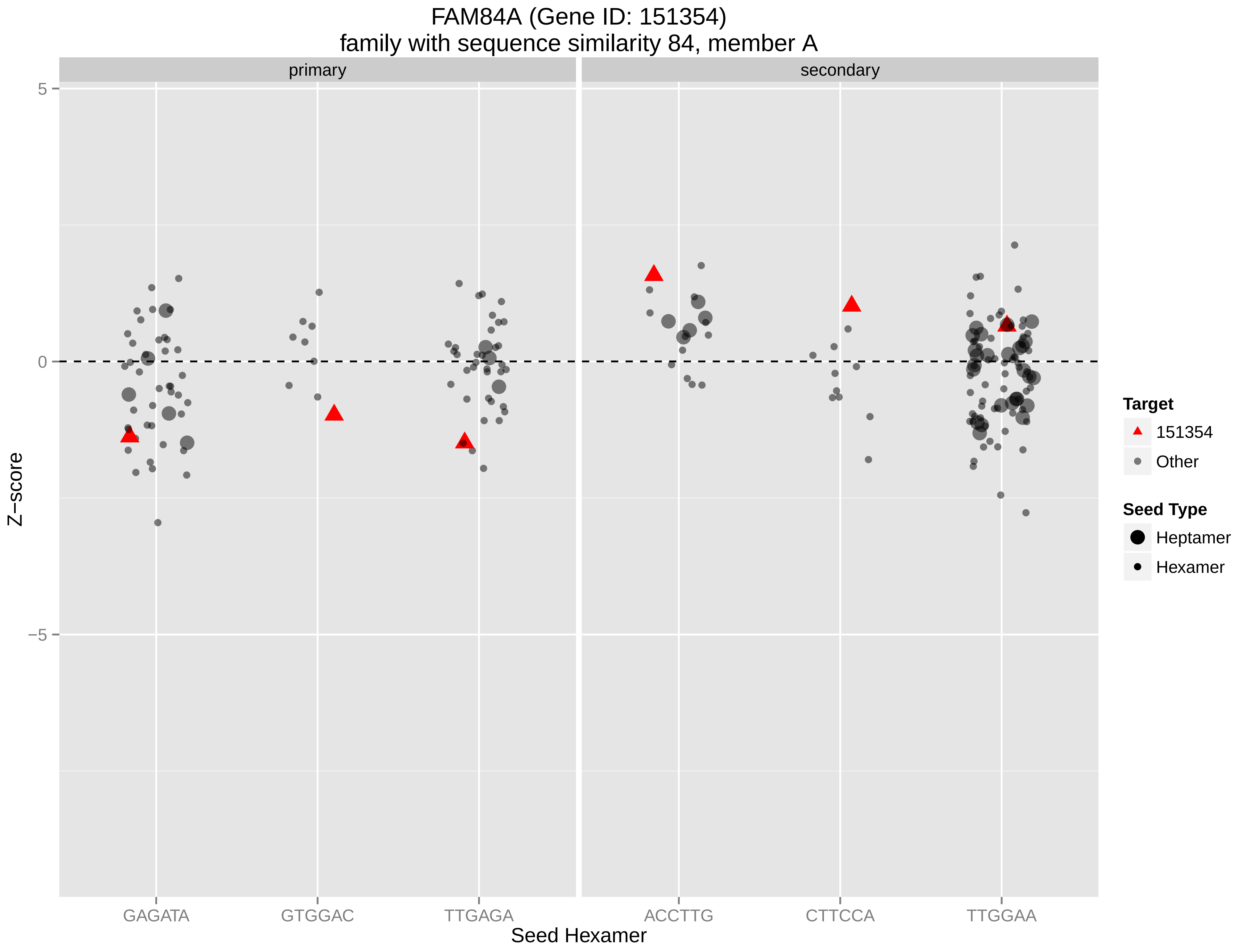
FAM84A (Gene ID: 151354)
family with sequence similarity 84, member A
primary
secondary
5
0
−5
Z−score
GAGATA
GTGGAC
TTGAGA
ACCTTG
CTTCCA
TTGGAA
Seed Hexamer
Target
151354
Other
Seed Type
Heptamer
Hexamer

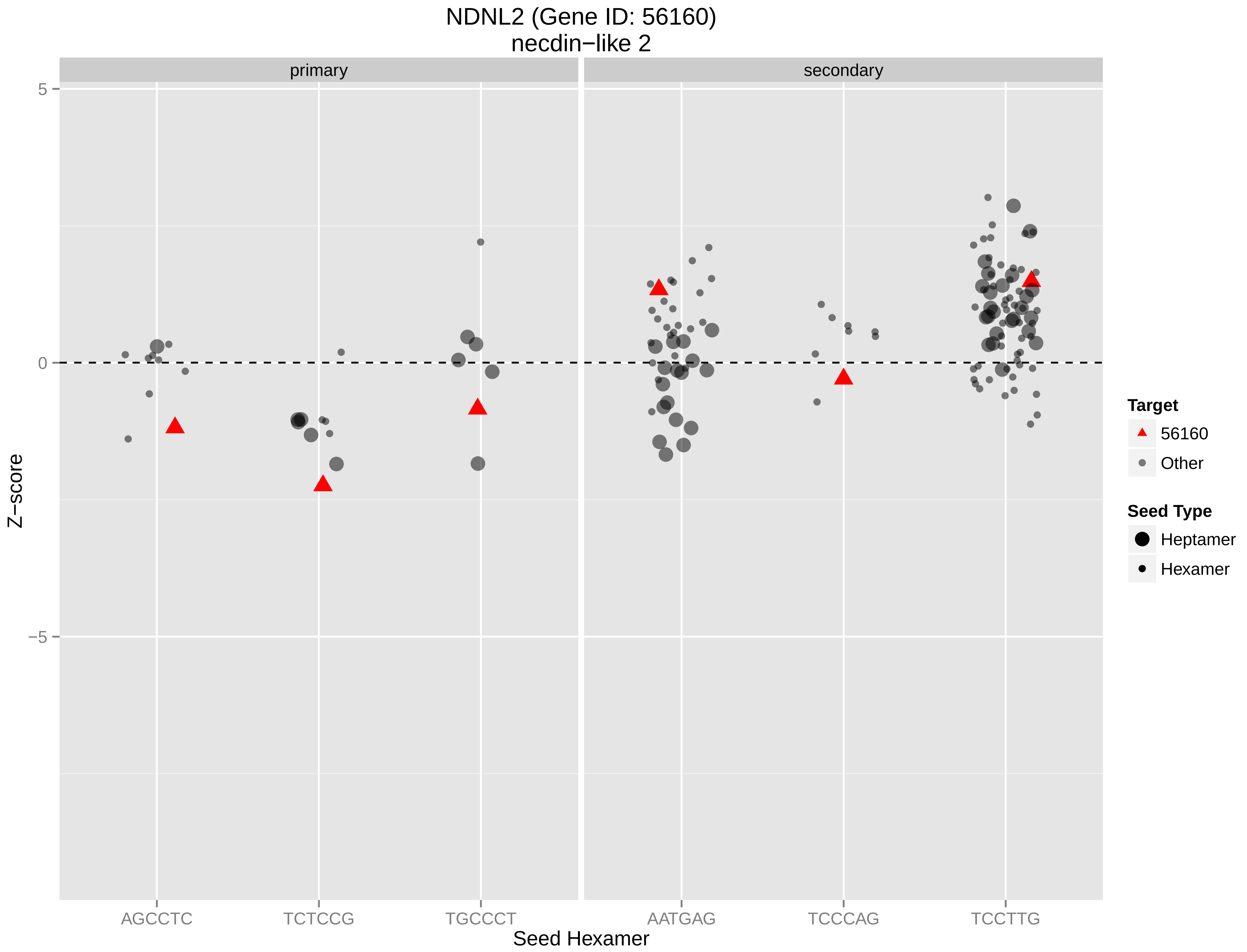
NDNL2 (Gene ID: 56160)
necdin−like 2
primary
secondary
Z−score
5
0
−5
AGCCTC
TCTCCG
TGCCCT
AATGAG
TCCCAG
TCCTTG
Seed Hexamer
Target
56160
Other
Seed Type
Heptamer
Hexamer

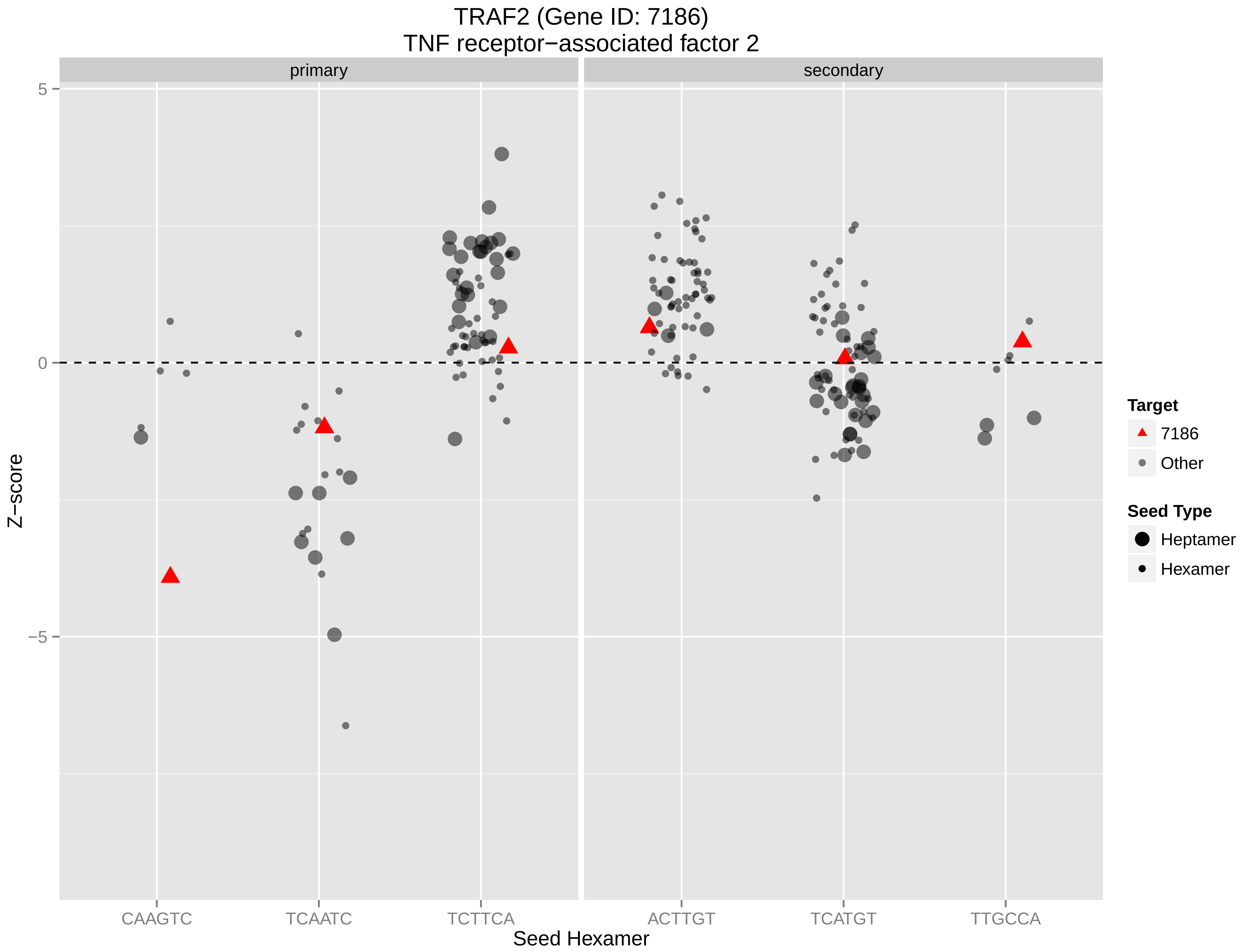
TRAF2 (Gene ID: 7186)
TNF receptor−associated factor 2
primary
secondary
5
0
−5
Z−score
CAAGTC
TCAATC
TCTTCA
ACTTGT
TCATGT
TTGCCA
Seed Hexamer
Target
7186
Other
Seed Type
Heptamer
Hexamer

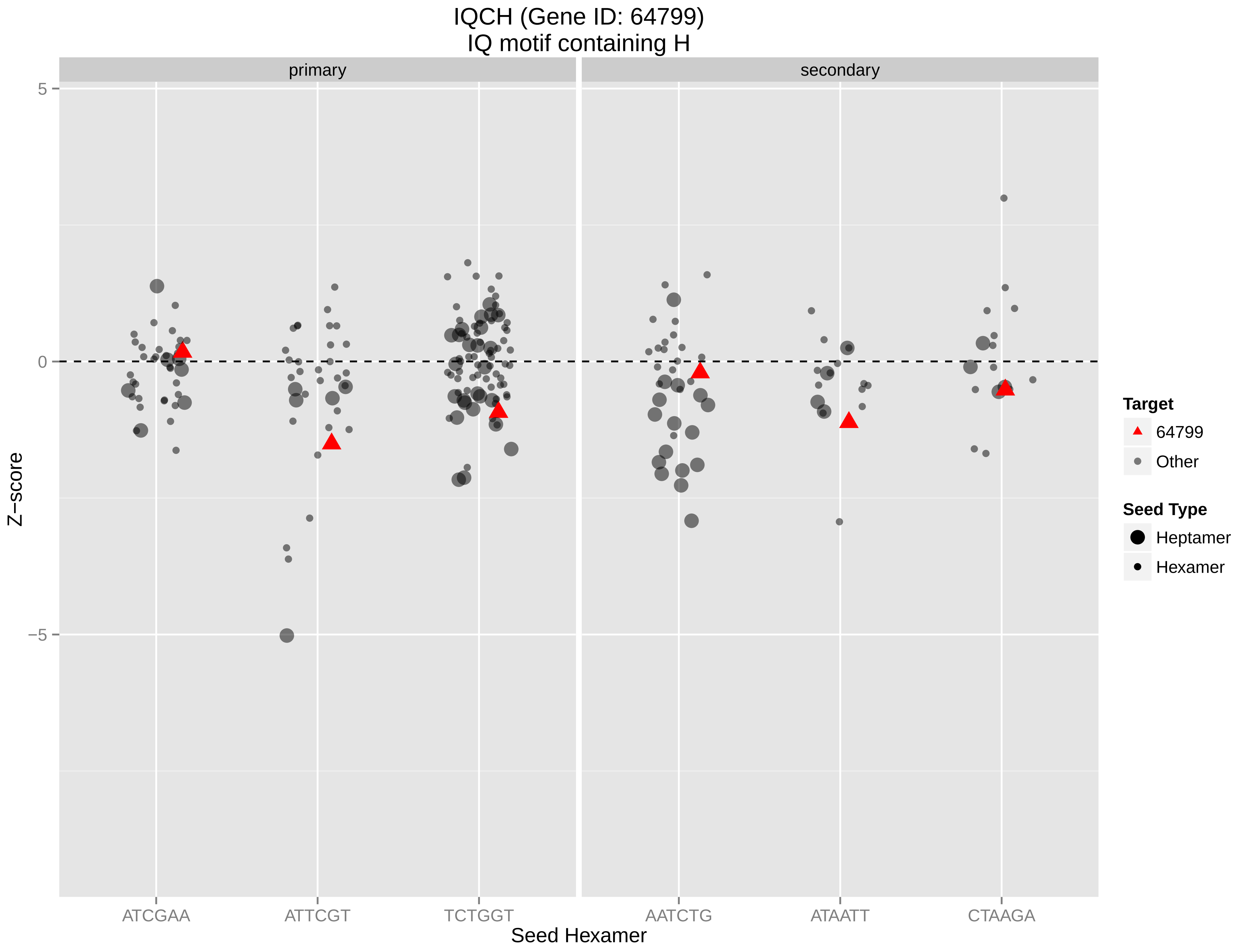
IQCH (Gene ID: 64799)
IQ motif containing H
primary
secondary
5
0
−5
Z−score
ATCGAA
ATTCGT
TCTGGT
AATCTG
ATAATT
CTAAGA
Seed Hexamer
Target
64799
Other
Seed Type
Heptamer
Hexamer

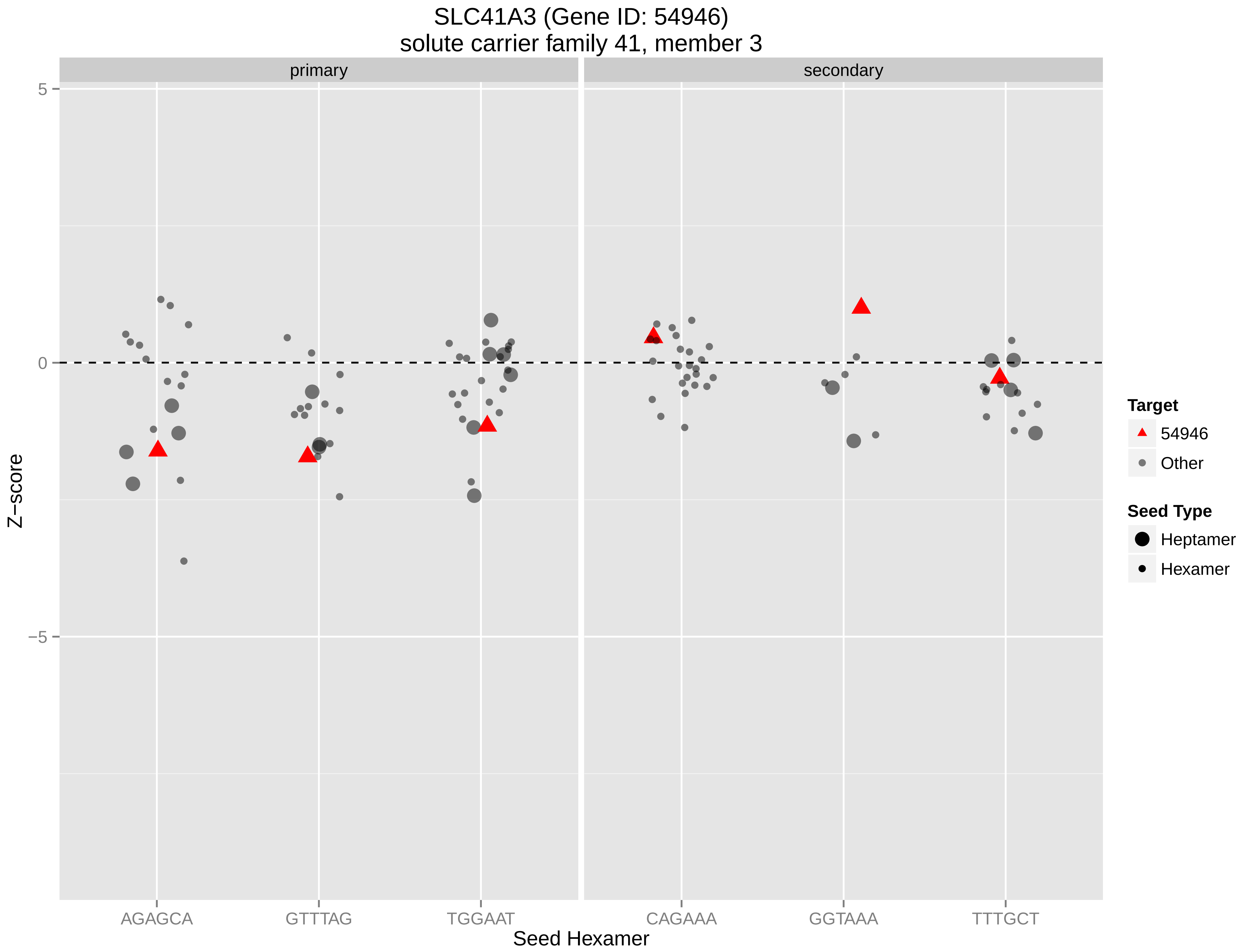

SLC41A3 (Gene ID: 54946)
solute carrier family 41, member 3
primary
secondary
5
0
−5
Z−score
AGAGCA
GTTTAG
TGGAAT
CAGAAA
GGTAAA
TTTGCT
Seed Hexamer
Target
54946
Other
Seed Type
Heptamer
Hexamer

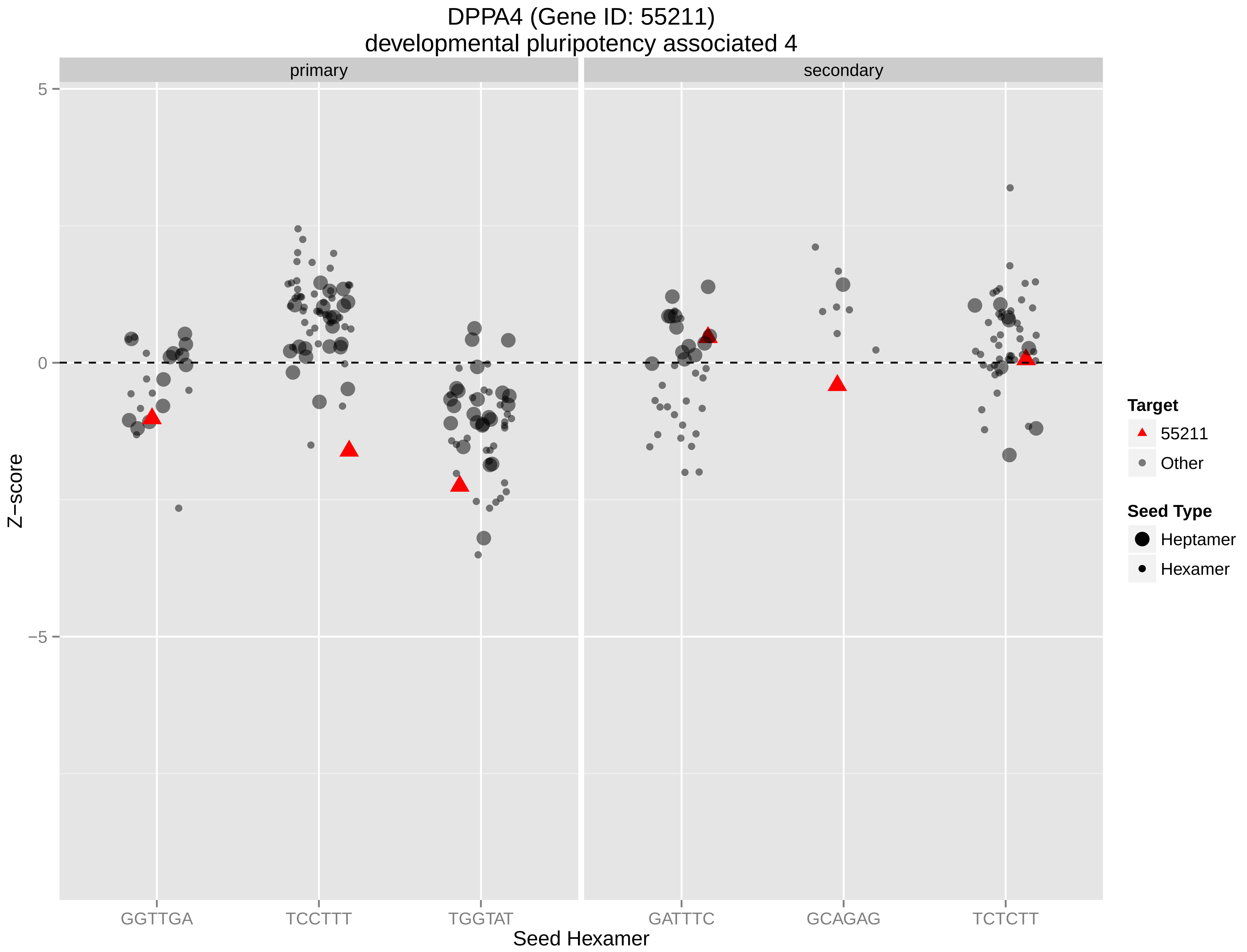

DPPA4 (Gene ID: 55211)
developmental pluripotency associated 4
primary
secondary
5
0
−5
Z−score
GGTTGA
TCCTTT
TGGTAT
GATTTC
GCAGAG
TCTCTT
Seed Hexamer
Target
55211
Other
Seed Type
Heptamer
Hexamer

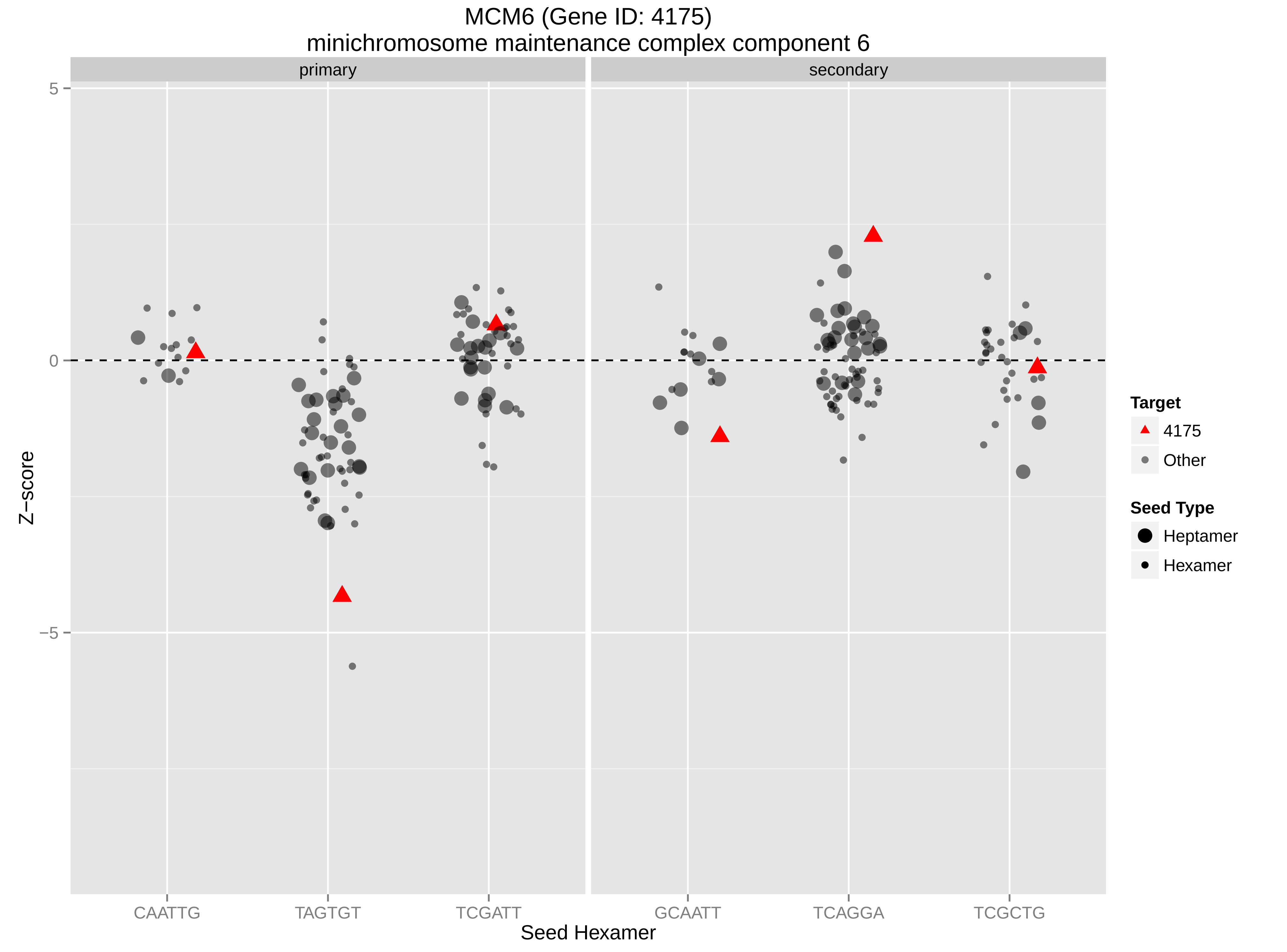
MCM6 (Gene ID: 4175)
minichromosome maintenance complex component 6
primary
secondary
5
0
−5
Z−score
CAATTG
TAGTGT
TCGATT
GCAATT
TCAGGA
TCGCTG
Seed Hexamer
Target
4175
Other
Seed Type
Heptamer
Hexamer

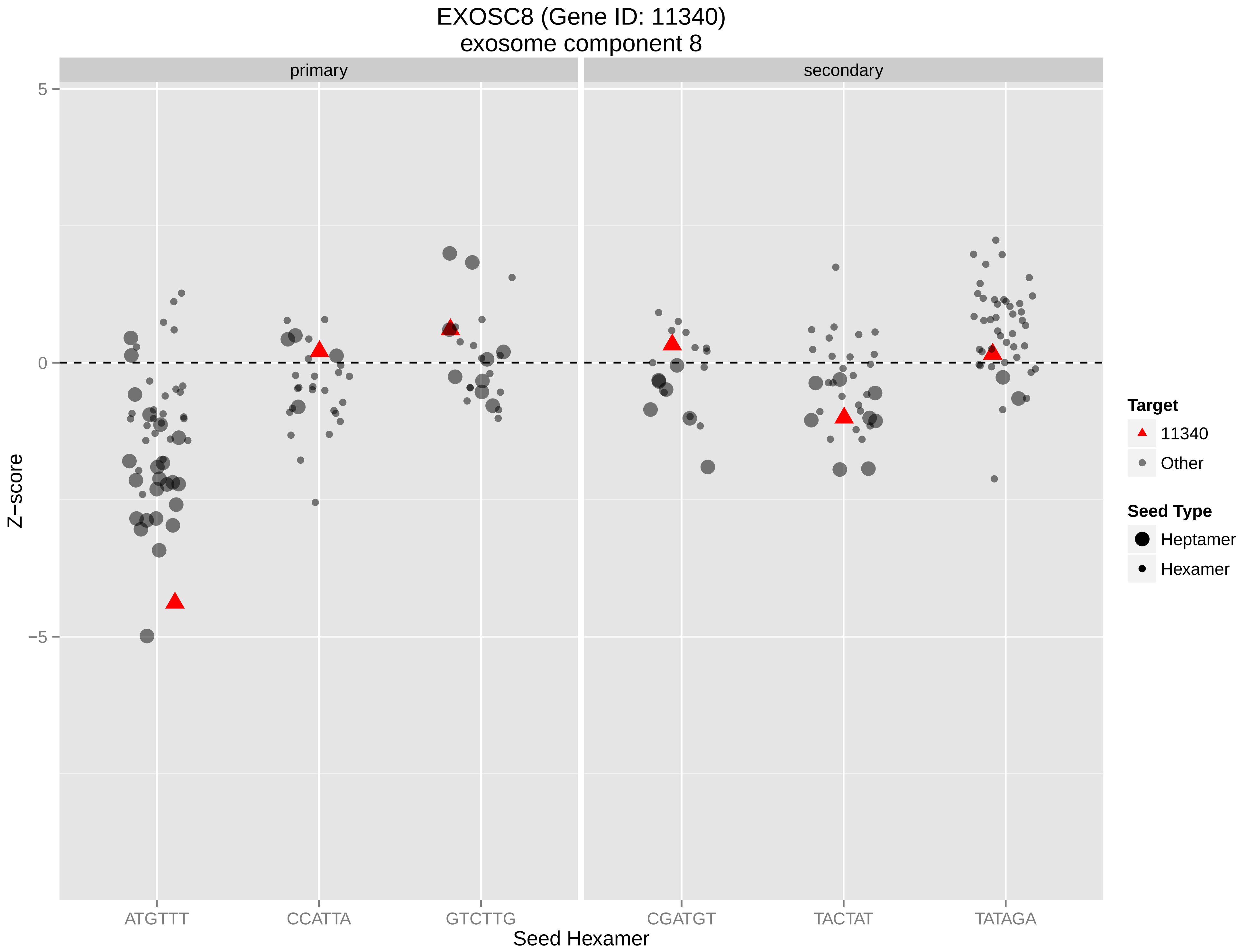

EXOSC8 (Gene ID: 11340)
exosome component 8
primary
secondary
5
0
−5
Z−score
ATGTTT
CCATTA
GTCTTG
CGATGT
TACTAT
TATAGA
Seed Hexamer
Target
11340
Other
Seed Type
Heptamer
Hexamer

MPEG1 (Gene ID: 219972)
macrophage expressed 1

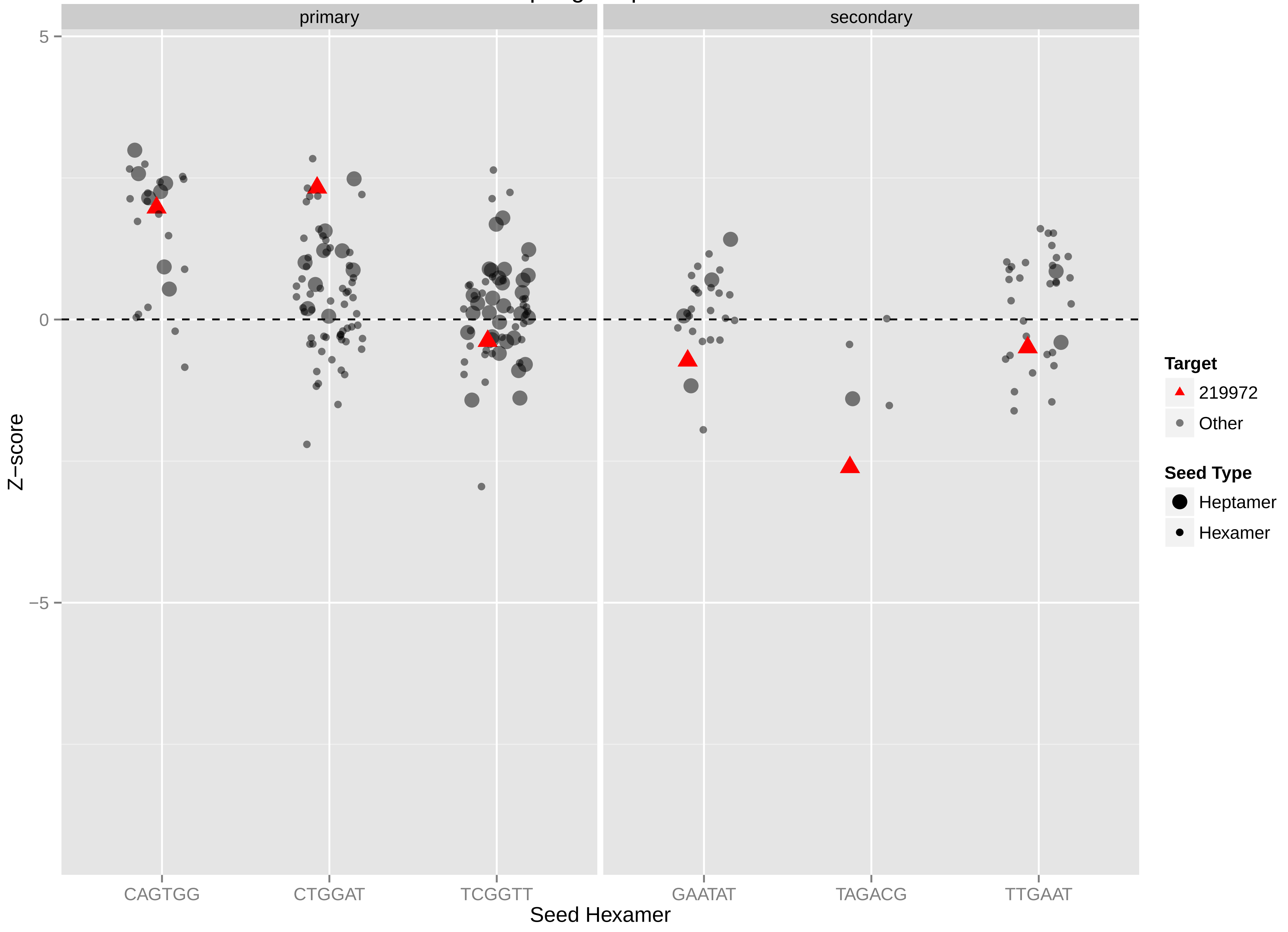

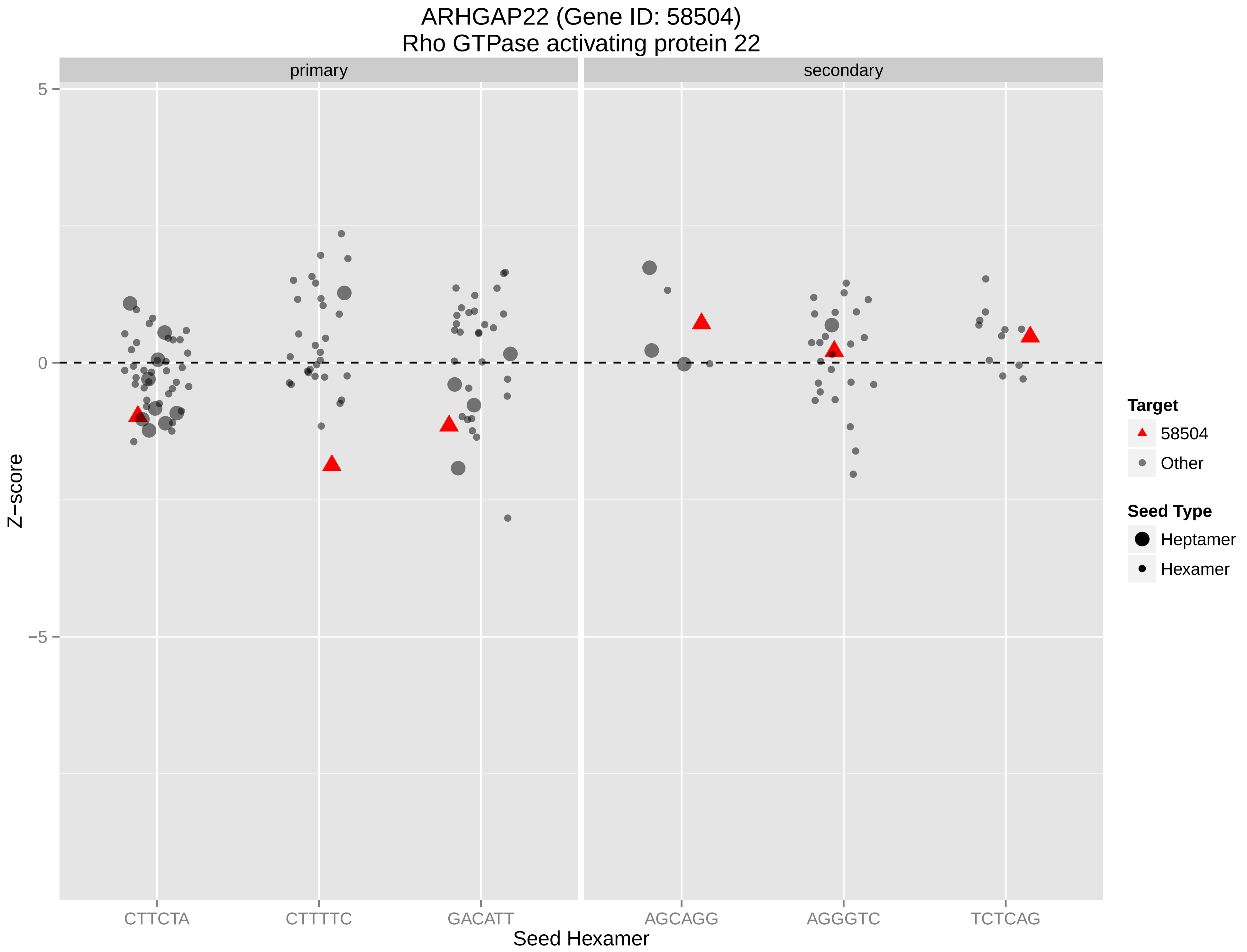
ARHGAP22 (Gene ID: 58504)
Rho GTPase activating protein 22
primary
secondary
5
0
−5
Z−score
CTTCTA
CTTTTC
GACATT
AGCAGG
AGGGTC
TCTCAG
Seed Hexamer
Target
58504
Other
Seed Type
Heptamer
Hexamer

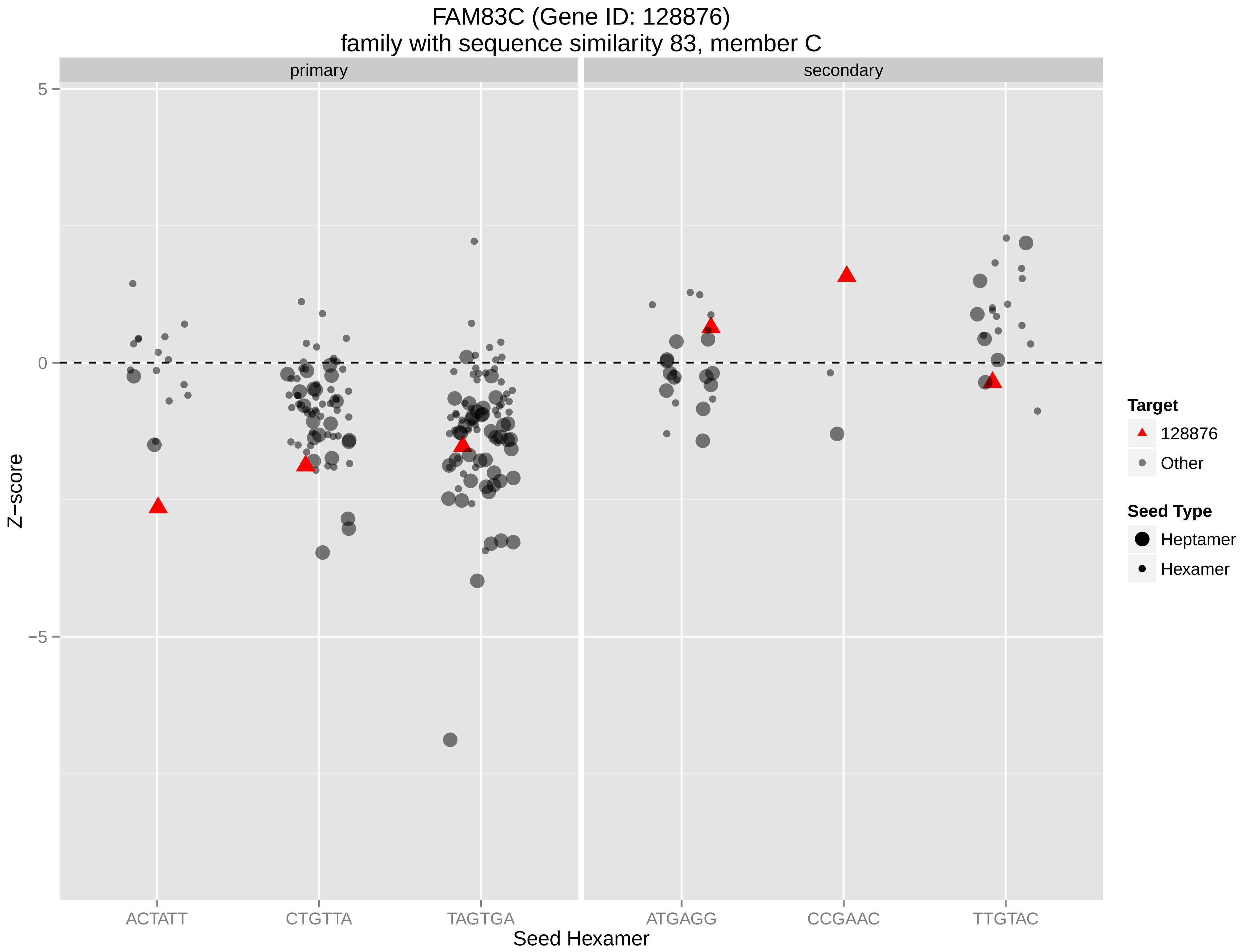
FAM83C (Gene ID: 128876)
family with sequence similarity 83, member C
primary
secondary
5
0
−5
Z−score
ACTATT
CTGTTA
TAGTGA
ATGAGG
CCGAAC
TTGTAC
Seed Hexamer
Target
128876
Other
Seed Type
Heptamer
Hexamer

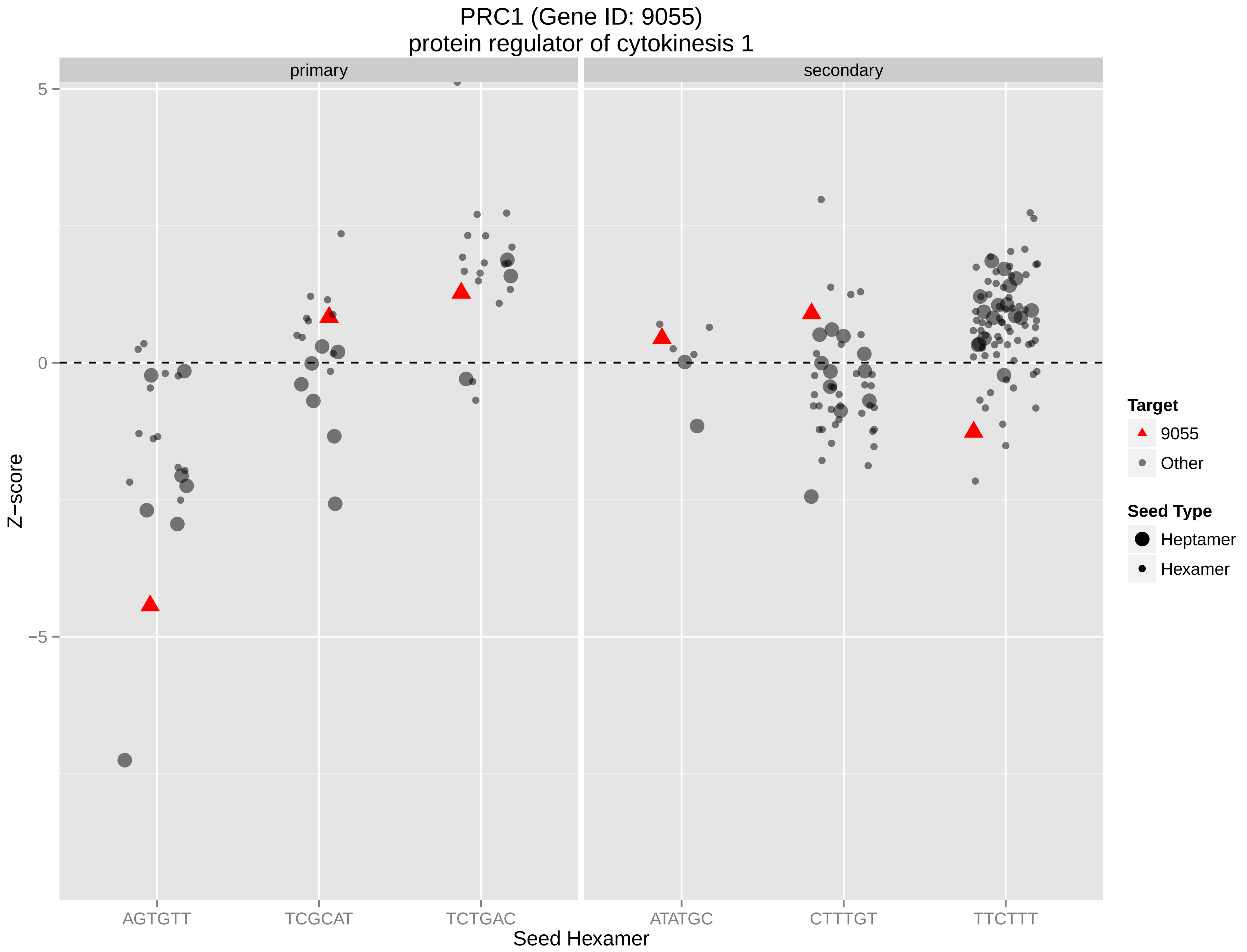
PRC1 (Gene ID: 9055)
protein regulator of cytokinesis 1
primary
secondary
5
0
−5
Z−score
AGTGTT
TCGCAT
TCTGAC
ATATGC
CTTTGT
TTCTTT
Seed Hexamer
Target
9055
Other
Seed Type
Heptamer
Hexamer

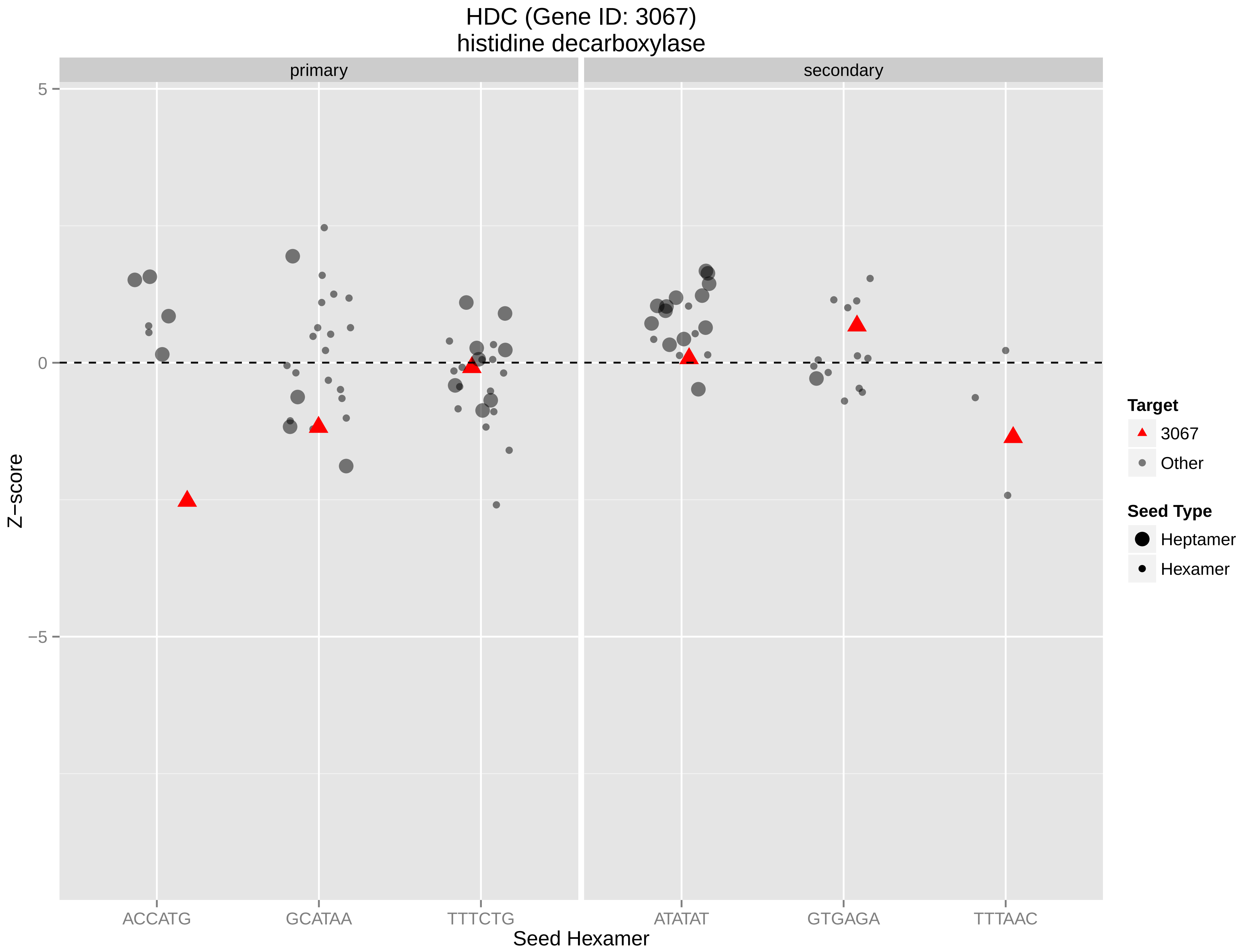
HDC (Gene ID: 3067)
histidine decarboxylase
primary
secondary
5
0
−5
Z−score
ACCATG
GCATAA
TTTCTG
ATATAT
GTGAGA
TTTAAC
Seed Hexamer
Target
3067
Other
Seed Type
Heptamer
Hexamer

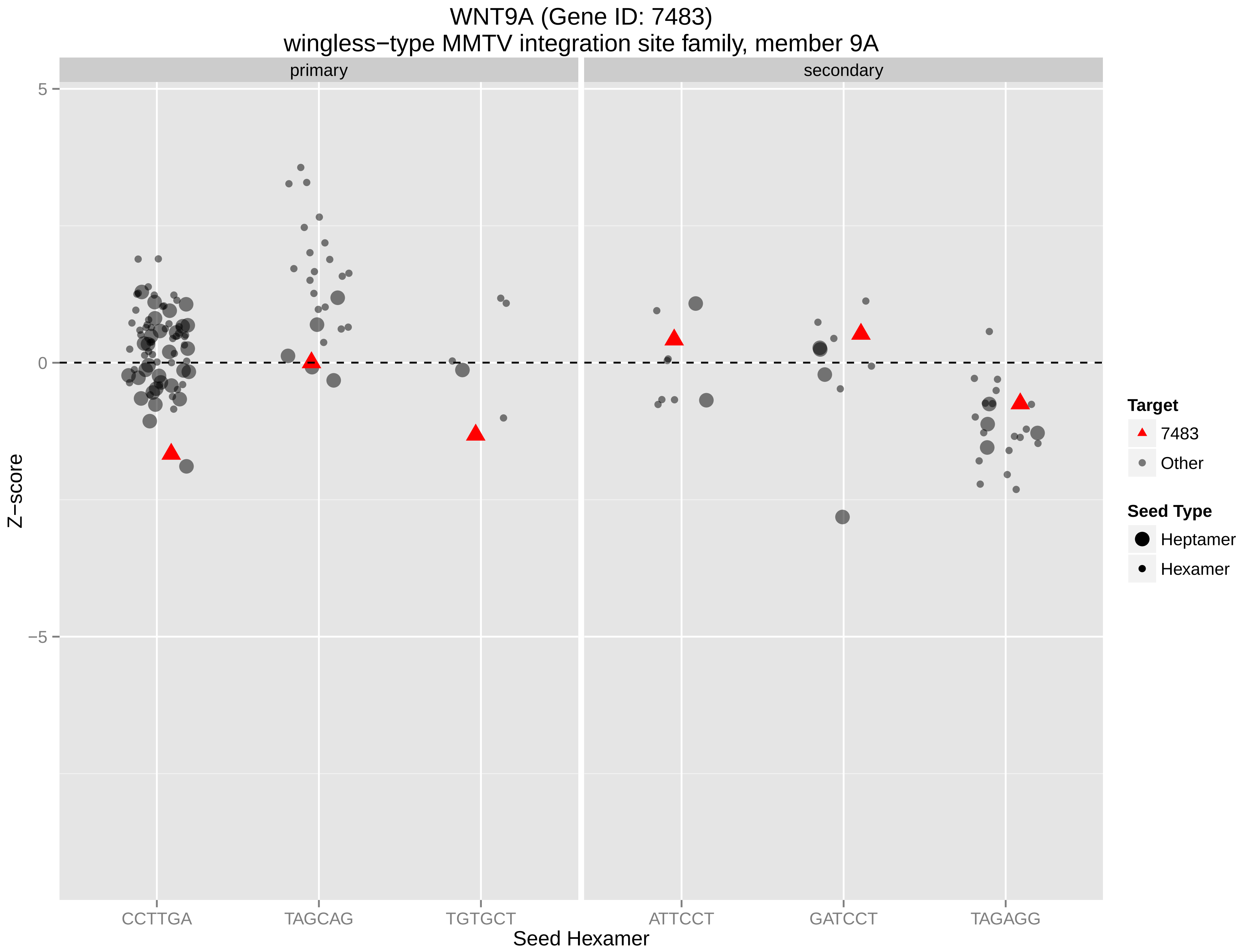
WNT9A (Gene ID: 7483)
wingless−type MMTV integration site family, member 9A
primary
secondary
5
0
−5
Z−score
CCTTGA
TAGCAG
TGTGCT
ATTCCT
GATCCT
TAGAGG
Seed Hexamer
Target
7483
Other
Seed Type
Heptamer
Hexamer

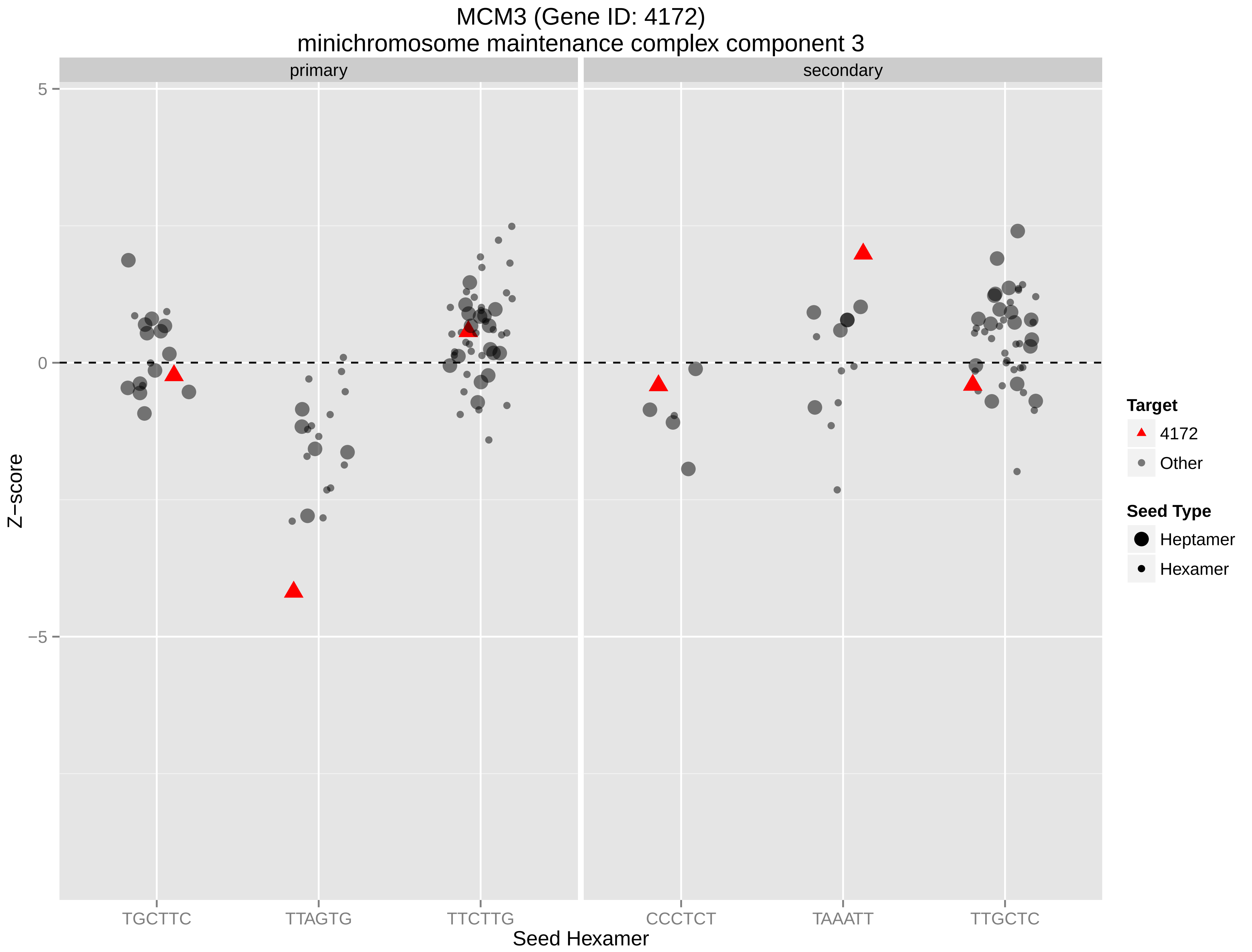

MCM3 (Gene ID: 4172)
minichromosome maintenance complex component 3
primary
secondary
5
0
−5
Z−score
TGCTTC
TTAGTG
TTCTTG
CCCTCT
TAAATT
TTGCTC
Seed Hexamer
Target
4172
Other
Seed Type
Heptamer
Hexamer

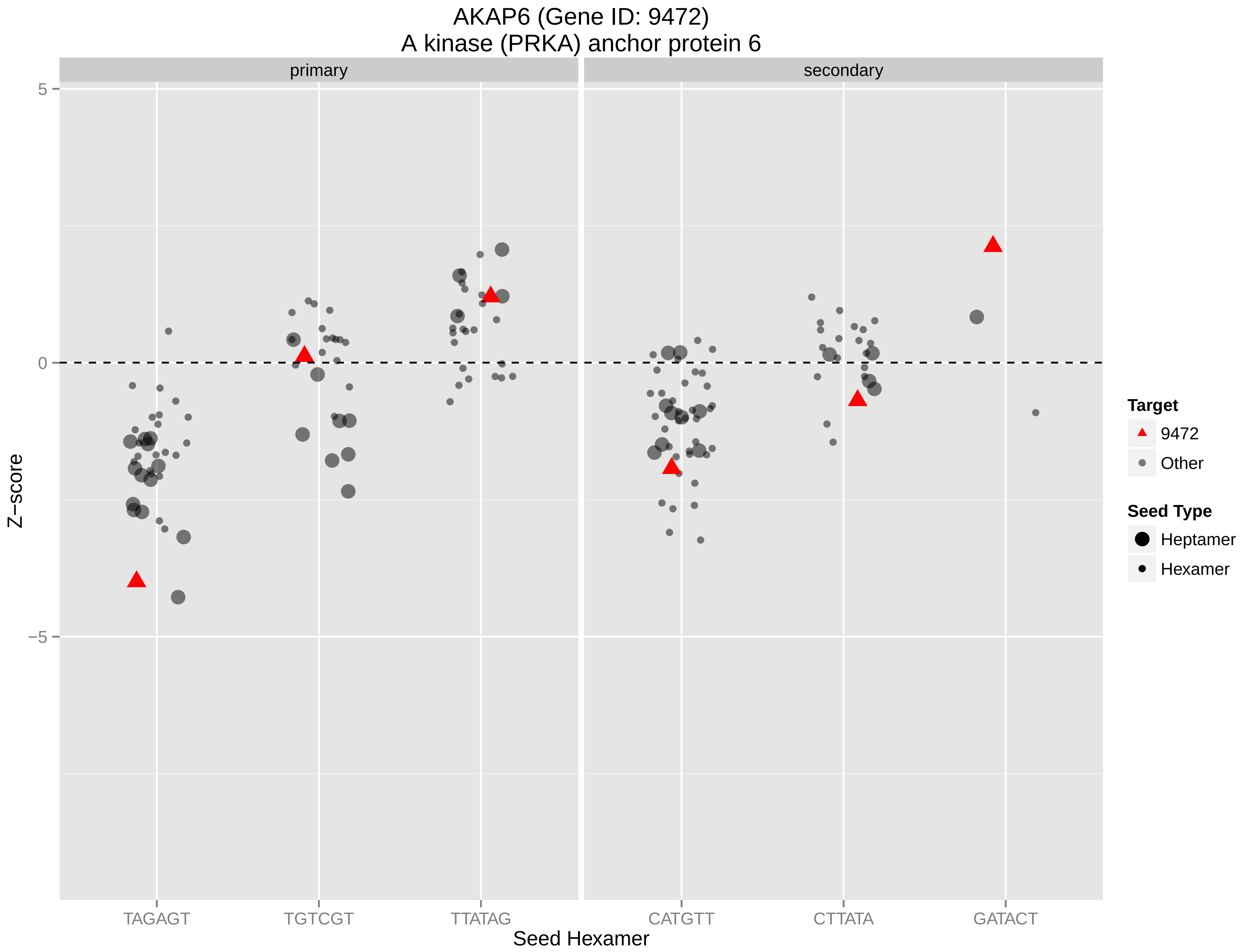
AKAP6 (Gene ID: 9472)
A kinase (PRKA) anchor protein 6
primary
secondary
5
0
−5
Z−score
TAGAGT
TGTCGT
TTATAG
CATGTT
CTTATA
GATACT
Seed Hexamer
Target
9472
Other
Seed Type
Heptamer
Hexamer

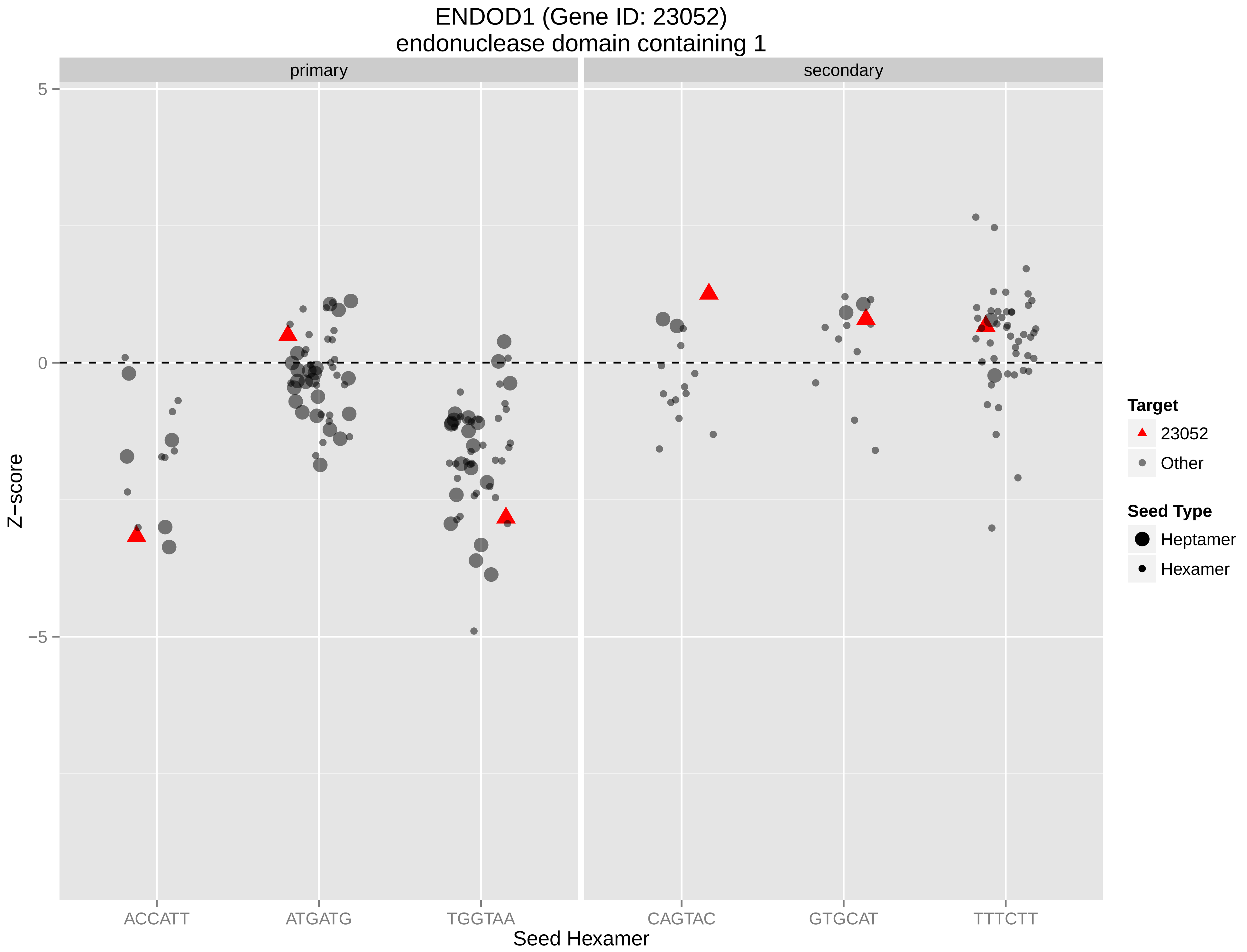

ENDOD1 (Gene ID: 23052)
endonuclease domain containing 1
primary
secondary
5
0
−5
Z−score
ACCATT
ATGATG
TGGTAA
CAGTAC
GTGCAT
TTTCTT
Seed Hexamer
Target
23052
Other
Seed Type
Heptamer
Hexamer

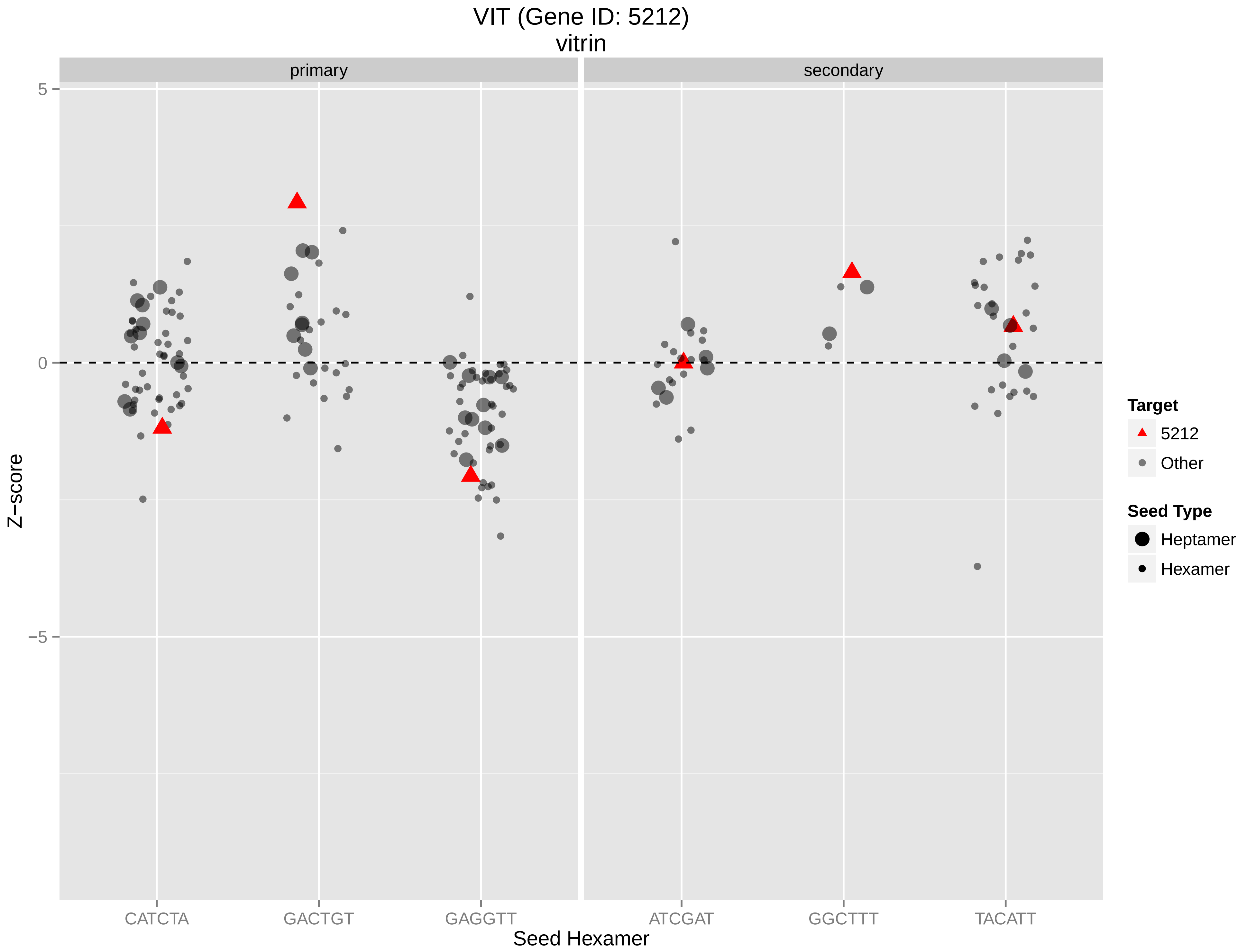

VIT (Gene ID: 5212)
vitrin
primary
secondary
5
0
−5
Z−score
CATCTA
GACTGT
GAGGTT
ATCGAT
GGCTTT
TACATT
Seed Hexamer
Target
5212
Other
Seed Type
Heptamer
Hexamer

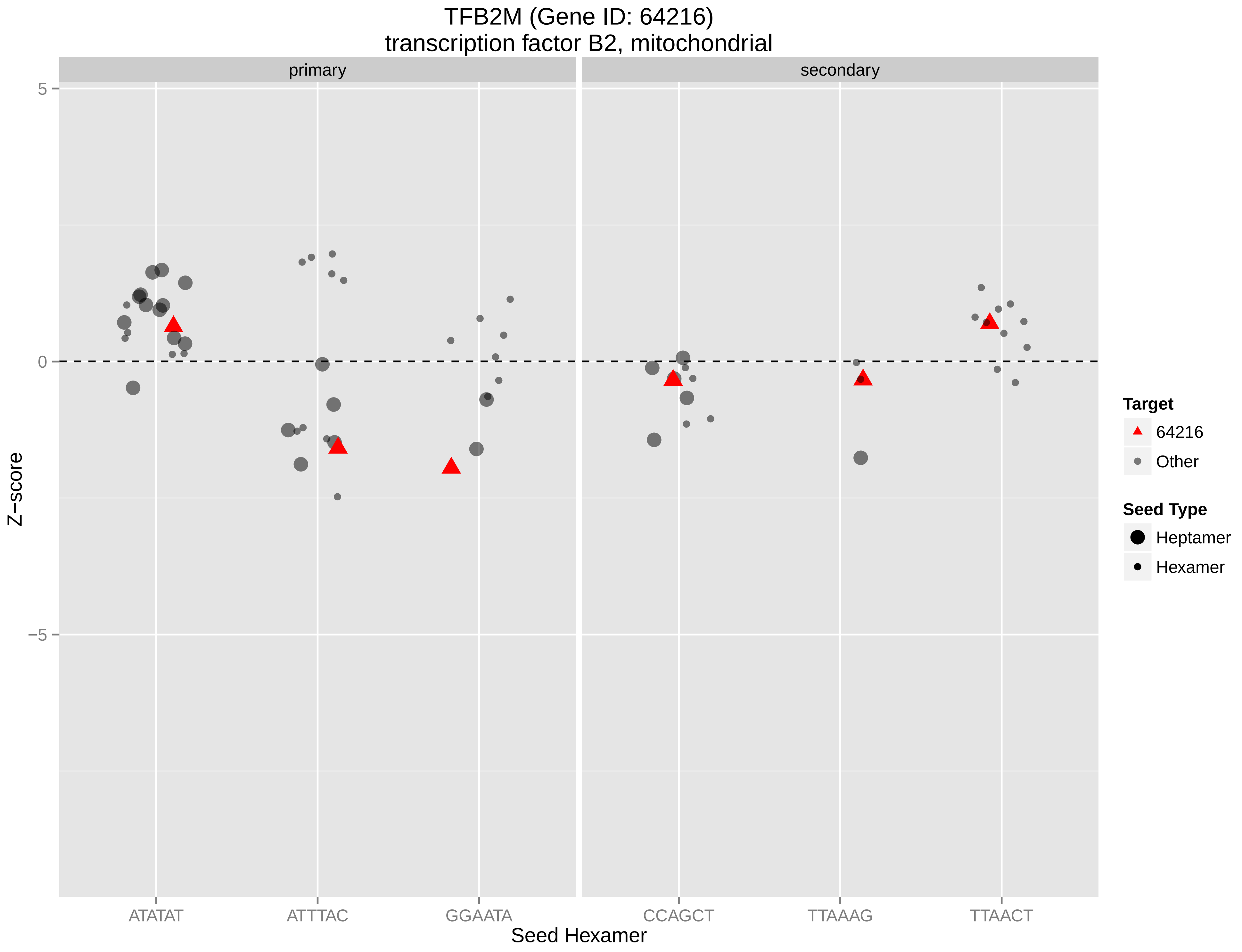
TFB2M (Gene ID: 64216)
transcription factor B2, mitochondrial
primary
secondary
5
0
−5
Z−score
ATATAT
ATTTAC
GGAATA
CCAGCT
TTAAAG
TTAACT
Seed Hexamer
Target
64216
Other
Seed Type
Heptamer
Hexamer

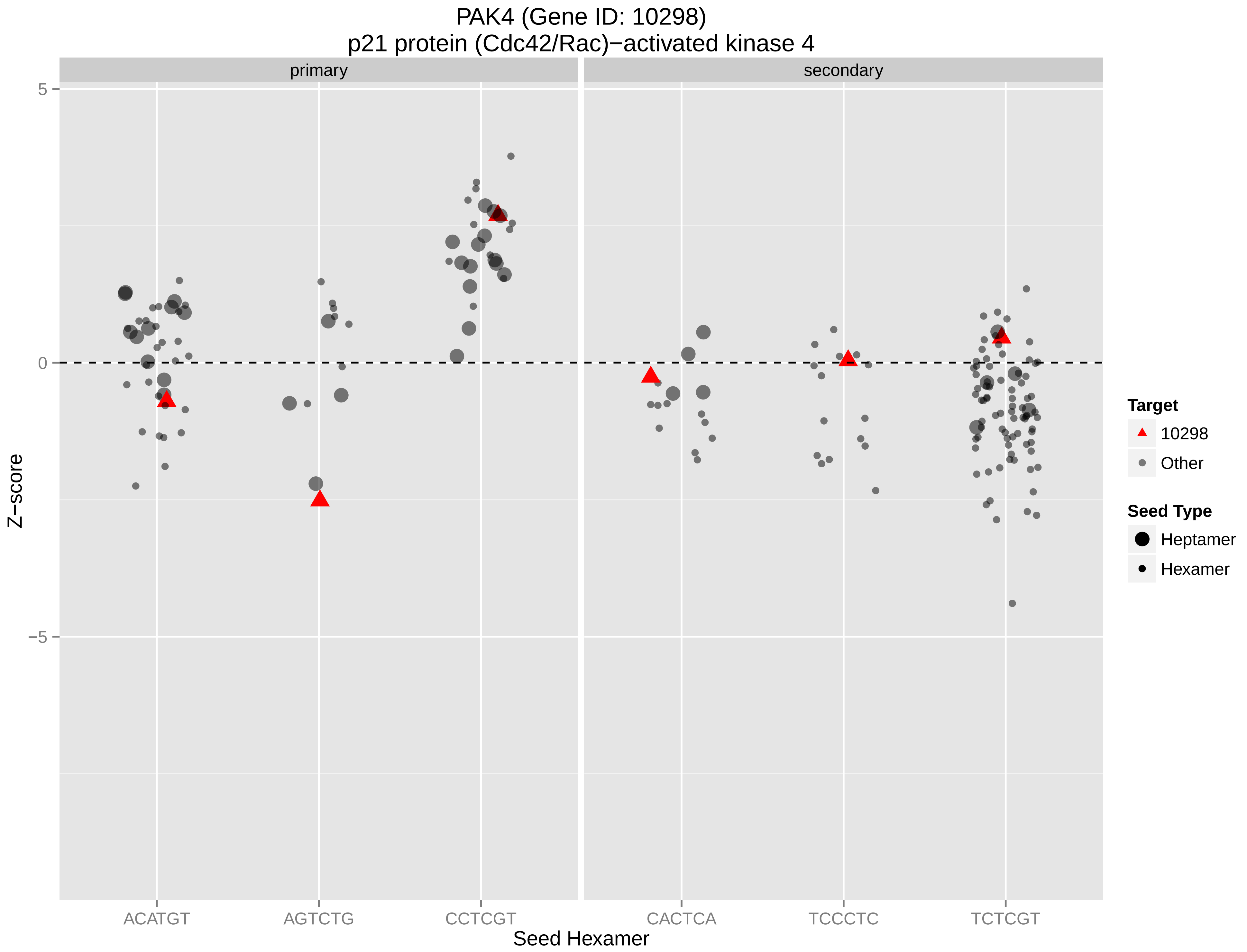
PAK4 (Gene ID: 10298)
p21 protein (Cdc42/Rac)−activated kinase 4
primary
secondary
5
0
−5
Z−score
ACATGT
AGTCTG
CCTCGT
CACTCA
TCCCTC
TCTCGT
Seed Hexamer
Target
10298
Other
Seed Type
Heptamer
Hexamer

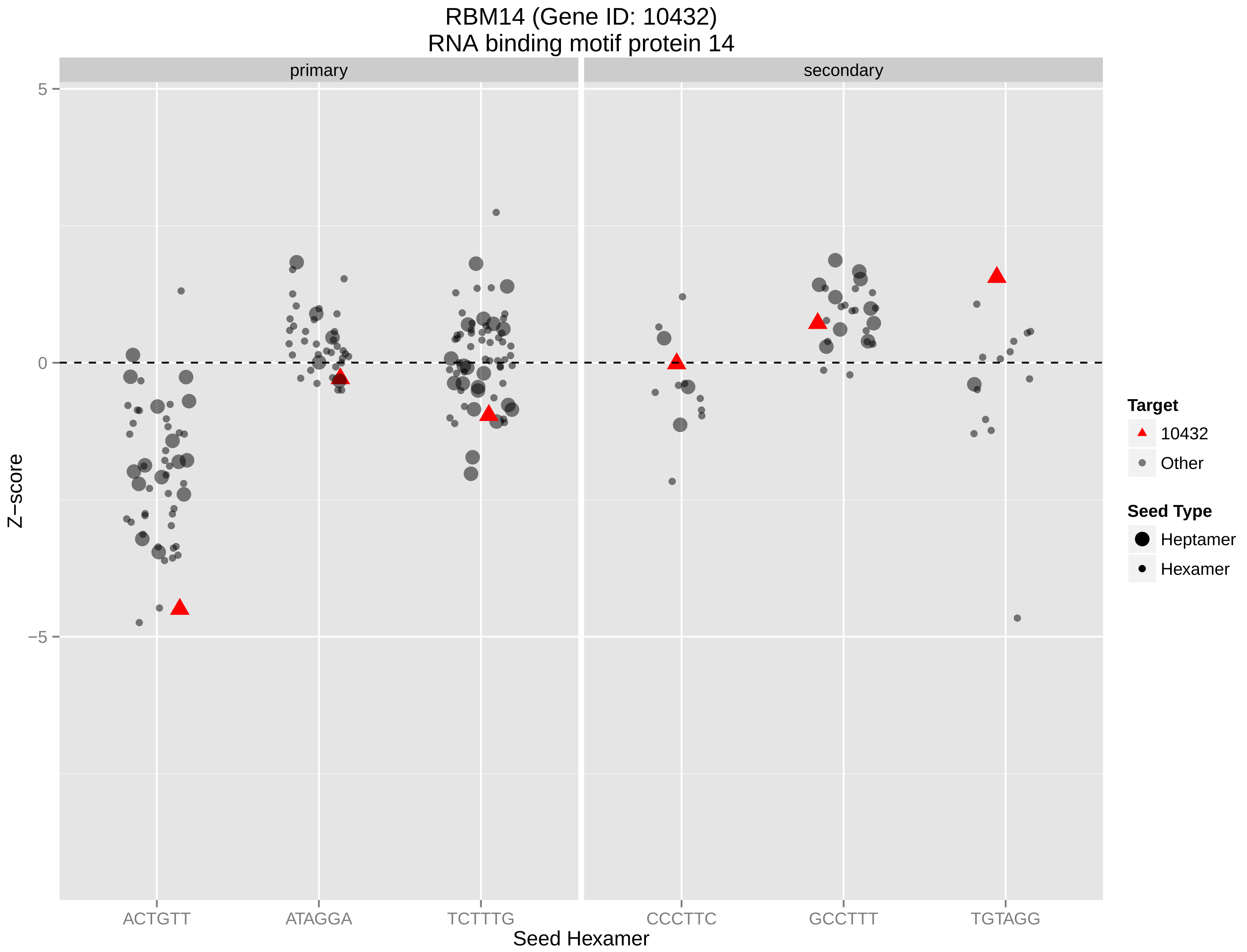
RBM14 (Gene ID: 10432)
RNA binding motif protein 14
primary
secondary
5
0
−5
Z−score
ACTGTT
ATAGGA
TCTTTG
CCCTTC
GCCTTT
TGTAGG
Seed Hexamer
Target
10432
Other
Seed Type
Heptamer
Hexamer

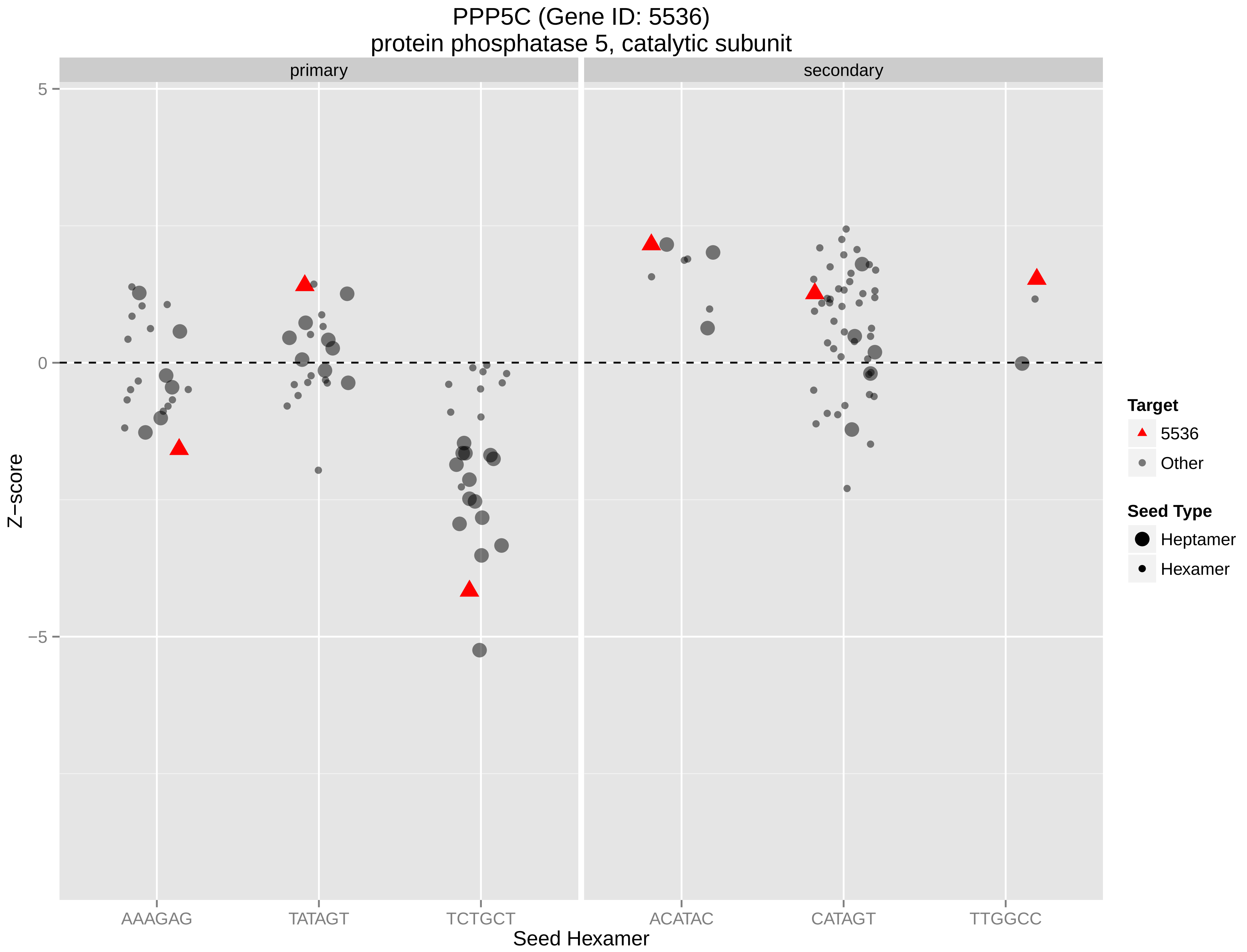

PPP5C (Gene ID: 5536)
protein phosphatase 5, catalytic subunit
primary
secondary
5
0
−5
Z−score
AAAGAG
TATAGT
TCTGCT
ACATAC
CATAGT
TTGGCC
Seed Hexamer
Target
5536
Other
Seed Type
Heptamer
Hexamer

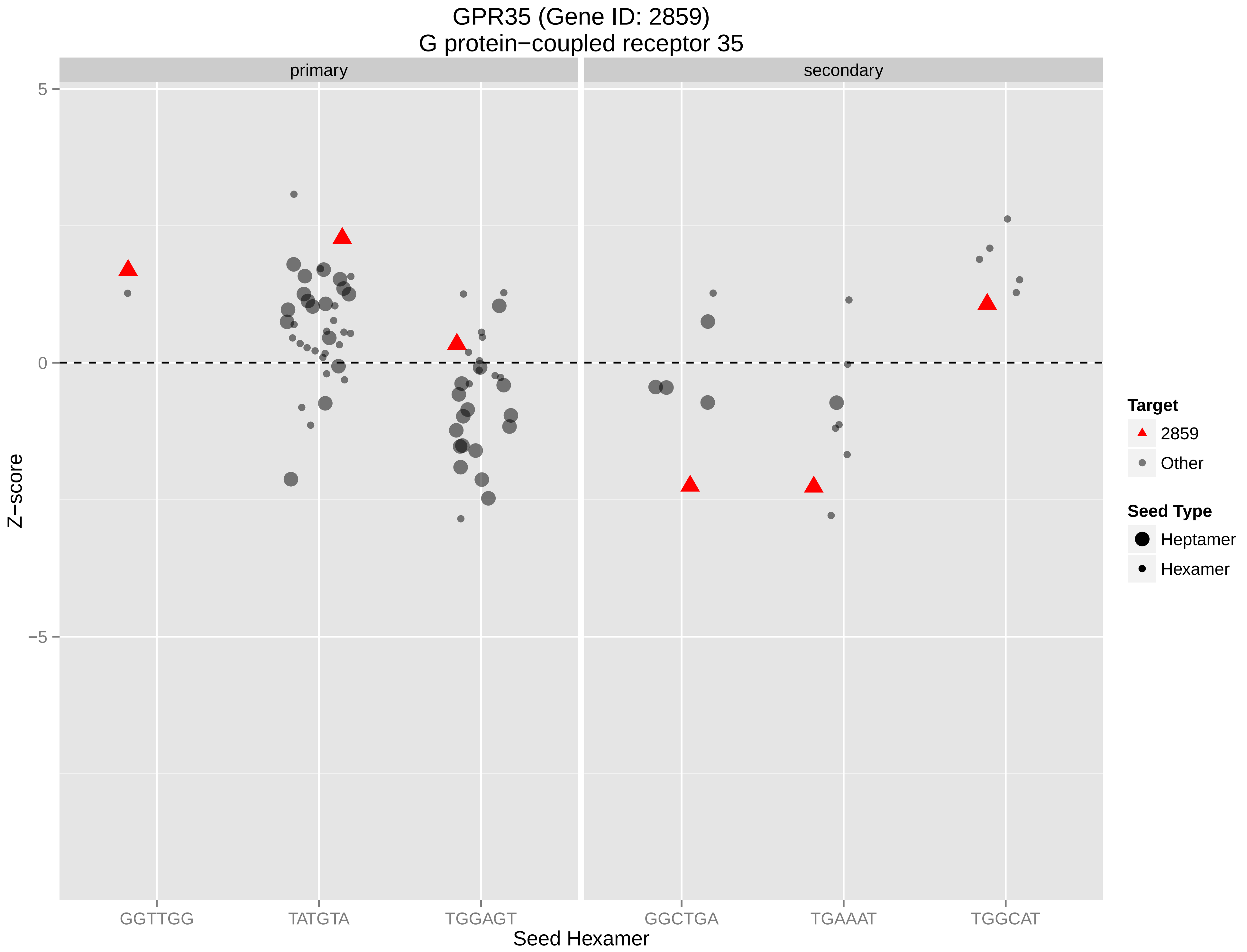

GPR35 (Gene ID: 2859)
G protein−coupled receptor 35
primary
secondary
5
0
−5
Z−score
GGTTGG
TATGTA
TGGAGT
GGCTGA
TGAAAT
TGGCAT
Seed Hexamer
Target
2859
Other
Seed Type
Heptamer
Hexamer

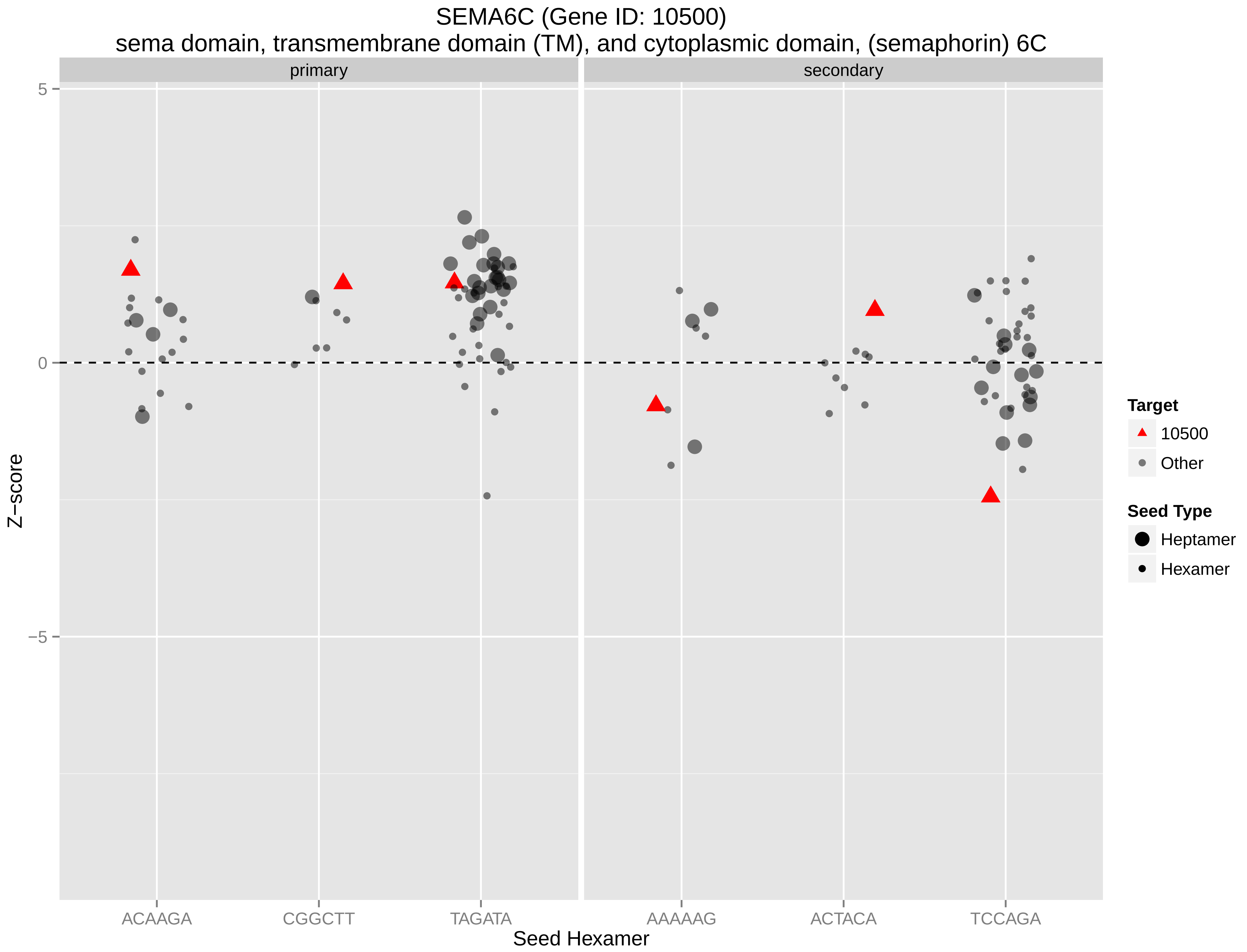
SEMA6C (Gene ID: 10500)
sema domain, transmembrane domain (TM), and cytoplasmic domain, (semaphorin) 6C
primary
secondary
5
0
−5
Z−score
ACAAGA
CGGCTT
TAGATA
AAAAAG
ACTACA
TCCAGA
Seed Hexamer
Target
10500
Other
Seed Type
Heptamer
Hexamer

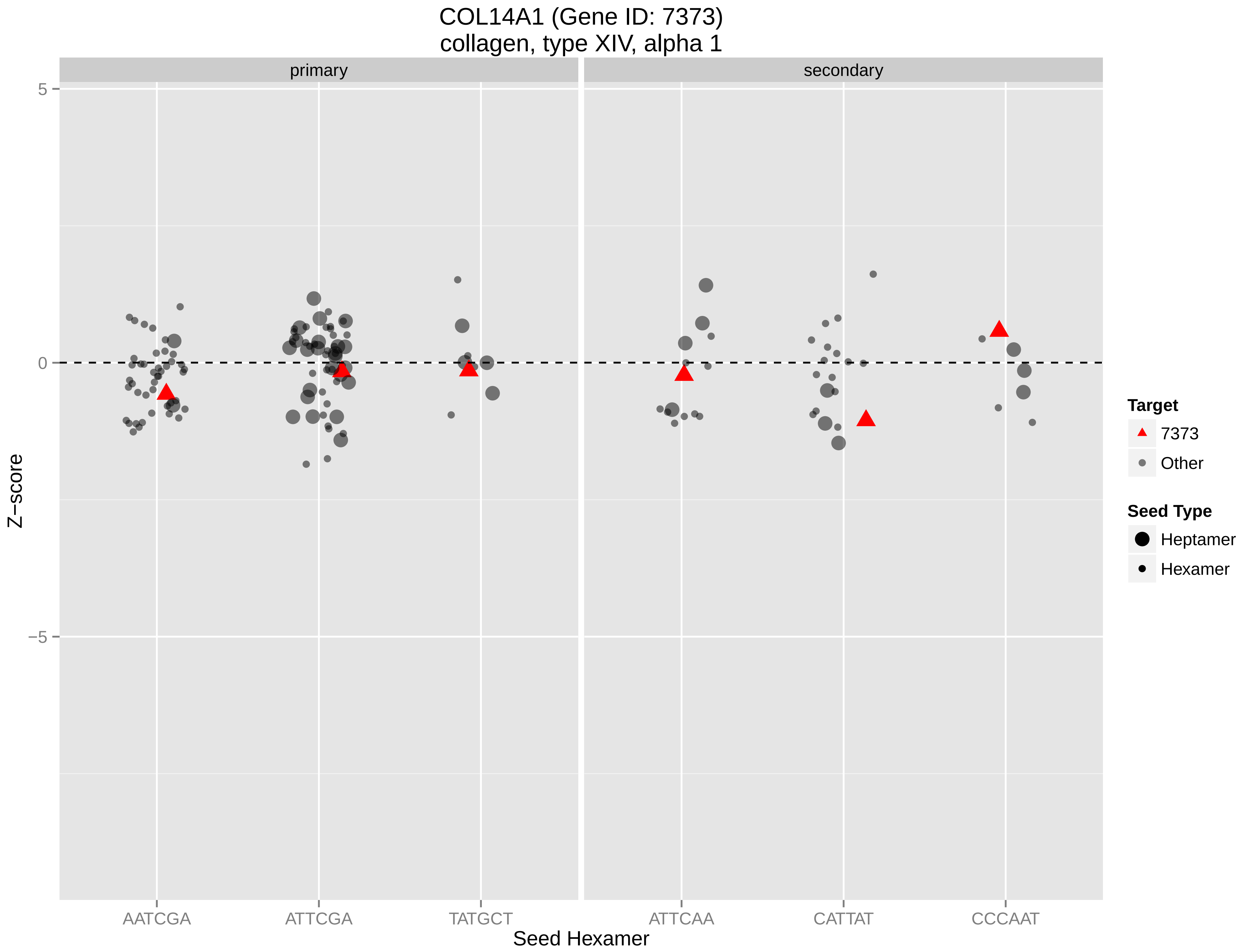

COL14A1 (Gene ID: 7373)
collagen, type XIV, alpha 1
primary
secondary
5
0
−5
Z−score
AATCGA
ATTCGA
TATGCT
ATTCAA
CATTAT
CCCAAT
Seed Hexamer
Target
7373
Other
Seed Type
Heptamer
Hexamer

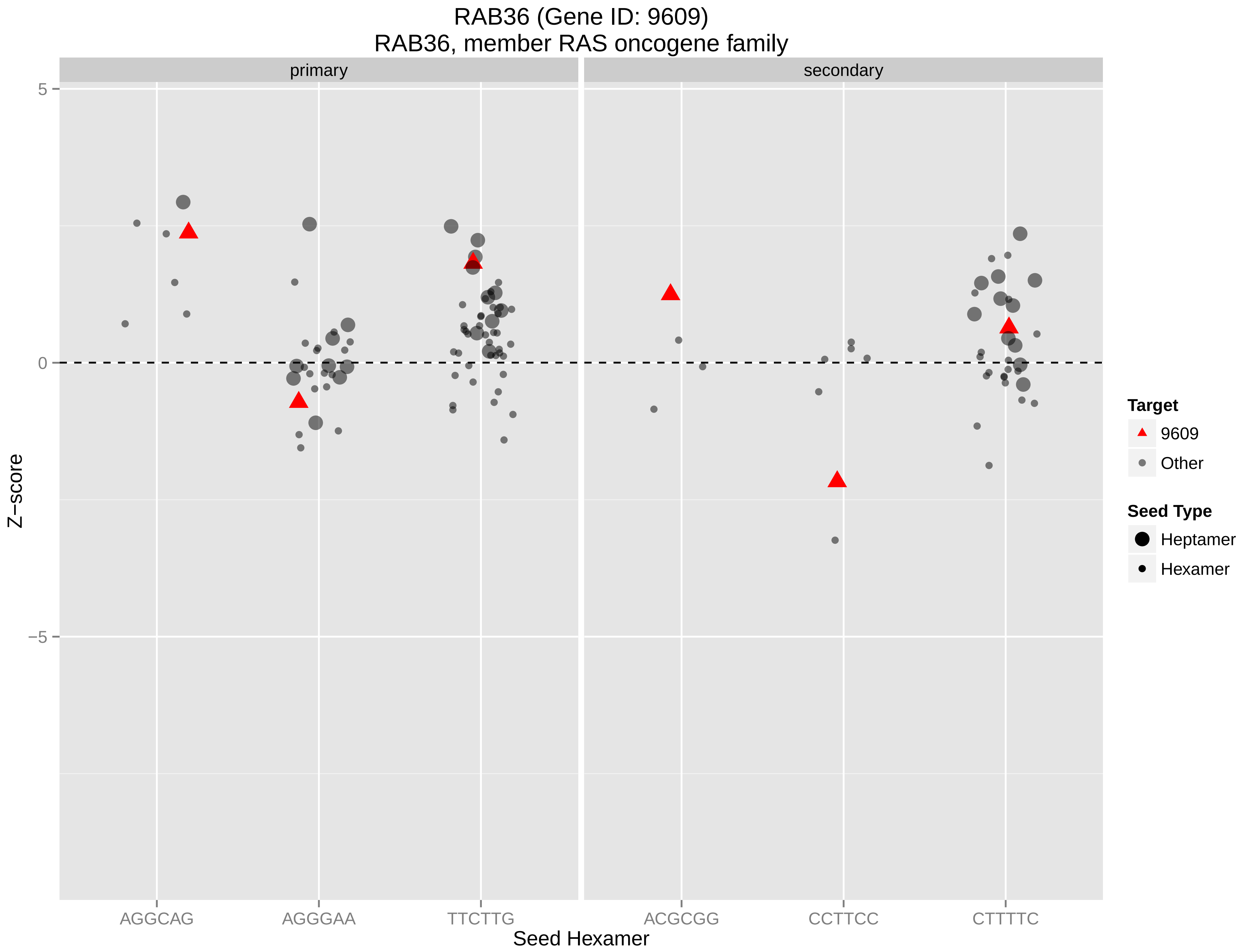

RAB36 (Gene ID: 9609)
RAB36, member RAS oncogene family
primary
secondary
5
0
−5
Z−score
AGGCAG
AGGGAA
TTCTTG
ACGCGG
CCTTCC
CTTTTC
Seed Hexamer
Target
9609
Other
Seed Type
Heptamer
Hexamer

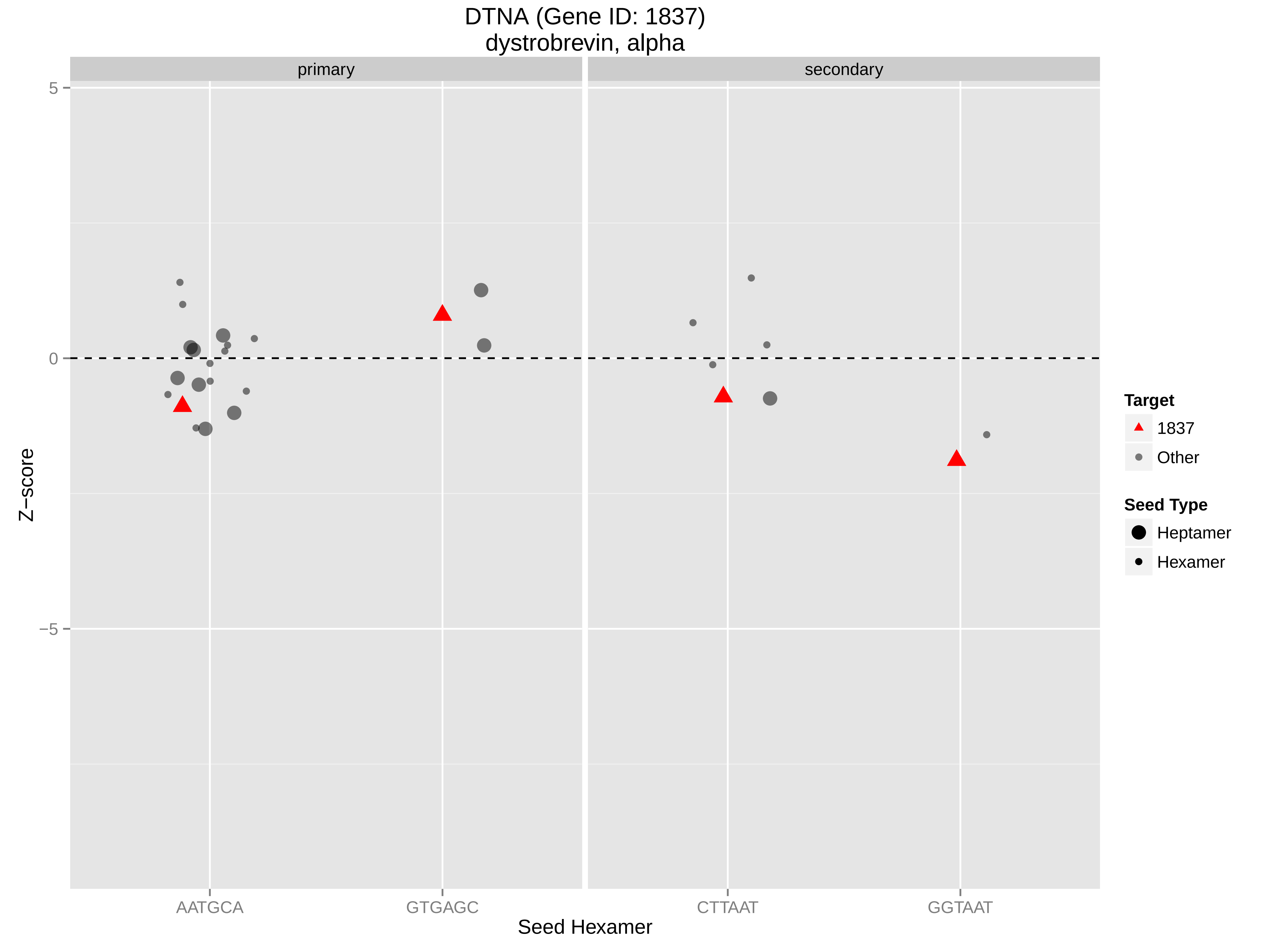
DTNA (Gene ID: 1837)
dystrobrevin, alpha
primary
secondary
5
0
−5
Z−score
AATGCA
GTGAGC
CTTAAT
GGTAAT
Seed Hexamer
Target
1837
Other
Seed Type
Heptamer
Hexamer

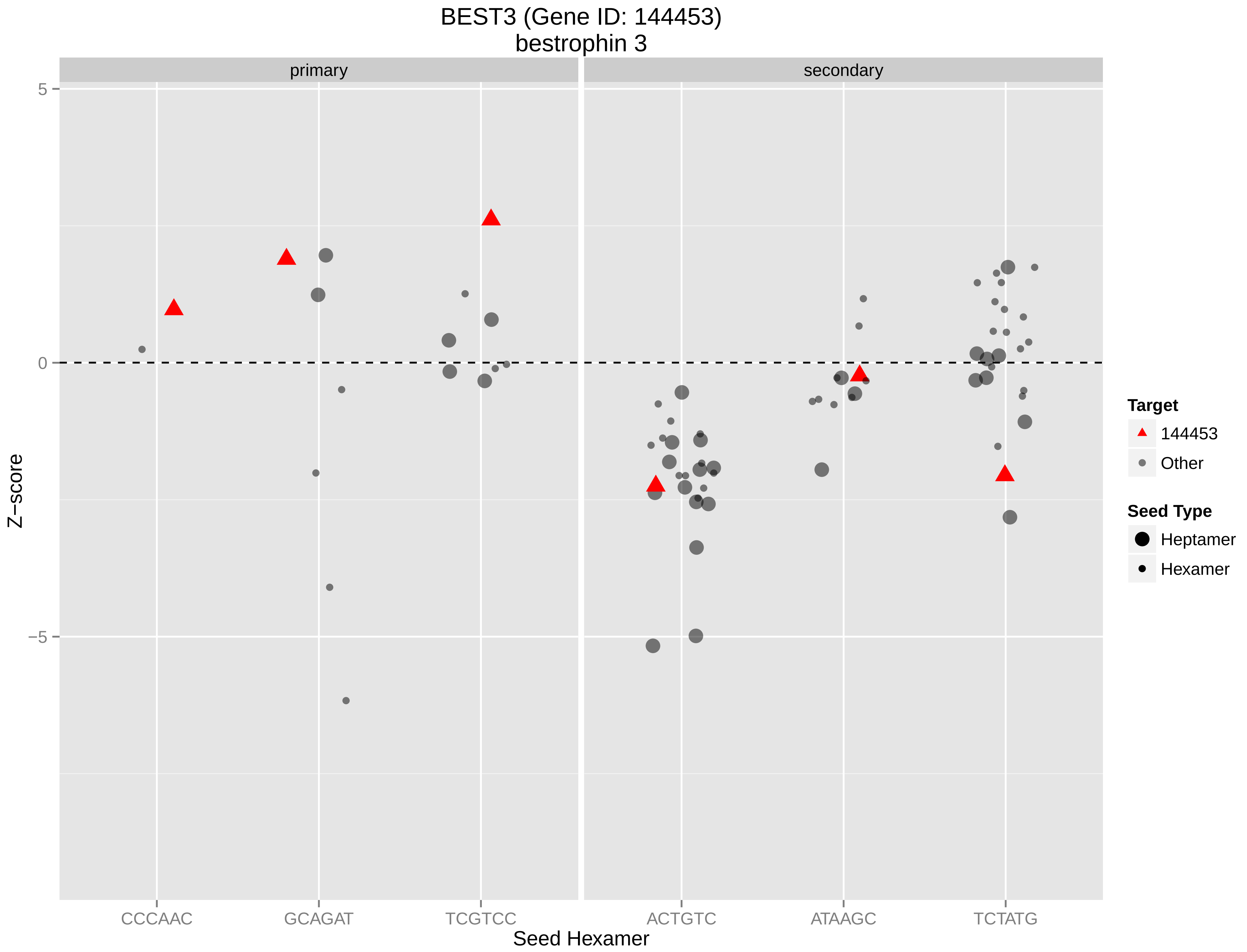
BEST3 (Gene ID: 144453)
bestrophin 3
primary
secondary
5
0
−5
Z−score
CCCAAC
GCAGAT
TCGTCC
ACTGTC
ATAAGC
TCTATG
Seed Hexamer
Target
144453
Other
Seed Type
Heptamer
Hexamer

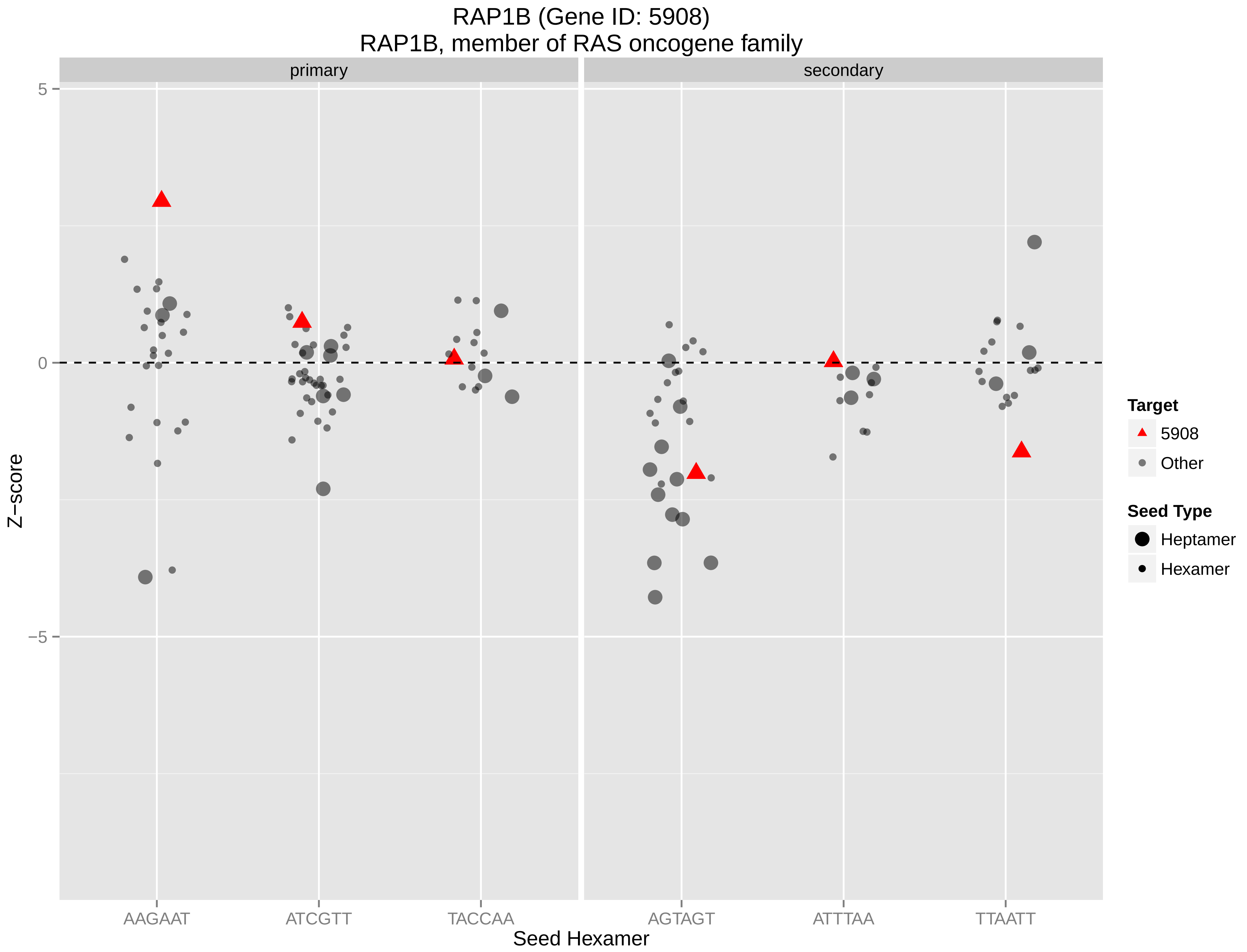

RAP1B (Gene ID: 5908)
RAP1B, member of RAS oncogene family
primary
secondary
Z−score
5
0
−5
AAGAAT
ATCGTT
TACCAA
AGTAGT
ATTTAA
TTAATT
Seed Hexamer
Target
5908
Other
Seed Type
Heptamer
Hexamer

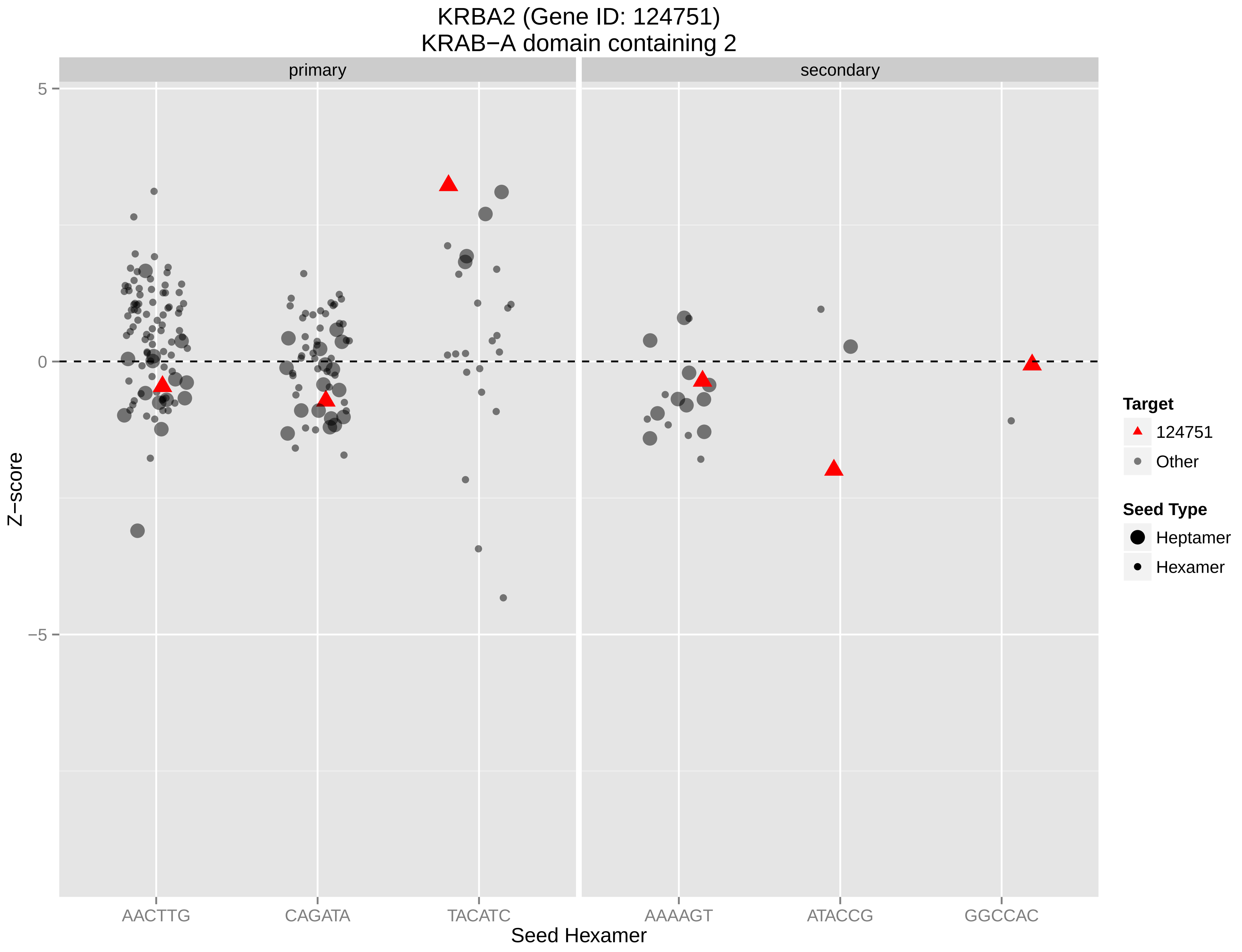
KRBA2 (Gene ID: 124751)
KRAB−A domain containing 2
primary
secondary
5
0
−5
Z−score
AACTTG
CAGATA
TACATC
AAAAGT
ATACCG
GGCCAC
Seed Hexamer
Target
124751
Other
Seed Type
Heptamer
Hexamer

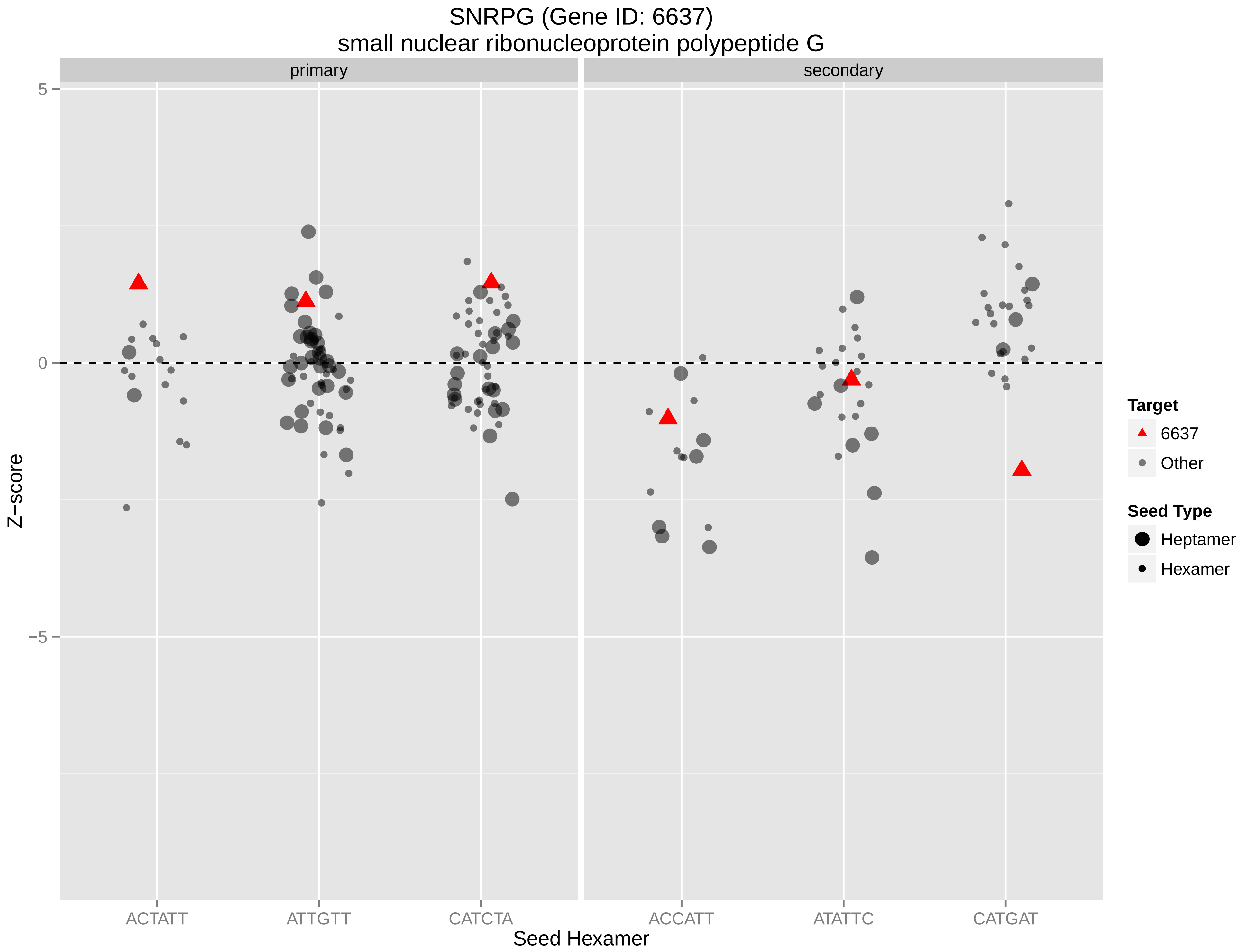

SNRPG (Gene ID: 6637)
small nuclear ribonucleoprotein polypeptide G
primary
secondary
5
0
−5
Z−score
ACTATT
ATTGTT
CATCTA
ACCATT
ATATTC
CATGAT
Seed Hexamer
Target
6637
Other
Seed Type
Heptamer
Hexamer

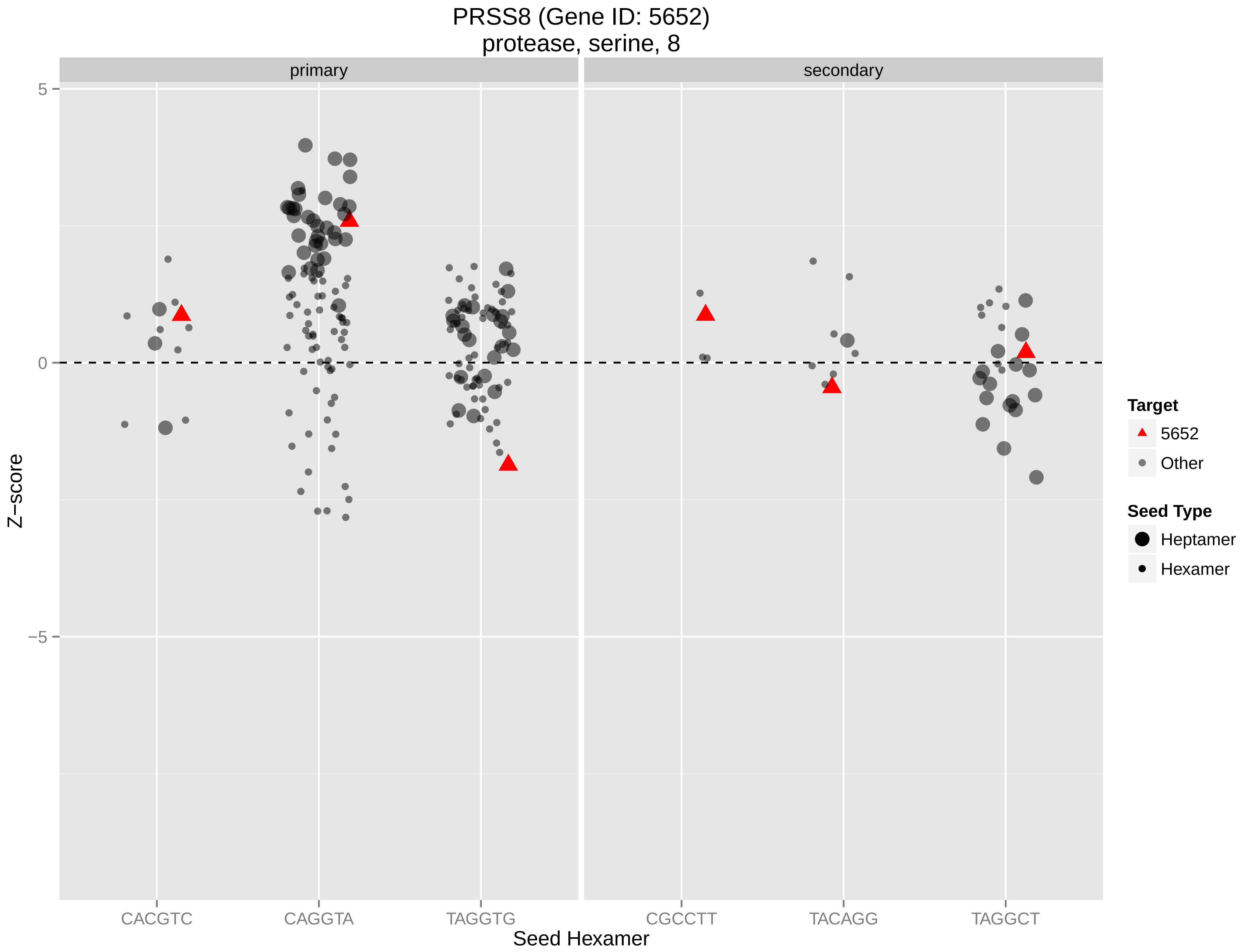

PRSS8 (Gene ID: 5652)
protease, serine, 8
primary
secondary
5
0
−5
Z−score
CACGTC
CAGGTA
TAGGTG
CGCCTT
TACAGG
TAGGCT
Seed Hexamer
Target
5652
Other
Seed Type
Heptamer
Hexamer

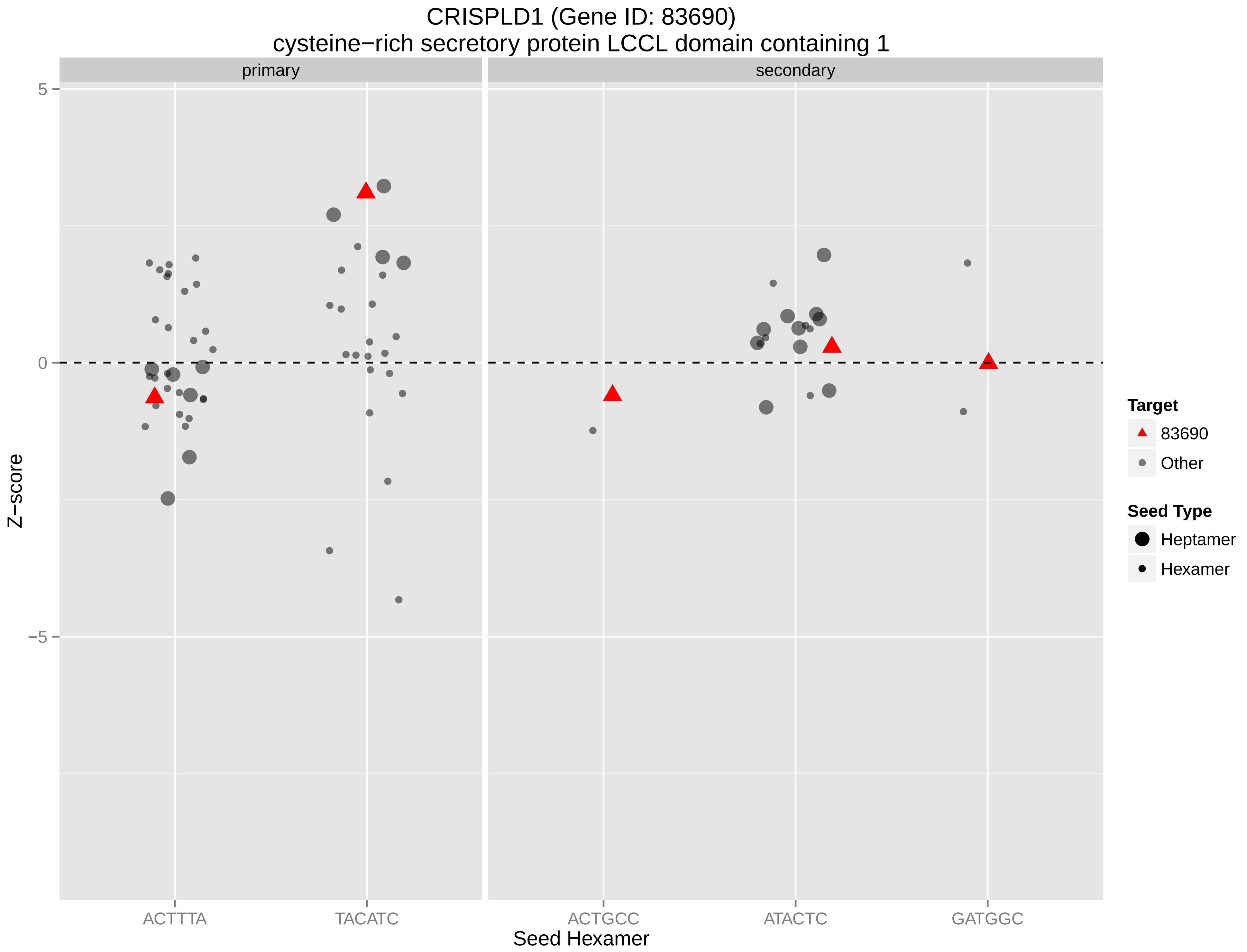

CRISPLD1 (Gene ID: 83690)
cysteine−rich secretory protein LCCL domain containing 1
primary
secondary
5
0
−5
Z−score
ACTTTA
TACATC
ACTGCC
ATACTC
GATGGC
Seed Hexamer
Target
83690
Other
Seed Type
Heptamer
Hexamer

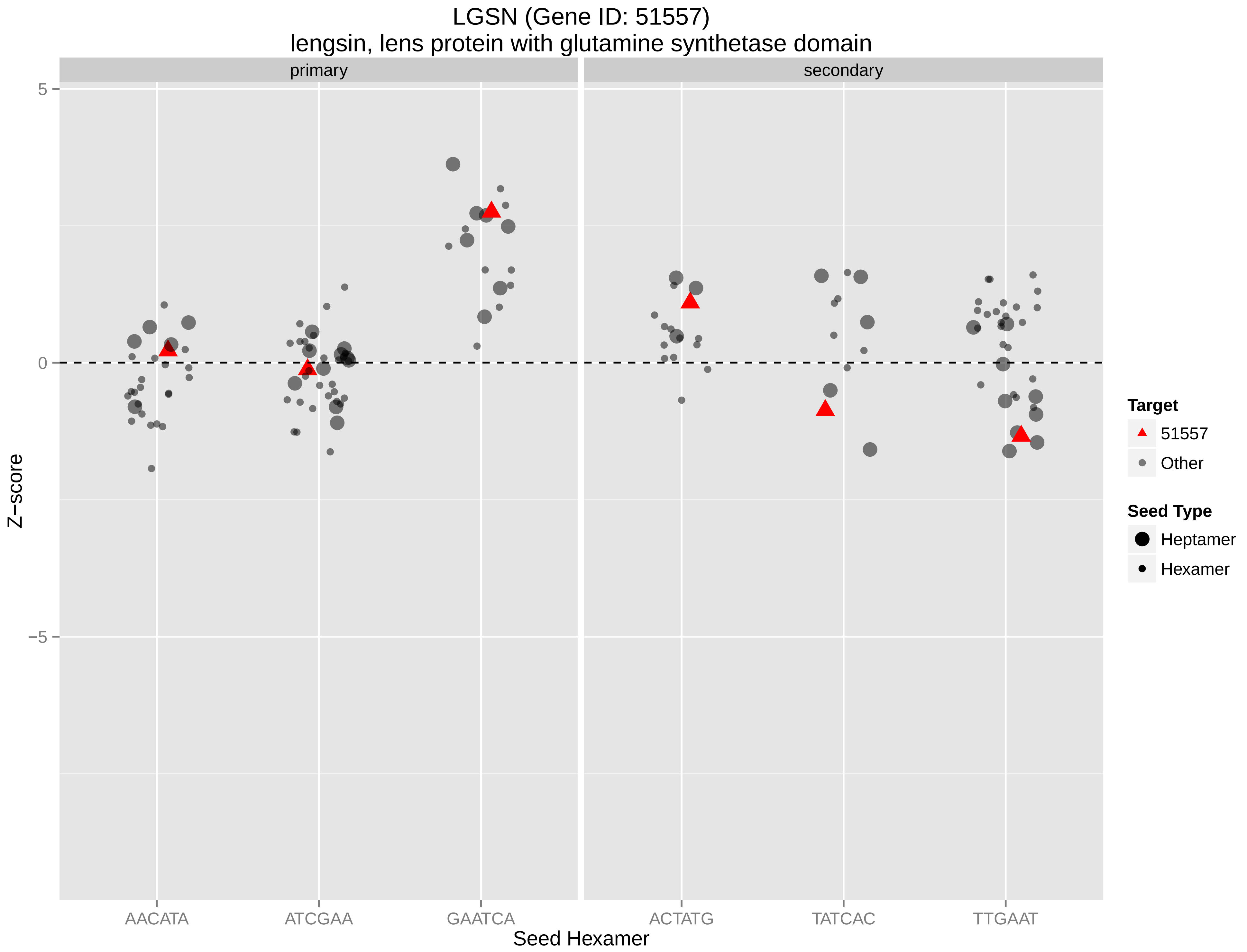

LGSN (Gene ID: 51557)
lengsin, lens protein with glutamine synthetase domain
primary
secondary
5
0
−5
Z−score
AACATA
ATCGAA
GAATCA
ACTATG
TATCAC
TTGAAT
Seed Hexamer
Target
51557
Other
Seed Type
Heptamer
Hexamer

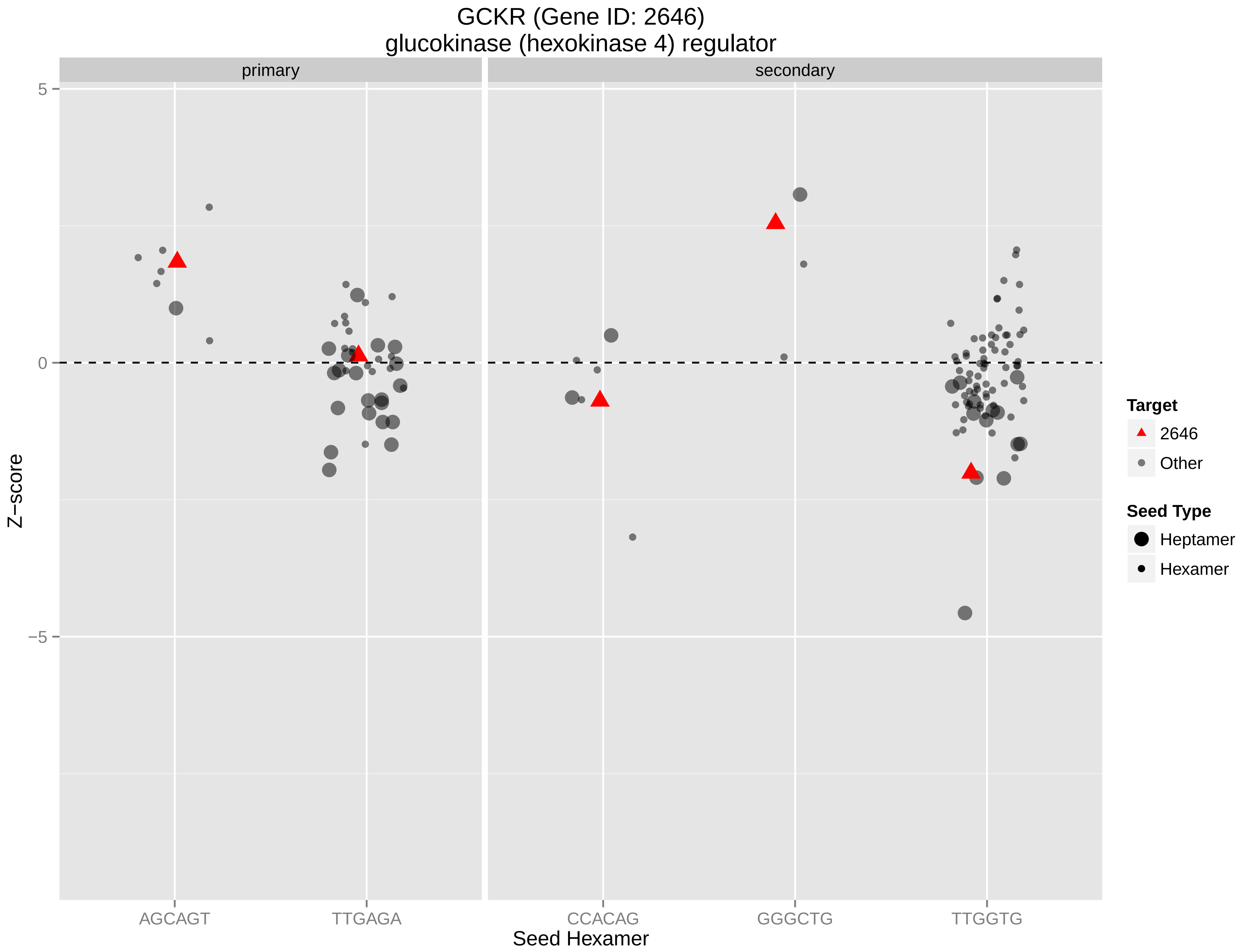

GCKR (Gene ID: 2646)
glucokinase (hexokinase 4) regulator
primary
secondary
5
0
−5
Z−score
AGCAGT
TTGAGA
CCACAG
GGGCTG
TTGGTG
Seed Hexamer
Target
2646
Other
Seed Type
Heptamer
Hexamer

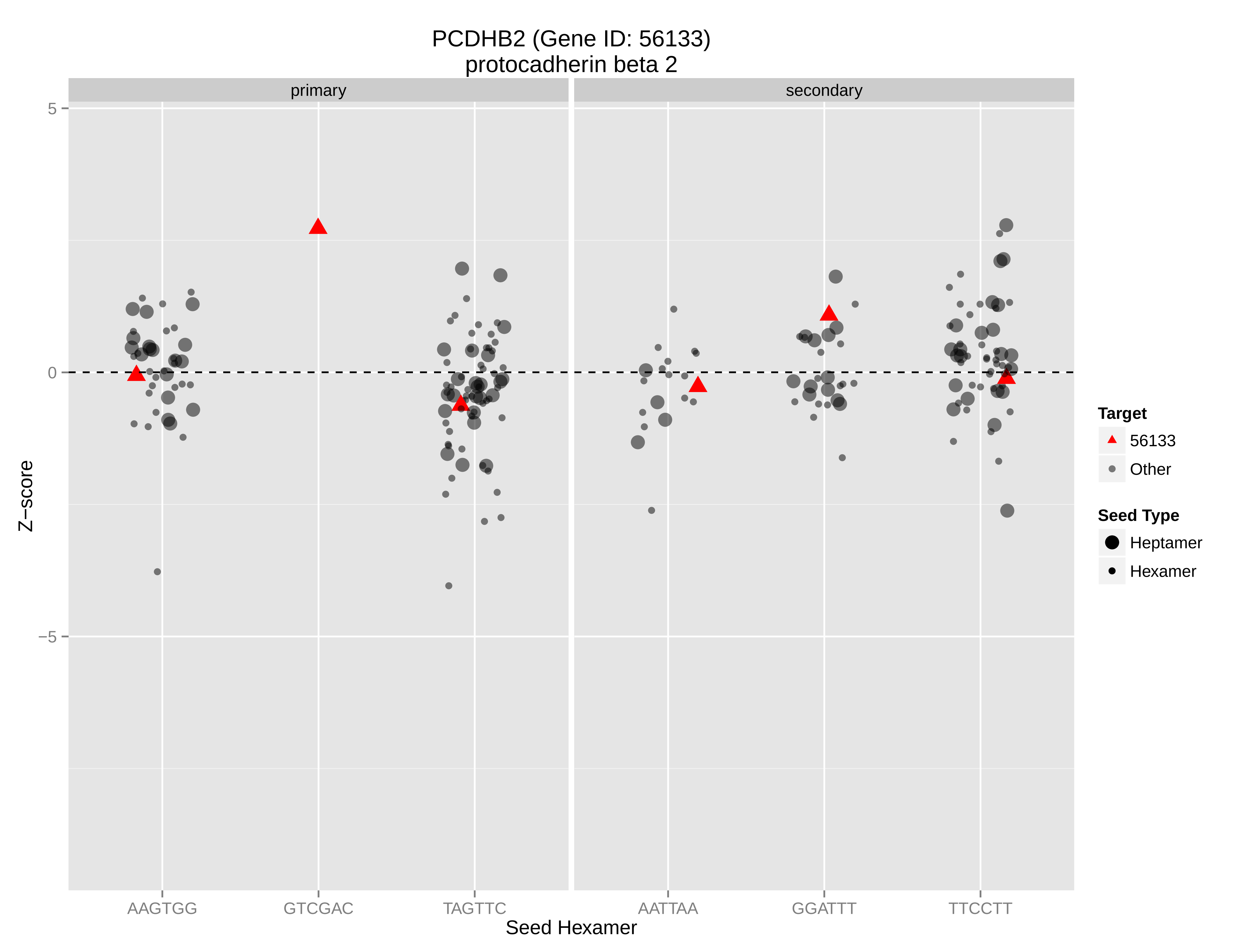
PCDHB2 (Gene ID: 56133)
protocadherin beta 2
primary
secondary
5
0
−5
Z−score
AAGTGG
GTCGAC
TAGTTC
AATTAA
GGATTT
TTCCTT
Seed Hexamer
Target
56133
Other
Seed Type
Heptamer
Hexamer

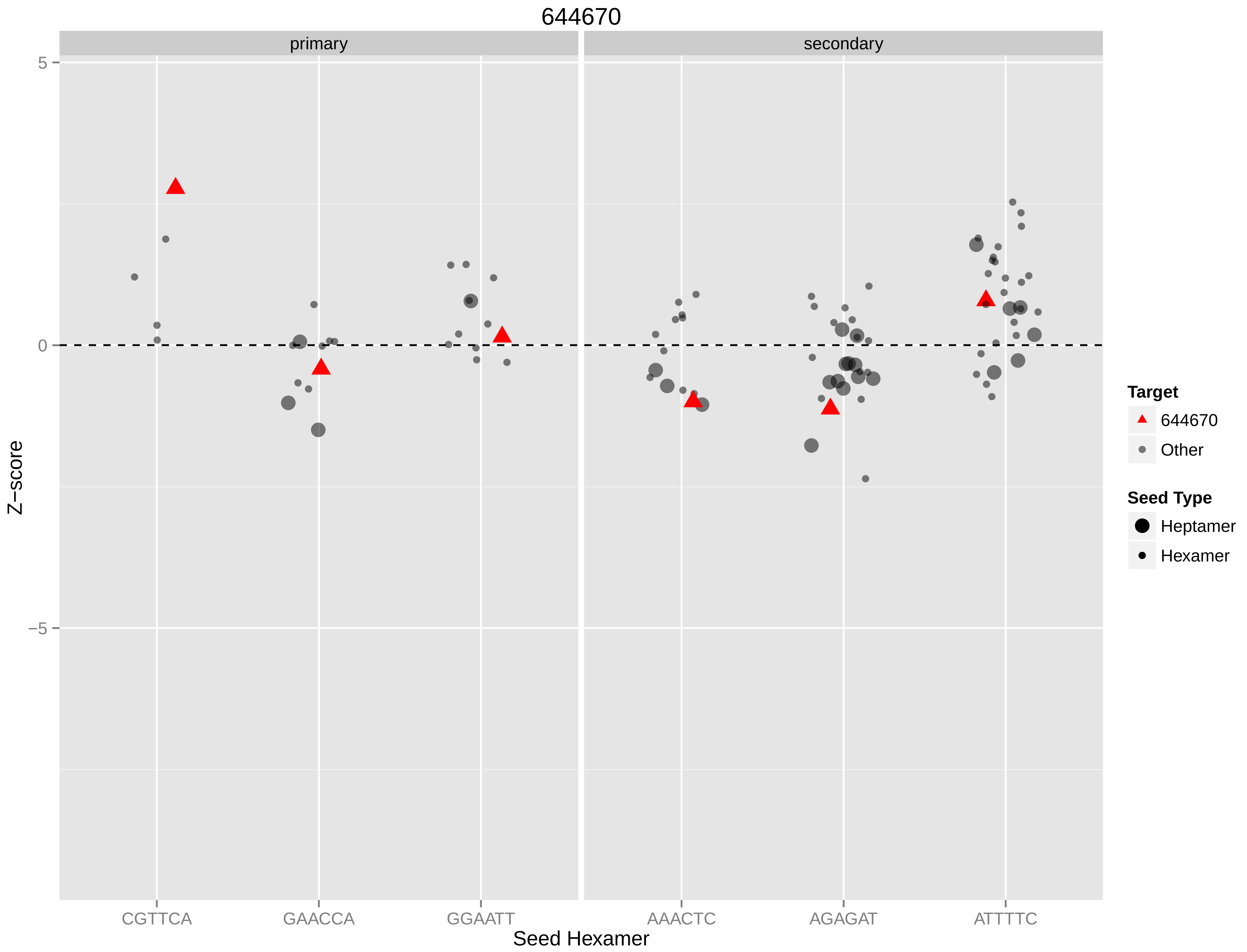

644670
primary
secondary
Z−score
5
0
−5
CGTTCA
GAACCA
GGAATT
AAACTC
AGAGAT
ATTTTC
Seed Hexamer
Target
644670
Other
Seed Type
Heptamer
Hexamer

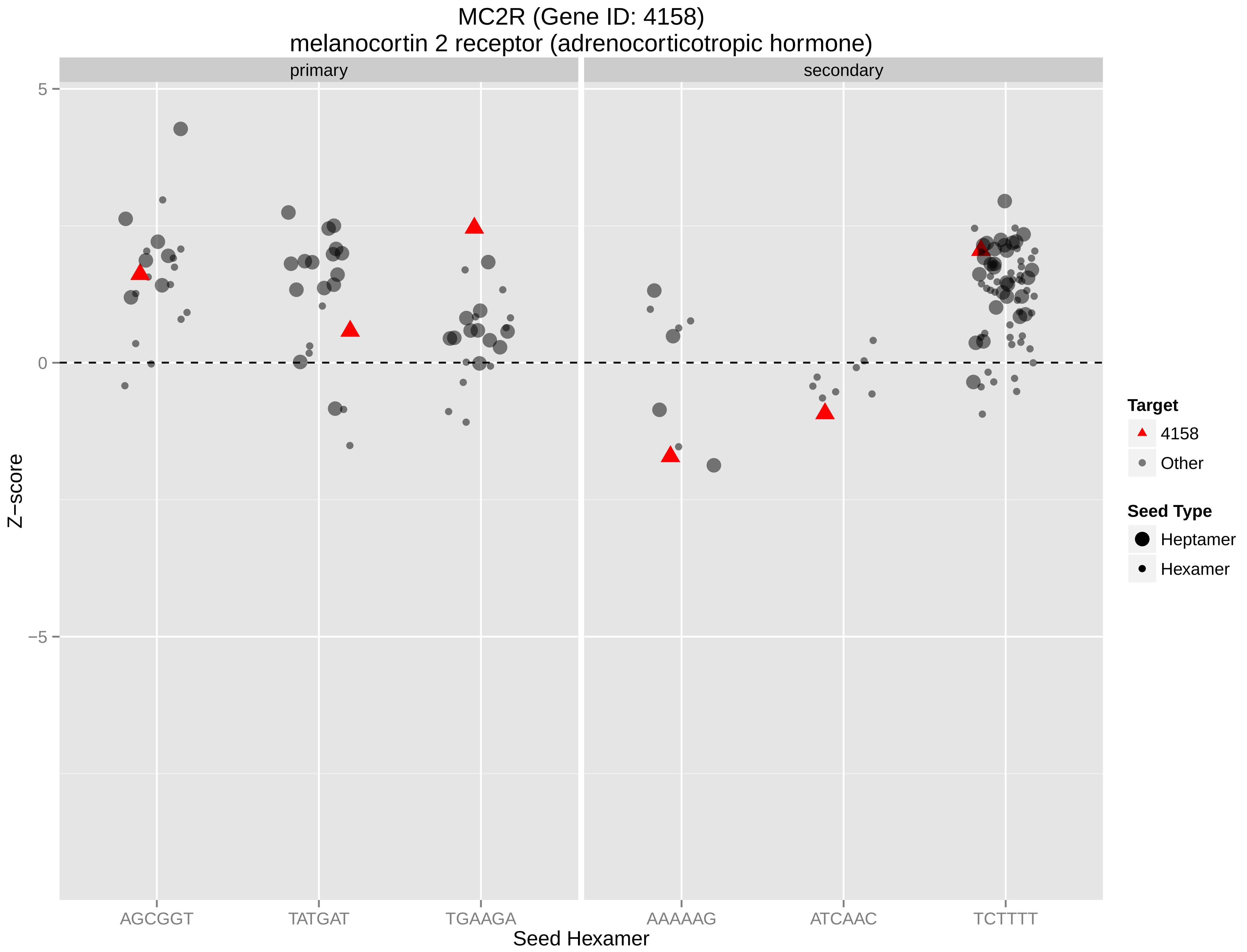

MC2R (Gene ID: 4158)
melanocortin 2 receptor (adrenocorticotropic hormone)
primary
secondary
5
0
−5
Z−score
AGCGGT
TATGAT
TGAAGA
AAAAAG
ATCAAC
TCTTTT
Seed Hexamer
Target
4158
Other
Seed Type
Heptamer
Hexamer

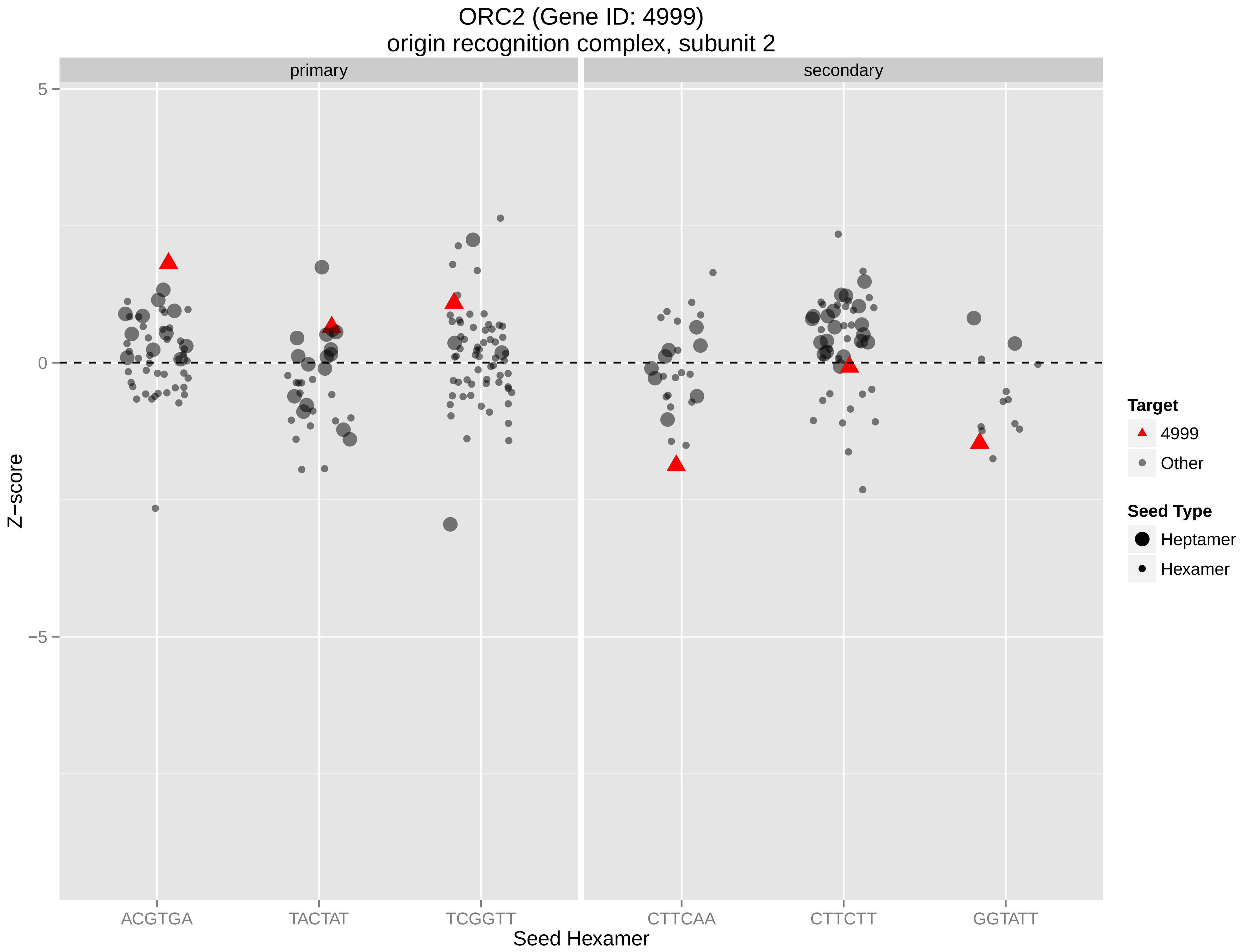
ORC2 (Gene ID: 4999)
origin recognition complex, subunit 2
primary
secondary
5
0
−5
Z−score
ACGTGA
TACTAT
TCGGTT
CTTCAA
CTTCTT
GGTATT
Seed Hexamer
Target
4999
Other
Seed Type
Heptamer
Hexamer

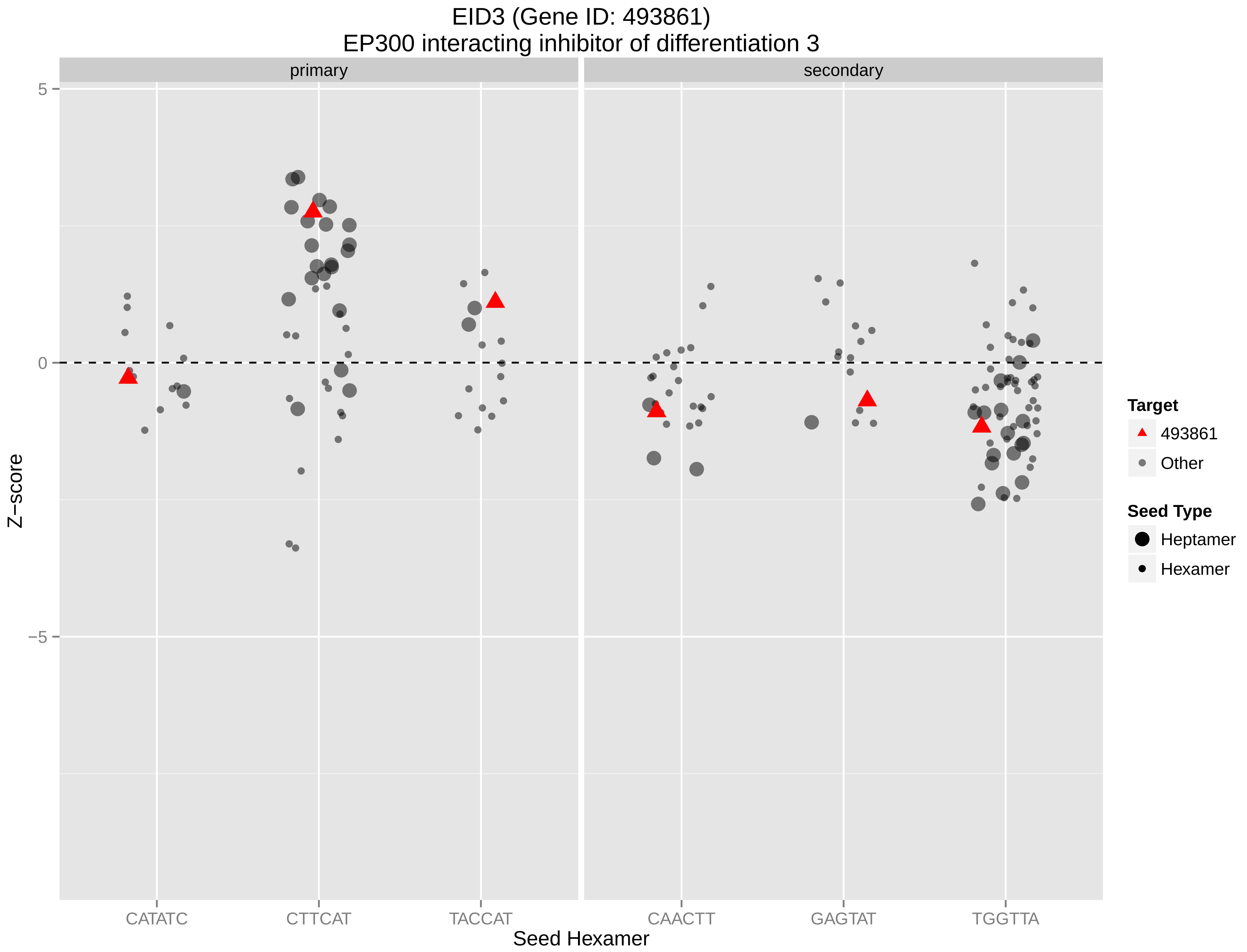
EID3 (Gene ID: 493861)
EP300 interacting inhibitor of differentiation 3
primary
secondary
5
0
−5
Z−score
CATATC
CTTCAT
TACCAT
CAACTT
GAGTAT
TGGTTA
Seed Hexamer
Target
493861
Other
Seed Type
Heptamer
Hexamer

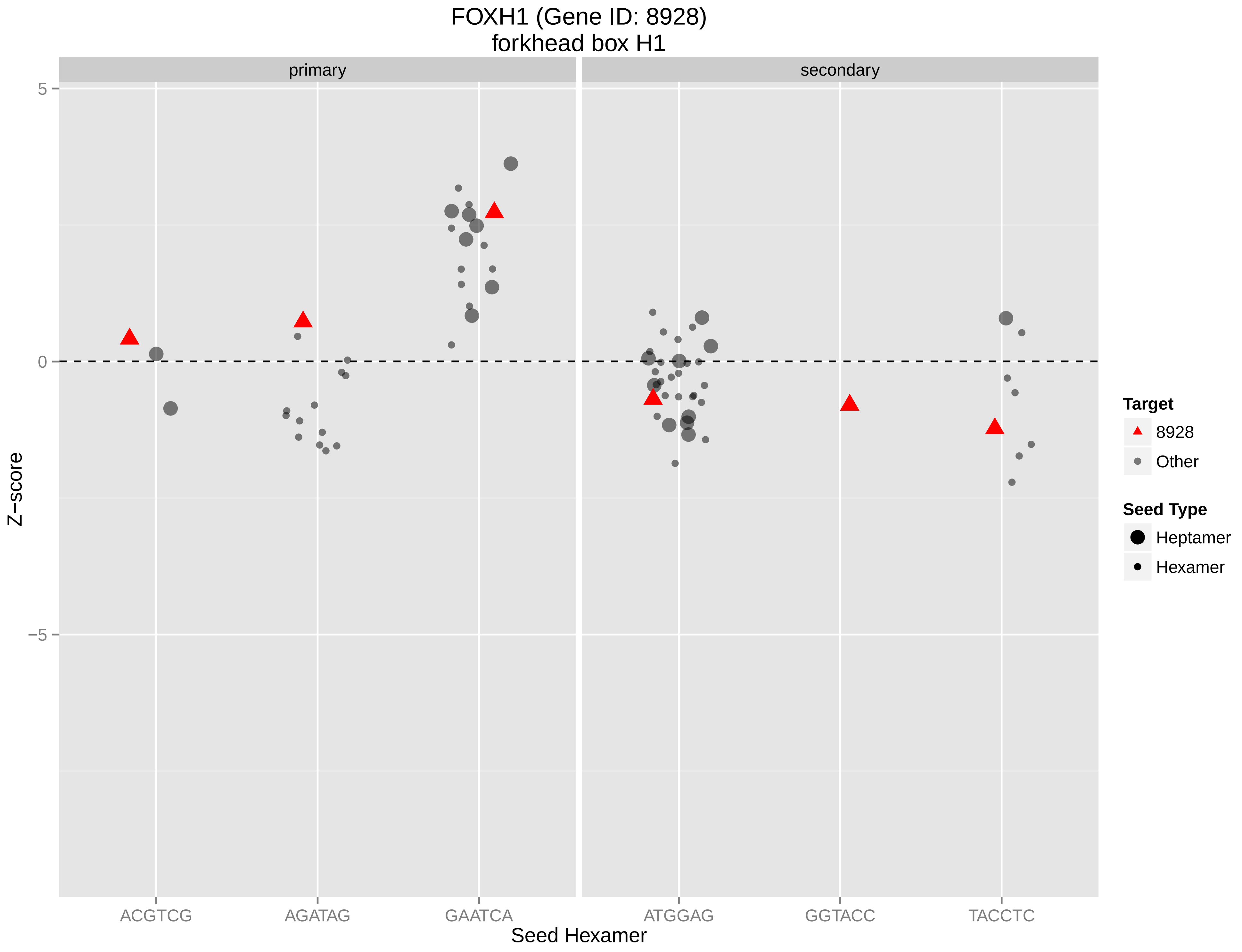
FOXH1 (Gene ID: 8928)
forkhead box H1
primary
secondary
5
0
−5
Z−score
ACGTCG
AGATAG
GAATCA
ATGGAG
GGTACC
TACCTC
Seed Hexamer
Target
8928
Other
Seed Type
Heptamer
Hexamer

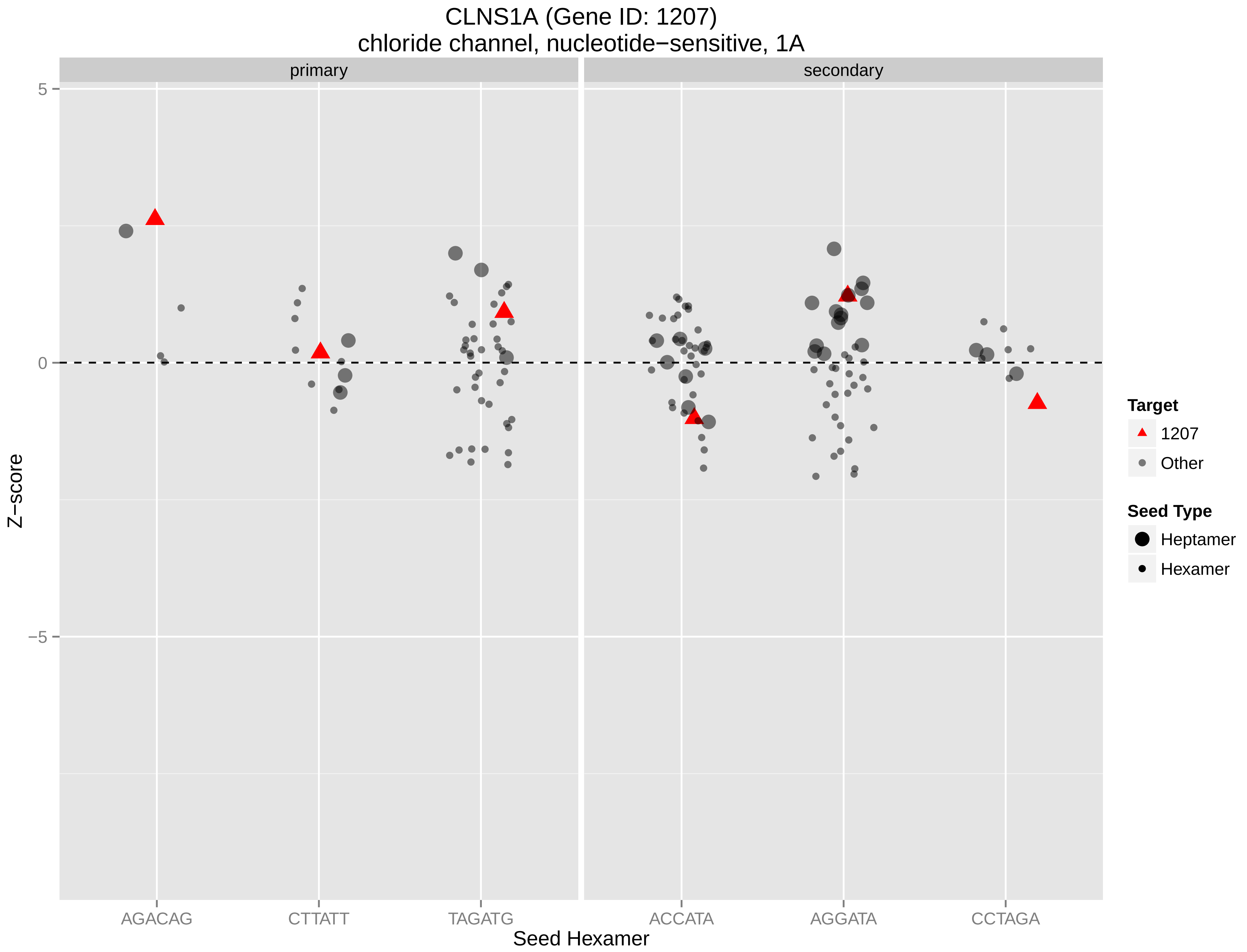

CLNS1A (Gene ID: 1207)
chloride channel, nucleotide−sensitive, 1A
primary
secondary
5
0
−5
Z−score
AGACAG
CTTATT
TAGATG
ACCATA
AGGATA
CCTAGA
Seed Hexamer
Target
1207
Other
Seed Type
Heptamer
Hexamer

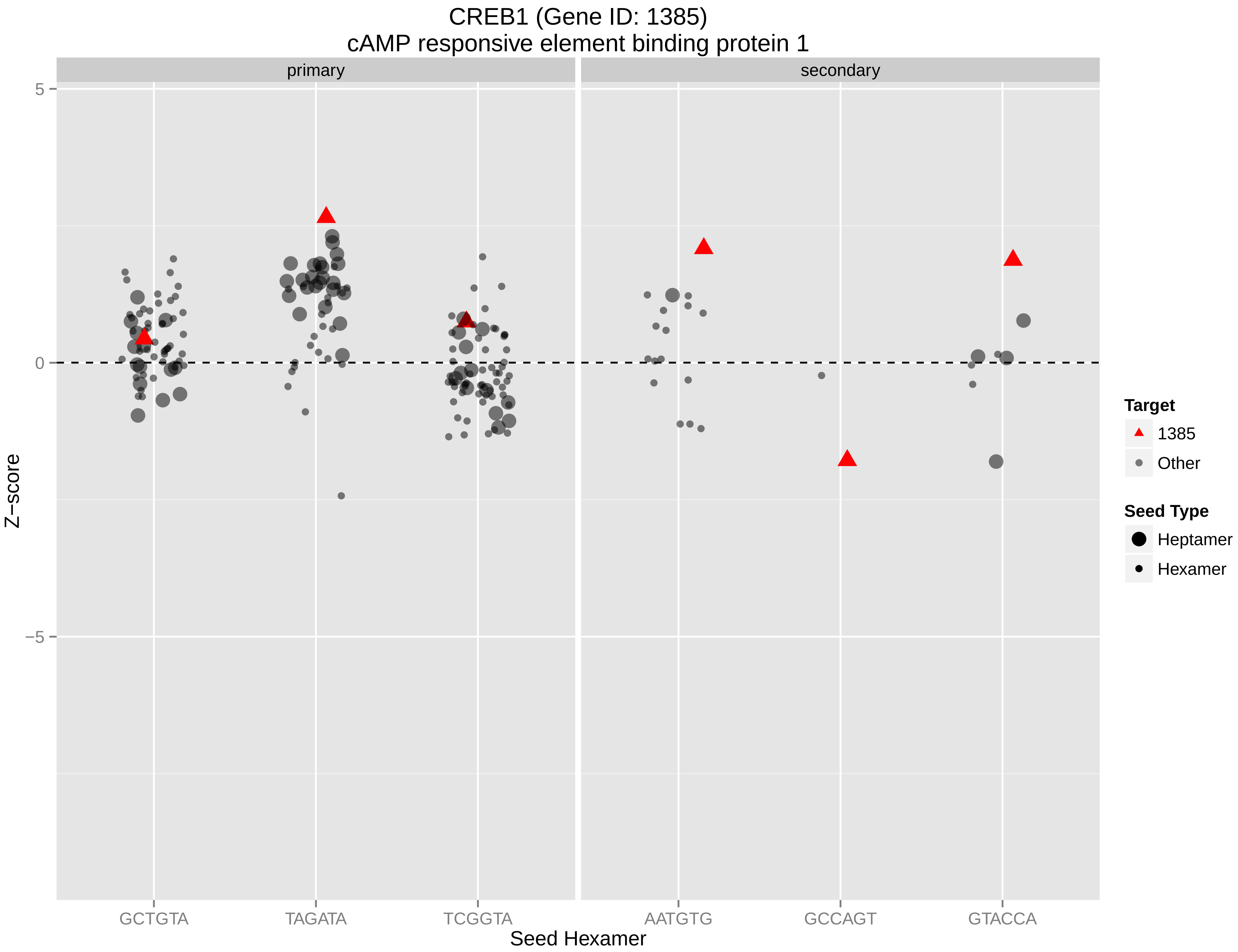

CREB1 (Gene ID: 1385)
cAMP responsive element binding protein 1
primary
secondary
5
0
−5
Z−score
GCTGTA
TAGATA
TCGGTA
AATGTG
GCCAGT
GTACCA
Seed Hexamer
Target
1385
Other
Seed Type
Heptamer
Hexamer

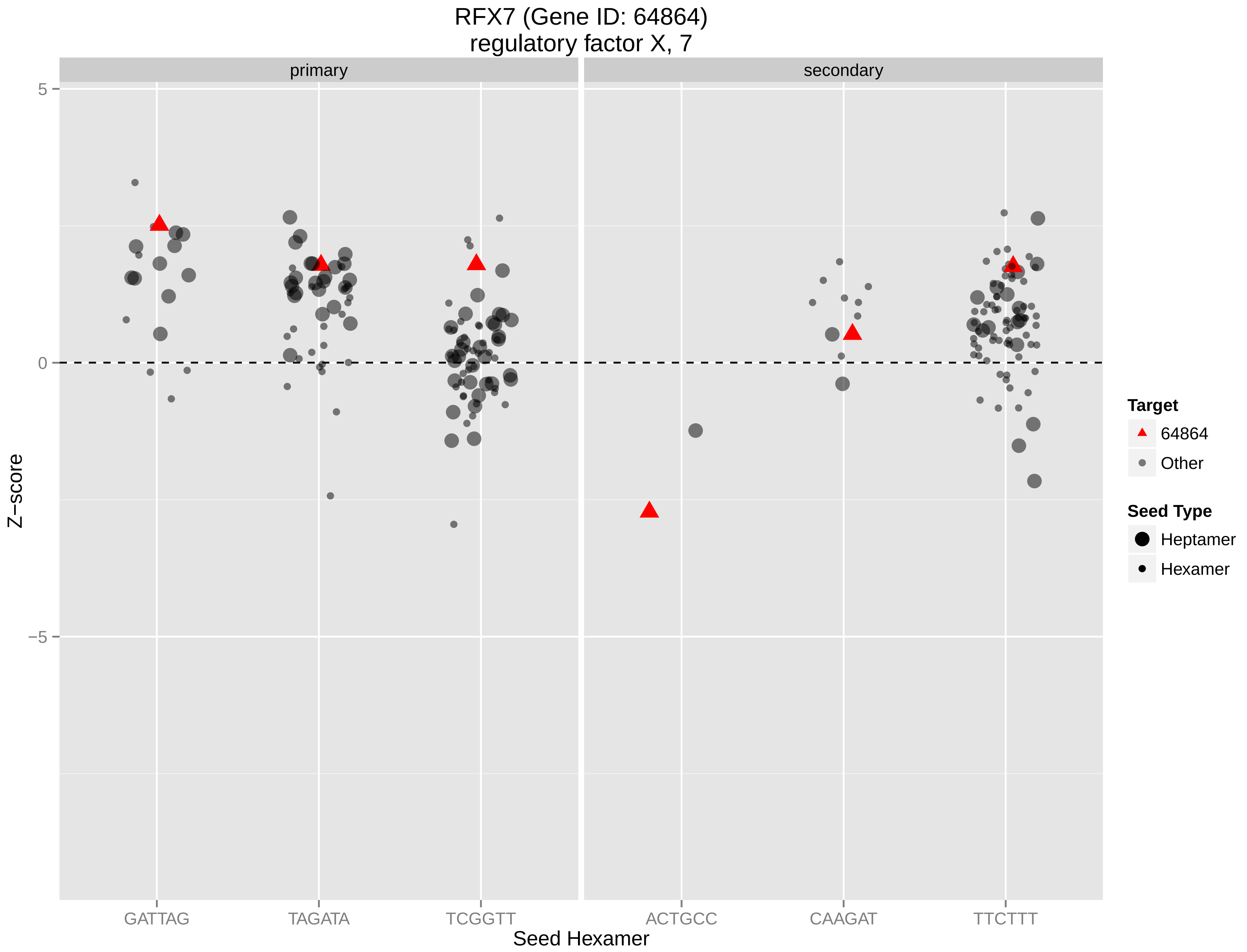
RFX7 (Gene ID: 64864)
regulatory factor X, 7
primary
secondary
5
0
−5
Z−score
GATTAG
TAGATA
TCGGTT
ACTGCC
CAAGAT
TTCTTT
Seed Hexamer
Target
64864
Other
Seed Type
Heptamer
Hexamer

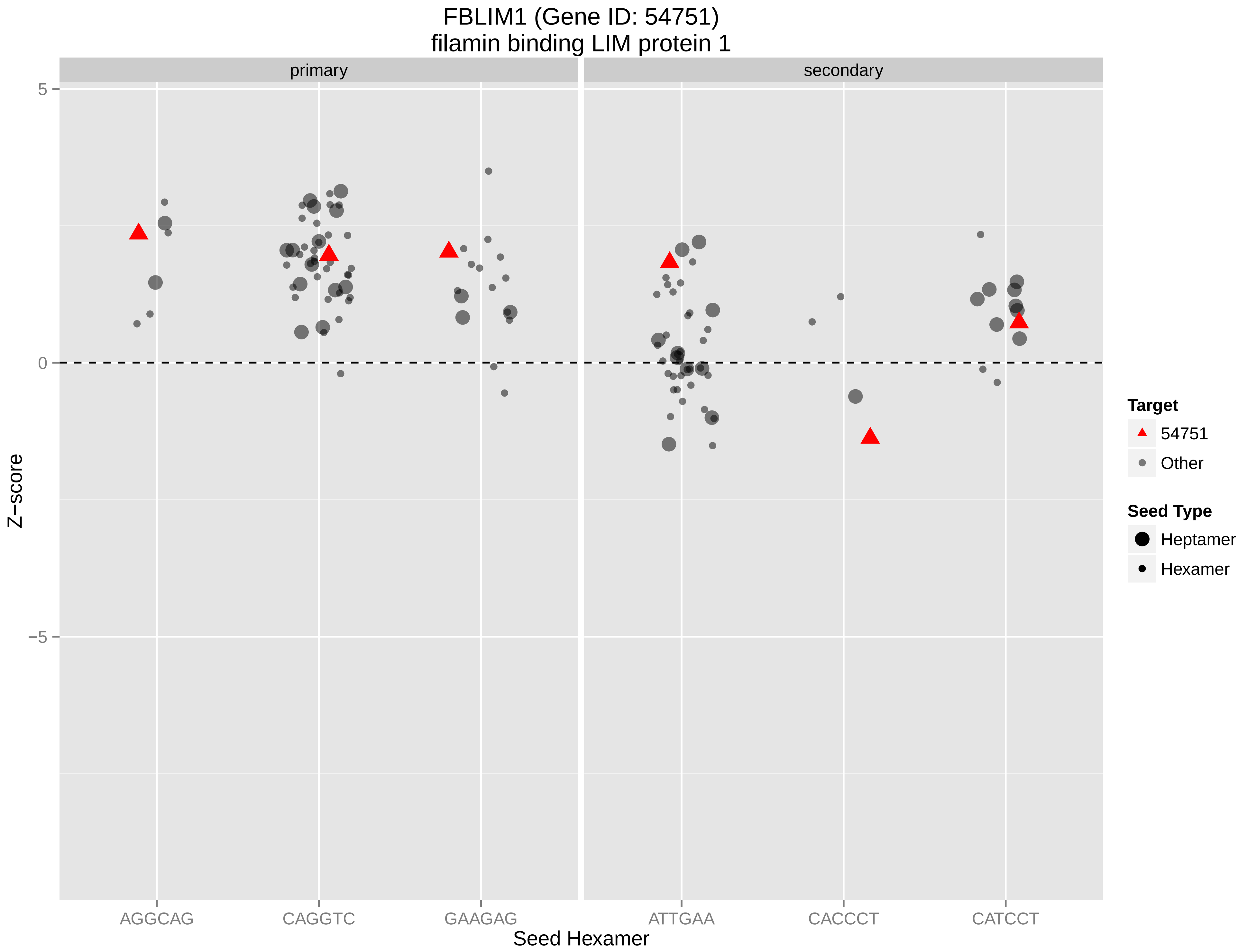
FBLIM1 (Gene ID: 54751)
filamin binding LIM protein 1
primary
secondary
5
0
−5
Z−score
AGGCAG
CAGGTC
GAAGAG
ATTGAA
CACCCT
CATCCT
Seed Hexamer
Target
54751
Other
Seed Type
Heptamer
Hexamer

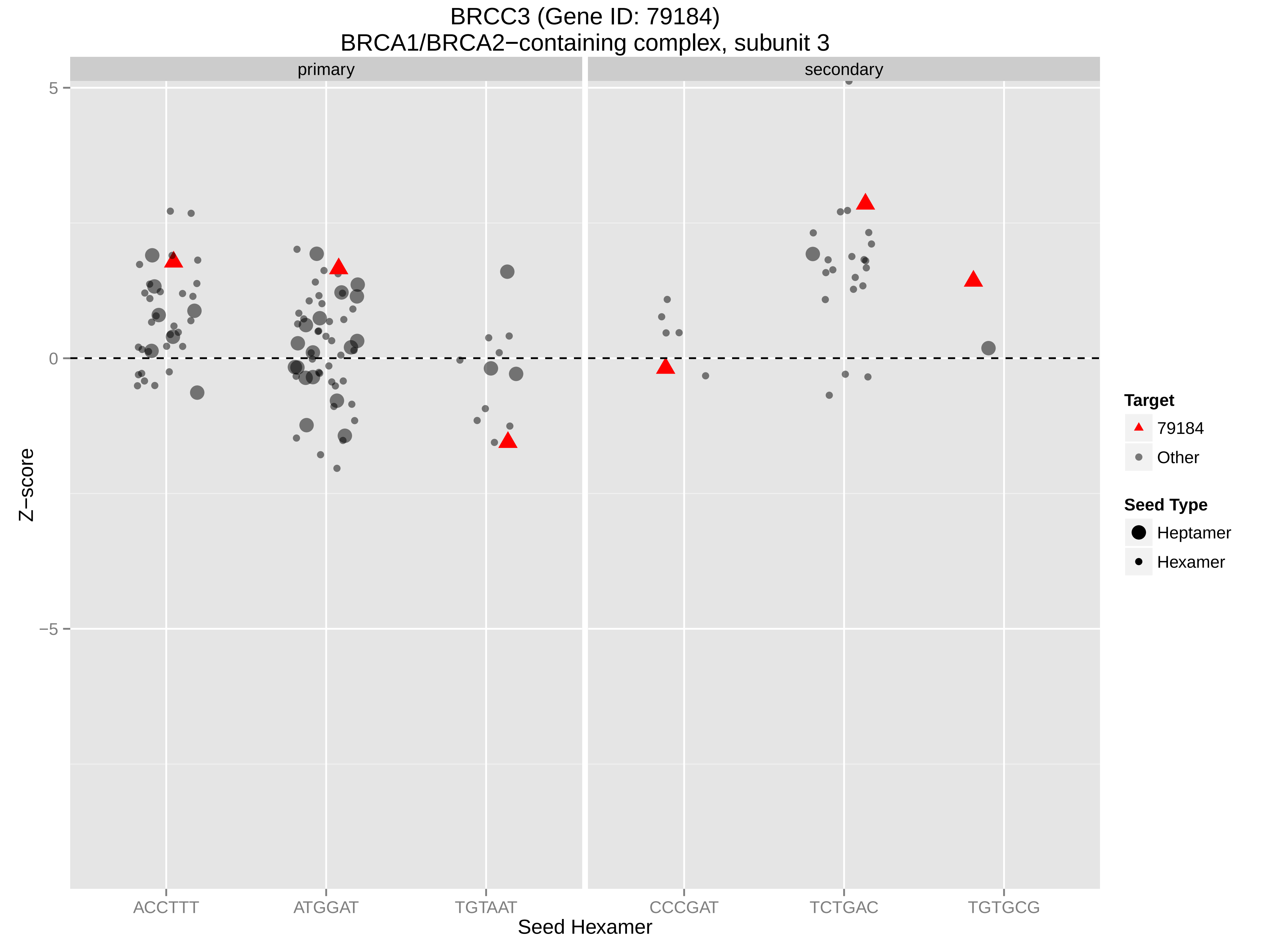

BRCC3 (Gene ID: 79184)
BRCA1/BRCA2−containing complex, subunit 3
primary
secondary
5
0
−5
Z−score
ACCTTT
ATGGAT
TGTAAT
CCCGAT
TCTGAC
TGTGCG
Seed Hexamer
Target
79184
Other
Seed Type
Heptamer
Hexamer

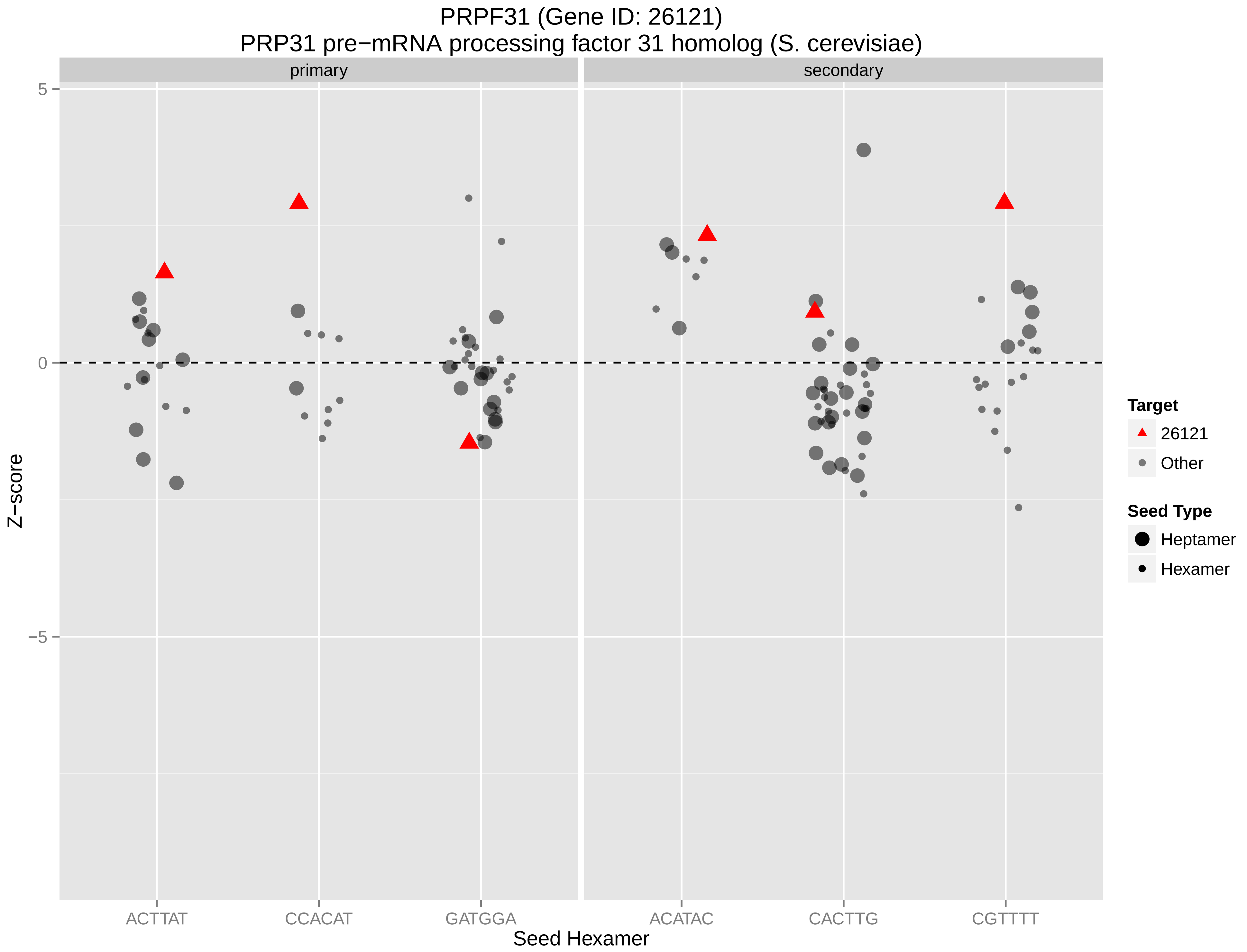

PRPF31 (Gene ID: 26121)
PRP31 pre−mRNA processing factor 31 homolog (S. cerevisiae)
primary
secondary
5
0
−5
Z−score
ACTTAT
CCACAT
GATGGA
ACATAC
CACTTG
CGTTTT
Seed Hexamer
Target
26121
Other
Seed Type
Heptamer
Hexamer

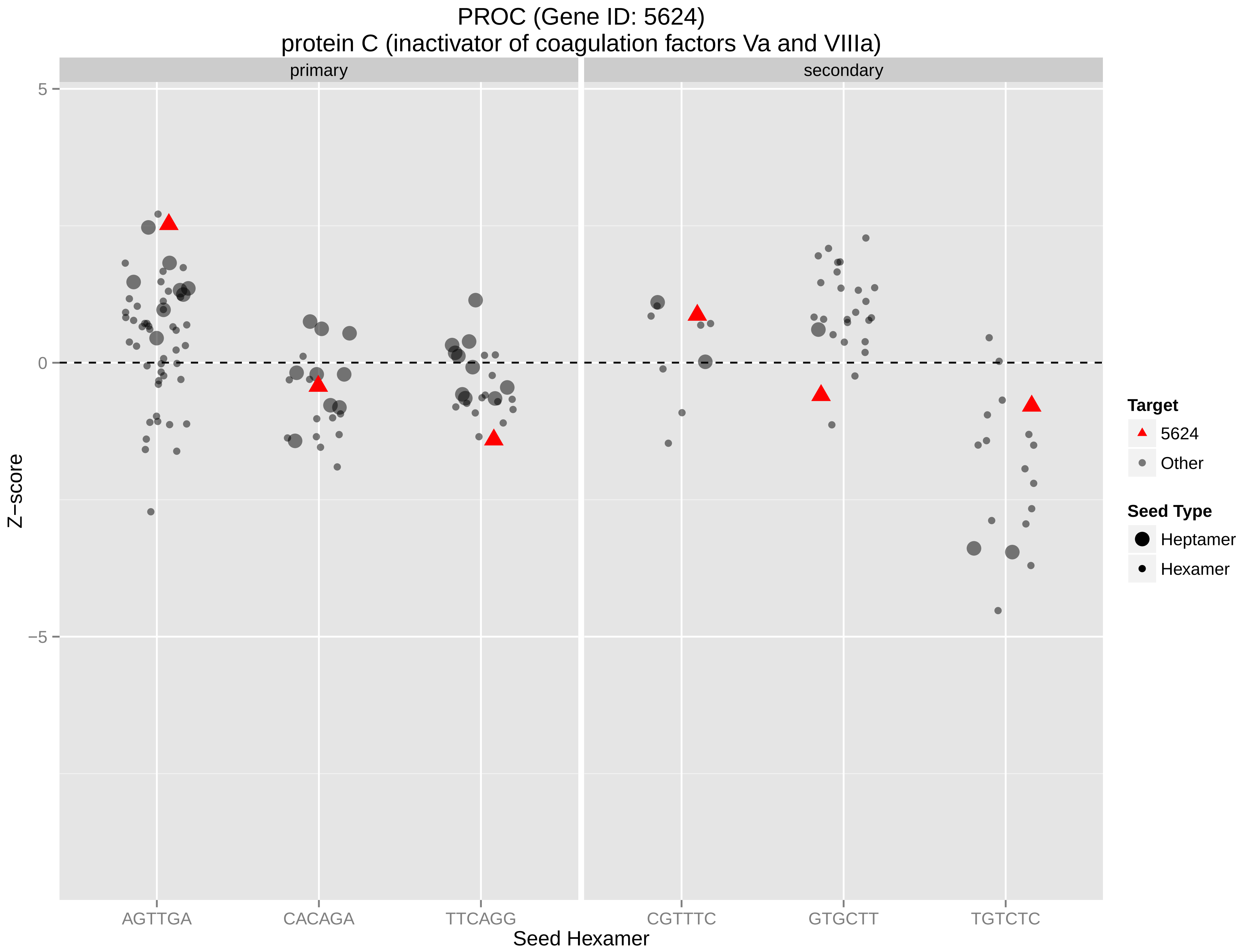

PROC (Gene ID: 5624)
protein C (inactivator of coagulation factors Va and VIIIa)
primary
secondary
5
0
−5
Z−score
AGTTGA
CACAGA
TTCAGG
CGTTTC
GTGCTT
TGTCTC
Seed Hexamer
Target
5624
Other
Seed Type
Heptamer
Hexamer

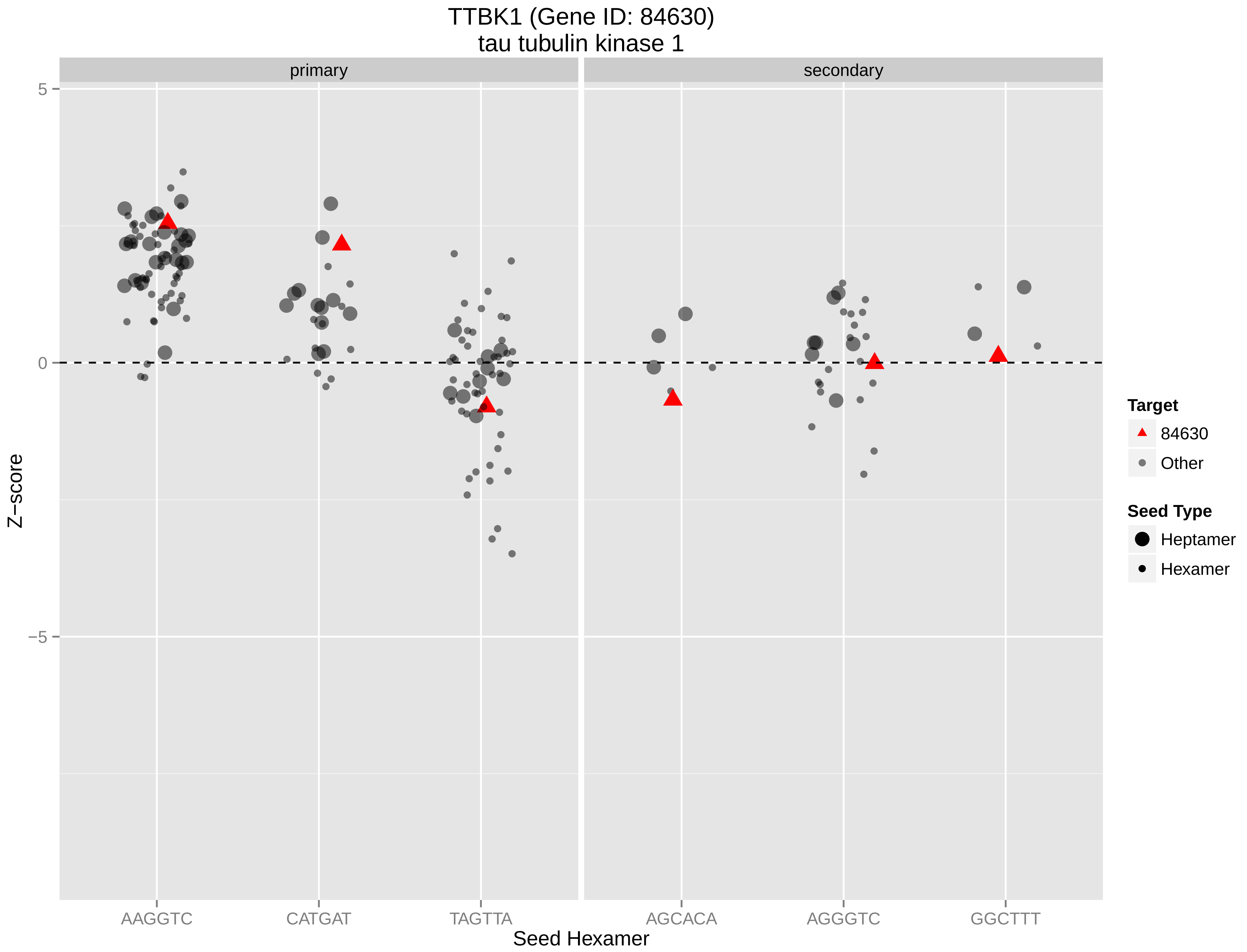

TTBK1 (Gene ID: 84630)
tau tubulin kinase 1
primary
secondary
5
0
−5
Z−score
AAGGTC
CATGAT
TAGTTA
AGCACA
AGGGTC
GGCTTT
Seed Hexamer
Target
84630
Other
Seed Type
Heptamer
Hexamer

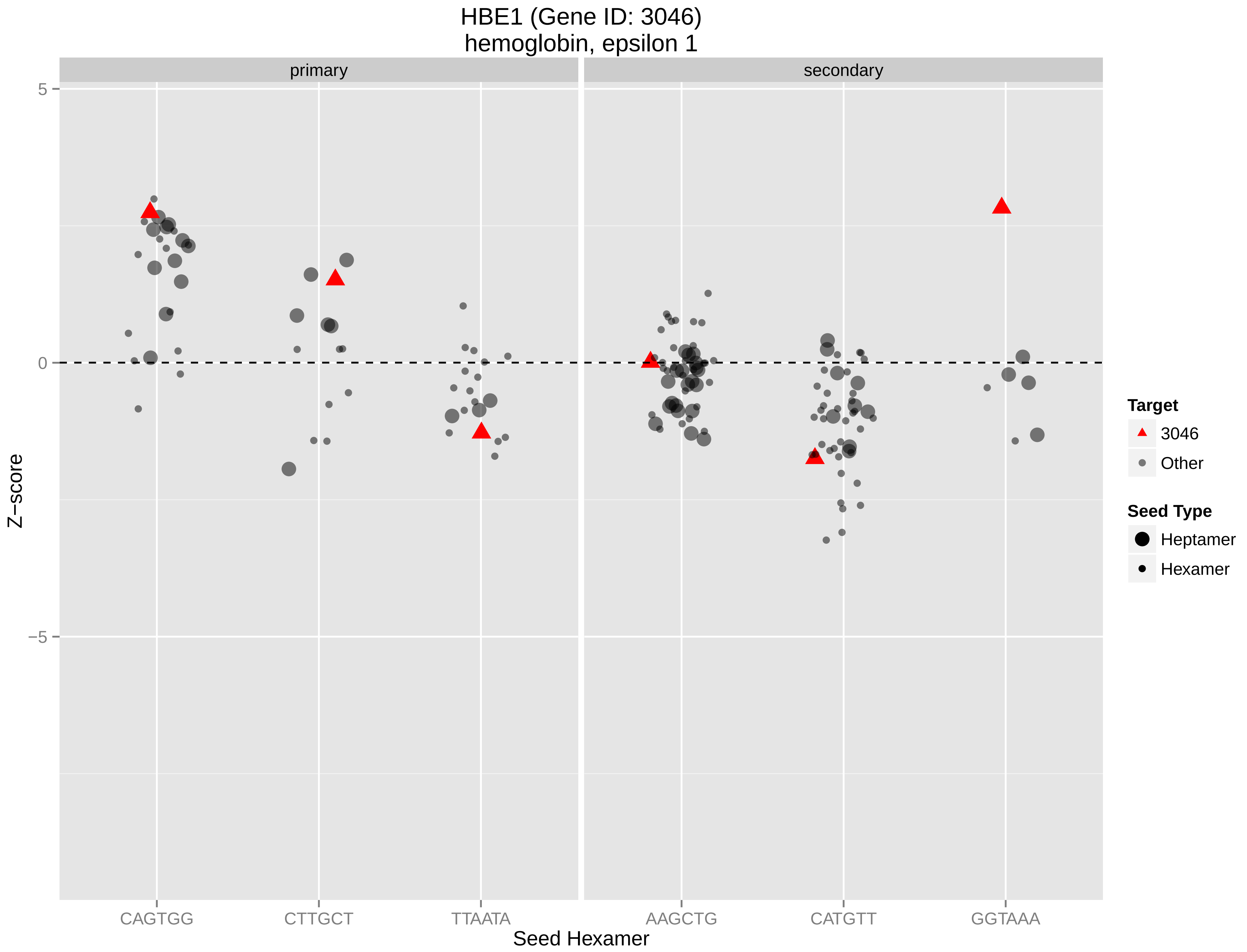

HBE1 (Gene ID: 3046)
hemoglobin, epsilon 1
primary
secondary
5
0
−5
Z−score
CAGTGG
CTTGCT
TTAATA
AAGCTG
CATGTT
GGTAAA
Seed Hexamer
Target
3046
Other
Seed Type
Heptamer
Hexamer

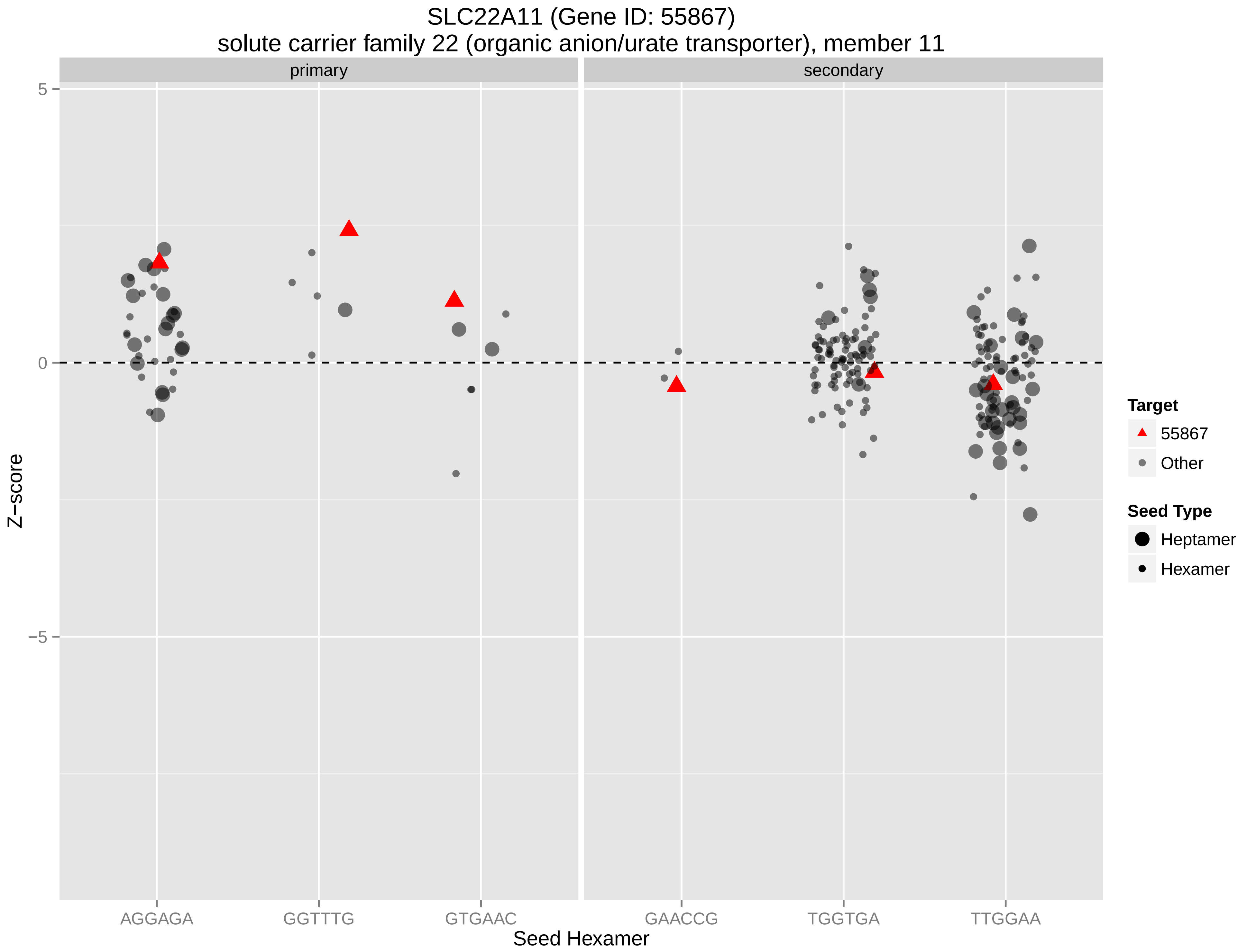
SLC22A11 (Gene ID: 55867)
solute carrier family 22 (organic anion/urate transporter), member 11
primary
secondary
5
0
−5
Z−score
AGGAGA
GGTTTG
GTGAAC
GAACCG
TGGTGA
TTGGAA
Seed Hexamer
Target
55867
Other
Seed Type
Heptamer
Hexamer

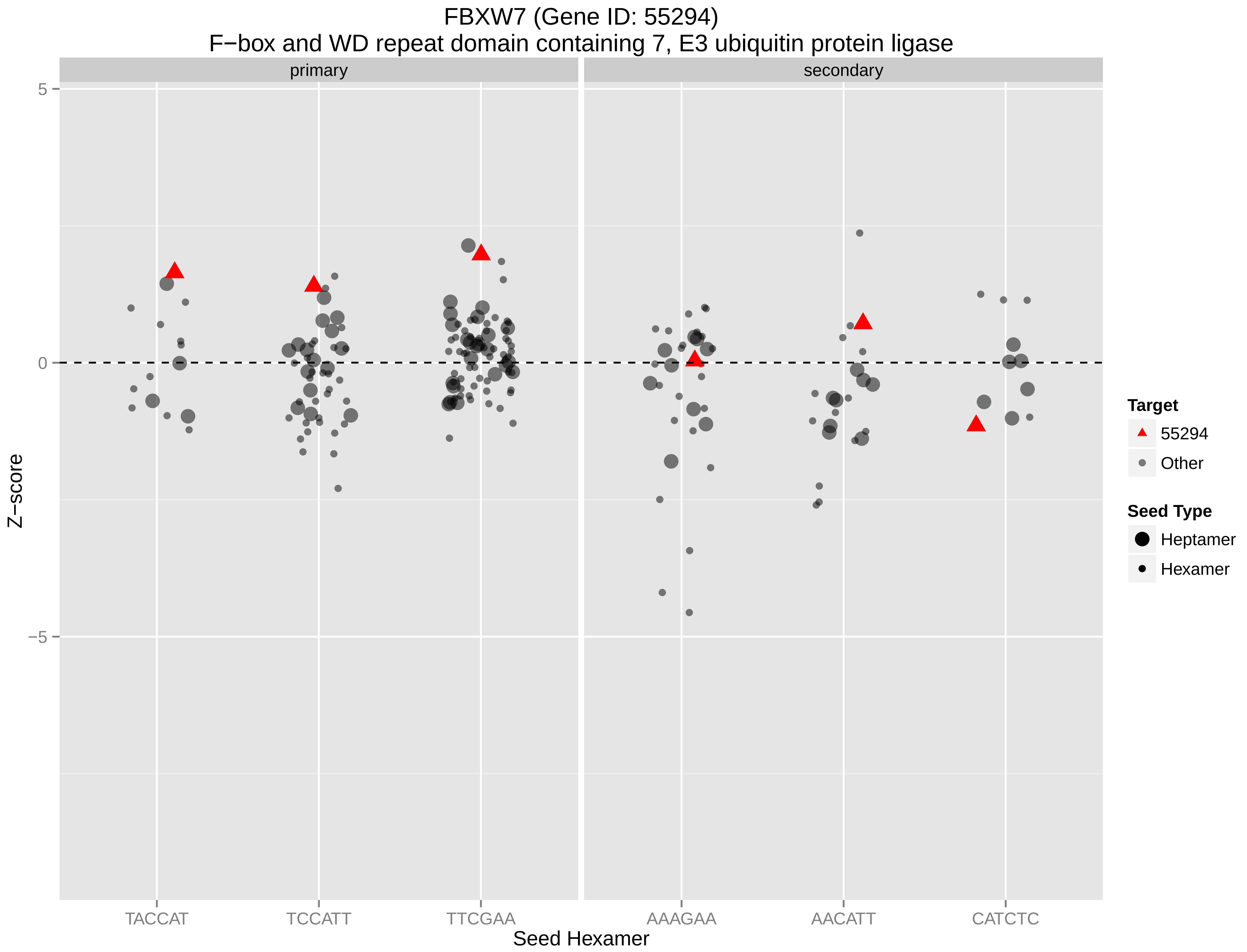
FBXW7 (Gene ID: 55294)
F−box and WD repeat domain containing 7, E3 ubiquitin protein ligase
primary
secondary
5
0
−5
Z−score
TACCAT
TCCATT
TTCGAA
AAAGAA
AACATT
CATCTC
Seed Hexamer
Target
55294
Other
Seed Type
Heptamer
Hexamer

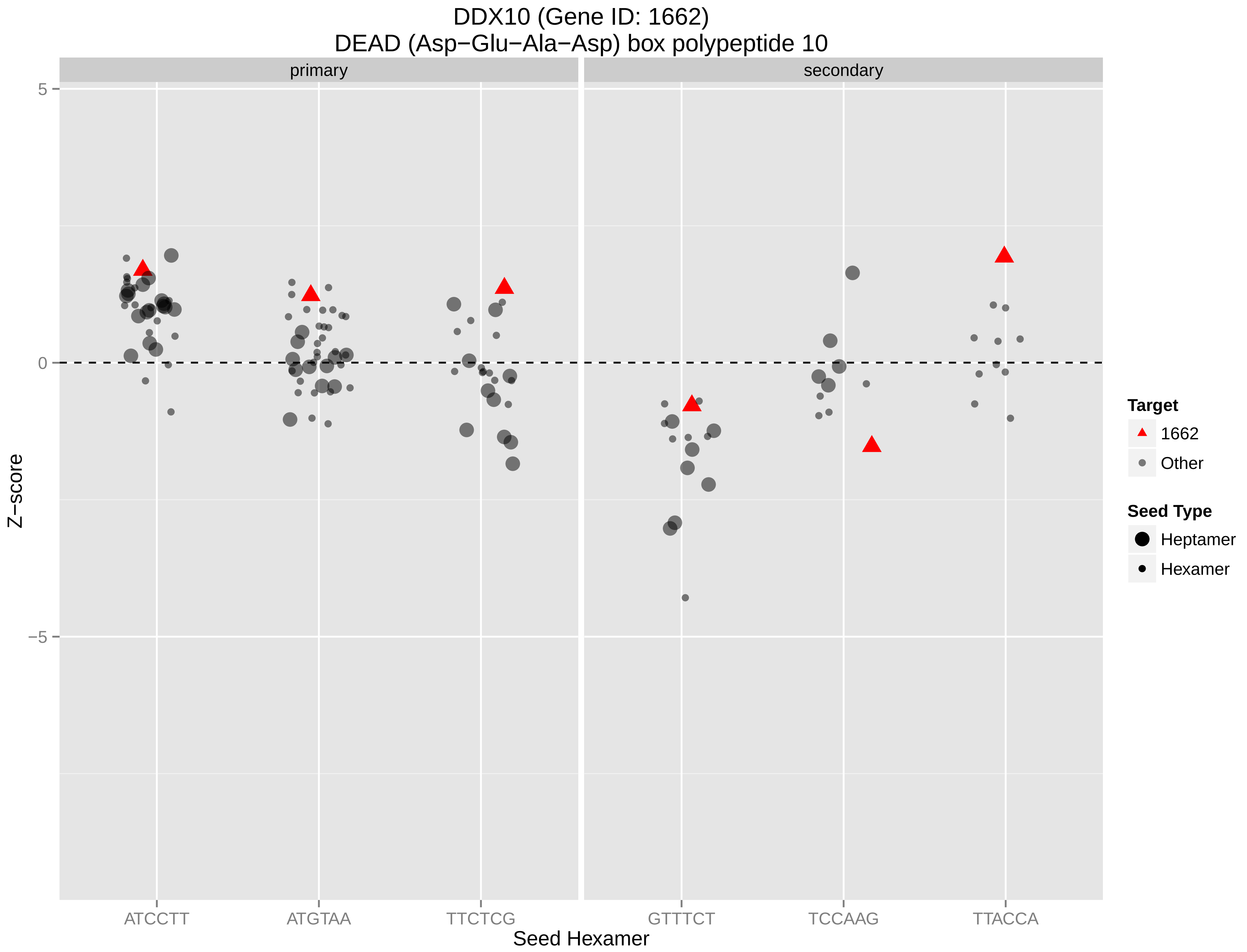

DDX10 (Gene ID: 1662)
DEAD (Asp−Glu−Ala−Asp) box polypeptide 10
primary
secondary
5
0
−5
Z−score
ATCCTT
ATGTAA
TTCTCG
GTTTCT
TCCAAG
TTACCA
Seed Hexamer
Target
1662
Other
Seed Type
Heptamer
Hexamer

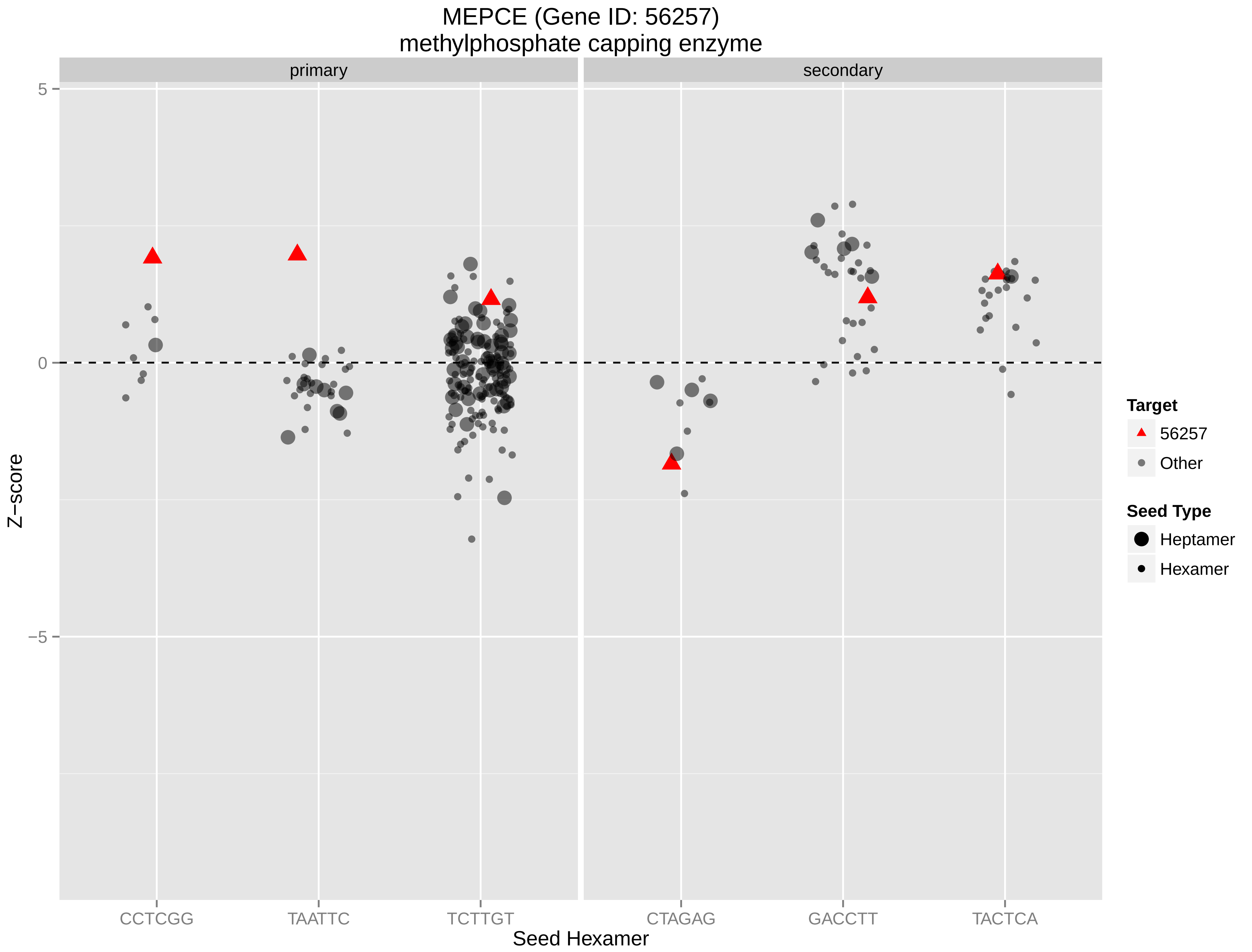

MEPCE (Gene ID: 56257)
methylphosphate capping enzyme
primary
secondary
5
0
−5
Z−score
CCTCGG
TAATTC
TCTTGT
CTAGAG
GACCTT
TACTCA
Seed Hexamer
Target
56257
Other
Seed Type
Heptamer
Hexamer

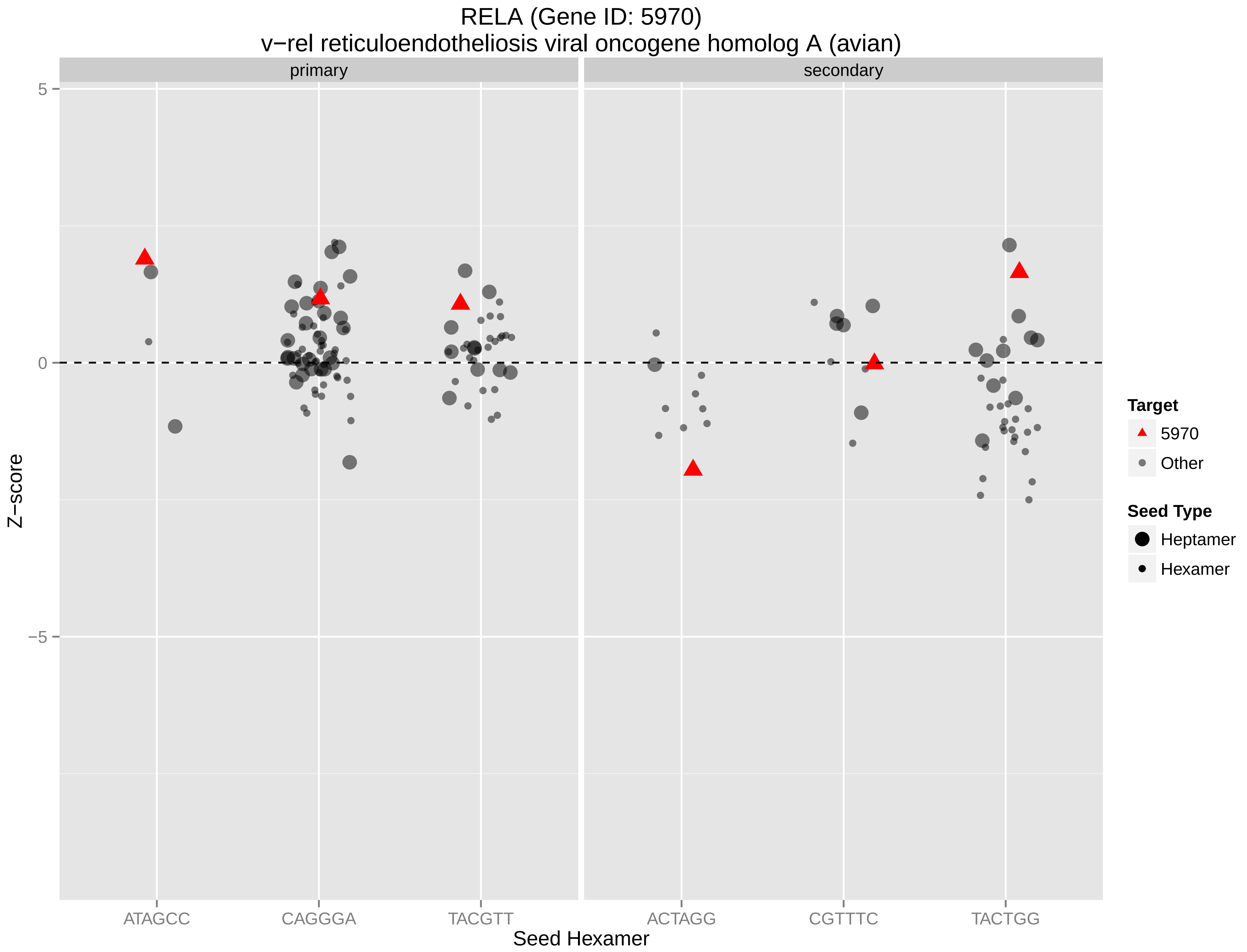

RELA (Gene ID: 5970)
v−rel reticuloendotheliosis viral oncogene homolog A (avian)
primary
secondary
5
0
−5
Z−score
ATAGCC
CAGGGA
TACGTT
ACTAGG
CGTTTC
TACTGG
Seed Hexamer
Target
5970
Other
Seed Type
Heptamer
Hexamer

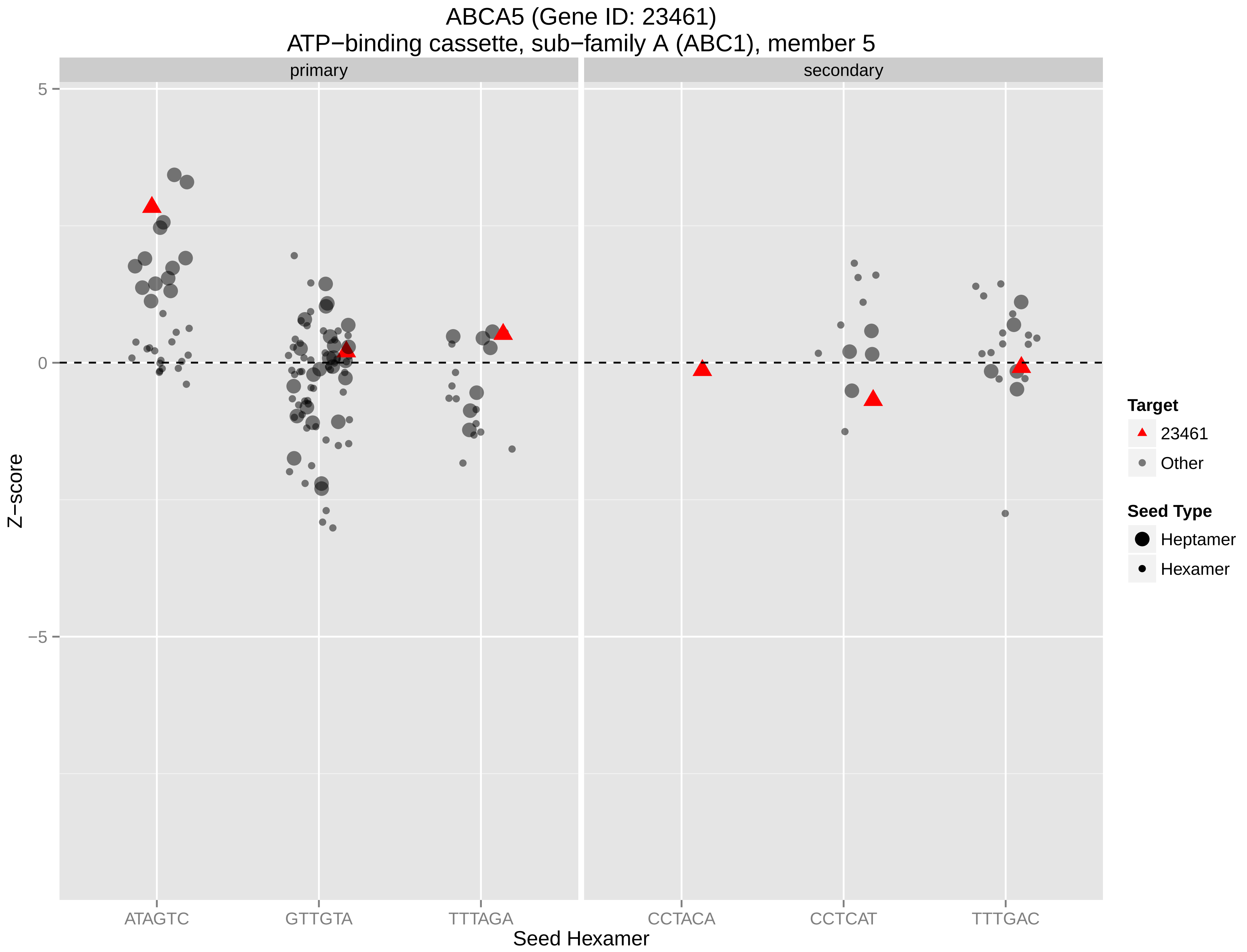
ABCA5 (Gene ID: 23461)
ATP−binding cassette, sub−family A (ABC1), member 5
primary
secondary
5
0
−5
Z−score
ATAGTC
GTTGTA
TTTAGA
CCTACA
CCTCAT
TTTGAC
Seed Hexamer
Target
23461
Other
Seed Type
Heptamer
Hexamer

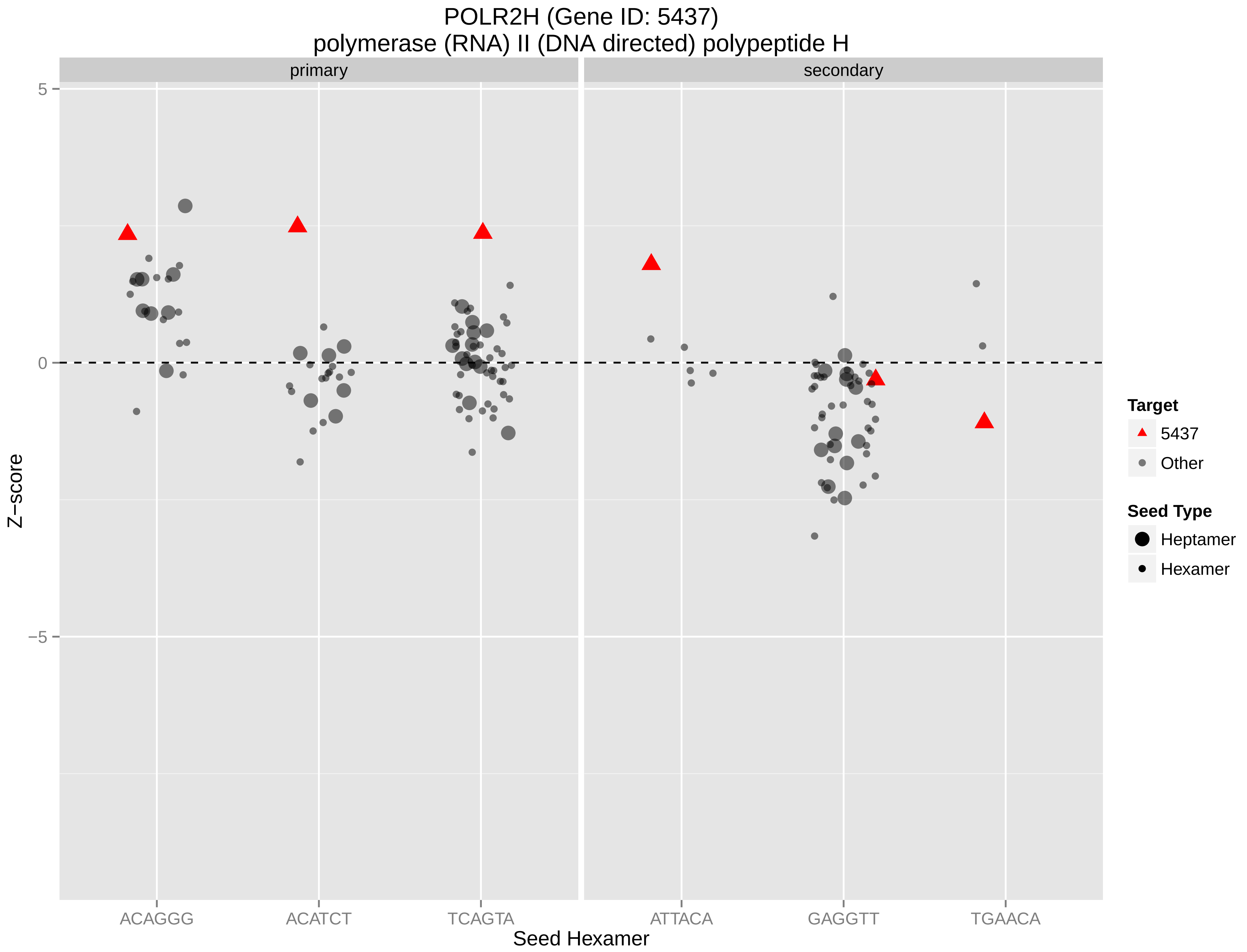

POLR2H (Gene ID: 5437)
polymerase (RNA) II (DNA directed) polypeptide H
primary
secondary
5
0
−5
Z−score
ACAGGG
ACATCT
TCAGTA
ATTACA
GAGGTT
TGAACA
Seed Hexamer
Target
5437
Other
Seed Type
Heptamer
Hexamer

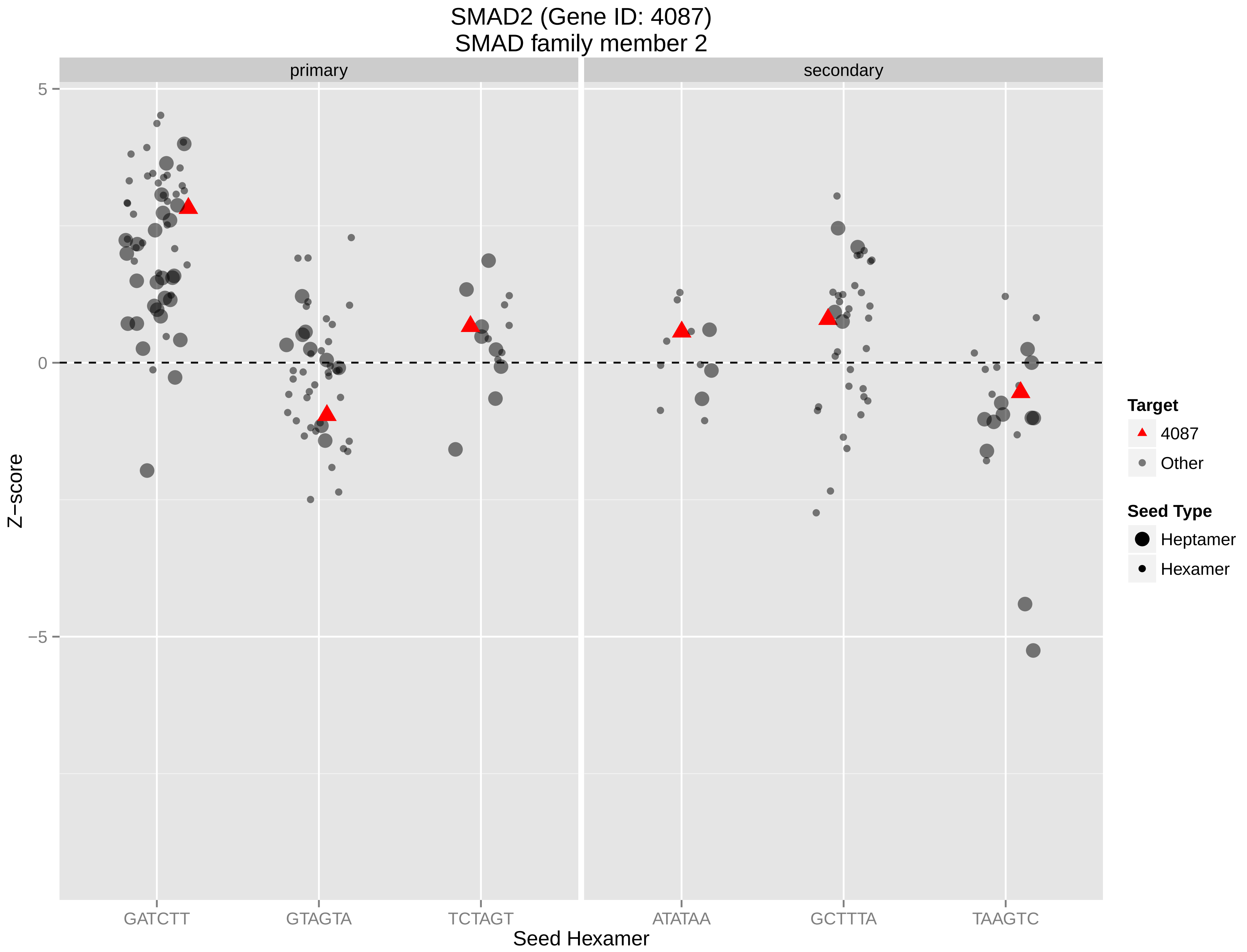
SMAD2 (Gene ID: 4087)
SMAD family member 2
primary
secondary
5
0
−5
Z−score
GATCTT
GTAGTA
TCTAGT
ATATAA
GCTTTA
TAAGTC
Seed Hexamer
Target
4087
Other
Seed Type
Heptamer
Hexamer

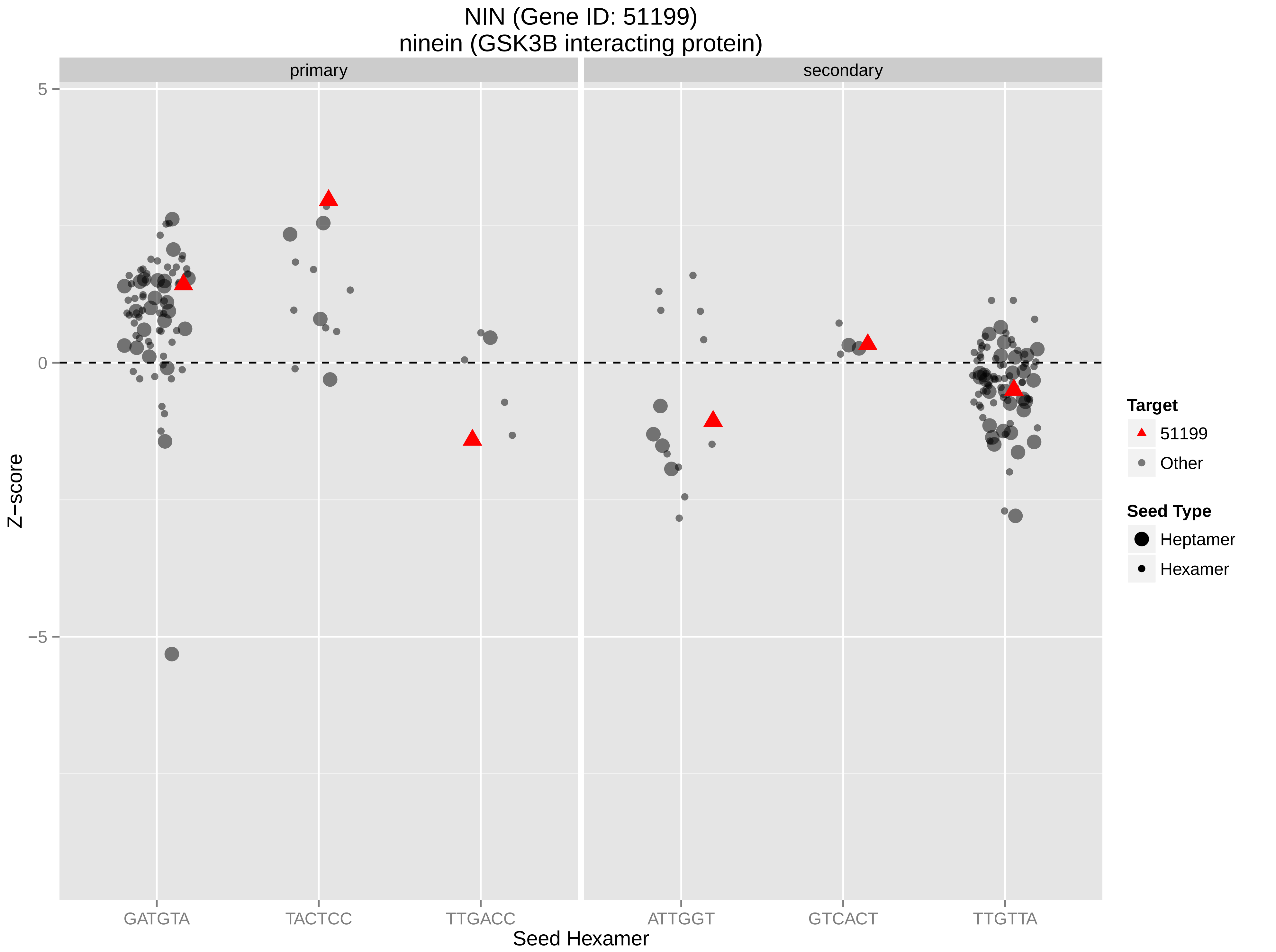

NIN (Gene ID: 51199)
ninein (GSK3B interacting protein)
primary
secondary
5
0
−5
Z−score
GATGTA
TACTCC
TTGACC
ATTGGT
GTCACT
TTGTTA
Seed Hexamer
Target
51199
Other
Seed Type
Heptamer
Hexamer

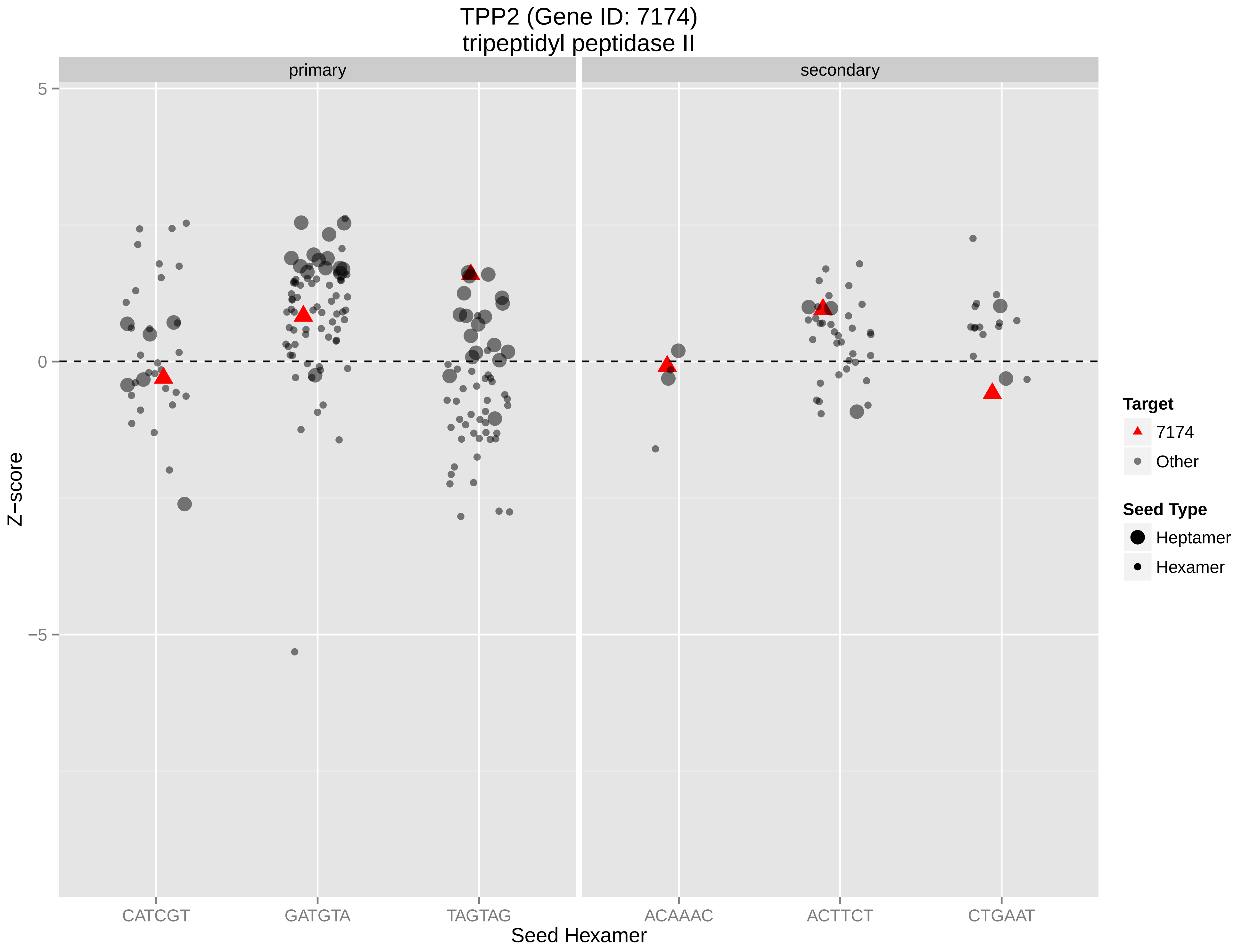

TPP2 (Gene ID: 7174)
tripeptidyl peptidase II
primary
secondary
5
0
−5
Z−score
CATCGT
GATGTA
TAGTAG
ACAAAC
ACTTCT
CTGAAT
Seed Hexamer
Target
7174
Other
Seed Type
Heptamer
Hexamer

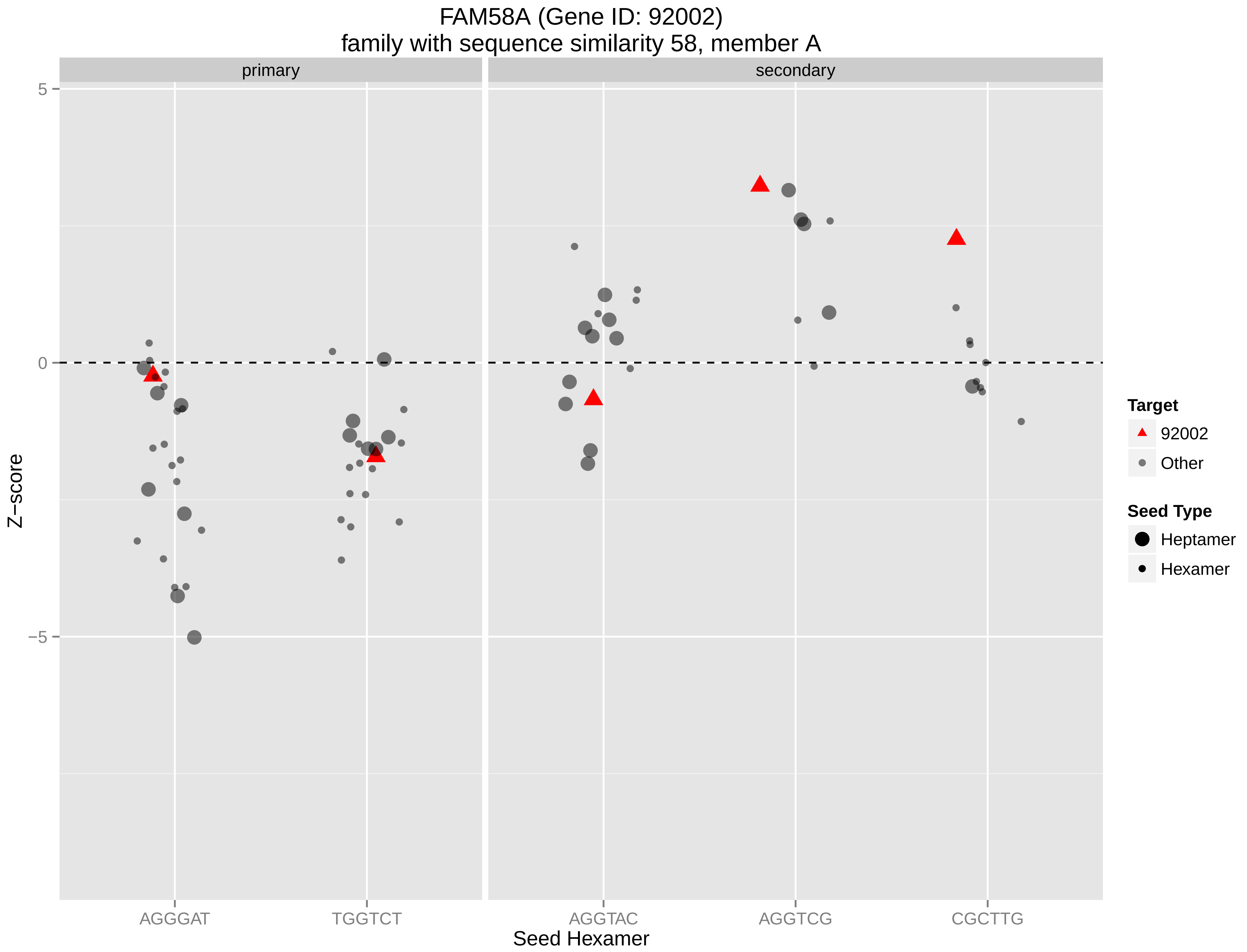
FAM58A (Gene ID: 92002)
family with sequence similarity 58, member A
primary
secondary
5
0
−5
Z−score
AGGGAT
TGGTCT
AGGTAC
AGGTCG
CGCTTG
Seed Hexamer
Target
92002
Other
Seed Type
Heptamer
Hexamer

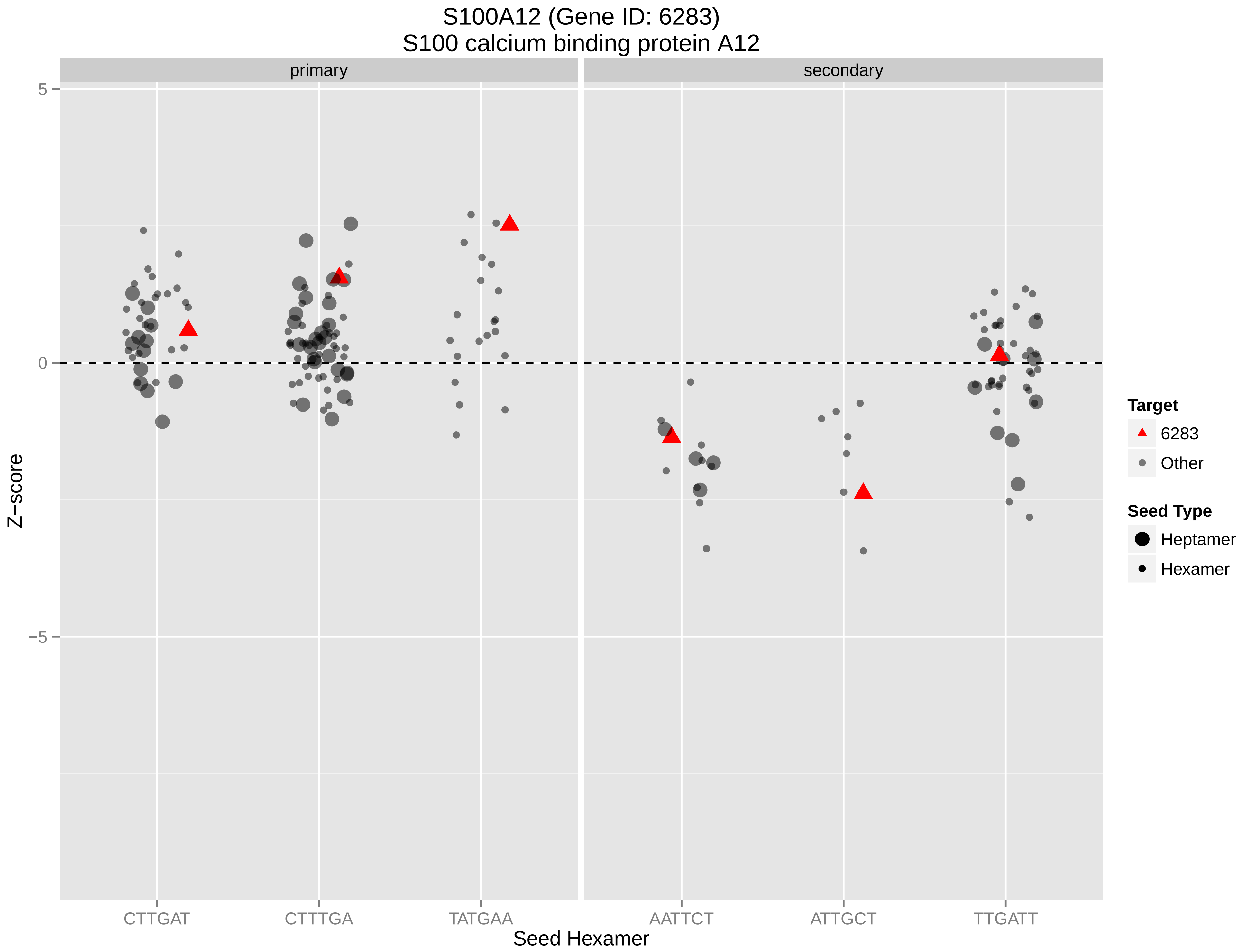
S100A12 (Gene ID: 6283)
S100 calcium binding protein A12
primary
secondary
5
0
−5
Z−score
CTTGAT
CTTTGA
TATGAA
AATTCT
ATTGCT
TTGATT
Seed Hexamer
Target
6283
Other
Seed Type
Heptamer
Hexamer

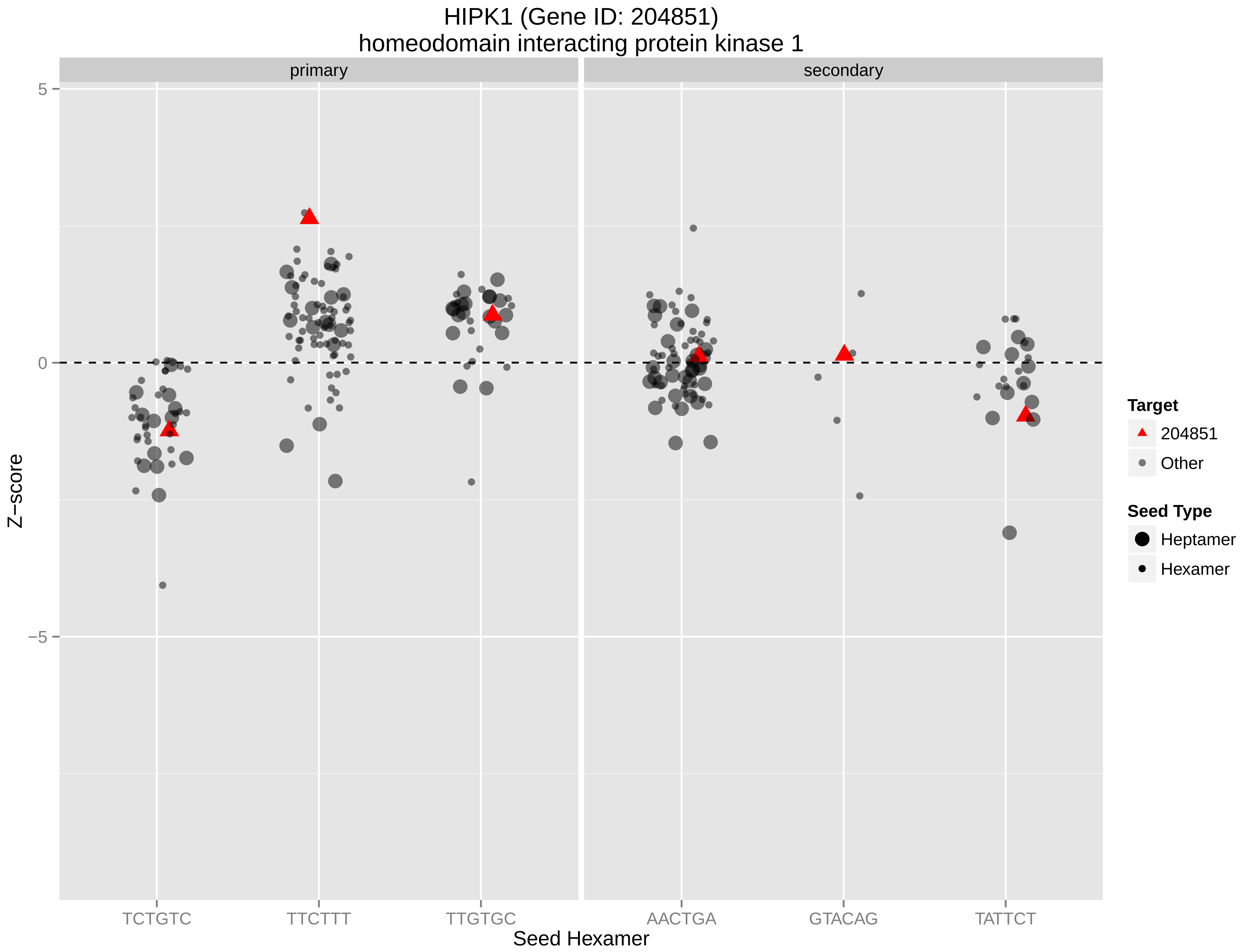
HIPK1 (Gene ID: 204851)
homeodomain interacting protein kinase 1
primary
secondary
5
0
−5
Z−score
TCTGTC
TTCTTT
TTGTGC
AACTGA
GTACAG
TATTCT
Seed Hexamer
Target
204851
Other
Seed Type
Heptamer
Hexamer

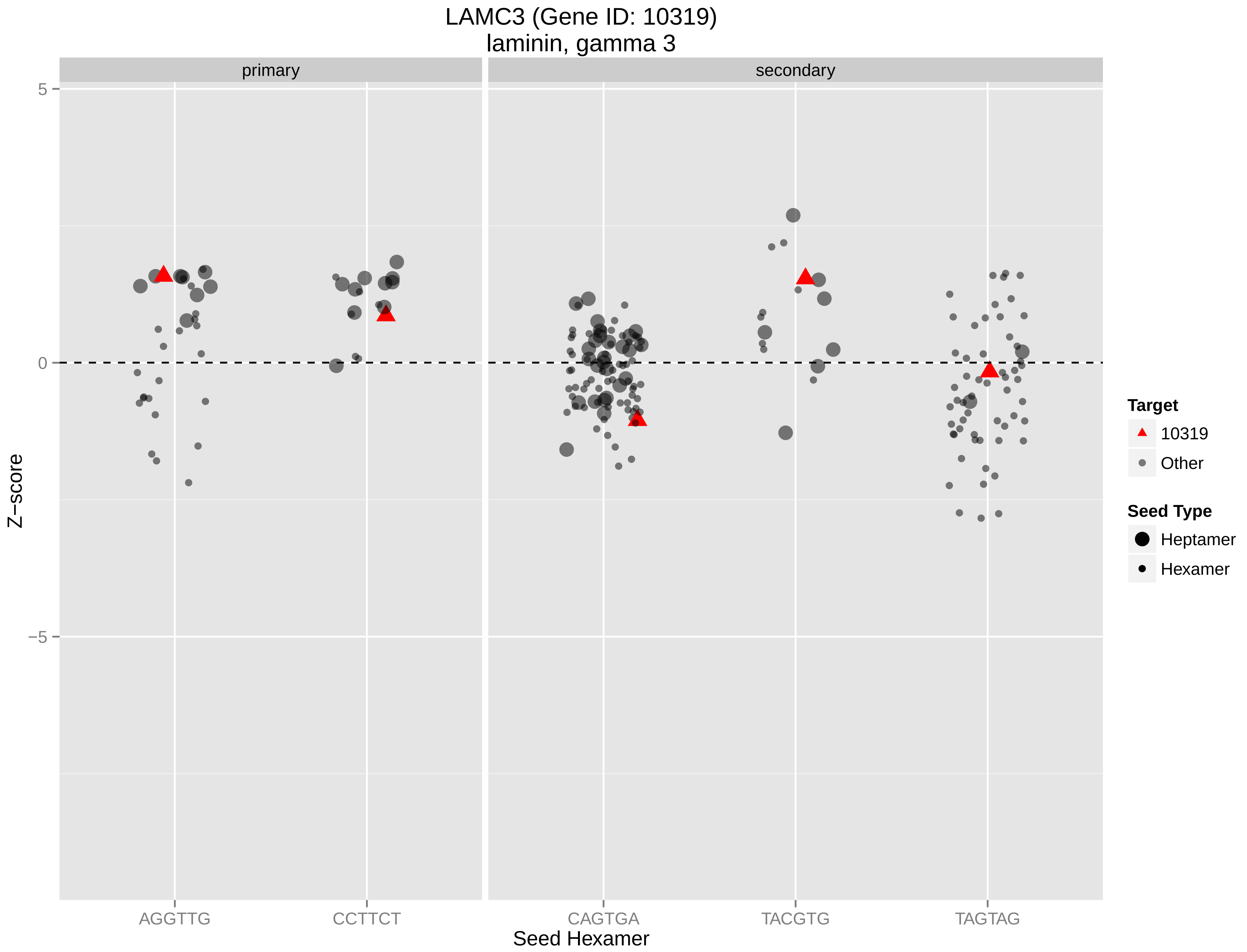
LAMC3 (Gene ID: 10319)
laminin, gamma 3
primary
secondary
5
0
−5
Z−score
AGGTTG
CCTTCT
CAGTGA
TACGTG
TAGTAG
Seed Hexamer
Target
10319
Other
Seed Type
Heptamer
Hexamer

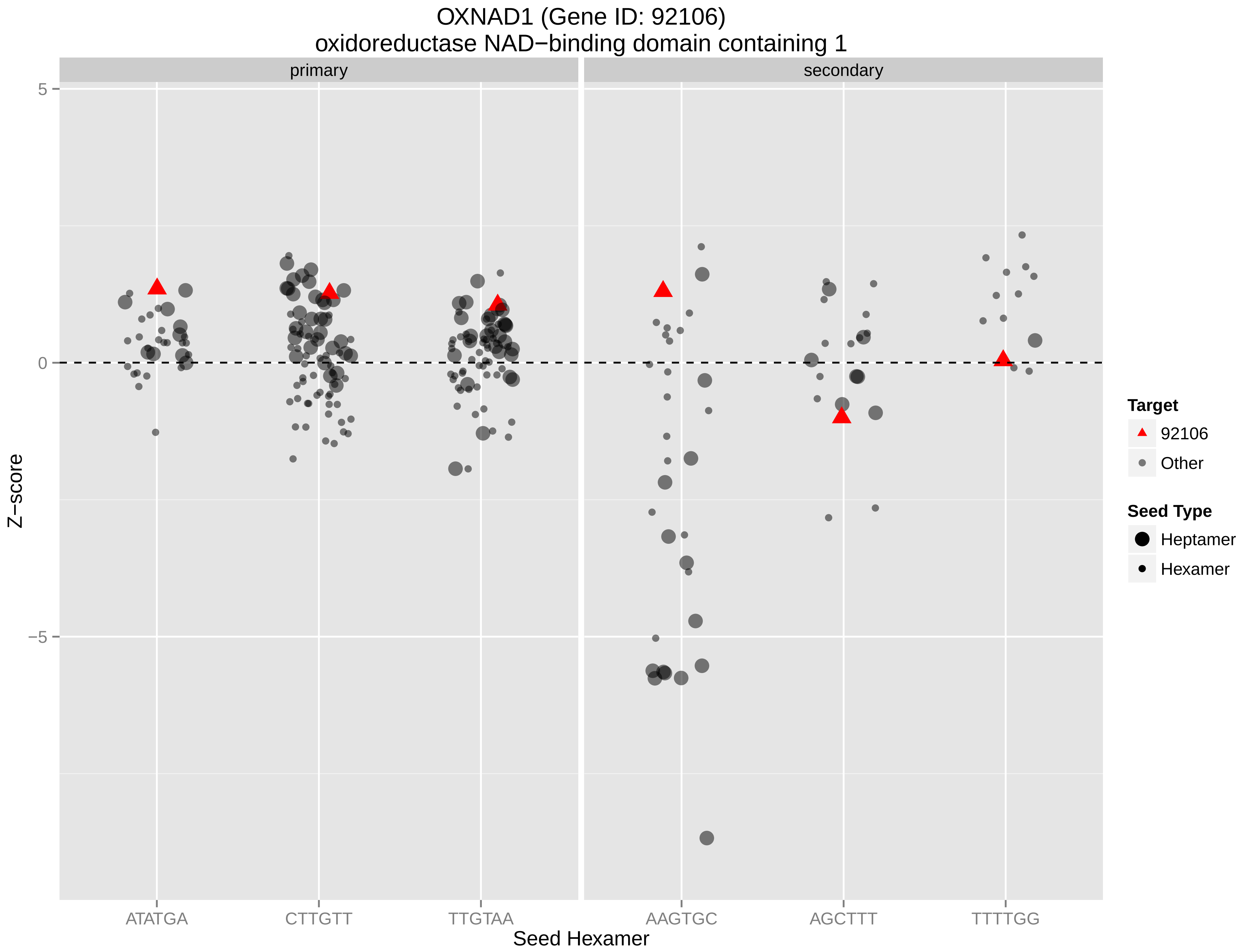
OXNAD1 (Gene ID: 92106)
oxidoreductase NAD−binding domain containing 1
primary
secondary
5
0
−5
Z−score
ATATGA
CTTGTT
TTGTAA
AAGTGC
AGCTTT
TTTTGG
Seed Hexamer
Target
92106
Other
Seed Type
Heptamer
Hexamer

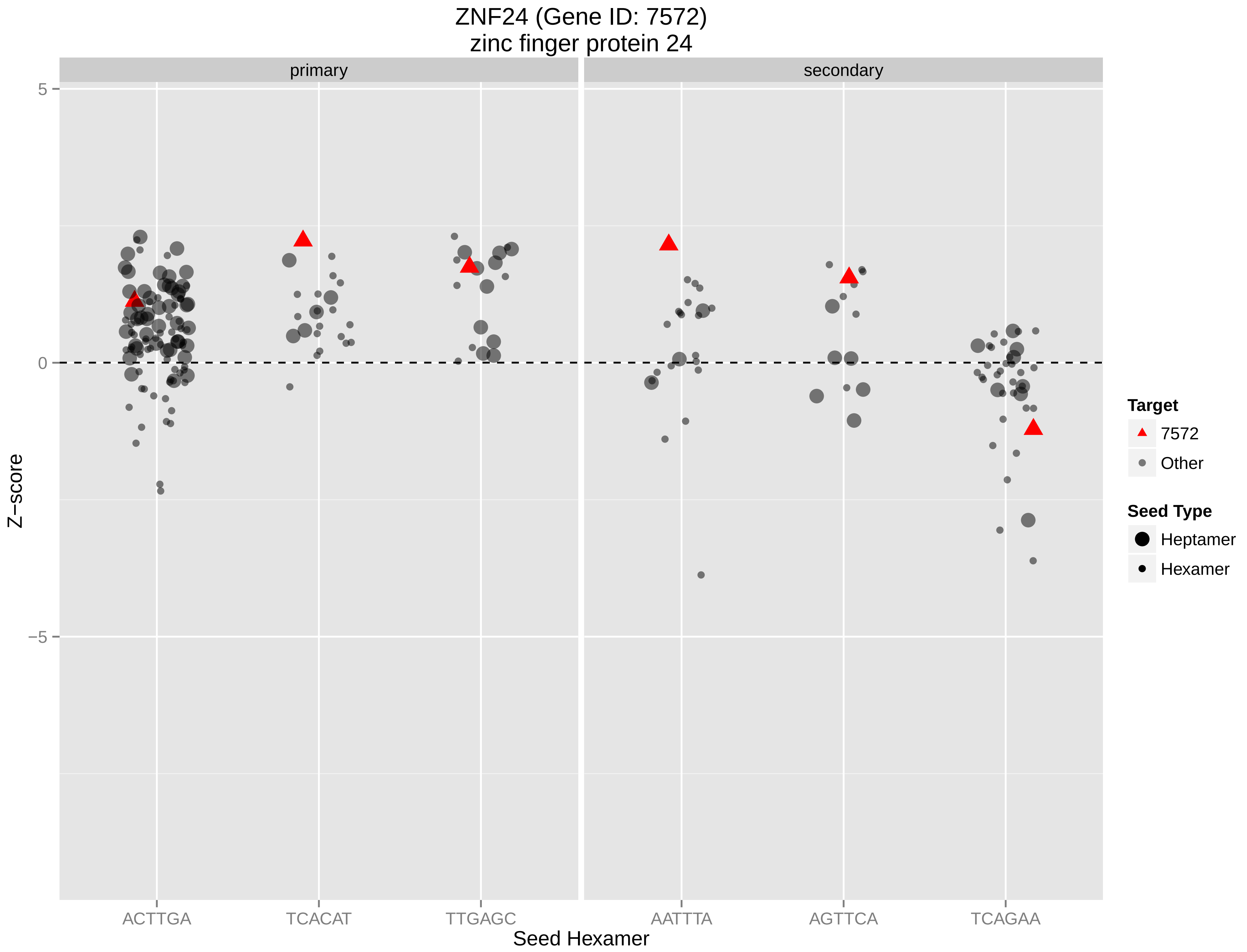

ZNF24 (Gene ID: 7572)
zinc finger protein 24
primary
secondary
5
0
−5
Z−score
ACTTGA
TCACAT
TTGAGC
AATTTA
AGTTCA
TCAGAA
Seed Hexamer
Target
7572
Other
Seed Type
Heptamer
Hexamer

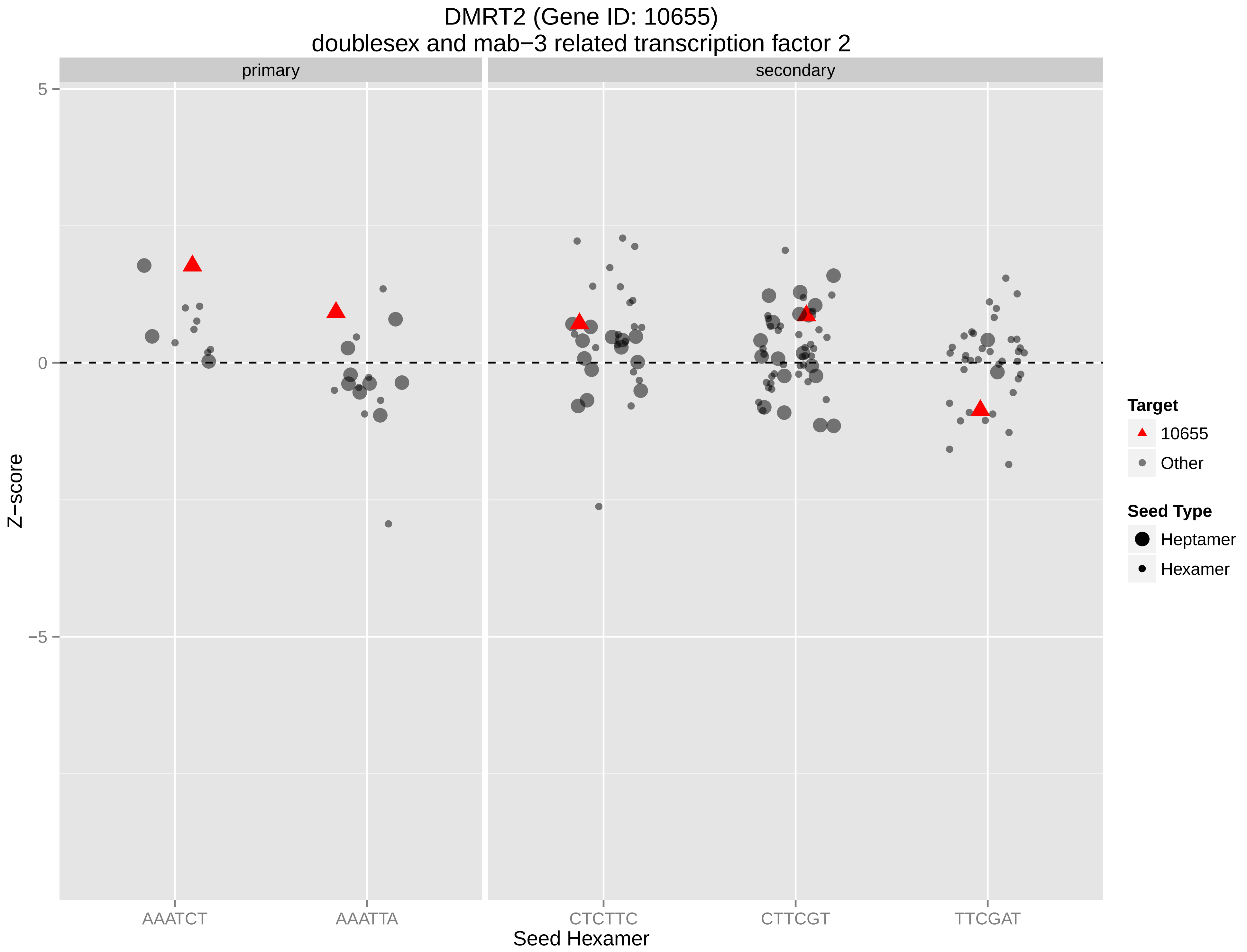
DMRT2 (Gene ID: 10655)
doublesex and mab−3 related transcription factor 2
primary
secondary
5
0
−5
Z−score
AAATCT
AAATTA
CTCTTC
CTTCGT
TTCGAT
Seed Hexamer
Target
10655
Other
Seed Type
Heptamer
Hexamer

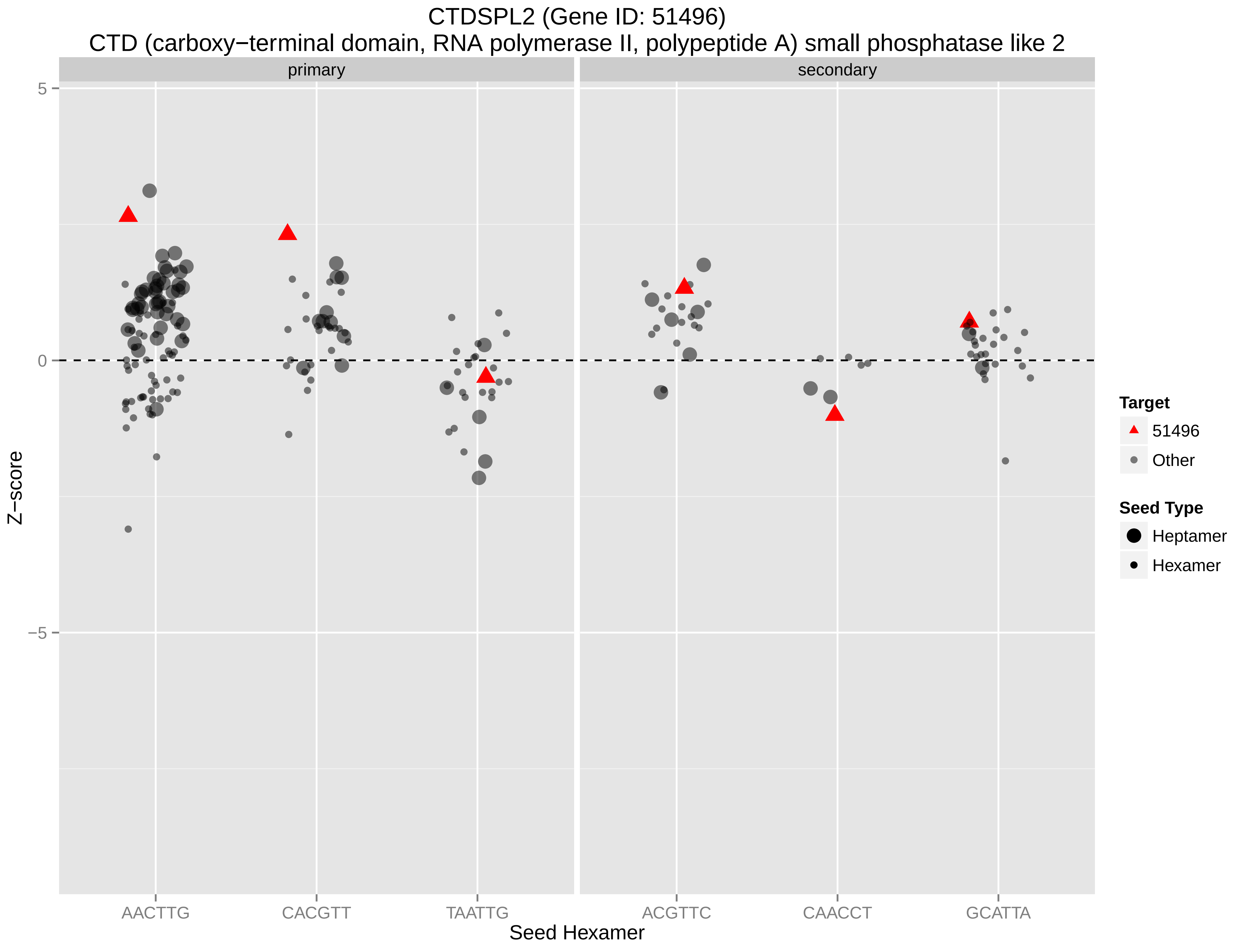
CTDSPL2 (Gene ID: 51496)
CTD (carboxy−terminal domain, RNA polymerase II, polypeptide A) small phosphatase like 2
primary
secondary
5
0
−5
Z−score
AACTTG
CACGTT
TAATTG
ACGTTC
CAACCT
GCATTA
Seed Hexamer
Target
51496
Other
Seed Type
Heptamer
Hexamer

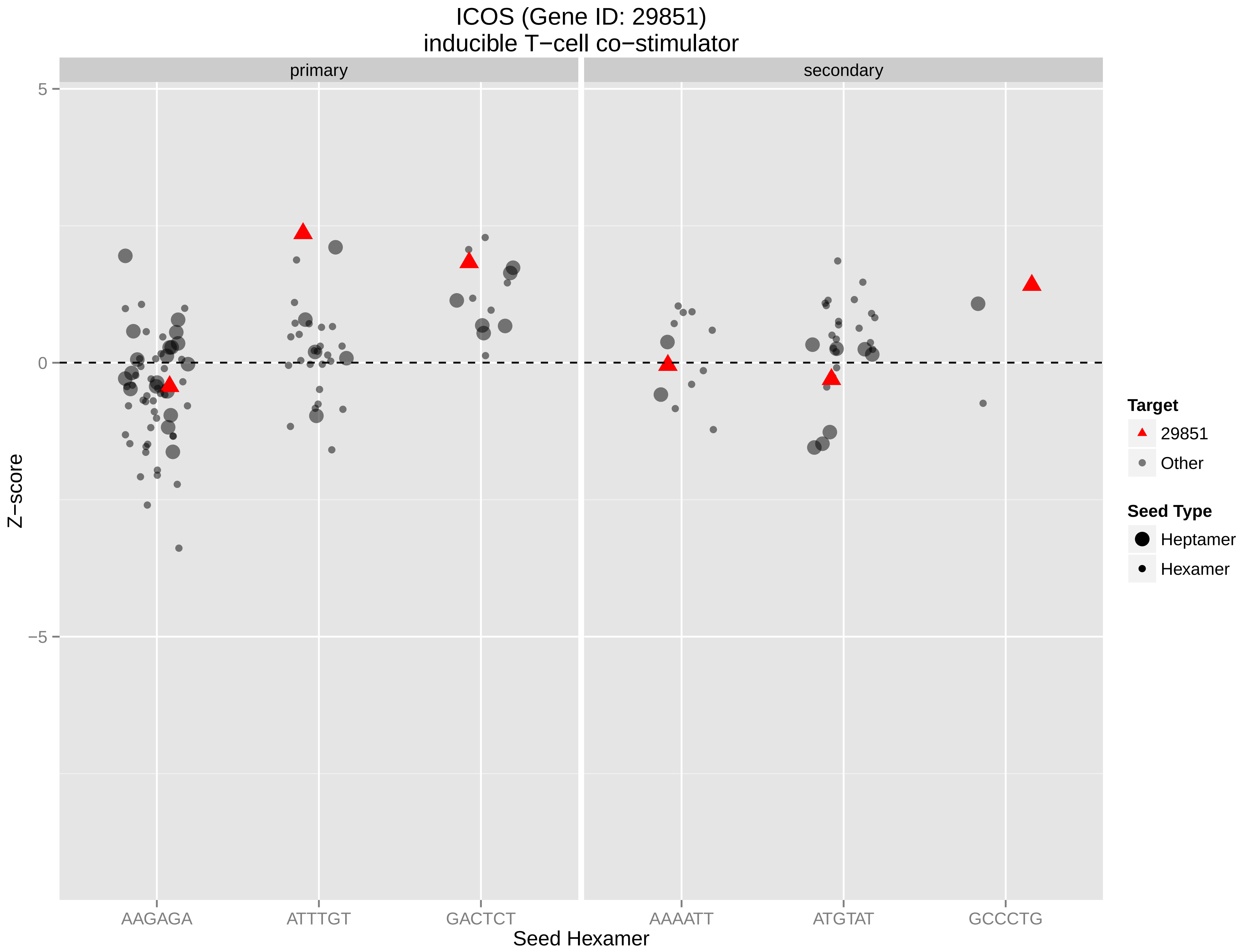
ICOS (Gene ID: 29851)
inducible T−cell co−stimulator
primary
secondary
5
0
−5
Z−score
AAGAGA
ATTTGT
GACTCT
AAAATT
ATGTAT
GCCCTG
Seed Hexamer
Target
29851
Other
Seed Type
Heptamer
Hexamer

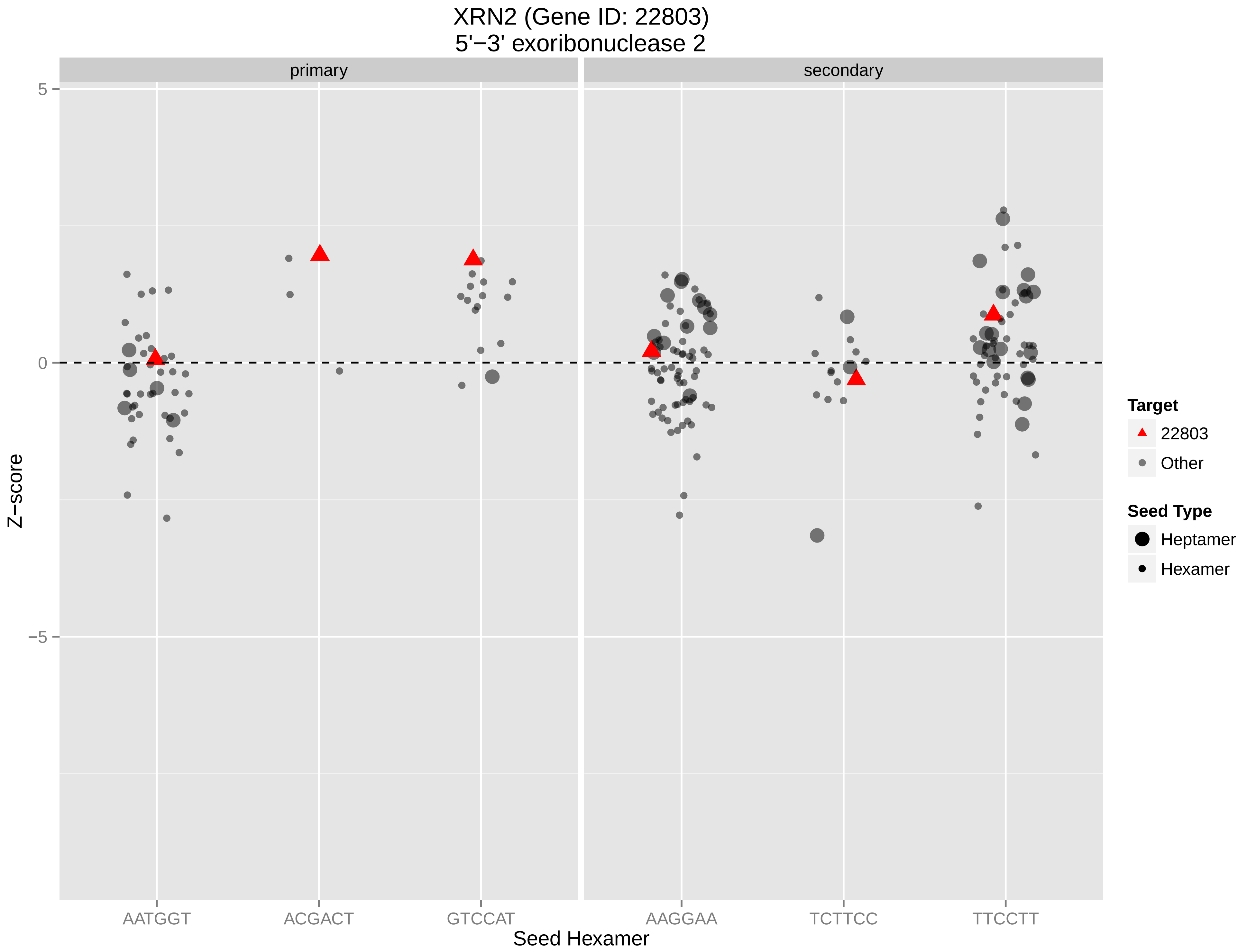
XRN2 (Gene ID: 22803)
5'−3' exoribonuclease 2
primary
secondary
Z−score
5
0
−5
AATGGT
ACGACT
GTCCAT
AAGGAA
TCTTCC
TTCCTT
Seed Hexamer
Target
22803
Other
Seed Type
Heptamer
Hexamer

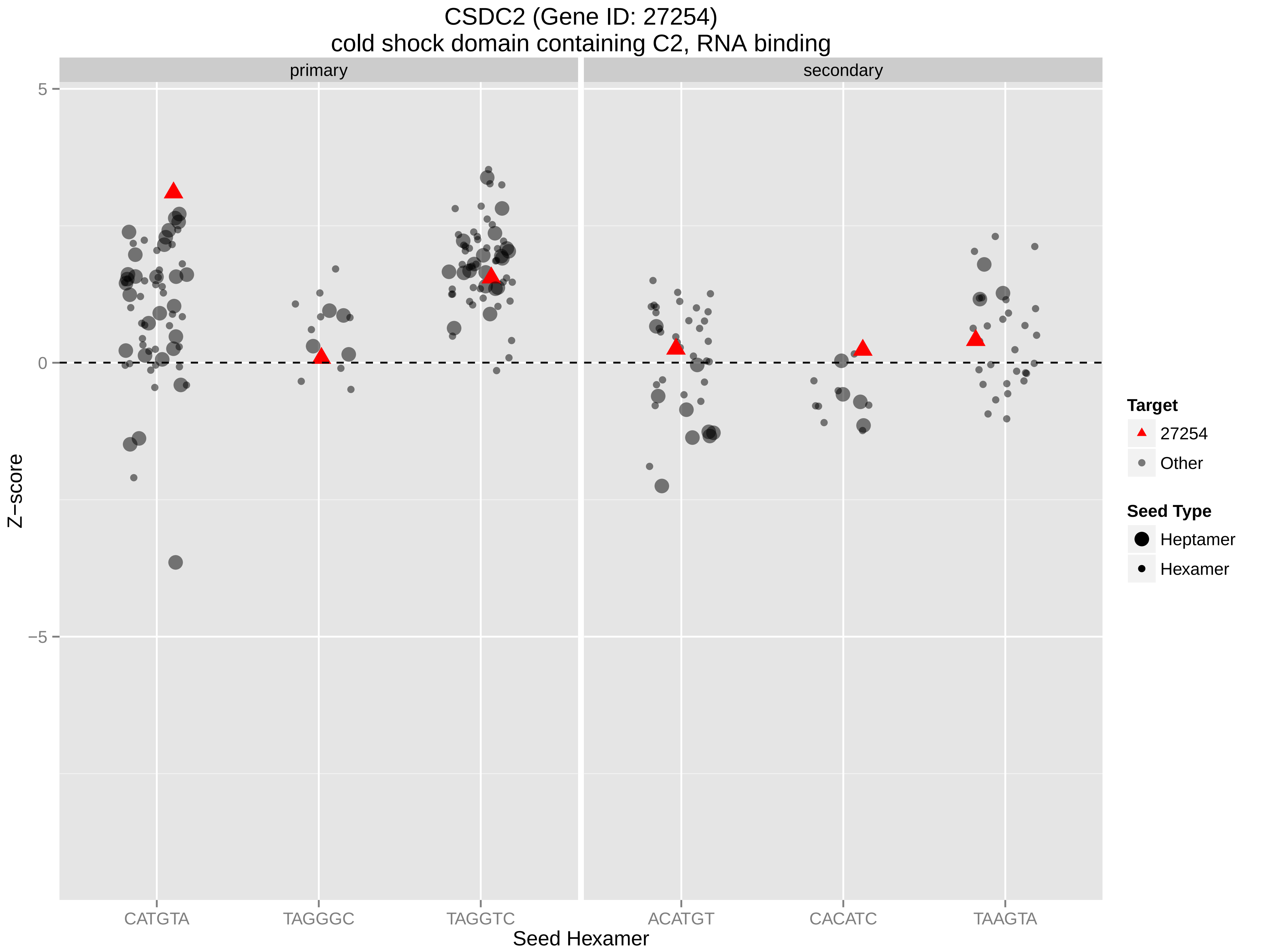
CSDC2 (Gene ID: 27254)
cold shock domain containing C2, RNA binding
primary
secondary
5
0
−5
Z−score
CATGTA
TAGGGC
TAGGTC
ACATGT
CACATC
TAAGTA
Seed Hexamer
Target
27254
Other
Seed Type
Heptamer
Hexamer

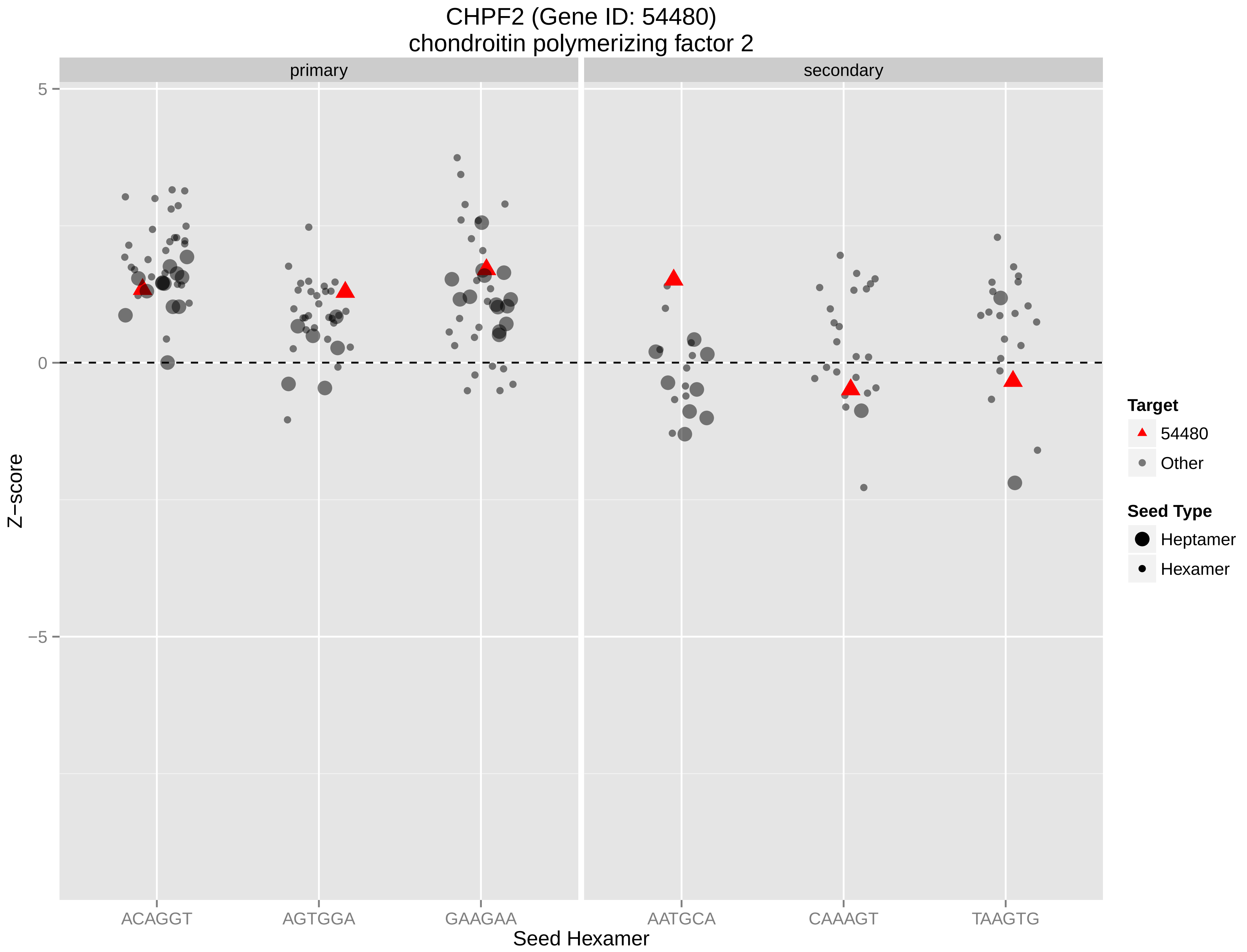
CHPF2 (Gene ID: 54480)
chondroitin polymerizing factor 2
primary
secondary
5
0
−5
Z−score
ACAGGT
AGTGGA
GAAGAA
AATGCA
CAAAGT
TAAGTG
Seed Hexamer
Target
54480
Other
Seed Type
Heptamer
Hexamer

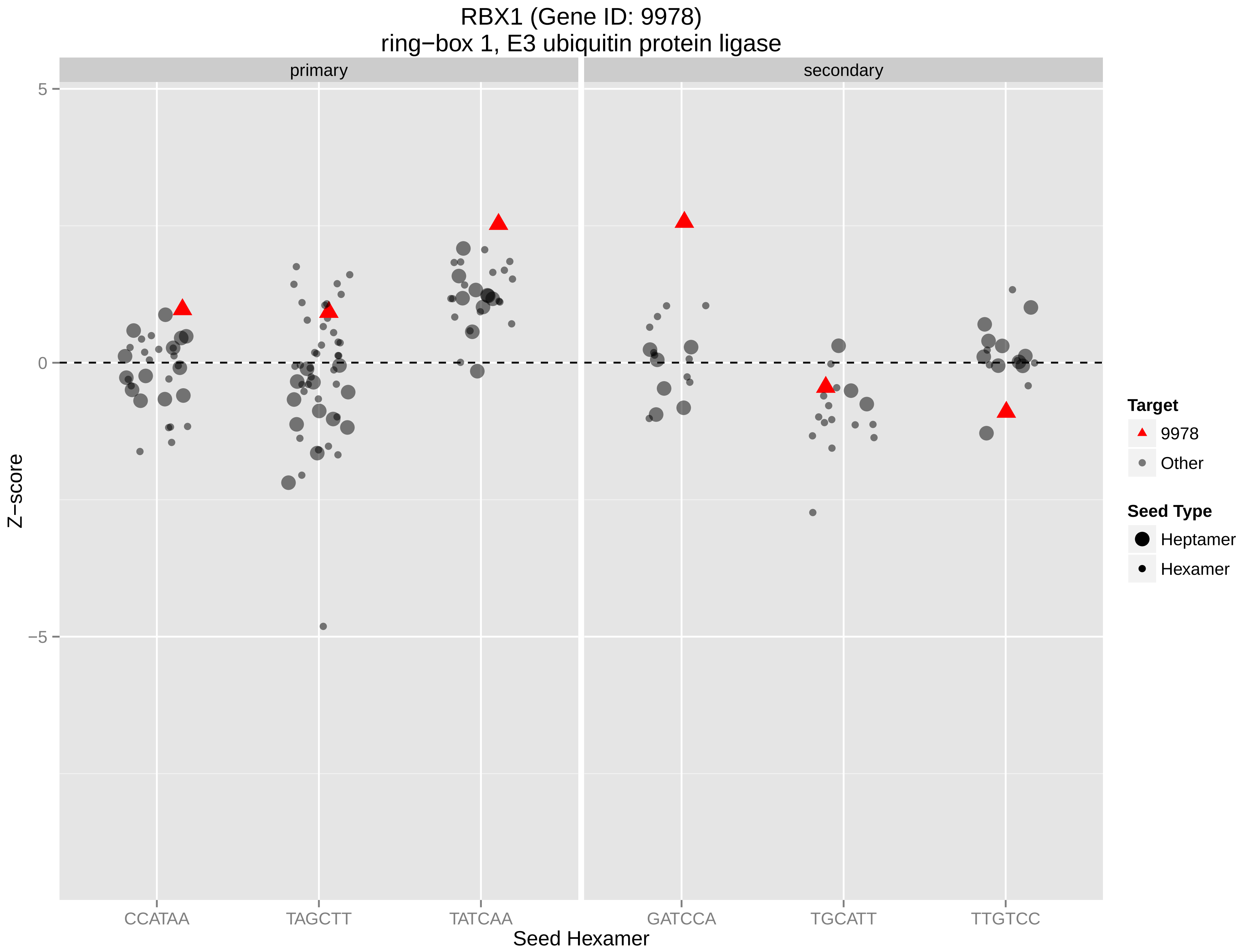
RBX1 (Gene ID: 9978)
ring−box 1, E3 ubiquitin protein ligase
primary
secondary
5
0
−5
Z−score
CCATAA
TAGCTT
TATCAA
GATCCA
TGCATT
TTGTCC
Seed Hexamer
Target
9978
Other
Seed Type
Heptamer
Hexamer

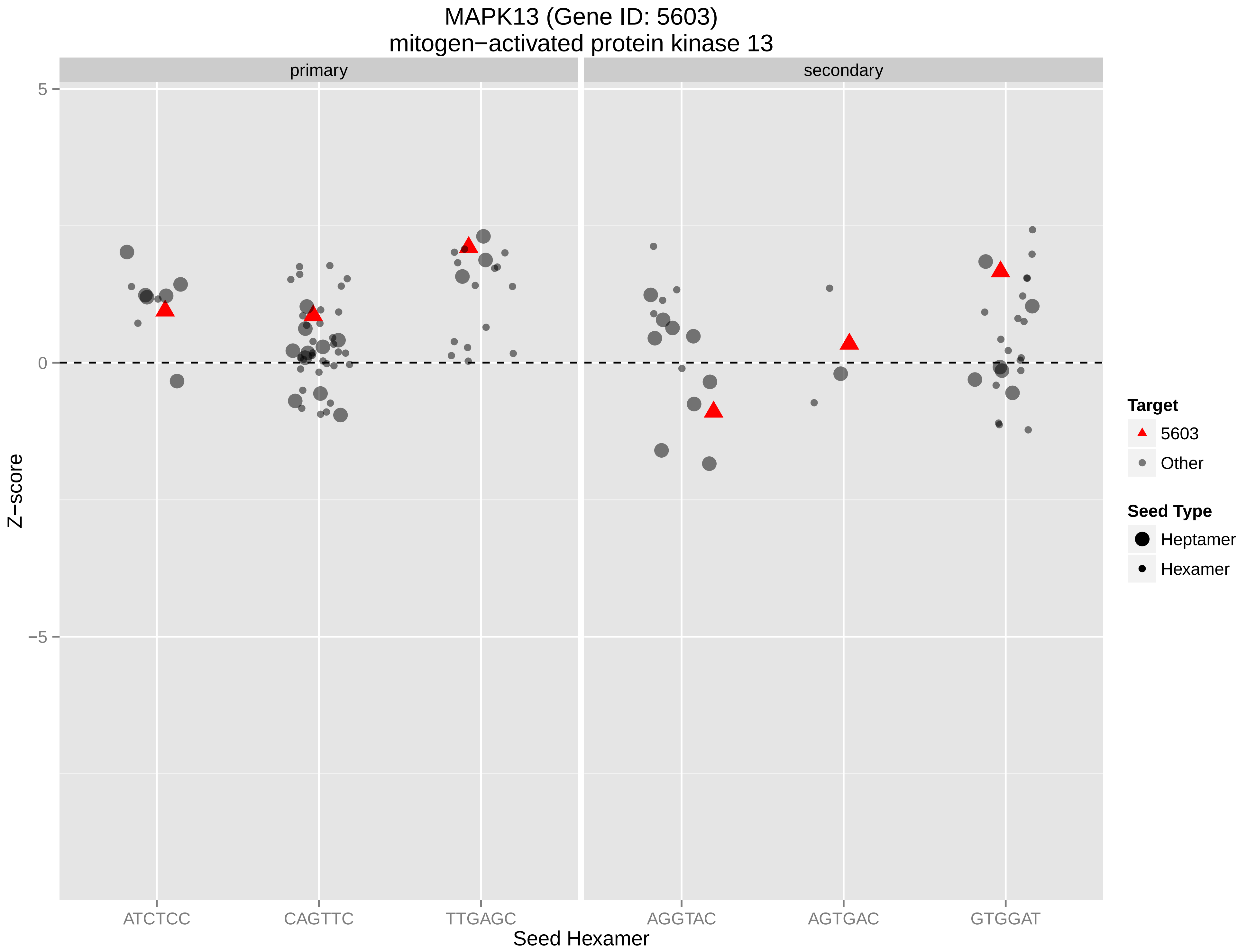

MAPK13 (Gene ID: 5603)
mitogen−activated protein kinase 13
primary
secondary
5
0
−5
Z−score
ATCTCC
CAGTTC
TTGAGC
AGGTAC
AGTGAC
GTGGAT
Seed Hexamer
Target
5603
Other
Seed Type
Heptamer
Hexamer

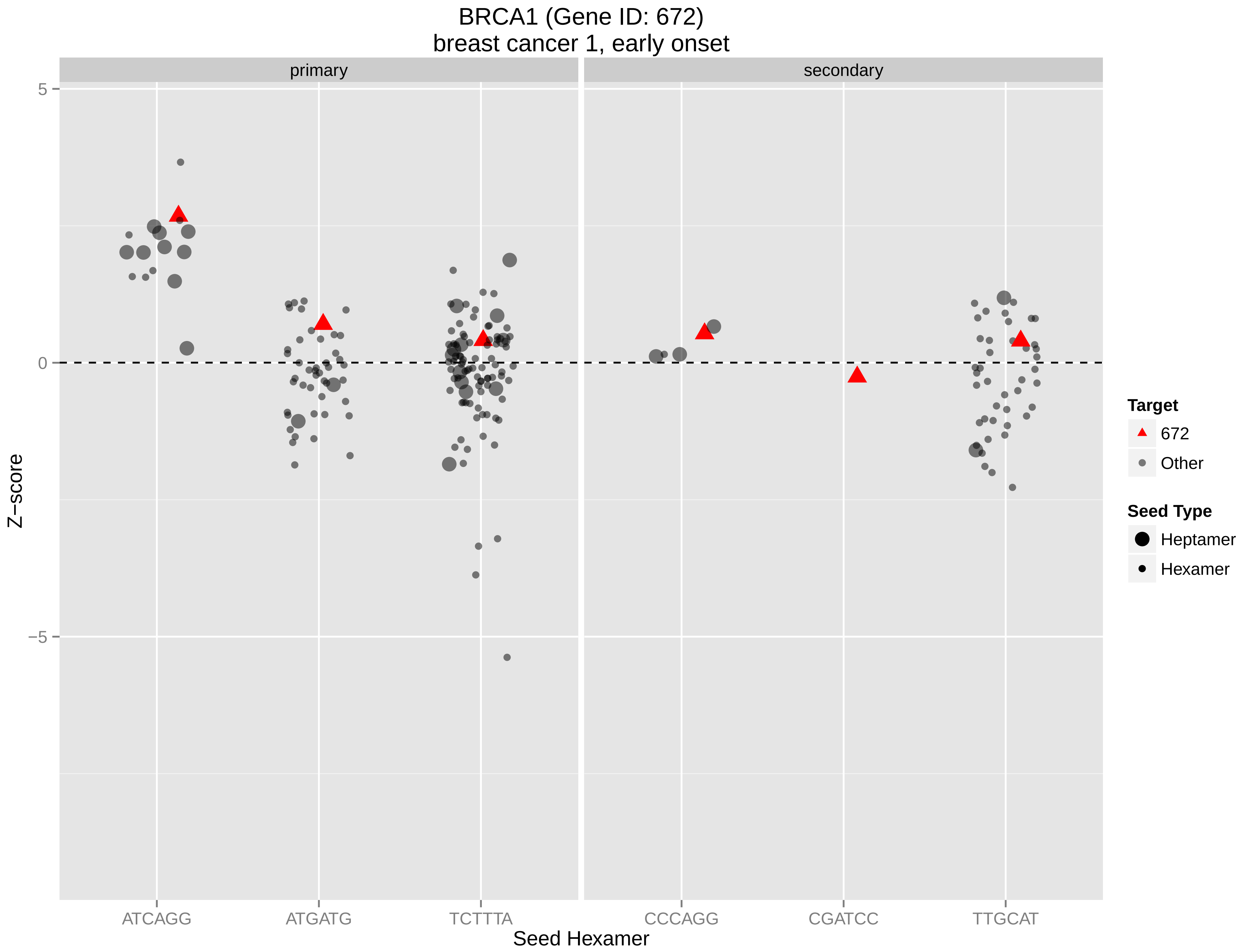
BRCA1 (Gene ID: 672)
breast cancer 1, early onset
primary
secondary
5
0
−5
Z−score
ATCAGG
ATGATG
TCTTTA
CCCAGG
CGATCC
TTGCAT
Seed Hexamer
Target
672
Other
Seed Type
Heptamer
Hexamer

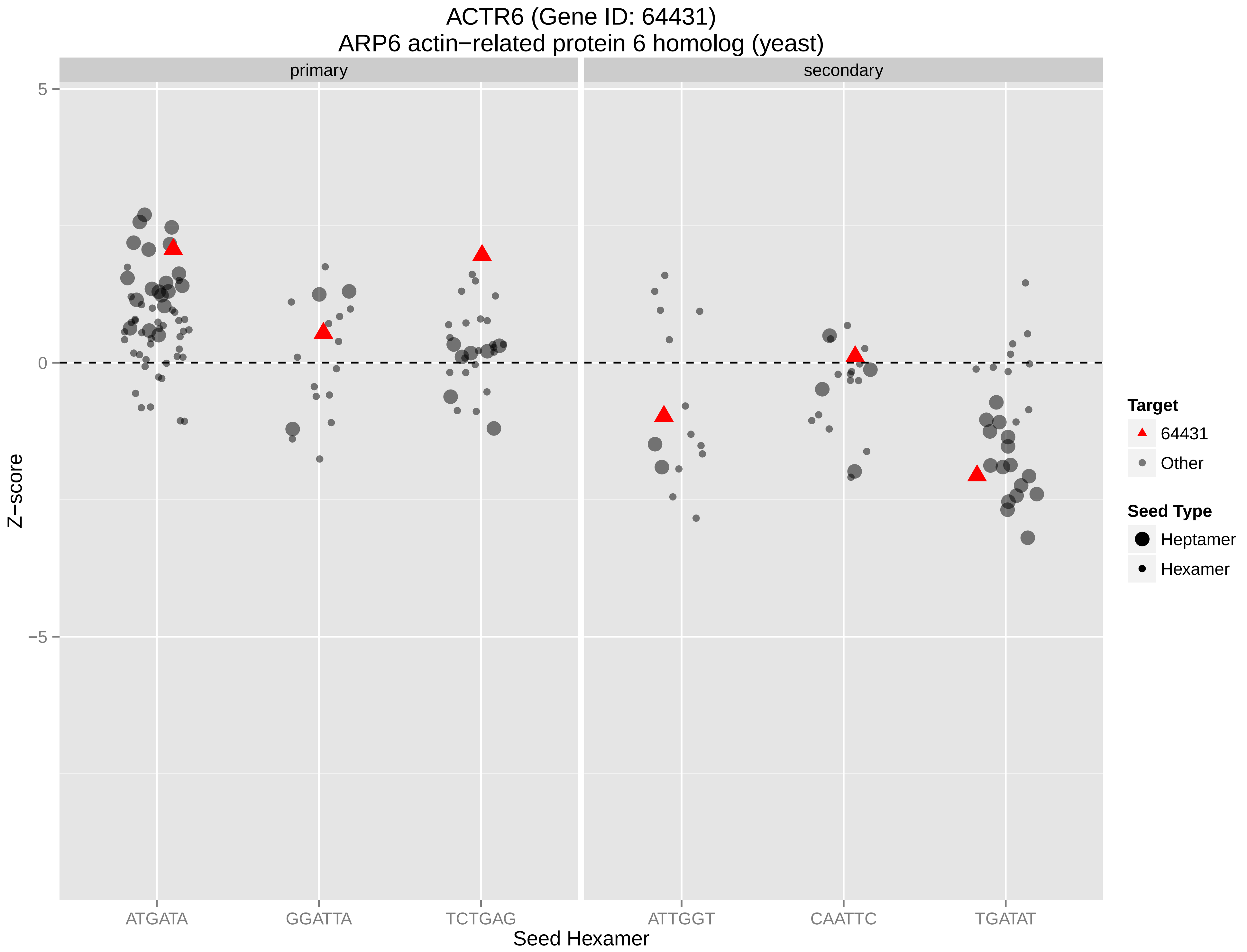
ACTR6 (Gene ID: 64431)
ARP6 actin−related protein 6 homolog (yeast)
primary
secondary
5
0
−5
Z−score
ATGATA
GGATTA
TCTGAG
ATTGGT
CAATTC
TGATAT
Seed Hexamer
Target
64431
Other
Seed Type
Heptamer
Hexamer

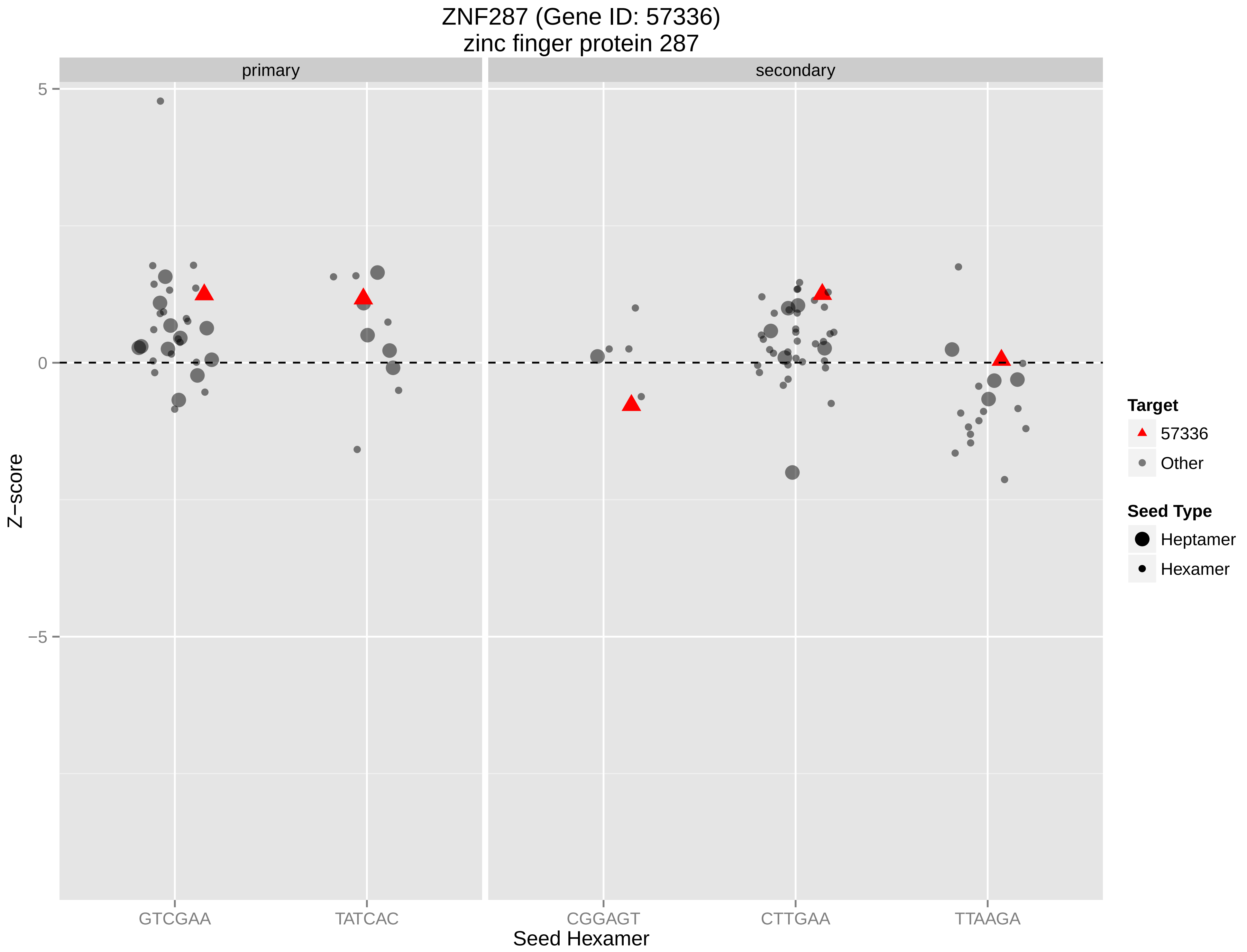

ZNF287 (Gene ID: 57336)
zinc finger protein 287
primary
secondary
5
0
−5
Z−score
GTCGAA
TATCAC
CGGAGT
CTTGAA
TTAAGA
Seed Hexamer
Target
57336
Other
Seed Type
Heptamer
Hexamer

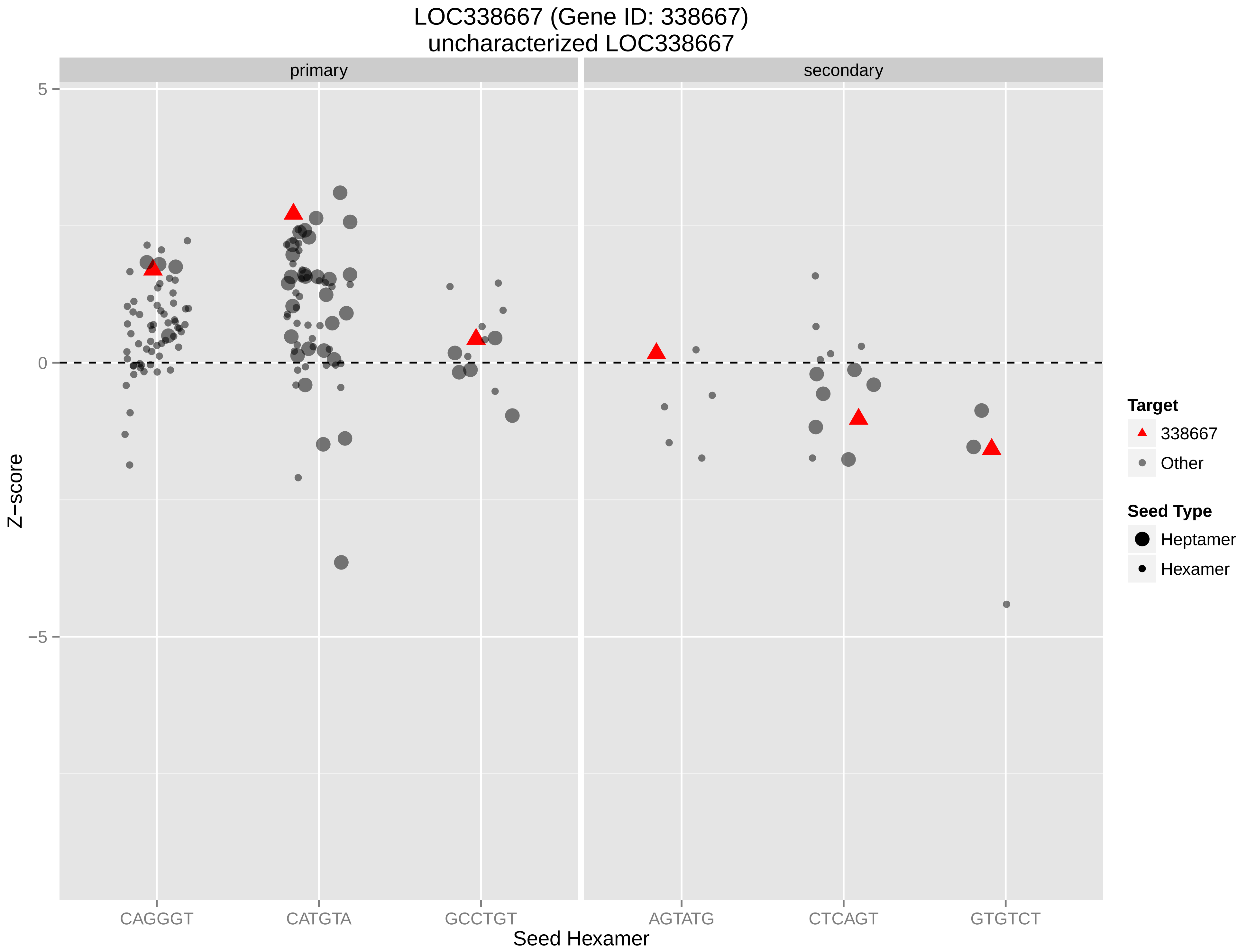

LOC338667 (Gene ID: 338667)
uncharacterized LOC338667
primary
secondary
5
0
−5
Z−score
CAGGGT
CATGTA
GCCTGT
AGTATG
CTCAGT
GTGTCT
Seed Hexamer
Target
338667
Other
Seed Type
Heptamer
Hexamer

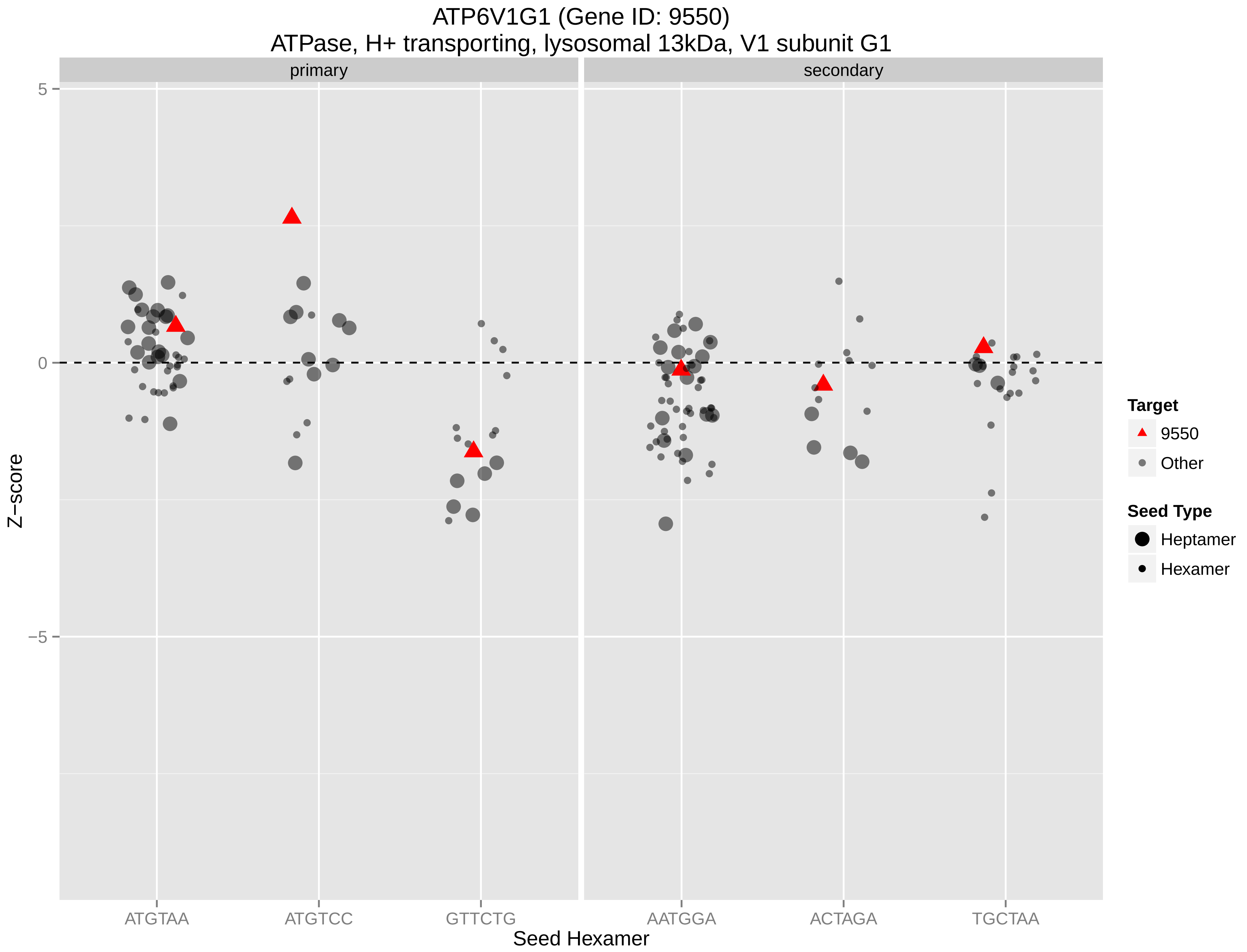
ATP6V1G1 (Gene ID: 9550)
ATPase, H+ transporting, lysosomal 13kDa, V1 subunit G1
primary
secondary
5
0
−5
Z−score
ATGTAA
ATGTCC
GTTCTG
AATGGA
ACTAGA
TGCTAA
Seed Hexamer
Target
9550
Other
Seed Type
Heptamer
Hexamer

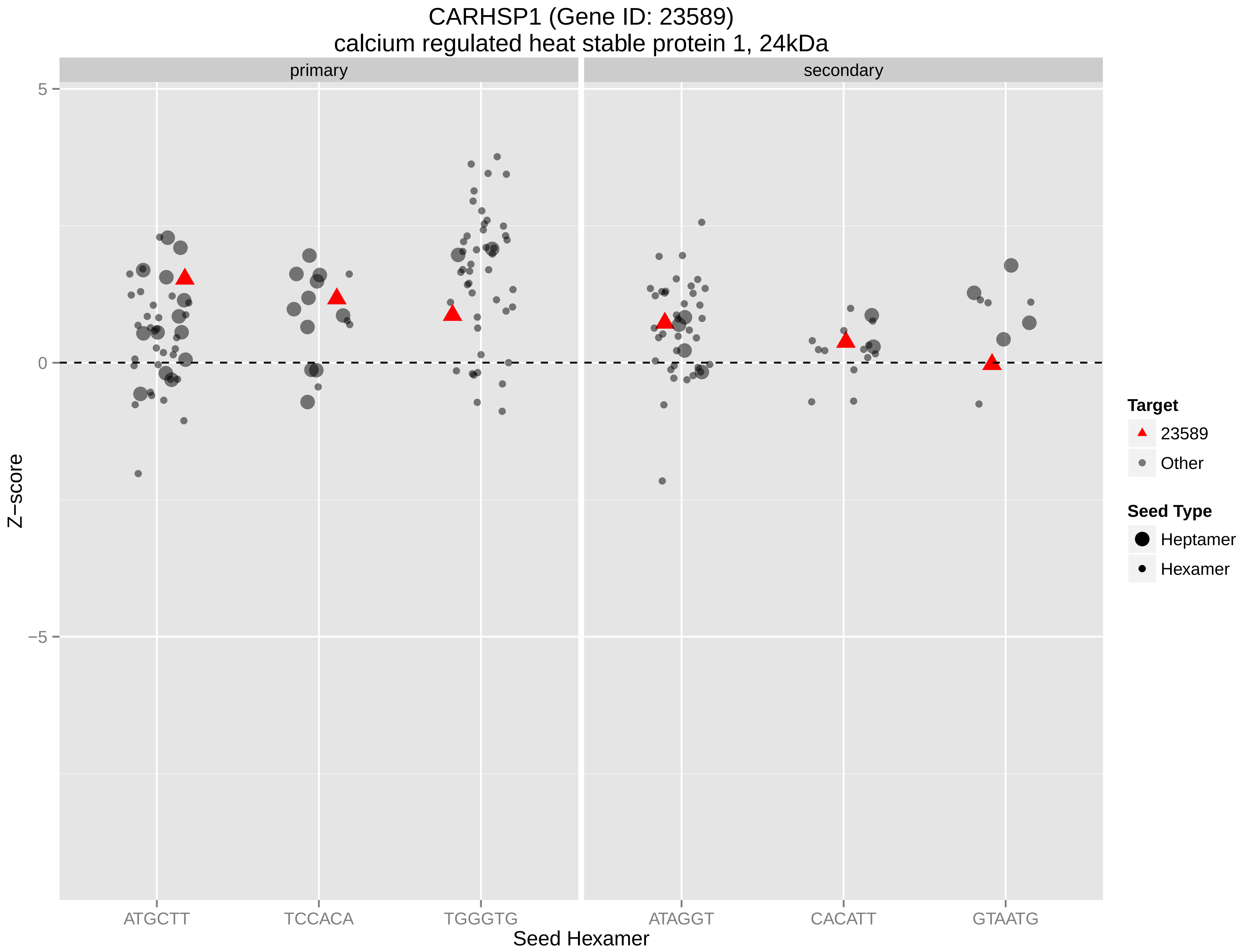

CARHSP1 (Gene ID: 23589)
calcium regulated heat stable protein 1, 24kDa
primary
secondary
5
0
−5
Z−score
ATGCTT
TCCACA
TGGGTG
ATAGGT
CACATT
GTAATG
Seed Hexamer
Target
23589
Other
Seed Type
Heptamer
Hexamer

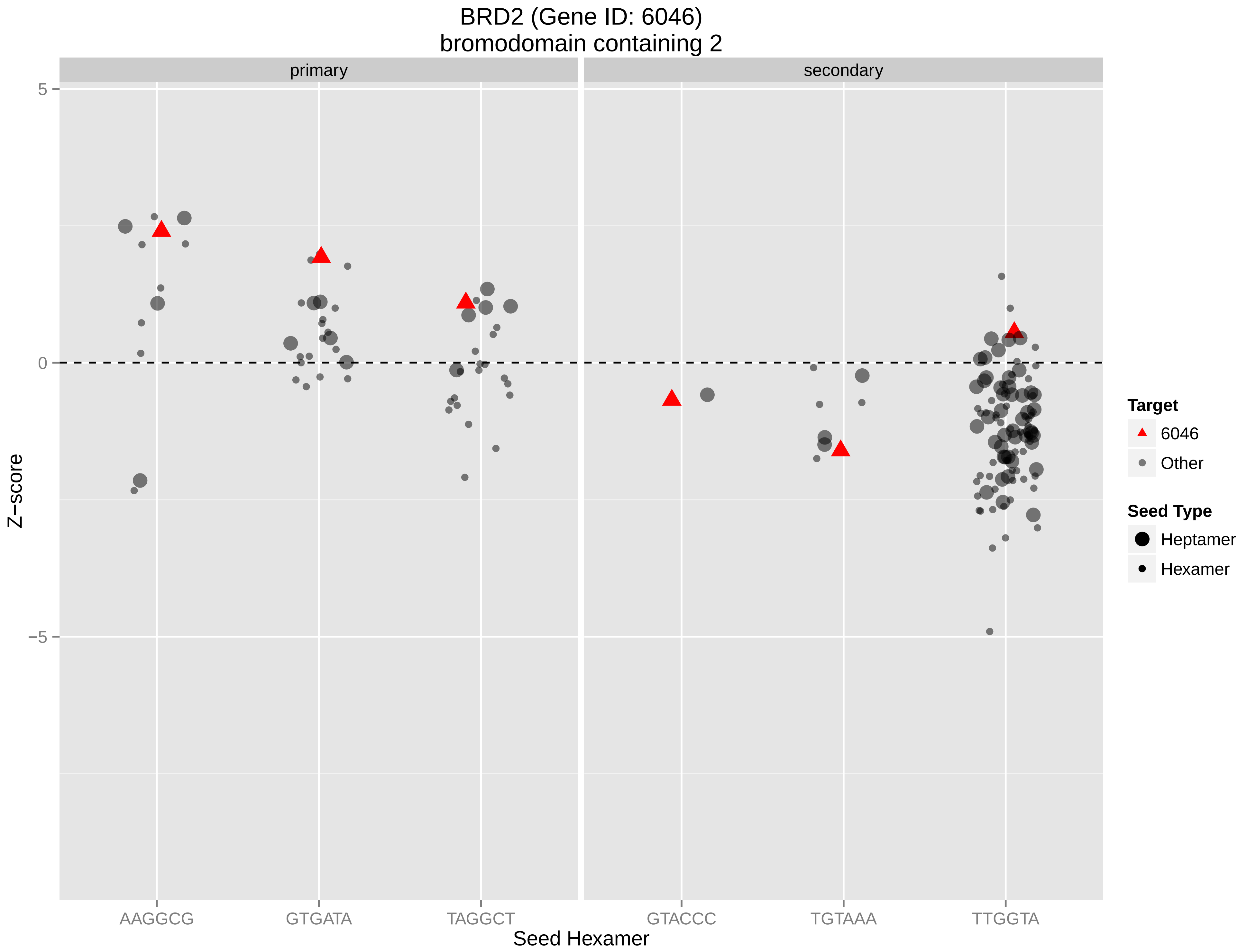
BRD2 (Gene ID: 6046)
bromodomain containing 2
primary
secondary
5
0
−5
Z−score
AAGGCG
GTGATA
TAGGCT
GTACCC
TGTAAA
TTGGTA
Seed Hexamer
Target
6046
Other
Seed Type
Heptamer
Hexamer

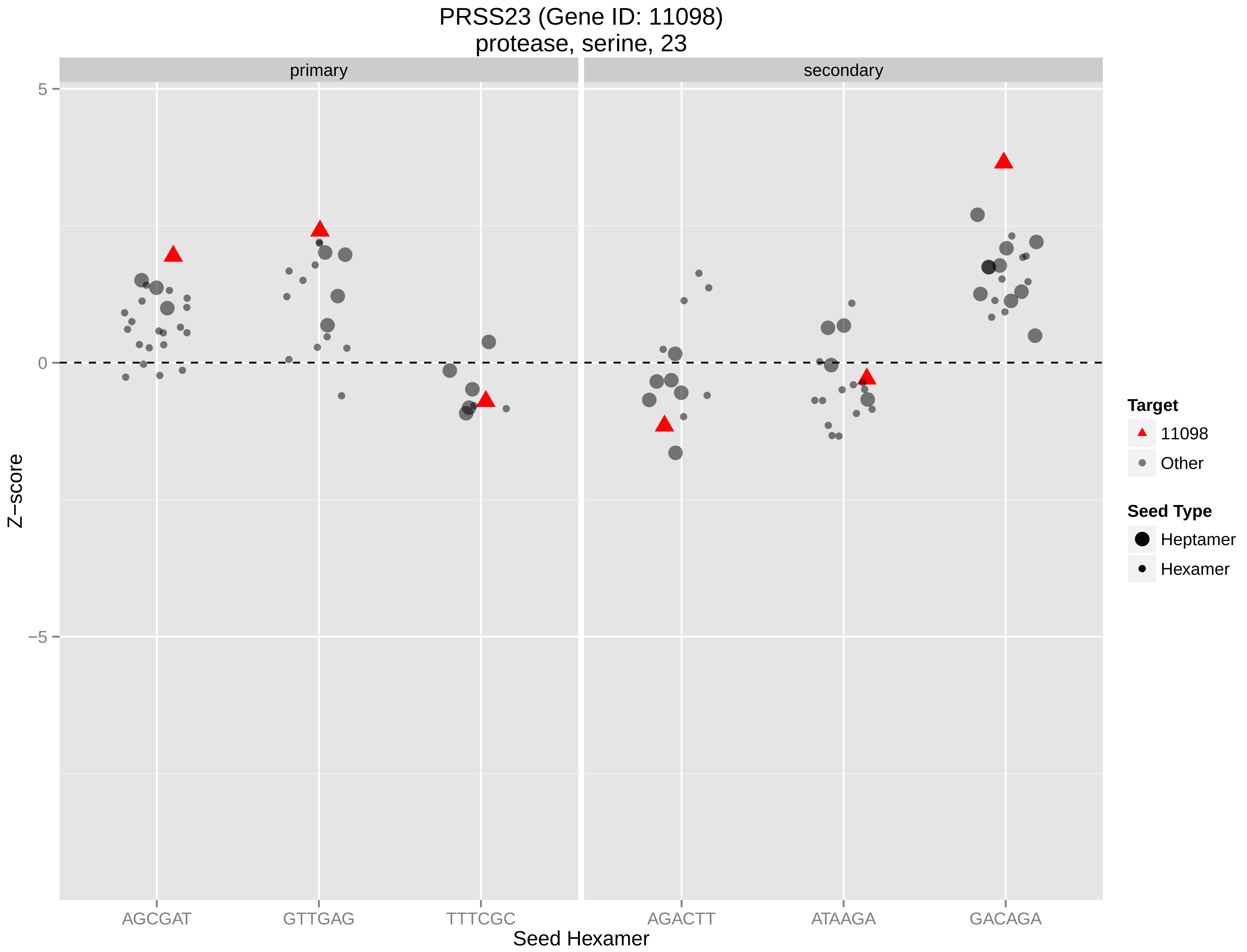
PRSS23 (Gene ID: 11098)
protease, serine, 23
primary
secondary
5
0
−5
Z−score
AGCGAT
GTTGAG
TTTCGC
AGACTT
ATAAGA
GACAGA
Seed Hexamer
Target
11098
Other
Seed Type
Heptamer
Hexamer

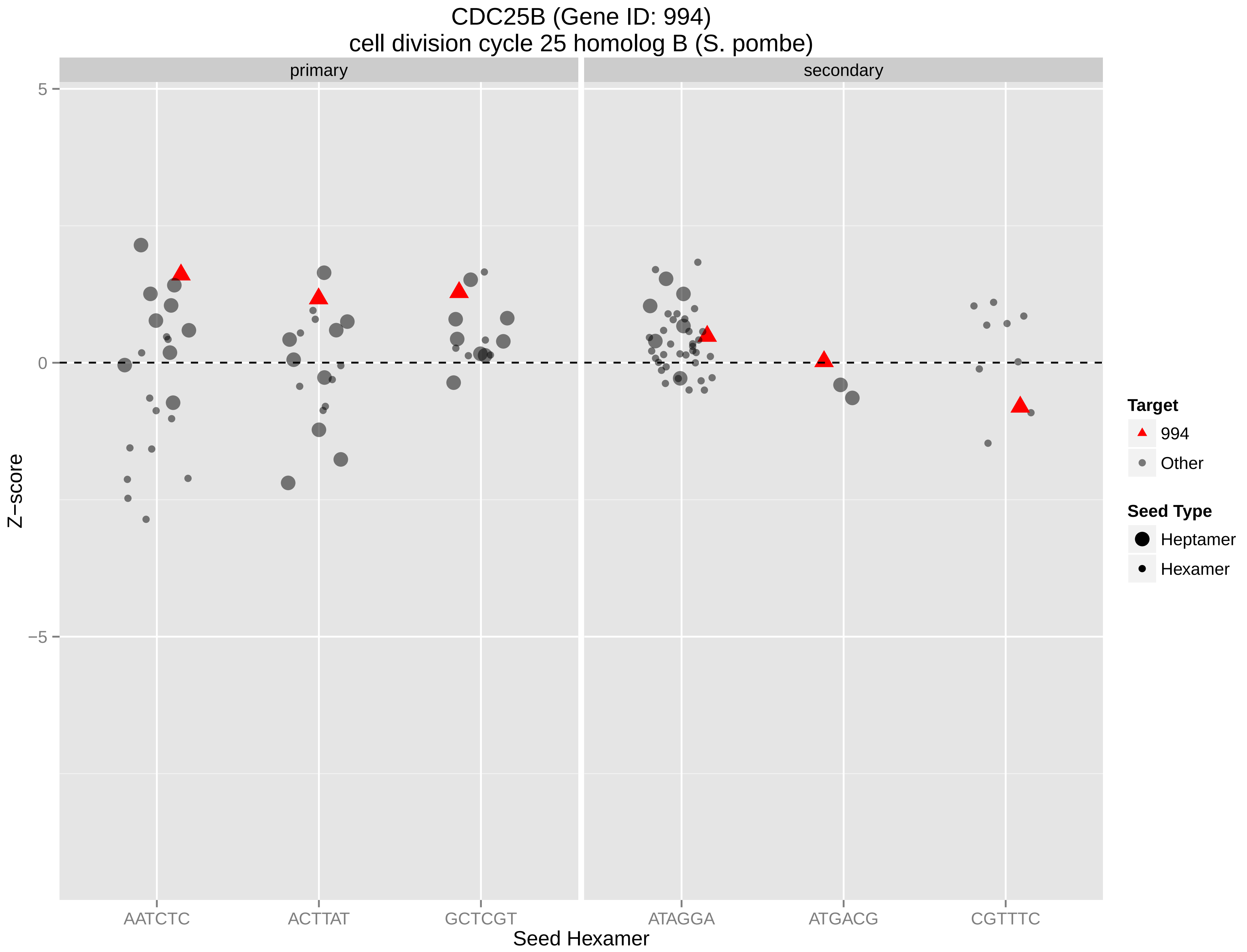

CDC25B (Gene ID: 994)
cell division cycle 25 homolog B (S. pombe)
primary
secondary
5
0
−5
Z−score
AATCTC
ACTTAT
GCTCGT
ATAGGA
ATGACG
CGTTTC
Seed Hexamer
Target
994
Other
Seed Type
Heptamer
Hexamer

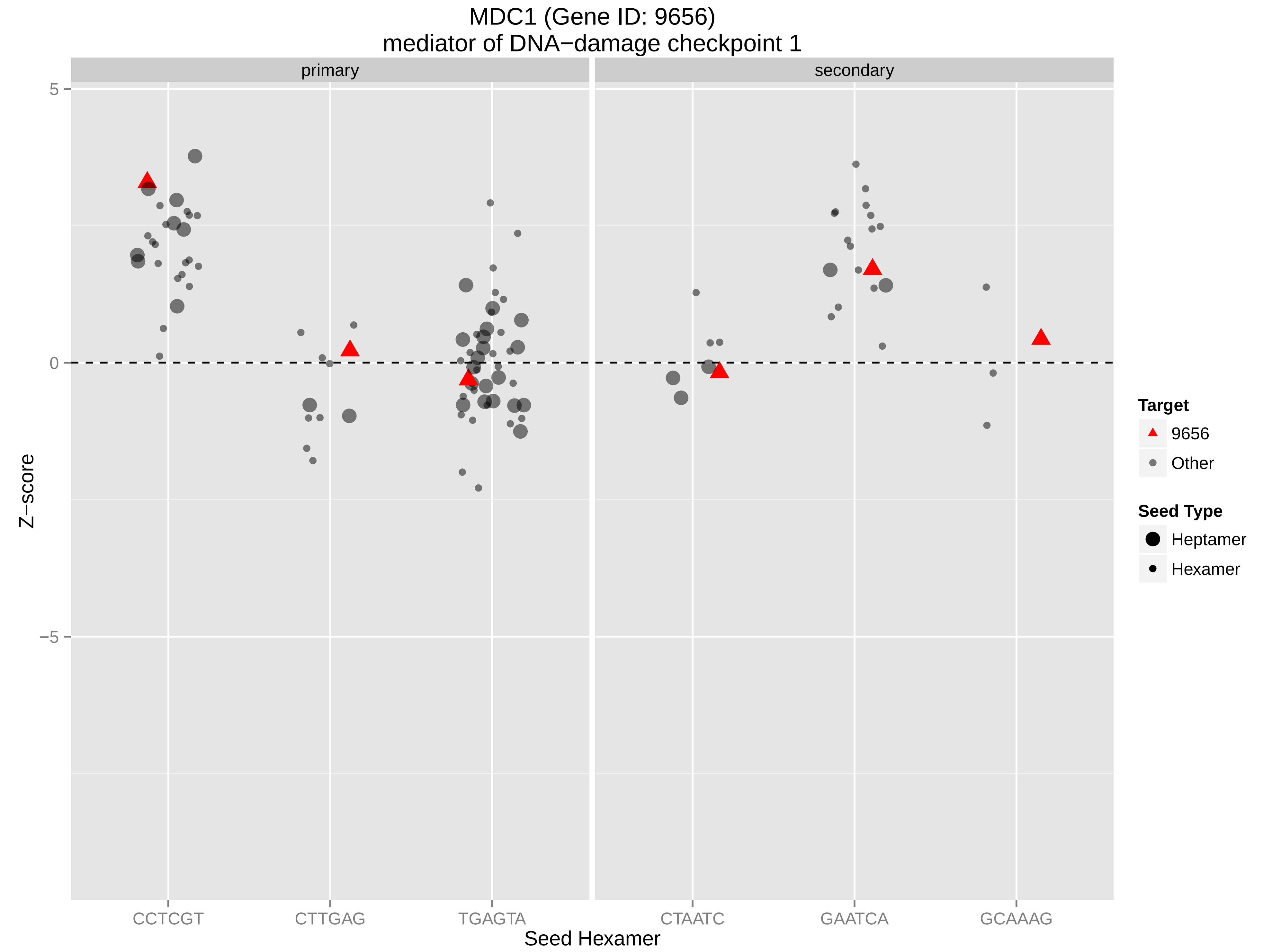

MDC1 (Gene ID: 9656)
mediator of DNA−damage checkpoint 1
primary
secondary
5
0
−5
Z−score
CCTCGT
CTTGAG
TGAGTA
CTAATC
GAATCA
GCAAAG
Seed Hexamer
Target
9656
Other
Seed Type
Heptamer
Hexamer

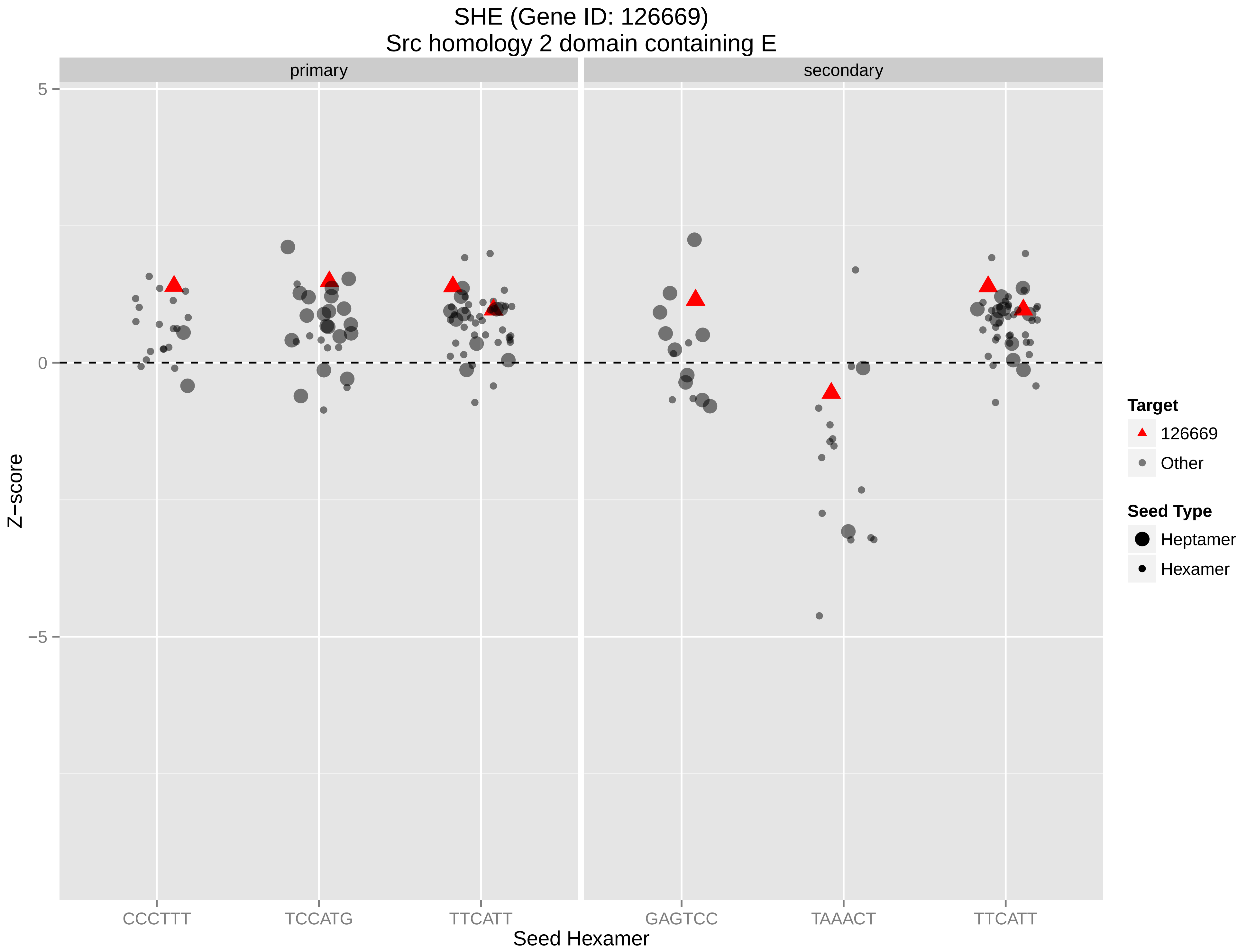
SHE (Gene ID: 126669)
Src homology 2 domain containing E
primary
secondary
5
0
−5
Z−score
CCCTTT
TCCATG
TTCATT
GAGTCC
TAAACT
TTCATT
Seed Hexamer
Target
126669
Other
Seed Type
Heptamer
Hexamer

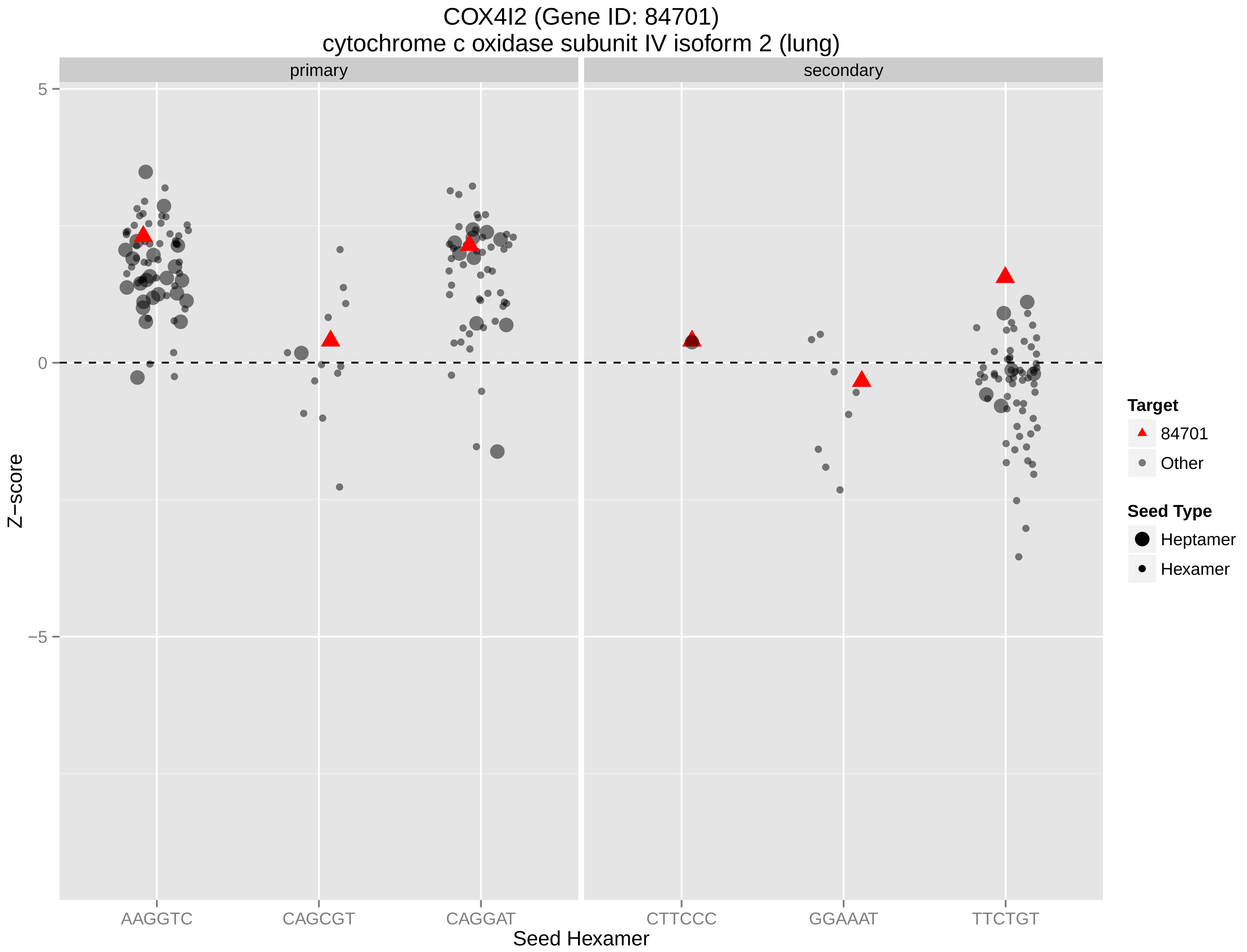
COX4I2 (Gene ID: 84701)
cytochrome c oxidase subunit IV isoform 2 (lung)
primary
secondary
5
0
−5
Z−score
AAGGTC
CAGCGT
CAGGAT
CTTCCC
GGAAAT
TTCTGT
Seed Hexamer
Target
84701
Other
Seed Type
Heptamer
Hexamer

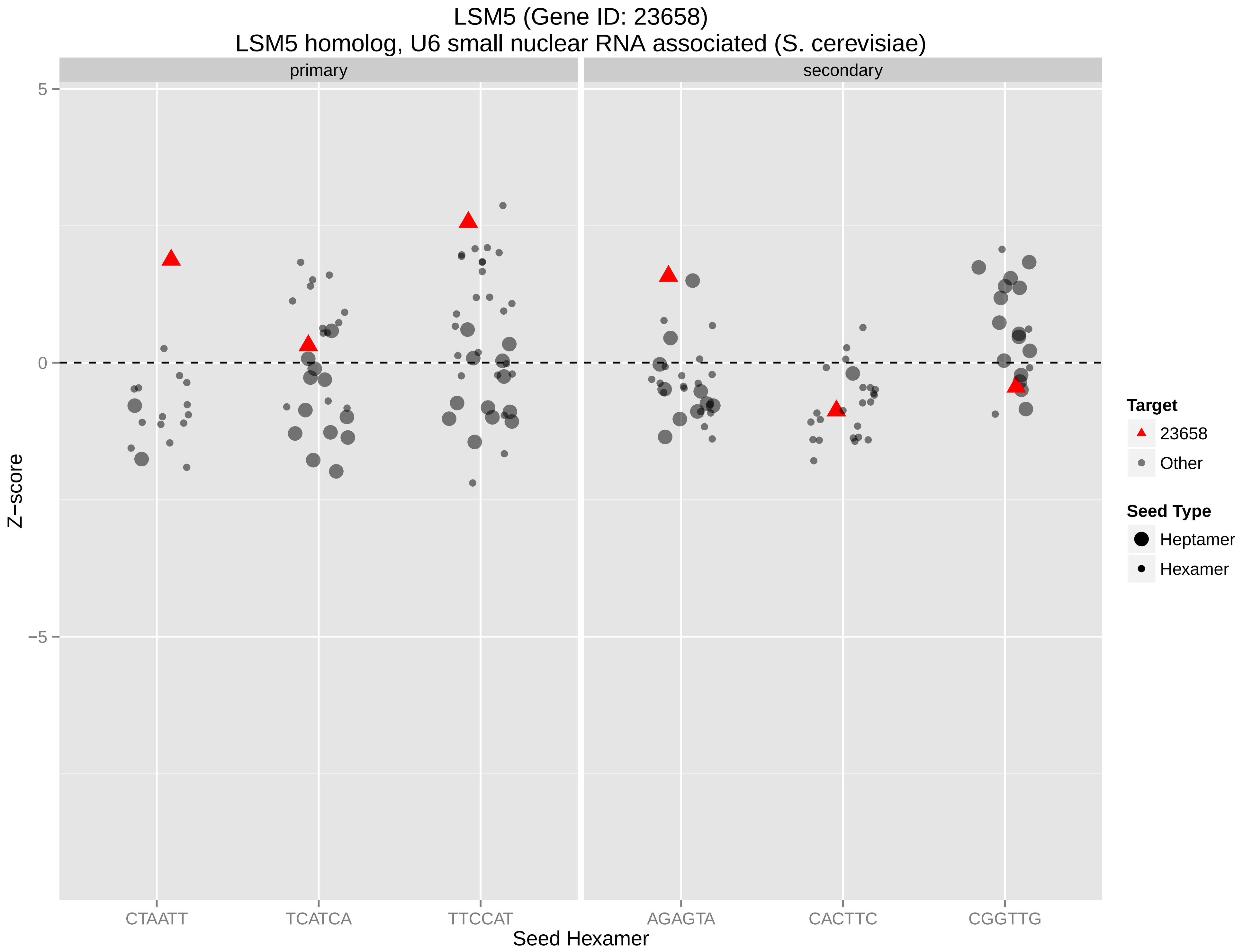
LSM5 (Gene ID: 23658)
LSM5 homolog, U6 small nuclear RNA associated (S. cerevisiae)
primary
secondary
5
0
−5
Z−score
CTAATT
TCATCA
TTCCAT
AGAGTA
CACTTC
CGGTTG
Seed Hexamer
Target
23658
Other
Seed Type
Heptamer
Hexamer

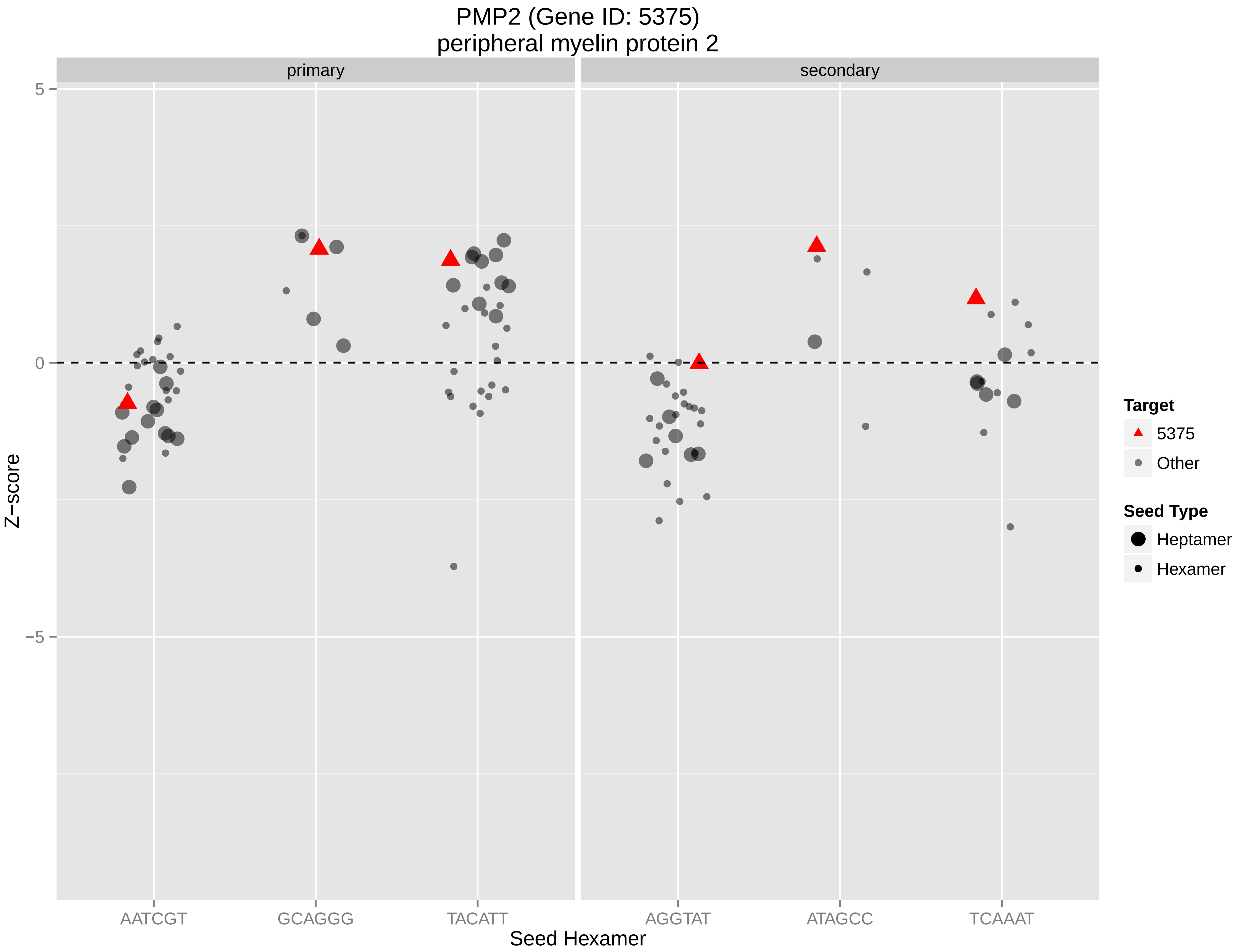
PMP2 (Gene ID: 5375)
peripheral myelin protein 2
primary
secondary
5
0
−5
Z−score
AATCGT
GCAGGG
TACATT
AGGTAT
ATAGCC
TCAAAT
Seed Hexamer
Target
5375
Other
Seed Type
Heptamer
Hexamer

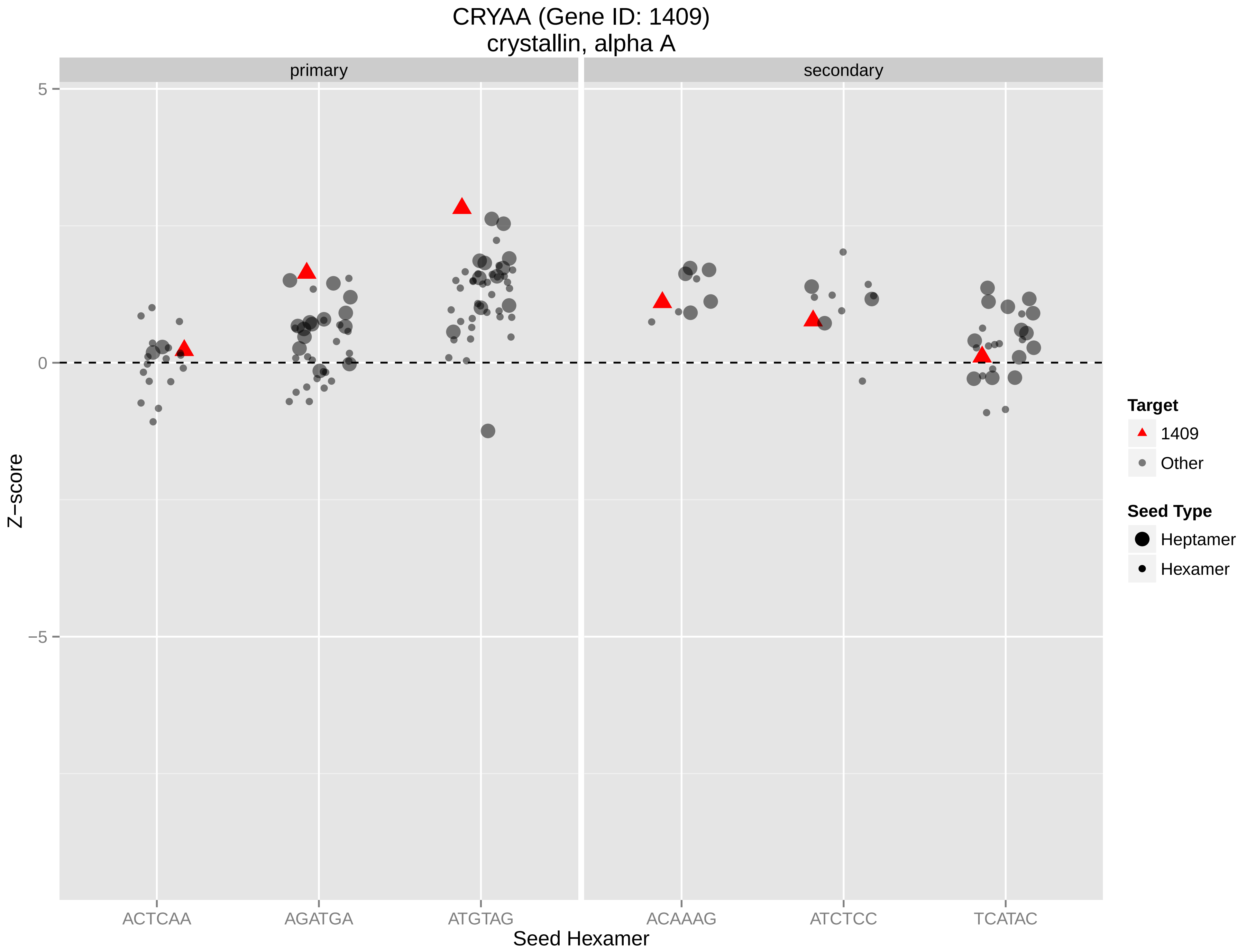

CRYAA (Gene ID: 1409)
crystallin, alpha A
primary
secondary
5
0
−5
Z−score
ACTCAA
AGATGA
ATGTAG
ACAAAG
ATCTCC
TCATAC
Seed Hexamer
Target
1409
Other
Seed Type
Heptamer
Hexamer

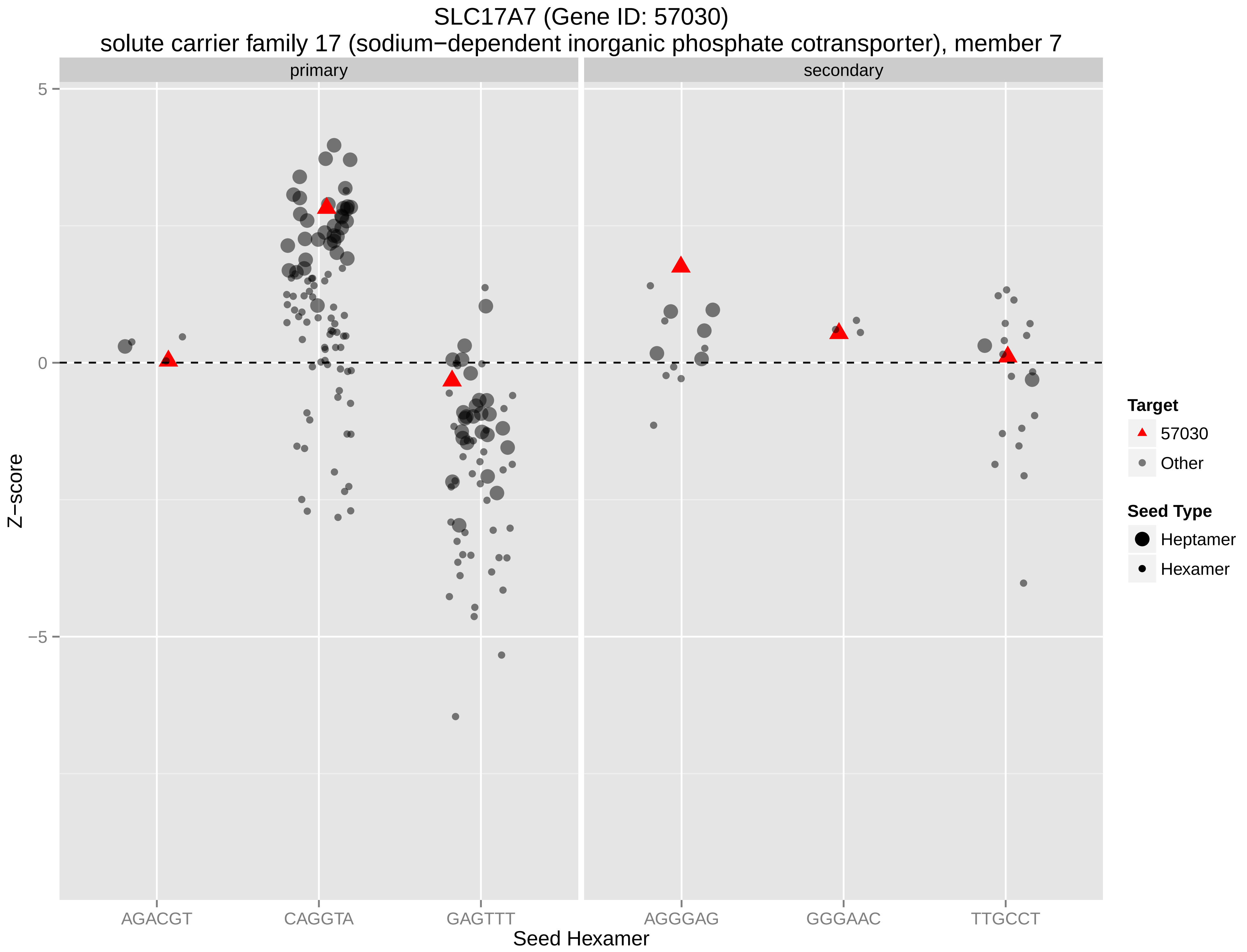
SLC17A7 (Gene ID: 57030)
solute carrier family 17 (sodium−dependent inorganic phosphate cotransporter), member 7
primary
secondary
5
0
−5
Z−score
AGACGT
CAGGTA
GAGTTT
AGGGAG
GGGAAC
TTGCCT
Seed Hexamer
Target
57030
Other
Seed Type
Heptamer
Hexamer

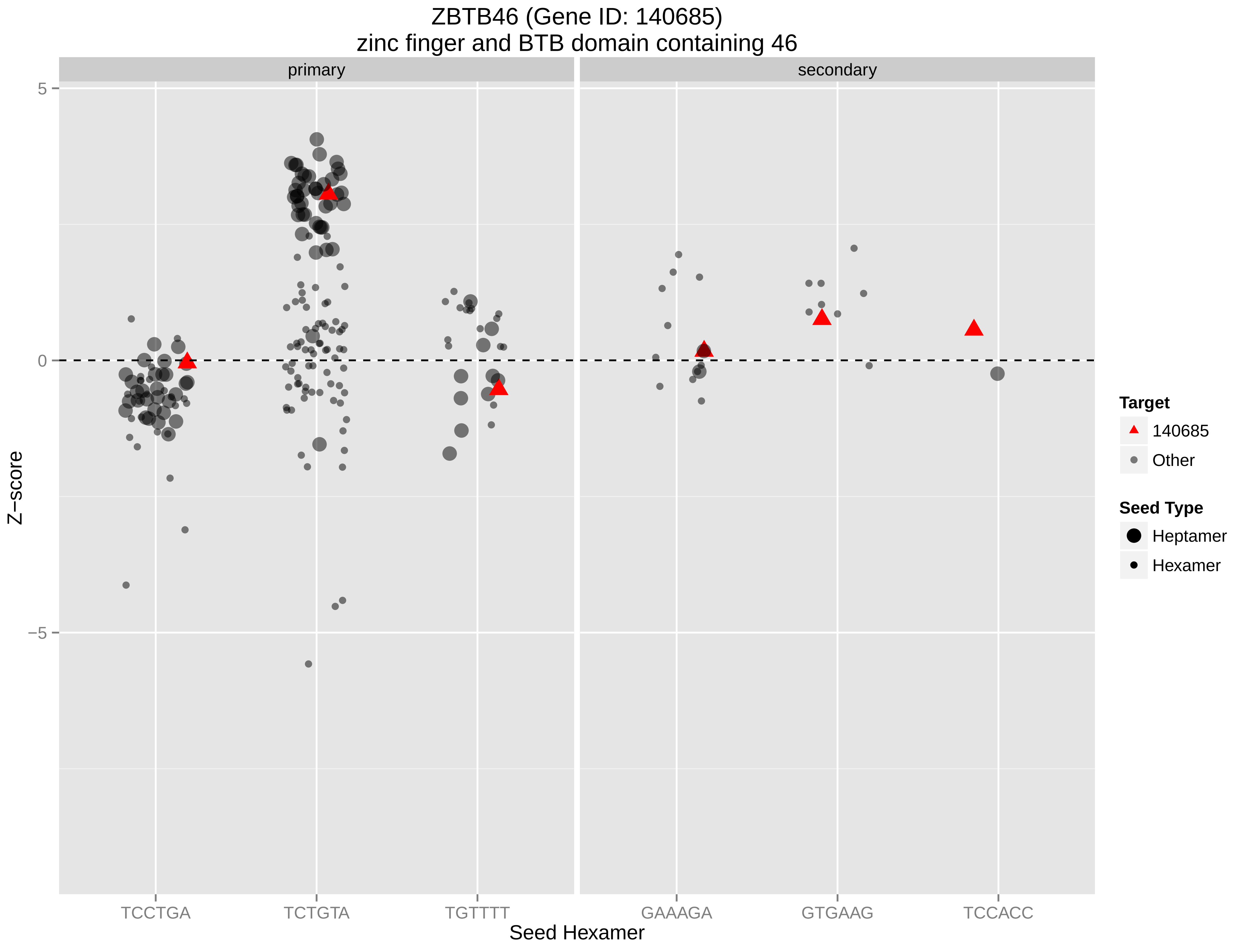

ZBTB46 (Gene ID: 140685)
zinc finger and BTB domain containing 46
primary
secondary
5
0
−5
Z−score
TCCTGA
TCTGTA
TGTTTT
GAAAGA
GTGAAG
TCCACC
Seed Hexamer
Target
140685
Other
Seed Type
Heptamer
Hexamer

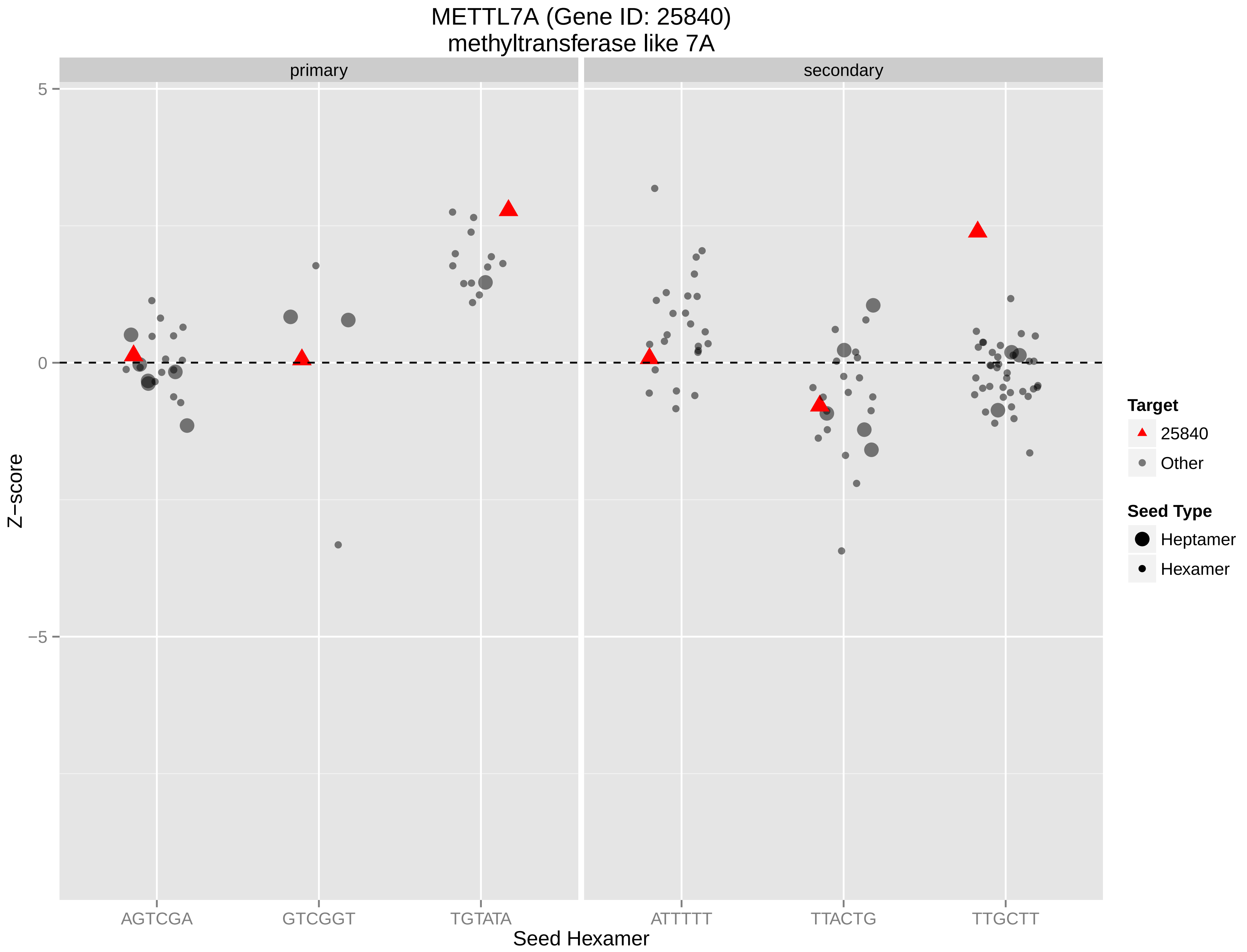

METTL7A (Gene ID: 25840)
methyltransferase like 7A
primary
secondary
5
0
−5
Z−score
AGTCGA
GTCGGT
TGTATA
ATTTTT
TTACTG
TTGCTT
Seed Hexamer
Target
25840
Other
Seed Type
Heptamer
Hexamer

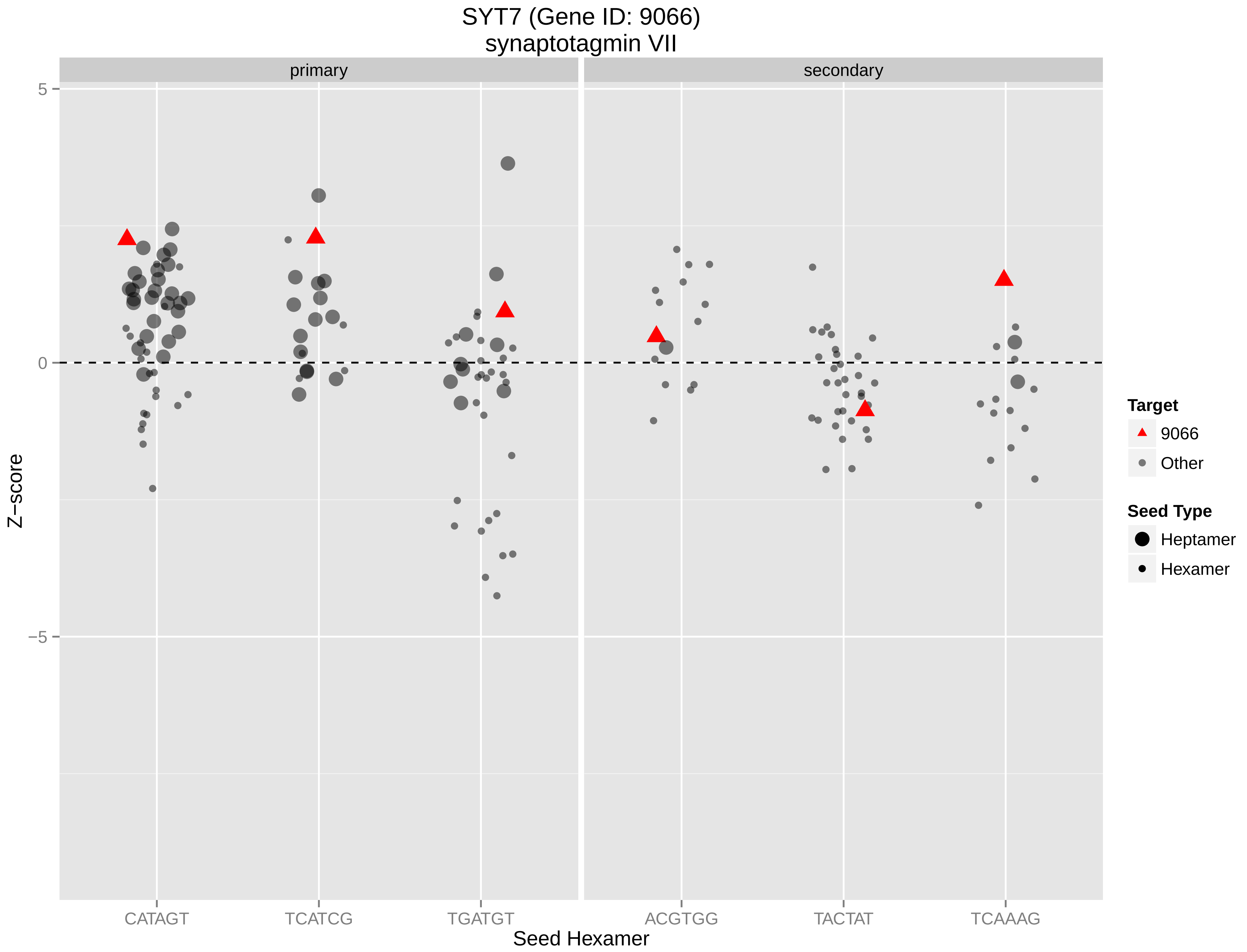
SYT7 (Gene ID: 9066)
synaptotagmin VII
primary
secondary
5
0
−5
Z−score
CATAGT
TCATCG
TGATGT
ACGTGG
TACTAT
TCAAAG
Seed Hexamer
Target
9066
Other
Seed Type
Heptamer
Hexamer

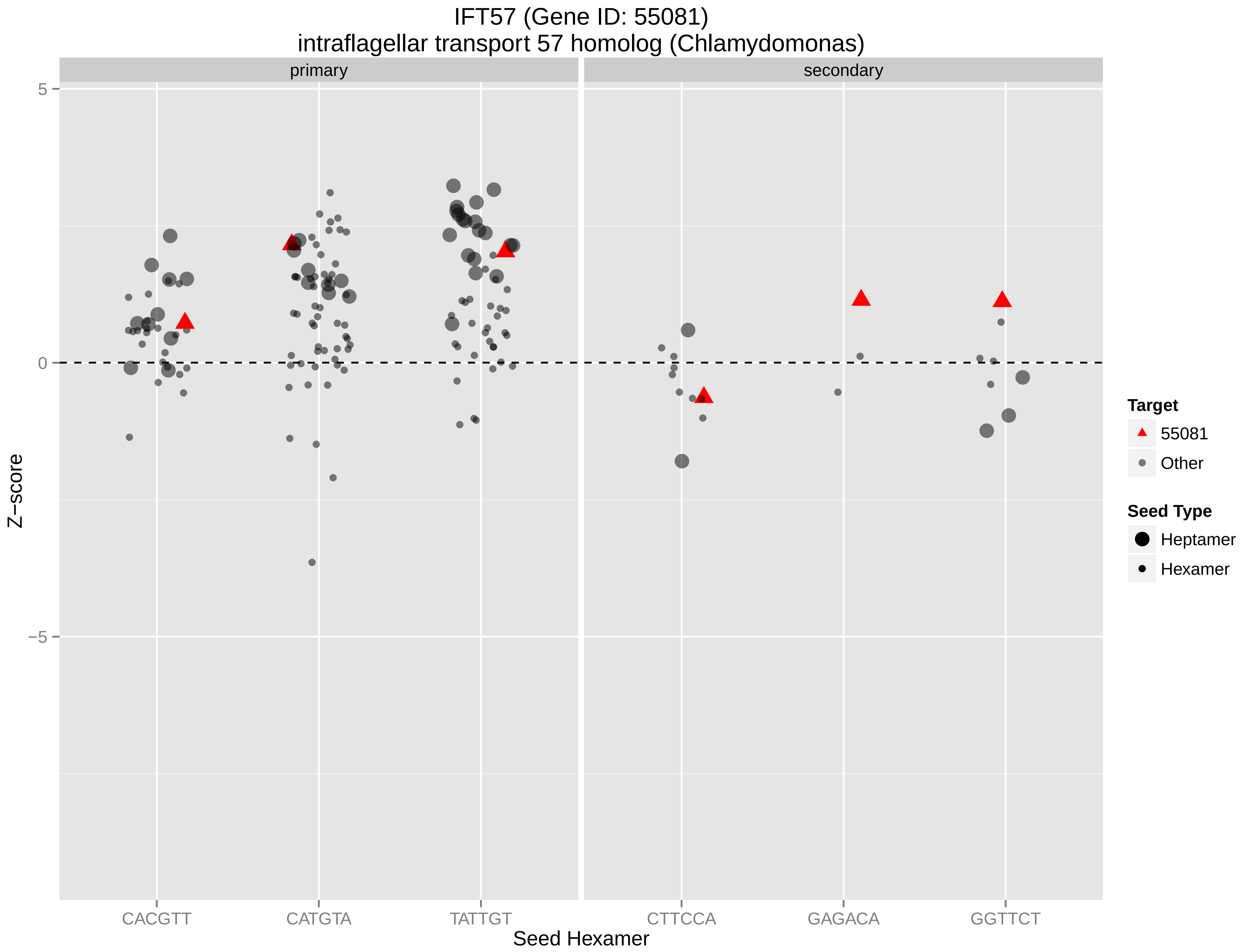

IFT57 (Gene ID: 55081)
intraflagellar transport 57 homolog (Chlamydomonas)
primary
secondary
5
0
−5
Z−score
CACGTT
CATGTA
TATTGT
CTTCCA
GAGACA
GGTTCT
Seed Hexamer
Target
55081
Other
Seed Type
Heptamer
Hexamer

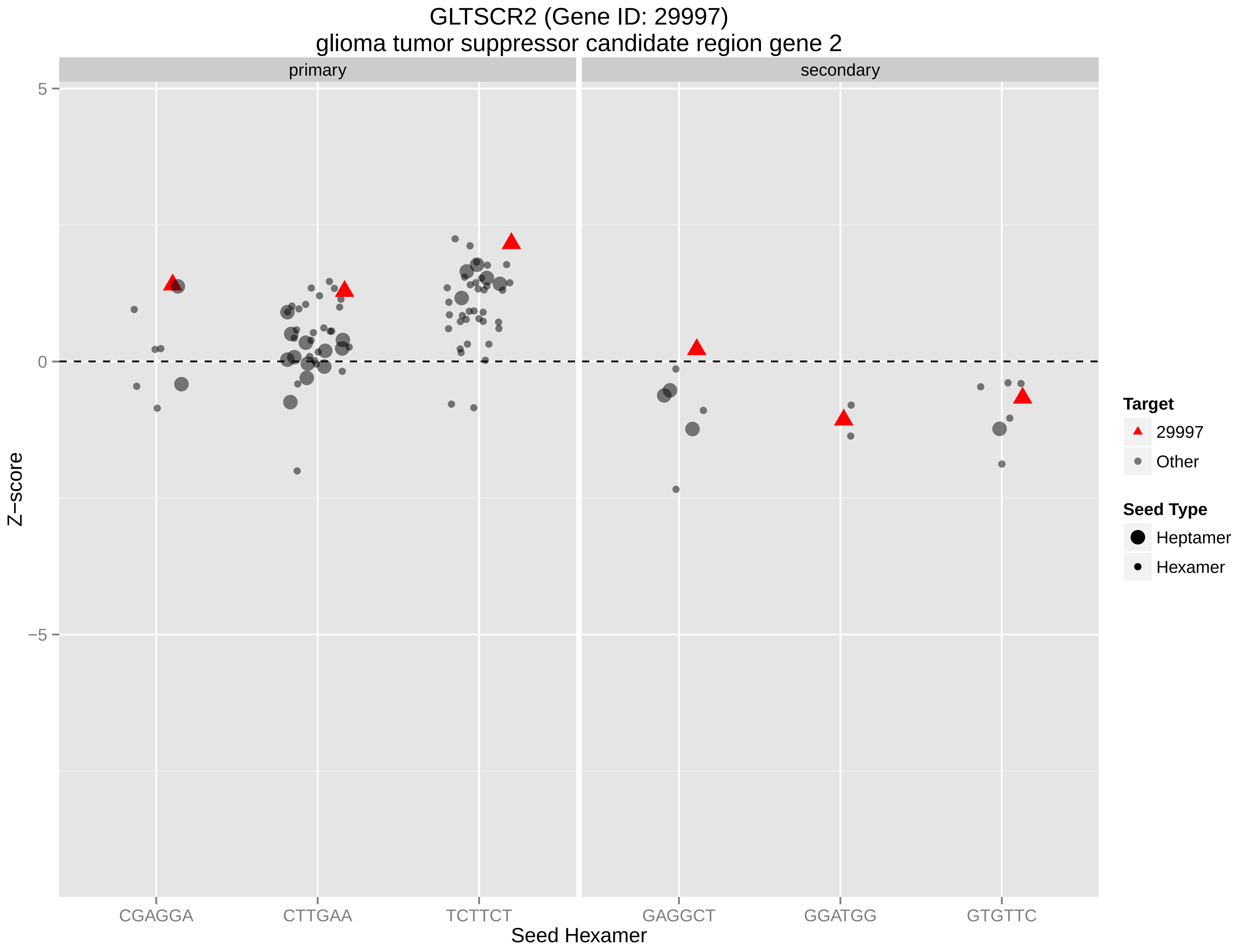
GLTSCR2 (Gene ID: 29997)
glioma tumor suppressor candidate region gene 2
primary
secondary
5
0
−5
Z−score
CGAGGA
CTTGAA
TCTTCT
GAGGCT
GGATGG
GTGTTC
Seed Hexamer
Target
29997
Other
Seed Type
Heptamer
Hexamer

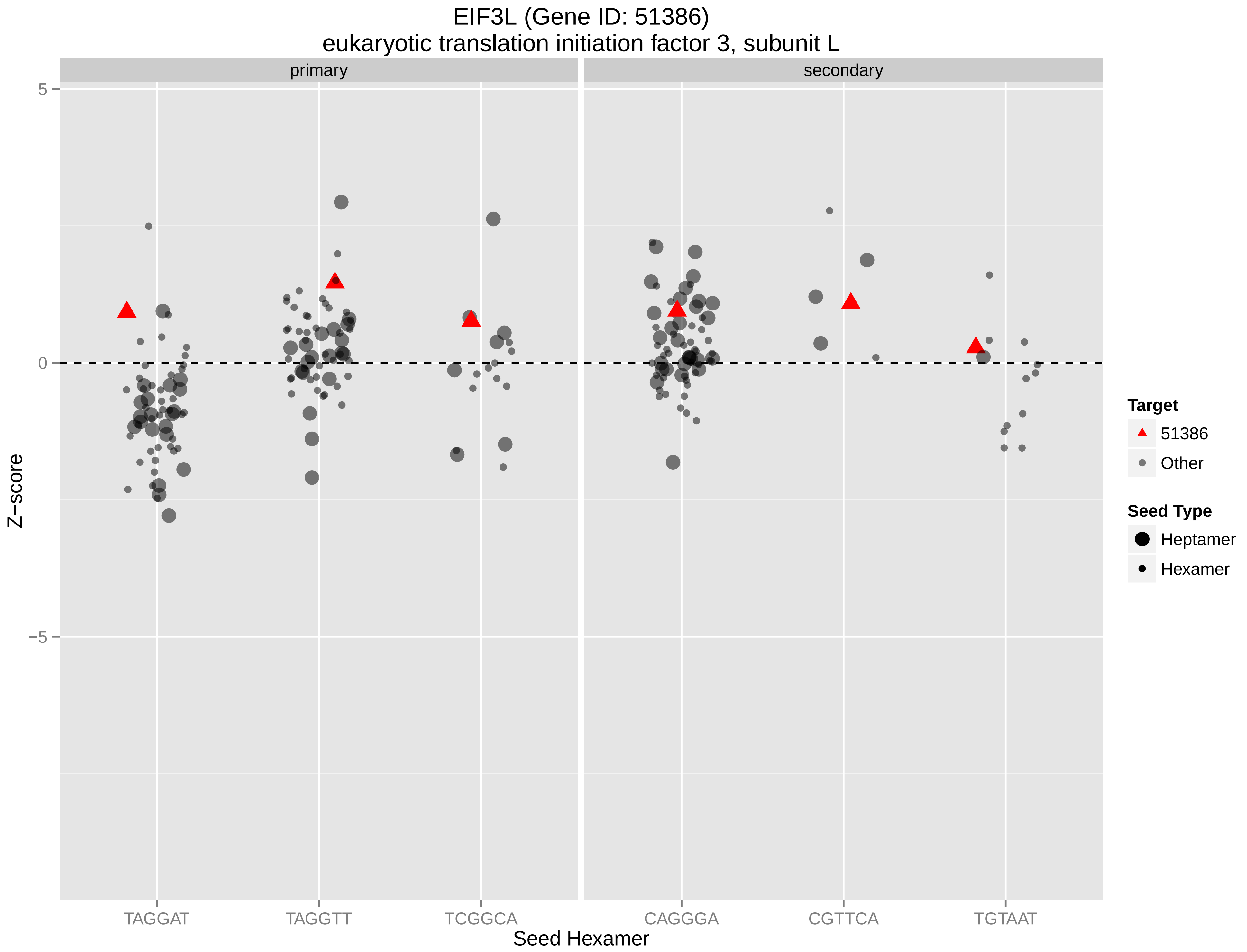
EIF3L (Gene ID: 51386)
eukaryotic translation initiation factor 3, subunit L
primary
secondary
5
0
−5
Z−score
TAGGAT
TAGGTT
TCGGCA
CAGGGA
CGTTCA
TGTAAT
Seed Hexamer
Target
51386
Other
Seed Type
Heptamer
Hexamer

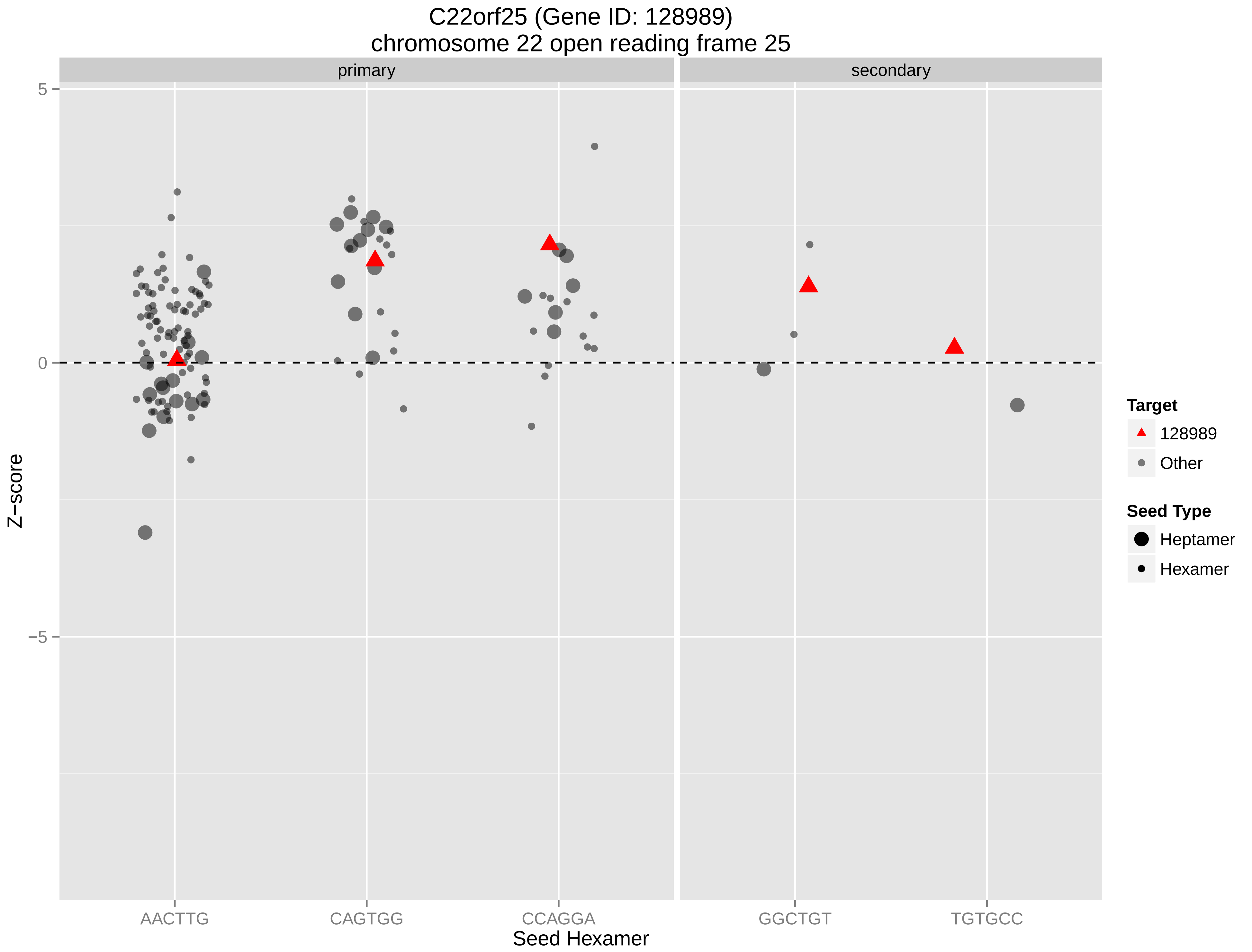
C22orf25 (Gene ID: 128989)
chromosome 22 open reading frame 25
primary
secondary
5
0
−5
Z−score
AACTTG
CAGTGG
CCAGGA
GGCTGT
TGTGCC
Seed Hexamer
Target
128989
Other
Seed Type
Heptamer
Hexamer

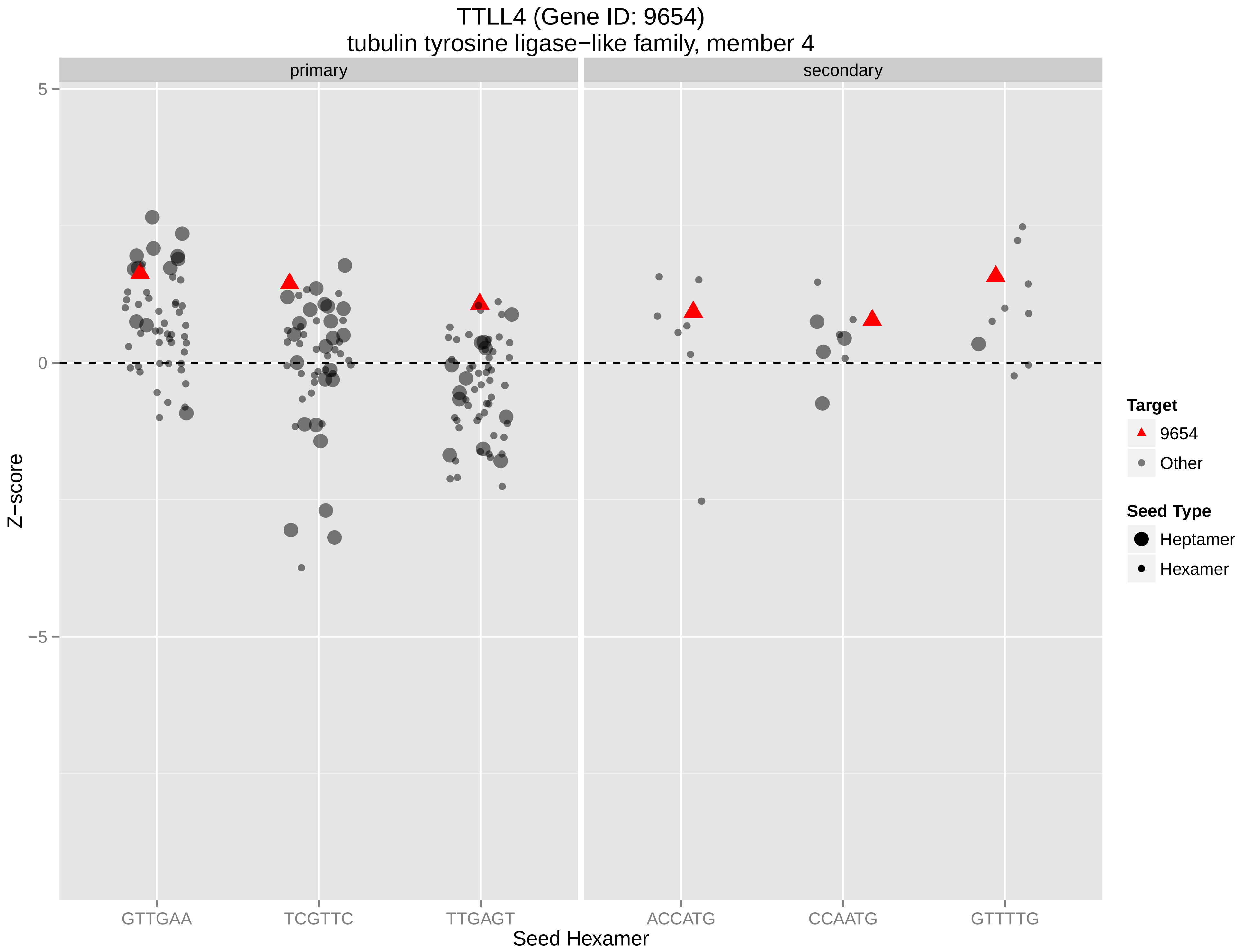

TTLL4 (Gene ID: 9654)
tubulin tyrosine ligase−like family, member 4
primary
secondary
5
0
−5
Z−score
GTTGAA
TCGTTC
TTGAGT
ACCATG
CCAATG
GTTTTG
Seed Hexamer
Target
9654
Other
Seed Type
Heptamer
Hexamer

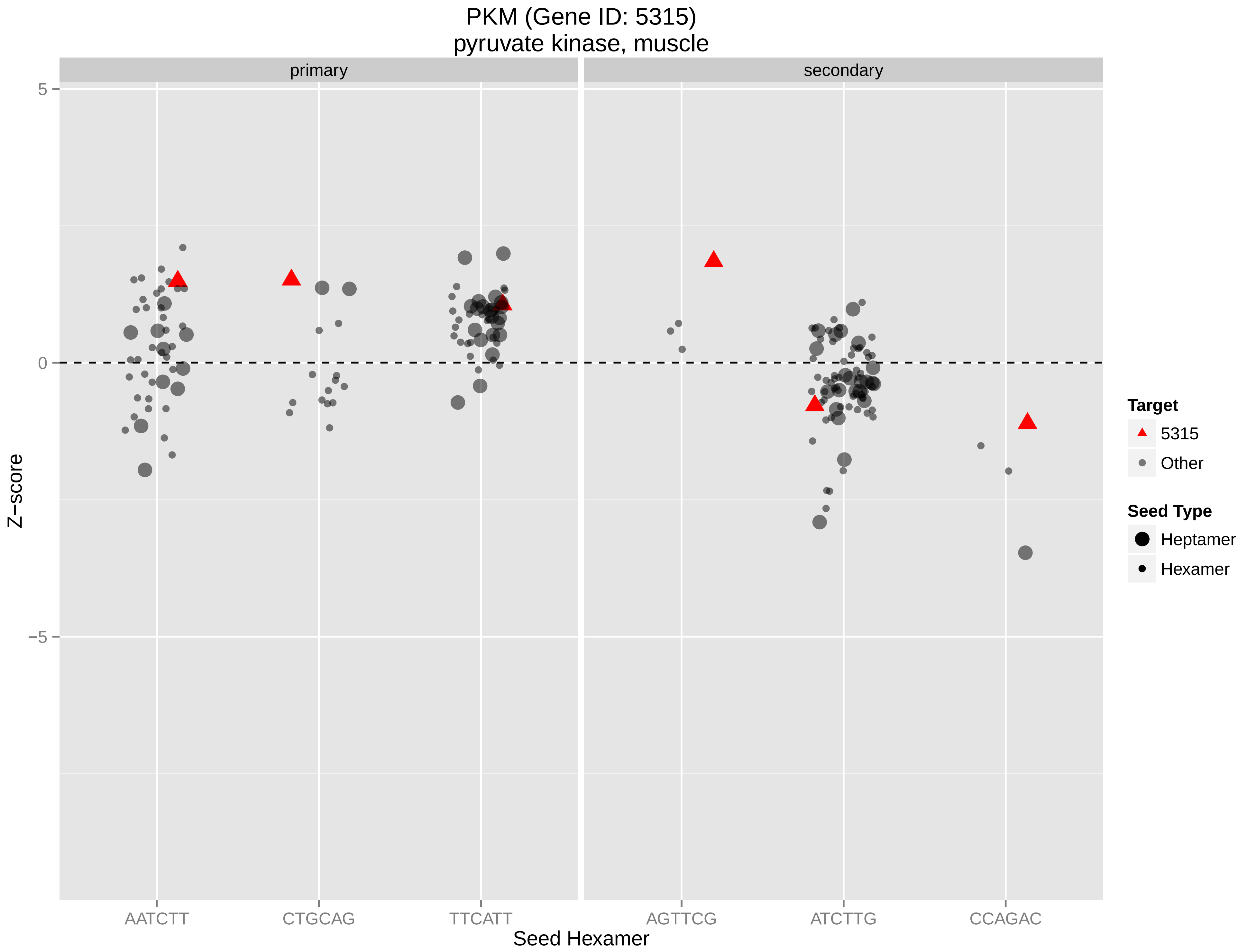

PKM (Gene ID: 5315)
pyruvate kinase, muscle
primary
secondary
5
0
−5
Z−score
AATCTT
CTGCAG
TTCATT
AGTTCG
ATCTTG
CCAGAC
Seed Hexamer
Target
5315
Other
Seed Type
Heptamer
Hexamer

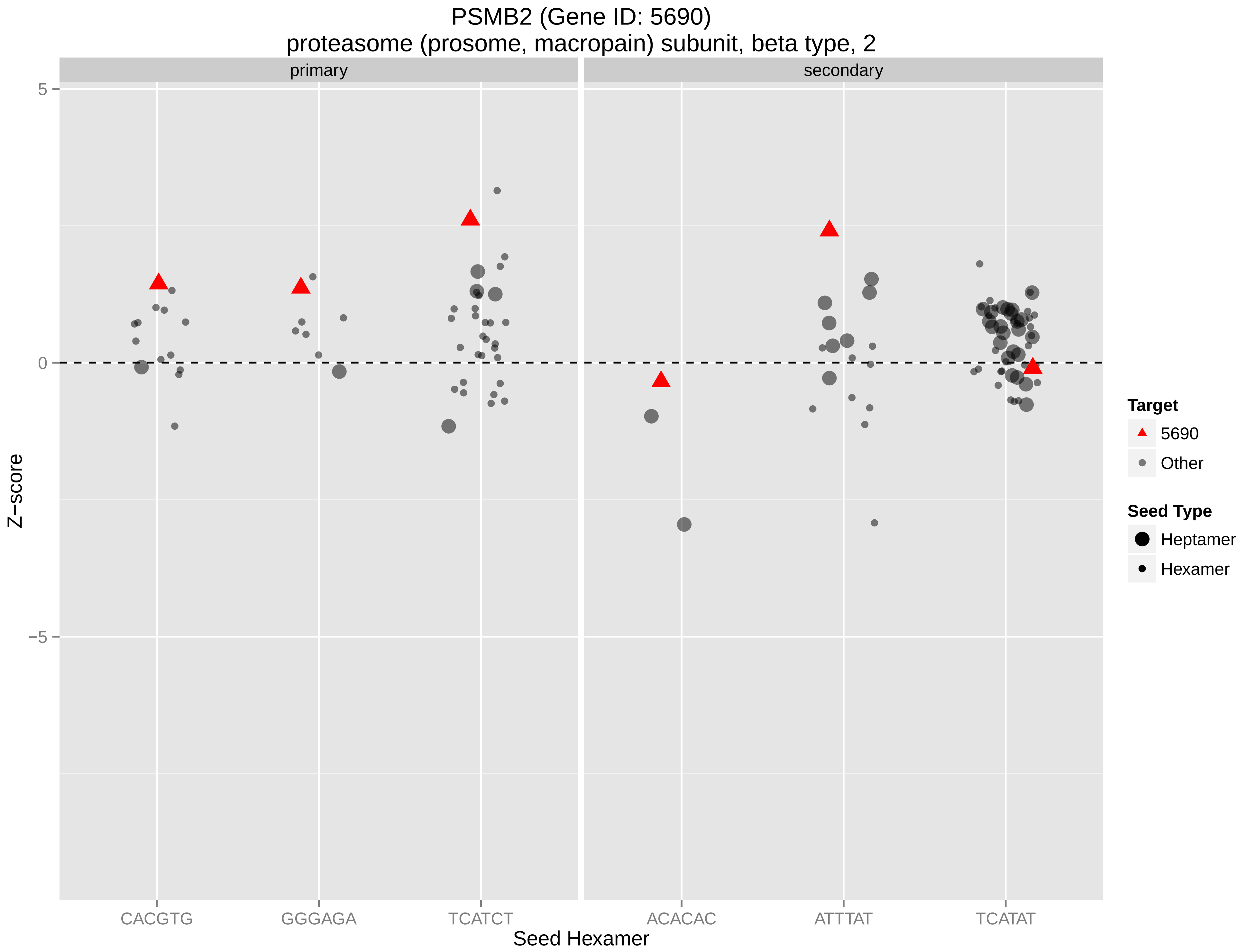

PSMB2 (Gene ID: 5690)
proteasome (prosome, macropain) subunit, beta type, 2
primary
secondary
5
0
−5
Z−score
CACGTG
GGGAGA
TCATCT
ACACAC
ATTTAT
TCATAT
Seed Hexamer
Target
5690
Other
Seed Type
Heptamer
Hexamer

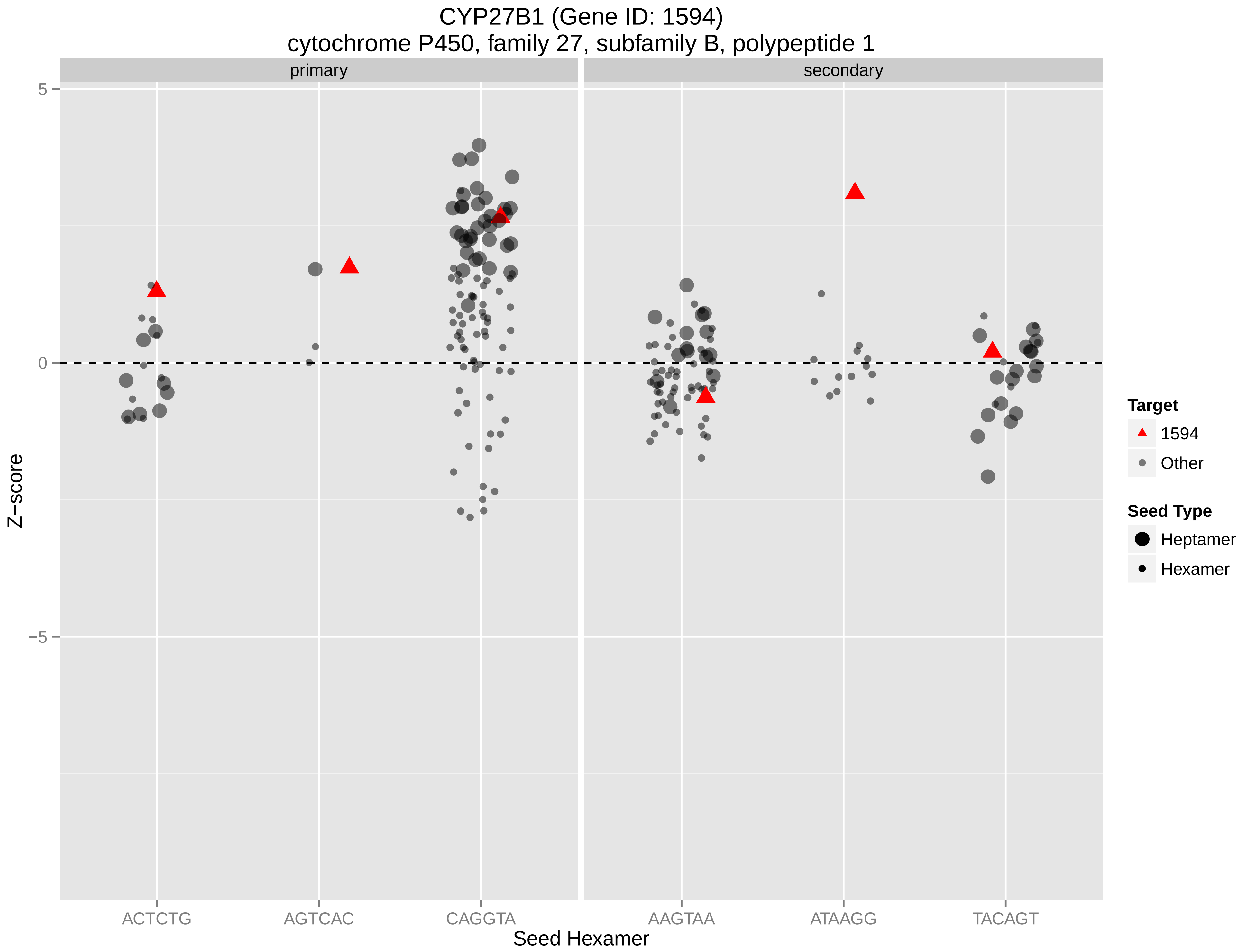
CYP27B1 (Gene ID: 1594)
cytochrome P450, family 27, subfamily B, polypeptide 1
primary
secondary
5
0
−5
Z−score
ACTCTG
AGTCAC
CAGGTA
AAGTAA
ATAAGG
TACAGT
Seed Hexamer
Target
1594
Other
Seed Type
Heptamer
Hexamer

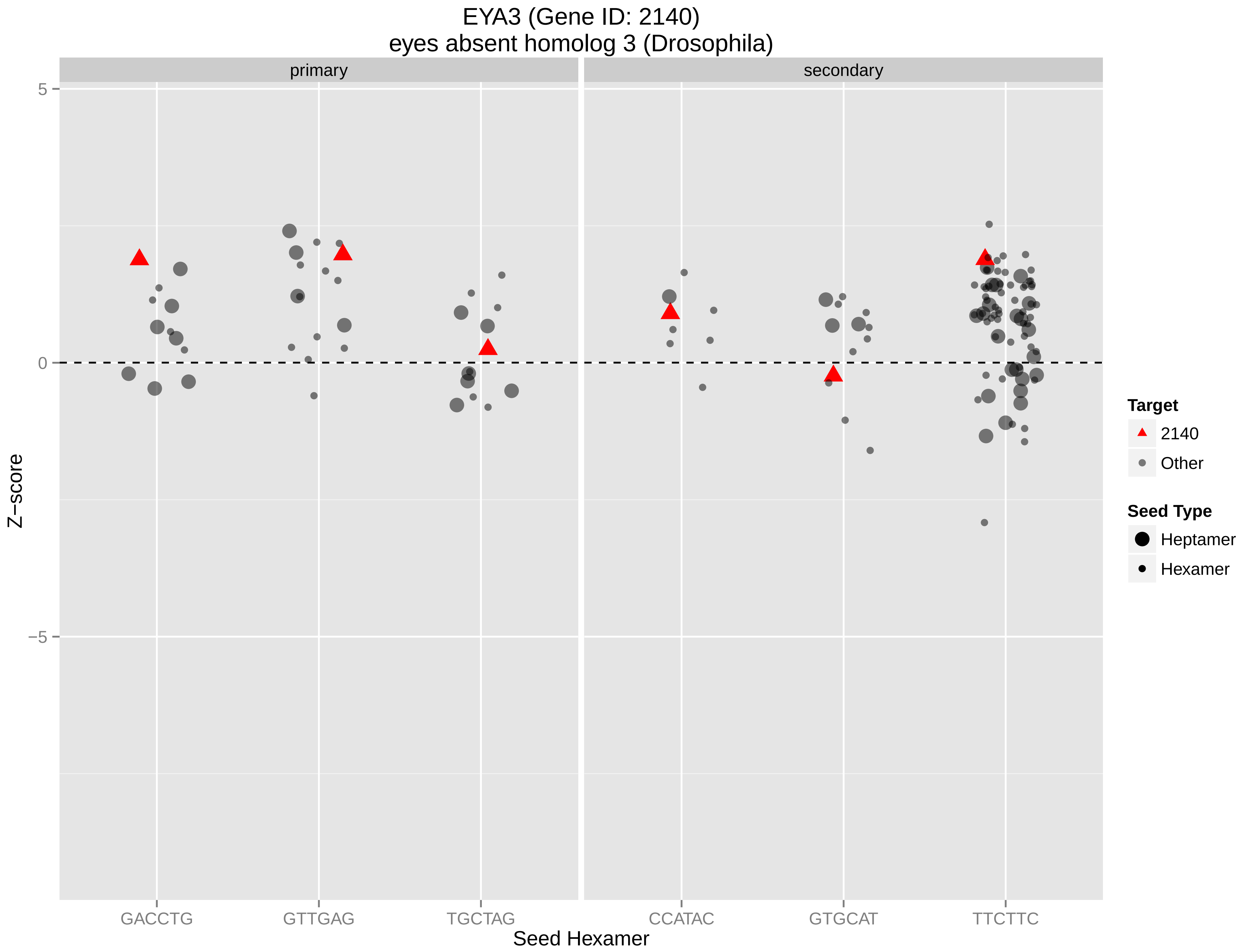
EYA3 (Gene ID: 2140)
eyes absent homolog 3 (Drosophila)
primary
secondary
5
0
−5
Z−score
GACCTG
GTTGAG
TGCTAG
CCATAC
GTGCAT
TTCTTC
Seed Hexamer
Target
2140
Other
Seed Type
Heptamer
Hexamer

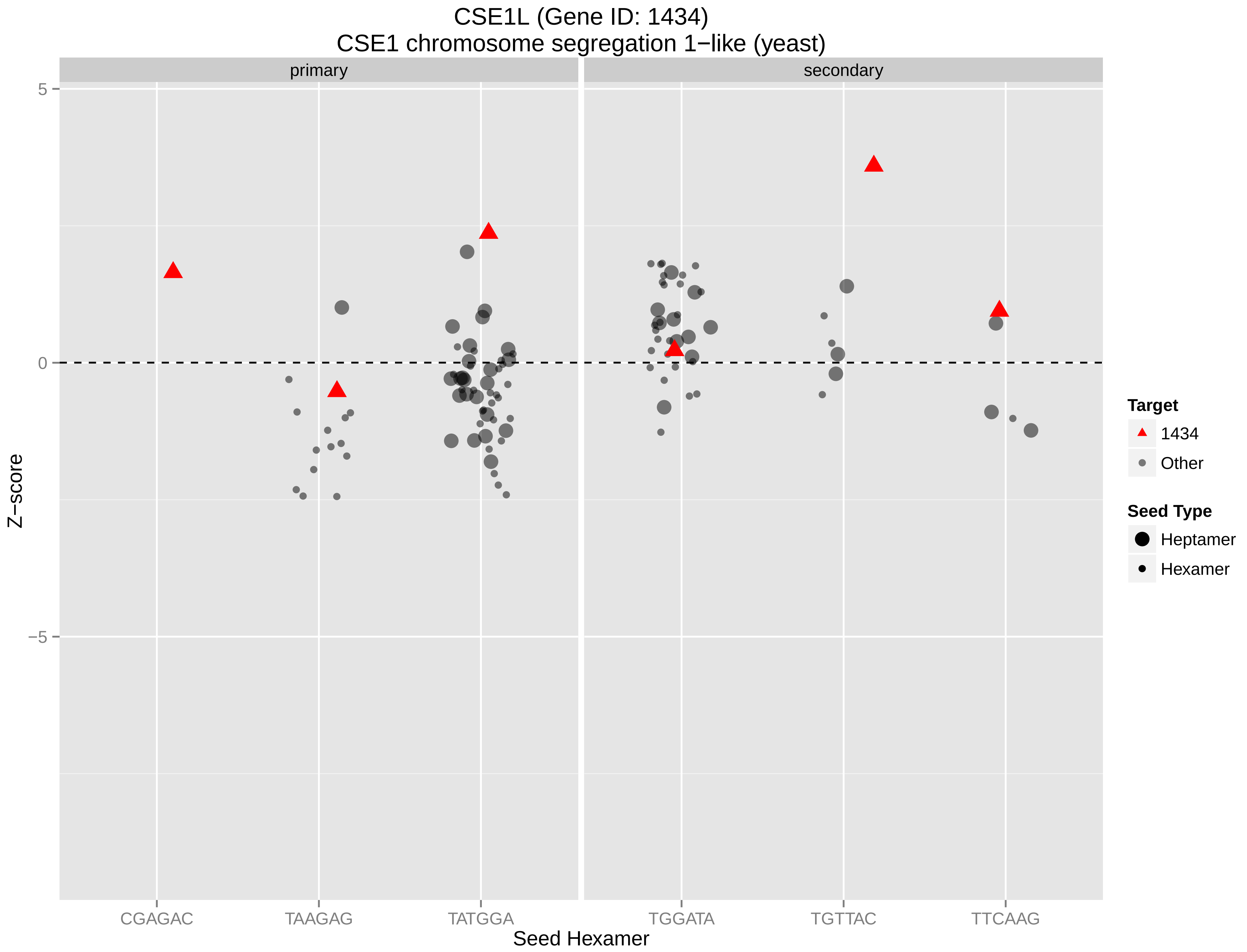

CSE1L (Gene ID: 1434)
CSE1 chromosome segregation 1−like (yeast)
primary
secondary
5
0
−5
Z−score
CGAGAC
TAAGAG
TATGGA
TGGATA
TGTTAC
TTCAAG
Seed Hexamer
Target
1434
Other
Seed Type
Heptamer
Hexamer

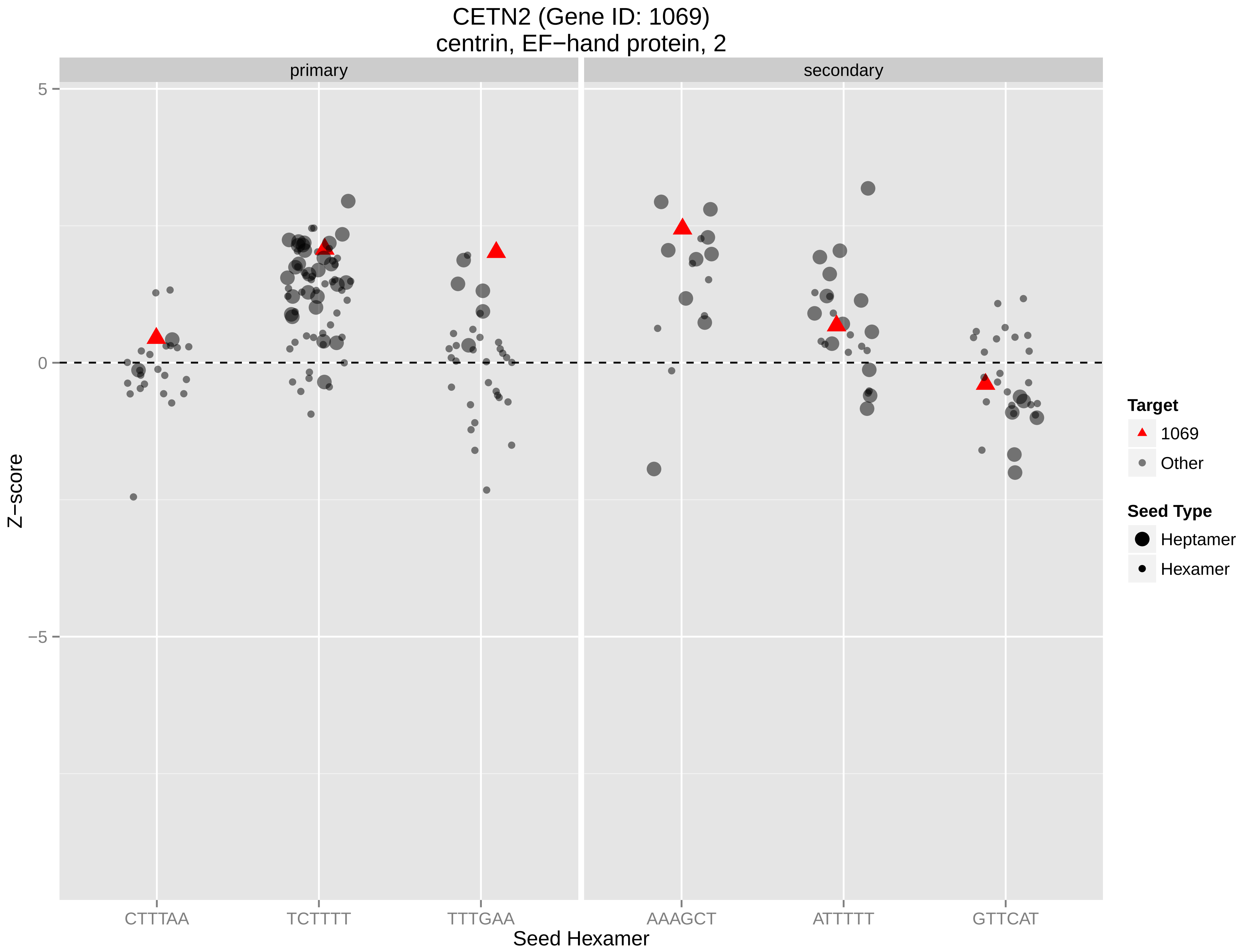
CETN2 (Gene ID: 1069)
centrin, EF−hand protein, 2
primary
secondary
5
0
−5
Z−score
CTTTAA
TCTTTT
TTTGAA
AAAGCT
ATTTTT
GTTCAT
Seed Hexamer
Target
1069
Other
Seed Type
Heptamer
Hexamer

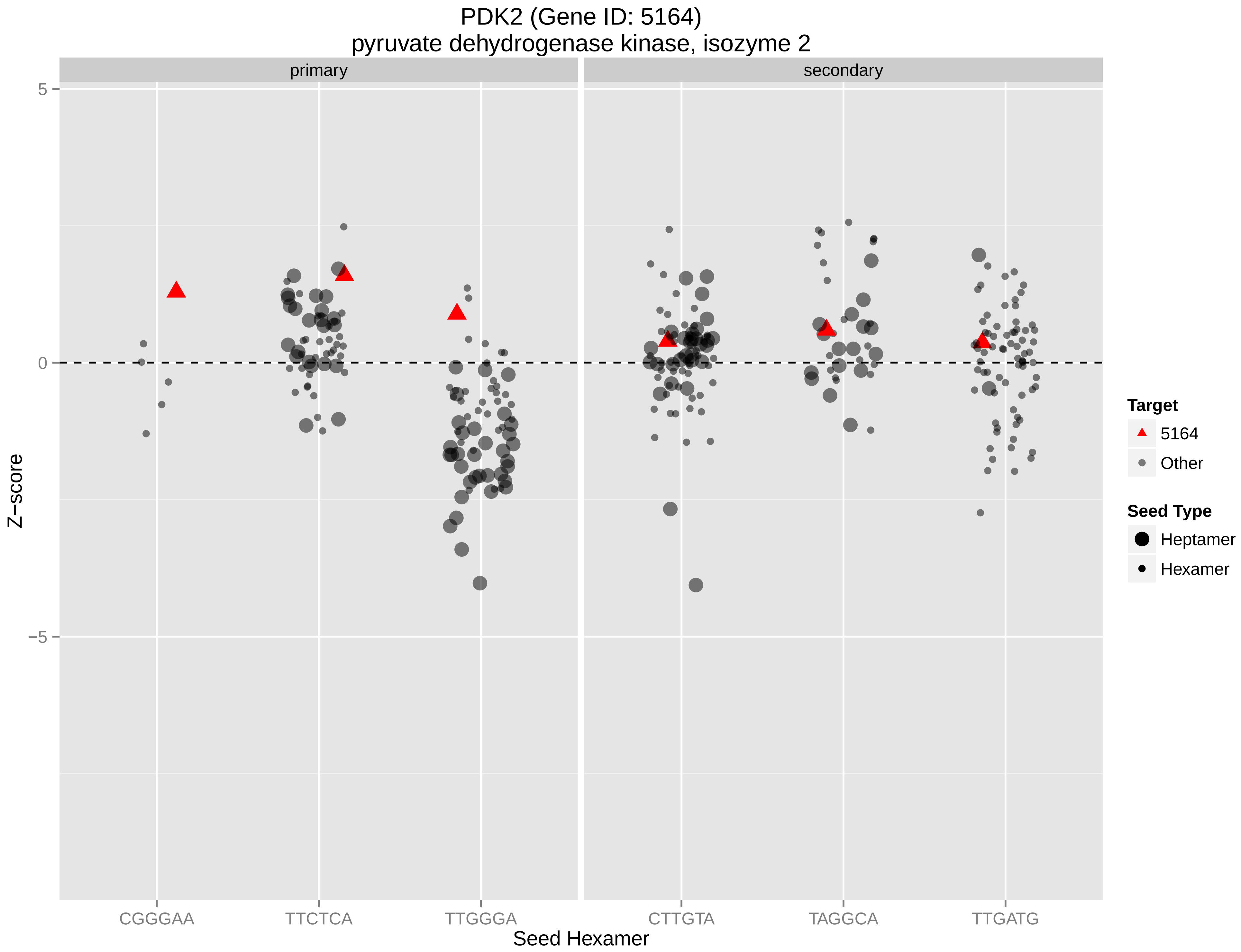
PDK2 (Gene ID: 5164)
pyruvate dehydrogenase kinase, isozyme 2
primary
secondary
5
0
−5
Z−score
CGGGAA
TTCTCA
TTGGGA
CTTGTA
TAGGCA
TTGATG
Seed Hexamer
Target
5164
Other
Seed Type
Heptamer
Hexamer

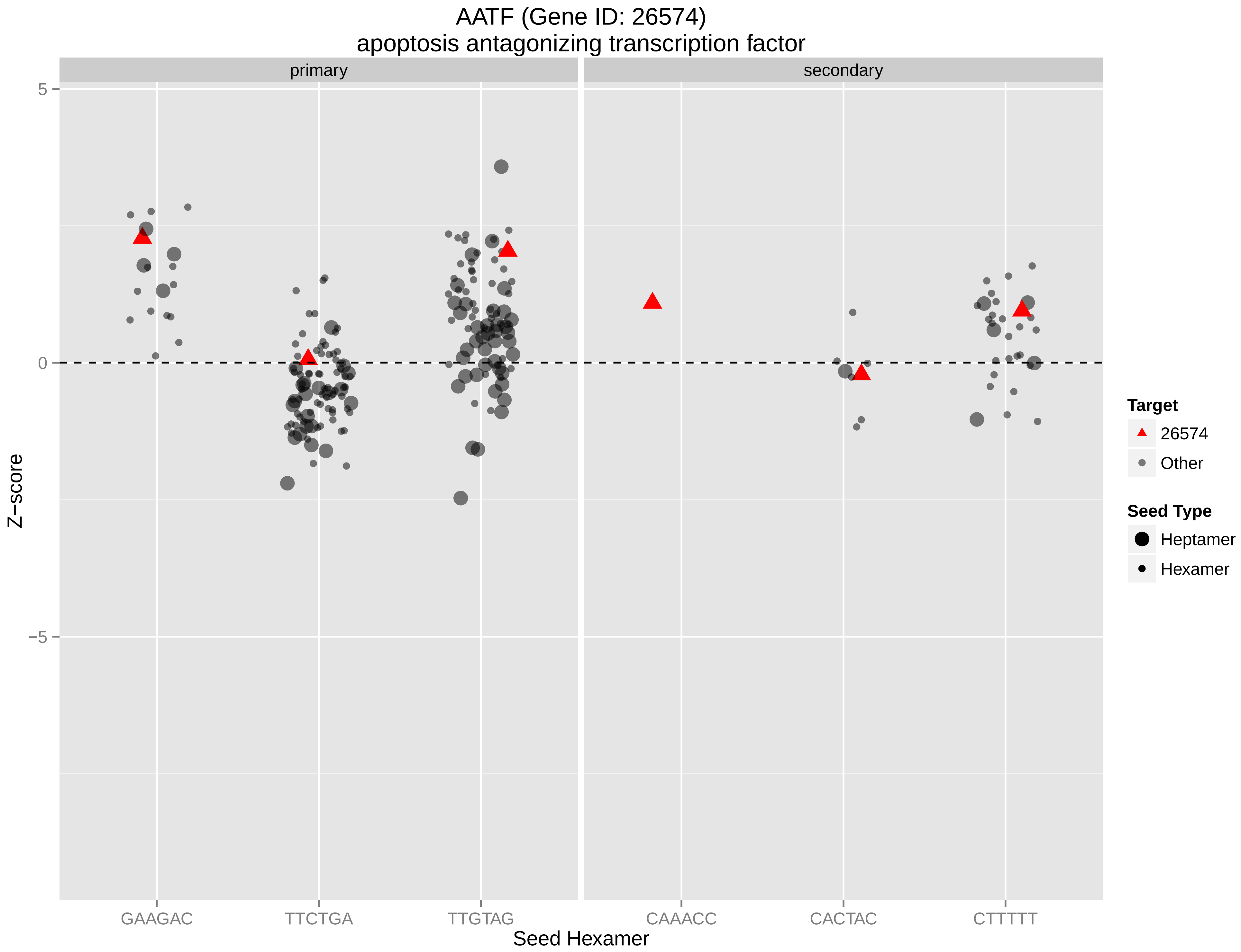

AATF (Gene ID: 26574)
apoptosis antagonizing transcription factor
primary
secondary
5
0
−5
Z−score
GAAGAC
TTCTGA
TTGTAG
CAAACC
CACTAC
CTTTTT
Seed Hexamer
Target
26574
Other
Seed Type
Heptamer
Hexamer

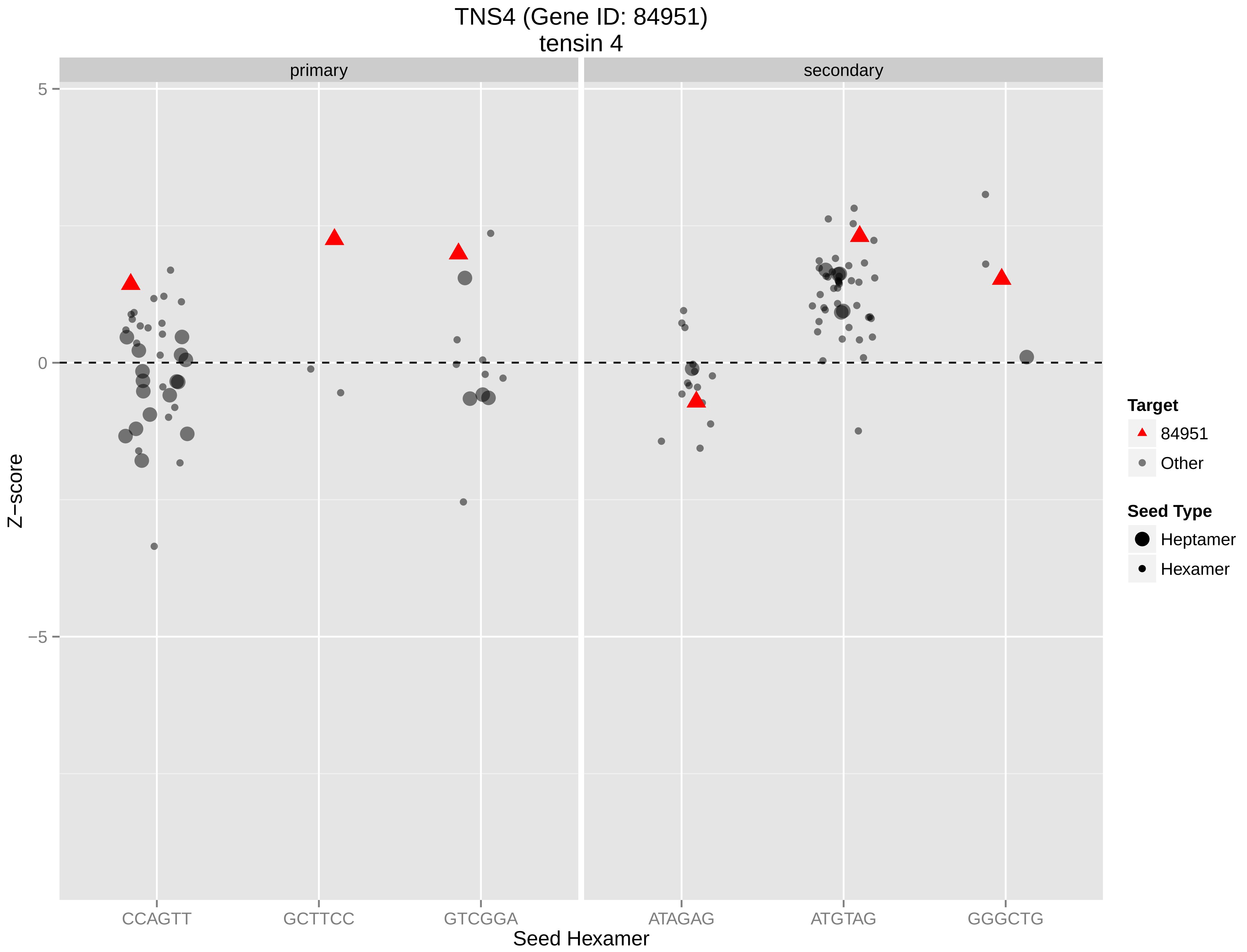
TNS4 (Gene ID: 84951)
tensin 4
primary
secondary
5
0
−5
Z−score
CCAGTT
GCTTCC
GTCGGA
ATAGAG
ATGTAG
GGGCTG
Seed Hexamer
Target
84951
Other
Seed Type
Heptamer
Hexamer

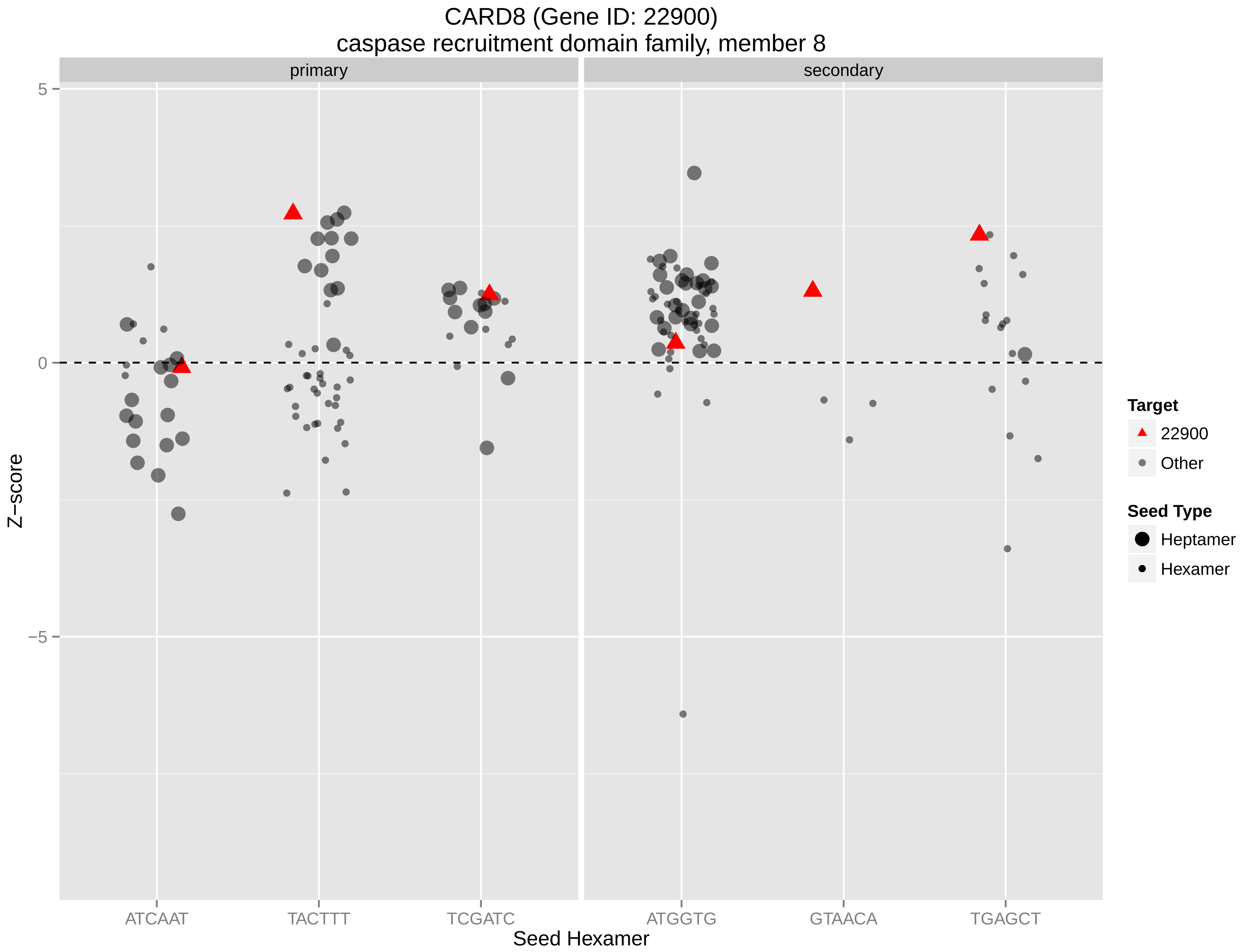

CARD8 (Gene ID: 22900)
caspase recruitment domain family, member 8
primary
secondary
5
0
−5
Z−score
ATCAAT
TACTTT
TCGATC
ATGGTG
GTAACA
TGAGCT
Seed Hexamer
Target
22900
Other
Seed Type
Heptamer
Hexamer

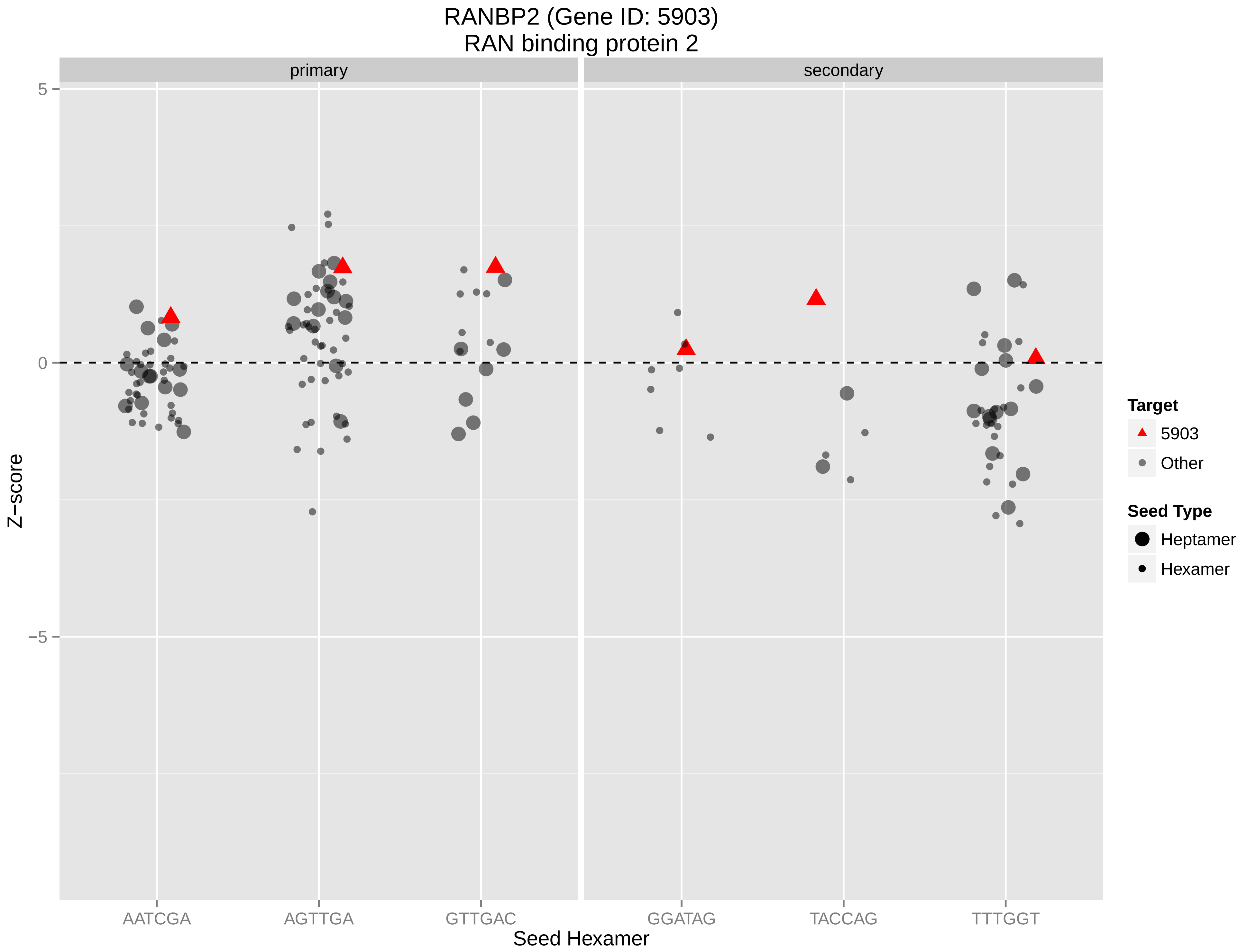
RANBP2 (Gene ID: 5903)
RAN binding protein 2
primary
secondary
5
0
−5
Z−score
AATCGA
AGTTGA
GTTGAC
GGATAG
TACCAG
TTTGGT
Seed Hexamer
Target
5903
Other
Seed Type
Heptamer
Hexamer

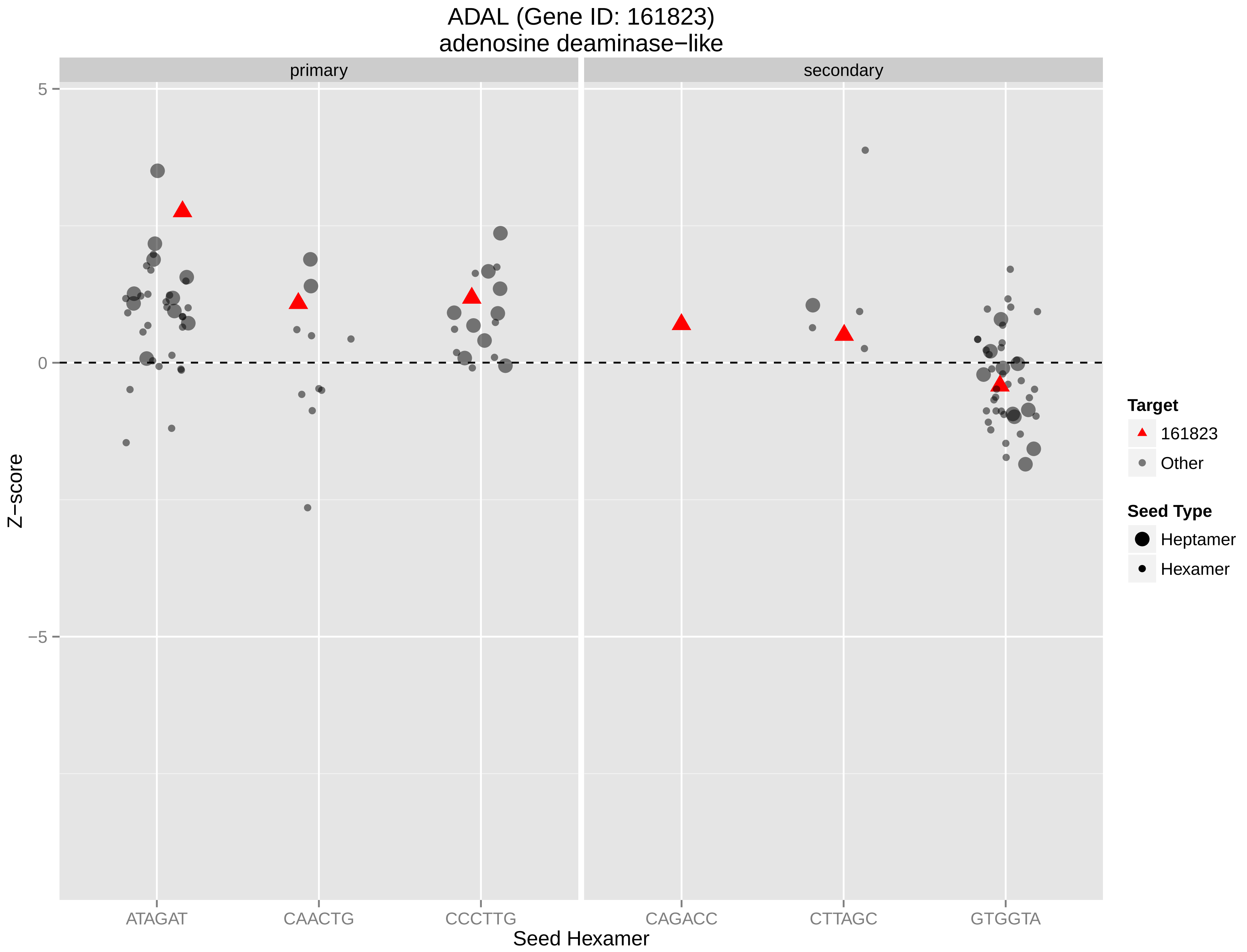

ADAL (Gene ID: 161823)
adenosine deaminase−like
primary
secondary
5
0
−5
Z−score
ATAGAT
CAACTG
CCCTTG
CAGACC
CTTAGC
GTGGTA
Seed Hexamer
Target
161823
Other
Seed Type
Heptamer
Hexamer

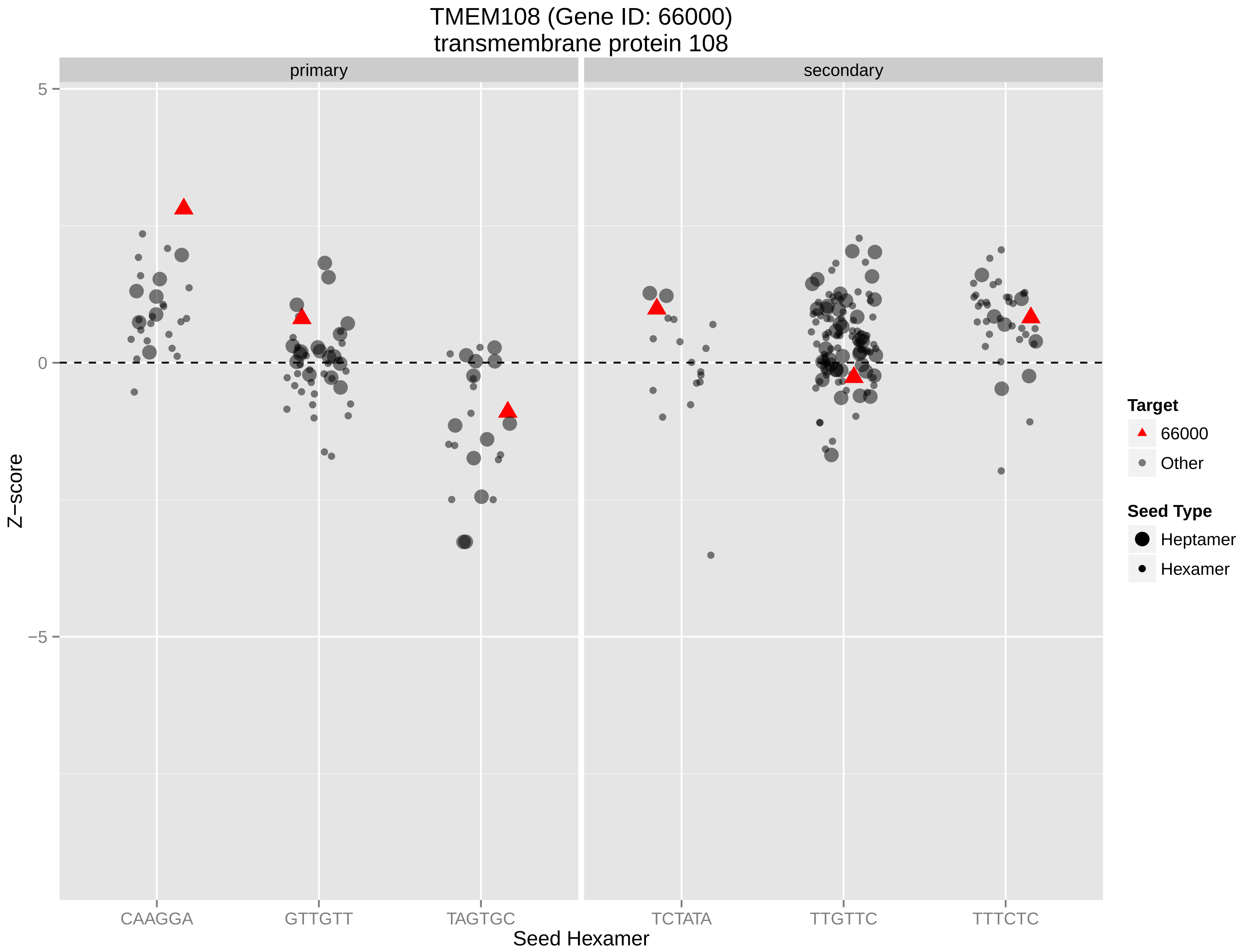
TMEM108 (Gene ID: 66000)
transmembrane protein 108
primary
secondary
5
0
−5
Z−score
CAAGGA
GTTGTT
TAGTGC
TCTATA
TTGTTC
TTTCTC
Seed Hexamer
Target
66000
Other
Seed Type
Heptamer
Hexamer

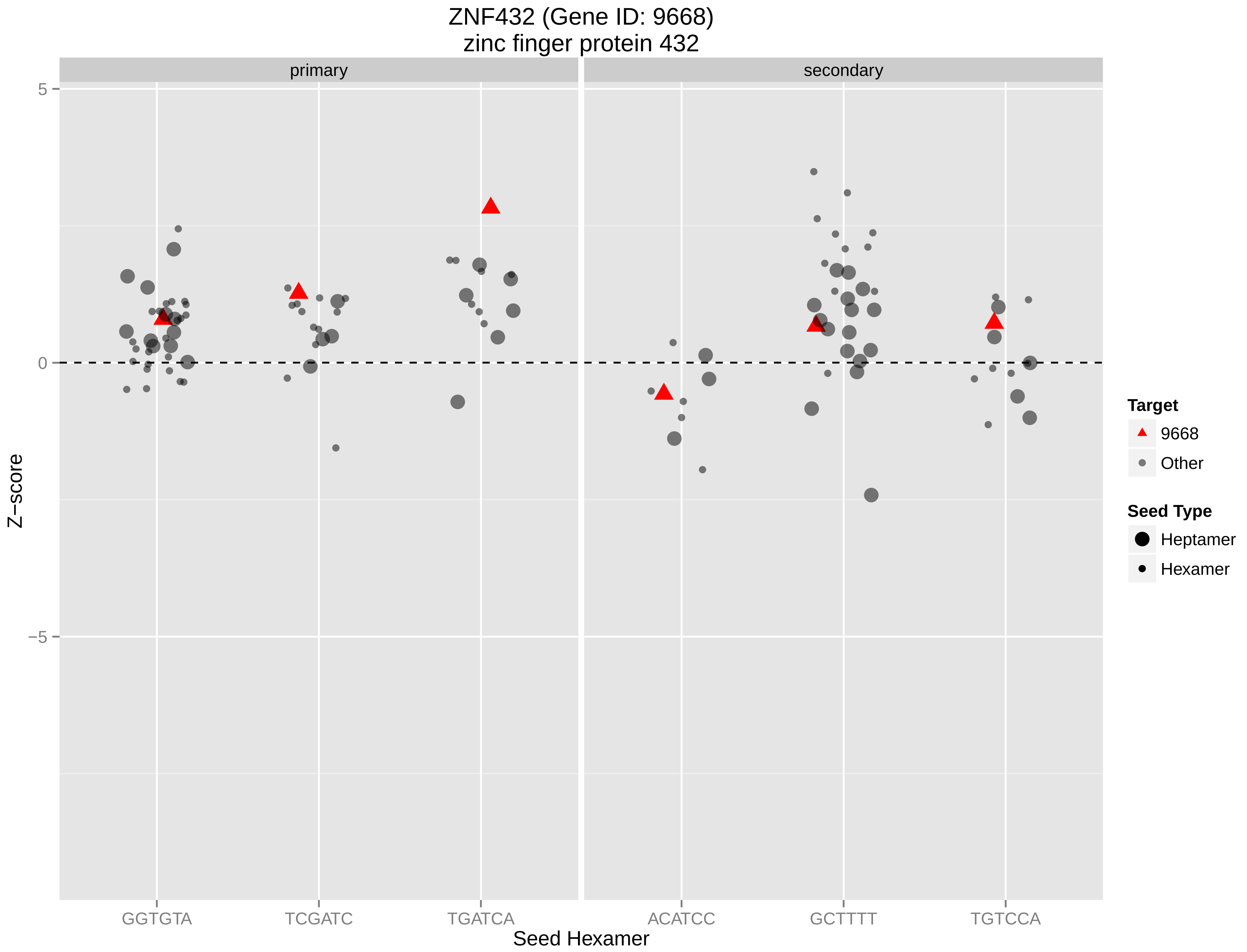
ZNF432 (Gene ID: 9668)
zinc finger protein 432
primary
secondary
5
0
−5
Z−score
GGTGTA
TCGATC
TGATCA
ACATCC
GCTTTT
TGTCCA
Seed Hexamer
Target
9668
Other
Seed Type
Heptamer
Hexamer

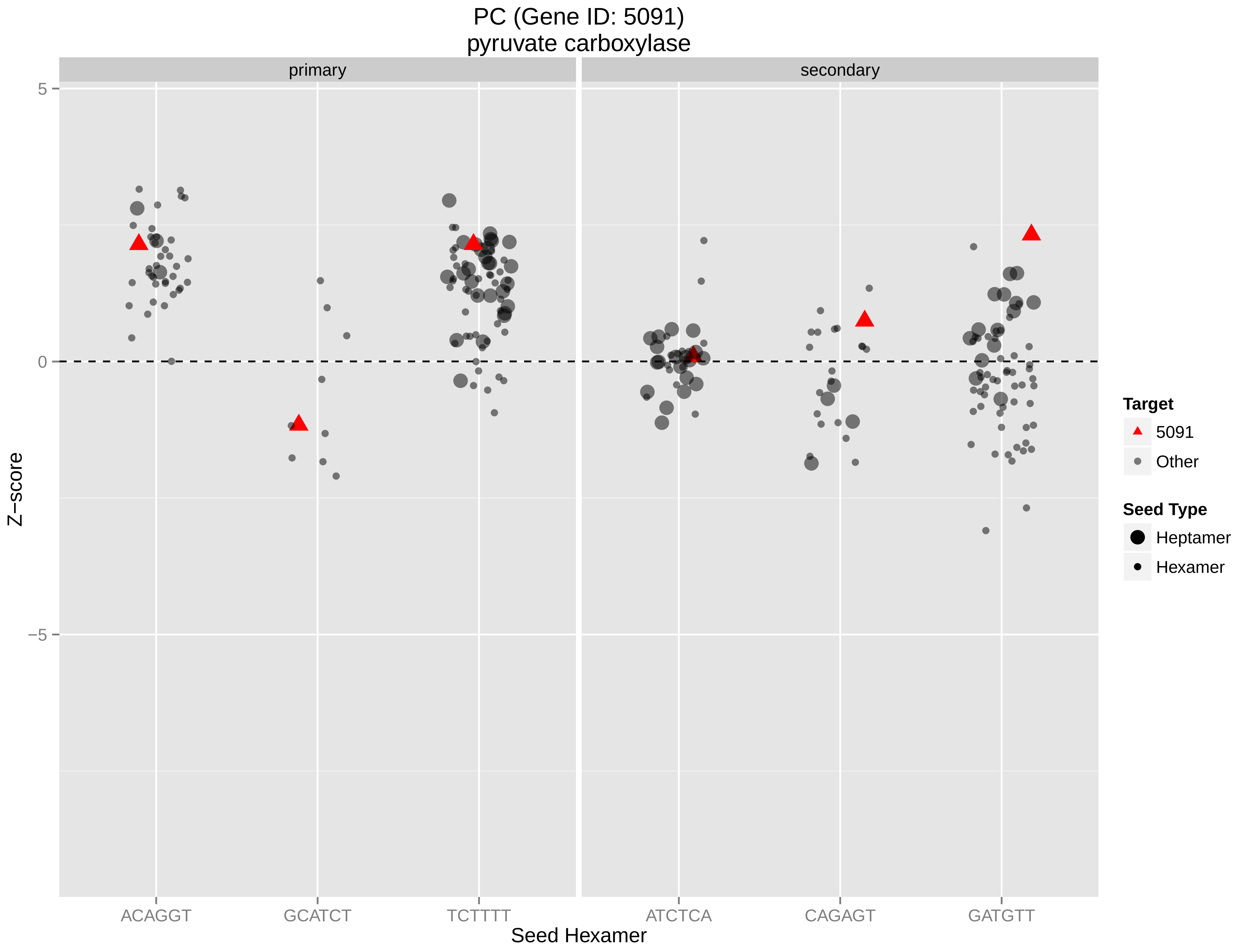
PC (Gene ID: 5091)
pyruvate carboxylase
primary
secondary
5
0
−5
Z−score
ACAGGT
GCATCT
TCTTTT
ATCTCA
CAGAGT
GATGTT
Seed Hexamer
Target
5091
Other
Seed Type
Heptamer
Hexamer

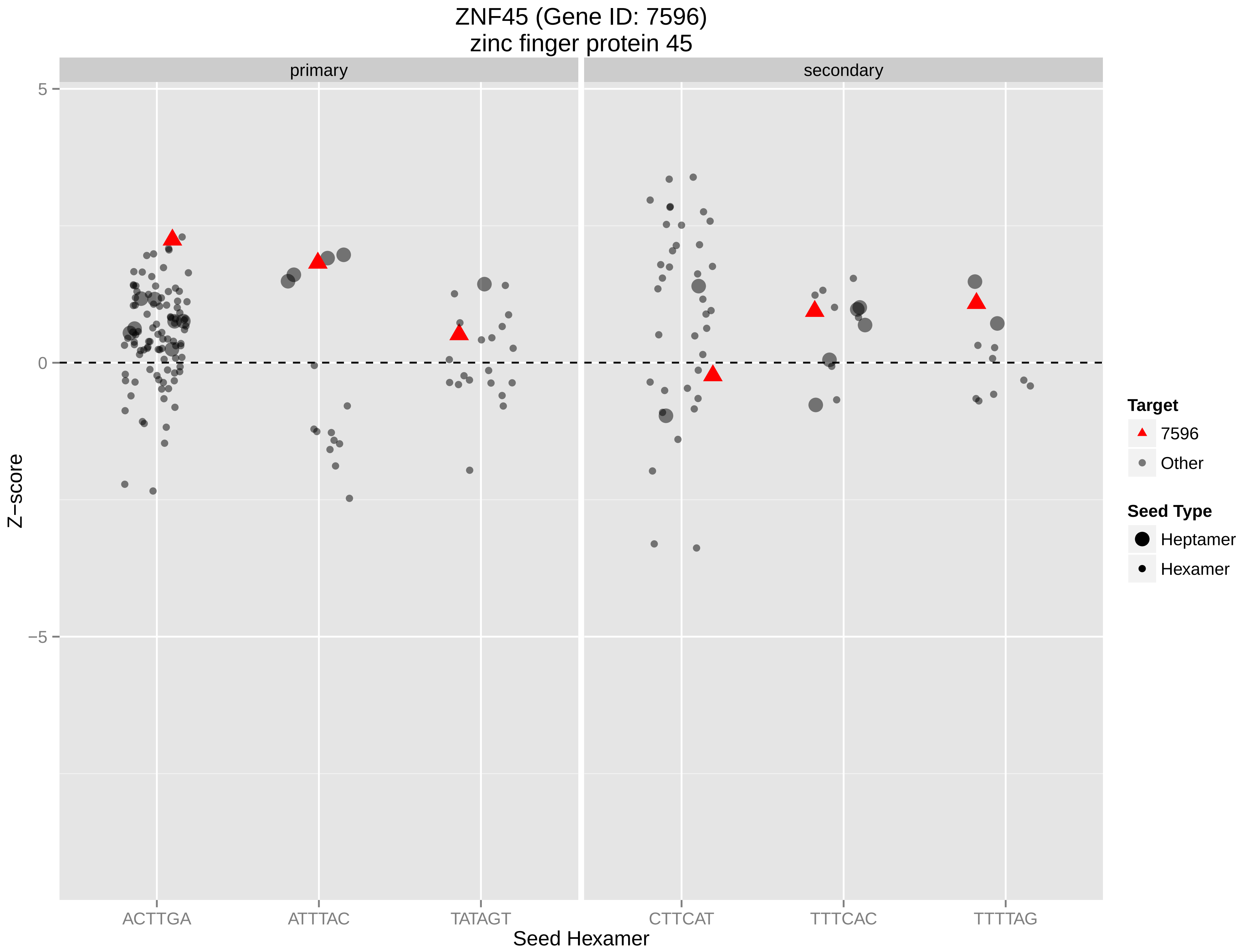
ZNF45 (Gene ID: 7596)
zinc finger protein 45
primary
secondary
5
0
−5
Z−score
ACTTGA
ATTTAC
TATAGT
CTTCAT
TTTCAC
TTTTAG
Seed Hexamer
Target
7596
Other
Seed Type
Heptamer
Hexamer

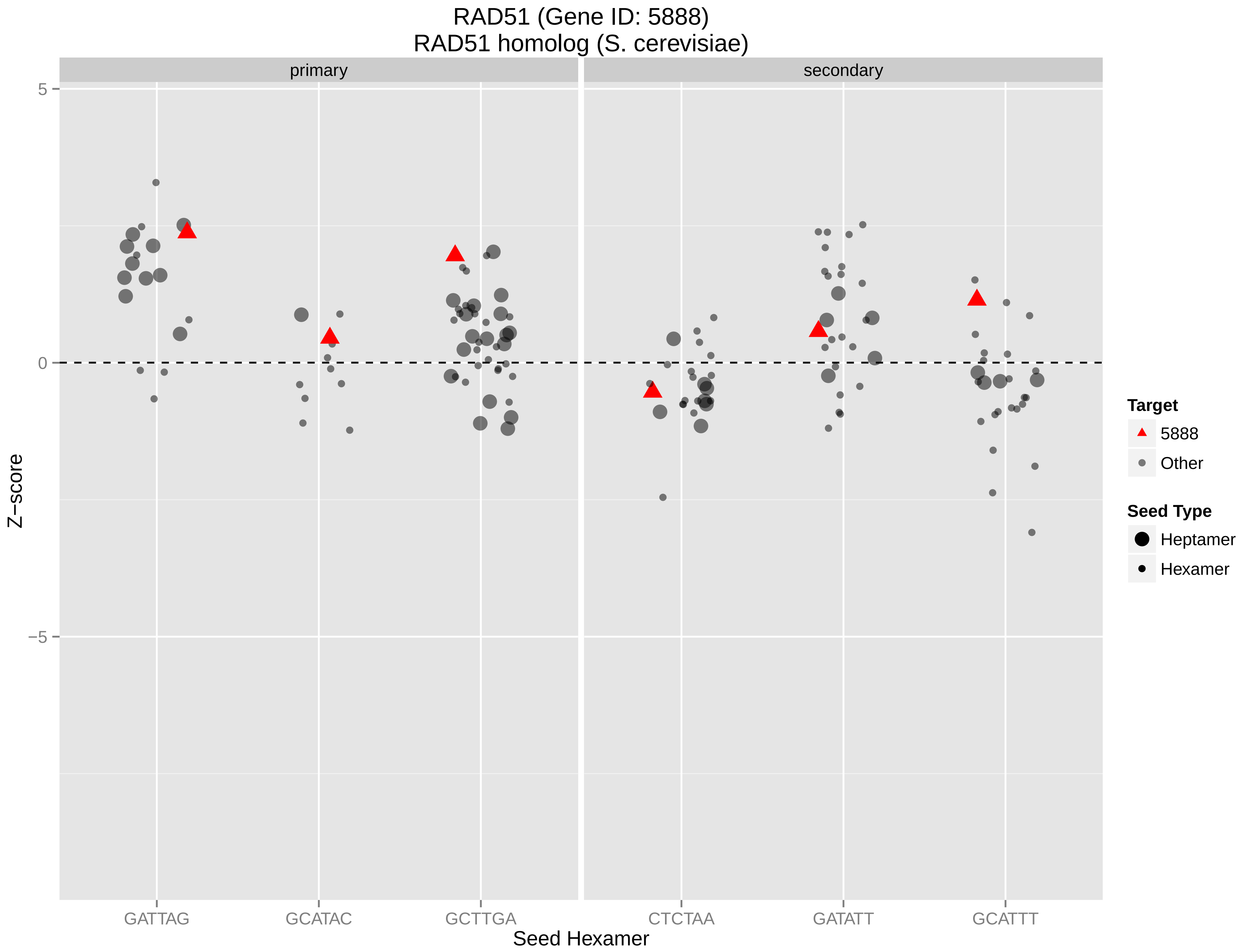

RAD51 (Gene ID: 5888)
RAD51 homolog (S. cerevisiae)
primary
secondary
5
0
−5
Z−score
GATTAG
GCATAC
GCTTGA
CTCTAA
GATATT
GCATTT
Seed Hexamer
Target
5888
Other
Seed Type
Heptamer
Hexamer

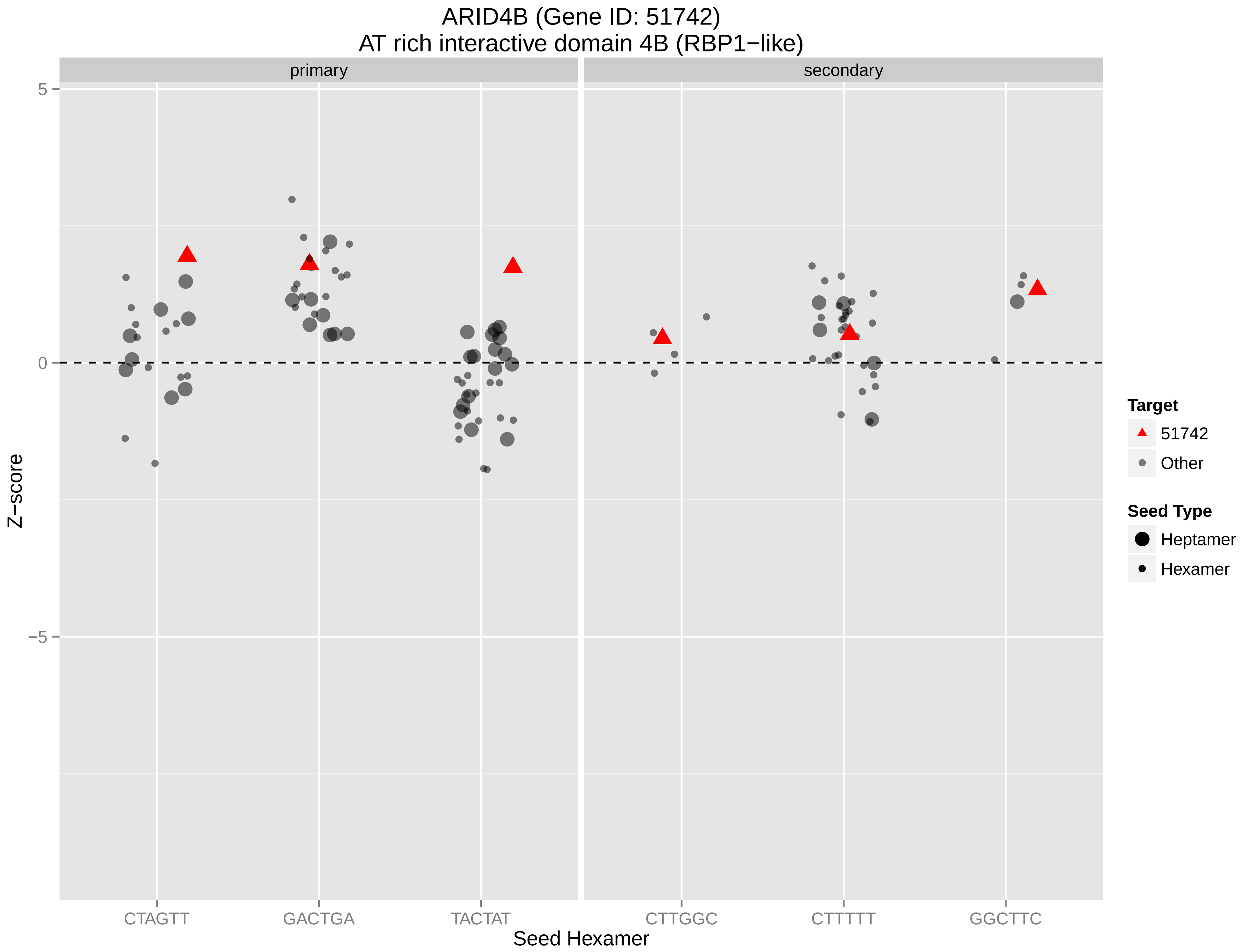

ARID4B (Gene ID: 51742)
AT rich interactive domain 4B (RBP1−like)
primary
secondary
5
0
−5
Z−score
CTAGTT
GACTGA
TACTAT
CTTGGC
CTTTTT
GGCTTC
Seed Hexamer
Target
51742
Other
Seed Type
Heptamer
Hexamer

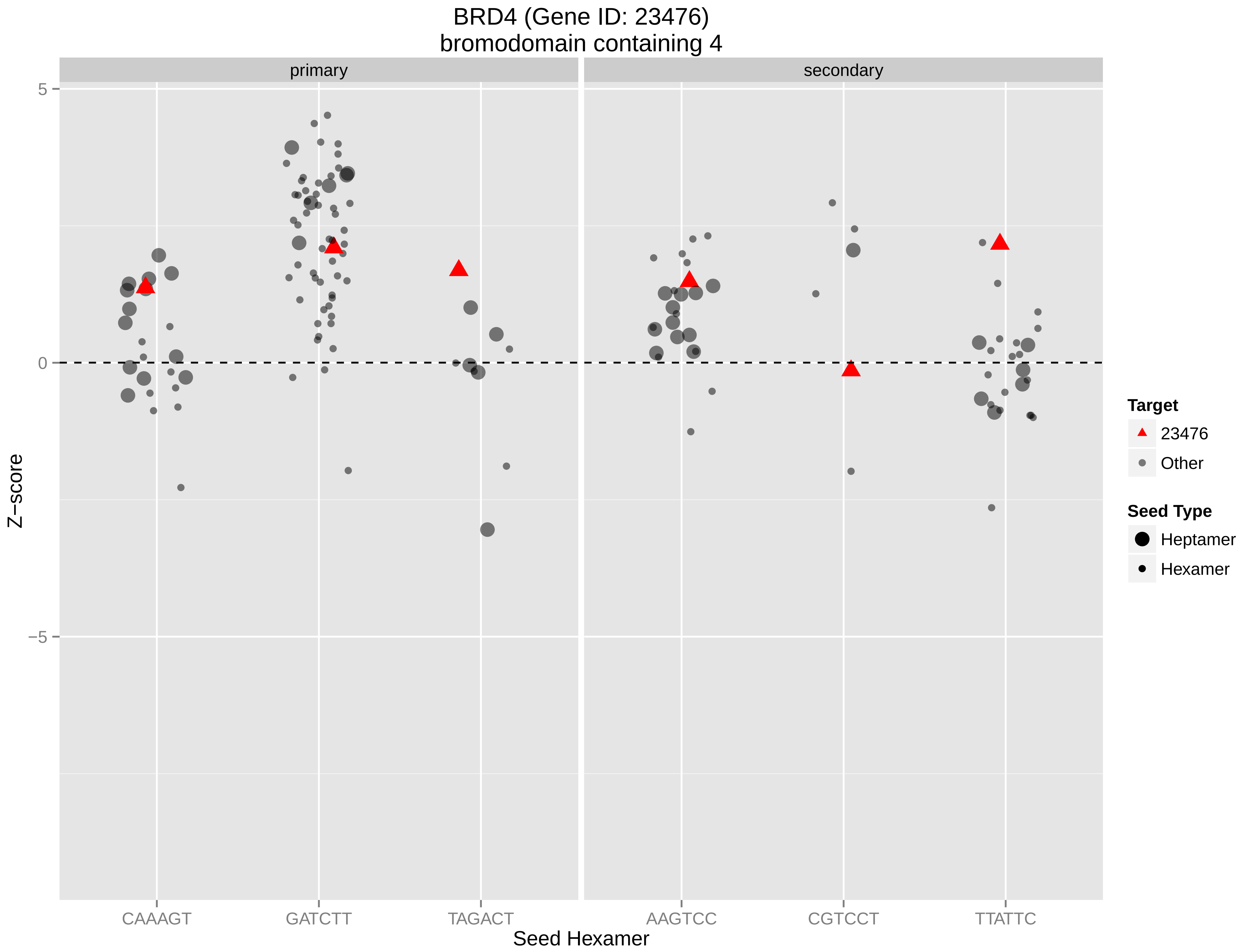
BRD4 (Gene ID: 23476)
bromodomain containing 4
primary
secondary
5
0
−5
Z−score
CAAAGT
GATCTT
TAGACT
AAGTCC
CGTCCT
TTATTC
Seed Hexamer
Target
23476
Other
Seed Type
Heptamer
Hexamer

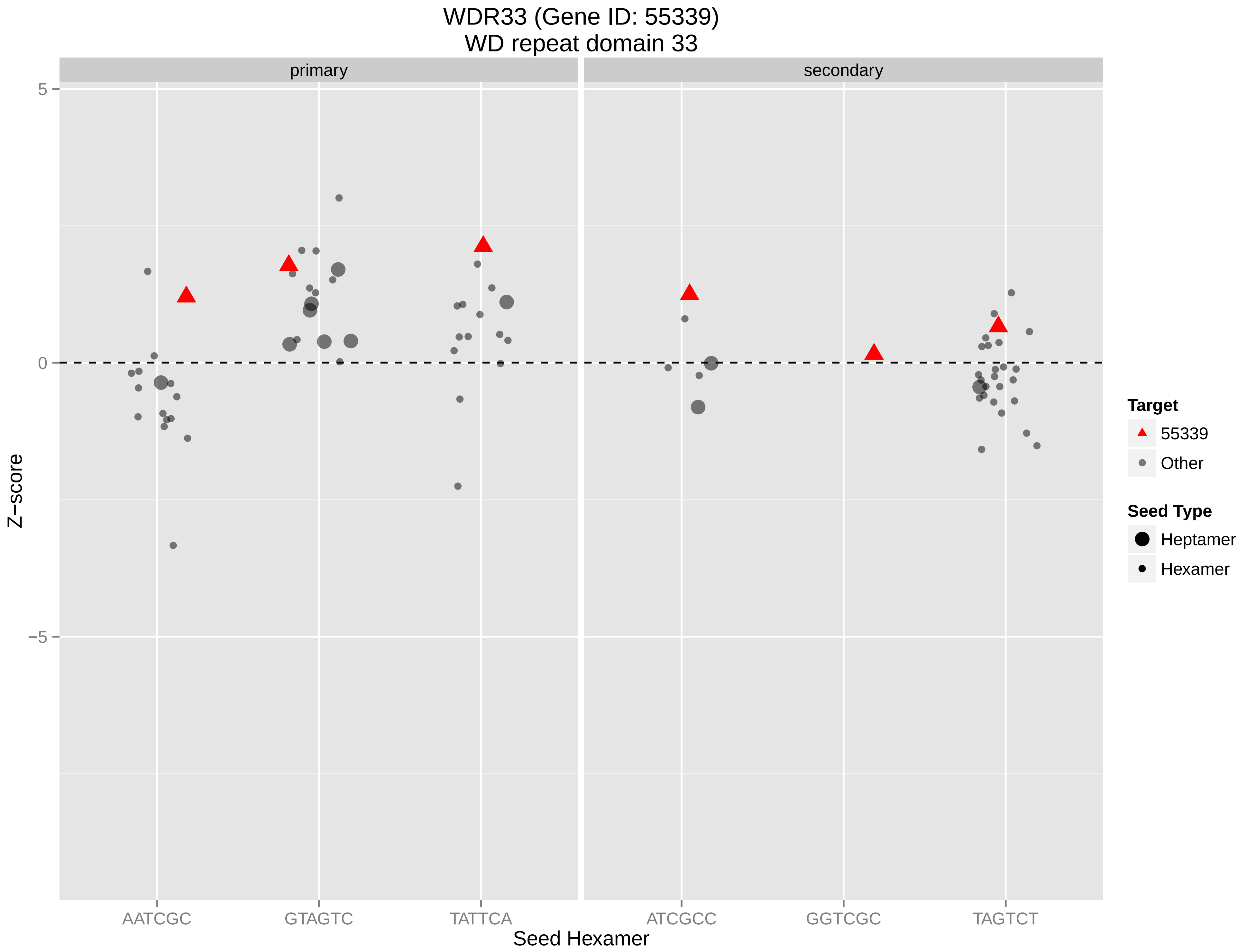
WDR33 (Gene ID: 55339)
WD repeat domain 33
primary
secondary
5
0
−5
Z−score
AATCGC
GTAGTC
TATTCA
ATCGCC
GGTCGC
TAGTCT
Seed Hexamer
Target
55339
Other
Seed Type
Heptamer
Hexamer

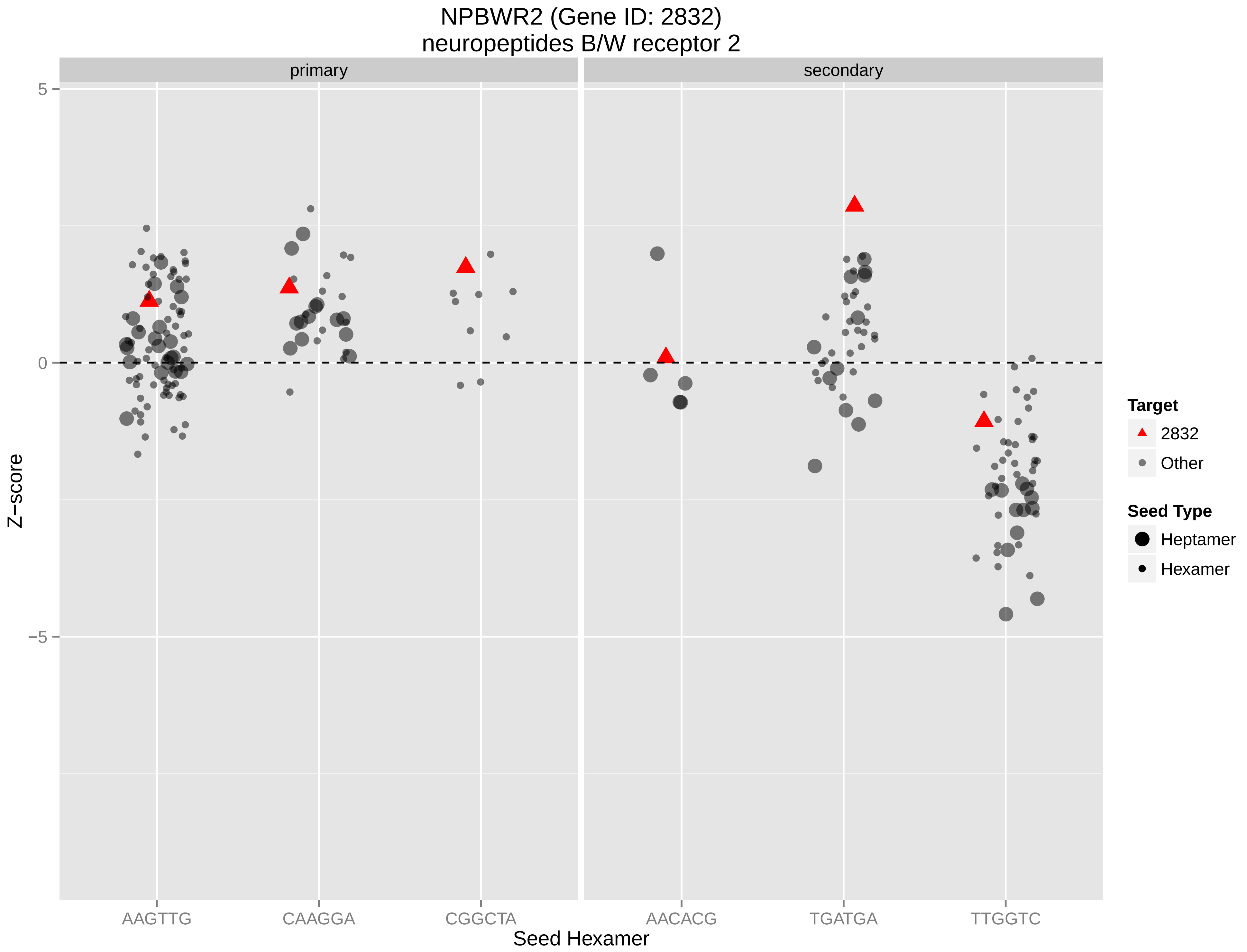
NPBWR2 (Gene ID: 2832)
neuropeptides B/W receptor 2
primary
secondary
5
0
−5
Z−score
AAGTTG
CAAGGA
CGGCTA
AACACG
TGATGA
TTGGTC
Seed Hexamer
Target
2832
Other
Seed Type
Heptamer
Hexamer

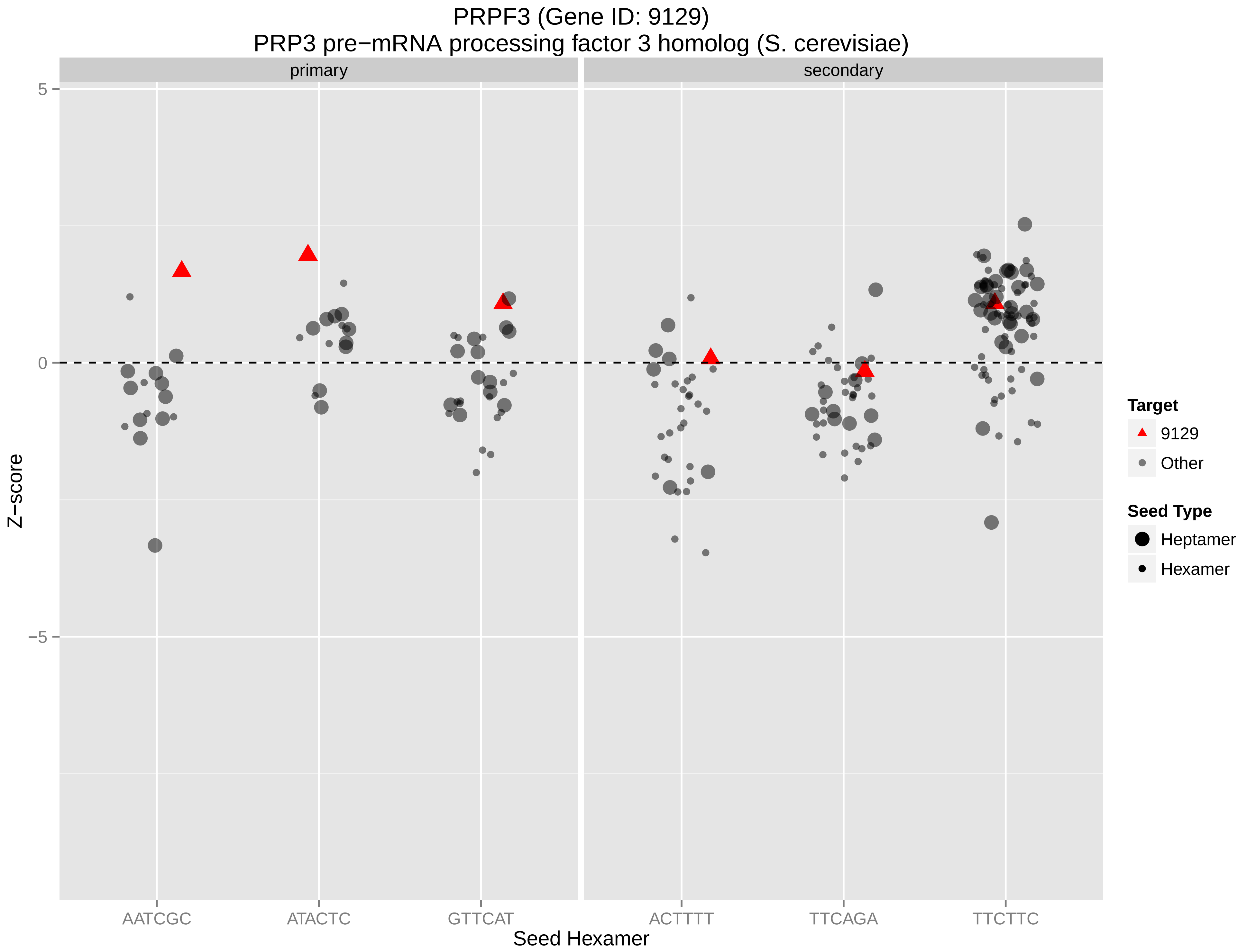
PRPF3 (Gene ID: 9129)
PRP3 pre−mRNA processing factor 3 homolog (S. cerevisiae)
primary
secondary
5
0
−5
Z−score
AATCGC
ATACTC
GTTCAT
ACTTTT
TTCAGA
TTCTTC
Seed Hexamer
Target
9129
Other
Seed Type
Heptamer
Hexamer

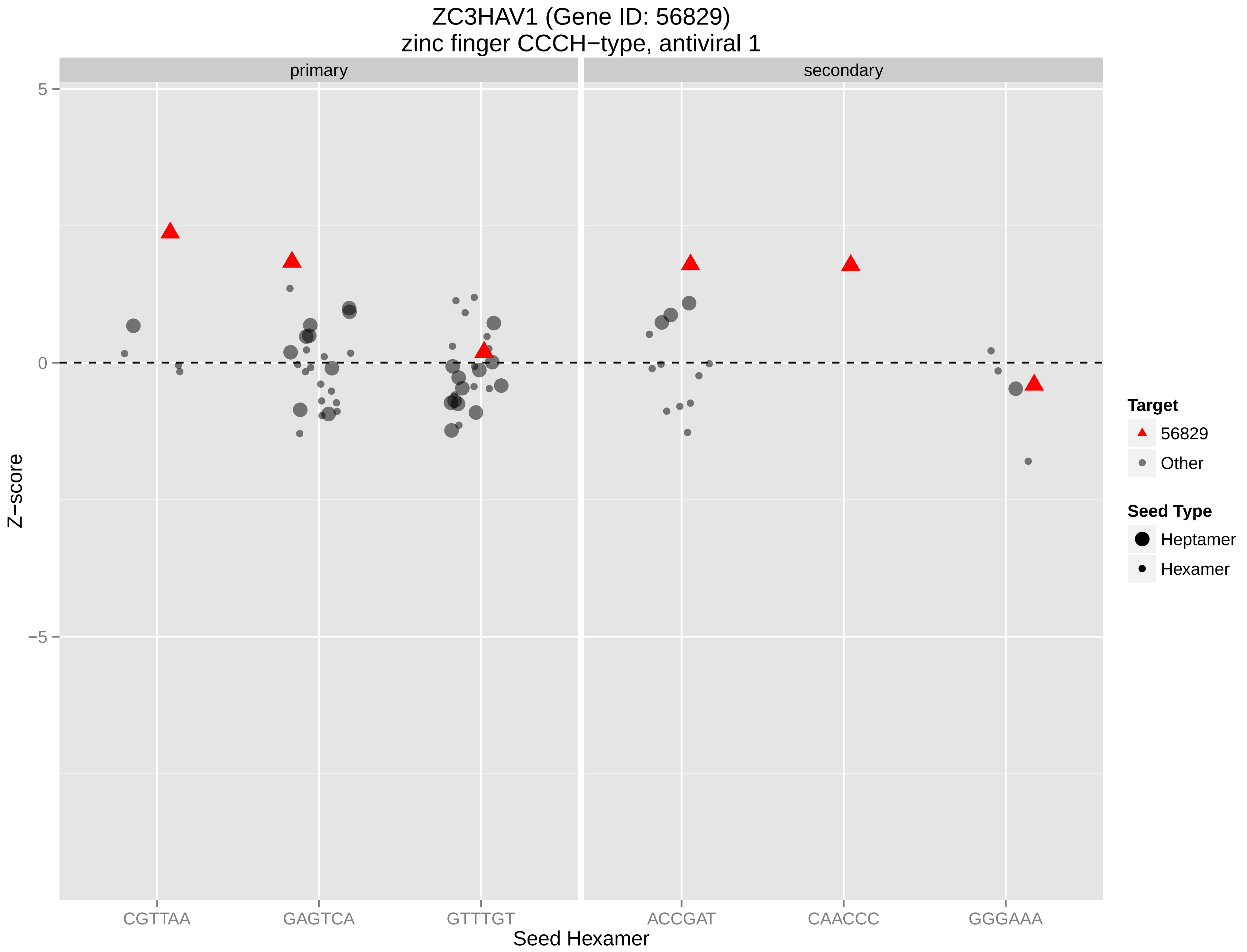

ZC3HAV1 (Gene ID: 56829)
zinc finger CCCH−type, antiviral 1
primary
secondary
5
0
−5
Z−score
CGTTAA
GAGTCA
GTTTGT
ACCGAT
CAACCC
GGGAAA
Seed Hexamer
Target
56829
Other
Seed Type
Heptamer
Hexamer

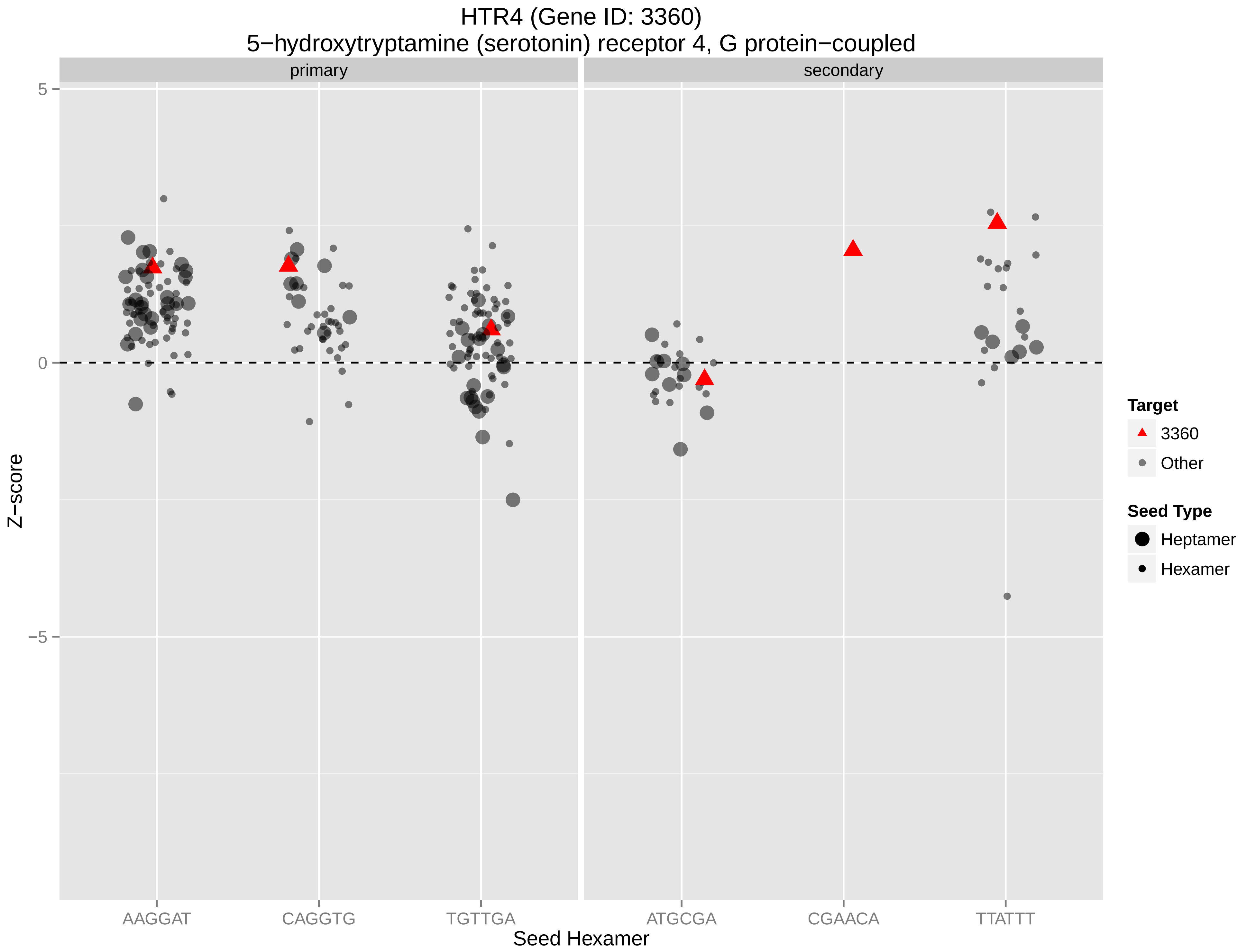
HTR4 (Gene ID: 3360)
5−hydroxytryptamine (serotonin) receptor 4, G protein−coupled
primary
secondary
5
0
−5
Z−score
AAGGAT
CAGGTG
TGTTGA
ATGCGA
CGAACA
TTATTT
Seed Hexamer
Target
3360
Other
Seed Type
Heptamer
Hexamer

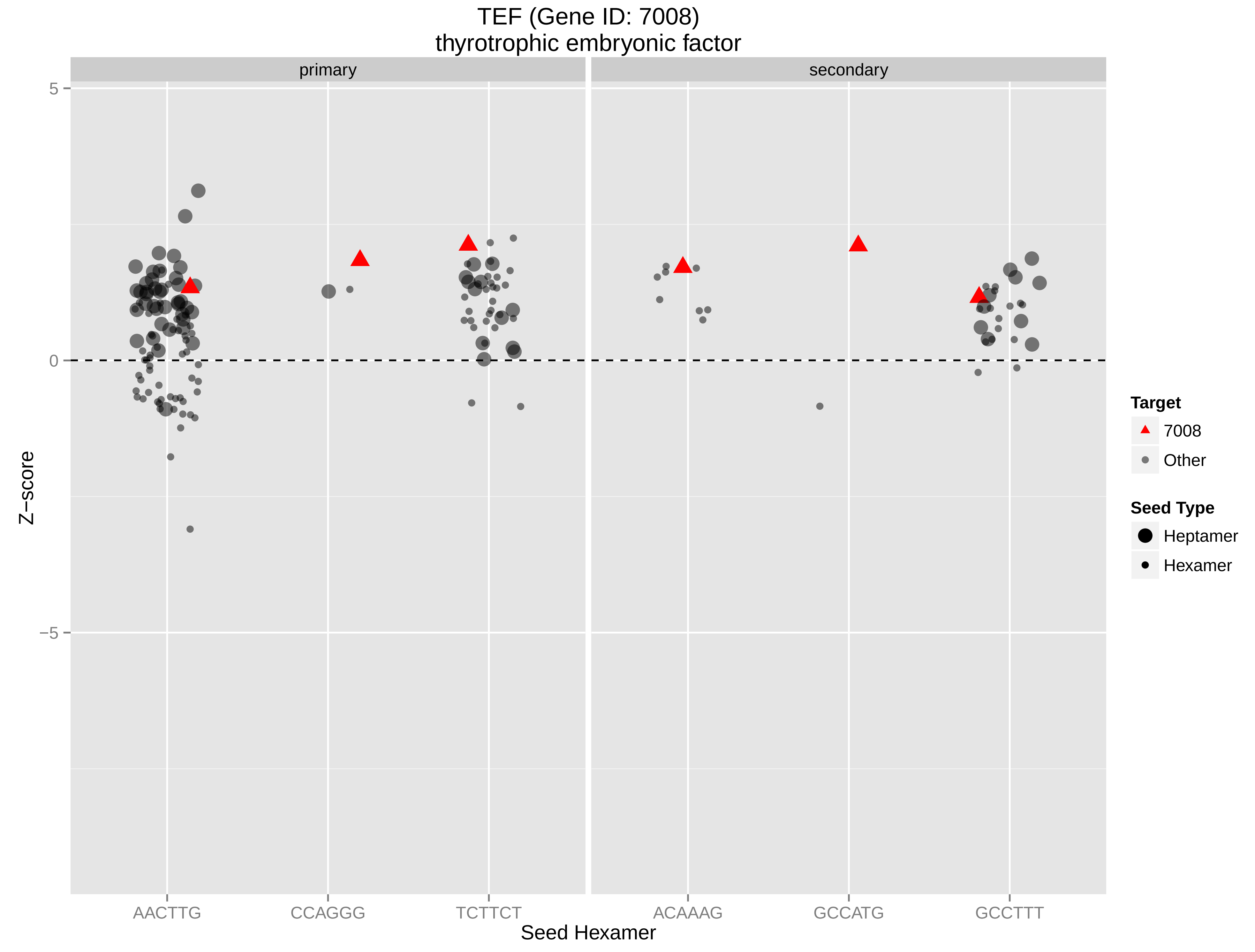
TEF (Gene ID: 7008)
thyrotrophic embryonic factor
primary
secondary
5
0
−5
Z−score
AACTTG
CCAGGG
TCTTCT
ACAAAG
GCCATG
GCCTTT
Seed Hexamer
Target
7008
Other
Seed Type
Heptamer
Hexamer

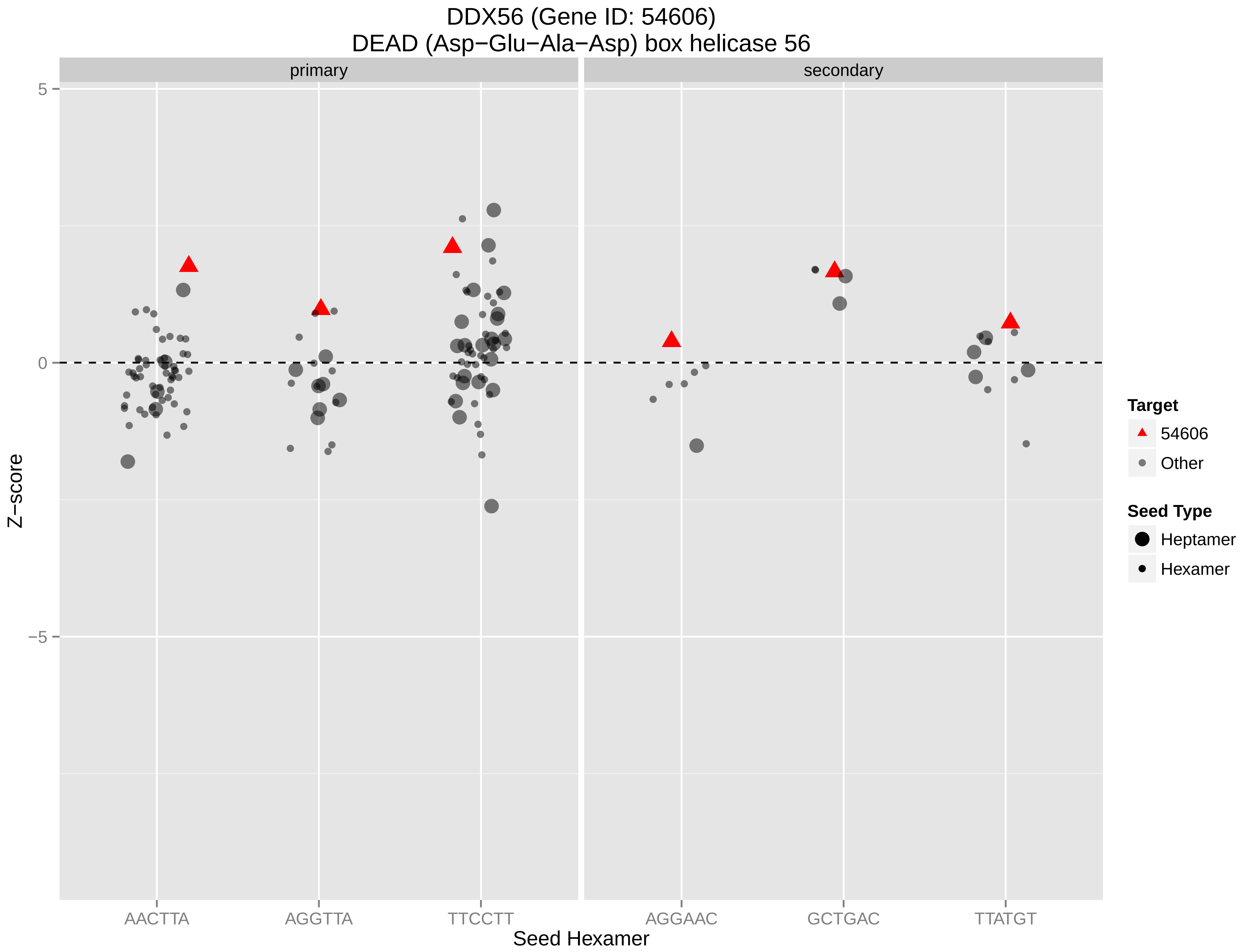
DDX56 (Gene ID: 54606)
DEAD (Asp−Glu−Ala−Asp) box helicase 56
primary
secondary
Z−score
5
0
−5
AACTTA
AGGTTA
TTCCTT
AGGAAC
GCTGAC
TTATGT
Seed Hexamer
Target
54606
Other
Seed Type
Heptamer
Hexamer

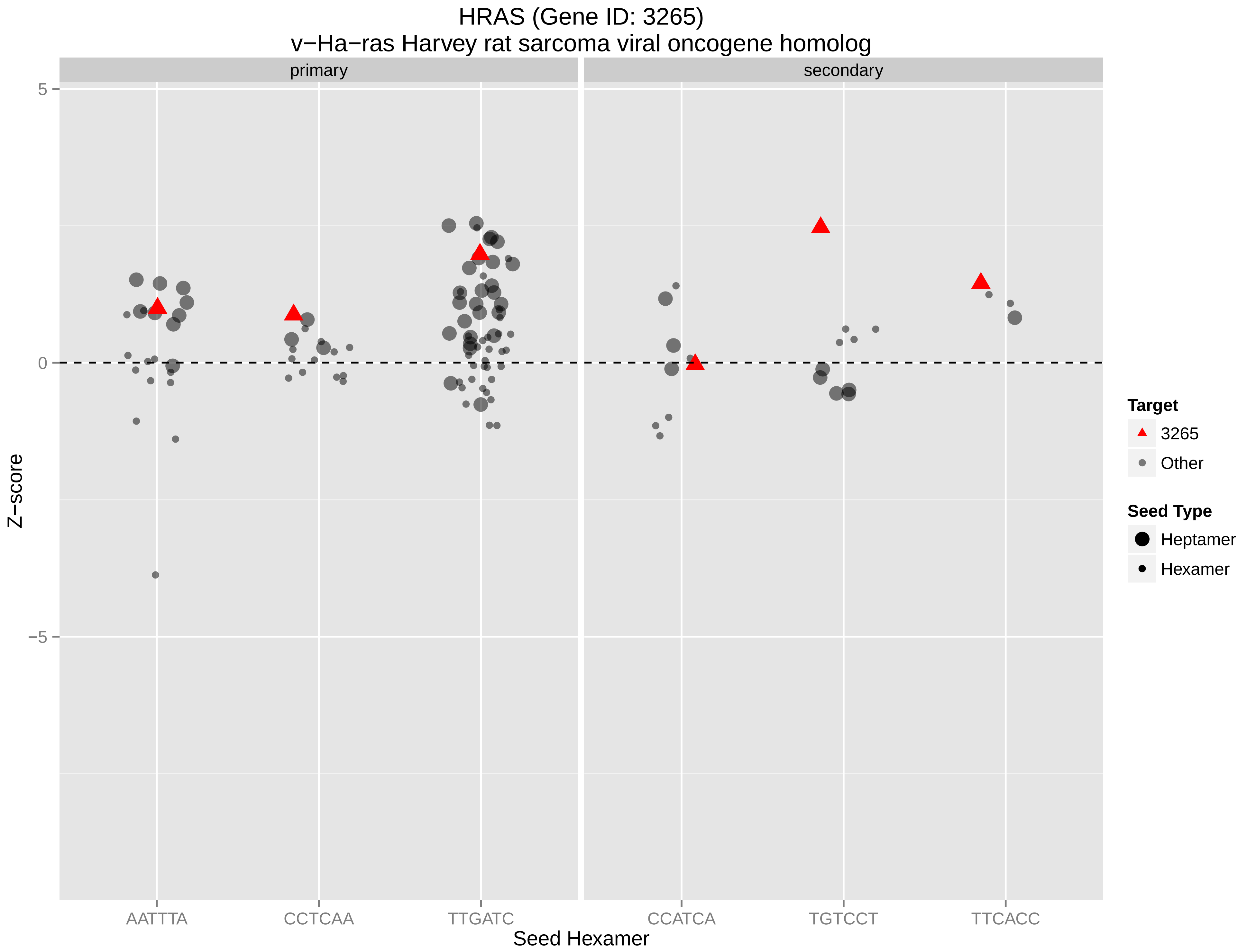
HRAS (Gene ID: 3265)
v−Ha−ras Harvey rat sarcoma viral oncogene homolog
primary
secondary
5
0
−5
Z−score
AATTTA
CCTCAA
TTGATC
CCATCA
TGTCCT
TTCACC
Seed Hexamer
Target
3265
Other
Seed Type
Heptamer
Hexamer

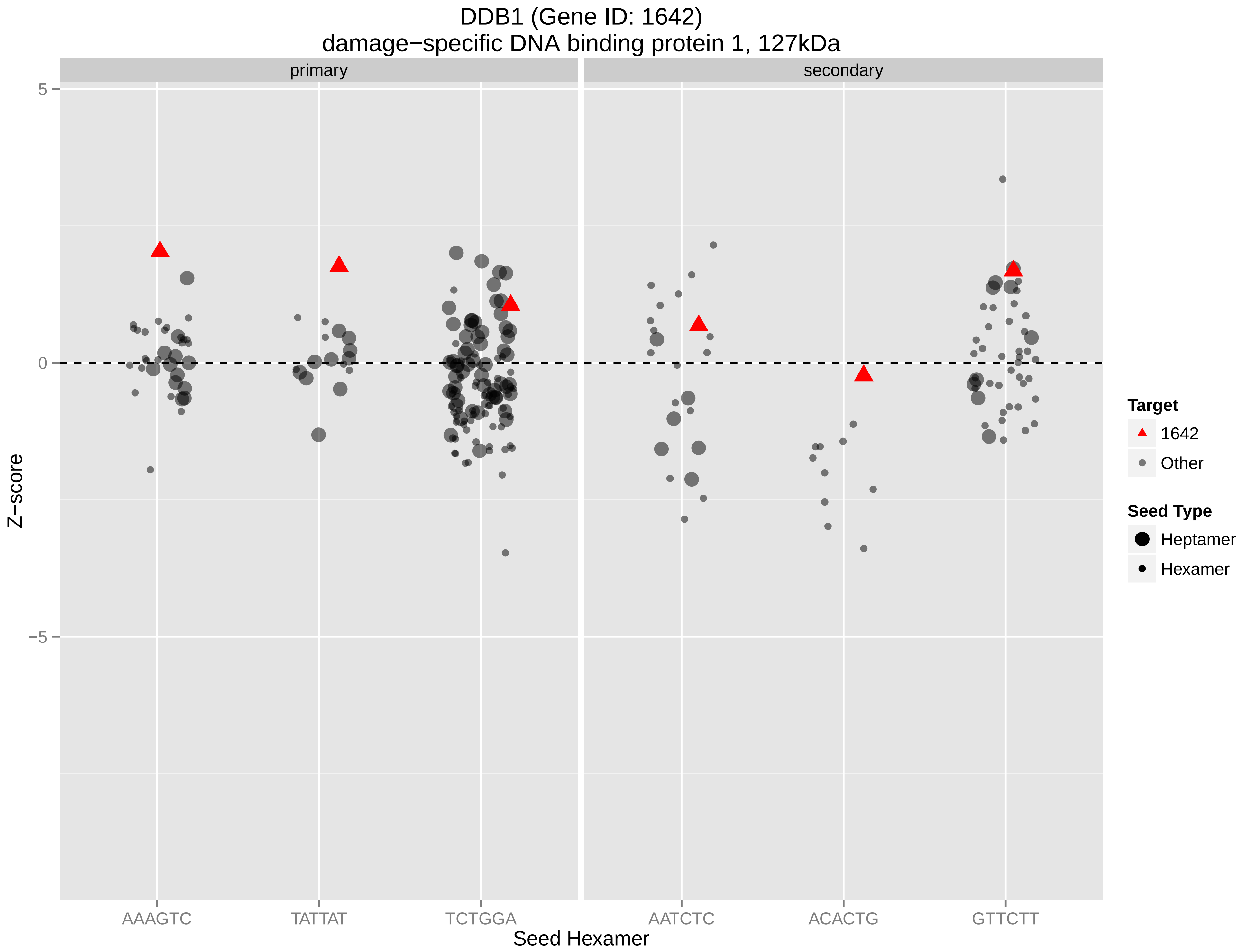

DDB1 (Gene ID: 1642)
damage−specific DNA binding protein 1, 127kDa
primary
secondary
5
0
−5
Z−score
AAAGTC
TATTAT
TCTGGA
AATCTC
ACACTG
GTTCTT
Seed Hexamer
Target
1642
Other
Seed Type
Heptamer
Hexamer

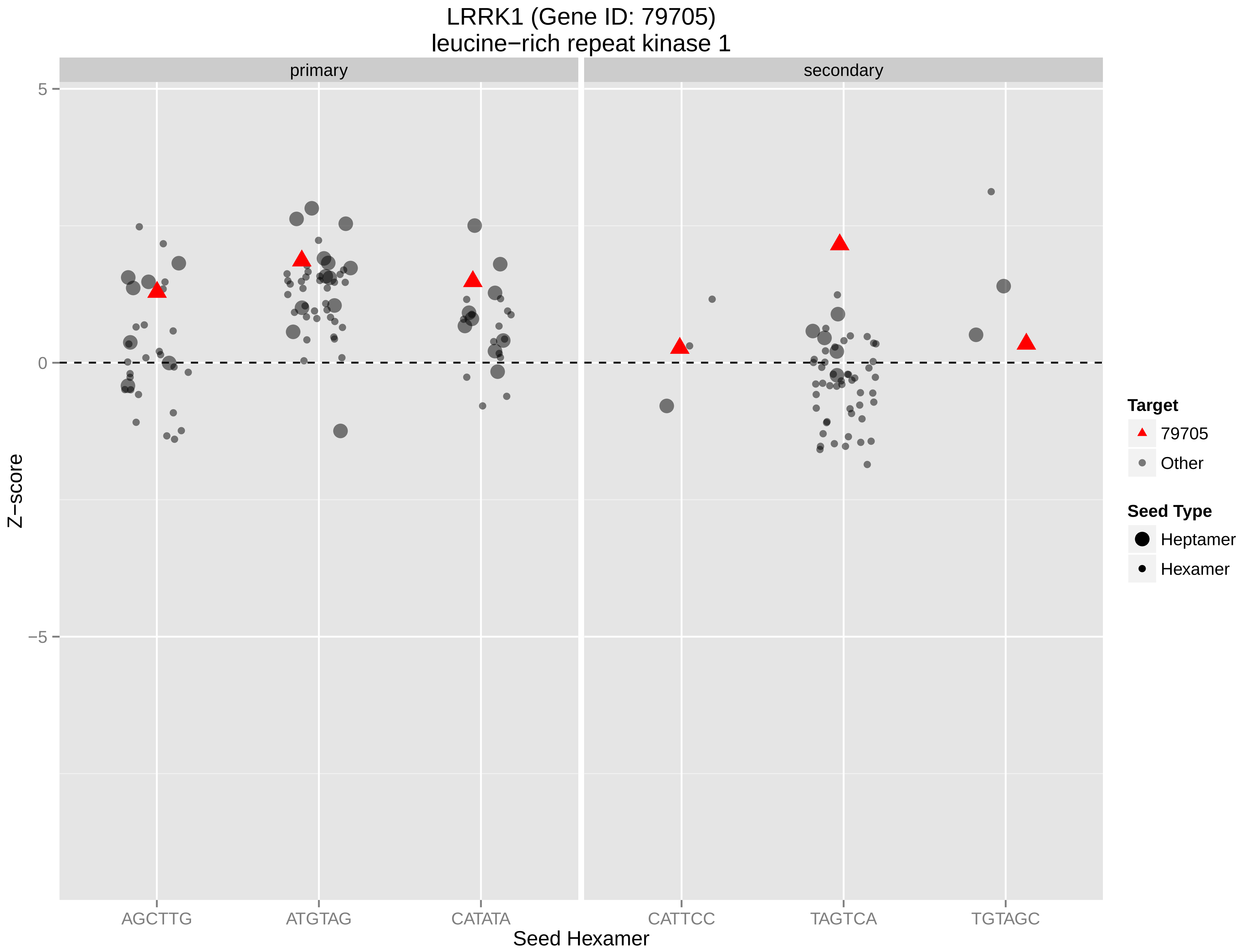
LRRK1 (Gene ID: 79705)
leucine−rich repeat kinase 1
primary
secondary
5
0
−5
Z−score
AGCTTG
ATGTAG
CATATA
CATTCC
TAGTCA
TGTAGC
Seed Hexamer
Target
79705
Other
Seed Type
Heptamer
Hexamer

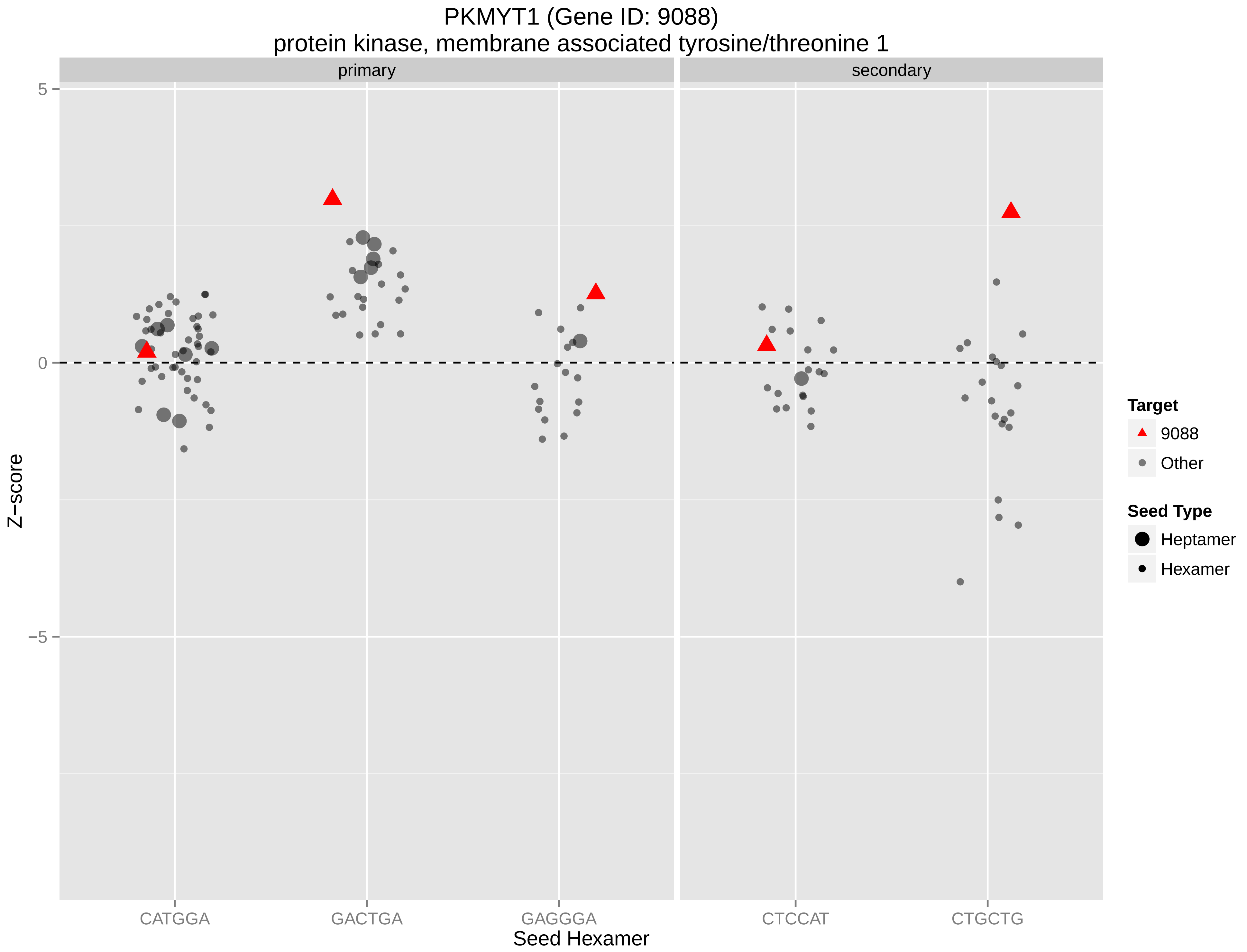

PKMYT1 (Gene ID: 9088)
protein kinase, membrane associated tyrosine/threonine 1
primary
secondary
5
0
−5
Z−score
CATGGA
GACTGA
GAGGGA
CTCCAT
CTGCTG
Seed Hexamer
Target
9088
Other
Seed Type
Heptamer
Hexamer

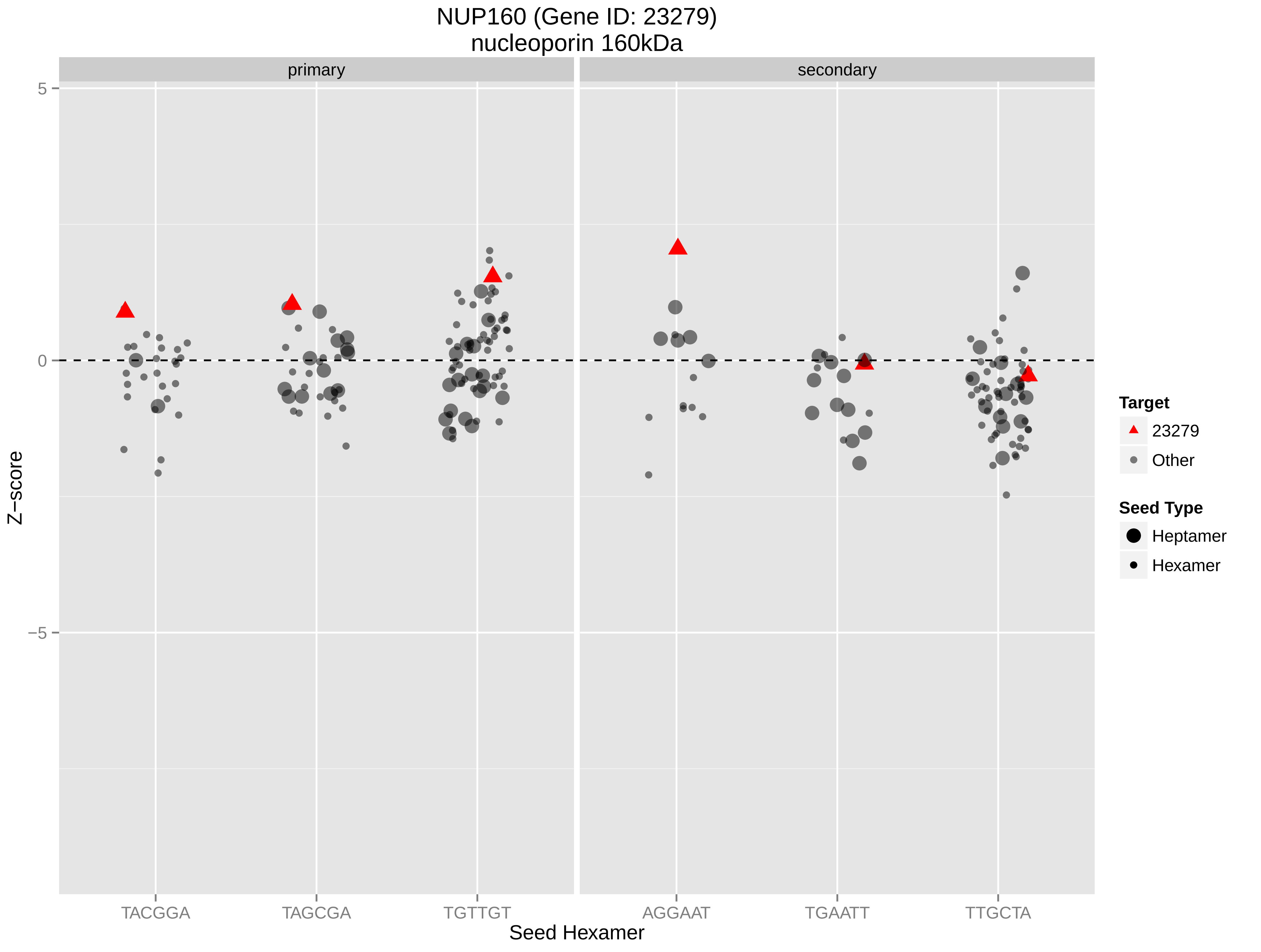
NUP160 (Gene ID: 23279)
nucleoporin 160kDa
primary
secondary
5
0
−5
Z−score
TACGGA
TAGCGA
TGTTGT
AGGAAT
TGAATT
TTGCTA
Seed Hexamer
Target
23279
Other
Seed Type
Heptamer
Hexamer

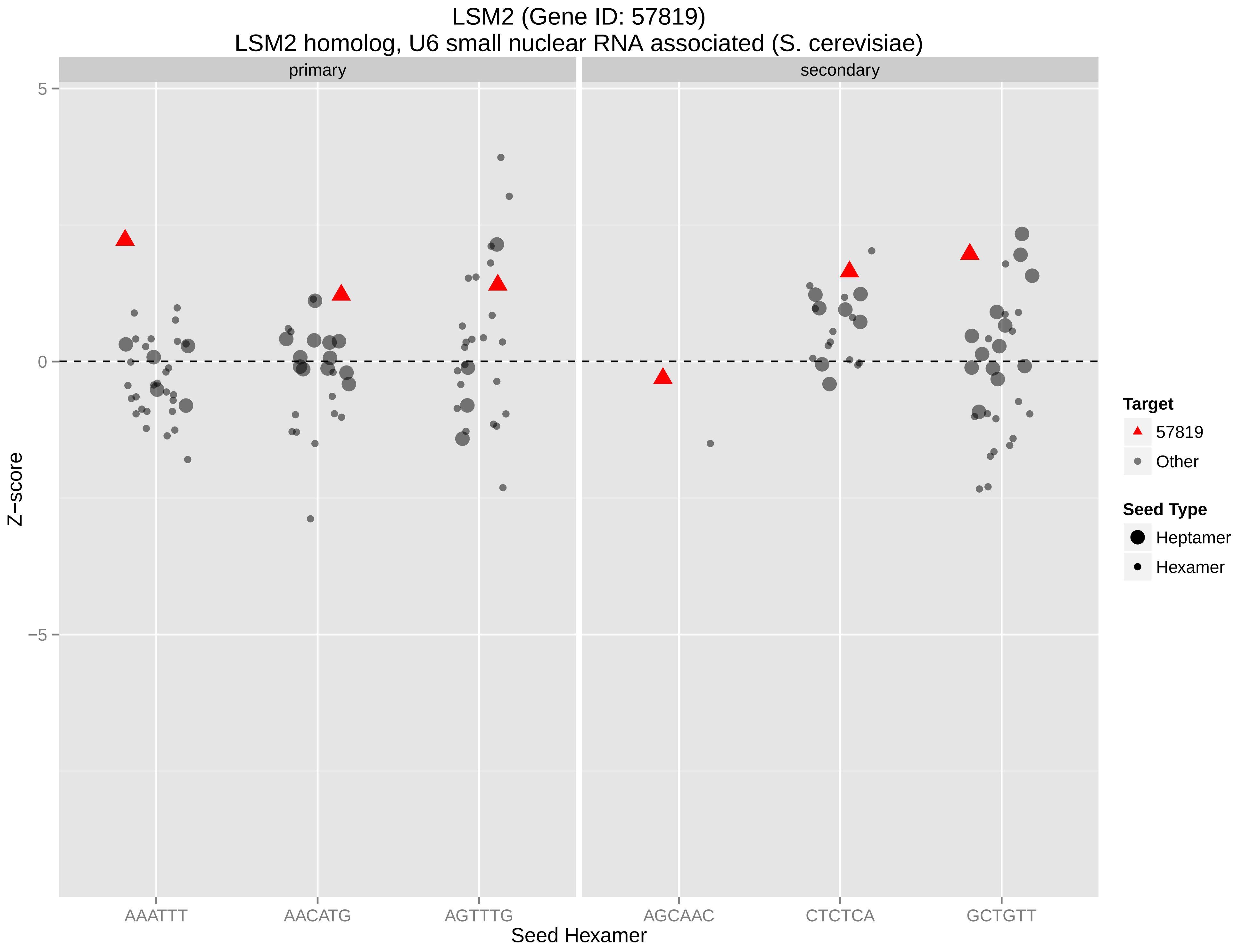

LSM2 (Gene ID: 57819)
LSM2 homolog, U6 small nuclear RNA associated (S. cerevisiae)
primary
secondary
5
0
−5
Z−score
AAATTT
AACATG
AGTTTG
AGCAAC
CTCTCA
GCTGTT
Seed Hexamer
Target
57819
Other
Seed Type
Heptamer
Hexamer

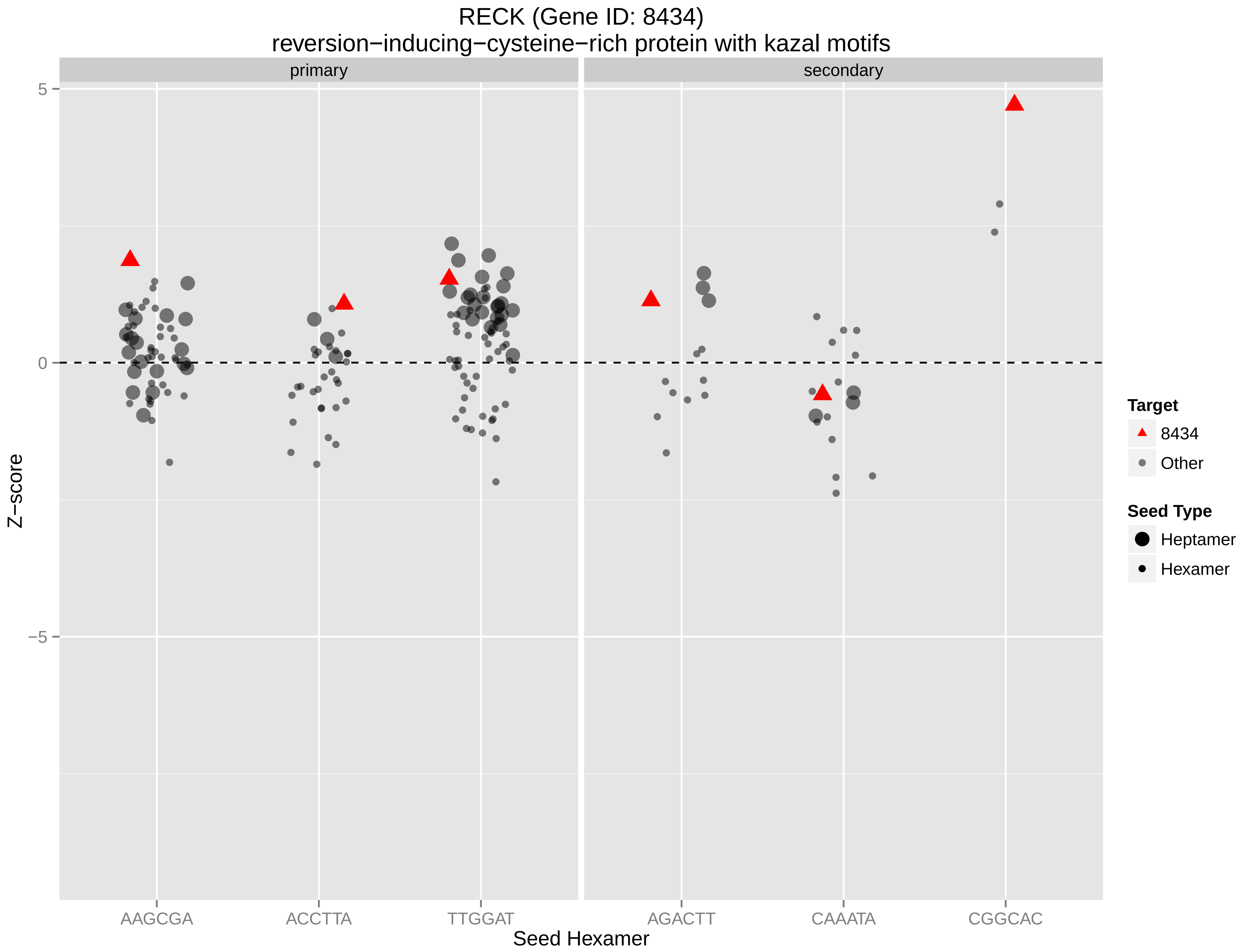

RECK (Gene ID: 8434)
reversion−inducing−cysteine−rich protein with kazal motifs
primary
secondary
5
0
−5
Z−score
AAGCGA
ACCTTA
TTGGAT
AGACTT
CAAATA
CGGCAC
Seed Hexamer
Target
8434
Other
Seed Type
Heptamer
Hexamer

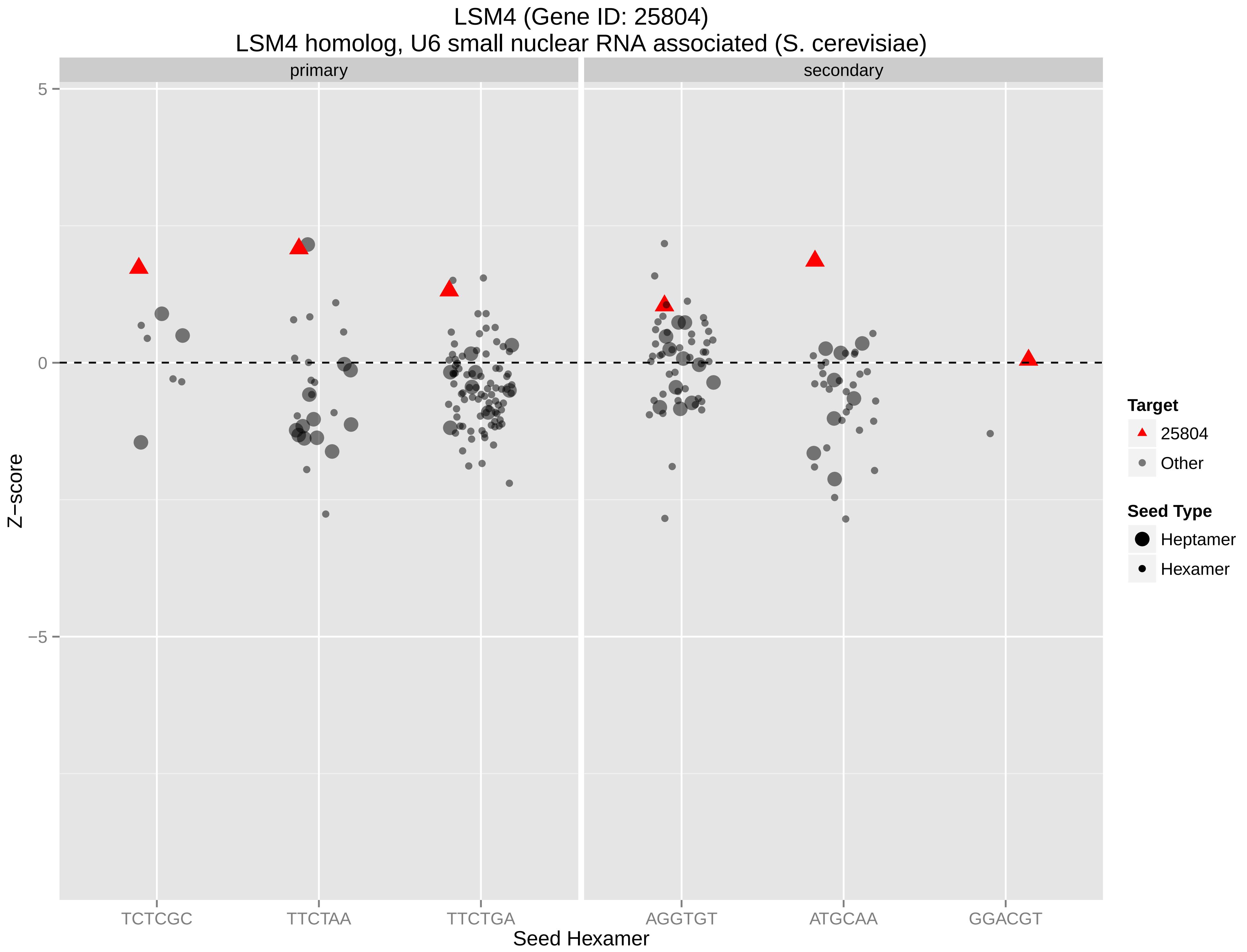
LSM4 (Gene ID: 25804)
LSM4 homolog, U6 small nuclear RNA associated (S. cerevisiae)
primary
secondary
5
0
−5
Z−score
TCTCGC
TTCTAA
TTCTGA
AGGTGT
ATGCAA
GGACGT
Seed Hexamer
Target
25804
Other
Seed Type
Heptamer
Hexamer

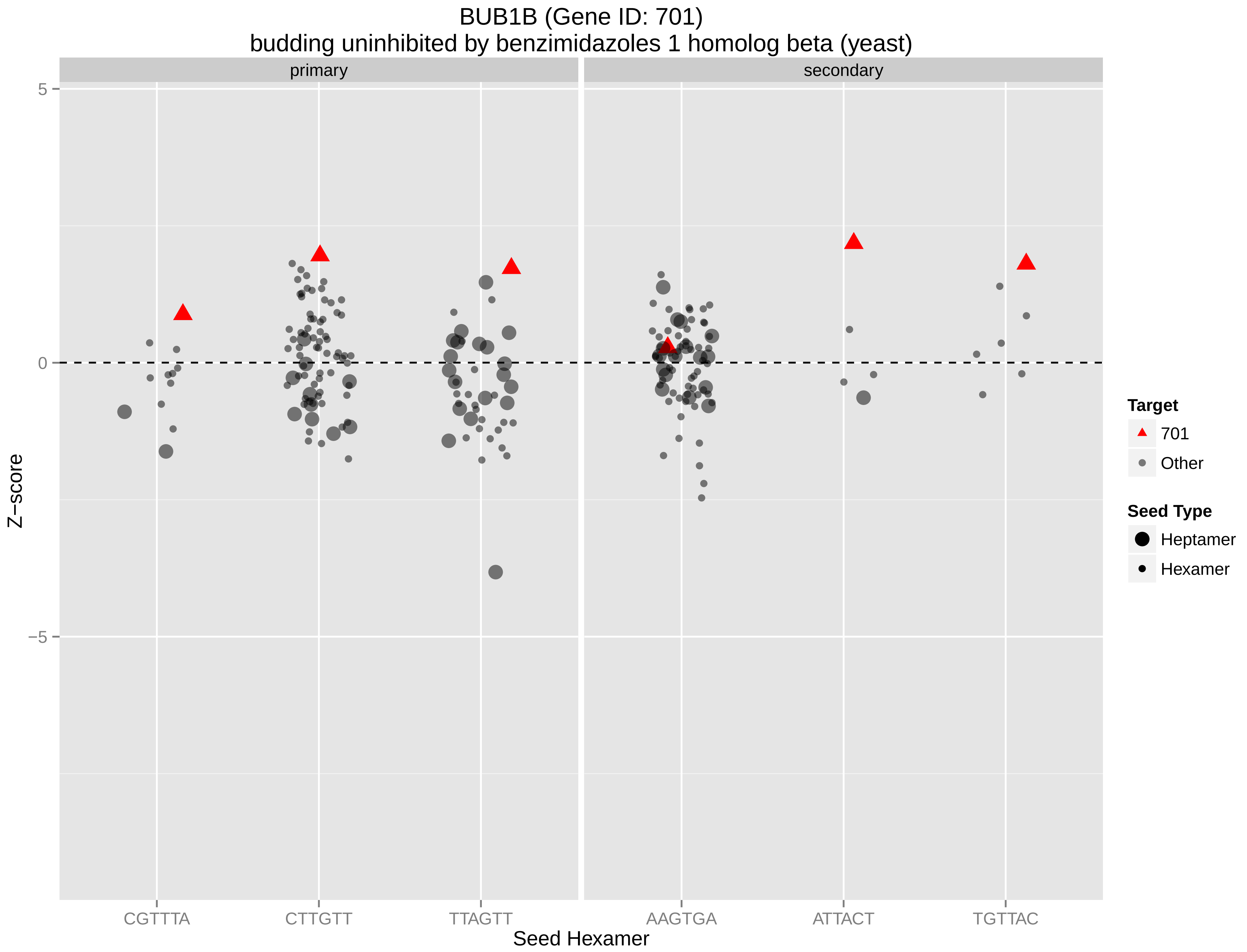
BUB1B (Gene ID: 701)
budding uninhibited by benzimidazoles 1 homolog beta (yeast)
primary
secondary
5
0
−5
Z−score
CGTTTA
CTTGTT
TTAGTT
AAGTGA
ATTACT
TGTTAC
Seed Hexamer
Target
701
Other
Seed Type
Heptamer
Hexamer

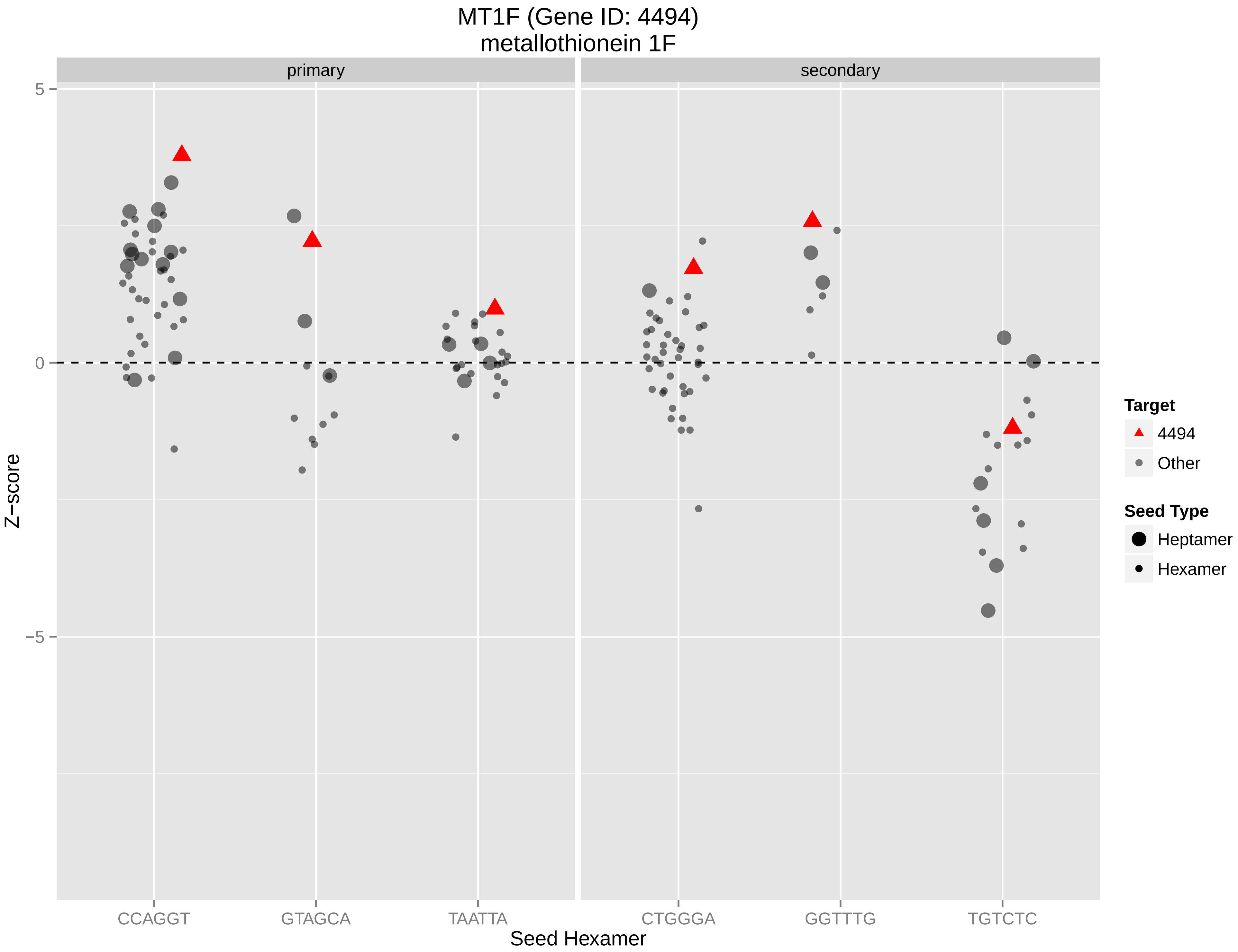
MT1F (Gene ID: 4494)
metallothionein 1F
primary
secondary
5
0
−5
Z−score
CCAGGT
GTAGCA
TAATTA
CTGGGA
GGTTTG
TGTCTC
Seed Hexamer
Target
4494
Other
Seed Type
Heptamer
Hexamer

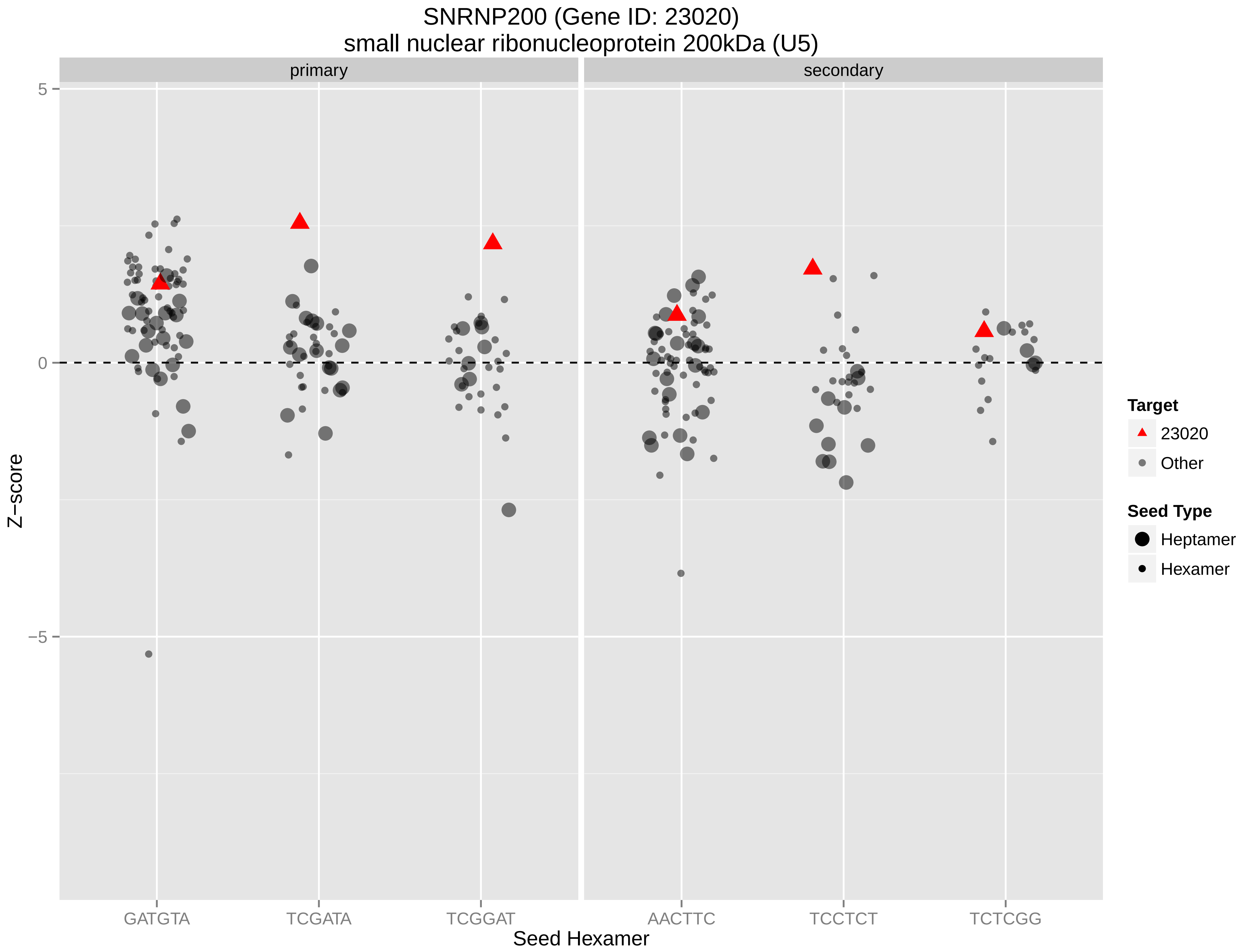

SNRNP200 (Gene ID: 23020)
small nuclear ribonucleoprotein 200kDa (U5)
primary
secondary
5
0
−5
Z−score
GATGTA
TCGATA
TCGGAT
AACTTC
TCCTCT
TCTCGG
Seed Hexamer
Target
23020
Other
Seed Type
Heptamer
Hexamer

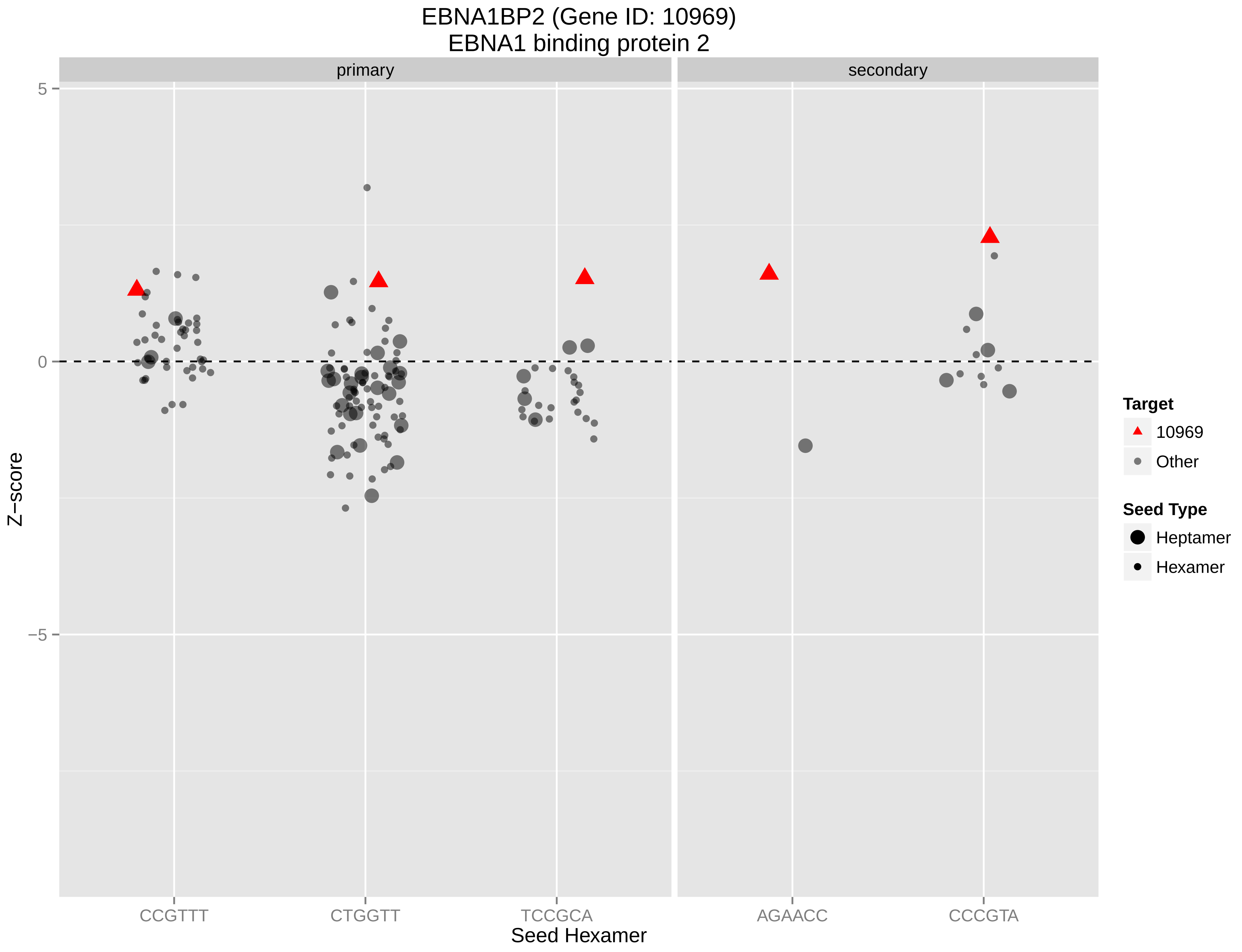

EBNA1BP2 (Gene ID: 10969)
EBNA1 binding protein 2
primary
secondary
5
0
−5
Z−score
CCGTTT
CTGGTT
TCCGCA
AGAACC
CCCGTA
Seed Hexamer
Target
10969
Other
Seed Type
Heptamer
Hexamer

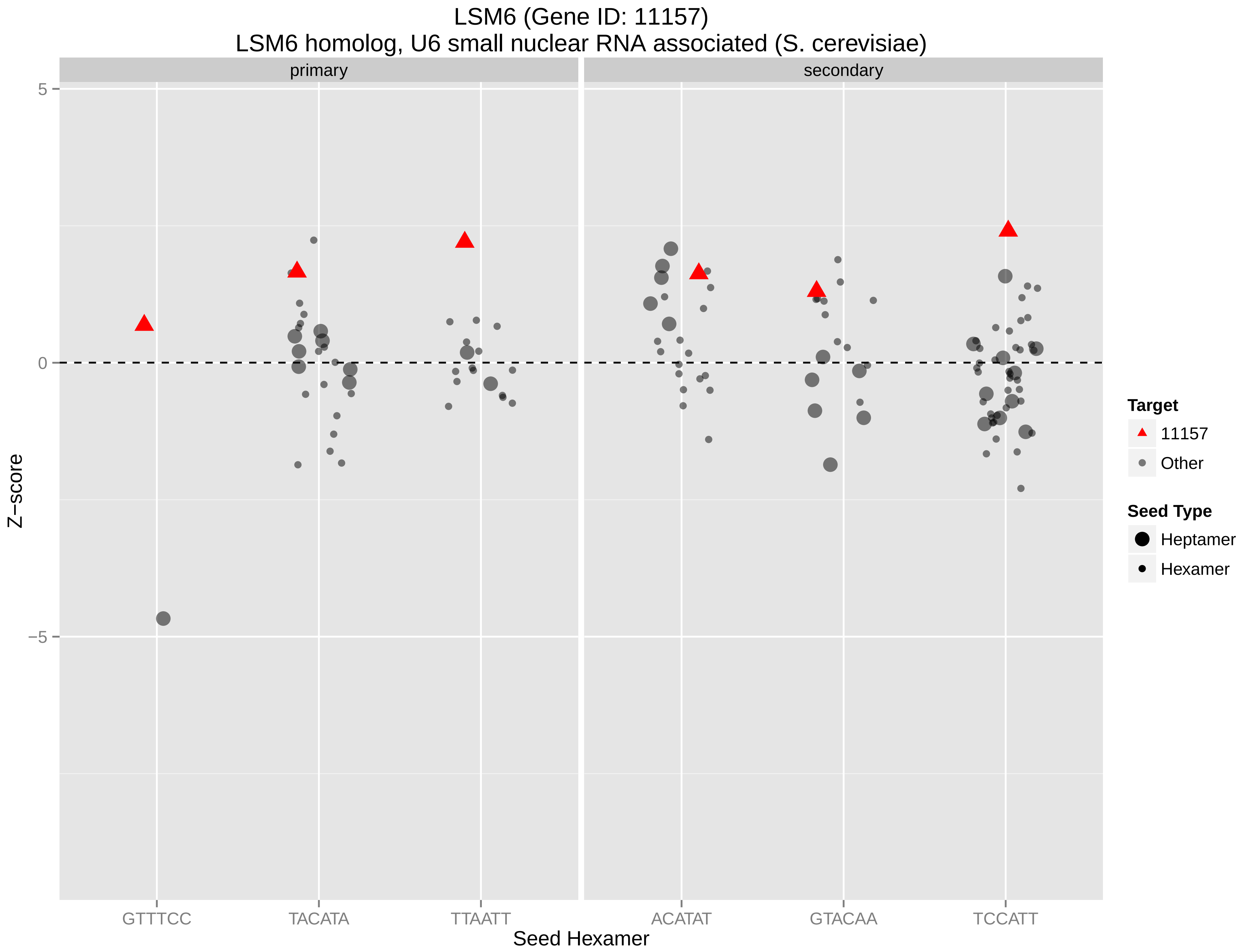
LSM6 (Gene ID: 11157)
LSM6 homolog, U6 small nuclear RNA associated (S. cerevisiae)
primary
secondary
5
0
−5
Z−score
GTTTCC
TACATA
TTAATT
ACATAT
GTACAA
TCCATT
Seed Hexamer
Target
11157
Other
Seed Type
Heptamer
Hexamer

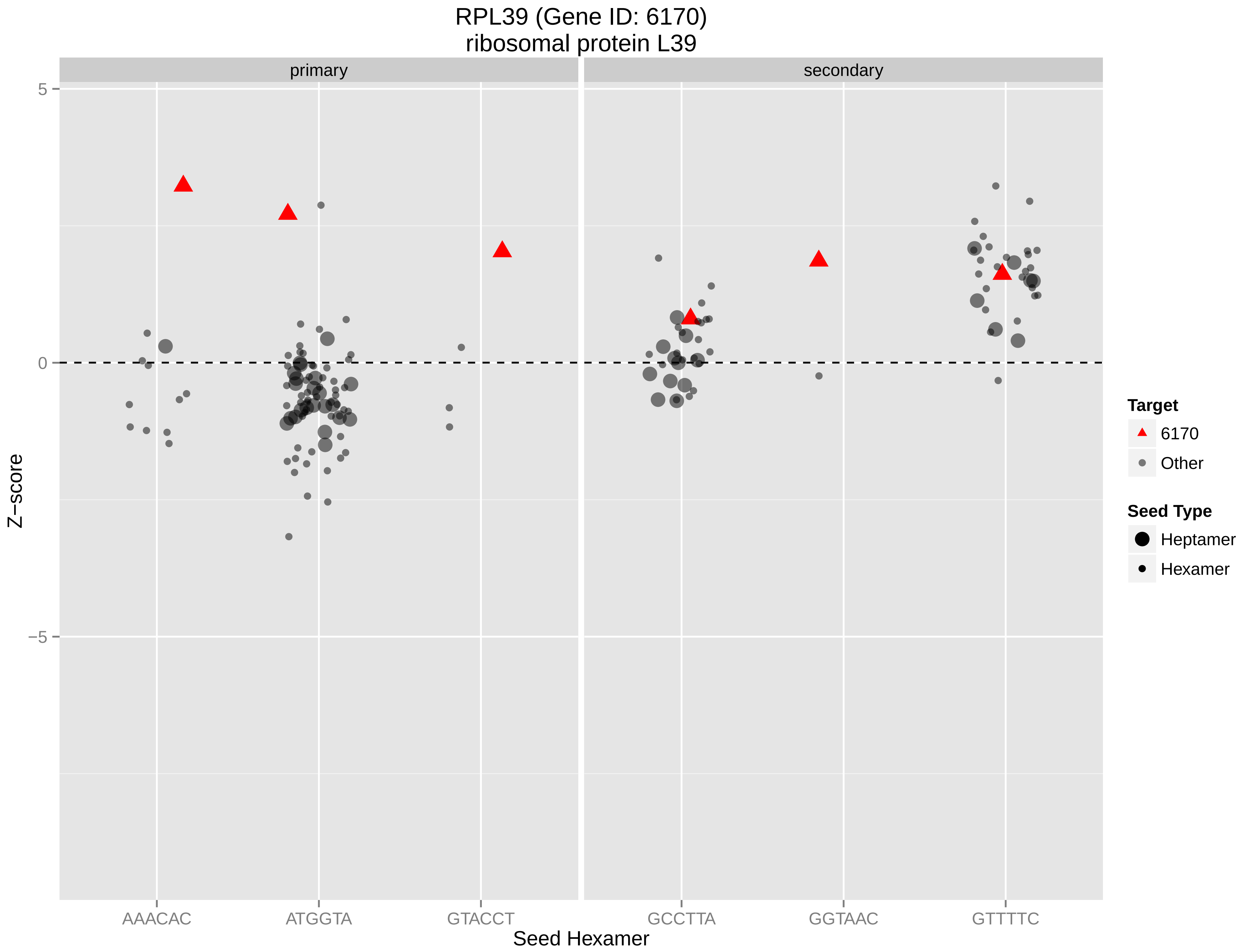

RPL39 (Gene ID: 6170)
ribosomal protein L39
primary
secondary
5
0
−5
Z−score
AAACAC
ATGGTA
GTACCT
GCCTTA
GGTAAC
GTTTTC
Seed Hexamer
Target
6170
Other
Seed Type
Heptamer
Hexamer

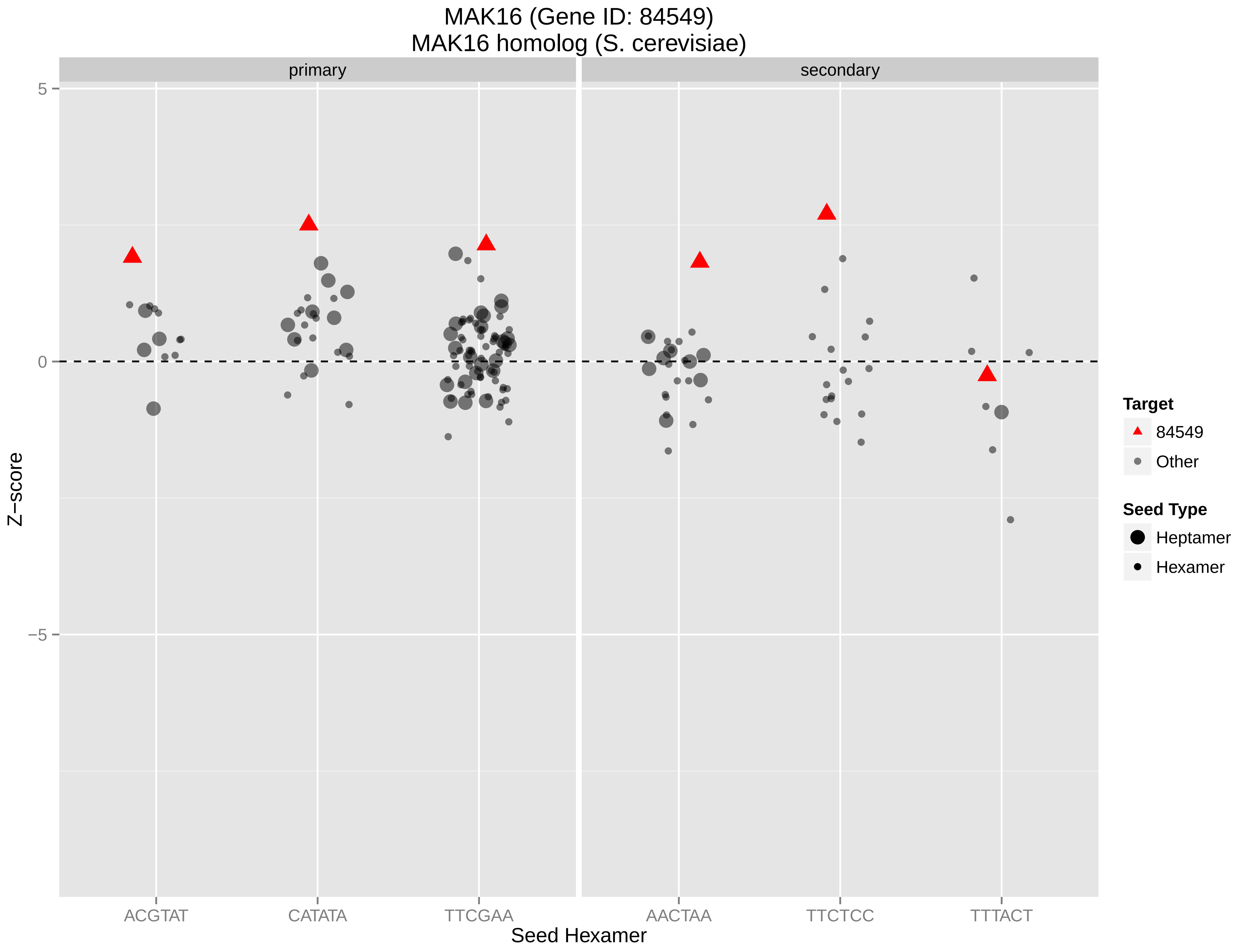

MAK16 (Gene ID: 84549)
MAK16 homolog (S. cerevisiae)
primary
secondary
5
0
−5
Z−score
ACGTAT
CATATA
TTCGAA
AACTAA
TTCTCC
TTTACT
Seed Hexamer
Target
84549
Other
Seed Type
Heptamer
Hexamer

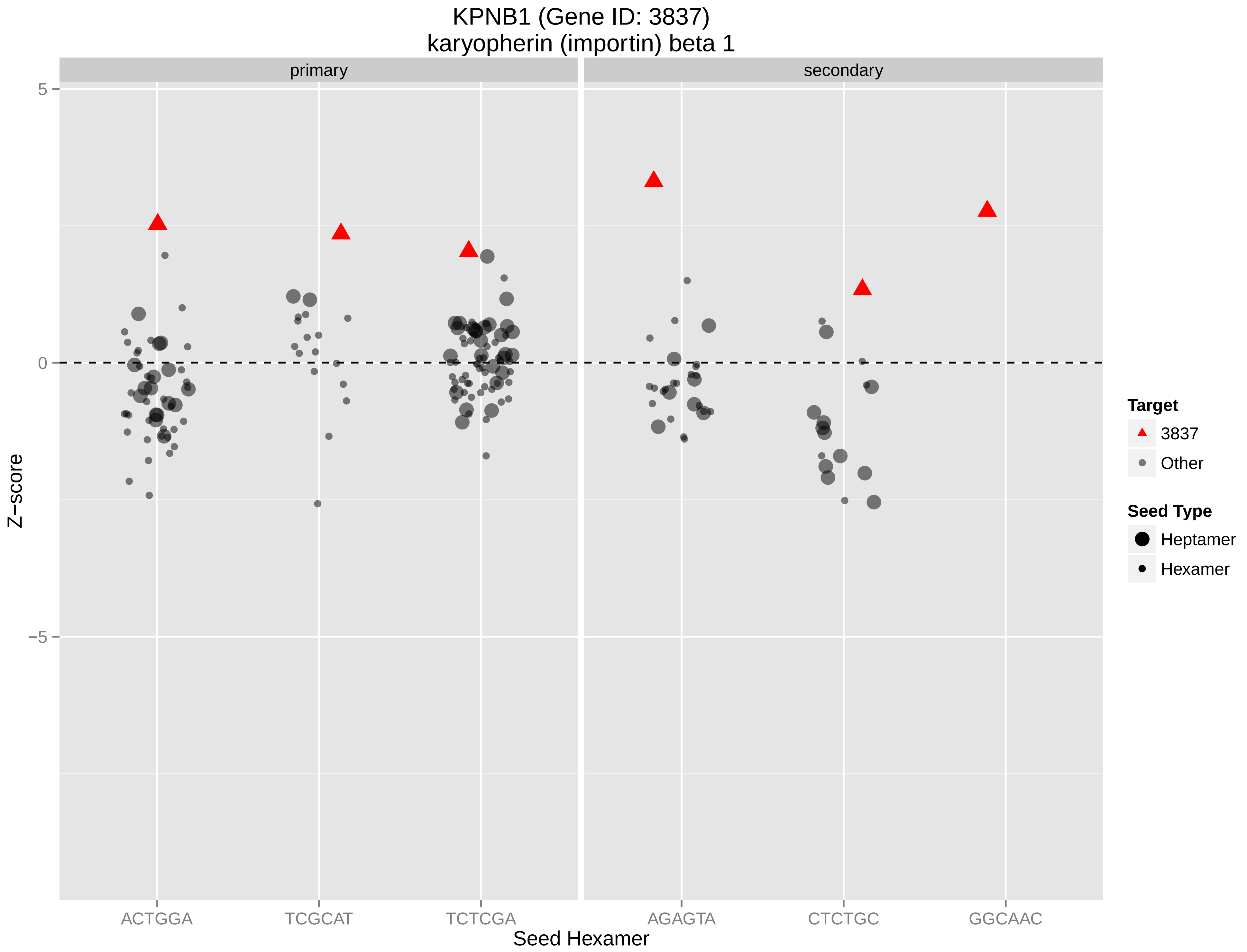
KPNB1 (Gene ID: 3837)
karyopherin (importin) beta 1
primary
secondary
5
0
−5
Z−score
ACTGGA
TCGCAT
TCTCGA
AGAGTA
CTCTGC
GGCAAC
Seed Hexamer
Target
3837
Other
Seed Type
Heptamer
Hexamer

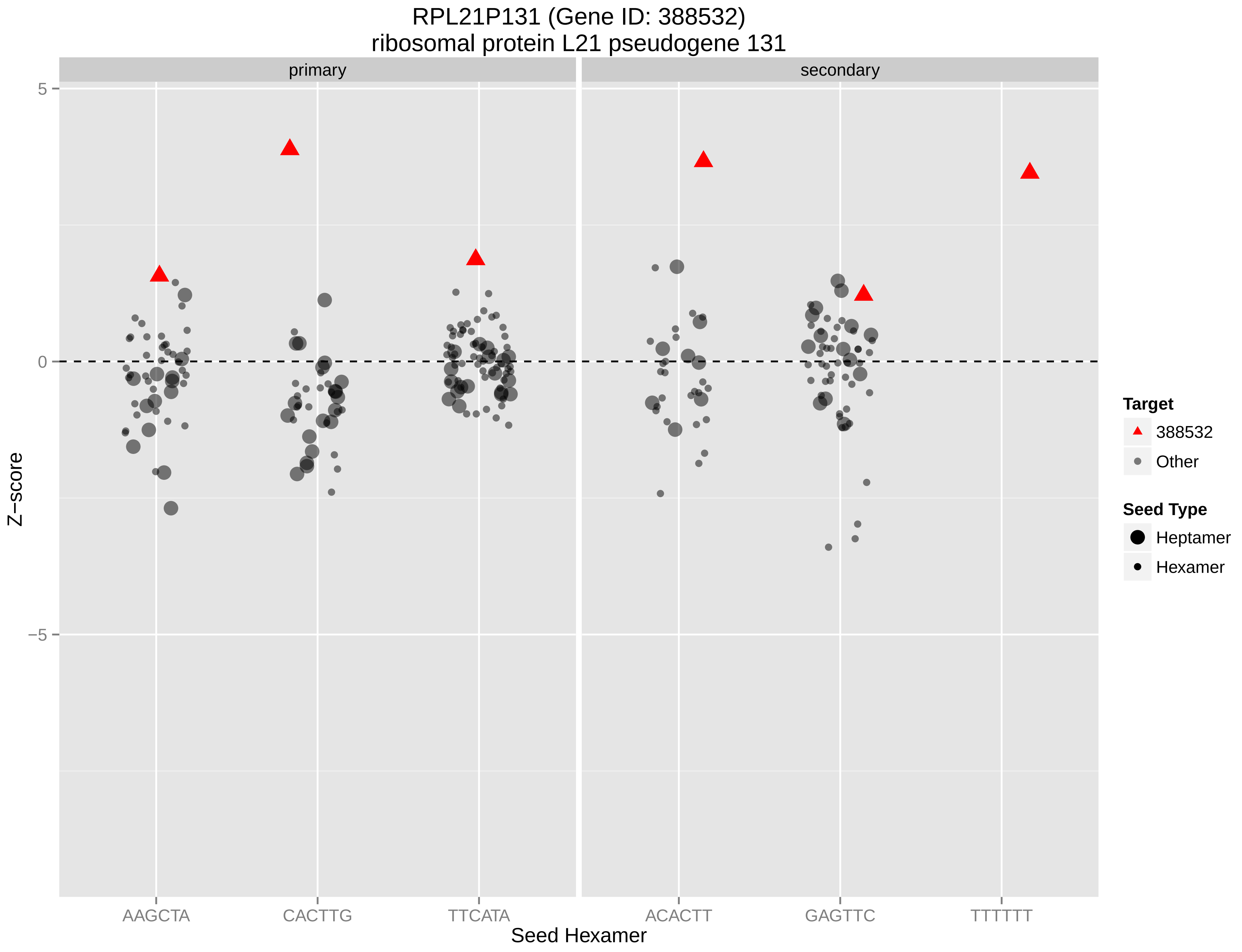

RPL21P131 (Gene ID: 388532)
ribosomal protein L21 pseudogene 131
primary
secondary
5
0
−5
Z−score
AAGCTA
CACTTG
TTCATA
ACACTT
GAGTTC
TTTTTT
Seed Hexamer
Target
388532
Other
Seed Type
Heptamer
Hexamer
